# Reciprocal regulation of MMP-28 and EGFR is required for sustaining proliferative signaling in PDAC

**DOI:** 10.1186/s13046-025-03323-9

**Published:** 2025-02-24

**Authors:** Zhengtao Hong, Xing Huang, Linghao Xia, Tingbo Liang, Xueli Bai

**Affiliations:** 1 Department of Hepatobiliary and Pancreatic Surgery, the First Affiliated Hospital, Zhejiang University School of Medicine, Hangzhou, 310003 China; 2 Zhejiang Provincial Key Laboratory of Pancreatic Disease, the First Affiliated Hospital, Zhejiang University School of Medicine, Hangzhou, 310003 China; 3 Clinical Research Center of Hepatobiliary and Pancreatic Diseases, Zhejiang Province, Hangzhou, 31003 China; 4 The Innovation Center for the Study of Pancreatic Diseases of Zhejiang Province, Hangzhou, 310003 China; 5 Zhejiang University Cancer Center, Hangzhou, 310063 China; 6 MOE Joint International Research Laboratory of Pancreatic Diseases, Hangzhou, 310003 China

**Keywords:** Pancreatic ductal adenocarcinoma, Proliferation signaling, MMP-28, EGFR, TGF-α

## Abstract

**Backgroud:**

Sustaining proliferation signaling is the top hallmarks of cancer, driving continuous tumor growth and resistance to drug treatments. Blocking proliferation signaling has shown limited benefit in clinical treatment of pancreatic ductal adenocarcinoma, highlighting the urgent need to deeply understand proliferation signaling and develop new therapeutic strategies.

**Methods:**

By leveraging clinical data and data from the TCGA and GDSC datasets, we investigated the association between MMP-28 expression and the sensitivity to EGFR inhibitors as well as the prognosis of PDAC. Transcriptomic and biological experiments explore the regulatory role of MMP-28 on the EGFR signaling pathway. Additionally, in vitro and in vivo studies are employed to evaluate MMP-28 as a biomarker for sensitivity to EGFR inhibitors.

**Results:**

We found that MMP-28, a metalloproteinase, was significantly associated with the sensitivity to EGFR inhibitors. Furthermore, MMP-28 could promote PDAC growth and metastasis. Mechanistically, MMP-28 facilitated the maturation and release of the TGF-α precursor, thus promoting EGFR activation. In return, EGFR upregulated MMP-28 through AP-1-mediated transcription, forming a positive feedback loop that provided sustaining proliferation signaling for PDAC. Subsequently, MMP-28 was identified to predict the response to EGFR inhibitors and recognize responsive patients.

**Conclusions:**

Our findings revealed the role of MMP-28 and EGFR in generation of sustaining proliferation signaling and provided a new therapy strategy for PDAC.

**Supplementary Information:**

The online version contains supplementary material available at 10.1186/s13046-025-03323-9.

## Introduction

Pancreatic ductal adenocarcinoma (PDAC) is responsible for approximately 250,000 deaths per year and ranks as the sixth leading cause of cancer-related deaths [1]. At diagnosis, only 10% to 15% of PDAC patients have localized disease suitable for surgical intervention. About 30% to 35% have locally advanced disease, which is often unresectable due to extensive tumor-vascular involvement. The remaining 50% present with metastatic disease, for which only non-surgical treatments are available [2, 3]. Current standard first-line treatment options for advanced PDAC include gemcitabine combined with albumin-bound paclitaxel or FOLFIRINOX (5-FU, leucovorin, irinotecan, oxaliplatin) [3, 4]. In a phase III clinical trial for metastatic PDAC, adding albumin-bound paclitaxel to gemcitabine improved median overall survival (OS) from 6.7 to 8.5 months (HR 0.72, 95% CI 0.62–0.83; *p* < 0.001) [5]. Another trial for metastatic PDAC showed that the median OS was 11.1 months in the FOLFIRINOX group compared to 6.8 months in the gemcitabine group (HR 0.57, 95% CI 0.45–0.73; *p* < 0.001) [6, 7]. Besides cytotoxic regimens, emerging treatment strategies like immunotherapy and targeted therapy are currently hot topics in PDAC research. However, PDAC has long been considered resistant to immunotherapy. A phase II trial of durvalumab (a PD-1 inhibitor) with or without tremelimumab (a CTLA-4 inhibitor) revealed the overall response rate (ORR) of only 3.1% (95% CI 0.08–16.22) for combination therapy and 0% (95% CI 0.00–10.58) for monotherapy [8]. As for targeted therapy, taking the KrasG12C inhibitors as an example, although achieving promising results in a multicohort phase I-II study (with an ORR of 33.3%), yet only about 1% of PDAC patients benefited [9]. Overall, current non-surgical treatments offer limited benefits to PDAC patients. There is an urgent need for new therapeutic targets, and innovative concepts in treatment may be more crucial.

Sustaining proliferative signaling is the first of ten hallmarks in cancer, but it is always ignored in treatment [10]. In clinic, the focus is frequently on how to kill tumor cells, such as using cytotoxic drugs to directly destroy tumor cells or employing immune stimulants to activate immune cells to indirectly eliminate tumor cells. However, according to Gompertzian growth curves and Norton–Simon hypothesis, drug treatment leads to a reduction in tumor burden, but followed by rapid growth of residual lesions [11–14]. Based on the above theory and phenomena, we propose the concept that tumor cells may evade or resist drug treatment by proliferating faster than be destroyed. Therefore, we believe that blocking sustaining proliferative signaling could be a crucial strategy for overcoming drug resistance.

Receptor tyrosine kinase (RTK) inhibitors are the most commonly used in clinic for blocking proliferation signaling. RTKs are the largest class of enzyme-linked receptors including EGFR, PDGFR, IGFR, VEGFR, and so on [15, 16]. They not only function as high-affinity cell surface receptors for growth factors but also activate a series of signaling cascades that promote cancer progression [17]. RTKs inhibitors have also been extensively tested in PDAC. Clinical trials of PDGFR, IGFR, and VEGFR inhibitors have all ended in failure [18]. Other strategies for blocking proliferation signaling, such as inhibitors targeting MEK, mTOR, and PI3K, still require further clinical research in PDAC [19–21]. But fortunately, the epidermal growth factor receptor (EGFR) inhibitor Erlotinib has shown promising potential and were approved by Food and Drug Administration (FDA) for PDAC. Median overall survival was 6.24 months in the gemcitabine-Erlotinib group compared with 5.91 months in the gemcitabine group (HR 0.82, 95% CI 0.69–0.99; *p* = 0.038) [22]. Despite some benefits, there are reports that Erlotinib resulted in increased grade 3/4 toxicity, making it unsuitable for first-line treatment [18]. Overall, the current approaches for blocking proliferation signaling are not yet fully developed for PDAC, possibly due to unresolved key mechanisms. Therefore, it is crucial to identify and elucidate the key mechanisms of sustaining proliferative signaling in PDAC and to assess their potential as therapeutic targets.

In this study, we found that Matrix Metalloproteinase 28 (MMP-28) was not only associated with the sensitivity to Erlotinib but also highly expressed in PDAC and related to poor prognosis. Further researches demonstrated that MMP-28 promotes PDAC growth and metastasis both in vivo and in vitro. Mechanistic studies revealed that MMP-28 promoted the progression of PDAC by EGFR activation. Subsequently, we discovered that MMP-28 enhances EGFR activation and progression of PDAC by facilitating the maturation and release of transforming growth factor-α (TGF-α). Interestingly, we found that EGFR, in turn, upregulated MMP-28 expression at transcription level through extracellular regulated protein kinases (ERK) and activated protein-1 (AP-1). Thus, we identified a positive feedback loop providing sustaining proliferative signaling in PDAC, consisting of MMP-28-TGF-α-EGFR-AP-1. Next, both in vitro and in vivo experiments revealed that PDAC cells with high MMP-28 expression exhibit increased sensitivity to Erlotinib. Finally, the patient-derived tumor xenograft (PDX) model confirmed that MMP-28 is a valuable biomarker for Erlotinib therapy. Overall, this study found that the reciprocal regulation of MMP-28 and EGFR is essential for sustaining proliferative signaling in PDAC, and the expression level of MMP-28 determines the therapeutic efficacy of EGFR inhibitors in PDAC.

## Materials and methods

### Antibodies, reagents, kits, plasmid and Ribonucleic Acid (RNA) interference

Antibodies: MMP-28 antibody (18,237–1-AP, Proteintech, 1:1000 for immunoblotting, 1:100 for IHC, IP, Duo-link, mIHC), Glyceraldehyde-3-phosphate Dehydrogenase (GAPDH) antibody (AF0006, Beyotime, 1:1000), c-JUN antibody (9165, CST, 1:1000 for immunoblotting, 1:100 for ChIP), Phospho-c-Jun (Ser73) antibody (3270, CST, 1:1000 for immunoblotting), c-FOS antibody (2250, CST, 1:1000 for immunoblotting, 1:100 for ChIP), Phospho-c-Fos (Ser32) antibody (9165, CST, 1:1000 for immunoblotting), EGFR antibody (4267, CST, 1:1000 for immunoblotting, 1:100 for IP), Phospho-EGF Receptor (Tyr1173) antibody (4407, CST, 1:1000 for immunoblotting, 1:100 for mIHC), Phospho-EGF Receptor (Tyr1068) antibody (3777, CST, 1:1000 for immunoblotting, 1:100 for mIHC), STAT1 antibody (14,994, CST, 1:1000 for immunoblotting), Phospho-STAT1 (Tyr701) antibody (9167, CST, 1:1000 for immunoblotting), STAT3 antibody (9139, CST, 1:1000 for immunoblotting), Phospho-Stat3 (Tyr705) antibody (9145, CST, 1:1000 for immunoblotting), STAT5 antibody (94,205, CST, 1:1000 for immunoblotting), Phospho-STAT5 (Tyr694) antibody (9359, CST, 1:1000 for immunoblotting), ERK1/2 antibody (4695, CST, 1:1000 for immunoblotting), Phospho-ERK1/2 (Thr202/Thr185) antibody (ab201015, Abcam, 1:1000 for immunoblotting), TGF-α antibody (ab227723, Abcam, 1:1000 for immunoblotting, 1:100 for IP), Epidermal Growth Factor (EGF) antibody (ab206423, Abcam, 1:1000 for immunoblotting), Heparin-binding EGF-like Growth Factor (HB-EGF) antibody (PA5-143,862, Invitrogen, 1:1000 for immunoblotting), c-Myc antibody (ab32072, Abcam, 1:1000 for immunoblotting), PIM1 antibody (ab300453, Abcam, 1:1000 for immunoblotting), B-cell Lymphoma 2-like 1 (BCL2L1) antibody (ab32370, Abcam, 1:1000 for immunoblotting), Vascular Endothelial Growth Factor (VEGF) antibody (ab214424, Abcam, 1:1000 for immunoblotting), MEK1/2 antibody (ab178876, Abcam, 1:1000 for immunoblotting), MEK1/2 (phospho S222) antibody (ab4750, Abcam, 1:1000 for immunoblotting), HRP-labeled Goat Anti-Rabbit IgG(H + L) antibody (A0208, Beyotime, 1:5000 for immunoblotting, 1:50 for IHC), HRP-labeled Goat Anti-Mouse IgG(H + L) antibody (A0216, Beyotime, 1:5000 for immunoblotting).

Reagents: Radio Immunoprecipitation Assay (RIPA) lysis buffer (P0013B, Beyotime), IP/Western lysing solution (P0013, Beyotime), protease inhibitor cocktail (B14001, Bimake), phosphatase inhibitor cocktail (B15001, Bimake), NuPAGE LDS Sample Buffer (4 ×) (NP0007, Thermo Fisher Scientific), JetPRIME transfection agent (Polyplus, Illkirch, France), Recombinant human TGF-α (HY-P7411,MCE), Recombinant mouse TGF-α (Huabio, China), Recombinant human pro-TGF-α (Huabio, China), Recombinant mouse pro-TGF-α (Huabio, China), Recombinant human MMP-28 (orb427161, Biorbyt), Recombinant mouse MMP-28 (Huabio, China), Erlotinib HCl (S1023, Selleck), Ravoxertinib (S7554, Selleck), STAT3-IN-1 (S0818, Selleck), STAT5-IN-1 (S6784, Selleck), PIK-90 (S1187, Selleck), Go 6983 (S2911, Selleck), Glecirasib (E1677, Selleck), HRS-4642 (E4651, Selleck), ZnCl2 solution (703,516, Merck).

Kits: diaminobenzidine (DAB) chromogen kit (Biocare, BDB2004), ECL Enhanced Kit (RM00021, ABclonal), Cell Counting Kit-8(C0038, Beyotime), FastPure Cell/Tissue Total RNA Isolation Kit V2 (RC112-01, Vazyme), Maxima First Strand cDNA Synthesis Kit (K1642, Thermo Scientific), ChamQ Universal SYBR RT-PCR Master Mix (Q711-03, Vazyme), human EGF Enzyme-Linked Immunosorbent Assay (ELISA) kit (ab217772, Abcam), Mouse EGF ELISA Kit (ab234560, Abcam), human TGF-α ELISA kit (ab100646, Abcam), Mouse TGF-α ELISA kit (EKE61812, Biomatik), human HB-EGF ELISA kit (ab100531, Abcam), Mouse HB-EGF ELISA kit (EKU04741, Biomatik), Duo-link kit (DUO92101, Sigma-Aldrich), Chromatin Immuno-Precipitation (ChIP) Assay Kit (P2080S,Beyotime), Dual Luciferase Reporter Assay Kit (FL101-01, Vazyme).

Plasmid: All plasmids were provided by OBiO Technology (Shanghai, China). The MMP-28 knockdown(KD) plasmid was based on the vector pSLenti-U6-shRNA-CMV-EGFP-F2A-Puro-WPRE, while the MMP-28 and various splice variant overexpression plasmids were based on the vector pSLenti-EF1-EGFP-P2A-Puro-CMV-MCS-3 × FLAG-WPRE. psPAX2 and pMD2G were used in lentivirus packaging. RNA interference sequence was shown in Table S3.

## Cell culture

KPC cell line, derived from spontaneous tumors of a KrasLSL-G12D; Trp53LSL-R172H; Pdx1-Cre mouse model, was a kind gift from the laboratory of Prof. Raghu Kalluri (MD Anderson Cancer Center, Houston, TX, USA). PANC-1, MiaPaCa-2, BxPC-3, T3M4 and SW1990 were purchased from ATCC (American Type Culture Collection). KPCs were maintained in McCoy’s 5A (Modified) Medium (Thermo Fisher Scientific). PANC-1, MiaPaCa-2, BxPC-3, T3M4 and SW1990 used in this study was routinely cultured in DMEM (SH30243.01, Life Sciences) supplemented with 10% fetal vovine serum (FBS) and 1% Pen/Strep (SH30022.01, Life Sciences), at 37 ℃ in a humidified 5% CO2 atmosphere.

## Generation of Erlotinib-resistant cells

SW1990 cells, 24 h post-passage, were cultured in complete medium supplemented with a final concentration of 1 nM Erlotinib. Subsequently, after each passage, a higher concentration of Erlotinib was added 24 h post-passage, increasing the dosage incrementally until a final concentration of 200 μM was reached. The cells continued to grow without significant inhibition, indicating the successful selection of an Erlotinib-resistant cell line.

## Proteomics

Sample was sonicated on ice using a high intensity ultrasonic processor (Scientz) in lysis buffer (8 M urea, 1% Protease Inhibitor Cocktail). The remaining debris was removed by centrifugation at 12,000 g at 4 °C for 10 min. Finally, the supernatant was collected and the protein concentration was determined with BCA kit according to the manufacturer’s instructions. For digestion, the protein solution was reduced with 5 mM dithiothreitol for 30 min at 56 °C and alkylated with 11 mM iodoacetamide for 15 min at room temperature in darkness. The protein sample was then diluted by adding 100 mM NH4HCO3 to urea concentration less than 2 M. Finally, trypsin was added at 1:50 trypsin-to-protein mass ratio for the first digestion overnight and 1:100 trypsin-to-protein mass ratio for a second 4 h-digestion. The tryptic peptides were dissolved in 0.1% formic acid (solvent A), directly loaded onto a home-made reversed-phase analytical column (25 cm*100 μm i.d.) packed with 1.9 μm Reprosil-Pur C18 beads (Dr. Maisch, Ammerbuch, Germany). The gradient was comprised of an increase from 3 to 6% solvent B (0.1% formic acid in 98% acetonitrile) over 3 min, 6% to 23% in 67 min, 23% to 38% in 12 min and climbing to 80% in 4 min then holding at 80% for the last 4 min, all at a constant flow rate of 450 nl/min on an UltiMate 3000 nanoLC system. The peptides were subjected to NSI source followed by tandem mass spectrometry (MS/MS) in Orbitrap Exploris 480 (Thermo Fisher Scientific,) coupled online to the UPLC. The electrospray voltage applied was 2.0 kV. The m/z scan range was 400 to 1200 for full scan, and intact peptides were detected in the Orbitrap at a resolution of 60,000. Peptides were then selected for MS/MS using NCE setting as 27 and the fragments were detected in the Orbitrap at a resolution of 15,000. A data-dependent procedure that alternated between one MS scan followed by 20 MS/MS scans with 30 s dynamic exclusion. Automatic gain control (AGC) was set at 5E4. Compensation voltage for FAIMS was set to − 45 V.The resulting MS/MS data were processed using Proteome Discoverer software v2.5 (Thermo Fisher). Tandem mass spectra were searched against human database using the SEQUEST algorithm. Trypsin(full) was specified as cleavage enzyme allowing up to 2 missing cleavages. The minimum peptide length was 6 amino acids with a maximum of 5 modifications per peptide. The mass tolerance for precursor ions was set as 10 ppm, and the mass tolerance for fragment ions was set as 0.02 Da. Carbamidomethyl on Cys was specified as fixed modification. The oxidation of Met (M), the acetyl, met-loss, and met-loss + acetyl of protein N-terminal were set as dynamic modifications. Proteins and PSMs were filtered with a maximum FDR of 1%.

## Bioinformatics analysis of online databases

In this study, the main databases used were the The Cancer Genome Atlas (TCGA) (https://www.cancer.gov/ccg/research/genome-sequencing/tcga) and the Genomics of Drug Sensitivity in Cancer (GDSC) (https://www.cancerrxgene.org/). The TCGA consortium experimentally measured gene expression profiles using the Illumina HiSeq 2000 RNA-sequencing platform. Data were downloaded from the TCGA Data Coordination Center by querying the Cancer Genomic Data Server (CGDS) using the R/CGDS package. The data from the TCGA database were used to analyze gene RNA levels in tissues and their relationship with prognosis. Additionally, the Gepia2 (http://gepia2.cancer-pku.cn/#index) and Kaplan–Meier Plotter (https://kmplot.com/analysis/) were also used to analyze TCGA data. The GDSC database records drug sensitivity test results and RNA sequencing results for various cell lines, using AUC to represent sensitivity. AUC stands for Area Under the Curve, indicating the area under the drug concentration–time curve. A larger AUC indicates that the cells can be exposed to higher drug concentrations and for longer durations, suggesting less sensitivity to the drug. AUC values and RNA levels were used for correlation analysis by *Pearson* correlation coefficient.

## Human tissue and specimen

Human pancreatic adenocarcinoma cancer tissues were obtained from the First Affiliated Hospital, School of Medicine, Zhejiang University. The protocol was approved by the Institutional Review Board at the First Affiliated Hospital, School of Medicine, Zhejiang University. Written informed consent was obtained from each patient at the time of enrollment. Tissue microarray slides containing 156 paraffin-embedded patient PDAC tissue samples were prepared with the assistance of Wuhan Servicebio Technology. Fourteen pairs of tumor tissue and para-tumor tissue paraffin specimens from PDAC patients were used for immunohistochemical staining, while frozen specimens from fourteen pairs of tumor tissue and para-tumor tissue from PDAC patients were used for immunoblotting detection. Twenty-four serum samples from PDAC patients and twenty-four serum samples from healthy donors were used for ELISA assay.

## Histology and immunohistochemistry (IHC)

Tissues were quickly dissected and fixed in 4% paraformaldehyde, embedded in paraffin and cut into slices. IHC was then performed with primary antibodies incubated overnight at 4℃ and then HRP-conjugated secondary antibody treated for 1 h in room temperature. Target proteins were visualized using a diaminobenzidine (DAB) chromogen kit, in which brown staining represented the targeted molecule. Slides were all counterstained with diluted hematoxylin for 3 min. Representative images of each tumor were captured using ImageScope software (Leica Biosystems). The IHC results were quantified by processing the images using Image J software.

## Immunoblotting and immunoprecipitation (IP)

For immunoblotting, cells were lysed using RIPA lysis buffer containing protease inhibitor cocktail and phosphatase inhibitor cocktail for 30 min on ice. For tissue samples, grinding is required using a homogenizer. The samples were heated to 100 °C in 1 × NuPAGE LDS Sample Buffer for 3–5 min. Equal amounts of protein were resolved by SDS-PAGE and transferred to polyvinylidene fluoride (PVDF) membranes (Bio-Rad). Then blocked with 5% skim milk for 1 h at room temperature, followed by Tris-buffered saline with Tween 20 (TBST) washed, and probed with primary antibodies at 1:1000 dilution overnight. The second antibody was incubated for 2–4 h at 4 °C. For co-immunoprecipitation (IP) experiments, cells were lysed in IP/Western lysing solution containing a phosphatase inhibitor cocktail and a protease inhibitor cocktail for 30 min on ice. Cleared lysates were analyzed by IP with the antibodies to pull down target protein. Then samples were heated to 100 °C in 1 × NuPAGE LDS Sample Buffer for 3–5 min and immunoblotted as described above. All corresponding bands were visualized using a ChemiScope Touch (Clinx Science Instruments). The intensity of immunoblot bands was evaluated using ImageJ 1.8.0 software (National Institutes of Health, Bethesda, USA).

## Transfection, lentivirus package and infection

For transfection, cells were seeded in 6-well plates at a density of 3 × 105 cells/well. PDAC cells or HEK-293 T cells were transfected for lentivirus package with a JetPRIME transfection agent according to the manufacturer’s instructions. For lentivirus package, after transfection, medium was replaced 6 h later and the supernatant was collected up to 48 h. After low-speed centrifugation to remove cellular components, virus was collected and concentrated by centrifuging at 25,000 rpm for 2–3 h and resuspended before adding to the cells. Targeted cells were selected based on Green fluorescent protein (GFP) expression fluorescence through flow cytometry or with puromycin to obtain stable transfected cell lines.

## Animal care

Six-week-old C57BL/6 and Balb/c nude mice were purchased from the Model Animal Research Center of Nanjing University and maintained in a specific-pathogen-free (SPF) environment in the Experimental Animal Center, the First Affiliated Hospital, School of Medicine, Zhejiang University. All animal experiments were approved by the Ethics Committee of the First Affiliated Hospital, School of Medicine, Zhejiang University and tumor burden was required to be controlled below 10% of body weight.

## Establishment of animal tumor model

The subcutaneous PDAC model was induced by a subcutaneous injection of 5 × 10^5^ PDAC cells into the right flank of C57BL/6 or nude mice which were randomly divided into different treatment groups (*n* = 5). Tumor growth was evaluated by measuring the volume with a caliper and assessed as length × width^2^ × 0.5. Tumors were harvested and weighed at the end of the experiment. The orthotopic PDAC model was established using C57BL/6 mice that were anaesthetized and treated with 5 × 10^5^ KPC cells injected into the tail of the pancreas, and mice were randomly assigned into different treatment groups (*n* = 6–10). The survival time of mice were recorded until the 30th day after cells injection. The liver metastasis model of PDAC was established using C57BL/6 mice that were anaesthetized and treated with 5 × 10^5^ KPC cells injected into spleen, and mice were randomly assigned into different treatment groups (*n* = 5). At the end of the experiment, mice were weighed before sacrifice. The mice’s livers were excised, weighed, and the number of metastatic foci (including inside the liver) was counted. In PDX model, fresh specimens from PDAC patients were implanted subcutaneously in 6-week-old male nude mice after pathological confirmation. When the tumor grows to about 2 cm in size, it was removed in a sterile procedure and cut into small pieces for implantation subcutaneously in new mice. A batch of stable passage PDX models were implanted in a new group of mice to form a research cohort, with excess tumor tissue subjected to immunoblotting analysis. Drug treatment was administered to the mice after approximately 7–14 days following tumor formation. Tumor volume of mice was recorded, and the tumors were removed and weighed at the end of the experiment.

## Colony formation assay

For the colony formation assay, single-cell suspensions were plated (500 cells per well) in 6-well plates, and medium was refreshed every 3 days for 15 days of culture. Inhibitors or DMSO were added after the cells adhered, and the inhibitors were administered throughout the entire process. Colonies were fixed in methanol, stained with crystal violet (0.5% crystal violet, 20% methanol). Positive clones (with more than 50 cells each) were photographed and counted.

## Cell Counting Kit-8 assay (CCK-8)

Cells were plated in 96-well plates at a density of 1 × 104 cells/well and were allowed to adhere overnight. Then cells were plated in four 96-well plates at a density of 2 × 10^3^ cells per well and were allowed to adhere. Each well was then treated with 10 μL of CCK-8 and incubated at 37 °C for 2 h. Cells were detected at absorbance (450 nm) using a microplate reader and recorded as 0 h. The same procedure was repeated every 24 h thereafter, with data recorded as 24, 48, and 72 h. Inhibitors or DMSO were added to all wells at the time of the first measurement.

## Transwell migration assays

Transwell assays were performed using Transwell 24-well plates (Costar, Corning). PDAC cells were starved in serum-free culture medium for 12–24 h. Each Transwell chamber was then seeded with 50,000 cells in serum-free medium. In the lower chamber of the 24-well plate, 500 µL of complete medium containing 10% FBS was added. Inhibitors or DMSO were added to both the upper and lower mediums. The system was incubated at 37 °C and 5% CO2 for 24 h. After removing excess medium and suspended cells from the upper chamber, the cells were fixed in 4% paraformaldehyde for 30 min and then stained with 0.5% crystal violet staining solution for another 30 min. Extra staining solution was washed off, and images were captured under a DMIL invert microscope (Leica). Five fields were counted and averaged for quantitative analysis.

## Wound healing assay

PDAC cells were plated into 6-well plates and grown to 100% density. Scratch wounds were made using a 1 mL pipette tip and washed with Phosphate Buffered Saline (PBS). Serum-free medium was added while inhibitors or DMSO were added simultaneously. The positions were marked and photographed under a DMIL invert microscope (Leica). After incubation at 37 °C and 5% CO2 for 24 or 30 h, photographs were taken again at the marked positions. ImageJ software was used to calculate the distances between five corresponding points in each image (left, center, right, left half, and right half). The average of these distances was then determined to obtain the scratch distance.The distance between the scratch wounds in the two sets of photographs was compared, and the wound healing rate was calculated.

## RNA-seq and gene expression analysis

RNA-seq was conducted at Shanghai Genechem Co. Ltd. (Shanghai, China). In brief, total RNA was extracted and its purity was assessed using a NanoPhotometer® spectrophotometer (IMPLEN, Westlake Village, CA, USA). Messenger RNA (mRNA) was isolated using Oligo Magnetic Beads and fragmented for cDNA synthesis. Libraries were prepared using the NEBNext Ultra™ RNA Library Prep Kit for the Illumina system (New England Biolabs, Ipswich, MA, USA) according to the manufacturer’s instructions, with index codes added to assign sequences to individual samples. After cluster generation, the libraries were sequenced on an Illumina HiSeq XTen platform. Raw data were subjected to quality control to produce high-quality clean data. Following read mapping, HTSeq was employed to count the number of reads mapped to each gene. The Fragments Per Kilobase of transcript per Million mapped reads (FPKM) value was then calculated based on gene length and read counts. Genes were ranked based on the normalized enrichment score (NES). Gene set enrichment analysis (GSEA) was performed using GSEA software (GSEA.4.1.0).

## RNA isolation and quantitative real-time PCR (RT-PCR)

Total RNA was isolated from cells using the FastPure Cell/Tissue Total RNA Isolation Kit, and cDNA was synthesized from 1,000 ng total RNA using Maxima First Strand cDNA Synthesis Kit for polymerase chain reaction (PCR) with reverse transcription. RT-PCR was performed in triplicate using ChamQ Universal SYBR RT-PCR Master Mix and protocols on a QuantStudio 5 Real-Time PCR system (Applied Biosystems). The target mRNA expression was quantified using the ΔΔCt method and normalized to GAPDH expression. All primers were designed using Primer 3 (http://frodo.wi.mit.edu/primer3/) and synthesized by Integrated DNA Technologies. Primer sequences are listed in Table S5.

## Duo-link

Cells were washed in PBS and incubated in 4% paraformaldehyde for 15 min and then permeabilized in 0.5% Triton X-100 solution for 15 min at room temperature. Samples were incubated with primary antibody overnight at 4 °C. Intramolecular interactions were detected and visualized by proximity ligation assay and rolling circle amplification using duo-link in situ kit according to the manufacturer’s instructions. Fluorescent images of the cells were observed under a TCS SP8 X confocal microscope (Leica).

## In vitro enzyme–substrate reaction

Recombinant MMP-28 were incubated with recombinant pro-TGF-α in PBS buffer with 1 mM ZnCl2 at 37 °C for 1 h, The kinase reaction was stopped by the addition of NuPAGE LDS Sample Buffer and boiling and subsequently subjected to immunoblotting.

## Chromatin Immuno-Precipitation

Chromatin Immuno-Precipitation (ChIP) was performed using a ChIP assay kit. Briefly, PDAC cells at 80% density were treated with 1% formaldehyde for 15 min at 37 °C, followed by neutralization with glycine for 5 min. After washing with PBS, the cells were collected in sodium dodecyl sulfate (SDS) lysis buffer and sonicated for 10 min. After centrifugation, the supernatants were collected and incubated with antibody or IgG overnight at 4 °C, followed by incubation with protein G magnetic beads for 6 h at 4 °C. The immunoprecipitated protein‒DNA complexes were eluted with 10 μg/ml proteinase K at 45 °C for 1 h. The immunoprecipitated DNA was further purified, collected using a DNA extraction kit, then amplified by PCR, and analyzed by nucleic acid electrophoresis. Primer sequences for ChIP are listed in Table S6.

## Dual-luciferase reporter gene assay

All plasmids were purchased from Sangon (Shanghai, China). HEK-293 T cells (5 × 105 cells/well) were seeded in 6-well plates for 1 day before transfection. The renilla-luciferase reporter plasmid with MMP-28 promoter were co-transfected with c-JUN and c-FOS overexpression plasmid. Corresponding vector for each plasmid were also transfected and served as a control. Drug or recombinant protein treatment was carried out at 24 h post-transfection, and luciferase activity were detected using the Dual Luciferase Reporter Assay Kit at 48 h post-transfection according to the manufacturer’s instructions.

## Multiplex immunohistochemistry (mIHC)

Opal™ 6-Plex Detection Kits (Akoya Biosciences, MA, USA) were utilized for mIHC according to the manufacturer’s protocol. Tissue slides were baked at 65 °C for 3 h to remove paraffin, then washed in xylene for 10 min, repeated three times. The slides were rehydrated through graded ethanol (100%−95%−70%−50%−30%) and subsequently washed three times with deionized distilled water. Epitope retrieval was performed using microwave heating with AR buffer, followed by cooling to room temperature for at least 15 min, and washing with TBST buffer. A PAP pen was used to outline the tissue on the slide. After incubating in blocking solution at room temperature for 15 min, slides were covered with primary antibody for 60 min, then with horseradish peroxidase-conjugated secondary antibody for 30 min, followed by staining with Opal fluorophore for 10 min. The slides were washed three times with TBST for 2 min each time. The process of epitope retrieval, blocking, primary antibody incubation, secondary antibody incubation, and Opal fluorophore staining was repeated for each primary antibody. Finally, tissue slides were counterstained with spectral 4',6-diamidino-2-phenylindole (DAPI) for 5 min and coverslipped with Fluoromount medium (SouthernBiotech, Birmingham, USA). Multi-color image acquisition was performed using the Vectra Polaris System (Akoya Biosciences), and spectral unmixing was conducted using inForm® software (Akoya Biosciences). The primary antibodies and their respective fluorescent dyes were as follows: MMP-28 antibody/Opal 570, Phospho-EGF Receptor (Tyr1173) antibody/Opal 520, and Phospho-EGF Receptor (Tyr1068) antibody/Opal 690.

## Statistical analyses

Statistical analysis was performed using SPSS (V20, IBM Corp., Armonk, NY, USA) and Prism software (GraphPad Inc, version 8.0). Data for all experiments from at least three biological replicates were presented as means ± SD. Student’s t test was used to analyze continuous variables for two groups. One-way and repeated-measures analysis of variance (ANOVA) was used to compare multiple groups. Survival analysis was conducted using the Kaplan–Meier method and compared using the log-rank test. Details were indicated in figure legends. The data are shown as mean ± standard error of mean (SEM). ns, not significant, *p < 0.05; **p < 0.01; ***p < 0.001.

## Results

### MMP-28 is associated with sensitivity to EGFR inhibitors and serves as a risk factor for PDAC

**Fig. 1:**
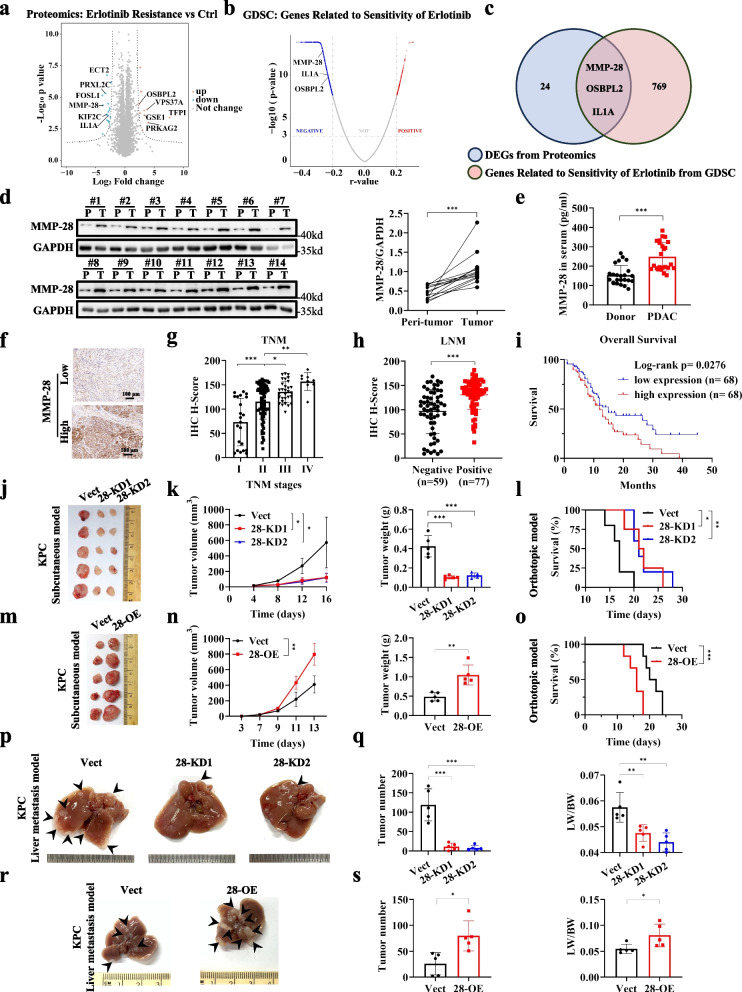
MMP-28 is associated with sensitivity to Erlotinib and serves as a risk factor for PDAC. **a** Starting from a low concentration and gradually increasing it, Erlotinib-resistant SW1990 cells were screened. Proteomics analysis was performed and the results were shown in a volcano plot. |Log_2_ Fold change × Log_10_ *p* value|> 1 was considered a significant change. **b** Genes significantly correlated with the AUC of Erlotinib across pan-cancer cell lines were displayed. *p* < 0.001 was considered significant. r < −0.2 indicated a negative correlation with the AUC, and r > 0.2 indicated a positive correlation with the AUC. Data was from GDSC database. **c** Venn diagram was used to present the intersection of the two datasets. MMP-28, IL1A, and OSBPL2 showed significant changes in proteomics and were significantly associated with sensitivity of Erlotinib. **d** Immunoblotting analysis of MMP-28 was performed in paired pancreatic tissue samples from PDAC patients (*n* = 14) (P: Peri-tumor tissue; T: Tumor tissue). **e** ELISA detection of MMP-28 was performed in the peripheral blood serum of PDAC patients (*n* = 24) and health donors (n = 24). **f**-**i** Tissue microarrays composed of 156 cases of PDAC tumors were used for IHC staining analysis of MMP-28 (136 cases had complete pathological and prognostic information). Representative images of IHC were shown in **f**. Correlation between expression levels of MMP-28 and TNM stage were analyzed in **g** (n = 136). Correlation between expression levels of MMP-28 and lymphatic nodes metastasis were analyzed in **h** (*n* = 136). Samples were divided into two groups according to the expression of MMP-28. Correlation between expression levels of MMP-28 and OS were analyzed in **i** (*n* = 136). **j**, **k** KPC cells with MMP-28 KD or not were injected subcutaneously into C57BL/6 J mice. Representative images of tumors were shown in **j**. Tumor growth curve and tumor weight were shown in **k**. **l** KPC cells with MMP-28 KD or not were injected orthotopically into the pancreas of C57BL/6 J mice. Survival time was recorded. **m**, **n** KPC cells with MMP-28 OE or not were injected subcutaneously into C57BL/6 J mice. Representative images of tumors were shown in **m**. Tumor growth curve and tumor weight were shown in **n**. **o** KPC cells with MMP-28 OE or not were injected orthotopically into the pancreas of C57BL/6 J mice. Survival time was recorded. **p**, **q** KPC cells with MMP-28 KD or not were injected into the spleen of C57BL/6 mice for the liver metastasis model. Representative images of liver were shown in **p**. Tumor number and the ratio of liver weight to body weight were shown in **q**. **r**, **s** KPC cells with MMP-28 OE or not were injected into the spleen of C57BL/6 mice for the liver metastasis model. Representative images of liver were shown in **r**. Tumor number and the ratio of liver weight to body weight were shown in **s**.^*^p < 0.05; ^**^p < 0.01; ^***^p < 0.001; ns, not significant

To improve the effectiveness of proliferation signaling inhibition strategies in PDAC, we aim to explore genes associated with Erlotinib resistance. For this purpose, we first constructed Erlotinib-resistant SW1990 cell lines and conducted proteomic analysis comparing with control SW1990 cells, and the set of 27 proteins significantly differentially expressed were identified (Fig. 1a, Table S1). Meanwhile, we analyzed the GDSC database and identified 772 genes significantly associated with Erlotinib sensitivity (Fig. 1b, Table S2). By taking the intersection of the two sets, we identified three genes that are highly associated with erlotinib sensitivity (Fig. 1c). Among these, MMP-28 shows more significant differential expression in the proteomics analysis and also exhibits stronger correlation with erlotinib sensitivity in the GDSC database analysis. Further investigations have shown that MMP-28 is associated with sensitivity of several other EGFR inhibitors, including Gefitinib, Afatinib, Sapitinib, Lapatinib, AZD3759, and Osimertinib (Fig. S1a-g). Therefore, we identified MMP-28 as research target. Subsequently, we analyzed the TCGA database and found that MMP-28 is significantly overexpressed in PDAC (Fig. S2a). Meanwhile, we assessed MMP-28 protein expression level in tumor tissues and corresponding adjacent peri-tumor tissue through immunohistochemistry (n = 14) and immunoblotting (*n* = 14). Results demonstrated a significantly upregulation of MMP-28 expression in PDAC tissues (Fig. 1d and S2b). Besides, we identified that PDAC patients displayed higher MMP-28 expression level in peripheral blood tissue than healthy donors (Fig. 1e). In addition, analysis of the TCGA database revealed that patients with high MMP-28 expression have significantly shortened OS and recurrence-free survival (RFS) (Fig. S2c-d). We then performed immunohistochemical (IHC) in a PDAC microarray composed of 156 samples (136 of whom had completed pathological and prognostic information) and calculated H-score (Fig. 1f and S2e). Results showed that MMP-28 expression was higher in advanced stage of PDAC (TNM stage at III and IV) (Fig. 1g). Data from the TCGA database also suggested that advanced stage PDAC patient presented the higher MMP-28 mRNA expression level (Fig. S2f). Additionally, analyzing the protein levels of MMP-28 in the PDAC microarray showed that high MMP-28 expression was positively correlated with lymph node metastasis (LNM) (Fig. 1h). Furthermore, survival analysis indicated that high MMP-28 expression was significantly associated with poor prognosis in PDAC (Fig. 1i and Table 1). These findings suggested that high MMP-28 expression indicated high malignancy in PDAC. In addition, a pan-cancer correlation analysis using the TCGA database revealed that MMP-28 expression was a significant risk factor specifically for PDAC (Fig. S2g-h). Collectedly, our results indicated that MMP-28 was a promising target. Exploring its role and mechanisms in PDAC may deepen our understanding of proliferation signaling and potentially enhance the efficacy of EGFR inhibitors in PDAC.

**Table 1:** Clinicopathological relevance of MMP-28 in PDAC patients

**Variable**	**MMP-28**		***p* value**
	**Low (H-score ≤ 125.5)**	**High (H-score > 125.5)**	
Gender			0.164
Male,* n *(%)	40 (58.8)	39 (57.4)	
Female,* n *(%)	28 (41.2)	16 (42.6)	
Age, years			0.999
>65,* n *(%)	34 (50)	34 (50)	
≤65,* n *(%)	34 (50)	34 (50)	
BMI, kg/m2			0.301
<18.5,* n *(%)	16 (23.5)	9 (13.2)	
18.5–23.9,* n *(%)	36 (53.0)	41 (60.3)	
>23.9,* n *(%)	16 (23.5)	18 (26.5)	
TNM stage			**0.010**
I,* n *(%)	16 (23.5)	6 (8.8)	
II,* n *(%)	39 (57.4)	36 (52.9)	
III,* n *(%)	12 (17.6)	18 (26.5)	
IV,* n *(%)	1 (1.5)	8 (11.8)	
Vascular invasion			**0.001**
Yes,* n *(%)	24 (35.3)	46 (67.6)	
No,* n *(%)	44 (64.7)	22 (32.4)	
Nerve invasion			0.196
Yes,* n *(%)	43 (63.2)	50 (73.5)	
No,* n *(%)	25 (36.8)	18 (26.5)	
Serum CA12-5, U/mL			0.317
≥35,* n *(%)	19 (27.9)	14 (20.6)	
<35,* n *(%)	49 (72.1)	54 (79.4)	
Serum CA19-9, U/mL			0.205
≥37,* n *(%)	44 (64.7)	54 (79.4)	
<37,* n *(%)	24 (35.3)	14 (20.6)	
Serum CEA, U/mL			0.853
≥5,* n *(%)	22 (32.4)	21 (30.9)	
<5,* n *(%)	46 (67.6)	47 (69.1)	
Tumor differentiation			0.071
Well,* n *(%)	6 (8.8)	2 (2.9)	
Moderate,* n *(%)	45 (66.2)	38 (55.9)	
Poor,* n *(%)	17 (25)	28 (41.2)	
Recurrence			**0.042**
Yes,* n *(%)	13 (61.8)	10 (48.5)	
No,* n *(%)	55 (38.2)	58 (51.5)

To further elucidate the impact of MMP-28 on PDAC progression, we first assessed MMP-28 expression level in PDAC cell lines, including PANC-1, MiaPaCa-2, BXPC-3, T3M4, SW1990, and KPC. MMP-28 expression level was highest in SW1990 cells and lowest in T3M4 cells (Fig. S3a-b). Consequently, we constructed stable MMP-28 KD cell lines in KPC and SW1990 using shRNA lentivirus (Fig. S3c-f), and constructed stable MMP-28 overexpression (OE) in KPC and T3M4 using lentivirus (Fig. S3g-j). In a subcutaneous tumor model derived from KPC with MMP-28 KD or not, results demonstrated that MMP-28 KD significantly inhibited tumor growth (Fig. 1j and 1k). In addition, MMP-28 KD significantly extended the survival of mice in an orthotopic tumor model (Fig. 1l). In turn, MMP-28 OE promoted tumor growth and shortened the survival of mice in vivo (Fig. 1m and 1o). In vitro, CCK-8 assays and colony formation assays similarly showed that MMP-28 KD significantly suppressed the proliferation of PDAC cells (Fig. S4a-b). Instead, MMP-28 OE markedly promoted the proliferation of PDAC cells (Fig. S4c-d). Meanwhile, we established liver metastasis mice models and investigated the effect of MMP-28 on PDAC metastasis using MMP-28 KD and OE cell lines. Results showed that MMP-28 KD significantly inhibited PDAC metastasis (Fig. 1p and 1q), while MMP-28 OE significantly promoted it (Fig. 1r and 1s). Similar results were confirmed in vitro. Wound healing assays and Transwell migration assays demonstrated that MMP-28 significantly enhanced the migration of PDAC cells (Fig. S4e-h).

Overall, we found that MMP-28 was highly expressed in PDAC and significantly promoted both growth and metastasis of PDAC. Clinically, MMP-28 expression was strongly associated with poor prognosis of PDAC patients.

## MMP-28 activates EGFR-mediated growth signaling pathway for PDAC progression in a TGF-α-dependent autocrine manner

**Fig. 2:**
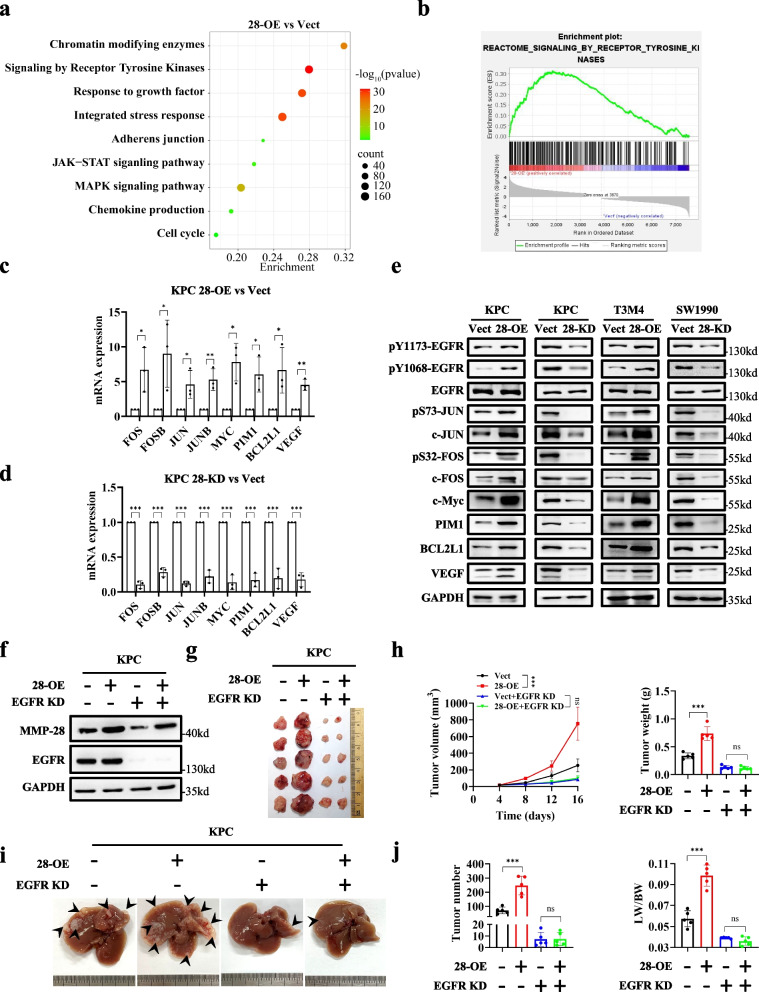
EGFR signaling pathway is identified as a representative effector cascade downstream of MMP-28 for PDAC progression. **a** Displayed in a bubble plot, the impact of MMP-28 OE on multiple signaling pathways in T3M4 cells was depicted through transcriptomic analysis. **b** GO enrichment analysis was performed for differentially expressed genes related to “response to growth factor” signaling pathway between Vect and 28-OE T3M4 cells from the transcriptomic analysis. **c**, **d** RT-PCR was employed to discern the transcriptional abundance of EGFR downstream genes. Regulatory role of MMP-28 OE on KPC cells were shown in **c**. Regulatory role of MMP-28 KD on KPC cells were shown in **d**. **e** Immunoblotting was employed to discern the protein levels of EGFR downstream genes in multiple PDAC cell lines. **f** Transfection of EGFR KD shRNA with vector was performed on Vect and 28-OE KPC cells. Immunoblotting was utilized to detect the expression of MMP-28 and EGFR. **g**, **h** Multitype KPC stable transfectants were injected subcutaneously into C57BL/6 J mice as indicated. Representative images were shown in **g**. Tumor growth curve and weight were shown in **h**. **i**, **j** Multitype KPC stable transfectants were injected into the spleen of C57BL/6 J mice as indicated. Representative images were shown in **i**. Tumor number and ratio of liver weight to body weight were shown in **j**. ^*^p < 0.05; ^**^p < 0.01; ^***^p < 0.001; ns, not significant

To explore the mechanism by which MMP-28 promoted PDAC progression, we performed transcriptomic analysis on T3M4 cell lines with MMP-28 OE or not, aiming to amplify the effects of MMP-28 as much as possible (Table S4). Gene set enrichment analysis (GSEA) revealed significant upregulation of pathways related to RTKs and growth factors in MMP-28 OE cell lines (Fig. 2a-b and S5a). We also observed notable changes in JAK-STAT and mitogen-activated protein kinase (MAPK) signaling pathways, which are classic downstream pathways of growth factors and RTKs [23, 24] (Fig. S5a). To validate the transcriptomic results, we next performed RT-PCR to assess the expression of several downstream molecules of growth factor and RTKs, including FOS, JUN, MYC, PIM1, BCL2L1, and VEGF, confirming that MMP-28 indeed activated proliferation signaling (Fig. 2c-d and Fig. S5b-c). There are currently more than 50 known RTKs, all of which share similar structures and functions [25]. Subsequently, we chose EGFR, one of the most classic RTKs, as our focus for further research [26, 27]. Immunoblotting analysis showed that MMP-28 significantly promoted the phosphorylation and activation of EGFR, with upregulation of downstream molecules including STAT3, STAT5, ERK1/2, c-JUN, and c-FOS (Fig. 2e and Fig. S5d). Immunoblotting across various PDAC cell lines further supported the correlation between MMP-28 expression and EGFR activity (Fig. S5e). To determine whether MMP-28’s promoting PDAC progression is an EGFR-dependent manner, we knocked down EGFR expression in PDAC cell lines with MMP-28 OE (Fig. 2f and Fig. S6a). And then stable transfectant of KPC were subcutaneously implanted into mice. Results indicated the indispensable role of EGFR in MMP-28’s promotion of PDAC growth in vivo (Fig. 2g and 2h). In vitro, CCK-8 and colony formation assays also demonstrated that MMP-28’s promotion of PDAC cell proliferation is EGFR-dependent (Fig. S6b-c). Meanwhile, the liver metastasis model in mice demonstrated that MMP-28 promotes PDAC metastasis in an EGFR-dependent manner (Fig. 2i and 2j). In vitro, wound healing and Transwell migration assays collectively demonstrated that MMP-28 promoted migration of PDAC cell relying on EGFR (Fig. S6d-e).

**Fig. 3:**
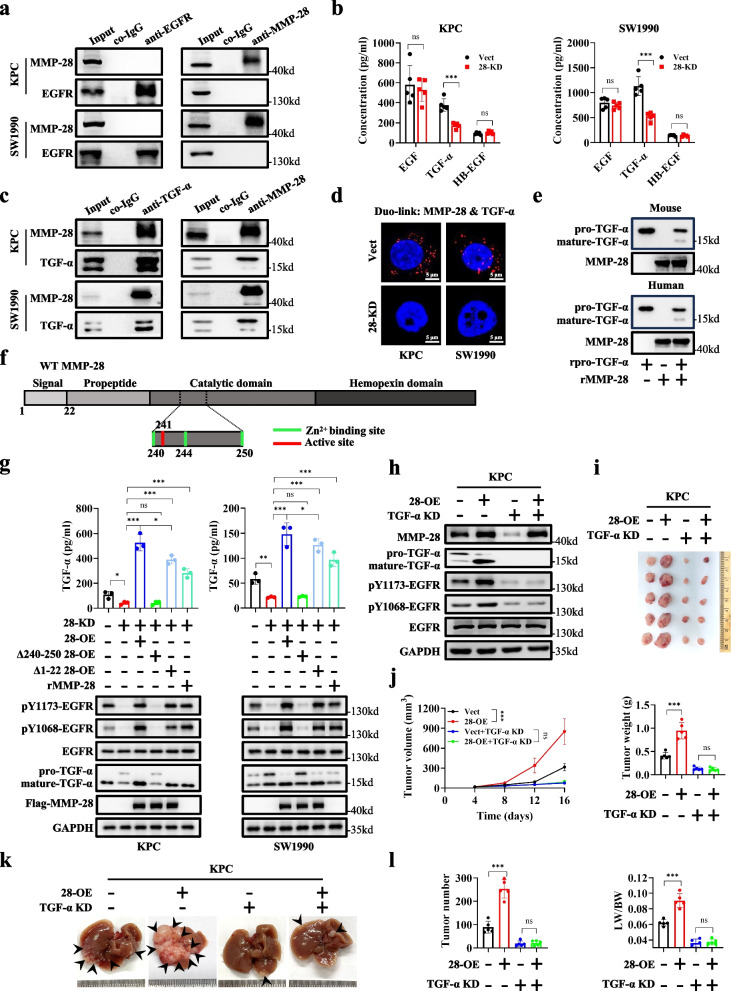
MMP-28 activates EGFR-mediated growth signaling pathway for PDAC progression in a TGF-α-dependent autocrine manner.**a** Cell lysates from KPC, and SW1990 separately analyzed by IP and immunoblotting analysis using the antibodies indicated. **b** ELISA was performed to detect soluble EGF, TGF-α, and HB-EGF in supernatant of PDAC cells with MMP-28 KD. **c** Cell lysates from KPC, and SW1990 separately analyzed by IP and immunoblotting analysis using the antibodies indicated. **d** Duo-link assay was performed to detect interaction between MMP-28 and TGF-α. Representative images were shown and the red dots indicated their interaction. **e** Recombinant pro-TGF-α and recombinant MMP-28 were co-incubated under appropriate conditions, and the maturity of TGF-α with MMP-28 was detected using immunoblotting. **f** The domains of MMP-28 were depicted according to uniport. The secretion signal peptide, active sites and some of the Zn^2+^ binding sites were shown. **g** Rescue of MMP-28 (wildtype or splice variant) or addition of exogenous recombinant MMP-28 were performed in MMP-28 KD PDAC cells. Soluble TGF-α were detected by ELISA assay and immunoblotting was performed to assess TGF-α and EGFR. **h** Transfection of TGF-α KD shRNA with vector was performed on Vect and 28-OE KPC cells. Immunoblotting was utilized to detect the expression of MMP-28 and TGF-α. **i, j** Multitype KPC stable transfectants were injected subcutaneously into C57BL/6 J mice as indicated. Representative images were shown in **i.** Tumor growth curve and weight were shown in **j**. **k**, **l** Multitype KPC stable transfectants were injected into the spleen of C57BL/6 J mice as indicated. Representative images were shown in **k**. Tumor number and ratio of liver weight to body weight were shown in **l**.^*^p < 0.05; ^***^p < 0.001; ns, not significant

Having determined the pivotal role of EGFR in the tumor promotion of MMP-28, we aimed to elucidate the mechanism by which MMP-28 regulated EGFR. However, co-immunoprecipitation (CO-IP) experiments did not reveal any direct protein–protein interaction between MMP-28 and EGFR (Fig. 3a). Thus, we hypothesized that MMP-28 might influence EGFR activation by its ligands. Classic EGFR ligands, such as EGF, TGF-α, and HB-EGF, all contain one or more EGF-like domains, which are crucial for binding to EGFR [28, 29]. These ligands are often stored inside cells or on the cell membrane as an inactive precursor. After being cleaved and matured, they are released and bind to EGFR [30]. We used ELISA to evaluate the effect of MMP-28 on three classic EGFR ligands: EGF, TGF-α, and HB-EGF individually. Results demonstrated that MMP-28 significantly increased the soluble form of TGF-α (Fig. 3b and Fig. S7a). Immunoblotting further confirmed that MMP-28 promoted the maturation of TGF-α from its precursor form, with no significant effect on EGF or HB-EGF (Fig. S7b). Moreover, the exogenous addition of recombinant MMP-28 protein also enhanced the release of TGF-α (Fig. S7c). Notably, through RT-PCR, we found that overexpression of MMP-28 slightly increased the RNA levels of EGFR and TGF-α, but knockdown of MMP-28 had almost no effect. Since overexpression is a relatively artificial approach, we believe that the knockdown results are more indicative. Therefore, we speculate that the regulation of EGFR and TGF-α by MMP-28 is not in a transcriptional manner but more likely at the protein level. Further CO-IP and Duo-link assays identified a direct protein–protein interaction between MMP-28 and TGF-α (Fig. 3c and 3d). In vitro experiments using recombinant MMP-28 protein and pro-TGF-α protein directly indicated that MMP-28 facilitated the maturation of TGF-α (Fig. 3e). To further investigate the mechanism by which MMP-28 promoted TGF-α maturation, we constructed overexpression plasmids for two splice variants of MMP-28. One was an inactive splice variant, Δ240–250, with the key metalloprotease active site and zinc ion-binding site removed. The other was a non-secreted splice variant, Δ1–22, with the signal peptide removed (Fig. 3f). In MMP-28 KD cell lines, immunoblotting analysis revealed reduced maturation of TGF-α and decreased EGFR activation. Rescue with wild-type MMP-28 significantly restored TGF-α maturation and EGFR activation. In contrast, rescue with the inactive Δ240–250 MMP-28 had no effect, indicating that the processing of TGF-α by MMP-28 was dependent on its metalloprotease activity. Additionally, rescue with non-secretory Δ1–22 MMP-28 or addition with exogenous recombinant MMP-28 protein were capable to cleave the TGF-α precursor, indicating that MMP-28 can cleave TGF-α both intracellularly and on the cell membrane (Fig. 3g).

To confirm whether MMP-28’s activation of EGFR and promotion of PDAC progression are dependent on TGF-α or not, we constructed PDAC cell lines with MMP-28 OE and TGF-α KD. Immunoblotting results indicated that MMP-28 could not activate EGFR without TGF-α (Fig. 3h and Fig. S7f). In vivo experiments showed that MMP-28’s promotion of PDAC growth was dependent on TGF-α (Fig. 3i and 3j). In vitro, similar results were observed in CCK-8 and colony formation assays (Fig. S8a-b). In vivo liver metastasis models (Fig. 3k and 3l), along with in vitro wound healing and Transwell migration assays (Fig. S8c-d), we confirmed that TGF-α was indispensable for MMP-28’s promotion of PDAC metastasis.

In summary, we found that MMP-28 cleaved the precursor of TGF-α and promoted its maturation and release, thereby activating EGFR and contributing to the progression of PDAC.

## EGFR promotes MMP-28 transcription for sustaining proliferative signaling in PDAC through an ERK-AP1 axis-dependent positive feedback

**Fig. 4:**
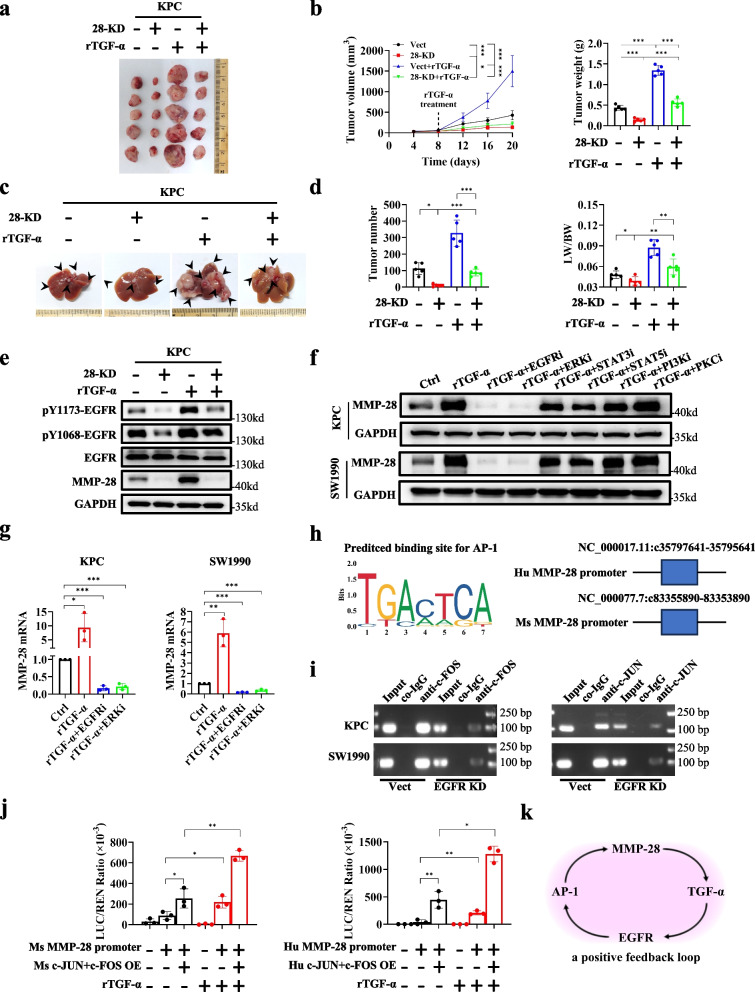
EGFR promotes MMP-28 transcription for sustaining growth signal in PDAC through an ERK-AP1 axis-dependent positive feedback. **a**, **b** Multitype KPC stable transfectants were injected subcutaneously into C57BL/6 J mice as indicated. Representative images were shown in **a**. Tumor growth curve and weight were shown in **b**. **c**, **d** Multitype KPC stable transfectants were injected into the spleen of C57BL/6 J mice as indicated. Representative images were shown in **c**. Tumor number and ratio of liver weight and body weight were shown in **d**. **e** Vect and 28-KD KPC cells were treated with exogenous recombinant TGF-α. Immunoblotting was utilized to detect the expression of MMP-28 and phosphorylated and total EGFR. **f** Immunoblotting was performed to detect MMP-28 in PDAC cells with exogenous recombinant TGF-α or multitype EGFR signaling pathway inhibitors as indicated. **g** RT-PCR was performed to detect transcriptional level of MMP-28 in PDAC cells with exogenous recombinant TGF-α or multitype EGFR signaling pathway inhibitors as indicated. **h** Predicted binding site for AP-1 was obtained from JASPAR database. **i** CHIP and nucleic acid electrophoresis detected the binding of c-JUN and c-FOS to the MMP-28 promoter in PDAC cells with EGFR KD or not. **j** Dual-luciferase reporter gene assay detected the activation of AP-1 on the MMP-28 promoter in 293 T cells with treatment as indicated. **k** A schematic diagram illustrated the positive feedback loop discovered in this study involving MMP-28, TGF-α, EGFR, and AP-1.^*^p < 0.05; ^**^p < 0.01; ^***^p < 0.001

While investigating whether the function of MMP-28 depends on TGF-α, we injected exogenous recombinant TGF-α protein into subcutaneous tumor model derived from KPC cells with or without MMP-28 KD. Unexpectedly, results showed that exogenous recombinant TGF-α protein was insufficient to promote growth in the MMP-28 KD group (Fig. 4a and 4b). Similarly, CCK-8 and colony formation assays confirmed this observation in vitro (Fig. S9a-b). Meanwhile, the liver metastasis model in mice (Fig. 4c and 4d), as well as wound healing and Transwell migration assays in vitro (Fig. S9c-d), demonstrated that MMP-28 was also required for TGF-α to fully exert its metastatic promoting effects. In addition, immunoblotting showed that the activation of TGF-α on EGFR was partially diminished when MMP-28 knocked down as well (Fig. 4e and Fig. S9e). Furthermore, immunoblotting showed that exogenous recombinant TGF-α protein significantly upregulated MMP-28 (Fig. 4e and Fig. S10a), and EGFR KD downregulated MMP-28 instead (Fig. S10b). These results suggested a hypothesis that there is reciprocal regulation between MMP-28 and EGFR, and both are required for sustaining proliferation signaling.

To further investigate how EGFR regulates MMP-28, we treated PDAC cells with recombinant TGF-α protein to activate EGFR, followed with inhibitors of EGFR or downstream classical signaling molecules (ERK, STATs, PI3K, PKC) [31]. Immunoblotting showed that the upregulation of TGF-α on MMP-28 was significantly inhibited by inhibitors of EGFR and ERK (Fig. 4f). Additionally, RT-PCR experiments revealed that inhibitors of EGFR and ERK could downregulate MMP-28 at transcriptional level (Fig. 4g). These results suggested that EGFR upregulates MMP-28 through ERK signaling pathway.

The ERK pathway, also known as MAPK pathway, mainly regulates tumor growth and metastasis through downstream transcription factors [32]. Notably, the classical downstream transcription factor AP-1 (a complex of JUN and FOS) has been reported to regulate MMPs [33, 34], and was found to be regulated by MMP-28 in the previous results. Therefore, we hypothesize that EGFR might upregulate MMP-28 through ERK and AP-1. To validate this, we predicted the binding sites on MMP-28 promoter of AP-1 via JASPAR database and designed corresponding primers (Fig. 4h). Chromatin Immunoprecipitation (ChIP) assays confirmed that AP-1 could bind to the promoter of MMP-28, which was inhibited by EGFR KD (Fig. 4i and Fig. S10c). Dual-luciferase reporter assays also demonstrated that EGFR indeed upregulated MMP-28 expression through AP-1-mediated transcription (Fig. 4j).

Thus, we have identified a positive feedback loop consisting of MMP-28-TGF-α-EGFR-AP-1-MMP-28 (Fig. 4k), which provided sustaining proliferation signaling to PDAC cells and promoted their proliferation and metastasis.

## Reciprocal regulation of MMP-28 and EGFR determines therapeutic efficacy of EGFR inhibitor in PDAC

**Fig. 5:**
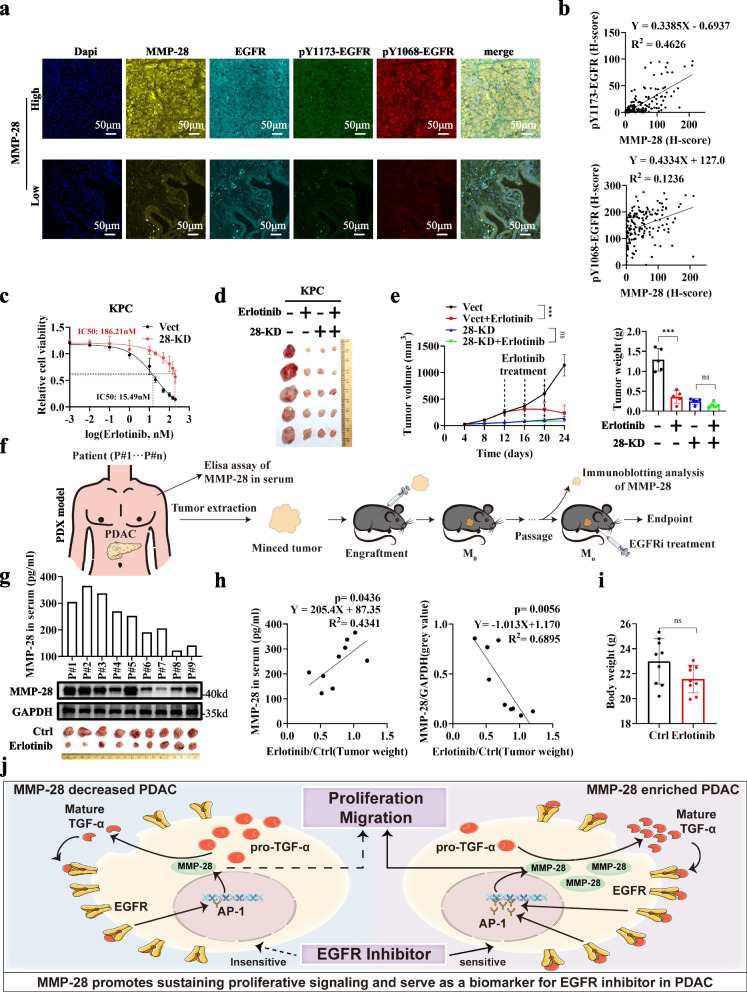
Reciprocal regulation of MMP-28 and EGFR determines therapeutic efficacy of EGFR inhibitor in PDAC. **a**, **b** Tissue microarrays composed of 156 cases of PDAC tumors were used for multicolor immunohistochemical staining analysis. Representative images of individual channels and merged images were shown in **a**. Correlation analysis between MMP-28 and pY1173-EGFR, pY1068-EGFR was presented in **b**. **c** CCK-8 assay was performed to detect the IC50 of Erlotinib in KPC cells with MMP-28 KD or not. **d**, **e** KPC cells with MMP-28 KD or not were injected subcutaneously into C57BL/6 J mice with Erlotinib treatment. Representative images were shown in **d**. Tumor growth curve with Erlotinib treatment indicated by dashed lines, and tumor weight were shown in **e**. **f**-**l** The PDX model was used to validate the correlation between MMP-28 expression levels and sensitivity to EGFR inhibitor Erlotinib. Schematic model for generating PDX of PDAC were shown in **f**. ELISA was used to detect the expression of MMP-28 in patients’ serum and immunoblotting was used to detect the expression of MMP-28 in tumor tissue of PDX model. Results were shown in **g**. At the experimental endpoint, representative images of tumors were shown in **g**. Correlation analysis between expression of MMP-28 and sensitivity to Erlotinib were shown in **h**. Body weight of mice were recorded in **i**. **j** Working model of this study. MMP-28 promotes TGF-α maturation and release by cleaving its precursor, thereby activating EGFR. Activated EGFR transcriptionally upregulate MMP-28 by AP-1, forming a positive feedback regulation. This positive feedback loop provides sustaining proliferative signaling to tumor cells, promoting their proliferation and migration. Furthermore, patients with high expression of MMP-28 are more sensitive to EGFR inhibitor treatment. ^***^p < 0.001; ns, not significant

Based on the positive regulation between MMP-28 and EGFR, we were dedicated to developing therapeutic strategies targeting this sustaining proliferation signaling. We performed mIHC on PDAC samples and found strongly positive correlation between MMP-28 expression and EGFR activity (Fig. 5a-b and S11a-b). Due to the current lack of specific inhibitors for MMP-28, we are unable to directly target MMP-28 or develop combination therapy strategies. Thus, we investigated whether the expression level of MMP-28 could serve as a biomarker for predicting the efficacy of EGFR inhibitors. In vitro studies showed that SW1990, which expressed high levels of MMP-28, were more sensitive to Erlotinib, whereas T3M4, which expressed low levels of MMP-28, were less responsive to Erlotinib (Fig. S11c). Additionally, knocking down MMP-28 in KPC also resulted in reduced sensitivity to Erlotinib in vitro (Fig. 5c). In vivo, tumor derived from T3M4 exhibited insensitivity to Erlotinib compared to SW1990 (Fig. S11d-e). MMP-28 KD in KPC reduced sensitivity to Erlotinib and MMP-28 OE increased it instead (Fig. 5d-e and S11f-g). In a patient-derived xenograft (PDX) model of PDAC, we analyzed the expression levels of MMP-28 in patients’ serum and PDX tumor tissues, and assessed the tumor’s sensitivity to Erlotinib (Fig. 5f and 5g). Correlation analysis revealed that PDAC with high MMP-28 expression level responded well to Erlotinib therapy at dose without significant side effects (Fig. 5h and 5i). These results suggested that detecting MMP-28 in patient serum could predict their response to Erlotinib, avoiding invasive procedures and benefiting patients who cannot undergo surgery.

Previous reports have suggested that Erlotinib is more effective in the treatment of PDAC with wild-type KRAS. With the rapid development of KRAS mutant inhibitors, particularly those targeting the G12 mutation, in recent years, the potential of dual-target therapy against KRAS and EGFR has also become a highly interesting area of research. In our study, we also attempted to combine Erlotinib with the KRAS G12D-targeted drug HRS-4642, demonstrating a strong synergistic effect (Fig. S11h-i). Additionally, in vitro treatment of MiaPaCa-2 with the Glecirasib (KRAS G12C inhibitor) and SW1990 with HRS-4642 revealed a dose-dependent significant downregulation of MMP-28 (Fig. S11j-k). This seems to indicate a close connection between KRAS mutant signaling and MMP-28, which may help explain the origin of the sustained growth signal in PDAC and pave the way for developing new therapeutic strategies. This requires further in-depth research.

In summary, the reciprocal regulation between MMP-28 and EGFR offers a promising clinical strategy: employing MMP-28 as a biomarker to identify patients who are more likely to respond to EGFR inhibitors (Fig. 5j). This strategy enhances Erlotinib’s efficacy, reduces its dose and toxicity, and making its clinical application more widespread.

## Discussion

Sustaining proliferative signaling is one of the ten hallmarks of cancer [10]. Normal tissues meticulously regulate the production and release of growth-promoting signals that guide cells through an orderly cycle of growth and division, thereby maintaining cellular homeostasis and the normal structure and function of tissues. In contrast, tumor cells produce self-sufficiency growth signals, enabling unlimited proliferation. Extensive research has been conducted on growth signals in the past. RTKs are activated by binding their extracellular domains to corresponding ligands, such as EGF and TGF-α, which determines RTKs’ oligomerization [35–37]. This process activates the intracellular domains, promotes the recruitment of proteins and signal cascades, and integrates various signaling pathways, such as MAPK and PI3K/AKT, to regulate the biological behaviors of tumor cells [38]. While the binding of ligands to receptors and the transmission of signals are relatively well understood, there is still limited information on how growth signals are sustaining continuously and self-sufficiently. This study identified a positive feedback loop in PDAC consisting of MMP-28-TGF-α-EGFR-AP-1-MMP-28. This loop explains how self-sufficiency growth signals are maintained. Moreover, since both MMP-28 and TGF-α are secreted proteins, this signaling can be transmitted to neighboring tumor cells via paracrine mechanisms and even affect distal lesions via endocrine actions.

Growth signaling plays a crucial role in tumor development and progression, leading to the development of various therapeutic strategies aimed at blocking these signals. Among these, RTK inhibitors are the most well-established [39–41]. For example, Erlotinib has been demonstrated to modestly extend the survival time of patients with PDAC [42–44]. Consequently, researchers are seeking ways to enhance their efficacy. Research has found that patients who develop a skin rash following Erlotinib treatment experience greater survival benefits [45]. Another study indicates that PDAC patients with Kras wild-type tumors respond better to Erlotinib [46]. These findings highlight the potential of identifying biomarkers for RTK inhibitors to improve their effectiveness. However, the current biomarker, rash, does not provide pre-treatment guidance for clinicians. Additionally, wild-type Kras tumors are rare in PDAC, limiting the population who could benefit [47]. Furthermore, both of these markers lack a robust theoretical basis. Our research identified MMP-28 as a promising biomarker for EGFR inhibitor therapy and provided the theoretical foundation. In addition, as a secreted protein, MMP-28 can be detected in serum, offering a great advantage. Without surgery, serological tests could be performed to determine the suitability for EGFR inhibitor therapy. Thus, our study offers new hope for therapeutic strategies that block growth signals in PDAC.

Besides MMP-28 serving as a predictive marker for EGFR inhibitor in PDAC, several other MMPs have also been reported to be associated with EGFRs inhibitor therapy. For example, MMP-17 notably increases the sensitivity of tumors to Erlotinib and Palbociclib combination therapy in triple-negative breast cancer [48]. Furthermore, in non-small cell lung cancer, MMP-9 levels have been found to correlate with the tumor’s responsiveness to the combined treatment of celecoxib and Erlotinib [49]. However, these studies mainly rely on clinical data to identify the relationship between MMPs expression and EGFR therapy resistance, with little exploration into the underlying mechanisms. Our study highlights the correlation between MMP-28 and EGFR sensitivity and provides a mechanistic explanation for this relationship. Notably, we find that the regulation of TGF-α by MMP-28 was dependent on its metalloprotease activity. This suggests that other metalloproteases might be involved in similar processes in other tumors. Considering previous reports on the relationship between MMPs and the efficacy of EGFR inhibitors, we believe that the correlation between MMP-28 and EGFR inhibitors in PDAC may be applicable to other cancers as well. Therefore, further investigation into the role of the metalloprotease family in predicting EGFR inhibitor sensitivity is highly warranted.

However, our study also has several limitations. Firstly, due to the absence of specific inhibitors for MMP-28, we don’t explore treatment strategies targeting MMP-28. Furthermore, the current data is largely derived from preclinical studies, and there is a lack of data from clinical research.

## Conclusion

In conclusion, our study found that enhanced MMP-28 contributes to increased TGF-α maturation, which activates EGFR and promoting progression of PDAC. PDAC with high MMP-28 expression shows better sensitivity to Erlotinib. Therefore, using MMP-28 as a biomarker for Erlotinib treatment can help identify responsive patients, thereby improving Erlotinib’s efficacy, reducing its dose and toxicity, and providing greater benefits to patients.

## Supplementary Information

Supplementary Material 1.

## Data Availability

All reagents and mice generated in this study are available from the lead contact with a completed Materials Transfer Agreement. RNA-seq data generated in this study have been deposited at Science Data Bank (10.57760/sciencedb.12954). Proteomic data have been deposited at Science Data Bank (10.57760/sciencedb.20886) and both two are publicly available as of the date of publication. Any additional information required to reanalyze the data reported in this paper is available from the lead contact upon request.

## Abbreviations

AP-1: Activated Protein-1
BCL2L1: B-cell Lymphoma 2-like 1
CCK-8: Cell Counting Kit-8
ChIP: Chromatin Immuno-Precipitation
CI: Confidence Interval
CTLA-4: Cytotoxic T-Lymphocyte Associated Antigen 4
DAB: Diaminobenzidine
DAPI: 4',6-Diamidino-2-phenylindole
DEGs: Differentially Expressed Genes
EGF: Epidermal Growth Factor
EGFR: Epidermal Growth Factor Receptor
ELISA: Enzyme-Linked Immunosorbent Assay
ERK: Extracellular Regulated Protein Kinases
FBS: Fetal Bovine Serum
FDA: Food and Drug Administration
GAPDH: Glyceraldehyde-3-phosphate Dehydrogenase
GDSC: Genomics of Drug Sensitivity in Cancer
GFP: Green fluorescent protein
GSEA: Gene Set Enrichment Analysis
HB-EGF: Heparin-binding EGF-like Growth Factor
HR: Hazard Ratio
IGFR: Insulin-like Growth Factor Receptor
IHC: Immunohistochemical
IP: Immuno-Precipitation
JAK: Janus Kinase
KD: Knockdown
LNM: Lymph node metastasis
MAPK: Mitogen-activated Protein Kinase
MEK: Mitogen-activated Protein Kinase Kinase
mIHC: Multiplex Immunohistochemical
MMP-28: Matrix Metalloproteinase-28
mRNA: Messenger RNA
mTOR: Mammalian Target of Rapamycin
NES: Normalized Enrichment Score
OE: Overexpression
ORR: Objective Response Rate
OS: Overall survival
PBS: Phosphate Buffered Saline
PCR: Polymerase Chain Reaction
PD-1: Programmed Death-1
PDAC: Pancreatic Ductal Adenocarcinoma
PDGFR: Platelet-derived Growth Factor Receptor
PDX: Patient-derived Tumor Xenograft
PI3K: Phosphatidylinositol 3-kinase
PKC: Protein Kinase C
PVDF: Polyvinylidene Fluoride
RFS: Relapse-free survival
RIPA: Radio Immunoprecipitation Assay
RNA: Ribonucleic Acid
RT-PCR: Real-time PCR
RTK: Receptor Tyrosine Kinase
SDS: Sodium dodecyl sulfate
SPF: Specific-pathogen-free
STAT: Signal Transducer and Activator of Transcription
TCGA: The Cancer Genome Atlas
TGF-α: Transforming Growth Factor-α
VEGF: Vascular Endothelial Growth Factor
VEGFR: Vascular Endothelial Growth Factor Receptor
