# Supplementary material for: Reciprocal regulation of MMP-28 and EGFR is required for sustaining proliferative signaling in PDAC
Source: J Exp Clin Cancer Res. 2025 Feb 24;44:68. doi: 10.1186/s13046-025-03323-9 (PMC11849219; doi:10.1186/s13046-025-03323-9)
Supplement: Supplementary file 1 — Supplementary Material 1. [file 13046_2025_3323_MOESM1_ESM.pdf]

Fig. S1

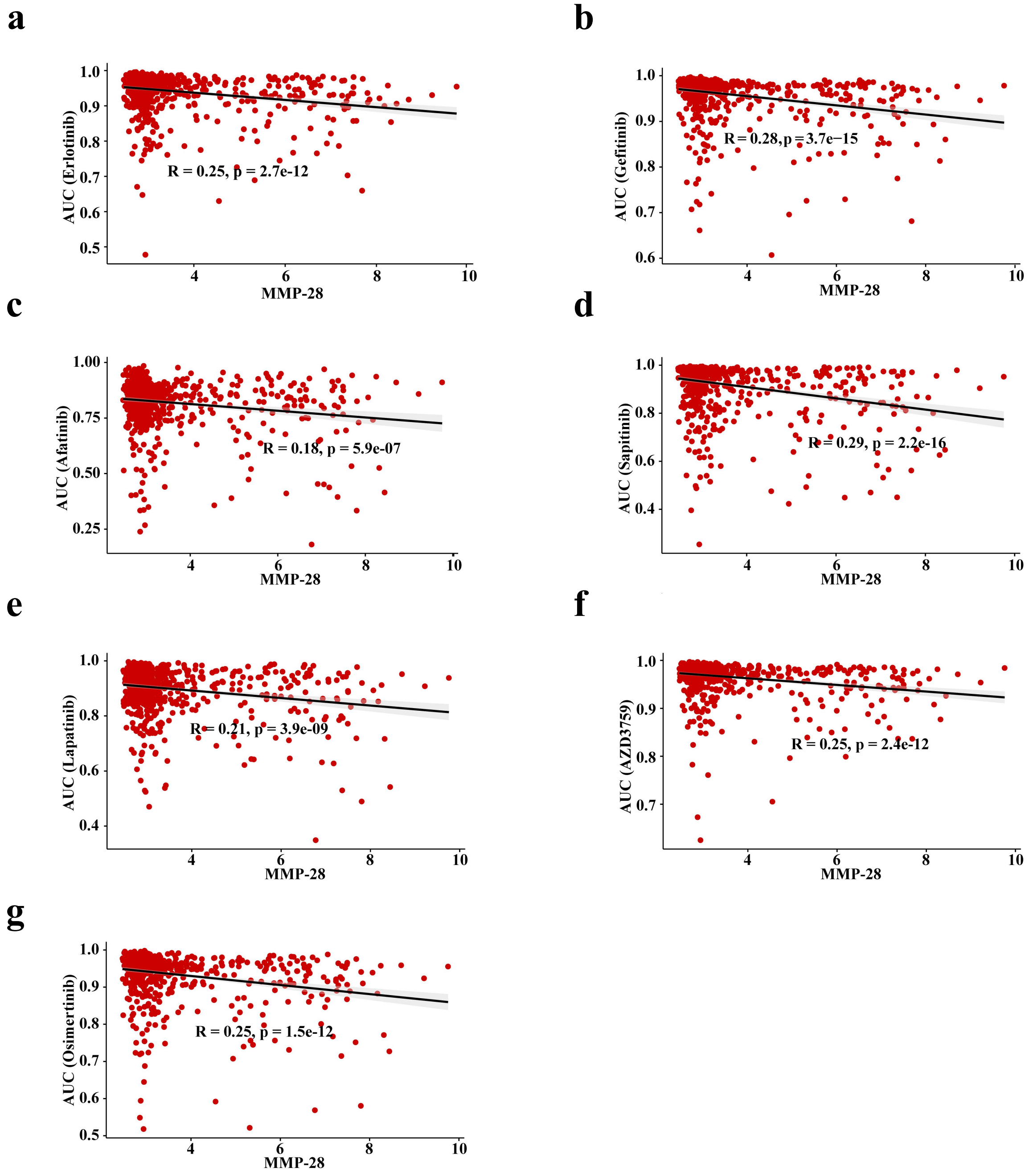
a
AUC (Erlotinib)
R = 0.25, p = 2.7e-12
MMP-28
b
AUC (Gefitinib)
R = 0.28, p = 3.7e−15
MMP-28
c
AUC (Afatinib)
R = 0.18, p = 5.9e-07
MMP-28
d
AUC (Sapitinib)
R = 0.29, p = 2.2e-16
MMP-28
e
AUC (Lapatinib)
R = 0.21, p = 3.9e-09
MMP-28
f
AUC (AZD3759)
R = 0.25, p = 2.4e-12
MMP-28
g
AUC (Osimertinib)
R = 0.25, p = 1.5e-12
MMP-28

Fig.S1 MMP-28 is associated with sensitivity to EGFR inhibitors

(a-g) Correlation between MMP-28 expression levels and AUC of multitype EGFR inhibitors across pan-cancer cell lines. Data was from GDSC. The correlation with Erlotinib was shown in **a**. The correlation with Gefitinib was shown in **b**. The correlation with Afatinib was shown in **c**. The correlation with Saptinib was shown in **d**. The correlation with Lapatinib was shown in **e**. The correlation with AZD3759 was shown in **f**. The correlation with Osimertinib was shown in **g**.

Fig. S2

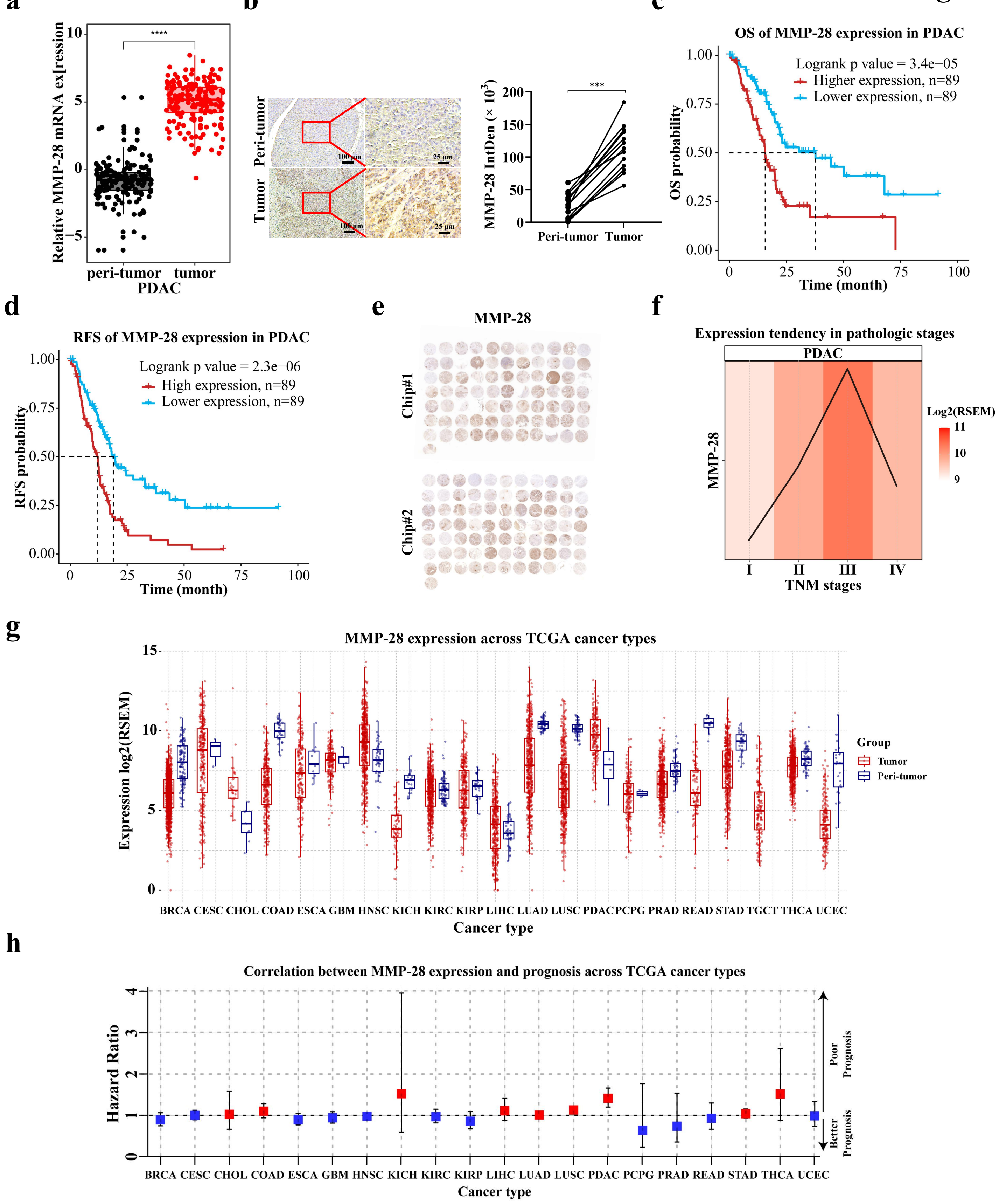
a
b
c
d
e
f
g
h
Relative MMP-28 mRNA ex[ression
****
peri-tumor
tumor
PDAC
Peri-tumor
Tumor
100 μm
25 μm
MMP-28 IntDen (× 10³)
***
Peri-tumor
Tumor
OS of MMP-28 expression in PDAC
Logrank p value = 3.4e−05
Higher expression, n=89
Lower expression, n=89
OS probability
Time (month)
RFS of MMP-28 expression in PDAC
Logrank p value = 2.3e−06
High expression, n=89
Lower expression, n=89
RFS probability
Time (month)
MMP-28
Chip#1
Chip#2
Expression tendency in pathologic stages
PDAC
MMP-28
Log2(RSEM)
I
II
III
IV
TNM stages
MMP-28 expression across TCGA cancer types
Expression log2(RSEM)
Group
Tumor
Peri-tumor
BRCA CESC CHOL COAD ESCA GBM HNSC KICH KIRC KIRP LIHC LUAD LUSC PDAC PCPG PRAD READ STAD TGCT THCA UCEC
Cancer type
Correlation between MMP-28 expression and prognosis across TCGA cancer types
Hazard Ratio
Poor Prognosis
Better Prognosis
BRCA CESC CHOL COAD ESCA GBM HNSC KICH KIRC KIRP LIHC LUAD LUSC PDAC PCPG PRAD READ STAD THCA UCEC
Cancer type

Fig.S2 Expression characteristic and prognostic relevance of MMP-28 in PDAC and across multiple cancer types

(a) Expression of MMP-28 in tumor tissues of PDAC and peri-tumor tissues was compared. Data was from TCGA database. **(b)** IHC staining analysis of MMP-28 was performed in paired tissue samples from PDAC patients (n = 14) (P: Peri-tumor tissue; T: Tumor tissue). Integrated density (IntDen) was used to represent the relative expression level of MMP-28. **(c)** Correlation of MMP-28 with OS of PDAC patients was analyzed. Data was from TCGA database. **(d)** Correlation of MMP-28 with RFS of PDAC patients was analyzed. Data was from TCGA database. **(e)** Tissue microarrays composed of 156 cases of PDAC tumors were used for IHC staining analysis of MMP-28, overview image were shown. **(f)** The correlation between the expression of MMP-28 and the TNM stages of PDAC was analyzed and shown. Data sourced from TCGA database and was analyzed using the GSCA website. **(g)** Expression of MMP-28 in tumor tissues of multiple cancers and peri-tumor tissues was compared. Data was from TCGA database. **(h)** Correlation of MMP-28 with OS of multiple cancers patients was analyzed. Data was from TCGA database. $^{***}p < 0.001$, $^{****}p < 0.0001$.

Fig. S3

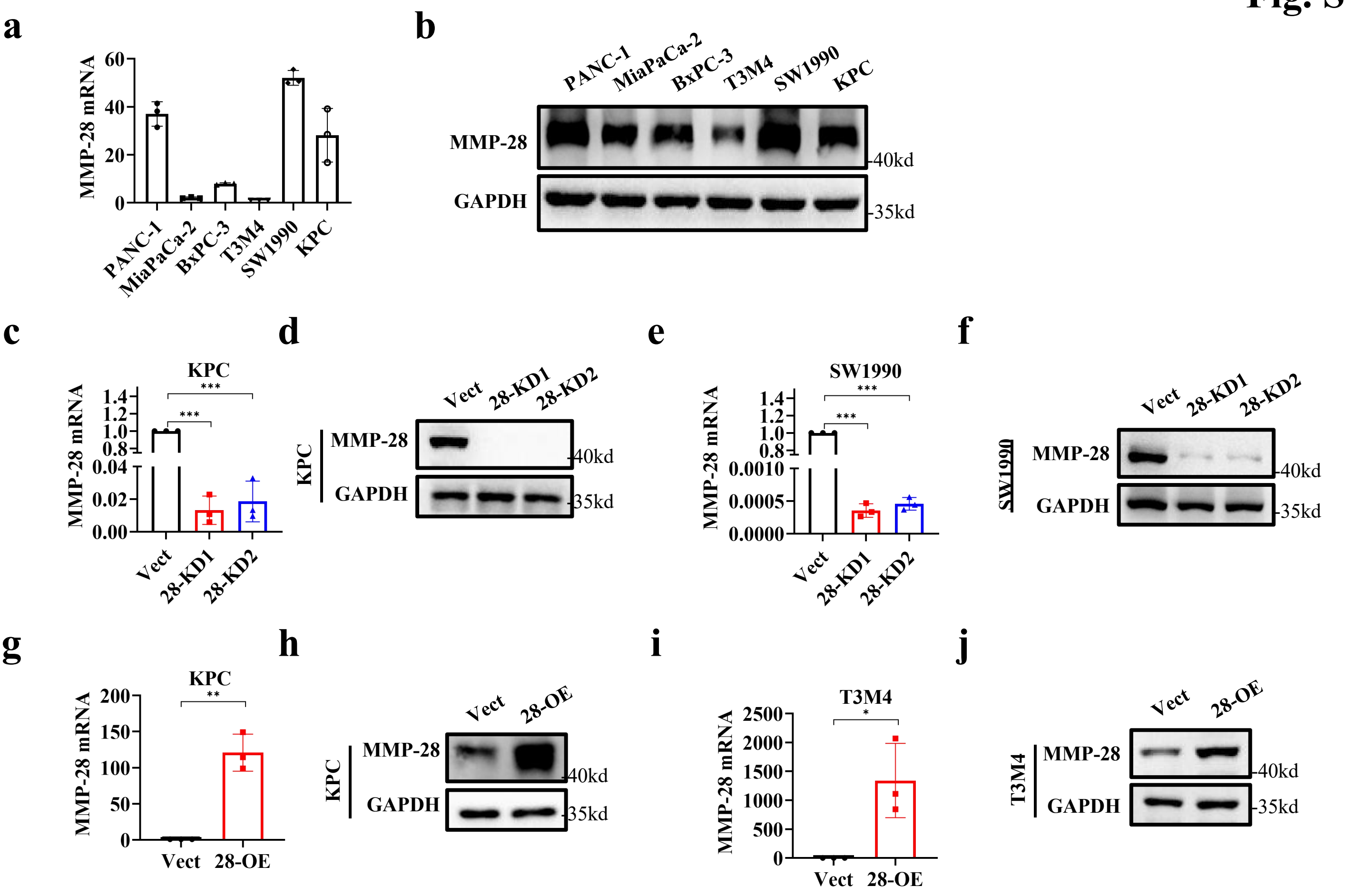
a
MMP-28 mRNA
60
40
20
0
PANC-1
MiaPaCa-2
BxPC-3
T3M4
SW1990
KPC
b
PANC-1
MiaPaCa-2
BxPC-3
T3M4
SW1990
KPC
MMP-28
40kd
GAPDH
35kd
c
KPC
MMP-28 mRNA
***
***
1.4
1.2
1.0
0.8
0.04
0.02
0.00
Vect
28-KD1
28-KD2
d
Vect
28-KD1
28-KD2
KPC
MMP-28
40kd
GAPDH
35kd
e
SW1990
MMP-28 mRNA
***
***
1.4
1.2
1.0
0.8
0.0010
0.0005
0.0000
Vect
28-KD1
28-KD2
f
Vect
28-KD1
28-KD2
SW1990
MMP-28
40kd
GAPDH
35kd
g
KPC
**
MMP-28 mRNA
200
150
100
50
0
Vect
28-OE
h
Vect
28-OE
KPC
MMP-28
40kd
GAPDH
35kd
i
T3M4
*
MMP-28 mRNA
2500
2000
1500
1000
500
0
Vect
28-OE
j
Vect
28-OE
T3M4
MMP-28
40kd
GAPDH
35kd

Fig.S3 Detection of MMP-28 expression in multitype PDAC cells

(a) RT-PCR was utilized to detect the RNA level of MMP-28 in multitype PDAC cells. **(b)** Immunoblotting was utilized to detect the expression of MMP-28 in multitype PDAC cells. **(c)** RT-PCR was utilized to detect the RNA level of MMP-28 in KPC cells transfected with control vector (Vect) and MMP-28 KD shRNA (28-KD1 and 28-KD2). **(d)** Immunoblotting was utilized to detect the expression of MMP-28 in KPC cells transfected with Vect and 28-KD1 and 28-KD2. **(e)** RT-PCR was utilized to detect the RNA level of MMP-28 in SW1990 cells transfected with Vect and 28-KD1 and 28-KD2. **(f)** Immunoblotting was utilized to detect the expression of MMP-28 in SW1990 cells transfected with Vect and 28-KD1 and 28-KD2. **(g)** RT-PCR was utilized to detect the RNA level of MMP-28 in KPC cells transfected with Vect and MMP-28 overexpression plasmid (28-OE). **(h)** Immunoblotting was utilized to detect the expression of MMP-28 in KPC cells transfected with Vect and 28-OE. **(i)** RT-PCR was utilized to detect the RNA level of MMP-28 in T3M4 cells transfected with Vect and MMP-28 28-OE. **(j)** Immunoblotting was utilized to detect the expression of MMP-28 in T3M4 cells transfected with Vect and 28-OE. $^{*}p < 0.05$; $^{**}p < 0.01$; $^{***}p < 0.001$.

Fig. S4

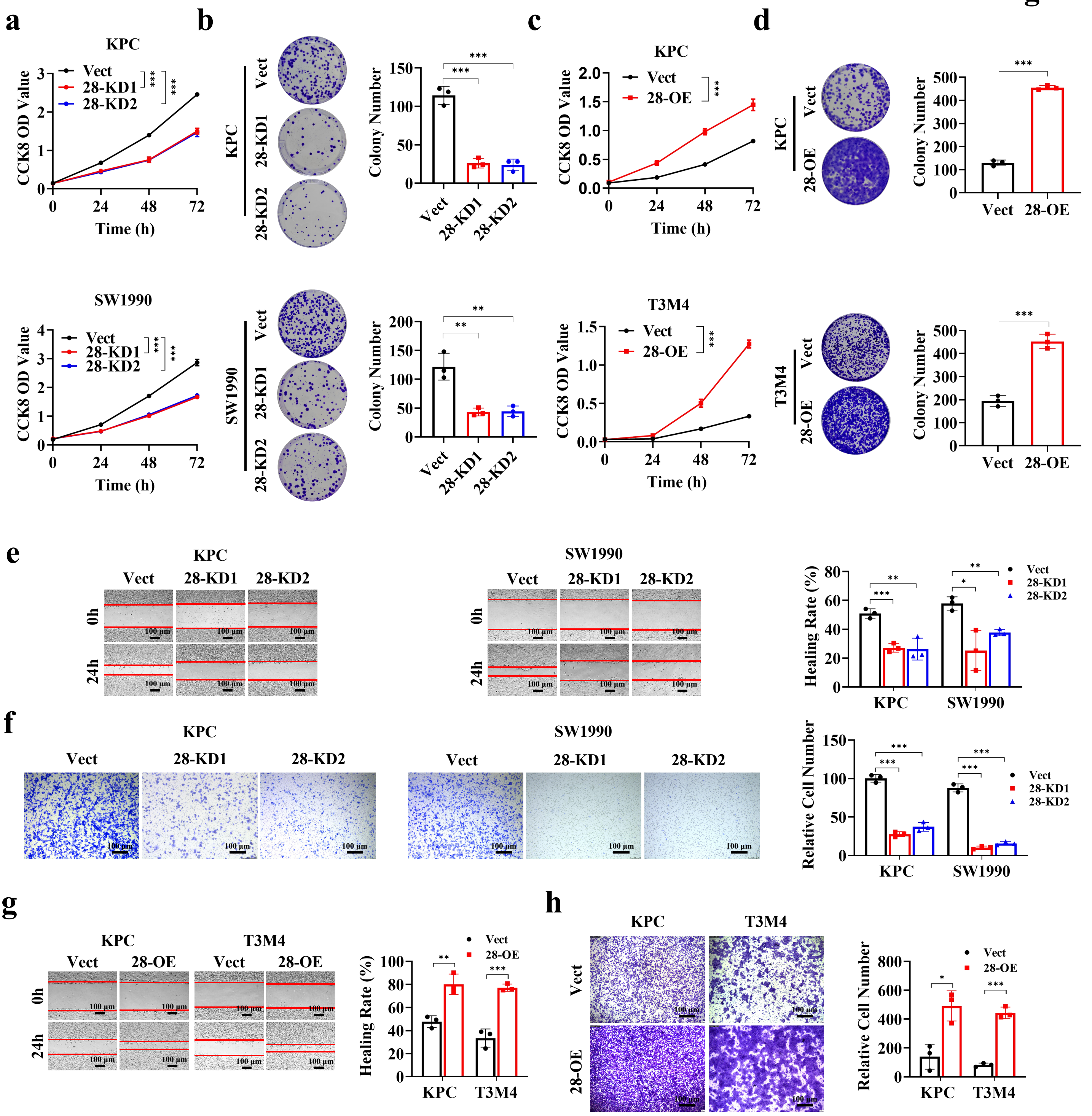
a
KPC
CCK8 OD Value
Time (h)
Vect
28-KD1
28-KD2
SW1990
b
Vect
28-KD1
28-KD2
Colony Number
c
KPC
28-OE
T3M4
d
e
0h
24h
Healing Rate (%)
f
Relative Cell Number
g
h
100 μm

Fig.S4 MMP-28 promotes PDAC cell proliferation and migration

(a) CCK8 assay was performed to detect the proliferation of PDAC cells with MMP-28 KD or not. **(b)** Colony formation assay was performed to detect the proliferation of PDAC cells with MMP-28 KD or not. **(c)** CCK8 assay was performed to detect the proliferation of PDAC cells with MMP-28 OE or not. **(d)** Colony formation assay was performed to detect the proliferation of PDAC cells with MMP-28 OE or not. **(e)** Wound healing assay was performed to detect the migration of PDAC cells with MMP-28 KD or not. **(f)** Transwell assay was performed to detect the migration of PDAC cells with MMP-28 KD or not. **(g)** Wound healing assay was performed to detect the migration of PDAC cells with MMP-28 OE or not. **(h)** Transwell assay was performed to detect the migration of PDAC cells with MMP-28 OE or not. $^{*}p < 0.05$; $^{**}p < 0.01$; $^{***}p < 0.001$.

Fig. S5

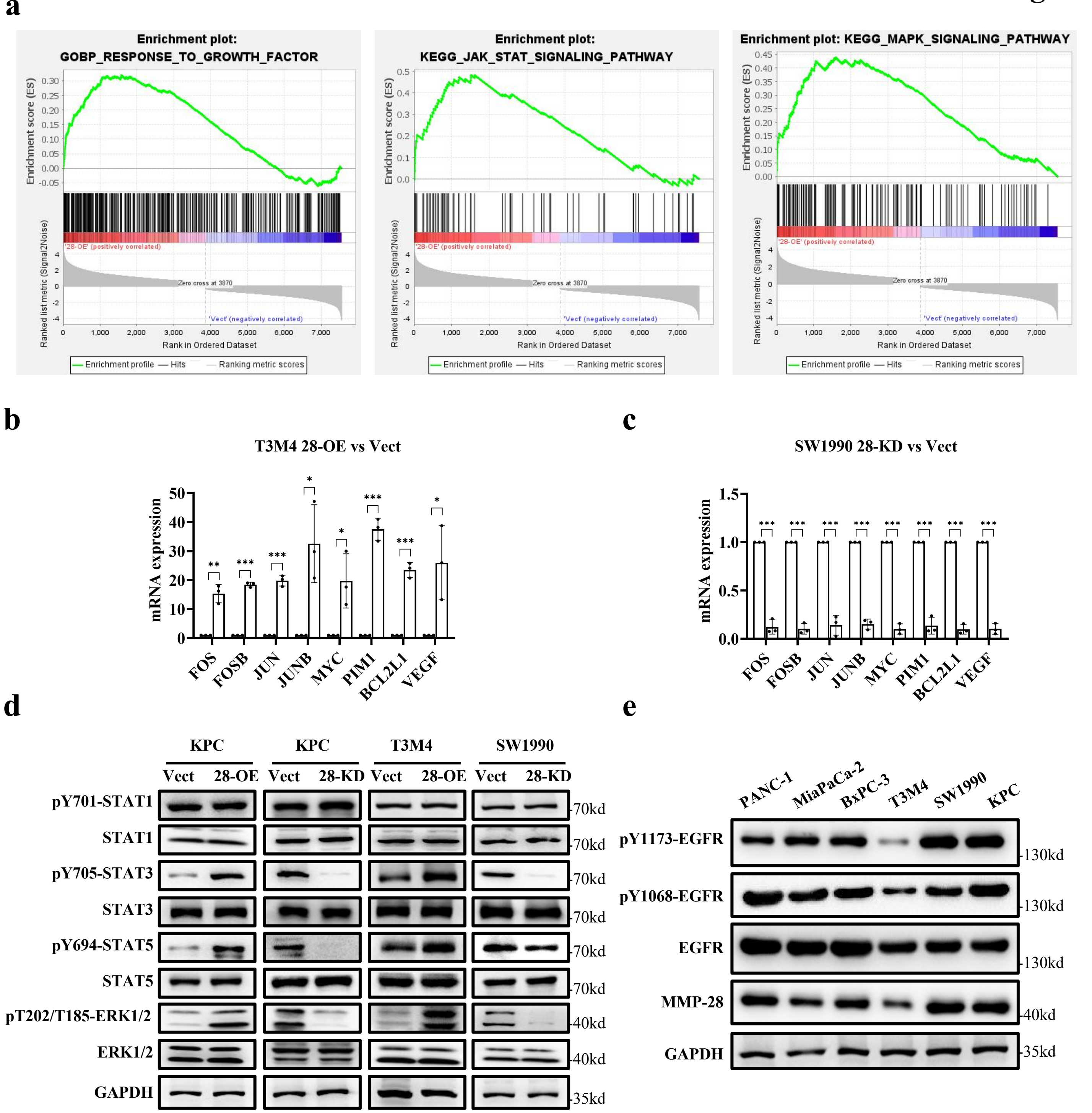
a
Enrichment plot: GOBP_RESPONSE_TO_GROWTH_FACTOR
Enrichment plot: KEGG_JAK_STAT_SIGNALING_PATHWAY
Enrichment plot: KEGG_MAPK_SIGNALING_PATHWAY
Enrichment score (ES)
Ranked list metric (Signal2Noise)
'28-OE' (positively correlated)
Zero cross at 3870
'Vect' (negatively correlated)
Rank in Ordered Dataset
Enrichment profile — Hits — Ranking metric scores
b
T3M4 28-OE vs Vect
mRNA expression
FOS FOSB JUN JUNB MYC PIM1 BCL2L1 VEGF
c
SW1990 28-KD vs Vect
mRNA expression
FOS FOSB JUN JUNB MYC PIM1 BCL2L1 VEGF
d
KPC KPC T3M4 SW1990
Vect 28-OE Vect 28-KD Vect 28-OE Vect 28-KD
pY701-STAT1
STAT1
pY705-STAT3
STAT3
pY694-STAT5
STAT5
pT202/T185-ERK1/2
ERK1/2
GAPDH
-70kd
-40kd
-35kd
e
PANC-1 MiaPaCa-2 BxPC-3 T3M4 SW1990 KPC
pY1173-EGFR
pY1068-EGFR
EGFR
MMP-28
GAPDH
-130kd
-40kd
-35kd

Fig.S5 MMP-28 promotes EGFR signaling pathway activation

(a) GO enrichment analysis was performed for differentially signaling pathway related to EGFR between Vect and 28-OE T3M4 cells from the transcriptomic analysis. **(b, c)** RT-PCR was employed to discern the transcriptional abundance of EGFR downstream genes. Regulatory role of MMP-28 OE on T3M4 cells were shown in **b**. Regulatory role of MMP-28 KD on SW1990 cells were shown in **c**. **(d)** Immunoblotting was employed to verify protein level changes of EGFR downstream genes and pathway in multiple PDAC cell lines with MMP-28 KD or OE. **(e)** Immunoblotting was utilized to detect the expression of MMP-28 and phosphorylated and total EGFR in multitype PDAC cells. $^{*}p < 0.05$; $^{**}p < 0.01$; $^{***}p < 0.001$.

Fig. S6

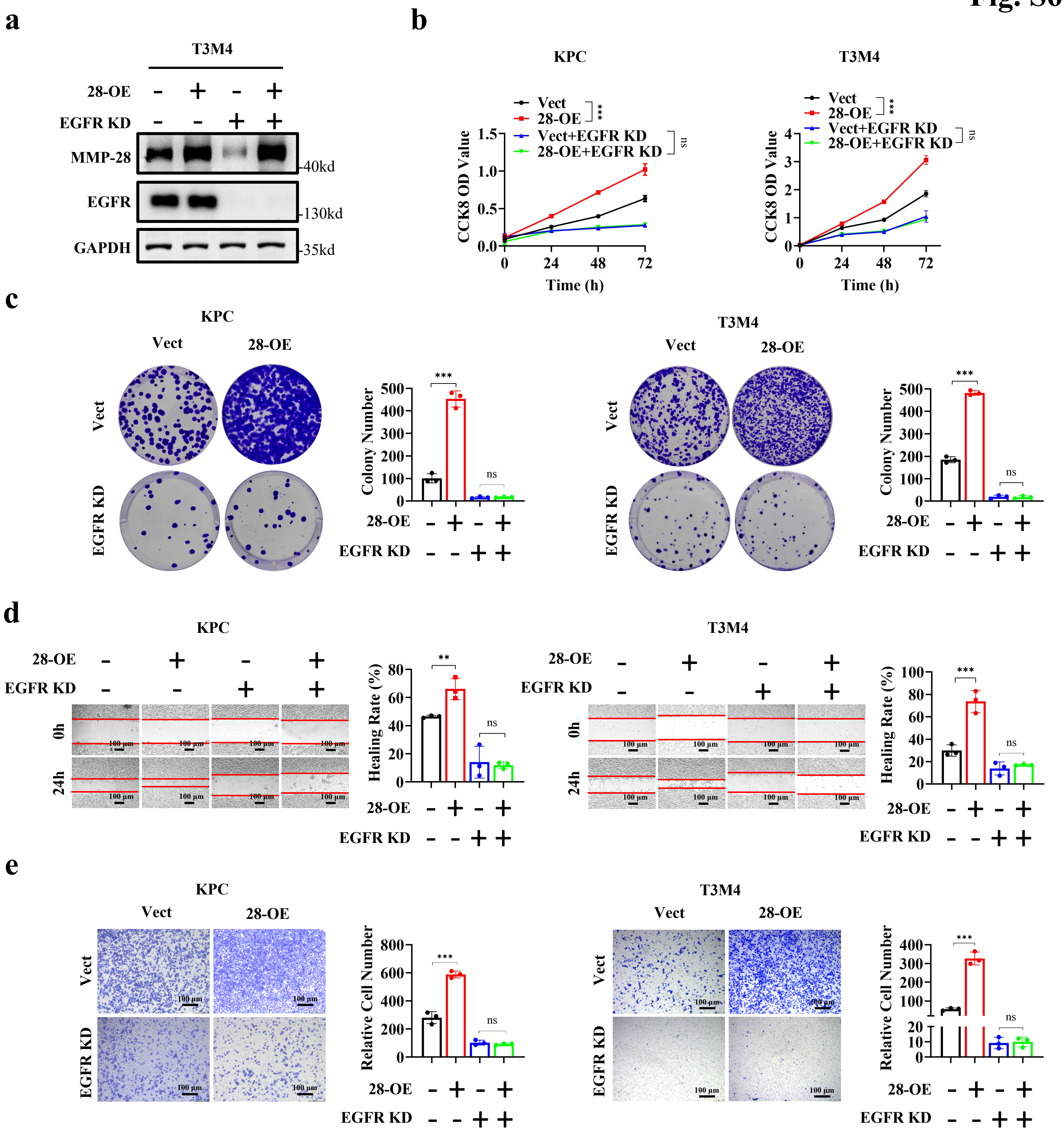
a
T3M4
28-OE
EGFR KD
MMP-28
EGFR
GAPDH
40kd
130kd
35kd
b
KPC
T3M4
Vect
28-OE
Vect+EGFR KD
28-OE+EGFR KD
***
ns
CCK8 OD Value
Time (h)
c
KPC
T3M4
Vect
28-OE
EGFR KD
***
ns
Colony Number
28-OE
EGFR KD
d
KPC
T3M4
28-OE
EGFR KD
0h
24h
100 μm
**
***
ns
Healing Rate (%)
e
KPC
T3M4
Vect
28-OE
EGFR KD
100 μm
***
ns
Relative Cell Number
28-OE
EGFR KD

Fig.S6 MMP-28 promotes PDAC cell proliferation and migration by EGFR

(a) Transfection of EGFR KD shRNA with vector was performed on Vect and 28-OE T3M4 cells. Immunoblotting was utilized to detect the expression of MMP-28 and EGFR. **(b)** CCK8 assay was performed to detect the effect of MMP-28 OE on cell proliferation in PDAC cells with EGFR KD or not. **(c)** Colony formation assay was performed to detect the effect of MMP-28 OE on cell proliferation in PDAC cells with EGFR KD or not. **(d)** Wound healing assay was performed to detect the effect of MMP-28 OE on cell migration in PDAC cells with EGFR KD or not. **(e)** Transwell assay was performed to detect the effect of MMP-28 OE on cell migration in PDAC cells with EGFR KD or not. $^{**}p < 0.01$; $^{***}p < 0.001$; ns, not significant.

Fig. S7

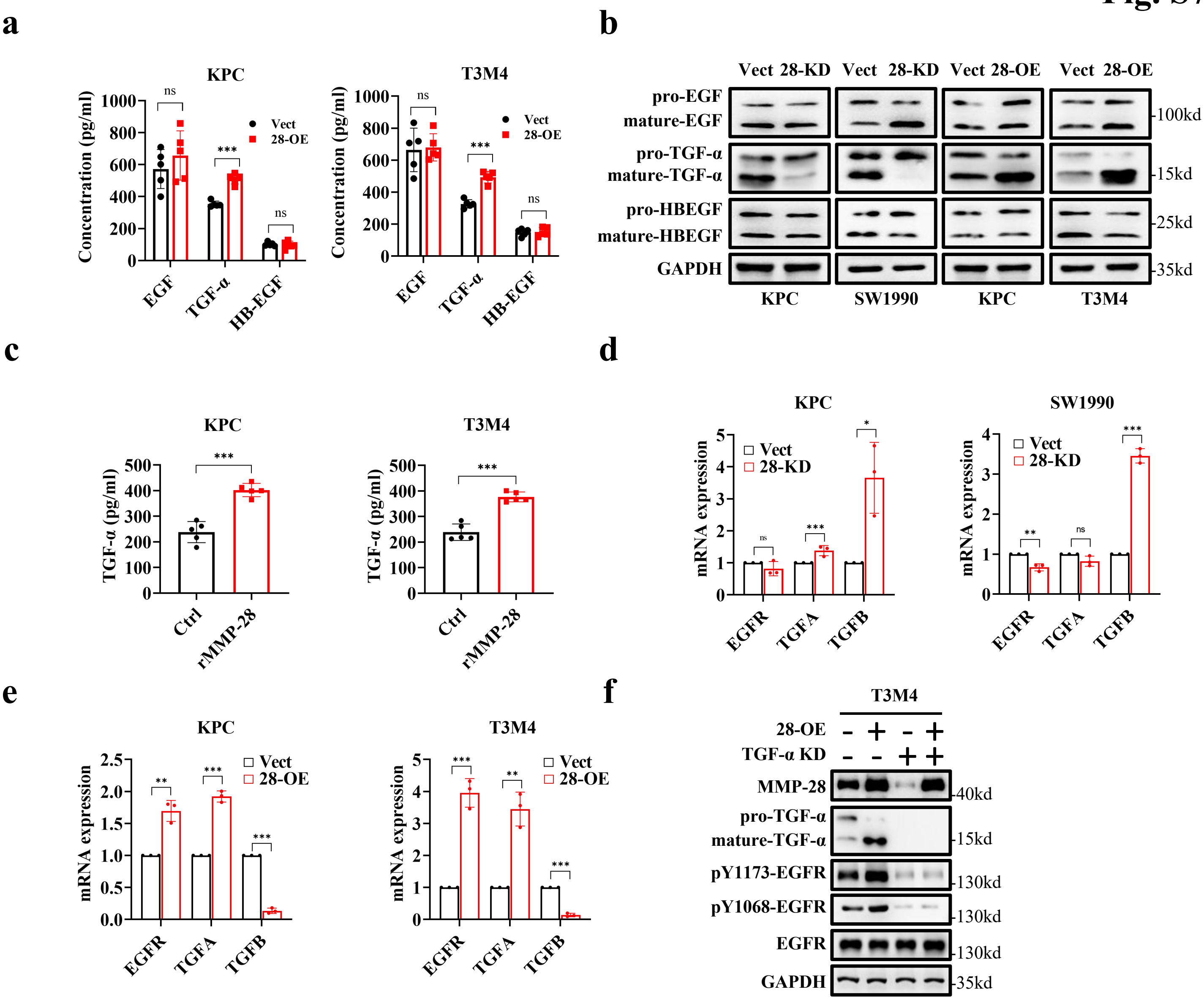

a
KPC
T3M4
Concentration (pg/ml)
Vect
28-OE
ns
***
EGF
TGF-α
HB-EGF
b
Vect 28-KD Vect 28-KD Vect 28-OE Vect 28-OE
pro-EGF
mature-EGF
pro-TGF-α
mature-TGF-α
pro-HBEGF
mature-HBEGF
GAPDH
100kd
15kd
25kd
35kd
KPC SW1990 KPC T3M4
c
KPC
T3M4
TGF-α (pg/ml)
Ctrl
rMMP-28
d
KPC
SW1990
mRNA expression
Vect
28-KD
EGFR
TGFA
TGFB
e
KPC
T3M4
mRNA expression
Vect
28-OE
EGFR
TGFA
TGFB
f
T3M4
28-OE
TGF-α KD
MMP-28
pro-TGF-α
mature-TGF-α
pY1173-EGFR
pY1068-EGFR
EGFR
GAPDH
40kd
15kd
130kd
130kd
130kd
35kd

Fig.S7 TGF-α is identified as MMP-28 downstream effector

(a) ELISA was performed to detect soluble EGF, TGF-α, and HB-EGF in supernatant of PDAC cells with MMP-28 OE. **(b)** Immunoblotting was performed to assess TGF-α in PDAC cells with MMP-28 KD or OE. **(c)** ELISA was performed to detect soluble EGF, TGF-α, and HB-EGF in supernatant of PDAC cells with rMMP-28 treated. **(d)** RT-PCR was performed to detect the RNA level of EGFR, TGF-α and TGF-β in PDAC cells with MMP-28 KD or not. **(e)** RT-PCR was performed to detect the RNA level of EGFR, TGF-α and TGF-β in PDAC cells with MMP-28 OE or not. **(f)** Transfection of TGF-α KD shRNA with vector was performed on Vect and 28-OE T3M4 cells. Immunoblotting was utilized to detect the expression of MMP-28 and TGF-α. $^{*}p < 0.05$, $^{**}p < 0.01$; $^{***}p < 0.001$; ns, not significant.

Fig. S8

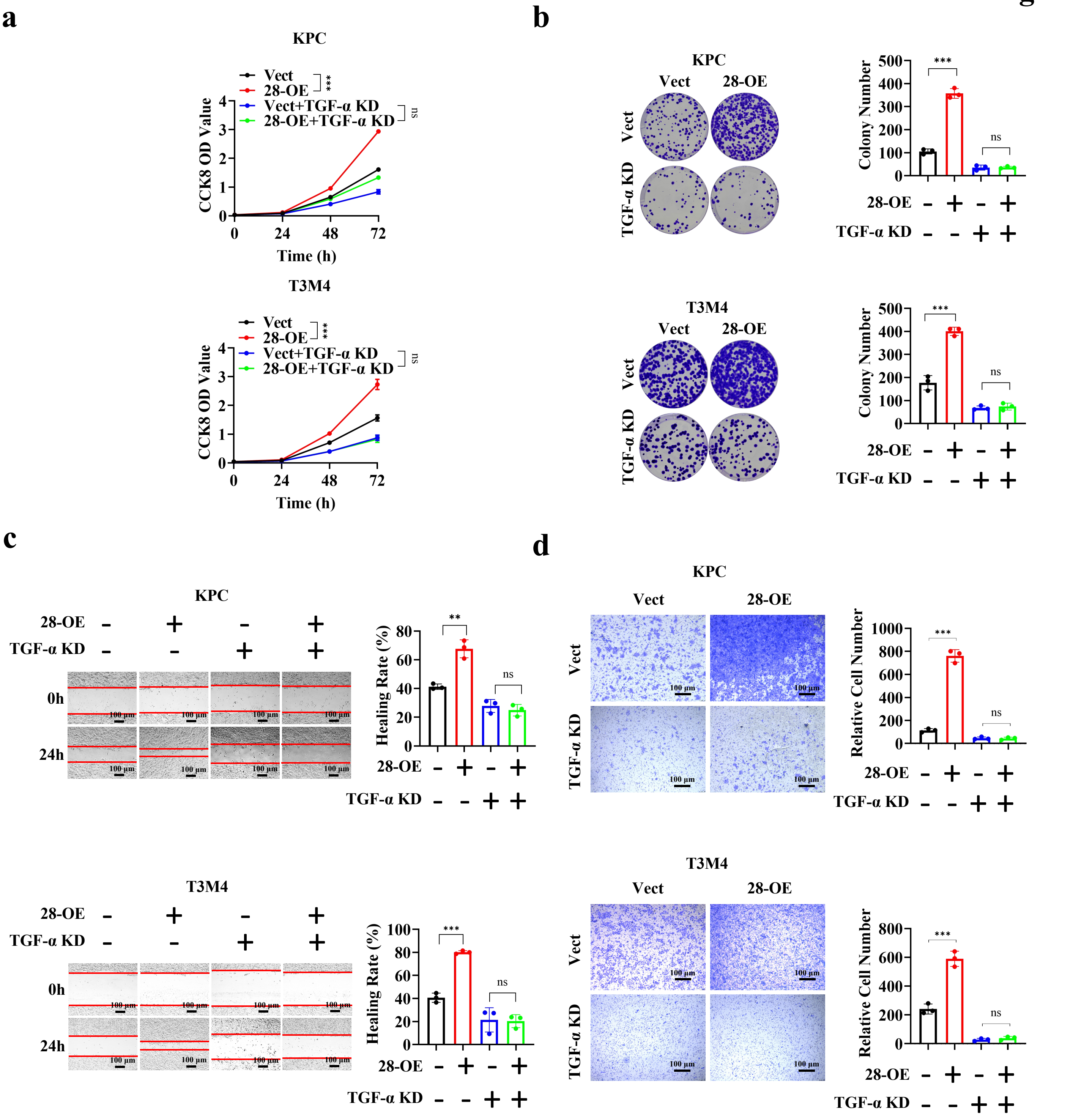
a
KPC
Vect
28-OE
Vect+TGF-α KD
28-OE+TGF-α KD
***
ns
CCK8 OD Value
Time (h)
0
24
48
72
T3M4
b
KPC
Vect
28-OE
TGF-α KD
Colony Number
***
ns
28-OE
TGF-α KD
T3M4
c
KPC
0h
24h
100 μm
Healing Rate (%)
**
ns
T3M4
***
d
KPC
Vect
28-OE
TGF-α KD
Relative Cell Number
***
ns
T3M4

Fig.S8 MMP-28 promotes proliferation and migration of PDAC through TGF-α

(a) CCK8 assay was performed to detect the effect of MMP-28 OE on cell proliferation in PDAC cells with TGF-α KD. **(b)** Colony formation assay was performed to detect the effect of MMP-28 OE on cell proliferation in PDAC cells with TGF-α KD. **(c)** Wound healing assay was performed to detect the effect of MMP-28 OE on cell migration in PDAC cells with TGF-α KD. **(d)** Transwell assay was performed to detect the effect of MMP-28 OE on cell migration in PDAC cells with TGF-α KD. $^{**}p < 0.01$; $^{***}p < 0.001$; ns, not significant.

Fig. S9

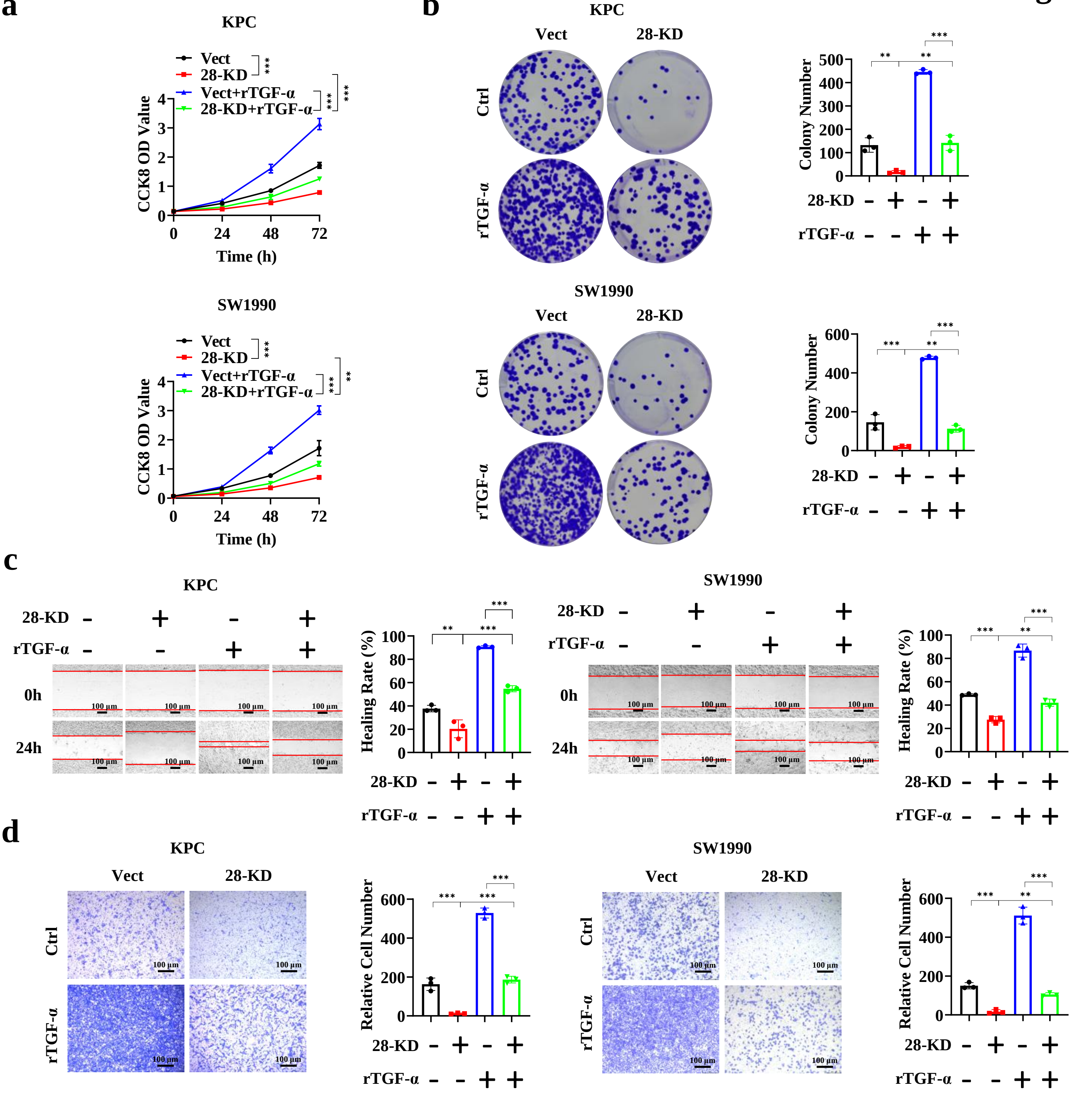

a
KPC
Vect
28-KD
Vect+rTGF-α
28-KD+rTGF-α
CCK8 OD Value
Time (h)
SW1990
b
KPC
Vect
28-KD
Ctrl
rTGF-α
Colony Number
SW1990
c
Healing Rate (%)
0h
24h
100 μm
d
Relative Cell Number

Fig.S9 Sustaining TGF-α-mediated proliferative signaling rely on MMP-28

(a) CCK8 assay was performed to detect the effect of exogenous recombinant TGF-α on cell proliferation in PDAC cells with MMP-28 KD. **(b)** Colony formation assay was performed to detect the effect of exogenous recombinant TGF-α on cell proliferation in PDAC cells with MMP-28 KD. **(c)** Wound healing assay was performed to detect the effect of exogenous recombinant TGF-α on cell migration in PDAC cells with MMP-28 KD. **(d)** Transwell assay was performed to detect the effect of exogenous recombinant TGF-α on cell migration in PDAC cells with MMP-28 KD. $^{**}p < 0.01$; $^{***}p < 0.001$.

Fig. S10

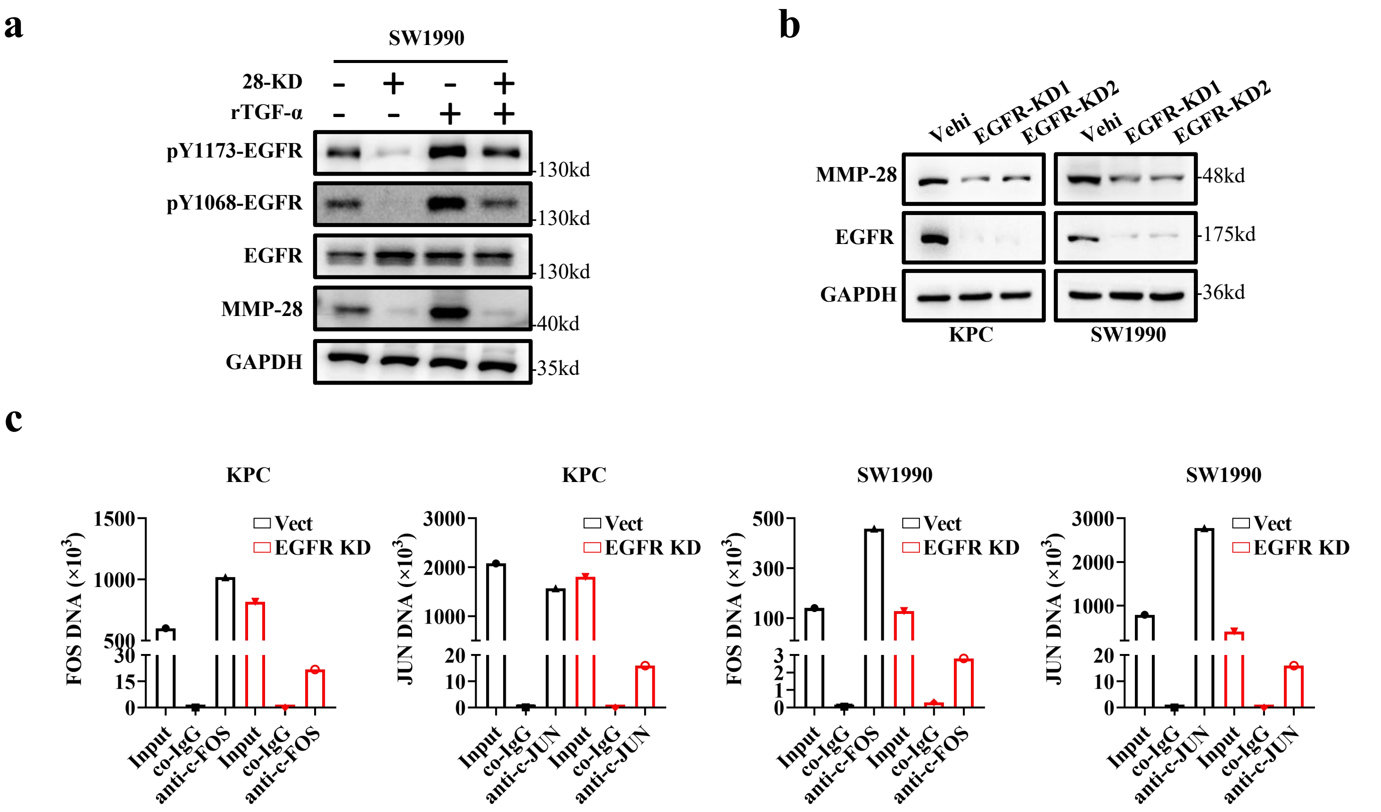
a
SW1990
28-KD
rTGF-α
pY1173-EGFR
pY1068-EGFR
EGFR
MMP-28
GAPDH
130kd
130kd
130kd
40kd
35kd
b
Vehi
EGFR-KD1
EGFR-KD2
MMP-28
EGFR
GAPDH
48kd
175kd
36kd
KPC
SW1990
c
KPC
SW1990
Vect
EGFR KD
FOS DNA (×10³)
JUN DNA (×10³)
Input
co-IgG
anti-c-FOS
anti-c-JUN

Fig.S10 Activated EGFR transcriptionally upregulates MMP-28

(a) Vect and 28-KD SW1990 cells were treated with exogenous recombinant TGF-α. Immunoblotting was utilized to detect the expression of MMP-28 and phosphorylated and total EGFR. **(b)** Immunoblotting was performed to detect MMP-28 in PDAC cells with EGFR KD or not. **(c)** CHIP and qPCR detected the binding of c-JUN and c-FOS to the MMP-28 promoter in PDAC cells with EGFR KD or not.

Fig. S11

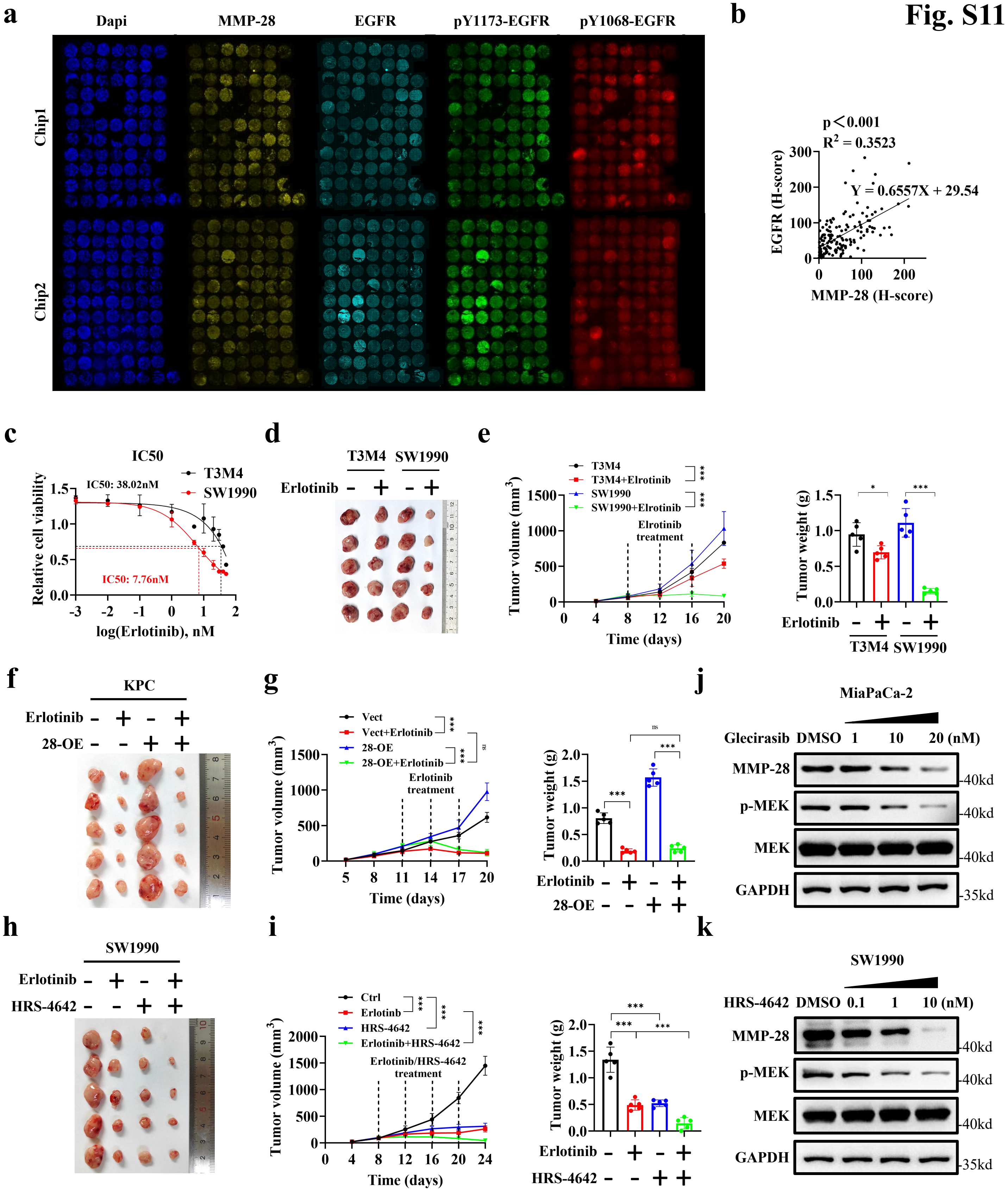
a
Dapi
MMP-28
EGFR
pY1173-EGFR
pY1068-EGFR
Chip1
Chip2
b
p＜0.001
R2 = 0.3523
Y = 0.6557X + 29.54
EGFR (H-score)
MMP-28 (H-score)
c
IC50
T3M4
SW1990
IC50: 38.02nM
IC50: 7.76nM
Relative cell viability
log(Erlotinib), nM
d
T3M4
SW1990
Erlotinib
e
T3M4
T3M4+Elrotinib
SW1990
SW1990+Elrotinib
Elrotinib treatment
Tumor volume (mm3)
Time (days)
Tumor weight (g)
Erlotinib
T3M4
SW1990
f
KPC
Erlotinib
28-OE
g
Vect
Vect+Erlotinib
28-OE
28-OE+Erlotinib
Erlotinib treatment
Tumor volume (mm3)
Time (days)
Tumor weight (g)
Erlotinib
28-OE
j
MiaPaCa-2
Glecirasib DMSO 1 10 20 (nM)
MMP-28
p-MEK
MEK
GAPDH
40kd
35kd
h
SW1990
Erlotinib
HRS-4642
i
Ctrl
Erlotinib
HRS-4642
Erlotinib+HRS-4642
Erlotinib/HRS-4642 treatment
Tumor volume (mm3)
Time (days)
Tumor weight (g)
Erlotinib
HRS-4642
k
SW1990
HRS-4642 DMSO 0.1 1 10 (nM)
MMP-28
p-MEK
MEK
GAPDH
40kd
35kd

Fig.S11 MMP-28 determines therapeutic efficacy of EGFR inhibitor in PDAC

(a, b) Tissue microarrays composed of 156 cases of PDAC tumors were used for multicolor immunohistochemical staining analysis. Overview images of individual channels were shown in **a**. Correlation analysis between MMP-28 and EGFR was presented in **b**. **(c)** CCK-8 assay was performed to detect the IC50 of Erlotinib in T3M4 and SW1990 cells. **(d, e)** T3M4 and SW1990 cells were injected subcutaneously into nude mice with Erlotinib treatment. Representative images were shown in **d**. Tumor growth curve with Erlotinib treatment indicated by dashed lines, and tumor weight were shown in **e**. **(f, g)** KPC cells with MMP-28 OE or not were injected subcutaneously into C57BL/6J mice with Erlotinib treatment. Representative images were shown in **f**. Tumor growth curve with Erlotinib treatment indicated by dashed lines, and tumor weight were shown in **g**. **(h, i)** SW1990 cells were injected subcutaneously into nude mice with Erlotinib and HRS-4642 treatment. Representative images were shown in **h**. Tumor growth curve with Erlotinib treatment indicated by dashed lines, and tumor weight were shown in **i**. **(j)** Immunoblotting was performed to detect MMP-28 in MiaPaCa-2 cells with Glecirasib treatment. **(k)** Immunoblotting was performed to detect MMP-28 in SW1990 cells with HRS-4642 treatment. $^{*}p < 0.05$; $^{**}p < 0.01$; $^{***}p < 0.001$; ns, not significant.

**Table S1 differentially expressed genes in proteomics, related to Fig. 1a**		
**Gene symbol**	**Log_2FoldChange**	**P value**
FOSL1	-3.824377157	7.31557E-06
MMP28	-3.749317244	4.28662E-05
PRXL2C	-3.316399008	4.77268E-06
KIF2C	-3.135067946	0.00022525
ECT2	-3.056473736	1.84973E-07
KIF20A	-3.053628177	0.000660069
MASTL	-2.995157112	0.000261588
SLC12A6	-2.860234918	0.000288002
PTX3	-2.847504099	3.23982E-05
IL1A	-2.841520811	0.000745326
SKP2	-2.824195911	0.000144701
SNX13	-2.768338819	0.000869718
BCAR3	-2.746573657	0.000974711
CKAP2L	-2.656368874	0.000103409
LEO1	-2.60798652	7.24999E-05
BBX	-2.479090662	0.000397574
SMN1; SMN2	-2.348755122	6.3585E-07
DR1	2.434084954	0.000395151
OSBPL2	2.469066959	3.41037E-05
TAF6L	2.572889668	0.000184747
PDCD4	2.63317322	4.51914E-08
TXNIP	2.719510872	3.42107E-06
CEP164	2.781504172	3.50424E-06
GSE1	3.280062633	0.000112021
PRKAG2	3.29199006	0.000264134
VPS37A	3.779310787	9.18423E-05
TFPI	7.643711913	0.000405249

Table S2 Genes significantly associated with AUC of Erlotinib, related to Fig. 1b

Gene symbol	r value	p value
EGFR	-0.426658413	0
GJB3	-0.41626819	0
MIR205HG	-0.404418244	0
ALS2CL	-0.400325822	0
GJB5	-0.399123563	0
FGFBP1	-0.394121331	0
KLF5	-0.393802851	0
LAMC2	-0.392219117	0
GPR87	-0.391550376	0
TRIM29	-0.389292205	0
SFN	-0.382805821	0
ITGB6	-0.375665804	0
RHOD	-0.374177515	0
XDH	-0.372986437	0
LAMB3	-0.371360617	0
LAMA5	-0.370628334	0
CDH3	-0.369895408	0
KRT15	-0.366606259	0
CRB3	-0.366594344	0
SERPINB5	-0.365225674	0
C19orf33	-0.363748416	0
INADL	-0.362430415	0
SSH3	-0.361008454	0
FAM83B	-0.360900512	0
MPZL2	-0.358651734	0
TACSTD2	-0.358104698	0
MAL2	-0.357591319	0
COL17A1	-0.356675941	0
PPL	-0.35600386	0
C1orf116	-0.355419336	0
LCN2	-0.352454294	0
FAM83H	-0.352198508	0
TSPAN1	-0.351102437	0

LAMA3	-0.349391765	0
ARHGEF5	-0.348873945	0
ITGB4	-0.348687962	0
SLPI	-0.348093857	0
ANXA3	-0.347873463	0
KRT6A	-0.345679429	0
IRF6	-0.344789235	0
S100A2	-0.344584735	0
IL18	-0.343995145	0
SH2D3A	-0.343718212	0
SPINT1	-0.342705897	0
S100A14	-0.339618959	0
DDR1	-0.338665702	0
CAPN1	-0.337444695	0
CDS1	-0.336235099	0
F3	-0.33615291	0
UCA1	-0.33588708	0
TMC4	-0.335316131	0
CYP4F11	-0.33276719	0
F11R	-0.332272968	0
PRRG4	-0.332142719	0
TINAGL1	-0.332101489	0
PKP3	-0.331663647	0
KIAA1522	-0.331446856	0
SCEL	-0.33060646	0
LSR	-0.329511259	0
KLK8	-0.322619116	0
JUP	-0.322228899	0
CDH1	-0.3217597	0
CST6	-0.321509529	0
GPRC5A	-0.321464328	0
TMEM40	-0.319169184	0
TBC1D2	-0.318927445	0
KRT13	-0.318863389	0
WNT7B	-0.318205384	0
MARVELD3	-0.318059838	0

RIPK4	-0.317284887	0
HLA-V	-0.316917484	0
KLC3	-0.316917344	0
EPS8L2	-0.316597217	0
TP63	-0.316430078	0
CLDN1	-0.315519987	0
DSP	-0.315354901	0
LAD1	-0.313707741	0
PRSS8	-0.313680195	0
ZBED2	-0.313244014	0
PTGES	-0.313108611	0
DMKN	-0.312770908	0
GPC1	-0.312473783	0
EPCAM	-0.312229851	0
CTNND1	-0.311784456	0
DSG2	-0.311616736	0
PROSER2	-0.311148258	0
ESRP2	-0.310442829	0
KIAA1671	-0.309165934	0
SPINT2	-0.309123455	0
KRT80	-0.30877586	0
PPP1R14C	-0.308761294	0
AP1M2	-0.308636458	0
SLC47A2	-0.307729789	0
CLDN4	-0.307198232	0
CXCL16	-0.306937354	0
TPD52L1	-0.306639879	0
CNKSR1	-0.306484486	0
SAP30	0.305647866936794	0
C19orf21	-0.304941744	0
RAB25	-0.304642918	0
ELMO3	-0.304421651	0
KRT19	-0.304402506	0
SPRR1B	-0.304238337	0
ITGA2	-0.303975047	0
PERP	-0.301019757	0

ANXA6	0.300477811136535	0
CHMP4C	-0.300326159	0
STX2	0.299768607554058	0
FXYD3	-0.299532836	0
FAM83A	-0.29860944	0
PPP1R13L	-0.297258914	0
ELF3	-0.297253627	0
PLEK2	-0.297210669	0
DSG3	-0.296804828	0
LYPD3	-0.296790043	0
ZNF165	-0.295901035	0
ZEB1	0.295285531084984	0
TSKU	-0.295140087	0
SOX15	-0.295031892	0
GALNT3	-0.295030973	0
CELSR1	-0.294976311	0
ABCC3	-0.29331959	0
C1orf172	-0.292771793	0
CORO2A	-0.291993177	0
FERMT1	-0.290717128	0
KLK10	-0.290636264	0
S100A9	-0.289333718	0
SERINC2	-0.288586508	2.22044604925031e-16
USP43	-0.288368566	2.22044604925031e-16
RHOV	-0.288261934	2.22044604925031e-16
CTSL2	-0.288155063	2.22044604925031e-16
NDFIP2	-0.2880112	2.22044604925031e-16
SDC1	-0.287845679	2.22044604925031e-16
EFNA1	-0.287529451	2.22044604925031e-16
STAP2	-0.286275743	4.44089209850063e-16
DHRS3	-0.28588911	4.44089209850063e-16
PI3	-0.285752935	4.44089209850063e-16
ARRDC1	-0.285738752	4.44089209850063e-16
KRT14	-0.284406534	4.44089209850063e-16
SERPINB13	-0.283340655	6.66133814775094e-16
CAV2	-0.283079776	6.66133814775094e-16

MUC16	-0.283006379	6.66133814775094e-16
GBP6	-0.282733559	8.88178419700125e-16
HS3ST1	-0.282573521	8.88178419700125e-16
SUGP2	0.282405312403043	8.88178419700125e-16
TGFA	-0.282385706	8.88178419700125e-16
C16orf74	-0.282010124	8.88178419700125e-16
EPPK1	-0.281786982	8.88178419700125e-16
PEX13	-0.281671508	8.88178419700125e-16
DSC2	-0.281670773	8.88178419700125e-16
LIX1L	0.281609640568082	8.88178419700125e-16
KRT4	-0.281430808	1.11022302462516e-15
LTBR	-0.280874786	1.33226762955019e-15
KRT5	-0.280783744	1.33226762955019e-15
PRSS16	-0.280613242	1.33226762955019e-15
WNT7A	-0.2804442	1.33226762955019e-15
PTHLH	-0.280370855	1.33226762955019e-15
PAK6	-0.280326788	1.33226762955019e-15
FOS	-0.280325496	1.33226762955019e-15
KLK7	-0.279915325	1.55431223447522e-15
FAT2	-0.279435625	1.77635683940025e-15
SLC52A1	-0.279051489	1.77635683940025e-15
ESRP1	-0.278800756	1.99840144432528e-15
KCNK1	-0.278378116	2.22044604925031e-15
RXRA	-0.278105275	2.22044604925031e-15
PLEKHO1	0.277852623740614	2.44249065417534e-15
OVOL2	-0.277731598	2.66453525910038e-15
P2RY2	-0.277682416	2.66453525910038e-15
ALDH3A1	-0.277659119	2.66453525910038e-15
KLK5	-0.277507008	2.66453525910038e-15
GRHL2	-0.277441688	2.66453525910038e-15
KRT8	-0.277241541	2.88657986402541e-15
ITPKC	-0.27530695	4.44089209850063e-15
FOXQ1	-0.27506313	4.88498130835069e-15
RNASE7	-0.27415202	5.99520433297585e-15
AKR1C2	-0.273934526	6.21724893790088e-15
SDC4	-0.273856216	6.43929354282591e-15

GPR110	-0.273605948	6.66133814775094e-15
TNFAIP2	-0.272827065	8.21565038222616e-15
DSC3	-0.272634944	8.43769498715119e-15
AFAP1L2	-0.272439281	8.88178419700125e-15
TMEM30B	-0.271973681	9.99200722162641e-15
BAIAP2L1	-0.271555507	1.11022302462516e-14
EPHA1	-0.270736911	1.33226762955019e-14
LLGL2	-0.26973849	1.68753899743024e-14
S100A16	-0.269605768	1.73194791841524e-14
MYOF	-0.269215055	1.88737914186277e-14
PRSS22	-0.268893929	2.04281036531029e-14
C10orf54	-0.267823945	2.59792187762287e-14
ALDH3B1	-0.267012633	3.10862446895044e-14
TMPRSS4	-0.266752685	3.30846461338297e-14
ZFYVE21	-0.266600849	3.41948691584548e-14
KCTD11	-0.266140104	3.81916720471054e-14
RHBDL2	-0.266080245	3.86357612569554e-14
SAV1	-0.266014774	3.90798504668055e-14
PACSIN3	-0.265935472	3.99680288865056e-14
AIM1L	-0.265880375	4.04121180963557e-14
GDA	-0.265840153	4.08562073062058e-14
STK3	-0.265498401	4.39648317751562e-14
FOXE1	-0.265210002	4.70734562441066e-14
WWC1	-0.265004776	4.9293902293357e-14
RASAL2	-0.264118215	6.01740879346835e-14
LRRC1	-0.262987045	7.74935671188359e-14
KIAA1609	-0.262963203	7.7937656328686e-14
ALDH1A3	-0.262784183	8.12683254025615e-14
PLXNB2	-0.262432148	8.79296635503124e-14
SRM	0.262350726349393	8.92619311798626e-14
SH3YL1	-0.262319124	9.01501095995627e-14
GSDMC	-0.262112141	9.41469124882133e-14
AHR	-0.262020489	9.63673585374636e-14
MAP7	-0.261885838	9.9031893796564e-14
PTPRK	-0.261792738	1.01252339845814e-13
AMIGO2	-0.26163335	1.04805053524615e-13

KRTCAP3	-0.261332269	1.12354570092066e-13
TAX1BP3	-0.261215892	1.15019105351166e-13
ARL14	-0.261051881	1.19459997449667e-13
CTSH	-0.260943059	1.22346577313692e-13
CD9	-0.260812977	1.25899290992493e-13
TUFT1	-0.26080888	1.26121335597418e-13
KCTD1	-0.260468026	1.35891298214119e-13
SOWAHC	-0.260317772	1.40554234917545e-13
NIPAL1	-0.259898865	1.54098955817972e-13
PLEKHG3	-0.259682967	1.61648472385423e-13
ANXA4	-0.259670716	1.62092561595273e-13
RASSF7	-0.259466661	1.69642078162724e-13
PVRL4	-0.25912614	1.82964754458226e-13
VGLL1	-0.258964545	1.89626092605977e-13
CCDC64B	-0.258803463	1.96287430753728e-13
DIRC2	-0.257827807	2.43360886997834e-13
NTN4	-0.257789242	2.4535928844216e-13
S100A7	-0.257530931	2.59570143157362e-13
IVL	-0.257338063	2.70894418008538e-13
TNS4	-0.257303599	2.72670774847938e-13
PDLIM1	-0.256759949	3.07309733216243e-13
ID1	-0.256606201	3.1774582964772e-13
ZNF488	-0.255862852	3.73479025483903e-13
BCOR	0.255699503237957	3.8702374638433e-13
EPB41L1	-0.255638986	3.92130772297605e-13
HSPA1B	-0.255548213	3.99902333469981e-13
PTPN3	-0.255188851	4.32320845789036e-13
TMBIM1	-0.254730194	4.77395900588817e-13
TNFSF10	-0.254671151	4.83613149526718e-13
GIPC1	-0.254603482	4.90718576884319e-13
BCL9L	-0.253930296	5.6754601018838e-13
ELOVL7	-0.253813791	5.81756864903582e-13
TNFRSF21	-0.253731463	5.92415005939984e-13
MMP7	-0.253612605	6.07514039074886e-13
MMP13	-0.253331548	6.45483666517066e-13
PCDH1	-0.253179871	6.67021993194794e-13

CCDC28B	0.252649866811995	7.4718009557273e-13
SLC35B4	0.252485467931843	7.74047492768659e-13
MYO5B	-0.252170109	8.28004331765442e-13
ANXA1	-0.251867612	8.83293438391775e-13
HERPUD2	-0.251453193	9.65005853004186e-13
CTDSPL	-0.251392106	9.77662395484913e-13
PRR15	-0.25110374	1.039612840259e-12
CTTN	-0.250981508	1.06692432666478e-12
STARD9	0.250895821809745	1.0866862965031e-12
MCU	-0.250888328	1.0884626533425e-12
TNFRSF12A	-0.250862849	1.09423581307055e-12
RBM10	0.250858015581097	1.09534603609518e-12
WDR7	0.250660705134587	1.14219744773436e-12
MARVELD2	-0.250539354	1.17195142479432e-12
PLEKHA1	-0.250358691	1.2179146580138e-12
PRRG2	-0.250255697	1.24478205520973e-12
FAM216A	0.250244246327119	1.24789067967868e-12
AP1S2	0.250012131218274	1.31072930287246e-12
RBM47	-0.249986702	1.31783473023006e-12
YAP1	-0.249948052	1.32871491587139e-12
SLC44A3	-0.249913375	1.33848487848809e-12
GPR115	-0.24952421	1.45350398383925e-12
AP1M1	0.249481695126465	1.46638257092491e-12
NREP	0.249337882952801	1.51167967032961e-12
EID2B	0.249264302305287	1.53543844305659e-12
BLCAP	-0.249100577	1.58939528205337e-12
ST14	-0.24894735	1.64157576421076e-12
TMOD3	-0.248896333	1.65933933260476e-12
ST6GALNAC2	-0.248812711	1.68887126505979e-12
PPAP2C	-0.248796106	1.69464442478784e-12
SLC2A1	-0.248760867	1.70752301187349e-12
SHROOM3	-0.248629599	1.75526260193237e-12
ANO1	-0.248051307	1.98241423277068e-12
AHNAK2	-0.247305541	2.3179236308124e-12
ITGA6	-0.246990226	2.4757973449141e-12
ZNF185	-0.246942065	2.50111042987555e-12

MICAL1	0.246931692300725	2.50643950039375e-12
MMP28	-0.246505311	2.73980838016996e-12
S100A11	-0.24613652	2.95874436062604e-12
MICALL1	-0.246013127	3.03579383853503e-12
EDN1	-0.245999619	3.04423153352218e-12
EGR1	-0.24597098	3.06243919112603e-12
ZBTB4	-0.245920173	3.09485770344509e-12
PKP2	-0.245861814	3.13282733088727e-12
RIN2	-0.245784193	3.1836755454151e-12
LRRC8E	-0.245585701	3.31779048678982e-12
B3GNT3	-0.245415757	3.43725048423948e-12
VAMP8	-0.244535538	4.12470058108738e-12
CHMP3	-0.244017752	4.5905501622201e-12
PCNT	0.243973493157195	4.63251659255093e-12
BTC	-0.243886258	4.71667149781752e-12
GJB6	-0.243780727	4.8203663283175e-12
CLIP4	-0.243724353	4.87654361336354e-12
C1QTNF6	-0.243453027	5.15676390477893e-12
TLR3	-0.243402409	5.21094278838063e-12
SLC9B2	0.243218227378192	5.41211520044271e-12
FTH1	-0.243096299	5.54933876628638e-12
SEMA4B	-0.242942663	5.72741853943626e-12
JUNB	-0.242842068	5.84687853688592e-12
PSEN1	-0.242801424	5.89572834996943e-12
AVPI1	-0.242417178	6.37911945489122e-12
MAPK13	-0.242392721	6.41109387800043e-12
MROH6	-0.242391485	6.4126481902349e-12
PLA2G16	-0.242377449	6.43129993704861e-12
C11orf80	-0.242171099	6.7088556932049e-12
C9orf40	0.24205841775497	6.86517509507212e-12
FOSL2	-0.242039044	6.8924865814779e-12
PTGFRN	-0.242026331	6.91047219447682e-12
DUOX1	-0.24198585	6.96775970254748e-12
IGFBP3	-0.241728675	7.34390326329049e-12
TMEM125	-0.241153831	8.25739476795206e-12
TMEM159	-0.240989338	8.53850323778715e-12

AKR1B10P1	-0.240929628	8.64286420210192e-12
SHANK2	-0.240920224	8.6595175474713e-12
CDCP1	-0.240801933	8.87023787754515e-12
IFFO1	0.240490526394673	9.44977429639948e-12
DYNC1LI1	0.240371520810835	9.68114477473137e-12
EHF	-0.240054026	1.0325074129014e-11
SPTLC1	-0.240030992	1.03734798528876e-11
DOT1L	0.240005047242287	1.04281028256992e-11
PHLDB2	-0.239834699	1.07940323346156e-11
GJB2	-0.239828322	1.08080211447259e-11
AKR1B10	-0.239794826	1.08815179089561e-11
SCNN1A	-0.239780789	1.09126041536456e-11
LPIN3	-0.239624098	1.12643228078468e-11
HSBP1L1	-0.239499142	1.15525367050395e-11
GPX2	-0.239493806	1.15649712029153e-11
STX19	-0.239376345	1.18427490036765e-11
SIK1	-0.23935659	1.18900445045256e-11
LINC00341	0.239221294861062	1.22195586982343e-11
PROM2	-0.239208272	1.22517551659485e-11
G6PD	-0.239146418	1.24056320771615e-11
KRT7	-0.239059344	1.26256782806422e-11
FRK	-0.239011567	1.27480248579559e-11
ATP8B2	0.238874829147377	1.31044064488606e-11
NFE2L2	-0.238832534	1.32165389743477e-11
MET	-0.238156639	1.51425538774674e-11
FANK1	-0.23810491	1.53006496361741e-11
AMOTL2	-0.238007352	1.56035184772918e-11
IER3	-0.237951944	1.57780455367629e-11
ALPP	-0.237733228	1.64859237372639e-11
ANXA2	-0.237561793	1.70625735762542e-11
HES1	-0.237539768	1.71380687419287e-11
TSPAN6	-0.237479328	1.73467906705582e-11
TUBA1A	0.237449045643422	1.74522618578976e-11
MCM3AP	0.237250148115155	1.81614723260282e-11
TSPAN15	-0.237240518	1.81965553736063e-11
MYL12B	-0.23700063	1.90909510422443e-11

HCFC2	0.236868422996075	1.96020977227818e-11
AKR1C1	-0.236794775	1.98925320660237e-11
ZNF775	0.236490747138382	2.11368700320236e-11
ITCH	-0.23629605	2.19735341033811e-11
SOBP	0.236092670254915	2.28816965375245e-11
ARHGEF16	-0.235813186	2.41902053943477e-11
TMX4	0.235741018053504	2.45397036024997e-11
FGD6	-0.235566915	2.54034571156581e-11
DDX39A	0.235447633405797	2.60123034223625e-11
FAM160A1	-0.235311861	2.67226241135177e-11
SACS	0.235030715576925	2.82542877982905e-11
SLC43A1	0.234978983622345	2.85453882753472e-11
DCP1B	0.234589999740997	3.0829561126211e-11
GCLC	-0.234455287	3.16613402162602e-11
SPRR3	-0.234438798	3.17648130021553e-11
RNF149	-0.234146057	3.36548566792771e-11
TGIF1	-0.23411339	3.38724603921037e-11
SMCR7	-0.233714256	3.66449093291976e-11
HAS3	-0.233661494	3.70277142280884e-11
EIF2B3	0.233454476913401	3.85673715186385e-11
ATP6V1B2	0.233422343907481	3.8811842628661e-11
CEP170	0.233365907531676	3.92450516528697e-11
DNAJB1	-0.2333253	3.95594668134436e-11
CYP24A1	-0.233242616	4.02076150152197e-11
LYRM7	0.233096952635142	4.13746814587057e-11
TERF2IP	0.23297905233677	4.23434620699936e-11
ZNF428	0.232709360994489	4.4643622132412e-11
LPIN1	0.232599801405459	4.56124027436999e-11
AP1S3	-0.232060449	5.06896746799157e-11
TSHZ1	0.231945975700395	5.18363130197486e-11
HOOK2	-0.231907554	5.22266674352068e-11
GRHL1	-0.231880611	5.25020027453138e-11
PMEPA1	-0.231751786	5.38391553561723e-11
MYO1B	-0.231743417	5.39270850197227e-11
MINK1	-0.231711377	5.42654809976284e-11
ITPRIPL2	-0.231433259	5.72897285167073e-11

MAPRE2	0.230987551005967	6.24824636474841e-11
FAM129B	-0.230692492	6.61697363568692e-11
LRRC8A	-0.230634811	6.69149180509976e-11
PDZK1IP1	-0.230628427	6.69979627332395e-11
THBS1	-0.230449705	6.93625157310862e-11
ZDHHC12	-0.230310079	7.12663261737134e-11
PBRM1	0.230292803526481	7.15054682132177e-11
PLEKHG6	-0.229903812	7.71003261235137e-11
S100P	-0.229802087	7.86326559421013e-11
POLR3GL	0.229732858339141	7.96922527968036e-11
C4orf46	0.229661108967592	8.08051403566878e-11
GALNT12	-0.229649728	8.09832201298377e-11
HIBADH	-0.229633946	8.12305778197242e-11
MPZL3	-0.229318092	8.63393800898393e-11
S100A10	-0.229314828	8.6393558973441e-11
FUT3	-0.229183388	8.8612228665852e-11
PPIP5K2	0.229118658345482	8.97251162257362e-11
TMEM184A	-0.22903603	9.11657416224898e-11
MUC4	-0.228940204	9.28643828501663e-11
MICALL2	-0.228816875	9.50963752188727e-11
FZD6	-0.228769332	9.59705648284626e-11
PHLDA3	-0.228725626	9.67812496810438e-11
RBMS2	-0.228662485	9.79643033360844e-11
ZSCAN31	-0.228626728	9.86402071134762e-11
ADORA2B	-0.228623232	9.87068204949537e-11
TCF4	0.22854225215179	1.00255803658911e-10
SMAP2	0.228479827206751	1.01466390844962e-10
PXK	0.228353229093737	1.03964836739578e-10
CYP4F3	-0.228255974	1.05925046511857e-10
EPHA2	-0.228048274	1.10232711847402e-10
CSTA	-0.228010967	1.1102430086396e-10
MON1A	0.227543745202908	1.21420651311155e-10
TNFAIP1	-0.227168616	1.30449873125826e-10
MAP3K3	0.22713872637809	1.31196831176794e-10
LRG1	-0.226951725	1.35965461112164e-10
LSS	0.226929371604111	1.36546773887858e-10

TJP2	-0.226508626	1.47950540707598e-10
GRB7	-0.226359237	1.52218682103467e-10
HN1L	-0.226285048	1.54382728823066e-10
B3GALT4	-0.226240457	1.55697899018037e-10
CLPP	0.226142112508385	1.5863710345343e-10
CYP1A1	-0.226126544	1.59107393926661e-10
ADRB2	-0.226100658	1.59892099560466e-10
ASNSD1	0.225929567348025	1.65175428890052e-10
SLC35F5	-0.225869068	1.67084124313988e-10
BAIAP2	-0.225571135	1.76801684403927e-10
PRSS23	-0.22554564	1.77658332489727e-10
ST3GAL2	0.22487281119786	2.01788585840745e-10
LRRC16A	-0.224776437	2.05496730742993e-10
FLII	-0.224757607	2.06229033850036e-10
C1orf216	0.224686621931057	2.09012362972771e-10
SLC29A3	-0.224610158	2.12051265435775e-10
ABCA12	-0.224563562	2.13924433722923e-10
RNF217	-0.22449789	2.16591633517282e-10
MPP5	-0.224426775	2.19516405053355e-10
SEPT6	0.224386346708569	2.21196394534218e-10
TTC37	0.224371905477391	2.21799467681194e-10
STYK1	-0.224361576	2.22232010571588e-10
SLC16A5	-0.224281548	2.25609753101708e-10
TNK1	-0.224242463	2.272775301293e-10
C15orf48	-0.224053559	2.35509389767685e-10
CSTB	-0.223969237	2.39277042624053e-10
EFEMP1	-0.223767081	2.48550513504142e-10
ETHE1	-0.223608678	2.56060728176521e-10
CBLC	-0.223477726	2.62436294917734e-10
PRKCZ	-0.223434679	2.6456548063436e-10
SLC20A2	-0.223094223	2.8201019297569e-10
JAG1	-0.222852253	2.95084845447491e-10
COQ10A	0.222693060460009	3.04007041762588e-10
HDHD2	0.222635091722495	3.0732083544649e-10
SOX12	0.222571111530651	3.11019210386121e-10
MTFR1L	0.222478886543701	3.1642666264986e-10

TMEM139	-0.222447268	3.18301385249242e-10
PHF21A	0.222382138702013	3.22197601931862e-10
ZNF750	-0.22230256	3.27021298929253e-10
RAB38	-0.222223159	3.31904281836159e-10
UEVLD	-0.222216988	3.3228664264584e-10
PLK2	-0.222144284	3.36825234370508e-10
KRTDAP	-0.222141052	3.37028405184014e-10
KLHL15	0.222123866036892	3.38110872633024e-10
CAST	-0.221744276	3.62896823702386e-10
ITGA3	-0.221706297	3.65472097030306e-10
RIOK2	0.221566684682011	3.75092623627893e-10
FKBP7	0.221515867929302	3.78655329313915e-10
PTPRF	-0.221489286	3.80532050314741e-10
ZNF333	0.221451953950159	3.83182818808336e-10
PYGL	-0.221317275	3.92897048229202e-10
TLCD1	-0.221105053	4.08692857334358e-10
RAB20	-0.221006842	4.16210621523305e-10
DLK2	-0.220941479	4.21288337548731e-10
TEAD3	-0.220910561	4.23711288277673e-10
TPBG	-0.22079214	4.33117541831507e-10
RAB39B	0.22070511743946	4.4015902034289e-10
GOLGA5	-0.220540588	4.53778348230571e-10
CHP1	-0.220488841	4.58145965609447e-10
LEPRE1	0.220409328429538	4.64936977806474e-10
PQBP1	0.220364773445822	4.6878523285443e-10
LITAF	-0.22025349	4.78532768966033e-10
S100PBP	0.219902703298733	5.10568476386197e-10
CCDC88A	0.219635638005731	5.36349631374833e-10
SH2D4A	-0.219624119	5.37489608376518e-10
SPR	-0.219524111	5.47487166713267e-10
SPAG1	-0.219292303	5.71362068768622e-10
GRTP1	-0.219274783	5.73207259435549e-10
CCND1	-0.219106681	5.91208415556821e-10
PLAU	-0.219041473	5.98338267820964e-10
CARD10	-0.219009977	6.01811933620411e-10
PRR3	0.218927796523564	6.10968609038309e-10

GCLM	-0.218803336	6.2509464271443e-10
FBXO27	-0.21865006	6.42929265382008e-10
CHMP4B	-0.218643828	6.4366467711352e-10
ASAP2	-0.218587725	6.50322906636802e-10
ATXN7L2	0.218572947633095	6.52087717156746e-10
PAIP2	0.218414085252168	6.71358302284375e-10
TJP1	-0.218370764	6.76708911129253e-10
EPS15L1	0.218334038868382	6.81277256830981e-10
MGST2	-0.218281572	6.87855772341095e-10
PAQR7	-0.218181222	7.00611124671013e-10
ACE2	-0.21813486	7.06581460008238e-10
C20orf24	-0.218114691	7.0919425887439e-10
AGPAT2	-0.217743191	7.59036833386517e-10
CNPY4	0.217689983335926	7.66449126388125e-10
WNT10A	-0.217598602	7.79343700685331e-10
MTMR9	0.217476449693005	7.9691098164858e-10
ZEB2	0.217475366289508	7.97068633318077e-10
FOXC1	-0.217461835	7.99038613052971e-10
RHPN2	-0.217435827	8.02838240332449e-10
KIAA0922	0.217430277765027	8.03651145631079e-10
USP40	-0.217352517	8.15127965125839e-10
PDXK	-0.217306375	8.22013568324564e-10
SV2A	0.217278989630145	8.26126944630801e-10
EMP3	0.217115330347391	8.51129389189964e-10
EVPL	-0.21705466	8.60584270512277e-10
EPN3	-0.217023238	8.65520988213575e-10
DTX2	-0.216964654	8.74798899985763e-10
SMPDL3B	-0.216869849	8.90018725385744e-10
ZDHHC7	-0.216795552	9.02126373603096e-10
IL1A	-0.216590747	9.36337229973105e-10
BEX1	0.216454266460357	9.59832657798643e-10
CNRIP1	0.216453283372534	9.60004076233645e-10
ARHGEF28	-0.216357488	9.76839942268271e-10
SPRR1A	-0.216254846	9.95198146114262e-10
ZCCHC18	0.216198433258849	1.00543062764302e-09
TMSB15B	0.216132540249917	1.0175122966416e-09

CDC42BPB	-0.21603618	1.03543484897273e-09
C5orf38	-0.216024386	1.03764952186225e-09
F2RL1	-0.216022282	1.03804498330362e-09
SLC44A2	-0.215943484	1.05296571462077e-09
WDR66	-0.215926391	1.05622977031317e-09
CLIC3	-0.215897607	1.06174846692397e-09
GPATCH11	0.215883773912132	1.06441078173702e-09
WDR48	0.215786703478997	1.08327569137145e-09
RAC1	-0.215735401	1.0933769445387e-09
EIF1B	0.21570139663821	1.10012265963633e-09
PAICS	0.215655393473637	1.10931352992338e-09
RASEF	-0.215372572	1.16747989054033e-09
WIPF1	0.215372015124751	1.16759757418095e-09
DHX30	0.215352876280538	1.171639674169e-09
ME1	-0.215190902	1.20639764844555e-09
VIM	0.215145699158484	1.21627530269564e-09
DPY19L3	0.215048014912291	1.23789090089588e-09
RAET1L	-0.215003675	1.24782539856483e-09
DLG5	-0.214964774	1.25660504224356e-09
PLXNB1	-0.214804103	1.29350841149289e-09
LGALS3	-0.214798213	1.29488109124054e-09
ZFP36L1	-0.214773172	1.30073285475873e-09
PPP1R26	-0.214651788	1.32946498254682e-09
ANK3	-0.214580799	1.34655353534185e-09
B4GALT4	-0.214549853	1.35406974521857e-09
A1BG	0.214486685318568	1.36953870466527e-09
MATR3	0.214434567141446	1.38243105851643e-09
OSBPL2	-0.214427341	1.38422806550409e-09
PPP4R1	-0.214365422	1.39971767509905e-09
NDUFB11	0.214250595654748	1.42889056142792e-09
PTGR1	-0.214183729	1.44615031061335e-09
POGZ	0.2141164303342	1.46372625131619e-09
NDUFAF4	0.214004884521799	1.49331658150231e-09
AHNAK	-0.21381126	1.54606771829435e-09
CEP170B	-0.213799106	1.54943902153093e-09
SLC25A20	0.213748191855812	1.56363832992668e-09

C9orf69	-0.21369304	1.57916257848001e-09
TSPAN2	-0.213663339	1.58758428625561e-09
APLP2	-0.213644922	1.59282875777933e-09
CHST10	0.213372800534374	1.67230940206764e-09
HSF2	0.213283994734121	1.69907243829925e-09
ZDHHC5	-0.213262895	1.70549219191685e-09
USP13	0.213220912256675	1.71833614004413e-09
TMEM144	-0.213171687	1.73351555332601e-09
FADD	-0.213036713	1.7758088333153e-09
VANGL1	-0.213016614	1.78219172752847e-09
AKR1C3	-0.212973853	1.79584658255294e-09
ATP8B1	-0.212964953	1.79870118799386e-09
OVOL1	-0.212890665	1.82270243342941e-09
PLLP	-0.212847745	1.83671078346492e-09
TC2N	-0.212838725	1.83966841760252e-09
PPP3CC	0.212733568824525	1.87448789823463e-09
CGGBP1	0.212709792855204	1.88244886345501e-09
ANKS6	-0.212662448	1.89840010378362e-09
ATP1B1	-0.212628375	1.90996063409443e-09
EBF1	0.212516527879116	1.94839344658249e-09
TRADD	-0.212254704	2.04132444281413e-09
MRPS25	0.212139587792726	2.08353445607656e-09
CEBPD	-0.212118422	2.09138706352974e-09
HES4	-0.212062631	2.11222461743432e-09
TMPRSS11E	-0.212040579	2.12051620707143e-09
LPXN	0.211902165768636	2.17328777196713e-09
PPDPF	-0.21182113	2.20477369694549e-09
SH2B3	0.211789969236777	2.21699902880346e-09
LAMP1	-0.211781287	2.22041673936246e-09
ZNF204P	-0.211776123	2.22245244430042e-09
NDUFS7	0.211750117548739	2.23272955679477e-09
DTX4	-0.211697446	2.25368745887522e-09
EPS8L1	-0.211637676	2.27770158289786e-09
RPGR	0.211250482261452	2.4394004594086e-09
CMTM4	-0.211187492	2.46674058956842e-09
UBE2M	0.210985239896058	2.55655430159152e-09

AGPAT6	0.210910337821969	2.59061594398702e-09
IP6K1	0.210767414280628	2.65683852695986e-09
PVRL1	-0.210445346	2.81215428721282e-09
AMPD2	0.210371998999303	2.84874013267711e-09
PPIC	-0.210366356	2.85157408796977e-09
TGM1	-0.210266521	2.90216517484509e-09
GRHL3	-0.210224198	2.92387491995783e-09
ERMP1	-0.210116885	2.9796300982099e-09
PLS1	-0.209941659	3.07289904633024e-09
S100A8	-0.209758269	3.17355119960894e-09
TMEM54	-0.209704791	3.20350057592123e-09
B3GNT5	-0.209606735	3.25912941079309e-09
CD46	-0.209539074	3.29806137955302e-09
SOWAHB	-0.209508591	3.31574878664753e-09
TOP2B	0.209497615591033	3.32213923037727e-09
CAV1	-0.209326446	3.42337047598562e-09
TMX3	0.209297184486422	3.44097328408566e-09
RAB33A	0.209289477386461	3.44562445242502e-09
RND3	-0.20922516	3.48467699140542e-09
RASA3	0.209223355915617	3.48577833264585e-09
SCFD2	0.20906470721598	3.58398510869051e-09
SH3D19	-0.208959677	3.65047392314466e-09
LIPH	-0.208951431	3.65574504002097e-09
ZFP82	0.208915595607897	3.67873553841491e-09
EP400NL	0.208879545978384	3.70200536892185e-09
KIF1C	-0.208855609	3.71753561267951e-09
ZBTB17	0.208799891034297	3.75393049978356e-09
SLC7A5	-0.208770517	3.77325681810703e-09
DTNBP1	0.208758687460454	3.78106723708527e-09
LRP11	-0.208691613	3.82565024104053e-09
DAB2IP	-0.208523975	3.93932153563981e-09
CYR61	-0.20836204	4.05223721244852e-09
ADK	-0.208276491	4.11315270731905e-09
DOK7	-0.208114452	4.23098045487791e-09
RCE1	-0.208055419	4.27471635866539e-09
JAM3	0.207980227116528	4.33106039920972e-09

FHL2	-0.207826489	4.44851533387691e-09
ARHGEF2	0.207798914199404	4.46990711111539e-09
CALB1	-0.207797938	4.47066628161963e-09
CITED4	-0.207549133	4.66828686818133e-09
PTAFR	-0.207509511	4.7005306313963e-09
CD320	0.207431240735858	4.76486361478123e-09
SLC3A2	-0.207413522	4.77954520405888e-09
GNAZ	0.207364165758479	4.82067430418454e-09
PLEC	-0.207234241	4.93059149064834e-09
CCT8	0.207198613665796	4.96115504233785e-09
GRAMD1A	0.207180543559872	4.97672725252585e-09
TNKS	0.207100389013139	5.04637531761887e-09
PRKCI	-0.207006584	5.12908737704265e-09
PTBP3	-0.20684039	5.27887134005312e-09
WFDC2	-0.2068135	5.30350208194363e-09
RNF207	-0.206809214	5.30743804461054e-09
ERO1L	-0.2067952	5.32032773392643e-09
DGKA	-0.206776494	5.33758104381832e-09
UBE2G2	0.206718252136972	5.39164801693914e-09
SLC31A2	-0.206716732	5.39306599378619e-09
CHN1	0.206474667317708	5.62359137035173e-09
TMEM141	-0.206411891	5.68492053432124e-09
FA2H	-0.206393969	5.70254798937242e-09
HTATIP2	-0.206085296	6.01461058735708e-09
CAPN2	-0.206001426	6.10222761210366e-09
MYO6	-0.205972437	6.13279826922053e-09
PPCS	-0.205912474	6.19650597499799e-09
LONP1	0.2058742844408	6.23741502892017e-09
DUOXA1	-0.205854673	6.2585252535996e-09
RAB10	-0.205633873	6.50103171295768e-09
ZNF708	0.205550945757814	6.5944485427849e-09
BLVRB	-0.205355005	6.82037937238533e-09
GDE1	-0.205339479	6.83859924244246e-09
FAM108C1	-0.205339253	6.83886414165613e-09
NPC2	-0.205329326	6.85053902493848e-09
CREG1	-0.205028904	7.21315052132354e-09

ELAVL1	0.204980052210189	7.27384952270427e-09
PXN	-0.204907601	7.36478411589303e-09
THRB	-0.204868515	7.41429895256829e-09
CASP4	-0.204839751	7.4509445280313e-09
FYN	0.204817033201024	7.4800108329498e-09
SYT11	0.204765885183233	7.54585571804967e-09
TNKS1BP1	-0.204746443	7.57103224557909e-09
ARHGAP23	-0.204734932	7.58597584749054e-09
HPSE	-0.204602778	7.75960895538219e-09
KLK6	-0.204499363	7.89816745339067e-09
SQRDL	-0.204485747	7.91658805177065e-09
IGFL1	-0.204392399	8.04400612786083e-09
MIR22HG	-0.204369821	8.07512234857199e-09
RBPJ	0.204368289280637	8.0772371013893e-09
HOOK1	-0.204282637	8.19637513416183e-09
AQP3	-0.204259701	8.22856560667162e-09
PPFIA1	-0.204030796	8.55663540022533e-09
AP4B1	0.203885799649353	8.77097372509184e-09
PPP2R3C	0.203864722447286	8.80256134649926e-09
IL20RB	-0.203797617	8.90386564478263e-09
R3HCC1	0.203796108370206	8.90615692306085e-09
MST1R	-0.203777	8.93521745481962e-09
TM4SF1	-0.203635986	9.15253783873027e-09
PAWR	-0.203542339	9.2996828016112e-09
MAP3K9	-0.203534552	9.31202093212846e-09
FKBP5	0.203519806400834	9.33542931846887e-09
ANKRD2	-0.203519151	9.33647070766597e-09
CYB561	-0.20347029	9.41445232882643e-09
CLASP2	0.203349821854934	9.60942614369742e-09
TES	-0.203285953	9.71437907892891e-09
MKRN2	0.20317967439397	9.89149206986895e-09
RBKS	-0.203172223	9.90402715395078e-09
ZNF407	0.203150301093078	9.94099247364488e-09
EZH2	0.203137183230911	9.96317650603373e-09
APPL1	0.203082094588392	1.0056864230279e-08
BLMH	0.203066606746207	1.00833574823156e-08

TNRC6C	0.20306560067135	1.00850807704944e-08
KLF4	-0.203038851	1.01310062561311e-08
TXNDC15	0.202887202876986	1.03952202401558e-08
RNF141	-0.202672813	1.07801827464726e-08
DENND4B	0.202667957792415	1.07890592015991e-08
ZNF546	0.202667176899739	1.07904876145426e-08
GMEB1	0.202638846840517	1.08424287326159e-08
PIP4K2C	-0.202593836	1.09254516544866e-08
CEP85L	0.20258187891843	1.0947610595835e-08
PGBD1	0.2025760054582	1.09585109875354e-08
TTC9	-0.202313219	1.14571339082659e-08
ABLIM1	-0.202250848	1.15786717991284e-08
FOXN3	0.202232659783426	1.16143485939801e-08
SNX33	-0.202197211	1.16841896158348e-08
ZNF330	0.202193937315212	1.16906599956224e-08
HRASLS2	-0.202177444	1.17233107665982e-08
DOCK9	-0.202171838	1.17344285399668e-08
SUGP1	0.20215399314999	1.17698881751949e-08
DOCK10	0.202145052716718	1.17876925997962e-08
FGFR3	-0.201987265	1.21062506686087e-08
MYO1E	-0.201953771	1.21749370585178e-08
PARD6B	-0.201933855	1.22159575788316e-08
SERPINB1	-0.201896384	1.22935026602988e-08
TNFRSF1A	-0.201895635	1.22950560843549e-08
SMURF1	-0.201856265	1.23770549365076e-08
CREG2	-0.201743608	1.26146293411011e-08
MID2	-0.2016736	1.27644890213219e-08
CMIP	-0.201668594	1.27752706191586e-08
DCK	0.201579008783202	1.29697128592454e-08
GPR56	-0.201527038	1.30838273548761e-08
ARHGEF6	0.201526060420427	1.30859822977669e-08
BAG3	-0.201470918	1.32081434678355e-08
TATDN2	0.201431219447912	1.32967756805158e-08
SRSF10	0.20141796277731	1.33265007917771e-08
DOCK11	0.201407428118242	1.33501685262161e-08
DLG4	0.201374274122783	1.34249200645087e-08

ZNF275	0.201346133644862	1.34886861680172e-08
LY6K	-0.201230541	1.37537132793852e-08
PLD2	-0.201194117	1.3838264312227e-08
ANKRD22	-0.201162647	1.39117233288744e-08
TMEM55A	0.201128833636881	1.3991073410935e-08
AGR2	-0.200887527	1.45702254705782e-08
FGD4	-0.200810797	1.4759203637027e-08
SHQ1	0.200632014642021	1.52087948901425e-08
EPAS1	-0.200575671	1.5353214255498e-08
SLC39A1	-0.200573245	1.53594625906806e-08
PTPRU	-0.200447376	1.56870394452113e-08
PLS3	-0.200442246	1.57005344281202e-08
FOSB	-0.200430701	1.57309409942741e-08
ARAP1	-0.200380754	1.58631563440537e-08
PKP1	-0.200346107	1.59555004763945e-08
ARNTL2	-0.200170232	1.64323596951732e-08
DNAJC12	0.200072870664537	1.67022524699689e-08
NXN	-0.200005475	1.68915894604993e-08
ADAM9	-0.200001472	1.69029004126742e-08

Table S3 RNA interference sequence,

related to Fig. 2f, 3h and Fig. S3c-j, S6a, S7f, S10b

Gene Symbol	Species	Sequence
MMP-28	Human	GAAGTACGGATACCTCAATGA
		GGGTTACAGATACCAACAGTT
	Mouse	GGCATTCCTGGAGAAGTATGG
		GCTTTGCAAAGCCAGGTAACA
EGFR	Human	GCAGAGGAATTATGATCTTTC
		GCAGATCATCAGAGGAAATAT
	Mouse	GGAAATTACCTATGTGCAAAG
		GCAAAGGAATTACGACCTTTC
TGF-α	Human	AAUAAACUAAAAUCUACUCGG
	Mouse	UCAACUUGUCUUUAAAAUGUG

Table S4 Relative mRNA expression level in transcriptomics, related to Fig. 2a-b and Fig. S5a

Gene Symbol	Relative mRNA expression level					
	Vect_1	Vect_2	Vect_3	28-OE_1	28-OE_2	28-OE_3
MMP28	459.4978593	418.2855314	431.1241303	148512.4929	145742.4097	141638.3812
PTX3	227.2678397	239.5907578	261.8541355	4682.448255	5488.466893	4277.087276
TNFAIP3	1921.35606	2076.453234	1888.155356	10958.80623	10955.15415	11134.69709
IL24	529.960814	519.1133086	419.9018102	5339.507301	5713.140976	5017.352381
STC2	3637.277871	3589.868188	3322.741941	20641.40162	21507.95829	19247.88373
ATF3	402.9290084	335.427061	306.7434159	3802.458462	4533.602039	3725.109145
ERN1	638.1363359	615.9479066	684.5615258	3713.105652	3740.364969	3838.08133
CXCL8	576.6053051	674.8473012	750.0250597	5146.360823	5049.435393	4789.426044
DUSP1	314.602206	303.4816266	276.817229	2403.500328	2614.70196	2303.046995
DUSP8	148.8653972	153.7374029	127.1862944	1868.286023	1937.240821	1634.132743

LURAP1L	57.56128691	72.87552217	81.36182069	1448.598583	1643.788957	1366.567042
PPP1R15A	1539.268207	1399.609344	1083.889082	12107.75701	14016.91169	11500.37021
ARHGEF2	1627.595009	1635.206922	1635.653154	6581.421102	7049.722513	6657.431029
AKAP12	2726.22164	3153.61335	3076.786093	13338.84016	14692.08023	14189.901
DDIT3	411.8609322	325.4441127	303.0026426	3628.26561	4880.929831	3834.117394
GDF15	1200.847537	982.3221071	905.2671543	6927.099144	7852.129953	7026.077105
PCNA	3385.199132	3113.681557	3252.602441	653.4488313	602.9518767	635.2207933
KLF6	1055.951884	1193.96061	1138.130296	5385.537537	5378.422444	5025.280254
BIRC3	301.7005383	323.4475231	403.0683301	2514.514425	2680.040851	2649.891422
CDKN2B	897.162127	909.4465849	890.3040608	4283.519549	4471.702036	3944.116626
SESN2	955.7158499	882.4926246	947.3508547	4220.340794	4579.453892	4149.25033
ERRFI1	7409.527036	8234.934005	6005.811637	43796.41511	45530.89076	43503.20999

MICAL2	983.5040574	1098.124307	968.8603015	4625.587376	4682.620563	5053.027808
ELL2	1967.008115	2175.284422	2245.399212	8993.044413	9969.3393	9089.305953
IL1A	6123.330004	6212.388691	5853.375123	21266.87129	20908.44531	22934.34449
SPRY4	442.6264476	460.213914	389.9756233	2357.470093	2330.420466	2349.623246
MAP1LC3B	1574.003466	1561.333105	1645.005087	8180.746142	10309.78931	7872.377507
HBEGF	3371.305028	3363.255263	2741.051683	14539.2365	16575.44513	14778.54554
KLF5	4175.178173	4388.504047	4534.752512	14047.34477	15355.78582	15188.81295
KRT14	8872.377672	7362.424329	6419.167094	1212.129531	1317.094499	1178.280067
KIF21B	594.4691527	695.8114925	669.5984323	3236.557333	2893.251971	3123.58181
TSC22D3	634.166592	581.0075877	612.5516385	2579.498287	2563.118624	2492.324953
CLK1	856.4722518	919.4295331	886.5632875	3399.016987	3848.116825	3674.568957
CARS	1750.657071	1691.111432	1691.764754	5532.653779	5678.752086	5407.800108

TRIB3	2585.295731	2187.26396	2338.918546	10563.48774	12581.74867	10234.88355
CREB5	25.80333551	27.95225508	24.31502687	695.8688521	772.6037355	744.2290418
SLC9A1	987.4738013	949.3783779	958.5731748	3348.473984	3582.176073	3424.84097
CXCL2	158.789757	150.7425185	157.1124813	1128.192043	1156.613011	1023.686551
GADD45A	1187.94587	1159.020291	908.0727343	6463.186576	7140.279924	5576.267401
RGS3	313.6097701	290.5037939	350.697503	1597.519933	1774.46674	1634.132743
KRT80	338.4206696	286.5106146	277.7524223	1697.703386	1914.314894	1616.295029
ETS1	1547.207695	1748.014237	1599.180613	5263.692796	5364.666888	5435.547662
H2AFX	2923.716401	2742.315882	2598.902295	723.8480148	674.02225	733.3282169
ABL2	1256.423952	1562.3314	1409.336365	5348.532837	5290.157625	5599.060035
TOP1	2976.315508	2988.894704	3116.064213	8575.162081	8959.452221	8439.220399
LIMA1	2833.404726	2980.908345	2871.978751	8016.481381	8447.057755	8507.5983

FOSL2	2944.557556	3157.606529	2947.729412	8709.642572	8921.624442	8512.553221
NFIL3	793.948785	762.6972457	749.0898663	2836.726073	3183.264946	2797.548049
INTS6	659.9699275	748.7211182	764.0529598	2763.619229	2761.427892	2990.789944
KLF4	1578.965646	1588.287065	1406.530785	5452.326505	5975.642839	5200.684436
KIAA0040	716.5387784	745.7262337	762.1825731	2572.277858	2572.288995	2720.251291
VEGFA	6198.755139	6235.349472	5622.382367	18255.95236	17983.09704	17025.10644
IFRD1	734.4026261	741.7330544	793.0439534	2665.240882	3007.881605	2802.50297
RNF19A	630.1968481	659.8728788	675.2095924	2287.973463	2379.711209	2356.560135
SNAI2	1438.039737	1485.462698	1339.196865	4373.774912	4526.724261	4454.473426
CEBPG	1013.277137	1160.018586	1122.232009	3834.047839	4179.396468	3745.919811
TRIML2	86.34193037	66.88575323	67.33392057	795.1497519	1163.490789	895.8496055
GTPBP2	2192.291083	2076.453234	1969.517177	7015.5494	8000.002181	6791.213879

PRDX1	8964.674218	8625.267282	8603.778739	2999.185727	3148.876056	2802.50297
LAMP3	150.8502691	195.6657856	194.520215	1151.658438	1369.824131	1158.460386
HAS3	6387.317975	6629.675928	6059.117658	1794.276625	1434.016726	1750.07788
OASL	4160.291633	3675.721543	3987.664407	1114.653738	1191.001901	1037.560329
CHAC1	197.4947603	161.7237615	203.8721484	1140.827794	1180.685234	1048.461153
PNRC1	381.0954168	388.3366866	431.1241303	1898.070293	2222.66861	1783.771338
PLAU	3940.963281	3690.695965	3716.458338	10408.24851	11289.87269	10341.90983
SLC38A2	11179.79133	13090.64003	13758.56444	43609.58651	41983.10358	40539.17662
PABPC1L	1051.98214	1017.262426	1034.323835	3129.15345	3460.668661	3420.877033
MAP1B	211.388864	246.5788216	327.3176694	1611.058237	1677.031551	1789.717243
ICAM1	1296.121391	1224.907749	1267.186977	3928.815971	3797.679786	3670.605021
INHBE	27.78820747	16.97101201	24.31502687	537.9219661	671.7296573	551.9781308

TRIM25	3335.577333	3327.316649	3355.473708	8677.150641	8469.983682	8390.662179
MINDY2	314.602206	469.1985674	433.9297103	2172.446598	2173.377867	2271.335504
HMGN2	7513.732814	7201.698862	7598.445897	2654.410239	2672.016777	2370.433912
GSK3B	1338.796139	1447.527495	1349.483991	3957.697687	3869.896456	4005.557639
CLDN1	8064.534783	8763.031967	8716.937134	21511.46332	22196.8824	21137.69036
MXD1	1070.838424	1021.255605	1178.34361	3656.244773	4274.539065	3714.20832
PDE4D	345.3677215	451.2292606	444.2168371	2153.492972	1819.172297	2209.894492
HPDL	612.3330004	544.0706792	592.9125783	53.25066443	63.04629889	49.54920385
FEN1	3162.893472	2671.43695	2740.11649	802.370181	680.900028	748.1929781
KDM6B	964.6477737	1025.248785	870.6650007	3226.629243	3036.539014	3042.321116
AMOTL2	2896.920629	2903.041349	2614.800582	7443.359823	7472.705863	7565.172443
NR1D1	437.6642677	412.2957624	367.5309831	1579.46886	1892.535263	1732.240166

NFE2L2	1805.24105	1799.925568	1958.294857	5003.757349	5528.587265	5492.033754
CAMSAP2	1622.632829	2077.451529	2162.167005	7102.194549	7295.02993	7464.092067
FAM129A	1078.777912	1280.81226	1314.881838	4003.727922	4495.774259	4148.259346
HES2	1496.59346	1308.764515	1184.889963	275.2788585	257.9166773	221.9804332
GRPEL2	809.8277607	917.4329435	904.331961	2887.269076	3099.585313	2727.18818
HSPA6	9.924359812	5.989768946	11.22232009	538.8245197	627.0240999	561.8879716
CDKN1A	1833.029257	1745.019353	1515.013213	5852.157765	6455.941007	5375.097633
E2F7	1007.322521	1084.148179	1121.296816	3066.87725	3257.774208	3114.662954
USP53	573.6279971	700.8029666	706.0709726	2305.121982	2379.711209	2437.820829
FAM13B	409.8760602	395.3247504	461.0503172	1578.566307	1568.133398	1758.996737
KANSL1	796.9260929	796.6392698	761.2473798	2285.265802	2351.053801	2441.784766
UNC5B	415.8306761	396.3230452	480.6893774	1697.703386	1758.418591	1565.754842

ARL14EPL	188.5628364	206.6470286	224.4464019	1048.767323	1294.168572	1020.713599
GPC1	6343.650792	6034.692213	5622.382367	2136.344453	1916.607486	1781.78937
RASSF10	1671.262192	1589.28536	1760.033868	388.0980628	257.9166773	352.7903314
QKI	1250.469336	1548.355272	1474.799899	4459.517508	4507.237223	4401.95127
ANKRD11	2993.186919	3175.575836	3232.028187	7865.754924	7516.265124	7835.711096
CTH	508.1272224	506.1354759	555.5048447	1748.24639	1843.24452	1681.699979
MARCKSL1	2470.173157	2131.35945	2159.361425	696.7714058	646.5111377	670.8962201
PHLDB2	1580.950518	1743.022763	1731.042875	4414.389826	4454.507591	4643.751384
HERPUD1	895.1772551	876.5028557	880.9521274	3040.703194	3696.805708	2884.754648
BCAR3	2803.631647	2872.094209	2541.855501	6953.273199	7069.20955	7153.914051
AARS	7929.56349	7337.466958	7173.868121	18914.81652	21601.95459	19331.12639
FAM111B	643.0985158	736.7415803	795.8495334	89.35280981	111.1907453	67.38691723

MAFF	1251.461772	1064.182283	933.3229545	4262.760815	4523.285372	3780.604253
RUNX1	1333.833959	1491.452467	1437.392165	3777.18696	3946.698311	3786.550158
TSLP	112.1452659	123.7885582	100.0656875	723.8480148	982.3759664	744.2290418
EFNB2	424.7626	382.3469177	412.4202635	1414.301545	1361.800056	1373.503931
KDM2A	3124.188469	3223.493988	3158.147913	7289.023151	7150.596591	7405.624007
ATG12	4361.756137	4740.90212	4762.939687	11597.8142	11648.66344	10785.87069
KLF7	1340.781011	1576.307528	1462.642386	4242.002082	3953.576089	4174.024932
ID1	6750.549544	6037.687097	6094.655005	2168.836383	1782.490814	2159.354304
PPP2R5B	349.3374654	396.3230452	339.4751829	1272.600625	1317.094499	1344.765392
GRB10	1039.080472	1051.20445	1005.332842	2781.670301	2692.650111	2720.251291
SIAH2	2957.459224	2589.576774	2539.985115	7005.62131	7266.372522	7200.490303
KLF3	1345.743191	1443.534316	1551.485753	4111.131805	4032.670536	3828.171489

SAMD4A	158.789757	211.6385027	185.1682816	855.6208454	956.0111505	929.5430641
ZBTB10	278.8745107	294.4969732	340.4103762	1259.964874	1144.003751	1209.000574
HK2	2971.353328	3015.848664	2560.559368	7708.710591	7718.013281	7446.254354
RRM2	2895.928193	2980.908345	3068.369353	1111.946078	1023.642635	1143.595625
PAQR4	760.2059616	702.7995563	699.5246192	148.018796	145.5796356	149.6385956
SLC7A11	2250.844805	2801.215277	3197.426034	10568.00051	9383.581868	10016.86705
ELMSAN1	1047.01996	1094.131127	1050.222122	2761.814121	2713.283445	2746.016877
SCEL	767.1530135	797.6375646	804.2662735	2185.984903	2505.803807	2296.110106
OCLN	155.8124491	150.7425185	160.8532547	727.4582293	730.1907708	716.4814876
ADM2	265.972843	281.5191404	268.4004889	1072.233718	1213.927828	1011.794743
MT1E	599.4313327	466.2036829	451.6983838	39.71235991	44.7055574	54.50412423
BTG1	943.8066181	932.4073659	922.1006344	2988.355084	3514.544589	2743.043925

WSB1	2044.418121	2239.175291	2083.610764	5064.228443	5468.979855	5252.215608
WIPF2	1089.694707	1243.875351	1107.268916	3117.420253	3207.337169	3057.185877
PTPN14	1748.672199	1957.65615	2114.472145	5493.843973	5145.724286	5471.223089
WAC	2083.123125	2175.284422	2210.797059	5085.88973	4961.170575	5015.370413
HMGB3	2151.601207	2057.485633	2101.379438	791.5395374	753.1166977	729.3642806
AKR1C2	895.1772551	798.6358594	846.3499738	167.874976	210.9185272	182.3410702
PSPH	1247.492028	1181.981072	1303.659518	3158.93772	3371.257546	3158.266253
ABCG1	592.4842808	526.1013724	596.6533517	1776.225553	2072.503789	1800.618068
KDM3A	1052.974576	1129.071446	1033.388642	2732.932405	3054.879755	2967.99731
PIP5K1A	1959.068627	2093.424246	2071.453251	4760.970421	4706.692786	4865.731818
ULK1	1720.883991	1801.922158	1648.745861	4345.79575	4668.865007	4181.952805
SLC30A1	458.5054233	523.1064879	500.3284376	1674.236992	1561.25562	1505.304813

KMT5B	1022.209061	1193.96061	1200.78825	3159.840274	3350.624212	3127.545747
FAM84B	1388.417938	1568.321169	1523.429953	3820.509534	3814.874231	3705.289464
PTPDC1	414.8382401	494.155938	490.0413108	1527.120749	1750.394517	1561.790905
GFPT1	1323.909599	1466.495097	1587.0231	3936.0364	4210.34647	3893.576438
SPOP	709.5917266	692.816608	686.4319125	1914.316259	1972.776007	1850.167272
TES	3305.804253	3370.243327	3415.326082	7224.03929	7770.742913	7605.80279
GM2A	2802.639211	2825.174353	2832.700631	1130.899704	965.1815212	1017.740647
BEST1	14.88653972	15.97271719	14.96309346	349.2882565	394.3259422	314.1419524
CCSER2	363.2315691	404.3094038	438.605677	1293.359358	1384.725983	1455.755609
E2F1	1339.788575	1205.940148	1181.14919	379.9750801	327.8407542	354.7722995
NTN4	399.9517004	389.3349815	418.0314235	1244.621462	1286.144497	1405.215421
NEDD4L	1114.505607	1123.081677	1023.101515	2735.640066	2799.255671	2965.024358

NHS	418.8079841	479.1815156	494.7172775	1587.591843	1444.333393	1707.465565
DLG1	1460.865764	1669.148946	1610.402934	3908.057237	3932.942755	4119.520808
SULT1A1	1012.284701	1036.230028	963.2491415	292.4273775	238.4296395	241.8001148
MCL1	9661.364277	9741.360895	9589.472521	20641.40162	21446.05829	19643.28637
TNC	2085.107997	2291.086622	2196.769159	5218.565114	5920.620614	5351.314015
GSE1	1533.313591	1715.070508	1540.263433	3890.908718	3901.992753	3942.134658
TSC22D2	1433.077557	1516.409838	1408.401172	3424.288489	3483.594588	3441.687699
MBD6	1975.940039	1872.80109	2017.212037	4672.520165	4633.32982	4404.924222
DDX60	2592.242783	3184.560489	3388.205475	941.3634407	889.5259626	969.1824272
ASF1B	1530.336283	1351.691192	1354.159958	460.3023535	401.2037202	410.2674078
CDCA4	1553.162311	1445.530906	1441.132939	514.4555716	484.8833533	500.4469588
PPP1R3B	417.8155481	386.340097	323.5768961	1268.99041	1396.188946	1422.06215

IFIT1	1296.121391	1263.841248	1464.512772	413.3695646	409.2277946	368.6460766
TUBA1B	10614.10282	10686.74609	9789.603896	3917.082773	3550.079776	4355.375018
IL6R	421.785292	442.2446071	432.994517	1335.779379	1506.233395	1306.117013
TNFRSF10B	3683.922362	3585.875009	3425.613209	8280.929596	8505.518869	7698.955294
CLIP4	328.4963098	377.3554436	381.5588832	1229.27805	1354.922278	1172.334163
KPNA4	3393.13862	3541.950036	3540.64199	7413.575553	8096.291074	7725.711864
RHOB	248.1089953	271.5361922	230.0575619	908.8715098	1056.885229	934.4979845
ARHGAP29	780.0546812	921.4261228	894.9800276	2337.613913	2446.196397	2351.605215
SARS	4154.337017	3652.760762	3638.837291	9338.722455	10956.30045	9607.590626
LPCAT1	2962.421404	2908.032823	2969.238858	1055.085199	880.3555918	1124.766927
SRGAP1	1415.213709	1412.587176	1421.493879	3424.288489	3336.868656	3805.378855
DNAJB1	2271.685961	2028.535083	1999.443364	5846.742444	7269.811411	5764.554375

CEMIP	1975.940039	2033.526557	1841.395689	684.1356549	583.4648389	700.6257424
NEDD4	371.171057	371.3656746	429.2537436	1243.718908	1205.903753	1248.639937
EGLN1	758.2210896	849.5488954	763.1177664	2235.625352	2399.198247	2063.228848
GPX3	5253.956085	4595.151076	4256.064896	1654.380812	1701.103774	1813.500861
ZCCHC8	993.4284172	1111.102139	1122.232009	2635.456612	2840.522339	2735.116052
PPP1R15B	1453.918712	1666.154062	1726.366908	4023.584102	4119.789059	3989.701894
WDR34	4349.846906	3608.83579	3442.446689	1344.804915	1257.487089	1344.765392
POGZ	1233.597925	1197.953789	1243.807144	2891.781845	2868.033451	3171.149046
LARP6	315.594642	315.4611645	270.2708756	1021.690714	1192.148197	1024.677536
SYT7	317.579514	281.5191404	259.0485555	961.2196206	1102.737082	1018.731631
SEC24A	525.99107	648.8916358	676.1447857	1859.260487	1875.340818	1897.734507
MCM5	2324.285068	2018.552135	2084.545958	759.9501602	662.5592866	787.8323411

STK40	656.9926196	657.8762892	686.4319125	1886.337096	1909.729708	1682.690963
ANKRD50	744.3269859	887.4840988	945.480468	2510.001657	2393.466765	2465.568383
MIA2	560.7263294	557.0485119	552.6992647	1472.967531	1687.348218	1546.926144
CDV3	2778.820747	2824.176058	2944.923832	6074.18596	6453.648414	5984.55284
EHF	1556.139619	1781.956261	2000.378557	4955.019453	4633.32982	5018.343365
RANBP1	4899.656439	4287.67627	4440.297984	1868.286023	1968.190822	1952.238632
SLC7A1	3308.781561	3653.759057	3689.337731	8039.045222	7626.309573	7730.666784
LYPD3	1227.643309	1102.117486	889.3688675	253.6175713	265.9407517	266.5747167
IFIT3	1161.150098	1125.078267	1381.280565	296.9401457	283.1351968	369.6370607
NAV1	1052.974576	1148.039048	1098.852176	370.9495437	333.572236	361.7091881
LPIN1	385.0651607	419.2838262	429.2537436	1194.078458	1210.488939	1336.83752
ARHGAP23	792.956349	732.748401	771.5345065	1928.757117	1916.607486	1879.896794

ITPRIP	1005.337649	978.3289278	897.7856076	2472.094405	2294.88528	2408.091307
CHD2	2419.558922	2653.467643	2517.540475	5385.537537	5440.322446	5644.645302
MTHFD2	3000.133971	2928.997014	3163.759073	6508.314258	7151.742887	6512.747353
BRD4	1725.846171	1700.096086	1622.560447	3630.070718	3954.722385	3976.819101
CLCN3	826.6991724	845.5557161	921.1654411	2139.052114	2108.038976	2185.11989
CREBRF	213.373736	225.6146303	230.0575619	826.7391291	974.351892	781.8864367
BACH1	670.8867233	842.5608317	853.8315205	2248.261103	2370.540838	2262.416648
RBMS2	2613.083939	2920.012361	2779.39461	6321.485655	5898.840984	6458.243229
TUBB	22683.11679	20578.84951	19851.34905	9280.056469	10014.04486	9507.501234
JMY	495.2255546	568.029755	609.7460585	1700.411047	1544.061175	1704.492612
IGF2BP2	3115.256545	3161.599708	3255.408021	6648.210071	6564.839159	7195.535382
SLFN5	2266.723781	2658.459117	2910.321678	6669.871358	7227.398446	6763.466325

CDC25A	632.18172	592.9871256	638.7370521	129.0651697	83.67963308	135.7648185
HS3ST1	1036.103164	982.3221071	903.3967676	295.1350385	307.2074201	295.3132549
SLC38A1	3689.876978	4460.381275	4706.828086	11091.48161	10885.23008	10727.40263
JMJD1C	806.8504527	974.3357485	1064.250022	2807.844357	2623.87233	2920.430075
SLC22A15	125.0469336	108.8141358	104.7416542	573.1215578	511.2481692	524.2305767
LMNB1	3387.184004	3253.442832	3482.660003	1425.132189	1200.172272	1411.161326
ETV5	869.3739195	933.4056607	889.3688675	2175.154259	2103.45379	2135.570686
AMMECR1L	508.1272224	574.0195239	567.6623581	1498.239033	1460.381542	1488.458084
EIF1	7394.640496	6621.689569	6189.109532	17672.00016	21208.77495	16666.37021
ZNF697	328.4963098	382.3469177	404.9387168	1181.442707	1156.613011	1131.703816
FOXO3	1178.02151	1220.91457	1168.056483	2633.651505	2678.894555	2705.38653
MCM4	8654.041756	8704.132573	8789.882214	4067.80923	3534.031627	4099.701126

ZFAND3	943.8066181	966.3493899	981.0178149	2236.527906	2474.853806	2275.299441
RSRC2	1457.888456	1501.435416	1643.134701	3431.508918	3668.148299	3556.641852
TNFRSF21	1736.762967	1874.79768	1818.951049	665.1820286	570.8555791	702.6077105
HIPK3	956.7082859	1100.120896	1212.01057	2887.269076	2843.961228	2975.925183
SKP2	1711.952068	1811.905106	1948.942923	611.9313641	473.4203899	627.2929207
KIF20A	2046.402993	2124.371386	2189.287612	875.4770254	797.8222551	891.8856692
RIT1	482.3238869	471.195157	476.948604	1273.503178	1407.65191	1280.351427
KDM5B	1962.045935	2132.357745	2234.176892	4585.875016	4770.885382	4867.713786
SOCS7	111.1528299	101.8260721	113.1583943	516.2606789	514.6870582	481.6182614
BLZF1	410.8684962	460.213914	456.3743505	1204.909102	1352.629685	1283.32438
EPPK1	1186.953434	1523.397902	1447.679292	442.2512809	412.6666837	428.1051212
UHRF1BP1	902.1243069	1015.265836	1120.361623	2755.496246	2531.022326	2747.007861

KDM7A	684.780827	794.6426801	726.6452261	1841.209414	1866.170447	1853.140224
GPRC5A	6041.950254	6015.724611	5324.055692	12060.82422	13181.26165	12634.056
SIGMAR1	4823.238869	4396.490406	4504.826325	2160.713401	2018.627861	2134.579702
CBX4	1836.006565	1784.951146	1652.486634	4759.165314	4948.561315	3981.774021
PRDM1	25.80333551	33.94202402	43.95408704	292.4273775	421.8370544	359.7272199
GARS	5104.098251	4816.772527	5084.646196	10545.43666	12762.86349	11276.40781
OGT	1961.053499	2395.907578	2571.781688	6298.019261	5854.135426	6684.187599
N4BP3	263.987971	251.5702957	205.7425351	950.388977	902.1352223	800.7151341
SLBP	1823.104897	1871.802795	1966.711597	767.1705892	776.0426246	808.6430068
TMEM40	255.0560472	212.6367976	160.8532547	862.8412745	923.9148529	862.1561469
AKR1C1	552.7868415	462.2105036	504.0692109	97.47579252	114.6296344	105.0443122
XPOT	5548.709571	6148.497823	6673.539683	13335.22995	14465.11356	13721.16553

SLC2A3	1387.425502	1322.740642	1079.213116	3419.775721	3923.772384	3360.427005
PPP1R9A	54.58397897	50.91303604	66.39872723	342.9703811	384.0092751	403.3305193
CHIC2	339.4131056	336.4253558	299.2618692	1010.860071	1069.494489	938.4619208
SHOC1	169.7065528	197.6623752	190.7794416	692.2586376	643.0722487	699.6347583
MALT1	289.7913065	314.4628696	350.697503	947.6813161	1047.714858	1000.893918
DUSP14	1157.180354	1088.141358	998.7864884	2558.739554	2744.233446	2515.117587
DUT	2329.247248	2089.431067	2244.464019	948.5838698	872.3315174	807.6520227
UAP1	1271.310492	1298.781566	1351.354378	3025.359783	3497.350144	2976.916167
EPC2	320.5568219	308.4731007	314.2249627	905.2612953	910.1592968	953.326682
DAPP1	324.5265659	325.4441127	367.5309831	1029.813697	1385.872279	1159.45137
E2F2	295.7459224	255.563475	299.2618692	23.46639449	29.80370493	27.74755415
GUCD1	1709.967196	1544.362093	1530.9115	607.418596	640.779656	626.3019366

SH3BP4	2148.623899	2069.465171	2038.721484	4187.848864	4244.73536	4316.726639
NCOA7	778.0698093	870.5130868	1018.425549	2404.402882	2363.66306	2434.847877
MCM3	3696.82403	3385.217749	3406.909342	1611.058237	1493.624136	1627.195854
IRF2BP2	1505.525384	1394.617869	1274.668524	3642.706468	3979.940905	3242.4999
TGFBR2	942.8141822	1059.190809	1010.008809	2372.813505	2251.326019	2339.713406
TAB2	1375.51627	1513.414954	1563.643267	3288.00289	3472.131625	3779.613269
FUT11	363.2315691	375.3588539	271.206069	1096.602666	1145.150047	1159.45137
IL20RB	1822.112462	1615.241026	1627.236414	3852.098912	4574.868707	3971.86418
EGFR	9387.451946	11933.61633	11994.78979	28340.18412	26362.52331	28999.16704
C17orf51	258.0333551	242.5856423	249.6966221	808.6880564	776.0426246	765.0397074
COLGALT1	5327.396347	5136.226871	4661.003613	2389.962024	2180.255645	2170.255128
CHPF	1724.853735	1567.322874	1344.808025	540.629627	513.5407619	525.2215608

HSPA1B	755.2437817	732.748401	614.4220252	2111.072951	2109.185272	1783.771338
CDCA3	1139.316506	1060.189103	1067.990796	398.9287064	339.3037177	392.4296945
PTGS2	1018.239317	1260.846363	1160.574936	2783.475409	2858.863081	2775.746399
WWC1	1204.817281	1206.938443	1109.139303	2760.009014	2532.168623	2635.026661
TUBB4B	16121.13008	13860.32534	13090.83639	6098.554908	6369.968781	6410.675994
TELO2	1540.260643	1392.62128	1351.354378	563.1934679	503.2240948	477.6543251
C16orf72	1505.525384	1646.188165	1832.978949	3777.18696	4069.352019	3884.657581
NRG1	6089.587181	6353.148262	5930.060977	12078.87529	11558.10603	12671.71339
TGFA	1508.502691	1641.196691	1384.086145	3521.764281	3441.181623	3349.52618
ORC1	976.5570055	914.438059	992.240135	334.8473984	294.5981603	343.8714747
PREPL	542.8624817	640.9052772	693.9134592	1677.847206	1664.422291	1781.78937
SPAG1	263.987971	306.476511	306.7434159	859.23106	982.3759664	893.8676374

RIMKLB	1615.685777	1845.84713	1831.108562	3986.579403	3730.048302	4025.37732
CHMP4C	353.3072093	364.3776109	443.2816437	1166.099296	1409.944503	1169.361211
SGMS1	405.9063163	466.2036829	431.1241303	1177.832493	1125.663009	1182.244004
CDK17	481.3314509	586.9973567	578.8846782	1463.941995	1446.625986	1471.611354
CREBBP	1263.371004	1377.646857	1397.178852	3098.466627	2950.566788	3470.426237
RBM14	1502.548076	1419.57524	1706.727848	583.0496478	465.3963155	536.1223856
ZNF217	1249.4769	1398.611049	1590.763873	3445.047223	3417.1094	3437.723763
BEX2	272.9198948	226.6129251	257.1781688	870.0617036	1085.542637	816.5708794
GOT1	1275.280236	1114.097024	1111.009689	2571.375304	2816.450116	2815.385763
ZFP36L1	1871.734261	1963.645919	1811.469502	3932.426185	4319.244622	3869.79282
HSPA1A	1296.121391	1068.175462	836.9980404	3559.671534	3926.064977	3232.590059
STK24	1112.520735	1195.957199	1217.62173	2869.218004	2982.663086	2526.018412

FOSL1	3544.981325	3141.633812	2620.411742	7620.260335	8168.507744	7973.457883
TM4SF1	2014.645042	1881.785744	1718.885361	773.4884647	764.5796611	695.670822
ARHGAP26	217.3434799	194.6674907	202.936955	676.9152258	652.2426195	760.084787
ESRP1	2519.794956	2782.247675	2906.580905	5614.78616	6108.613215	5792.30193
IGF2R	3808.969296	4243.751298	4024.136947	7891.026426	7815.44847	8107.240733
ARHGEF28	447.5886275	467.2019778	406.8091034	1136.315026	1153.174122	1222.874351
MFSD2A	451.5583715	404.3094038	418.0314235	80.32727346	74.50926233	101.0803758
GPR153	5541.762519	5462.669278	5078.099843	2751.886031	2625.018627	2585.477457
IARS	5761.090871	6071.629121	6479.019468	11631.20869	12284.85791	12251.53614
AP1S1	5074.325172	4397.488701	4092.406061	1995.546086	1805.416741	1751.068864
FOXQ1	182.6082205	179.6930684	146.8253546	737.3863193	765.7259575	578.7347009
SPRYD4	850.5176359	887.4840988	767.7937331	280.6941803	251.0388992	279.4575097

SLC12A4	748.2967298	708.7893252	631.2555053	1729.292764	1789.368592	1645.033568
ARNTL	432.7020878	392.3298659	453.5687705	1087.577129	1158.905603	1206.027622
LMO4	695.6976228	668.8575323	717.2932927	1551.489698	1701.103774	1659.898329
ASB1	735.3950621	719.7705683	732.2563862	1649.868044	1709.127848	1612.331093
MCM6	2285.580065	2525.685905	2477.327161	1071.331164	900.988926	1056.389026
WDR26	2022.58453	2162.306589	2255.686339	4313.303819	4586.33167	4352.402066
DSN1	1097.634195	1085.146474	1128.778363	449.4717099	403.4963129	387.4747741
KREMEN1	1371.546526	1562.3314	1587.0231	620.0543468	585.7574315	583.6896213
ICMT	2524.757136	2597.563133	2633.504449	1258.159766	1125.663009	1209.991558
CSGALNACT2	244.1392514	286.5106146	220.7056285	787.0267692	880.3555918	796.7511978
ZFP36	725.4707023	632.9189186	563.9215848	1674.236992	1805.416741	1651.970456
CEP85	1007.322521	941.3920193	972.6010749	342.9703811	380.570386	349.8173791

SYNRG	569.6582532	635.9138031	692.0430725	1550.587144	1578.450065	1673.772106
HERC5	801.8882728	841.5625368	890.3040608	302.3554675	264.7944554	288.3763664
BCL2L1	1779.437714	1667.152357	1479.475866	4251.027618	5243.159475	3958.981387
LGR4	902.1243069	1074.165231	1108.204109	347.4831492	270.5259371	327.0247454
SPC24	647.0682598	522.1081931	582.6254516	161.5571006	123.8000051	144.6836752
CLGN	69.47051869	55.90451016	66.39872723	345.678042	483.737057	359.7272199
TARS	9519.445932	9480.805946	10004.69836	17795.65001	20159.91379	18727.61709
EPSTI1	130.0091135	137.7646857	144.9549679	508.1376962	690.0703988	554.9510831
CCNF	1046.027524	965.3510951	1000.656875	364.6316683	312.9389018	382.5198537
FAM83B	499.1952986	591.9888308	619.0979919	1478.382853	1442.0408	1498.367924
EFNA1	1484.684228	1234.890698	1112.880076	3170.670918	3303.626062	3253.400725
JDP2	290.7837425	250.5720009	267.4652956	765.365482	806.9926258	859.1831947

GPT2	1152.218174	1160.018586	1104.463336	2371.910951	2336.151948	2445.748702
STMN1	3908.212894	3728.631169	3906.302586	1927.854563	1952.142673	2005.751772
SKIL	1019.231753	1151.033932	1243.807144	2530.760391	2736.209372	2713.314403
TEAD1	1932.272855	2345.992837	2366.039153	5581.391675	4837.37057	5592.123146
NFKB1	1547.207695	1709.080739	1678.672048	3382.771022	3358.648287	3307.904849
ERBIN	1864.787209	2236.180406	2316.473906	4997.439474	4746.813159	4839.966232
ALDH1B1	1092.672015	1046.212976	1055.833282	416.0772255	347.3277921	414.2313441
CCHCR1	1124.429967	959.3613261	966.9899148	355.606132	369.1074226	370.6280448
PRPF38B	1347.728062	1473.483161	1471.059126	2925.176329	3003.29642	3243.490884
FUT1	502.1726065	470.1968622	436.7352904	1186.858029	1149.735233	1253.594857
OXSR1	2223.056598	2334.013299	2345.4649	4388.21577	4476.287221	4364.293875
EP300	1183.976126	1405.599113	1338.261671	3071.390018	2786.646411	3160.248221

IMPDH1	2001.743374	1834.865887	1749.746741	835.7646655	794.3833661	806.6610386
HIVEP2	1059.921628	1295.786682	1443.003326	3292.515658	3162.631612	3673.577973
PPP4R3A	1126.414839	1244.873646	1181.14919	2462.166315	2467.976028	2469.53232
CMTR2	455.5281154	523.1064879	525.5786578	1268.99041	1248.316718	1221.883367
IL6ST	1413.228837	1774.968198	1969.517177	4485.691563	4295.172399	4521.860343
CDC42SE1	2517.810084	2513.706367	2450.206554	4786.241923	4781.202049	4570.418563
UBE2Z	3393.13862	3265.42237	3277.852661	6205.056237	6842.242874	6232.29886
CBX2	984.4964934	958.3630313	907.137541	371.8520974	298.0370493	332.9706498
UPP1	2379.861483	2195.250319	1954.554083	5119.284214	6308.068778	5017.352381
CLSPN	665.9245434	708.7893252	789.30318	236.4690522	175.3833406	212.0705925
DTL	1203.824845	1248.866825	1338.261671	491.8917308	429.8611288	527.2035289
TPBG	3618.421588	3671.728364	3624.809391	6500.191275	6804.415095	6623.73757

SEMA6A	451.5583715	501.1440018	470.4022506	1330.364057	1115.346342	1269.450603
RESF1	927.9276424	1137.057805	1117.556043	2428.77183	3048.001977	2816.376747
MBNL2	449.5734995	526.1013724	577.0142915	1427.83985	1293.022275	1428.008055
LRIF1	362.2391331	320.4526386	397.45717	975.6604788	1129.101898	991.975061
ARID4B	682.7959551	797.6375646	793.0439534	1744.636175	1729.761182	1868.004985
PRKAB2	960.6780298	964.3528002	1037.129415	2058.72484	2082.820456	2211.87646
FOS	266.9652789	270.5378974	220.7056285	787.0267692	727.8981781	780.8954526
FOSB	280.8593827	256.5617698	231.9279486	750.0220702	872.3315174	755.1298666
QPCTL	1115.498043	1069.173757	926.7766012	389.9031701	374.8389043	341.8895065
ZNF367	428.7323439	458.2173243	555.5048447	118.2345261	98.58148554	114.9541529
TRPM4	1044.042652	921.4261228	943.6100813	374.5597583	326.6944579	361.7091881
S1PR5	311.6248981	285.5123197	273.0764556	41.51746718	41.26666837	53.51314015

KLF10	1230.620617	1365.66732	1218.556924	2584.011055	2854.277895	2767.818527
UHRF1	2427.49841	2386.922925	2284.677333	1083.064361	897.550037	1085.127564
FERMT2	299.7156663	315.4611645	299.2618692	806.8829492	810.4315149	851.2553221
ISG15	6132.261928	5066.346233	4472.094558	1938.685207	2158.476015	1810.527909
ANXA3	1829.059513	1905.74482	1709.533428	3612.019645	4307.781659	4021.413384
CSNK1G3	689.7430069	799.6341542	883.7577075	1940.490314	1946.411191	1895.752539
LMO7	1805.24105	1838.859066	2038.721484	3809.678891	4478.579814	4119.520808
F2RL1	1155.195482	1204.941853	1282.150071	2516.319533	2567.703809	2457.640511
SYTL3	382.0878528	401.3145194	324.5120894	1100.21288	1102.737082	950.3537298
XDH	248.1089953	302.4833317	256.2429755	760.8527138	831.064849	785.850373
JMJD6	758.2210896	703.7978511	652.7649522	1581.273967	1613.985252	1639.087663
VEZF1	1017.246881	1137.057805	1167.12129	2571.375304	2293.738983	2612.234027

PATJ	1103.588811	1133.064626	1201.723443	2347.542003	2391.174173	2621.152883
SYS1	435.6793958	443.242902	463.8558972	1053.280091	1160.0519	1090.082485
FRMD4B	403.9214444	365.3759057	442.3464504	1037.034126	1038.544487	1144.586609
BIRC2	1077.785476	1311.759399	1315.817031	2810.552018	2806.133449	2761.872622
RNASE7	71.45539065	46.91985674	63.5931472	319.5039866	453.933352	345.8534428
HSPA9	9053.993457	9231.23224	9625.009868	17626.87248	19871.04711	17146.0065
YY1AP1	813.7975046	829.582999	823.9053336	1681.457421	1808.85563	1852.14924
FOXC1	365.2164411	369.369085	374.0773365	83.937488	77.94815136	92.16151915
TTBK2	338.4206696	417.2872365	471.337444	1296.969573	1102.737082	1270.441587
KIFC1	1632.557189	1565.326284	1455.160839	678.7203331	601.8055803	689.7249175
PLIN2	1169.089586	943.3886089	738.8027396	2674.266419	3273.822357	3061.149814
AC118549.1	1027.171241	1232.894108	1197.047477	2608.380003	2462.244546	2584.486473

FYN	120.0847537	123.7885582	125.3159077	421.4925473	476.8592789	473.6903888
PCDH7	707.6068546	720.7688631	725.7100328	239.1767131	226.966676	272.5206212
AJUBA	3646.209795	3504.014833	3352.668128	7165.373304	7117.353997	6375.000567
NFE2L1	6179.898855	5854.999144	6046.960144	11348.7094	12349.05051	10820.55514
KRT13	372.163493	305.4782162	295.5210958	60.47109351	53.87592815	61.44101277
IL32	190.5477084	196.6640804	168.3348014	643.5207413	758.8481794	568.8248601
UNG	2778.820747	2577.597236	2669.041796	1314.118092	1170.368567	1060.352962
AHNAK2	1197.870229	1484.464404	1288.696424	3021.749568	2973.492715	2862.952998
RNMT	1005.337649	1106.110665	1103.528143	2222.989602	2504.657511	2260.434679
BBC3	293.7610504	213.6350924	224.4464019	1101.115434	1064.909303	747.201994
MAT2A	4532.455126	4960.526982	5271.684864	2408.013097	2304.05565	2511.153651
CDCA7	1646.451293	1744.021058	1903.118449	778.9037865	645.3648414	751.1659303

FERMT3	331.4736177	353.3963678	313.2897693	64.98386168	71.0703733	71.35085354
VCP	9588.91645	8925.754024	8799.234148	4916.209647	5056.313171	5003.478604
PPP2R2A	1491.63128	1580.300707	1624.430834	2999.185727	3104.170498	3084.933431
MVP	3592.618252	3169.586067	3001.035432	1526.218196	1485.600061	1331.882599
GOSR1	691.7278789	669.8558271	722.9044528	1510.874784	1491.331543	1529.088431
INPP4B	516.0667102	553.0553326	539.6065579	1190.468244	1251.755607	1254.585841
TJP1	1561.101798	1831.871003	1785.284088	3636.388593	3462.961254	3766.730476
CDC42EP1	1295.128955	1094.131127	1031.518255	2622.820862	2738.501965	2524.036444
FAM53C	1072.823296	1180.982777	1180.213997	2418.84374	2410.66121	2275.299441
SPATS2	1020.224189	1048.209565	960.4435614	2021.720141	2117.209346	2157.372335
TMEM154	973.5796976	1078.15841	1019.360742	2191.400224	2058.748233	2148.453479
BRAF	357.2769532	395.3247504	417.0962302	1024.398375	944.5481871	1025.66852

EGLN3	2001.743374	1843.85054	1295.242778	4747.432117	5652.38727	4920.235942
ARID1A	1825.089769	2065.471991	2006.92491	4026.291763	3866.457567	4453.482442
MPZL3	266.9652789	295.495268	305.8082226	810.4931637	905.5741114	759.0938029
AVPI1	747.3042939	628.9257393	548.9584913	1677.847206	1663.275994	1623.231918
GPR157	646.0758238	726.7586321	671.468819	238.2741595	200.6018601	237.8361785
BNIP3	2407.64969	2377.938271	2007.860104	5196.903827	6053.59099	4821.137534
BNIP3L	1709.967196	1790.940915	1778.737735	3505.518316	3951.283496	3393.129479
TRIB1	422.777728	358.3878419	386.2348499	950.388977	1194.44079	1092.064453
RMI2	887.2377672	823.59323	807.0718535	304.1605748	311.7926054	336.9345861
CSTF3	840.5932761	907.4499952	919.2950544	326.7244157	366.8148299	336.9345861
LAMB3	7045.303031	6576.766302	5844.958383	14143.01545	17035.10996	13531.88757
RAB10	1869.749389	1853.833489	1965.776403	3499.200441	3663.563114	3579.434486

IL1RAP	1826.082205	1969.635688	2066.777284	3838.560607	3732.340894	3849.973139
BCL10	799.9034009	845.5557161	948.286048	1942.295421	2317.811207	1969.085361
ADAMTS1	1788.369638	1897.758461	1857.293976	874.5744717	809.2852185	912.6963348
CDC45	1614.693341	1375.650268	1471.059126	587.562416	605.2444694	670.8962201
CDCP1	3646.209795	3826.464061	3566.827403	6580.518548	7210.204001	6756.529436
AXL	1164.127406	1180.982777	1085.759469	2501.878675	2552.801957	2232.687125
ZNF488	327.5038738	279.5225508	273.0764556	53.25066443	21.77963053	39.63936308
SLC29A1	1711.952068	1554.345041	1402.790012	619.1517932	555.9537266	673.8691723
ASNS	58.55372289	87.84994453	96.32491415	407.0516891	358.7907555	400.3575671
BCAT1	2174.427235	2347.989427	2717.67185	5225.785543	5145.724286	5016.361397
GPATCH8	1055.951884	1143.047574	1226.03847	2496.463353	2294.88528	2481.424129
MYORG	714.5539065	752.7142975	779.9512466	275.2788585	291.1592713	261.6197963

HSPA13	555.7641495	706.7927356	700.4598126	1615.571006	1591.059325	1583.592555
AKNA	230.2451476	273.5327818	242.2150754	686.8433158	776.0426246	696.6618061
KCNN4	2667.667918	2241.17188	2153.750265	1050.57243	1024.788931	955.3086501
LRP8	1165.119842	1241.878761	1183.95477	439.54362	378.2777934	517.2936881
ALDH1A3	1010.299829	1073.166936	1024.971902	428.7129763	356.4981628	433.0600416
SPRED2	832.6537882	906.4517004	810.8126268	1789.763857	1767.588962	1803.59102
SPHK1	1070.838424	925.4193021	822.9701403	314.9912184	340.450014	266.5747167
SLC1A4	157.797321	148.7459288	181.4275082	512.6504643	530.735207	543.0592741
SNN	625.2346682	645.8967513	675.2095924	213.0026577	218.9426016	242.7910988
DDX11	2355.050583	2098.415721	2116.342531	1050.57243	852.8444796	960.2635705
AFF4	1949.144267	2495.737061	2612.930195	5625.616803	5278.694662	5581.222321
SCAF8	505.1499144	603.9683687	589.171805	1370.076417	1314.801906	1304.135045

SLC6A9	367.201313	318.4560489	413.3554568	988.2962297	982.3759664	949.3627457
POLD1	3344.509257	3048.792393	2945.859025	1607.448023	1460.381542	1521.160558
SLC9A3R1	1369.561654	1170.001534	1090.435436	460.3023535	443.6166849	493.5100703
GPX2	683.7883911	597.9785997	607.8756718	172.3877442	222.3814906	151.6205638
ZSWIM6	442.6264476	533.0894362	499.3932442	1262.672535	1134.83338	1382.422787
GINS1	1009.307393	1112.100434	1104.463336	450.3742636	361.0833482	437.0239779
MARK3	1404.296913	1470.488276	1425.234652	2672.461311	3002.150124	2836.196428
TFE3	900.139435	869.5147919	839.8036204	1763.589802	1940.67971	1778.816418
ZDHHC12	1491.63128	1348.696308	1252.223884	578.5368797	597.220395	578.7347009
ZFAND1	1060.914064	1153.030522	1121.296816	2167.93383	2375.126024	2226.741221
P4HA1	1204.817281	1303.77304	1253.159077	2507.293996	2913.885305	2528.00038
SNRNP25	861.4344317	781.6648474	727.5804195	296.0375921	298.0370493	276.4845575

ATF2	436.6718317	454.224145	516.2267244	1084.869469	1140.564862	1151.523497
ATXN2	1056.94432	1089.139653	1179.278803	2273.532605	2229.546388	2537.910221
MED1	982.5116214	1216.921391	1268.122171	2849.361824	2517.26677	2898.628425
IFI44	2600.182271	2737.324408	2741.051683	1388.12749	1443.187096	1402.242469
TBC1D20	619.2800523	671.8524167	618.1627986	1325.851289	1471.844505	1450.800689
CTSC	3938.978409	4072.044588	3973.636507	2142.662328	2155.037126	2268.362552
TTC17	1075.800604	1189.967431	1132.519136	2178.764473	2365.955653	2319.893724
LSS	1426.130505	1281.810554	1227.908857	582.1470942	561.6852083	576.7527328
PMEPA1	1180.998818	1212.928211	1082.018696	2333.101145	2419.831581	2254.488775
ATP2B1	1584.920262	1743.022763	1995.70259	3782.602282	3990.257572	3668.623053
APBB2	641.1136439	743.7296441	809.8774335	1735.610639	1612.838955	1799.627084
CCNE1	246.1241233	264.5481284	301.1322559	52.3481108	49.29074277	42.61231531

ARL5B	756.2362177	926.4175969	1001.592068	2074.970806	2088.551938	2127.642813
N4BP2L2	1283.219724	1511.418364	1605.726967	3210.383278	3029.661236	3225.65317
WASL	945.7914901	1061.187398	1096.046596	2189.595117	2322.396392	2076.111641
TOM1L1	747.3042939	800.6324491	882.8225141	1686.872743	1893.681559	2007.73374
PKMYT1	1908.454392	1741.026174	1667.449727	842.0825409	699.2407695	662.9683475
SSH1	4636.660904	5151.201293	4803.153001	8892.86096	8555.955908	9383.628224
INSIG2	511.1045303	543.0723844	457.3095439	1197.688673	1223.098199	1126.748895
KLHDC7B	29.77307944	30.94713955	40.21331367	201.2694605	261.3555663	248.7370033
UBE2T	1194.892921	1107.10896	1053.027702	488.2815162	410.374091	472.6994047
RBM22	1143.28625	1126.076562	1230.714437	2227.50237	2563.118624	2442.77575
RBCK1	2074.191201	1930.70219	1797.441602	4058.783694	4495.774259	3722.136193
DAG1	3167.855652	3166.591183	3284.399014	1726.585103	1416.822281	1521.160558

TUBA4A	3269.084122	2724.346575	2615.735775	1277.113393	1266.65746	1347.738345
CWC22	588.5145369	635.9138031	653.7001455	1337.584486	1334.288944	1453.773641
MSN	7882.918999	7940.437032	7571.325291	4408.974504	4328.414993	4621.949735
NAMPT	2898.905501	3185.558784	3316.195588	6399.105268	7618.285499	6138.155372
C9orf3	955.7158499	947.3817882	962.3139481	393.5133846	334.7185323	417.2042964
SLC25A1	1824.097333	1642.194986	1549.615366	792.442091	745.0926233	700.6257424
KMT2C	740.357242	932.4073659	851.9611339	2046.991643	1836.366742	2295.119122
FKBP4	5917.895756	5556.508992	5477.4274	3213.090938	3125.950129	2971.961247
NR3C1	2063.274405	2480.762638	2604.513455	4968.557757	5008.168725	4940.055623
SETD5	2541.628548	2588.578479	2787.81135	5110.258678	4735.350195	5429.601757
RAB3GAP1	1310.015495	1245.871941	1362.576698	2431.479491	2621.579738	2653.855358
CHEK2	763.1832696	742.7313492	684.5615258	286.1095021	272.8185298	280.4484938

CBX6	2200.23057	2177.281012	2139.722365	1135.412472	1070.640785	966.209475
RYK	1243.522284	1325.735527	1240.06637	2466.679083	2442.757508	2378.361785
ADNP	2432.46059	2584.5853	2694.292016	4684.253363	4679.181674	5054.018792
VIM	262.003099	256.5617698	189.8442483	33.39448447	34.38889031	20.81066562
DSG3	1005.337649	1082.151589	1111.944883	2072.263145	2101.161198	2160.345288
LATS2	426.7474719	498.1491173	507.8099843	1278.9185	1070.640785	1209.000574
RYBP	537.9003018	647.8933409	626.5795386	1352.927898	1341.166722	1440.890848
GEMIN4	1165.119842	1109.10555	1080.148309	510.8453571	433.3000178	488.5551499
RNF185	1006.330085	907.4499952	886.5632875	1948.613297	2309.787132	1970.076345
KMT2E	2321.30776	2517.699547	2471.716001	4827.75939	4408.655737	5240.323799
HES4	1089.694707	961.3579158	936.1285346	428.7129763	385.1555714	359.7272199
ASS1	1801.271306	1653.176229	1569.254427	3527.179603	4417.826108	3503.128712

SPIRE1	1019.231753	1139.054394	1220.42731	2319.56284	2450.781582	2281.245345
MSH6	2151.601207	2369.951913	2482.003128	1146.243116	1055.738932	1173.325147
THSD4	233.2224556	233.6009889	237.5391087	630.8849905	607.5370621	650.0855545
PYCR3	965.6402097	746.7245285	794.91434	304.1605748	287.7203822	301.2591594
SNX24	276.8896388	243.5839371	264.6597156	666.9871358	693.5092878	705.5806628
RPP25	834.6386602	763.6955406	736.9323529	308.673343	290.0129749	239.8181466
TBL1X	684.780827	735.7432855	760.3121864	1476.577746	1513.111173	1574.673698
PLCXD2	85.34949438	98.8311876	107.5472342	405.2465818	335.8648287	419.1862645
AGO2	1393.380118	1630.215448	1660.903374	3334.033126	3029.661236	3405.021288
PDK1	818.7596845	1068.175462	962.3139481	2052.406965	2231.838981	2207.912523
PDPN	685.773263	702.7995563	631.2555053	237.3716058	191.4314894	256.6648759
RND3	1528.351411	1495.445647	1408.401172	2854.777146	3480.155699	2987.816992

STK32A	4.962179906	4.991474121	7.48154673	134.4804915	194.8703784	191.2599268
SERPINB8	149.8578332	145.7510443	131.8622611	453.0819245	476.8592789	439.0059461
TACSTD2	4969.126958	4578.180064	4549.715605	2535.273159	2662.846406	2538.901205
RELA	3051.740642	2799.218687	2701.773563	5223.980436	5964.179875	5392.935347
PRKCE	164.7443729	144.7527495	177.6867348	527.0913225	471.1277972	565.8519079
RREB1	1412.236401	1649.18305	1551.485753	3102.076841	2904.714934	3104.753113
PFKFB3	1449.948969	1569.319464	1297.113164	2968.498904	3170.655686	2833.223476
HSPA5	13759.13244	13119.59058	13175.00379	27336.54448	39063.48679	29072.49986
PDGFA	1159.165226	1053.20104	992.240135	2295.193892	2525.290845	2122.687893
C6orf223	477.361707	487.1678742	439.5408704	1248.231676	1437.455615	1048.461153
TICRR	1125.422403	1210.931622	1268.122171	491.8917308	395.4722385	528.194513
PLOD2	4531.46269	5264.008608	4980.839735	9011.095486	9406.507795	8862.3706

PSAT1	4791.480917	5089.307014	5368.944972	9561.653203	9647.230027	8752.371367
RBM39	5090.204148	5199.119445	5520.446293	8956.039714	9604.817062	9167.593696
PICALM	2951.504608	3246.454768	3221.741061	5497.454187	5668.435419	5820.049484
ITGA5	4196.019329	4083.025831	3739.838172	7530.004972	8604.100354	7348.14693
SLMAP	1180.998818	1221.912865	1312.076258	2400.792668	2330.420466	2501.24381
HIST1H2BK	1193.900485	1139.054394	1224.168084	535.2143052	572.0018754	514.3207359
DHCR24	5564.588547	5199.119445	5194.063817	2838.53118	2716.722334	3064.122766
SHCBP1	1054.959448	1077.160115	1035.259029	497.3070526	427.5685361	458.8256276
OR51B5	148.8653972	164.718646	202.936955	15.34341178	8.024074405	10.90082485
MARS	7792.607324	7156.775595	7022.366799	12482.31676	13127.38573	12920.45039
RIOK3	934.8746943	1040.223207	1011.879195	1919.73158	2175.67046	2031.517358
JUN	1860.817465	1853.833489	1172.73245	8866.686904	8546.785537	8536.336839

H1F0	4411.377936	3918.307185	3997.01634	8220.458502	9517.69854	7644.451169
NR1D2	904.1091789	1028.243669	1048.351736	2117.390826	2164.207497	1944.310759
GGNBP2	1277.265108	1325.735527	1327.974545	2388.156917	2650.237146	2489.352001
SH2D5	431.7096518	402.3128142	376.8829165	1027.106036	1068.348192	893.8676374
CXCL1	218.3359159	178.6947735	287.1043558	730.1658902	717.581511	808.6430068
RBAK	524.9986341	507.1337707	532.1250112	1098.407773	1114.200046	1165.397274
MCM2	3202.590911	3070.754879	3009.452172	1643.550168	1444.333393	1671.790138
CTBP2	1399.334734	1258.849773	1375.669405	2516.319533	2955.151974	2736.107036
CDK2AP1	2968.37602	2800.216982	3076.786093	1585.786736	1561.25562	1411.161326
MYBL2	5718.416124	5175.160369	5225.860391	2803.331588	2521.851956	2971.961247
FOXK2	2380.853919	2316.043992	2175.259712	4128.280324	4145.007578	4051.142906
SH3BP2	1012.284701	1288.798618	1260.640624	2946.837616	2586.044551	2564.666791

KRT15	1097.634195	999.2931191	981.9530083	460.3023535	439.0314996	454.8616913
H3F3A	3887.371738	3818.477703	3863.283693	2212.158958	2109.185272	1949.265679
MDM4	525.99107	616.9462014	685.4967191	1422.424528	1498.209321	1375.485899
THAP1	143.9032173	139.7612754	155.2420946	447.6666027	440.1777959	475.6723569
ZNF292	787.0017331	899.4636367	954.8324014	1880.01922	1795.100074	1938.364854
NFKBIA	2076.176073	1897.758461	2054.619771	3713.105652	3756.413118	3557.632836
DNAH11	664.9321074	743.7296441	691.1078792	1434.157725	1472.990801	1428.999039
TET3	788.9866051	999.2931191	907.137541	2029.843124	1873.048225	2007.73374
DCTN4	1228.635745	1373.653678	1521.559566	2784.377962	2774.037151	2894.664489
RFC4	1870.741825	1733.039815	1716.079781	896.235759	873.4778138	909.7233826
XPR1	2393.755587	2843.143659	2985.137145	5546.192083	5341.740961	5622.843652
PTPN12	806.8504527	892.4755729	889.3688675	1671.529331	1804.270445	1768.906577

PTGES	2081.138253	1869.806206	2049.008611	945.8762089	947.9870761	1052.42509
MED13L	2648.811634	3465.081335	3311.519621	7421.698536	6348.18915	7802.017638
CREB1	1001.367905	1170.001534	1206.39941	2360.177754	2228.400092	2445.748702
HES1	387.0500327	375.3588539	331.0584428	907.9689562	1025.935227	844.3184335
AGRN	14779.35663	14182.77457	13069.32694	8061.609063	6869.753987	7073.644341
FBXO11	485.3011948	602.9700739	647.1537921	1458.526673	1320.533388	1426.026087
CDC20	4374.657805	3786.532268	3514.456576	1939.58776	1759.564887	1910.6173
ANKLE2	3561.852737	3707.666977	3572.438563	6023.642956	6181.976181	6351.216949
SIVA1	1776.460406	1508.423479	1403.725205	710.3097103	656.8278048	621.3470162
MLLT10	722.4933943	811.6136921	760.3121864	1528.023303	1547.500064	1654.943408
LRRC3	265.972843	257.5600647	253.4373955	49.64044989	43.55926105	61.44101277
LARS	3749.423137	3947.257735	4091.470868	6856.699961	6647.372496	7034.995962

PNP	1538.275771	1344.703128	1349.483991	671.499904	652.2426195	652.0675226
YTHDC1	1900.514904	2077.451529	1960.165243	3656.244773	3531.739034	3965.918276
EDN1	149.8578332	132.7732116	132.7974545	424.2002082	439.0314996	440.9879142
CALM1	5418.700457	5165.177421	5349.305912	2993.770405	3155.753834	3081.960479
CASP4	1533.313591	1496.443942	1486.022219	2654.410239	3023.929754	2924.394011
GJB3	2230.00365	1911.734588	1749.746741	3982.066635	4522.139075	4060.061763
OSBP2	2220.07929	2082.443003	2119.148111	3732.961832	3767.876081	3937.179738
JAG1	5183.49313	5773.138969	5422.250992	10290.01399	9265.513345	10544.07058
SH2D3A	2318.330452	2111.393553	1871.321876	4198.679507	4680.327971	4120.511792
ZMYND19	1313.985239	1241.878761	1072.666762	507.2351425	506.6629838	545.0412423
THBS1	5560.618803	6257.311958	5267.944091	2777.157533	2254.764908	2850.070205
TET2	409.8760602	548.0638585	618.1627986	1493.726265	1330.850055	1684.672931

MAD2L1	1845.930925	1774.968198	1862.905136	974.7579252	898.6963333	842.3364654
RTL10	835.6310962	798.6358594	802.3958868	364.6316683	310.6463091	330.9886817
DUSP5	3150.98424	2921.010656	2077.064411	6712.291379	7193.009556	6576.170334
COX19	465.4524752	463.2087985	454.5039638	1010.860071	1100.44449	975.1283317
GPBP1	1370.55409	1359.677551	1443.003326	2522.637408	2808.426042	2590.432377
DOC2B	143.9032173	131.7749168	124.3807144	426.9078691	486.0296497	404.3215034
TSC22D1	3043.801154	3031.821381	2968.303665	5295.282173	5556.098377	5038.163047
SPAG5	2401.695075	2389.917809	2354.816833	1250.939337	1135.979676	1312.062918
HDAC1	5831.553826	5540.536275	5541.95574	3245.582869	3318.527914	3020.519466
EVA1C	331.4736177	301.4850369	285.2339691	67.69152258	73.36296599	80.26971023
UNC13D	2543.61342	2397.904168	2127.564851	4596.70566	5057.459468	4421.770951
DDX52	1142.293814	1256.853184	1316.752224	2400.792668	2345.322319	2448.721654

KPNB1	8236.226208	8194.003917	8792.687794	13747.69696	14261.07281	14594.2225
SRM	2007.69799	1832.869297	1704.857461	900.7485271	890.6722589	914.678303
MAP3K6	672.8715953	639.9069823	619.0979919	243.6894813	226.966676	264.5927485
USP25	762.1908336	825.5898197	796.7847267	1518.095213	1697.664885	1639.087663
MRPS34	2979.292816	2510.711483	2233.241699	1146.243116	1164.637085	1021.704583
TUFT1	600.4237686	525.1030776	460.1151239	1283.431268	1305.631535	1182.244004
EPHA2	7650.688979	7553.09864	6430.389414	13203.45712	13515.98019	13362.42929
WDR90	1642.481549	1602.263193	1485.087026	779.8063401	644.2185451	750.1749462
CPEB4	190.5477084	247.5771164	269.3356823	703.0892812	714.142622	644.13965
RCOR1	1067.861116	1151.033932	1219.492117	2180.569581	2206.620461	2245.569918
TGFB2	245.1316874	271.5361922	255.3077822	731.0684439	615.5611365	806.6610386
PLEKHG2	719.5160864	712.7825045	609.7460585	1423.327081	1503.940803	1430.981007

MOB3A	1663.322705	1559.336515	1340.132058	3000.990834	3215.361244	3058.176861
TXNL4B	385.0651607	425.2735951	402.1331367	929.6302434	967.4741139	874.0479558
PRAG1	618.2876163	667.8592374	544.2825246	1293.359358	1399.627835	1343.774408
CD24	611.3405644	631.9206238	712.617326	247.2996958	243.0148248	254.6829078
LRCH3	646.0758238	725.7603372	721.0340661	1411.593884	1416.822281	1457.737577
CCNB1IP1	844.56302	737.7398751	772.4696999	1522.607981	1729.761182	1716.384421
MEF2D	2012.66017	1764.985249	1646.875474	3534.400032	3534.031627	3517.002489
PIF1	595.4615887	626.9291496	570.4679381	240.9818204	181.1148223	189.2779587
PRXL2A	633.174156	530.0945517	561.1160047	192.2439241	201.7481565	217.0255128
ZBTB38	537.9003018	650.8882254	638.7370521	1407.98367	1268.950052	1476.566275
CCNJL	250.0938673	249.5737061	277.7524223	626.3722223	682.0463244	665.9412997
INTS3	1043.050216	967.3476847	1008.138422	1942.295421	1859.292669	2066.2018

ADRB2	1066.868<br>68	1028.243<br>669	911.8135<br>077	1909.803<br>49	2120.648<br>236	2141.516<br>59
AKIRIN1	1673.247<br>064	1767.980<br>134	1843.266<br>076	3189.624<br>544	3241.726<br>059	3110.699<br>017
AHR	721.5009<br>583	799.6341<br>542	912.7487<br>01	1972.079<br>691	2051.870<br>455	1661.880<br>297
SLC1A5	7138.592<br>013	6299.240<br>341	6301.332<br>733	11991.32<br>759	12429.29<br>125	11270.46<br>191
AL162171.<br>1	140.9259<br>093	116.8004<br>944	112.2232<br>009	407.0516<br>891	414.9592<br>764	392.4296<br>945
GADD45B	622.2573<br>602	635.9138<br>031	421.7721<br>969	1513.582<br>445	1640.350<br>068	1446.836<br>752
GPR87	1197.870<br>229	999.2931<br>191	1065.185<br>216	2165.226<br>169	2266.227<br>871	2121.696<br>909
PATL1	1969.985<br>423	1993.594<br>764	2051.814<br>191	3417.068<br>06	3851.555<br>714	3918.351<br>04
PGF	193.5250<br>163	143.7544<br>547	134.6678<br>411	562.2909<br>142	724.4592<br>891	488.5551<br>499
NASP	4226.784<br>844	4065.056<br>524	4049.387<br>168	2365.593<br>076	2214.644<br>536	2420.974<br>1
DCBLD2	2068.236<br>585	2367.955<br>323	2538.114<br>728	4424.317<br>916	4594.355<br>745	4431.680<br>792
NOL6	3168.848<br>088	3105.695<br>198	3157.212<br>72	1722.974<br>888	1462.674<br>134	1723.321<br>31

TAOK1	1287.189468	1768.978429	1919.95193	4643.638449	3718.585338	4641.769416
DGCR6L	586.5296649	565.0348705	596.6533517	230.1511768	231.5518614	201.1697676
PCYOX1	787.9941691	948.380083	926.7766012	374.5597583	340.450014	378.5559174
PLEKHA6	62.52346682	46.91985674	48.63005374	241.884374	272.8185298	241.8001148
CCNA2	1817.150282	1823.884644	1888.155356	973.8553715	972.0592993	1005.848838
ITSN2	610.3481284	756.7074768	785.5624066	1675.139545	1509.672284	1748.095912
FBXO28	795.9336569	876.5028557	936.1285346	1791.568964	1741.224146	1701.51966
IZUMO1	78.40244252	76.86870147	67.33392057	313.1861111	278.5500115	290.3583345
FBXL3	700.6598027	835.5727679	757.5066064	1555.099912	1652.959327	1538.998271
TK1	6057.829229	5080.322361	4801.282614	2741.957941	2631.896405	2548.811046
PIGA	344.3752855	297.4918576	343.2159562	757.2424993	833.3574417	777.9225004
SH3BGRL3	3694.839158	3072.751469	2946.794218	1473.870085	1630.0334	1559.808937

WDFY3	604.3935126	721.7671579	705.1357793	1540.659054	1361.800056	1633.141759
LIPG	107.183086	117.7987893	122.5103277	412.4670109	378.2777934	358.7362358
PON2	1763.558739	1800.923863	1821.756629	918.7995998	959.4500395	978.1012839
FABP5	2041.440813	1886.777218	1754.422708	907.9689562	996.1315225	897.8315737
MLEC	4479.856019	4654.050471	4700.281733	2745.568156	2417.538988	2585.477457
MCM10	908.0789228	865.5216126	1035.259029	361.9240074	295.7444566	405.3124875
LRRC49	56.56885093	66.88575323	66.39872723	260.8380003	272.8185298	267.5657008
PARP8	606.3783845	641.903572	712.617326	1314.118092	1414.529688	1532.061383
SMG7	2604.152015	2735.327818	2770.042677	4543.454996	4667.718711	4953.9294
TMEM129	1096.641759	975.3340433	1046.481349	481.9636408	491.7611314	468.7354684
BHLHE40	5643.983425	5222.080226	4150.388048	10186.22032	11100.73379	10547.04353
JUND	867.3890476	787.6546163	759.3769931	1691.385511	1944.118599	1589.538459

ANKRD12	421.785292	541.0757947	561.1160047	1252.744445	1141.711158	1319.99079
ARG2	249.1014313	246.5788216	239.4094954	617.3466859	701.5333622	590.6265098
CSRP1	3229.386683	3178.57072	2962.692505	5903.603323	6474.281748	5370.142713
CAMKK1	338.4206696	313.4645748	286.1691624	80.32727346	67.63148427	88.19758285
SLC35F6	662.9472355	645.8967513	677.079979	1332.169164	1493.624136	1298.189141
CBR1	1368.569218	1222.91116	1212.01057	611.0288105	605.2444694	530.1764811
WDR48	462.4751672	477.184926	502.1988242	1025.300929	991.5463371	1055.398042
DUSP3	1012.284701	1061.187398	975.4066549	1861.065594	2077.088974	1950.256663
DNAJC7	1340.781011	1252.860004	1324.233771	2323.173055	2600.946403	2426.920004
USP47	1412.236401	1517.408133	1517.818793	2674.266419	2611.263071	2737.09802
FKBP9	2199.238134	2221.205984	2169.648552	1189.56569	1103.883379	1237.739112
IL31RA	176.6536047	216.6299769	210.4185018	602.0032741	522.7111326	558.9150194

ATP8B2	97.25872616	101.8260721	103.8064609	328.5295229	361.0833482	341.8895065
XYLT2	1335.818831	1276.81908	1210.140184	631.7875441	569.7092827	632.2478411
BRPF3	426.7474719	422.2787107	443.2816437	945.8762089	964.0352249	893.8676374
PGP	803.8731448	682.8336598	662.1168856	285.2069485	246.4537139	286.3943982
FGFR3	1049.004832	1026.247079	996.9161017	500.9172671	478.0055752	482.6092455
NBL1	1016.254445	776.6733733	800.5255001	342.0678274	317.5240872	330.9886817
ESPL1	1207.794589	1230.897518	1204.529023	583.0496478	483.737057	596.5724143
TMEM201	894.1848191	806.622218	799.5903067	370.9495437	357.6444592	364.6821403
FUT3	493.2406827	439.2497227	393.7163967	1021.690714	1236.853755	1012.785727
UFM1	1407.274221	1430.556483	1469.188739	2551.519124	2737.355668	2518.090539
HSPA8	14179.9253	12946.88558	13689.36013	7113.025193	8122.65589	7914.989822
ENDOD1	576.6053051	600.9734842	628.4499253	254.5201249	206.3333418	203.1517358

GAL	815.7823766	722.7654528	607.8756718	277.9865194	248.7463065	239.8181466
IFIT2	682.7959551	726.7586321	831.3868803	302.3554675	286.5740859	308.1960479
CENPX	1163.13497	1015.265836	880.0169341	435.0308518	392.0333495	349.8173791
INCENP	1563.08667	1611.247846	1768.450608	839.37488	704.9722513	811.615959
JAK1	1676.224372	1779.959672	1914.340769	3152.619845	3333.429767	3444.660651
KDM6A	652.0304397	785.6580267	686.4319125	1507.264569	1423.700059	1645.033568
NOP56	4800.412841	4361.550087	4430.946051	2576.790626	2621.579738	2633.044692
FAM107B	1436.054865	1520.403017	1660.903374	2837.628627	2917.324194	2803.493954
FAM193A	346.3601574	372.3639694	368.4661764	800.5650737	804.7000331	913.6873189
CHAF1B	818.7596845	722.7654528	712.617326	312.2835575	283.1351968	325.0427772
PBK	870.3663555	889.4806884	913.6838944	431.4206372	380.570386	370.6280448
ACP7	91.30411027	78.86529112	79.491434	315.893772	367.9611263	284.4124301

MSL2	485.3011948	446.2377864	488.1709241	1021.690714	986.9611518	997.9209654
SNRPA1	1844.938489	1665.155767	1693.635141	929.6302434	858.5759613	884.9487807
CLMP	535.9154299	561.0416912	588.2366116	178.7056196	135.2629685	217.0255128
CCL20	181.6157846	165.7169408	198.2609883	466.620229	552.5148376	545.0412423
ATAD3B	1029.156113	1006.281183	999.7216818	507.2351425	449.3481667	466.7535002
KLHL29	287.8064346	240.5890526	273.0764556	758.1450529	719.8741037	619.3650481
LIF	708.5992906	705.7944407	657.4409189	1497.336479	1370.970427	1328.909647
LRRC45	477.361707	381.3486229	393.7163967	137.1881524	103.1666709	123.8730096
SQOR	1020.224189	802.6290387	748.154673	295.1350385	341.5963104	310.1780161
KMT5A	1427.122941	1361.67414	1363.511892	2385.449256	2529.87603	2515.117587
ARHGEF39	656.9926196	678.8404805	656.5057255	284.3043948	275.1111224	285.4034142
DDIT4	5604.285986	5229.068289	4271.963183	12077.97274	11423.98936	9551.104533

CNST	282.8442546	269.5396025	294.5859025	648.0335095	669.4370646	700.6257424
COBL	98.25116214	91.84312383	78.55624066	336.6525056	292.3055676	356.7542677
CD276	1404.296913	1289.796913	1258.770237	667.8896895	636.1944707	661.9773634
CHTF18	2151.601207	1981.615226	1820.821435	976.5630324	803.5537368	993.9570291
TTF2	1432.085121	1493.449057	1628.171607	723.8480148	648.8037304	787.8323411
TCFL5	914.0335387	929.4124814	953.8972081	446.764049	367.9611263	432.0690575
SCYL2	730.4328822	827.5864093	816.4237869	1544.269268	1508.525988	1607.376173
CALCOCO2	1621.640393	1509.421774	1757.228288	2878.24354	3342.600138	3303.940912
RAB6A	2075.183637	2122.374796	2285.612526	3652.634558	4127.813133	3941.143674
RUSC2	217.3434799	251.5702957	203.8721484	624.567115	580.0259498	560.8969875
IGSF3	1526.366539	1563.329695	1674.931274	831.2518973	750.824105	839.3635131
SH3RF2	957.7007219	1015.265836	981.0178149	1870.993684	1769.881554	2034.49031

RASA1	679.8186471	791.6477956	779.9512466	1521.705428	1489.03895	1475.575291
ITCH	3707.740826	4204.8178	4224.268322	6930.709359	7007.309548	7384.813341
ARID3A	524.9986341	512.1252448	422.7073902	1129.094597	1207.05005	1026.659504
ARAP2	258.0333551	347.4065988	352.5678896	858.3285063	771.4574392	839.3635131
PDP1	3402.070544	3617.820443	3926.87684	6347.659711	6392.894708	6248.154605
NRM	728.4480102	686.8268391	673.3392057	302.3554675	296.890753	242.7910988
PSMB2	3737.513905	3728.631169	3486.400776	2126.416363	2108.038976	2165.300208
GTPBP3	775.0925013	807.6205128	644.3482121	314.0886648	270.5259371	285.4034142
GAS2L1	1401.319605	1170.001534	1076.407536	555.9730388	468.8352045	507.3838474
ECM1	242.1543794	272.534487	233.7983353	55.0557717	63.04629889	56.48609238
CD274	56.56885093	49.91474121	54.24121379	261.740554	239.5759358	217.0255128
KIF22	1758.596559	1620.2325	1710.468621	890.8204372	899.8426297	926.5701119

SLC2A4RG	3279.008482	2899.04817	2666.236216	1551.489698	1501.64821	1517.196622
STARD4	895.1772551	903.456816	1005.332842	1854.747719	2031.237121	1729.267214
TACC3	2313.368272	2163.304884	2184.611645	1256.354659	1033.959302	1081.163628
CLK4	150.8502691	162.7220564	158.982868	472.9381044	421.8370544	437.0239779
INO80D	333.4584897	395.3247504	450.7631905	934.1430116	925.0611492	948.3717616
BRI3BP	1154.203046	1177.987893	1268.122171	609.2237032	468.8352045	518.2846722
SERINC1	1490.638844	1564.32799	1663.708954	2797.013713	3028.51494	2765.836559
SFN	21170.64435	17722.72802	15897.35161	8577.869741	9610.548544	7965.53001
RPUSD1	994.4208532	922.4244176	815.4885935	390.8057237	414.9592764	348.8263951
RNF144B	535.9154299	621.9376755	609.7460585	1165.196742	1281.559312	1230.802224
TRIO	4102.730346	4737.907236	4480.511298	8501.152682	7489.900309	8961.469008
ARRDC3	812.8050686	907.4499952	788.3679867	1712.144244	1580.742658	1623.231918

AAK1	658.9774915	766.690425	822.9701403	1538.853947	1514.25747	1686.654899
FARP1	150.8502691	190.6743114	148.6957413	527.0913225	429.8611288	532.1584493
SMIM13	443.6188836	481.1781053	515.291531	1137.217579	1077.518563	965.2184909
PLK1	2760.9569	2603.552902	2536.244341	1428.742403	1323.972277	1521.160558
ARFGEF3	136.9561654	170.7084149	146.8253546	443.1538345	442.4703886	425.132169
CASC3	1813.180538	1980.616931	2014.406457	3350.279091	3477.863106	3342.589291
LIG1	1069.845988	978.3289278	982.8882016	483.768748	437.8852032	498.4649907
ASB13	626.2271041	543.0723844	582.6254516	244.5920349	192.5777857	219.9984651
ZBTB7B	1041.065344	879.4977402	878.1465474	2075.873359	1997.994527	1755.0328
MLX	1067.861116	1115.095319	1038.064609	1913.413705	1937.240821	1980.97717
CKAP4	6195.777831	5951.833742	6350.89798	3721.228635	3840.092751	3717.181272
AKR7A2	693.7127509	693.8149029	661.1816922	301.4529139	304.9148274	287.3853823

CCND3	1138.32407	1103.115781	1010.944002	548.7526097	458.5185374	484.5912136
CCM2	824.7143004	693.8149029	717.2932927	333.9448447	287.7203822	294.3222708
TONSL	1766.536047	1689.114843	1595.43984	850.2055236	708.4111403	880.9848444
RABGGTB	2169.465055	2189.26055	2122.888885	3701.372455	4010.890906	3574.479565
HOXB9	115.1225738	107.815841	142.1493879	367.3393292	409.2277946	385.4928059
FLRT1	29.77307944	26.95396025	23.37983353	157.0443324	181.1148223	176.3951657
UBAP1	1566.063978	1405.599113	1346.678411	2658.020453	2878.350119	2589.441393
TMEM109	1655.383217	1413.585471	1377.539792	724.7505684	737.0685489	693.6888538
TJAP1	992.4359812	974.3357485	877.2113541	1796.984286	1853.561187	1735.213119
METRN	1080.762784	889.4806884	842.6092004	413.3695646	302.6222347	344.8624588
CSNK2A1	3138.082573	3090.720776	3272.241501	5114.771446	5808.283573	5488.069818
EEF2K	1004.345213	1074.165231	1125.972783	539.7270734	471.1277972	436.0329938

AP2B1	5100.128507	5397.780115	5475.557013	8807.118365	8411.522569	8910.92882
TIMELESS	3089.45321	3155.609939	3238.574541	1853.845165	1820.318594	1902.689428
MGST1	1744.702455	1715.070508	1654.357021	882.6974545	967.4741139	889.9037011
PTTG1	2449.332002	2283.100263	2185.546838	1183.247815	1283.851905	1283.32438
UQCR10	1546.215259	1307.76622	1126.907976	526.1887689	593.7815059	564.8609238
ATF1	417.8155481	405.3076986	436.7352904	862.8412745	883.7944809	908.7323985
PTPA	3169.840524	2969.927102	2887.877038	1753.661712	1733.200071	1638.096679
WDR76	557.7490214	550.0604482	594.782965	212.1001041	151.3111173	224.9533855
PFKFB4	601.4162046	554.0536275	481.6245707	1130.899704	1271.242645	1161.433338
MCM7	20019.41861	17422.24127	16590.32987	9841.44483	10328.13006	10266.59504
PSMD10	1334.826395	1337.715065	1370.993438	723.8480148	715.2889184	651.0765385
RPS26	8079.421323	6938.149029	6378.953781	3297.93098	3658.977929	3870.783804

ZNF644	846.547892	1029.241964	1100.722563	2101.144861	1955.581562	2104.850179
NMRAL1	1599.806802	1398.611049	1439.262552	747.3144093	784.066699	728.3732965
MCFD2	1279.24998	1402.604228	1428.040232	2486.535263	2447.342693	2404.127371
SRD5A1	1194.892921	1228.900929	1264.381397	647.1309559	612.1222474	642.1576818
LRCH1	543.8549177	609.9581376	563.9215848	1140.827794	1126.809306	1153.505466
NADK2	1123.437531	1201.946968	1330.780125	613.7364714	582.3185425	562.8789557
WARS	1848.908233	1651.179639	1803.987955	3151.717291	3960.453867	3376.28275
REST	796.9260929	975.3340433	1048.351736	1987.423103	1903.998227	1968.094377
ESPN	467.4373472	505.1371811	436.7352904	175.9979587	128.3851905	169.4582772
SMURF2	1071.83086	1141.050984	1146.547036	1935.074992	2075.942678	2080.075577
HSP90B1	7392.655624	7121.835276	7102.793427	11730.48959	14671.4469	13037.38652
CEBPB	3690.869414	3460.089861	3308.714041	6967.714058	7578.165127	5874.553608

ABCA12	183.6006565	208.6436183	215.0944685	518.9683398	527.296318	506.3928633
YEATS2	1904.484648	1978.620342	1900.312869	3251.900745	3190.142724	3470.426237
GOSR2	727.4555742	682.8336598	762.1825731	1454.916459	1328.557462	1449.809705
FGD4	397.9668285	413.2940572	473.2078307	920.6047071	900.988926	943.4168412
RBBP4	2684.539329	2731.334639	2910.321678	1610.155684	1579.596361	1628.186838
CD59	6084.625001	5880.95481	5728.994408	3602.091555	3723.170524	3556.641852
YDJC	1445.979225	1338.713359	1295.242778	714.8224785	683.1926207	599.5453665
ULBP1	18.85628364	42.92667744	31.7965736	197.6592459	186.846304	193.241895
TST	672.8715953	609.9581376	582.6254516	244.5920349	272.8185298	254.6829078
PPM1E	67.48564672	67.88404805	97.26010749	326.7244157	268.2333444	313.1509683
GLS	1215.734077	1388.628101	1380.345372	2493.755692	2353.346393	2414.037211
DNAJB9	178.6384766	166.7152357	144.0197745	427.8104227	597.220395	482.6092455

NUP188	3849.659171	3942.266261	3887.598719	2210.353851	2140.135273	2427.910988
FAM210A	500.1877345	486.1695794	514.3563377	1024.398375	1271.242645	1027.650488
MFSD12	1190.923177	1148.039048	953.8972081	2227.50237	2200.88898	2078.093609
CHORDC1	1194.892921	1336.71677	1381.280565	2557.837	2271.959353	2562.684823
MAFG	1938.227471	1804.917042	1666.514534	3380.965914	3673.879781	3120.608858
SH2B3	435.6793958	489.1644639	541.4769446	1066.818396	998.4241152	1097.019373
ERVMER34-1	638.1363359	706.7927356	707.006166	308.673343	299.1833457	291.3493186
CSTA	1286.197032	1128.073151	1068.925989	2111.072951	2427.855656	2285.209281
GSR	2830.427418	2960.942449	2938.377478	1763.589802	1660.983402	1703.501628
NT5DC2	1237.567669	1073.166936	961.3787548	463.0100145	502.0777985	494.5010544
ATP13A2	1340.781011	1292.791797	1197.047477	618.2492396	569.7092827	679.8150768
PFDN6	1056.94432	909.4465849	796.7847267	370.0469901	382.8629787	275.4935734

TNRC18	4913.550543	5370.826155	5220.249231	9090.520206	8656.829986	8187.510443
KRT19	31749.01948	27972.22098	25278.27601	15439.08247	16199.45993	15298.81218
FNIP1	603.4010766	718.7722735	825.7757203	1475.675192	1660.983402	1613.322077
PEBP1	6660.23787	5984.777471	6059.117658	3820.509534	3689.92793	3578.443502
DESI2	705.6219826	760.7006561	814.5534002	1435.060279	1501.64821	1443.8638
MICALL1	3343.516821	3332.308123	3104.841893	5452.326505	5977.935431	5320.593509
ATXN2L	3388.17644	3363.255263	3164.694267	5390.050305	5376.129851	5337.440238
HMGB2	3972.721233	3547.939805	3602.36475	2126.416363	2055.309344	2207.912523
CDC42EP4	1151.225738	1101.119191	1121.296816	583.9522015	580.0259498	583.6896213
TCEA1	3006.088587	3075.746354	2894.423391	4946.89647	6192.292848	5505.907531
SERINC3	3544.981325	3440.123964	3574.30895	5566.950817	6140.709512	5747.707646
EPRS	5518.936492	5904.913885	6277.9529	9530.966379	9908.585593	9957.408005

RPA1	2458.263925	2372.946797	2319.279486	1407.081116	1260.925978	1359.630154
MLLT6	622.2573602	641.903572	672.4040123	1443.183261	1210.488939	1274.405523
OSMR	4673.381036	5348.863668	5260.462544	8667.222551	8389.742938	9151.73795
RPS6KA4	3451.692343	3206.522976	2977.655598	1840.306861	1774.46674	1609.358141
SQSTM1	4153.344581	3936.276492	3682.791378	7529.102418	9325.120755	6951.7533
SGPP2	133.9788575	139.7612754	157.1124813	387.1955092	403.4963129	428.1051212
KBTBD2	1107.558555	1200.948674	1114.750463	2036.160999	1999.140823	2022.598501
ZFYVE21	431.7096518	385.3418022	414.2906502	143.5060279	154.7500064	145.6746593
CCDC47	2126.790308	2196.248613	2336.112966	3757.33078	3732.340894	3680.514862
PUM2	1838.983873	2188.262255	2284.677333	3899.934254	3832.068676	4057.088811
NIPA1	749.2891658	865.5216126	779.0160532	1529.82841	1484.453765	1531.070399
ARHGDIB	1002.360341	904.4551108	899.6559943	403.4414746	419.5444617	475.6723569

UFD1	1511.479999	1460.505328	1364.447085	784.3191083	711.8500293	769.9946278
PTGS1	645.0833878	632.9189186	560.1808114	272.5711976	226.966676	204.1427198
ARMCX3	813.7975046	867.5182023	791.1735667	1602.935255	1724.029701	1468.638402
NLRP1	68.4780827	56.90280498	51.43563377	243.6894813	296.890753	219.007481
ZNRF3	518.0515822	501.1440018	514.3563377	212.1001041	137.5555612	179.3681179
SAP30L	254.0636112	294.4969732	283.3635824	674.2075649	605.2444694	660.9863793
ARID3B	436.6718317	345.4100092	348.8271163	796.0523055	981.2296701	978.1012839
TNFRSF9	51.60667102	54.90621533	76.68585398	259.0328931	255.6240846	238.8271625
OXLD1	618.2876163	565.0348705	540.5417512	211.1975505	248.7463065	238.8271625
TMEM184A	381.0954168	378.3537384	282.4283891	876.379579	886.0870735	781.8864367
ZNF518A	354.2996453	528.097962	540.5417512	1305.995109	1102.737082	1413.143294
CTTNBP2NL	528.968378	609.9581376	655.5705322	1211.226977	1195.587086	1292.243236

FOXA2	453.5432434	376.3571487	388.1052366	880.8923472	986.9611518	849.2733539
ZNF609	1363.607038	1528.389376	1586.087907	2843.043948	2567.703809	2995.744865
NUP93	2486.052133	2490.745587	2344.529706	1439.573047	1315.948202	1423.053134
MICALL2	1024.193933	1030.240259	915.5542811	469.3278899	380.570386	481.6182614
PROM2	928.9200784	884.4892143	890.3040608	440.4461736	338.1574213	422.1592168
PCNX2	462.4751672	471.195157	501.2636309	935.9481189	953.7185578	1006.839822
PROSER1	964.6477737	991.3067605	1018.425549	1762.687248	1787.076	2045.391135
IL6	6.947051869	11.97953789	17.76867348	133.5779379	132.9703758	122.8820255
BRAT1	1419.183453	1236.887287	1247.547917	676.0126722	663.7055829	652.0675226
FGFR1OP2	778.0698093	859.5318437	869.7298073	1556.002466	1526.86673	1552.872049
TNFAIP2	2478.112645	2731.334639	2845.793337	5173.437432	4493.481667	4729.966999
TXNRD1	4011.426236	4173.87066	4547.845218	7051.651546	7350.052155	6897.249175

TLN2	57.56128691	59.89768946	71.07469393	239.1767131	237.2833431	250.7189715
EIF4G3	1503.540512	1690.113137	1682.412821	2878.24354	2762.574188	3075.023591
TMEM39A	450.5659355	447.2360813	468.531864	895.3332053	917.0370748	929.5430641
TSPAN3	1943.189651	1805.915337	1806.793535	1044.254555	998.4241152	1058.370994
GINS2	1116.490479	996.2982346	954.8324014	479.2559799	498.6389094	507.3838474
MSRB1	596.4540247	497.1508225	553.634458	231.0537304	182.2611186	214.0525606
ATP2C2	869.3739195	824.5915248	708.8765526	1533.438625	1722.883404	1562.781889
USP32	1192.908049	1345.701423	1313.011451	2238.333013	2257.0575	2330.794549
PPDPF	5117.992355	4587.164717	4274.768763	2497.365906	2666.285295	2205.930555
PEAR1	200.4720682	236.5958733	190.7794416	494.5993917	550.2222449	571.7978124
NECTIN4	484.3087588	426.27189	419.9018102	999.1268733	990.4000408	868.1020514
PGPEP1	443.6188836	462.2105036	442.3464504	950.388977	902.1352223	878.0118921

FHOD1	680.8110831	690.8200184	633.125892	309.5758966	286.5740859	301.2591594
AC092338.1	743.3345499	654.8814047	711.6821327	313.1861111	318.6703835	322.069825
NUP58	1471.78256	1764.985249	1851.682816	3231.142011	3199.313095	3133.491651
DAB2IP	2260.769165	2087.434478	2168.713358	1197.688673	1215.074124	999.9029336
EPC1	238.1846355	258.5583595	243.1502687	540.629627	624.7315072	594.5904461
C3	1906.46952	1748.014237	1645.005087	3019.944461	3095.000127	3164.212158
ZNF12	656.0001836	814.6085766	789.30318	1474.772639	1523.427841	1492.42202
DCP1A	616.3027443	664.864353	662.1168856	1297.872126	1165.783381	1338.819488
HNRNPAB	5267.850188	5092.301899	4807.828967	3017.2368	2604.385292	2319.893724
SEMA3C	1318.947419	1608.252962	1682.412821	3022.652122	2864.594562	2951.150581
RBPJ	882.2755873	1085.146474	1028.712675	1926.952009	1847.829706	1954.2206
FLAD1	1303.068443	1246.870235	1176.473223	645.3258486	659.1203975	559.9060035

NABP1	1414.221273	1509.421774	1560.837687	2527.150176	2613.555663	2550.793014
SELENOH	1417.198581	1306.767925	1215.751344	695.8688521	675.1685463	634.2298092
TEP1	1369.561654	1449.524085	1366.317472	760.8527138	777.1889209	738.2831373
AHSA1	2428.490846	2072.460055	2159.361425	1204.909102	1233.414866	1076.208708
RAD54L	529.960814	560.0433964	530.2546245	194.951585	158.1888954	237.8361785
SGPL1	1002.360341	901.4602263	965.1195281	1657.088473	1836.366742	1770.888545
WDR62	624.2422322	577.0144084	614.4220252	228.3460695	200.6018601	281.4394778
SPECC1	1188.938305	1291.793503	1204.529023	2047.894196	2289.153798	2273.317472
RASEF	421.785292	507.1337707	519.9674977	1070.42861	951.4259651	1114.857087
RECQL4	1513.464871	1523.397902	1380.345372	783.4165547	590.3426169	723.4183761
GBA2	1365.59191	1228.900929	1250.353497	2228.404923	2200.88898	2263.407632
RAD51	562.7112013	490.1627587	531.1898178	233.7613913	201.7481565	200.1787835

RBM18	621.2649242	660.8711737	643.4130188	1191.370797	1193.294494	1201.072701
CGGBP1	707.6068546	749.719413	804.2662735	1383.614722	1387.018576	1494.403988
NBR1	2170.457491	2238.176996	2104.185018	3465.805956	3912.30942	3671.596005
POLE2	402.9290084	457.2190295	452.6335772	166.0698687	163.9203771	173.4222135
SUPT20H	838.6084041	924.4210073	1031.518255	1702.216154	1816.879704	1852.14924
DMRT2	318.57195	286.5106146	274.011649	90.25536344	92.85000382	87.20659877
ABI2	761.1983976	826.5881145	851.0259405	1476.577746	1463.820431	1601.430268
LSM3	1032.13342	938.3971348	887.4984808	426.0053154	478.0055752	469.7264525
ADGRG1	3671.020695	3526.975614	3330.223488	5692.405772	5948.131727	5557.438703
NUPR1	131.9939855	124.786853	106.6120409	416.9797791	588.0500242	337.9255702
MED13	1616.678213	2007.570892	2203.315512	4143.623736	3611.979778	4226.547088
TNIP1	5236.092237	4816.772527	4996.738022	8112.152066	9224.246676	8075.529243

HBP1	503.1650<br>425	571.0246<br>395	568.5975<br>515	1033.423<br>911	1117.638<br>935	1095.037<br>405
UROD	1387.425<br>502	1141.050<br>984	1042.740<br>575	501.8198<br>207	581.1722<br>462	501.4379<br>429
PEA15	1901.507<br>34	1886.777<br>218	1748.811<br>548	3162.547<br>935	3806.850<br>157	3210.788<br>409
SYPL1	3069.604<br>49	3143.630<br>402	3245.120<br>894	1876.409<br>006	1964.751<br>933	1763.951<br>657
SNRPG	1963.038<br>371	1773.969<br>903	1435.521<br>779	828.5442<br>364	792.0907<br>734	814.5889<br>112
GMNN	969.6099<br>536	963.3545<br>054	931.4525<br>679	491.8917<br>308	402.3500<br>166	480.6272<br>773
SF1	3999.517<br>004	3636.788<br>045	3680.920<br>991	6014.617<br>42	6040.981<br>73	6256.082<br>478
KLF12	275.8972<br>028	313.4645<br>748	322.6417<br>027	701.2841<br>739	632.7555<br>816	743.2380<br>577
AKAP10	654.0153<br>116	787.6546<br>163	895.9152<br>209	1569.540<br>77	1634.618<br>586	1701.519<br>66
FYB1	205.4342<br>481	165.7169<br>408	257.1781<br>688	36.10214<br>538	46.99815<br>008	39.63936<br>308
HPS6	523.0137<br>621	484.1729<br>898	443.2816<br>437	195.8541<br>387	198.3092<br>674	167.4763<br>09
DMTF1	1824.097<br>333	2115.386<br>733	2111.666<br>564	3537.107<br>693	3404.500<br>14	3656.731<br>244

DCTPP1	1426.130505	1219.916275	1187.695543	671.499904	606.3907657	558.9150194
ZBTB43	729.4404462	770.6836043	743.4787063	1409.788777	1530.305619	1312.062918
FBXO42	577.5977411	582.0058825	612.5516385	1111.043524	1158.905603	1088.100516
MYBBP1A	2713.319973	2464.789921	2544.661081	1550.587144	1502.794506	1450.800689
PRXL2B	612.3330004	576.0161136	490.0413108	242.7869277	208.6259345	207.1156721
TLK2	1071.83086	1175.991303	1145.611843	1893.557525	2116.06305	2067.192784
PCSK9	1456.89602	1414.583766	1201.723443	723.8480148	628.1703962	627.2929207
AKR1C3	582.559921	529.0962569	466.6614773	184.1209414	229.2592687	182.3410702
FNTA	720.5085224	782.6631422	720.0988727	1321.338521	1459.235245	1367.558026
VPS26C	746.3118579	757.7057716	743.4787063	1366.466202	1397.335243	1311.071934
IFI16	4238.694076	4290.671155	4639.494166	7094.97412	9131.396672	8127.060415
DPF2	1634.542061	1445.530906	1551.485753	2623.723415	2620.433441	2671.693071

NDUFB10	1895.552724	1537.374029	1630.041994	908.8715098	842.5278125	803.6880864
C1orf116	2510.863032	2395.907578	2216.408219	3921.595541	4211.492766	3921.323992
KRT5	20627.78187	18163.97433	17362.79957	11513.87671	10845.10971	10930.55437
SLC45A1	49.62179906	37.93520332	53.30602045	196.7566923	225.8203797	196.2148472
CD9	5811.705106	5495.613008	5121.118737	3149.00963	3370.11125	3344.57126
ZNF440	370.178621	383.3452125	438.605677	131.7728306	110.044449	156.5754842
CEP120	535.9154299	665.8626478	682.6911391	1276.210839	1248.316718	1427.017071
TEDC2	503.1650425	427.2701848	424.5777769	182.3158342	153.60371	172.4312294
RNF24	334.4509257	359.3861367	308.6138026	747.3144093	680.900028	742.2470736
C11orf96	6.947051869	10.98124307	7.48154673	119.1370797	137.5555612	90.179551
MUS81	1049.997268	910.4448797	800.5255001	1881.824328	1987.67786	1730.258198
PASK	415.8306761	406.3059935	402.1331367	138.0907061	131.8240795	173.4222135

NCOA6	1639.504241	1963.645919	1827.367789	3292.515658	3190.142724	3879.702661
KAT6A	871.3587915	1000.291414	1010.944002	1726.585103	1745.809331	1929.445998
CLSTN1	10437.44921	9949.006219	9549.259207	6354.88014	6200.316922	6481.035863
MGLL	243.1468154	244.5822319	221.6408219	554.1679315	700.3870659	538.1043538
EML3	1531.328719	1457.510443	1275.603717	742.8016411	747.385216	726.3913284
KDM4A	424.7626	434.2582486	432.0593236	839.37488	851.6981832	858.1922106
ZNF770	580.575049	763.6955406	802.3958868	1637.232293	1423.700059	1676.745058
IL11	247.1165593	230.6061044	215.0944685	607.418596	632.7555816	494.5010544
EVPL	1302.076007	1252.860004	1428.975425	716.6275857	520.41854	584.6806054
CROT	453.5432434	513.1235397	563.9215848	187.731156	217.7963053	196.2148472
TNFAIP8L1	458.5054233	493.1576432	476.0134107	189.5362632	210.9185272	182.3410702
MKX	38.70500327	32.9437292	30.86138026	174.1928514	157.0425991	183.3320542

FNBP1L	870.3663555	909.4465849	963.2491415	1642.647615	1577.303769	1724.312294
C21orf58	248.1089953	236.5958733	280.5580024	78.52216619	71.0703733	63.42298092
ATP11A	1058.929192	1282.808849	1266.251784	2702.245581	2106.892679	2630.07174
NUBP2	1406.281785	1204.941853	1109.139303	617.3466859	612.1222474	548.0141945
DUSP16	420.792856	582.0058825	532.1250112	1083.966915	1212.781531	1103.956262
NRN1	126.0393696	154.7356978	140.2790012	404.3440282	450.494463	352.7903314
HR	532.9381219	568.029755	459.1799305	197.6592459	208.6259345	219.007481
TNKS2	1183.976126	1503.432005	1514.078019	2694.122599	2612.409367	2753.94475
AGAP1	794.941221	919.4295331	933.3229545	1725.682549	1569.279694	1664.853249
ZC3H12C	325.5190018	400.3162245	464.7910906	969.3426034	859.7222576	941.4348731
WDR41	866.3966116	837.5693576	915.5542811	1483.798175	1722.883404	1685.663915
SLC12A8	151.8427051	160.7254667	162.7236414	404.3440282	396.6185349	415.2223282

CST3	4052.116111	3567.905702	3332.093875	1975.689906	2082.820456	2087.012466
DOK7	250.0938673	222.6197458	180.4923149	575.8292188	598.3666913	509.3658155
APMAP	1734.778095	1651.179639	1543.069013	933.240458	923.9148529	874.0479558
RLF	804.8655808	917.4329435	963.2491415	1632.719525	1643.788957	1696.56474
TNFSF18	4.962179906	12.97783272	14.96309346	122.7472943	119.2148197	111.9812007
HS6ST1	1088.702271	1036.230028	936.1285346	453.0819245	249.8926029	384.5018218
LMNB2	10801.67322	10369.28834	9837.298756	6787.203331	6123.515067	6203.560321
C5orf51	1109.543427	1366.665614	1393.438078	2409.818204	2431.294545	2407.100323
RFX3	135.9637294	206.6470286	189.8442483	496.4044989	464.2500191	523.2395926
MT2A	8141.94479	6833.328072	5691.586675	3145.399416	3597.077926	3345.562244
ZNF827	315.594642	314.4628696	345.0863429	685.0382085	667.1444719	715.4905035
TUBE1	389.0349046	464.2070933	460.1151239	932.3379043	895.2574443	873.0569718

FER	444.6113196	621.9376755	555.5048447	1153.463545	1156.613011	1196.117781
SULT1E1	14.88653972	4.991474121	12.15751344	111.014097	116.922227	119.9090733
DYRK1A	851.5100719	963.3545054	921.1654411	1620.083774	1630.0334	1618.276998
ARHGAP21	2448.339566	2937.981668	3102.036313	5052.495245	5003.583539	5422.664869
SOS1	460.4902953	545.068974	560.1808114	1074.941379	1001.863004	1189.180892
NETO2	1075.800604	1145.044163	1190.501123	607.418596	588.0500242	618.374064
IGFBP6	855.4798158	669.8558271	623.7739586	268.0584294	318.6703835	253.6919237
PTMA	18989.27006	17765.65469	18933.92439	11589.69122	12484.31348	11811.53921
TTLL12	3861.568403	3414.168299	3241.380121	2054.212072	2004.872305	1988.905042
LSM4	3489.40491	3034.816266	2903.775325	1791.568964	1793.953778	1617.286014
FAM3C	3719.650058	3845.431663	3791.273805	2227.50237	2451.927879	2139.534622
ARHGEF7	582.559921	606.9632531	589.171805	1107.433309	1117.638935	1067.289851

P3H3	635.159028	548.0638585	535.8657845	259.9354467	241.8685285	224.9533855
CXCL3	116.1150098	72.87552217	104.7416542	375.4623119	300.329642	321.0788409
LY6E	3461.616702	2899.04817	2819.607924	1566.833109	1719.444515	1690.618835
ARHGAP12	510.1120943	580.0092929	584.4958383	1068.623503	1090.127823	1055.398042
SELENOW	1872.726697	1784.951146	1654.357021	1017.177946	973.2055956	862.1561469
CCND2	431.7096518	367.3724953	383.4292699	794.2471983	1030.520413	847.2913858
CPEB2	100.2360341	93.83971348	115.9639743	342.9703811	335.8648287	280.4484938
CARD11	221.3132238	175.6998891	178.6219282	472.9381044	482.5907606	475.6723569
PPP1R13L	4773.61707	4640.074343	4187.795782	7257.433774	7595.359572	7245.084586
IMPA2	699.6673668	692.816608	659.3113056	333.0422911	335.8648287	313.1509683
ENTPD3	264.980407	266.5447181	313.2897693	96.57323888	67.63148427	81.26069431
SIPA1L1	569.6582532	597.9785997	587.3014183	1055.987752	1080.957452	1122.784959

CWC25	481.3314509	444.2411968	443.2816437	853.8157381	957.1574468	905.7594463
SLC37A4	673.8640312	619.9410859	641.5426321	297.8426994	315.2314945	255.6738918
PLEKHG1	109.1679579	113.80561	89.77856076	303.2580212	335.8648287	302.2501435
PPARGC1B	541.8700457	600.9734842	632.1906987	265.3507685	177.6759332	236.8451944
CA9	1162.142534	1045.214681	833.257267	476.548319	428.7148325	416.2133123
FBXO25	265.972843	239.5907578	239.4094954	529.7989834	569.7092827	580.7166691
MZF1	532.9381219	547.0655637	552.6992647	1057.79286	984.6685591	1021.704583
PTGER4	472.3995271	577.0144084	435.800097	1046.059662	1050.007451	1017.740647
RASAL2	1049.997268	1229.899223	1288.696424	2273.532605	2126.379717	2656.82831
PHF21A	665.9245434	808.6188076	819.2293669	1463.039441	1417.968577	1568.727794
DONSON	505.1499144	492.1593484	476.0134107	203.9771214	223.527787	207.1156721
SGK1	1413.228837	1300.778156	1515.948406	700.3816203	667.1444719	800.7151341

CNOT2	829.6764803	808.6188076	802.3958868	1392.640258	1459.235245	1426.026087
DHTKD1	1129.392147	1213.926506	1238.195984	656.1564922	662.5592866	607.4732391
OSGIN2	581.567485	635.9138031	615.3572185	1124.581828	1236.853755	1101.974294
SRSF1	810.8201967	892.4755729	997.8512951	1748.24639	1665.568587	1628.186838
TSEN34	2250.844805	2120.378207	1937.720603	1235.595926	1116.492639	1182.244004
SFXN2	288.7988705	290.5037939	302.0674492	86.6451489	55.02222449	102.0713599
WNT7B	998.3905971	885.4875091	868.794614	469.3278899	460.8111301	457.8346435
NCAPD2	6065.768717	6086.603543	6046.960144	3771.771638	3637.198298	4066.007668
PCDH1	1883.643492	1907.741409	1760.969062	3014.529139	3257.774208	2967.99731
PHGDH	6240.43745	5284.9728	5188.452657	9557.140435	10341.88561	9181.467473
TMEM41B	851.5100719	887.4840988	1017.490355	1676.944653	1900.559338	1651.970456
FHL2	2267.716217	2093.424246	1876.933036	3464.903402	3707.122375	3649.794355

BMP2	241.1619434	241.5873475	279.622809	563.1934679	621.2926182	550.9871468
CDK13	718.5236504	833.5761783	944.5452746	1541.561608	1683.909329	1750.07788
PPM1F	1055.951884	1004.284593	1020.295935	548.7526097	503.2240948	569.8158442
CDCA8	1386.433066	1211.929917	1380.345372	721.1403539	641.9259524	727.3823125
MAPK12	657.9850555	629.9240341	606.9404785	310.4784502	279.6963078	296.304239
HJURP	982.5116214	917.4329435	922.1006344	482.8661944	402.3500166	498.4649907
NFATC2IP	1684.16386	1720.061982	1714.209394	1037.034126	943.4018907	990.9840769
BRD1	664.9321074	682.8336598	591.0421917	1217.544853	1179.538937	1208.00959
STX3	780.0546812	766.690425	792.10876	1366.466202	1581.888954	1368.54901
SHB	1029.156113	965.3510951	896.8504142	1870.993684	1772.174147	1628.186838
TOGARAM1	173.6762967	171.7067098	171.1403814	444.0563881	390.8870531	437.0239779
HIPK1	891.2075111	1062.185693	1170.862063	2042.478875	1939.533413	1992.868979

OTULINL	413.8458042	432.2616589	443.2816437	181.4132805	166.2129698	182.3410702
CDC73	1117.482915	1238.883877	1260.640624	2209.451297	1973.922304	2215.840396
RNF41	1787.377202	1666.154062	1731.042875	2850.264377	3107.609387	2751.962782
MRPS12	1051.98214	991.3067605	903.3967676	517.1632325	480.2981679	507.3838474
SMIM15	1016.254445	1037.228322	1113.815269	1804.204715	1894.827856	1765.933625
DDX24	3224.424503	3066.7617	3057.147032	1859.260487	1853.561187	2011.697676
YARS	4396.491397	4153.904764	4040.035234	6669.871358	7612.554017	6613.827729
FEM1C	603.4010766	684.8302494	629.3851186	1168.806957	1357.214871	1174.316131
ECI2	633.174156	607.961548	595.7181584	267.1558758	310.6463091	265.5837326
SLC16A14	171.6914248	148.7459288	206.6777284	43.32257445	25.21851956	26.75657008
FANCD2	1107.558555	1139.054394	1255.029464	642.6181877	580.0259498	629.2748888
TXNDC17	1228.635745	1041.221502	862.2482606	484.6713017	447.055574	371.6190288

DIS3	1300.091135	1537.374029	1553.35614	2602.062128	2498.926029	2639.981581
SNRPC	2037.471069	1808.910222	1860.099556	992.8089978	1149.735233	1022.695567
GCNT1	587.5221009	675.845596	654.6353389	319.5039866	262.5018627	258.6468441
EME1	552.7868415	553.0553326	523.7082711	263.5456612	231.5518614	214.0525606
CPNE8	846.547892	853.5420747	905.2671543	1468.454763	1563.548213	1510.259733
ANGPT4	14.88653972	17.96930684	10.28712675	138.0907061	119.2148197	102.0713599
RAB5A	829.6764803	803.6273335	883.7577075	1435.962832	1673.592662	1504.313829
VEZT	679.8186471	763.6955406	800.5255001	1308.70277	1444.333393	1432.962975
PPT1	3186.711936	3268.417255	3290.010174	1819.548127	1964.751933	2092.95837
FAM83D	1925.325804	1774.968198	1706.727848	935.0455652	797.8222551	1043.506233
KREMEN2	750.2816018	657.8762892	619.0979919	333.0422911	285.4277895	282.4304619
ARL6IP1	2285.580065	2245.16506	2413.734014	1383.614722	1302.192646	1439.899864

AKT3	418.8079841	505.1371811	440.4760637	904.3587417	879.2092955	934.4979845
OAF	643.0985158	577.0144084	535.8657845	266.2533222	265.9407517	251.7099555
SHOC2	455.5281154	475.1883363	526.5138511	925.1174753	939.9630017	943.4168412
SCARB1	3000.133971	2753.297125	2626.022902	1713.046798	1623.155622	1642.060615
BPTF	1521.404359	1687.118253	1702.051881	2925.176329	2634.188997	3180.067903
MAZ	789.979041	730.7518114	795.8495334	400.7338137	356.4981628	301.2591594
LARP1	5978.434351	6012.729726	6311.61986	9449.736552	9012.181853	9849.39074
MAPT	119.0923177	115.8021996	97.26010749	299.6478066	319.8166798	327.0247454
SHMT1	811.8126326	775.6750784	812.6830135	432.3231909	393.1796458	377.5649333
FEM1B	1012.284701	1122.083382	1219.492117	2059.627394	1897.120449	2081.066562
NKX2-8	141.9183453	158.7288771	125.3159077	24.36894813	19.48703784	27.74755415
CCDC50	660.9623635	792.6460905	735.9971595	1327.656396	1393.896354	1326.927679

HPGD	166.7292448	177.6964787	171.1403814	39.71235991	12.60925978	32.70247454
PRPS1	2249.852369	2126.367976	2266.908659	1372.784078	1334.288944	1319.99079
ABHD5	252.0787392	248.5754112	271.206069	595.6853987	589.1963206	523.2395926
TRAF1	53.59154299	35.93861367	46.75966706	173.2902978	265.9407517	192.2509109
SH3BP1	845.555456	727.7569269	651.8297588	310.4784502	351.9129775	335.9436021
RASIP1	66.49321074	79.86358594	52.37082711	238.2741595	241.8685285	227.9263377
NMNAT2	157.797321	174.7015942	166.4644147	380.8776337	448.2018703	416.2133123
RCN2	1528.351411	1559.336515	1614.143707	908.8715098	930.7926309	919.6332234
ABHD17C	2013.652606	1987.604995	1975.128337	1223.862728	1141.711158	1225.847303
MMP15	672.8715953	673.8490064	577.0142915	312.2835575	271.6722334	291.3493186
RNF6	942.8141822	1010.274362	994.1105217	1651.673151	1702.25007	1648.997504
ARL14	122.0696257	128.7800323	198.2609883	400.7338137	488.3222423	484.5912136

MXI1	777.0773733	814.6085766	852.8963272	1403.470902	1468.405616	1409.179357
CLIP1	2636.902402	2940.976552	3082.397253	4558.798407	4973.779835	4956.902353
SPEN	2836.382034	3340.294482	3165.62946	5556.120173	5000.14465	6200.587369
YBX1	19213.5606	17888.44496	17879.96149	11672.72615	11918.04308	10181.37041
CYP2R1	404.9138803	389.3349815	430.188937	797.8574128	817.3092929	776.9315163
TCF19	657.9850555	589.9922411	687.3671058	315.893772	224.6740833	273.5116052
FBLIM1	1648.436165	1369.660499	1306.465098	2617.40554	2860.009377	2535.928253
PHB	3158.923728	2801.215277	2723.28301	1727.487656	1683.909329	1733.231151
IRAK2	189.5552724	189.6760166	207.6129218	425.1027618	508.9555765	470.7174365
ZNF641	198.4871962	172.7050046	202.0017617	435.0308518	502.0777985	445.9428346
PLEKHH1	375.1408009	408.3025831	414.2906502	861.9387209	746.2389196	800.7151341
TRIM44	2410.626998	2690.404551	2701.773563	4113.839466	4167.933505	4364.293875

TRIM28	16293.81394	14762.78386	13994.23316	9451.54166	9636.91336	9019.937068
YTHDF3	1215.734077	1356.682666	1466.383159	2411.623311	2258.203797	2401.154418
FOXM1	1945.174523	1897.758461	1910.599996	1162.489081	930.7926309	1083.145596
RNF19B	1128.399711	1110.103845	1069.861182	1847.52729	2180.255645	1863.050065
EGR1	1761.573867	1759.993775	929.5821812	9315.256061	7936.955883	9394.529049
PTGFRN	2391.770715	2394.909283	2439.919427	1456.721566	1351.483389	1538.007287
ACOT7	2570.409191	2318.040582	2070.518057	1318.63086	1221.951902	1311.071934
CLEC2L	638.1363359	514.1218345	496.5876642	191.3413705	245.3074175	224.9533855
ACTG1	60633.86871	53797.10978	47057.99374	89798.6713	99017.07815	92090.1683
WNT9A	566.6809453	595.9820101	513.4211443	265.3507685	251.0388992	240.8091307
SMG5	3233.356427	3144.628696	3191.814874	4937.870934	4798.396494	4828.074423
TGIF1	898.154563	874.506266	877.2113541	1560.515234	1670.153773	1443.8638

DDR1	11756.39663	11133.98217	10434.8873	7288.120598	7129.963257	6679.232678
FLNB	15351.99219	16507.80321	15637.36786	25159.58511	22893.83057	25158.11276
SUPT16H	4321.066262	4232.770055	4617.984719	2812.357125	2678.894555	2841.151349
CCNB1	4460.0073	4135.935457	4156.934402	2694.122599	2779.768633	2617.188947
AFTPH	577.5977411	639.9069823	662.1168856	1143.535455	1117.638935	1262.513714
DOT1L	1413.228837	1352.689487	1324.233771	2243.748335	2169.938978	2253.497791
TBL3	1441.017045	1308.764515	1303.659518	757.2424993	761.1407721	774.9495481
HAGHL	499.1952986	462.2105036	368.4661764	184.1209414	159.3351917	147.6566275
ZNF148	470.4146551	511.12695	562.9863914	1063.208181	929.6463346	1039.542297
CALM3	8466.471356	7205.692041	6993.375806	4480.276241	4379.998329	4617.985798
TBC1D9	301.7005383	296.4935628	243.1502687	586.6598624	613.2685438	659.0044111
NFKB2	2325.277504	2053.492453	1991.961817	3686.931597	3842.385343	3382.228654

PRPF3	926.9352065	942.3903141	995.0457151	1567.735663	1688.494514	1635.123727
ZBTB21	438.6567037	508.1320655	489.1061175	916.9944926	882.6481845	987.0201406
BRSK2	75.42513457	67.88404805	76.68585398	252.7150176	225.8203797	232.8812581
SREK1	1583.927826	1820.889759	1906.859223	3022.652122	2958.590863	3077.005559
USP34	1009.307393	1516.409838	1592.63426	3200.455188	2799.255671	3300.96796
PRNP	4601.925645	4650.057291	4599.280852	7033.600473	7940.394772	6996.347583
CSRNP1	491.2558107	371.3656746	275.8820357	1778.03066	1917.753783	1641.069631
EPN2	808.8353247	807.6205128	845.4147805	1503.654355	1340.020426	1505.304813
ACAP3	1228.635745	1151.033932	1016.555162	612.8339178	584.6111352	585.6715895
IRS2	336.4357976	380.350328	304.8730292	715.7250321	832.2111454	684.7699971
PIDD1	765.1681415	664.864353	676.1447857	360.1189001	263.648159	303.2411275
MAPK6	1735.770531	1864.814732	2005.054524	3037.09298	3561.542739	3149.347396

SFPQ	5527.868415	5988.770651	5849.634349	8670.832766	8733.631841	8853.451743
GMEB2	1039.080472	1042.219797	994.1105217	1732.902978	1814.587112	1656.925377
HYAL2	517.0591462	434.2582486	413.3554568	194.951585	182.2611186	177.3861498
NRBF2	375.1408009	374.3605591	387.1700433	714.8224785	825.3333673	736.3011691
NLK	246.1241233	236.5958733	246.8910421	511.7479107	596.0740986	513.3297518
ADAM8	2363.982507	2249.158239	1937.720603	3778.992067	3913.455717	3582.407438
DYNC1LI2	1927.310676	2183.270781	2318.344293	3745.597583	3468.692735	3684.478798
RAB31	1771.498226	1957.65615	1756.293095	1000.931981	938.8167053	1118.821023
ACAP2	1480.714484	1929.703895	1950.81331	3270.854371	3349.477916	3324.751578
GOLT1A	12.90166776	6.98806377	14.96309346	118.2345261	116.922227	89.18856692
VARS2	700.6598027	652.8848151	637.8018587	323.1142011	342.7426067	283.421446
YPEL5	618.2876163	625.9308548	638.7370521	1137.217579	1444.333393	1146.568577

SPOPL	395.9819565	497.1508225	478.8189907	957.6094061	856.2833686	976.1193158
LIG4	135.9637294	133.7715065	137.4734212	352.8984711	357.6444592	327.0247454
ANK2	148.8653972	203.6521441	159.9180613	430.5180836	409.2277946	465.7625161
TGFBR1	1508.502691	1673.142125	1694.570334	960.317067	949.1333724	898.8225578
ATAD3A	1694.08822	1345.701423	1336.391285	754.5348384	703.8259549	773.9585641
GNAI3	2444.369822	2444.824025	2516.605281	3830.437624	4233.272397	3778.622285
ATAD2B	159.782193	249.5737061	229.1223686	521.6760007	530.735207	632.2478411
AMDHD2	498.2028626	474.1900415	359.114243	165.1673151	181.1148223	150.6295797
DBF4	1505.525384	1572.314348	1649.681054	883.6000081	874.6241101	967.2004591
RB1CC1	1786.384766	2197.246908	2283.742139	3734.766939	3661.270521	3686.460766
PMAIP1	761.1983976	761.6989509	798.6551134	1359.245773	1405.359317	1280.351427
IL13RA2	9.924359812	8.984653418	2.805580024	92.06047071	111.1907453	85.22463061

HYOU1	3958.827129	4053.076986	3801.560932	6093.139586	6616.422495	5883.472465
GOLGB1	1455.903584	1602.263193	1731.978068	2681.486848	2650.237146	3002.681753
LPP	1258.408824	1645.18987	1732.913261	3153.522399	2769.451966	3152.320349
CUX1	3061.665002	3357.265494	3145.055207	4969.460311	4820.176124	5185.819674
ADD2	200.4720682	183.6862477	178.6219282	434.1282981	421.8370544	439.9969301
STRN3	504.1574785	617.9444962	617.2276052	1105.628202	1099.298193	1103.956262
CCDC61	395.9819565	359.3861367	341.3455695	157.946886	124.9463014	108.0172644
HECTD4	1224.666001	1373.653678	1421.493879	2710.368564	2149.305644	2765.836559
CNOT1	3958.827129	4518.282375	4901.348301	7987.599665	7154.03548	8819.758285
HNRNPH1	5976.449479	6377.107337	6310.684667	9881.157189	9561.257801	9049.66659
RSRP1	572.6355612	592.9871256	608.8108651	1050.57243	1103.883379	1030.62344
RSBN1	257.0409191	319.4543438	323.5768961	636.3003123	631.6092853	655.0404748

YWHAQ	6943.082125	6916.186542	7102.793427	4700.499328	4788.079827	4581.319388
ZFAND2A	193.5250163	152.7391081	134.6678411	413.3695646	460.8111301	391.4387104
ZBTB1	459.4978593	530.0945517	448.8928038	868.2565963	975.4981883	956.2996342
MOGS	1929.295547	1801.922158	1781.543315	1148.950777	1063.763007	1008.82179
ATXN1	606.3783845	625.9308548	789.30318	1361.050881	1282.705608	1298.189141
HSPB11	519.0440182	522.1081931	518.097111	245.4945886	245.3074175	246.7550352
MDM2	1966.015679	2255.148008	2287.482913	3501.908101	3519.129775	3726.100129
PSMA7	4091.813551	3435.13249	3069.304546	1882.726881	2022.06675	1768.906577
ZKSCAN5	563.7036373	550.0604482	548.9584913	957.6094061	1004.155597	1066.298867
SMAD7	92.29654625	82.85847041	85.10259405	276.1814121	294.5981603	228.9173218
PTEN	865.4041756	1023.252195	1078.277922	1715.754459	1874.194522	1787.735275
SEC23A	176.6536047	202.6538493	189.8442483	450.3742636	412.6666837	447.9248028

SMARCD2	2891.958449	2627.511977	2589.550362	1671.529331	1694.225996	1623.231918
PPP1CB	2228.018778	2294.081506	2533.438761	3769.063977	4212.639062	3732.046034
TRIM8	904.1091789	888.4823936	894.0448342	1512.679891	1725.175997	1476.566275
AURKB	1790.35451	1541.367209	1577.671167	985.5885688	817.3092929	838.3725291
ATF6	901.1318709	988.311876	1020.295935	1614.668452	1721.737108	1637.105695
DTYMK	1162.142534	1080.155	1003.462455	604.7109351	588.0500242	578.7347009
CDT1	2860.200498	2365.958733	2453.947327	1523.510535	1477.575987	1341.79244
NDUFS5	4131.51099	3291.378036	3200.231614	1788.861303	2071.357493	1844.221367
KLHL9	919.9881546	1059.190809	1072.666762	1781.640874	1701.103774	1782.780354
LYN	655.0077476	673.8490064	641.5426321	332.1397375	322.1092725	343.8714747
SPC25	297.7307944	287.5089094	284.2987757	96.57323888	79.0944477	117.9271052
PACSIN3	2471.165593	2132.357745	1964.84121	1272.600625	1209.342642	1125.757911

CDKN2AIP	333.4584897	306.476511	355.3734697	684.1356549	665.9981756	647.1126022
TOR1AIP2	1475.752304	1456.512149	1564.57846	2299.70666	2553.948253	2522.054476
GPR1	11.90923177	31.94543438	21.50944685	123.6498479	158.1888954	132.7918663
ELF1	1042.05778	1179.984482	1293.372391	1987.423103	2082.820456	2111.787068
GNA13	1344.750755	1581.299002	1464.512772	2444.115242	2536.753808	2372.41588
NEURL1	90.31167429	96.83459795	85.10259405	244.5920349	292.3055676	262.6107804
LMNA	15296.41578	13343.20862	12251.96796	7862.144709	8567.418871	7374.9035
PANK3	1153.21061	1416.580356	1527.170726	2535.273159	2422.124174	2529.982348
SAC3D1	519.0440182	417.2872365	410.5498768	195.8541387	167.3592662	129.8189141
ATP6V1D	1021.216625	987.3135812	1007.203229	1708.53403	2085.113049	1684.672931
MYH14	307.6551542	256.5617698	216.0296618	564.0960215	652.2426195	596.5724143
WDR43	1994.796322	2372.946797	2231.371312	3625.557949	3506.520515	3800.423935

ARFGAP2	2477.120209	2264.132661	2262.232692	1478.382853	1429.43154	1374.494915
COL4A1	965.6402097	1131.068036	1154.963776	602.0032741	432.1537215	494.5010544
PTK7	2388.793407	2306.061044	2122.888885	1394.445365	1240.292644	1394.314596
XBP1	2699.425869	2429.849602	2669.041796	3994.702386	4692.93723	4234.474961
ZNF189	255.0560472	278.524256	270.2708756	534.3117516	602.9518767	551.9781308
FASN	15162.43692	15588.37368	14885.47241	10414.56639	8920.478145	9898.939944
MAOA	1053.967012	971.340864	991.3049417	560.485807	544.4907632	572.7887965
SUMO2	4114.639578	3845.431663	3923.136066	2307.829643	2634.188997	2163.31824
INTS5	793.948785	756.7074768	735.0619662	421.4925473	381.7166824	346.8444269
PGD	2402.687511	2183.270781	2023.75839	1263.575088	1342.313018	1272.423555
KLHL24	341.3979775	388.3366866	417.0962302	784.3191083	845.9667015	714.4995195
LATS1	587.5221009	685.8285443	808.9422402	1417.91176	1280.413016	1451.791673

FUBP1	1934.257727	2339.004773	2548.401855	3872.857645	4118.642762	4178.979852
TNKS	653.0228756	785.6580267	746.2842863	1361.953434	1241.43894	1454.764625
PITX1	1445.979225	1486.460993	1512.207633	901.6510808	833.3574417	899.8135418
POLR2A	5673.756505	5612.413502	5691.586675	3572.307285	3413.670511	3901.504311
GEMIN5	1060.914064	1152.032227	1271.862944	641.7156341	547.9296522	637.2027615
UBE2H	3406.040288	3532.965383	3505.104643	5346.72773	5336.009479	5062.937649
SUOX	678.8262112	593.9854204	621.9035719	287.0120557	301.4759383	329.0067135
COL4A2	2049.380301	2065.471991	2071.453251	1319.533414	1097.005601	1218.910415
DBI	1784.399894	1548.355272	1370.058245	870.0617036	821.8944783	749.1839621
ZBTB4	1109.543427	1076.161821	965.1195281	1840.306861	1730.907479	1768.906577
OSCAR	18.85628364	36.9369085	21.50944685	152.5315642	230.405565	116.9361211
PSMG4	568.6658172	473.1917467	432.994517	226.5409622	187.9926003	189.2779587

TXN	8129.043122	7317.501062	6954.097685	4144.526289	4755.983529	4680.417795
FIGNL1	1059.921628	1191.96402	1288.696424	2232.917692	1938.387117	2099.895259
BICD1	324.5265659	341.4168299	332.9288295	650.7411704	679.7537317	630.2658729
TMEM30B	958.6931579	1084.148179	1140.935876	597.490506	557.1000229	550.9871468
FADS3	1093.664451	975.3340433	912.748701	1676.042099	2012.896379	1760.978705
NAB2	2066.251713	1758.99548	1561.77288	3366.525056	3553.518665	3029.438323
CDKN1B	754.2513457	722.7654528	794.91434	1422.424528	1521.135248	1233.775176
FRMD6	1401.319605	1670.147241	1447.679292	2607.47745	2498.926029	2499.261842
STC1	5938.736912	6194.419385	5746.763082	3887.298503	3762.144599	4066.007668
YAP1	3023.952435	3180.56731	3206.777967	4721.258062	4679.181674	4782.489155
FOXRED1	1048.012396	930.4107762	880.9521274	525.2862152	495.2000204	486.5731818
LAPTM4B	7063.166878	6714.530988	6973.736746	4561.506068	4667.718711	4752.759633

TEDC1	491.2558107	487.1678742	407.7442968	194.0490314	216.6500089	180.359102
SPRED1	453.5432434	561.0416912	549.8936846	1031.618804	993.8389298	961.2545546
ZFX	408.8836243	372.3639694	432.0593236	804.1752883	758.8481794	765.0397074
ZXDC	571.6431252	558.0468068	577.0142915	981.0758006	1011.033375	1014.767695
GAGE1	1185.960998	1148.039048	976.3418482	623.6645614	490.614835	464.7715321
MRPL40	917.0108466	857.535254	823.9053336	426.9078691	498.6389094	433.0600416
CDCA2	759.2135256	772.680194	831.3868803	420.5899936	323.2555689	407.2944556
ZNF664	3184.727064	3403.187056	3738.902978	5427.957557	5583.609489	5379.061569
BACH2	132.9864215	166.7152357	153.371708	342.9703811	389.7407568	394.4116626
PIBF1	253.0711752	235.5975785	267.4652956	498.2096062	546.7833559	625.3109525
OAT	2980.285252	3203.528091	3345.186582	2009.08439	2032.383417	1991.877995
PARD6B	198.4871962	187.679427	210.4185018	405.2465818	512.3944656	474.6813728

MTFMT	286.8139986	285.5123197	283.3635824	118.2345261	96.28889286	94.14348731
DR1	1157.180354	1207.936737	1250.353497	1867.38347	2034.67601	2064.219832
DTX3L	1315.970111	1517.408133	1565.513653	2398.085007	2647.944554	2442.77575
WWC2	464.4600392	519.1133086	572.3383248	1056.890306	914.7444821	1032.605408
LTBP4	2822.487931	2506.718304	2403.446887	1585.786736	1438.601911	1559.808937
ETF1	4292.285619	4414.459713	4452.455498	6369.320998	6681.761386	6439.414532
MTX3	1029.156113	1155.027112	1257.835044	1962.151601	1937.240821	2021.607517
TPRG1L	1209.779461	1016.264131	1020.295935	550.557717	616.7074328	529.1854971
B3GNT5	994.4208532	1238.883877	1382.215758	2259.091747	2296.031576	2236.651062
MMP14	5507.02726	5095.296783	5051.914429	3357.49952	3374.696435	2893.673505
C1QTNF6	1297.113827	1271.827606	1250.353497	778.0012329	680.900028	740.2651055
GTF2F2	922.9654625	1092.134538	1082.953889	1769.907677	2081.67416	1768.906577

LPCAT4	1180.006382	1066.178872	982.8882016	604.7109351	513.5407619	576.7527328
LETM1	1319.939855	1326.733821	1251.288691	772.5859111	784.066699	736.3011691
NGF	6.947051869	9.982948243	7.48154673	81.2298271	87.11852211	96.12545546
ST14	1906.46952	1655.172819	1616.949287	1006.347302	1021.350042	940.443889
RSF1	1167.104714	1457.510443	1538.393046	2519.027194	2426.709359	2595.387297
ARIH1	911.0562308	992.3050553	1023.101515	1602.935255	1610.546363	1714.402453
JPT1	2995.171791	2730.336344	2593.291135	1724.779995	1701.103774	1708.456549
SPRR2D	7.93948785	5.989768946	9.351933412	78.52216619	111.1907453	81.26069431
E2F8	242.1543794	279.5225508	349.7623096	108.3064361	60.75370621	71.35085354
MPZL2	825.7067364	948.380083	887.4984808	466.620229	498.6389094	414.2313441
KPNA2	5992.328455	5913.898539	5815.967389	3825.924856	3977.648312	4088.800301
CLIC4	3126.173341	3640.781224	3890.4043	5875.62416	5901.133576	5819.0585

KDM4C	629.2044121	696.8097873	624.7091519	1164.294188	1215.074124	1093.055437
LSM7	1538.275771	1324.737232	1185.825157	691.356084	707.2648439	523.2395926
POLR2F	1493.616152	1271.827606	1099.787369	658.8641531	677.461139	568.8248601
PPM1B	225.2829677	224.6163355	255.3077822	531.6040907	581.1722462	463.780548
NRP2	825.7067364	856.5369592	903.3967676	468.4253363	411.5203873	484.5912136
HIST3H2BB	34.73525934	21.96248613	48.63005374	178.7056196	215.5037126	139.7287548
CENPW	579.582613	462.2105036	476.948604	212.1001041	208.6259345	240.8091307
KIF3B	1720.883991	1771.973313	1894.701709	2718.491547	2973.492715	2931.3309
STAT5A	163.7519369	177.6964787	131.8622611	420.5899936	466.5426118	349.8173791
IRF7	1090.687143	952.3732623	869.7298073	1680.554867	1931.509339	1705.483596
DCP2	739.364806	752.7142975	816.4237869	1284.333822	1301.04635	1373.503931
GRB7	1375.51627	1134.06292	1125.037589	2149.882757	2252.472315	2020.616533

DLC1	146.8805252	115.8021996	142.1493879	312.2835575	369.1074226	366.6641085
CTSA	2467.195849	2105.403784	2128.500045	1323.143628	1365.238945	1275.396507
PMPCA	2663.698174	2541.658623	2267.843852	1521.705428	1495.916728	1518.187606
SLC7A8	181.6157846	168.7118253	170.2051881	52.3481108	42.41296471	43.60329938
CDK1	1721.876427	1896.760166	1992.89701	1119.166507	1077.518563	1144.586609
TMEM243	812.8050686	776.6733733	864.1186473	433.2257445	452.7870557	437.0239779
PCLAF	469.4222191	404.3094038	436.7352904	188.6337096	206.3333418	186.3050065
POLE	3757.362625	3925.295249	4027.877721	2352.054771	1852.414891	2417.010164
STRIP2	386.0575967	482.1764001	460.1151239	193.1464778	196.0166747	149.6385956
KRT6A	21488.22387	18957.61871	17246.8356	11269.28468	11977.65049	12075.14098
CBL	704.6295467	1047.211271	1031.518255	2121.001041	1741.224146	2128.633797
ZNF674	31.7579514	43.92497227	50.50044043	178.7056196	163.9203771	164.5033568

MYADM	1876.69644	1815.898285	1652.486634	2790.695838	3057.172348	2823.313635
NSRP1	526.983506	488.1661691	561.1160047	897.1383126	1110.761157	1023.686551
NUMB	1027.171241	1091.136243	1156.834163	1722.072334	1859.292669	1904.671396
SKA2	1340.781011	1281.810554	1396.243658	733.7761048	780.6278099	823.5077679
SLC39A14	2316.34558	2523.689316	2624.152515	4159.869701	4182.835357	3721.145209
SBF2	817.7672485	915.4363538	933.3229545	1569.54077	1434.016726	1609.358141
HCFC2	73.44026261	107.815841	100.0656875	262.6431076	294.5981603	268.5566848
PRIM1	977.5494415	1135.061215	1196.112283	588.4649696	572.0018754	620.3560321
SNAPC4	1143.28625	1137.057805	1077.342729	679.6228867	623.5852109	587.6535576
B3GAT3	1061.9065	930.4107762	831.3868803	481.0610871	494.0537241	480.6272773
MRPL41	1574.995902	1301.776451	1078.277922	589.3675233	688.9241025	593.5994621
MSLN	55.57641495	63.89086875	50.50044043	203.9771214	198.3092674	179.3681179

MEX3D	1082.747656	1181.981072	1134.389523	677.8177794	616.7074328	544.0502582
RBM33	1082.747656	1061.187398	1111.009689	1702.216154	1745.809331	1819.446765
GGA2	1922.348496	2051.495864	1957.359663	1280.723607	1125.663009	1198.099749
CDH1	4175.178173	4470.364223	4513.243065	6450.550825	6489.183601	6777.340102
MIER1	572.6355612	665.8626478	750.960253	1244.621462	1310.216721	1195.126797
COL7A1	12363.76745	12416.79102	11640.35152	8560.721222	7840.66699	7963.548042
DNAJB4	287.8064346	361.3827264	402.1331367	789.7344301	902.1352223	682.788029
NTHL1	478.3541429	363.379316	355.3734697	169.6800833	145.5796356	143.6926912
BCR	3531.087221	3332.308123	3105.777086	2108.36529	1986.531563	2153.408399
SLC19A1	935.8671303	891.4772781	824.840527	501.8198207	363.3759409	422.1592168
CYB561A3	694.7051869	669.8558271	677.079979	376.3648655	340.450014	297.2952231
NCS1	1684.16386	1581.299002	1596.375033	1033.423911	856.2833686	912.6963348

NCKAP5L	580.575049	585.9990618	615.3572185	1093.895005	1030.520413	1002.875886
GTSE1	786.0092971	805.6239232	828.5813003	436.8359591	372.5463116	453.8707072
MT-ND5	38780.4284	35476.40317	33253.60483	19914.84594	22657.69353	23659.74484
FGF2	221.3132238	243.5839371	268.4004889	505.4300353	542.1981705	508.3748315
DUSP6	1794.324254	1707.084149	1494.438959	2633.651505	3030.807532	2835.205444
CAMTA2	531.9456859	530.0945517	578.8846782	972.0502643	981.2296701	950.3537298
LFNG	201.4645042	181.689658	175.8163482	61.37364714	38.97407568	48.55821977
TTC7B	185.5855285	202.6538493	188.9090549	442.2512809	425.2759434	405.3124875
EIF1B	410.8684962	380.350328	376.8829165	819.5187	918.1833712	680.8060608
CPT2	356.2845173	325.4441127	346.0215363	131.7728306	158.1888954	123.8730096
TRAFD1	1486.6691	1393.619575	1383.150952	839.37488	889.5259626	794.7692297
NCOR1	1507.510255	1701.094381	1758.163482	2777.157533	2575.727884	2972.952231

RALGAPB	1559.116926	1810.906811	1934.915023	3184.209222	2804.987153	3186.013807
NEBL	62.52346682	68.88234287	72.00988727	205.7822286	249.8926029	198.1968154
ZZEF1	666.9169794	842.5608317	800.5255001	1482.895621	1299.900054	1532.061383
ZNF207	3227.401811	3380.226275	3509.78061	4994.731813	5009.315021	5240.323799
PTHLH	2713.319973	2674.431834	2623.217322	1630.914417	1810.001926	1647.015536
WDR45B	2887.988705	2737.324408	2655.013896	4115.644573	4751.398344	4291.952037
TYMP	1812.188102	1595.275129	1578.60636	972.0502643	989.2537445	815.5798953
KLHL21	1852.877977	1818.89317	1686.153594	1093.895005	1101.590786	943.4168412
DCAF10	714.5539065	791.6477956	767.7937331	1277.113393	1343.459315	1249.630921
CRYBG1	2859.208062	3380.226275	3721.134305	1854.747719	1721.737108	1999.805867
NDUFAB1	1851.885541	1702.092675	1705.792654	1060.50052	1108.468564	1061.343946
CSNK1A1	2497.961365	2888.066927	3092.684379	4689.668684	4502.652037	4744.83176

YIPF4	749.2891658	842.5608317	880.0169341	1655.283366	1863.877855	1343.774408
CTNNA1	8797.944973	8704.132573	8577.593326	12170.93576	13725.75242	13008.64798
LCOR	808.8353247	1005.282888	1088.565049	1904.388169	1658.690809	1995.841931
TMEM135	433.6945238	458.2173243	508.7451776	874.5744717	900.988926	826.4807201
USP5	3109.301929	2815.191404	2769.107483	1855.650272	1738.931553	1860.077112
CCNL1	4348.85447	5004.451954	4944.367195	7332.345726	7620.578092	7213.373096
ADGRG6	857.4646878	1097.126012	1085.759469	1954.028618	1720.590812	1884.851714
SLC27A3	256.0484832	242.5856423	260.9189422	96.57323888	93.99630017	81.26069431
CCNT1	779.0622453	832.5778834	937.0637279	1556.002466	1387.018576	1570.709762
PKHD1	49.62179906	63.89086875	60.78756718	191.3413705	182.2611186	213.0615765
TFAP4	798.9109649	717.7739786	687.3671058	389.0006164	393.1796458	331.9796658
HCFC1R1	830.6689163	760.7006561	679.8855591	364.6316683	414.9592764	368.6460766

MYO10	3970.736361	4208.810979	4439.362791	6455.063593	6151.026179	6750.583532
BIRC6	1149.240866	1491.452467	1578.60636	2936.909526	2372.833431	3125.563779
RBM48	541.8700457	499.1474121	599.4589317	944.9736552	1047.714858	1021.704583
TRAK1	1295.128955	1449.524085	1328.909738	2285.265802	2157.329718	2127.642813
NDRG1	20775.65483	20126.62195	14621.74789	33236.53759	38227.83676	32992.83287
RAPGEF1	2327.262376	2331.018415	2367.90954	3629.168164	3488.179773	3438.714747
ADAM17	806.8504527	883.4909195	938.9341146	1464.844549	1450.064875	1537.016303
MBOAT7	4734.912066	4311.635346	4317.787656	2980.232101	2851.985303	2929.348931
LRPPRC	4310.149466	4707.958391	4985.515702	6989.375345	7167.791036	7423.46172
PGRMC1	3192.666552	3135.644043	3210.51874	2155.298079	2061.040826	1902.689428
ZNF75A	290.7837425	279.5225508	255.3077822	538.8245197	552.5148376	566.842892
FN3KRP	744.3269859	701.8012615	607.8756718	338.4576129	363.3759409	319.0968728

TNFAIP1	1604.768982	1385.633216	1408.401172	2346.639449	2489.755658	2346.650294
B4GALT5	5510.997004	5640.365757	5882.366116	3701.372455	3625.735335	3970.873196
BICDL2	169.7065528	196.6640804	184.2330882	422.3951009	434.4463142	381.5288696
DZIP1L	223.2980958	265.5464233	215.0944685	78.52216619	53.87592815	83.24266246
CSNK2A2	1703.020144	1819.891465	1895.636903	2812.357125	3069.781608	2763.854591
AP1B1	3886.379302	3666.736889	3499.493483	2252.773871	2285.714909	2495.297906
SLC25A10	441.6340116	385.3418022	292.7155158	151.6290106	128.3851905	123.8730096
ASH1L	1197.870229	1533.38085	1743.200388	2914.345686	2643.359368	3178.085935
SORD	1780.43015	1673.142125	1870.386682	1126.386936	1053.44634	1075.217723
PSEN1	736.3874981	765.6921302	764.9881531	1227.472943	1414.529688	1259.540762
WASHC4	1478.729612	1613.244436	1783.413702	2769.937104	2543.631586	2798.539033
AGTRAP	1209.779461	977.3306329	999.7216818	574.9266651	583.4648389	542.0682901

PARP1	5297.623268	5294.955748	5458.723533	3580.430268	3124.803832	3609.164008
CCN3	234.2148916	262.5515388	272.1412623	83.03493437	102.0203746	90.179551
LAMTOR3	378.1181088	346.408304	362.8550164	688.6484231	741.6537343	650.0855545
STUB1	1200.847537	980.3255174	986.628975	580.3419869	574.2944681	542.0682901
BLACAT1	333.4584897	341.4168299	322.6417027	136.2855988	130.6777832	149.6385956
MDK	1919.371188	1676.13701	1553.35614	963.9272815	1020.203746	980.0832521
HMGB1	10957.48567	10860.44939	11648.76826	7315.197207	7928.931808	7314.453472
VCL	4355.801522	4611.123793	4509.502291	6932.514466	6330.994705	6968.600029
SAMD1	831.6613523	784.6597319	798.6551134	443.1538345	417.251869	465.7625161
GALNT7	1051.98214	1288.798618	1332.650511	669.6947967	613.2685438	683.7790131
TTI1	1434.069993	1342.706539	1602.921387	782.514001	750.824105	898.8225578
MBD1	941.8217462	860.5301385	828.5813003	1422.424528	1506.233395	1462.692498

HAS2	31.75795<br>14	40.93008<br>779	34.60215<br>363	137.1881<br>524	173.0907<br>479	138.7377<br>708
LDLRAP1	836.6235<br>322	804.6256<br>284	785.5624<br>066	453.0819<br>245	463.1037<br>228	429.0961<br>053
UBL5	1758.596<br>559	1412.587<br>176	1134.389<br>523	713.9199<br>248	685.4852<br>134	628.2839<br>048
TADA2B	181.6157<br>846	203.6521<br>441	198.2609<br>883	457.5946<br>926	412.6666<br>837	407.2944<br>556
TMPO	2335.201<br>864	2643.484<br>695	2805.580<br>024	1658.893<br>58	1431.724<br>133	1485.485<br>131
TNFSF15	38.70500<br>327	27.95225<br>508	54.24121<br>379	170.5826<br>369	168.5055<br>625	159.5484<br>364
RRAS2	1399.334<br>734	1461.503<br>623	1388.762<br>112	2261.799<br>408	2482.877<br>88	2139.534<br>622
PLEKHM1	433.6945<br>238	425.2735<br>951	398.3923<br>634	760.8527<br>138	801.2611<br>441	741.2560<br>895
RAB26	252.0787<br>392	203.6521<br>441	214.1592<br>751	68.59407<br>621	81.38704<br>039	69.36888<br>538
SOD1	3533.072<br>093	3139.637<br>222	2860.756<br>431	1852.040<br>058	1997.994<br>527	1675.754<br>074
CCNB2	1401.319<br>605	1351.691<br>192	1389.697<br>305	834.8621<br>118	872.3315<br>174	832.4266<br>246
LZTS3	160.7746<br>29	156.7322<br>874	138.4086<br>145	342.0678<br>274	444.7629<br>813	346.8444<br>269

DIPK2A	286.8139986	368.3707902	382.4940766	667.8896895	770.3111428	711.5265672
C7orf50	2072.206329	1794.934094	1722.626135	1117.361399	1021.350042	844.3184335
KCMF1	1357.652422	1317.749168	1312.076258	1991.033318	2322.396392	2169.264144
PNPO	665.9245434	638.9086875	648.0889855	370.0469901	294.5981603	316.1239205
KIRREL1	1705.005016	1680.130189	1616.949287	2858.38736	2439.318619	2808.448874
CDK12	1160.157662	1242.877056	1472.929512	2299.70666	2110.331568	2363.497023
AZIN1	3037.846538	3350.27743	3576.179337	5418.029467	5072.36132	5129.333582
HSP90AB1	50318.48912	46864.95052	46985.04866	31057.77311	34288.01623	32861.03199
TSEN15	971.5948256	1070.172052	1111.944883	1680.554867	1765.296369	1722.330326
QSOX2	1696.073092	1712.075624	1774.061768	1133.607365	986.9611518	1037.560329
BRD2	3553.913249	3566.907407	3416.261275	5067.838657	5235.135401	5115.459805
FANCA	1072.823296	1025.248785	1101.657756	593.8802914	431.0074252	591.6174939

NME3	1051.98214	810.6153973	730.3859995	429.61553	375.9852007	297.2952231
RORA	28.78064346	33.94202402	30.86138026	121.8447406	149.0185247	137.7467867
KIAA0753	320.5568219	343.4134195	330.1232495	609.2237032	620.1463218	692.6978698
NDUFB2	939.8368742	764.6938354	704.2005859	410.6619037	387.4481641	310.1780161
PNPLA8	408.8836243	496.1525277	453.5687705	827.6416828	991.5463371	824.498752
PHYKPL	970.6023896	955.3681468	997.8512951	1604.740362	1605.961177	1505.304813
PER3	426.7474719	530.0945517	488.1709241	224.735855	198.3092674	232.8812581
MAP1LC3B2	44.65961915	45.92156192	59.85237384	170.5826369	210.9185272	164.5033568
TUBGCP5	478.3541429	562.0399861	527.4490445	259.9354467	246.4537139	256.6648759
SMOC1	948.768798	823.59323	809.8774335	1409.788777	1513.111173	1475.575291
RBL1	997.3981611	1228.900929	1272.798137	600.1981669	518.1259473	659.9953952
PLD6	260.0182271	238.592463	214.1592751	90.25536344	81.38704039	69.36888538

ERH	2140.684411	1891.768692	1791.830442	1077.649039	1227.683384	1091.073469
SLC43A2	168.7141168	156.7322874	164.5940281	53.25066443	37.82777934	36.66641085
NDUFB6	1090.687143	909.4465849	1031.518255	592.0751842	523.857429	472.6994047
ANKRD37	102.2209061	84.85506006	61.72276052	239.1767131	304.9148274	240.8091307
SLC6A6	1586.905134	1708.082444	1765.645028	1042.449448	1023.642635	1062.33493
SLC44A2	2622.015862	2336.009889	2332.372193	1583.079075	1494.770432	1478.548243
BIRC5	2621.023426	2121.376502	2190.222805	1400.763241	1361.800056	1243.685017
RTN4R	175.6611687	179.6930684	148.6957413	47.83534262	37.82777934	50.54018792
AMIGO2	2651.788942	2755.293715	2496.966221	4167.09013	4026.939055	3853.937075
BARD1	468.4297831	535.0860258	571.4031315	268.0584294	196.0166747	236.8451944
CLDN15	173.6762967	164.718646	131.8622611	417.8823327	338.1574213	385.4928059
TTC37	1775.46797	2032.528262	2068.647671	3227.531797	2970.053826	3291.058119

PLTP	2521.779828	2095.420836	2042.462257	1247.329123	1372.116723	1187.198924
STK3	502.1726065	563.0382809	545.2177179	903.456188	966.3278176	965.2184909
CAVIN3	517.0591462	424.2753003	387.1700433	199.4643532	193.7240821	179.3681179
ATP6V0A1	805.8580167	790.6495008	786.4976	1336.681933	1247.170422	1477.557259
GPCPD1	2145.646591	2381.931451	2312.733133	3620.142628	4219.51684	3455.561476
SPRY2	460.4902953	539.0792051	402.1331367	903.456188	873.4778138	912.6963348
CHCHD10	1700.042836	1646.188165	1521.559566	963.0247279	794.3833661	612.4281595
VLDLR	376.1332369	380.350328	378.7533032	725.6531221	682.0463244	676.8421245
PRKAA2	127.0318056	173.7032994	159.9180613	431.4206372	333.572236	383.5108378
ADGRF1	138.9410374	157.7305822	138.4086145	37.90725265	38.97407568	31.71149046
CTPS1	1962.045935	1819.891465	1993.832203	1235.595926	1107.322268	1220.892383
RRP1	1131.377019	986.3152864	975.4066549	573.1215578	609.8296548	533.1494334

SYT1	202.4569402	242.5856423	252.5022021	459.3997999	502.0777985	539.0953378
ZFHX3	341.3979775	423.2770055	489.1061175	909.7740635	774.8963282	1018.731631
BNIP1	274.9047668	242.5856423	225.3815952	474.7432117	561.6852083	532.1584493
SLITRK6	771.1227574	900.4619315	846.3499738	1373.686632	1419.114873	1420.080182
MET	6342.658356	7869.5581	7575.066064	11972.37396	11122.51342	12097.93361
GOLM1	2105.949152	2134.354334	2128.500045	1383.614722	1192.148197	1370.530978
PAFAH1B1	2301.45904	2289.090032	2296.834846	3326.812696	3457.229772	3407.99424
PRPH	2.977307944	2.994884473	4.675966706	65.88641531	90.55741114	66.39593315
MFSD3	508.1272224	402.3128142	415.2258435	205.7822286	199.4555638	178.3771338
ASAP1	820.7445565	890.4789832	894.9800276	1449.501137	1361.800056	1564.763857
DBT	512.0969663	580.0092929	634.9962787	1050.57243	1003.009301	1047.470169
BTAF1	1000.375469	1279.813965	1319.557804	2289.77857	1978.507489	2419.983116

BTN3A3	215.3586079	211.6385027	211.3536951	71.30173712	76.80185502	72.34183761
CAPRIN2	1200.847537	1416.580356	1419.623492	2178.764473	2166.500089	2285.209281
NEDD9	163.7519369	173.7032994	175.8163482	339.3601665	419.5444617	439.9969301
NIPBL	2311.3834	2821.181173	2886.941844	4501.034975	4202.322395	4828.074423
TP53BP2	940.8293102	1108.107255	1106.333723	1720.267227	1726.322293	1814.491845
EGR2	18.85628364	22.96078096	5.611160047	111.9166507	106.6055599	110.9902166
ARHGAP1	2280.617885	2184.269075	2116.342531	1344.804915	1468.405616	1383.413771
RPL22L1	828.6840443	868.5164971	764.9881531	1315.923199	1660.983402	1436.926912
THAP7	624.2422322	518.1150138	490.0413108	257.2277858	268.2333444	206.124688
MRPL28	2344.133788	1915.727768	1675.866467	1084.869469	1088.981526	940.443889
ZRANB1	231.2375836	275.5293715	268.4004889	487.3789626	561.6852083	554.9510831
NUFIP2	2148.623899	2684.414782	2868.237978	4649.956324	4259.637213	4411.86111

ADAT2	512.0969663	509.1303604	544.2825246	284.3043948	232.6981577	243.7820829
ROR1	26.79577149	25.95566543	37.40773365	147.1162424	111.1907453	155.5845001
SYNPO	270.9350229	333.4304713	305.8082226	724.7505684	621.2926182	562.8789557
ZFP91	1901.50734	2088.432772	2240.723246	3194.137312	3357.50199	3262.319581
TTYH3	2775.843439	2633.501746	2413.734014	1729.292764	1549.792656	1565.754842
SOS2	355.2920813	446.2377864	526.5138511	862.8412745	946.8407797	914.678303
ZNF789	637.1438999	629.9240341	670.5336257	351.9959174	338.1574213	350.8083632
ROCK1	1049.997268	1132.066331	1264.381397	1886.337096	1848.976002	1993.859963
AHNAK	23035.43156	28431.43659	28100.68952	42022.89722	41216.23133	43075.10487
PLRG1	1274.2878	1316.750873	1406.530785	2020.817587	2197.450091	2110.796084
INO80	1058.929192	1068.175462	1154.028583	1692.288065	1741.224146	1868.004985
DERA	963.6553378	955.3681468	923.9710211	563.1934679	565.1240974	508.3748315

ZMYM2	1001.367905	1236.887287	1230.714437	2010.889497	1881.0723	2049.355071
PLEKHG3	1716.914248	1735.036405	1505.661279	2812.357125	2882.935304	2481.424129
SLC25A36	1442.009481	1886.777218	1817.080662	3070.487464	2972.346419	2851.061189
SMIM10L1	438.6567037	429.2667744	475.0782173	787.9293228	975.4981883	801.7061182
DENND4C	606.3783845	849.5488954	880.9521274	1674.236992	1391.603761	1661.880297
PARD3	996.4057251	950.3766727	1024.971902	1523.510535	1605.961177	1662.871281
GORAB	178.6384766	164.718646	203.8721484	395.3184919	396.6185349	413.2403601
AP3S1	7244.782663	7215.67499	7316.017508	10668.18396	12337.58755	10430.10741
FAF2	1919.371188	2033.526557	2015.34165	2948.642724	2983.809382	2992.771912
CCDC186	358.2693892	512.1252448	510.6155643	907.0664026	946.8407797	952.3356979
CD99L2	531.9456859	527.0996672	537.7361712	296.9401457	231.5518614	228.9173218
LRRFIP1	2438.415206	2759.286894	2842.052564	3983.871742	4553.089076	4311.771719

CLIP2	563.7036373	528.097962	512.485951	920.6047071	909.0130004	941.4348731
GLIS2	255.0560472	272.534487	254.3725888	534.3117516	504.3703911	504.4108951
EEPD1	192.5325804	170.7084149	171.1403814	51.44555716	57.31481718	54.50412423
C20orf27	1230.620617	1085.146474	932.3877612	582.1470942	543.3444668	597.5633984
PCK2	1188.938305	1115.095319	1118.491236	1788.861303	2023.213046	1768.906577
TCF12	737.379934	765.6921302	837.9332337	1369.173863	1227.683384	1460.710529
KRT7	2116.865948	1922.715832	1754.422708	3033.482765	3687.635337	3151.329365
EXOSC2	2086.100433	1896.760166	1931.17425	1304.190002	1202.464864	1195.126797
NDEL1	561.7187654	597.9785997	592.9125783	1009.054963	998.4241152	965.2184909
EHD1	1345.743191	1276.81908	1224.168084	2154.395525	2441.611212	1944.310759
KIF23	1662.330269	1732.04152	1849.812429	1024.398375	1046.568562	1145.577593
TBC1D16	1481.70692	1413.585471	1439.262552	944.0711016	831.064849	863.147131

MAN1B1	2532.696624	2475.771164	2354.816833	1666.114009	1556.670434	1513.232685
NIT1	744.3269859	647.8933409	644.3482121	1133.607365	1184.124123	1163.415306
NRP1	390.0273406	358.3878419	466.6614773	817.7135928	737.0685489	800.7151341
PEX26	1378.493578	1363.67073	1545.874593	843.8876482	850.5518869	889.9037011
DGCR8	1116.490479	1190.965725	1189.56593	729.2633366	647.6574341	694.6798379
CYBC1	932.8898223	859.5318437	909.943121	527.0913225	518.1259473	517.2936881
CTDSP2	5710.476636	5787.115096	5647.632588	8272.806613	8179.970707	7932.827536
KIAA1324L	950.75367	1000.291414	1067.055602	1584.884182	1609.400066	1669.80817
POLE4	570.6506892	496.1525277	454.5039638	228.3460695	261.3555663	197.2058313
ZWINT	2171.449927	1879.789154	1809.599115	1201.298887	1163.490789	1175.307115
KANK2	681.8035191	727.7569269	714.4877127	403.4414746	392.0333495	344.8624588
PCGF2	151.8427051	140.7595702	148.6957413	312.2835575	349.6203848	355.7632836

ZNF562	445.6037556	506.1354759	503.1340176	819.5187	887.2333699	899.8135418
PRPF4	1748.672199	1656.171113	1754.422708	1078.551593	1129.101898	1064.316899
DNAJC18	94.28141822	109.8124307	143.0845812	291.5248239	359.9370519	295.3132549
HSPE1	1645.458857	1281.810554	1085.759469	615.5415787	710.703733	530.1764811
MECP2	684.780827	732.748401	776.2104732	1429.644957	1142.857454	1344.765392
DECR2	316.587078	325.4441127	338.5399895	134.4804915	128.3851905	154.593516
EIF5A	16069.52341	13583.79767	12364.19116	8493.932253	8856.28555	8239.041615
ZNF131	1161.150098	1176.989598	1266.251784	1901.680508	1823.757483	2025.571453
SEC62	1751.649507	1806.913632	2048.073417	2932.396758	2912.739009	2958.08747
HAUS8	309.6400261	283.5157301	320.771316	127.2600625	128.3851905	127.8369459
FAM114A1	425.7550359	394.3264556	454.5039638	193.1464778	202.8944528	206.124688
LOX	225.2829677	242.5856423	245.9558487	478.3534262	516.9796509	454.8616913

SUMO3	1954.106447	1726.051751	1686.153594	1018.983053	1157.759307	1031.614424
EMC10	3015.020511	2641.488105	2530.633181	1679.652314	1748.101924	1498.367924
UNC45A	3254.197582	2954.95268	2785.00577	1950.418404	1928.07045	1825.39267
DGCR2	1901.50734	1784.951146	1773.126575	1210.324424	1103.883379	1122.784959
APOL2	926.9352065	757.7057716	837.9332337	476.548319	386.3018678	448.9157868
SLC35F2	875.3285354	903.456816	899.6559943	1380.004507	1490.185247	1426.026087
TCP11L2	229.2527117	249.5737061	246.8910421	472.9381044	555.9537266	467.7444843
KLHL2	464.4600392	504.1388862	533.0602045	879.9897935	870.0389247	879.0028762
GABARAPL1	1006.330085	927.4158917	936.1285346	1523.510535	1752.687109	1510.259733
HSPG2	2880.049217	3431.139311	3490.141549	1048.767323	716.4352147	1171.343179
CNTN1	1034.118292	1308.764515	1360.706311	595.6853987	600.659284	721.436408
LSM2	784.0244252	713.7807993	724.7748395	386.2929555	437.8852032	389.4567422

TBK1	1113.513171	1129.071446	1269.057364	1835.794092	1862.731558	1927.46403
FOXRED2	736.3874981	745.7262337	902.4615743	419.68744	395.4722385	439.0059461
ETV3	800.8958368	842.5608317	848.2203605	1351.122791	1401.920428	1292.243236
LIMK2	2630.947786	2463.791626	2172.454132	3931.523631	3712.853857	3859.88298
YOD1	595.4615887	815.6068714	822.9701403	1396.250472	1420.26117	1379.449835
TLCD1	475.376835	447.2360813	443.2816437	231.0537304	217.7963053	223.9624014
SLITRK5	315.594642	362.3810212	428.3185503	154.3366715	137.5555612	163.5123727
ATP1B3	3247.250531	3148.621876	3128.221726	2184.179795	2150.45194	2118.723956
SUPT7L	577.5977411	637.9103927	681.7559458	1025.300929	1137.125973	1209.000574
PSMD2	12527.51939	11231.81507	11100.74496	7985.794557	7889.957732	7778.23402
CCNE2	91.30411027	121.7919686	105.6768476	21.66128723	9.170370748	19.81968154
NPEPPS	1854.862849	1874.79768	2028.434357	2815.064786	3053.733459	2947.186645

RSL24D1	1988.841706	1985.608405	2042.462257	3079.513001	3577.590888	2969.979279
HSPH1	2949.519736	2978.911756	3210.51874	4374.677466	4853.418718	4571.409547
DUSP10	205.4342481	205.6487338	177.6867348	386.2929555	441.3240923	440.9879142
ARHGEF26	335.4433617	331.4338817	397.45717	154.3366715	127.2388941	163.5123727
TRAF7	3989.592644	3936.276492	3691.208118	2709.46601	2501.218622	2496.28889
TMEM97	494.2331186	456.2207347	522.7730777	255.4226785	236.1370468	237.8361785
SLC31A1	1165.119842	1199.950379	1281.214877	1893.557525	1922.338968	1871.968921
MYC	6628.479919	6288.259098	6037.608211	8971.383126	9084.398522	9488.672536
MROH6	2020.599658	1799.925568	1631.91238	1118.263953	1011.033375	1079.18166
POGK	1098.626631	1186.972546	1163.380516	1863.773255	1751.540813	1790.708227
FNDC3A	1145.271122	1411.588881	1591.699067	2530.760391	2316.66491	2653.855358
STX6	1333.833959	1360.675845	1423.364265	2161.615954	2055.309344	2102.868211

SH3BGRL2	248.1089953	281.5191404	256.2429755	492.7942844	531.8815034	526.2125448
GATAD1	1223.673565	1268.832722	1230.714437	1895.362632	1991.116749	1868.004985
FANCG	1316.962547	1125.078267	1131.583943	660.6692604	628.1703962	733.3282169
MON1B	999.3830331	1041.221502	1024.036709	1561.417788	1616.277844	1620.258966
RNF26	935.8671303	910.4448797	812.6830135	504.5274816	460.8111301	518.2846722
HSPBAP1	206.4266841	187.679427	199.1961817	375.4623119	516.9796509	449.9067709
NAGS	161.7670649	157.7305822	124.3807144	361.9240074	331.2796433	343.8714747
CSNK1D	3297.864766	3236.47182	2808.385604	4985.706276	4934.805759	4622.940719
SH3PXD2B	849.5251999	911.4431745	927.7117945	1601.130147	1406.505613	1432.962975
ZCCHC7	460.4902953	514.1218345	506.8747909	839.37488	880.3555918	863.147131
C1QBP	3878.439815	3567.905702	3457.409783	2426.064169	2448.48899	2277.281409
AP002956.1	212.3813	201.6555545	200.131375	74.00939802	74.50926233	64.413965

BTN3A1	449.5734995	425.2735951	453.5687705	197.6592459	213.2111199	231.890274
LIMK1	537.9003018	478.1832208	483.4949574	272.5711976	189.1388967	209.0976402
PDLIM5	1153.21061	1237.885582	1211.075377	1829.476217	1857.000076	1882.869746
UCK2	1605.761418	1460.505328	1515.948406	897.1383126	981.2296701	962.2455387
LONP1	2676.599841	2396.905873	2347.335286	3728.449064	3938.674236	3701.325527
SOX9	529.960814	645.8967513	550.828878	1018.0805	1041.983376	985.0381725
SERINC2	4320.073826	3524.979024	3327.417908	2165.226169	2187.133423	2280.254361
KIAA0355	292.7686145	307.4748059	262.7893289	595.6853987	594.9278023	516.3027041
MRPL51	2205.19275	1834.865887	1674.931274	983.7834615	1108.468564	758.1028188
DENND5A	1243.522284	1203.943558	1188.630737	1803.302162	1894.827856	2024.580469
TAPBP	3016.012947	2715.361922	2858.886044	1824.963449	1960.166747	1820.437749
AP2M1	10639.90615	9408.928719	8636.510506	6151.805572	6002.007655	6253.109525

SIK3	628.2119761	736.7415803	617.2276052	1102.920541	1132.540787	1198.099749
FANCE	817.7672485	780.6665526	708.8765526	426.0053154	436.7389069	419.1862645
SDC4	8609.382137	8006.324491	7777.067826	12966.08551	14911.02284	11640.09897
NET1	3843.704555	4016.140078	3939.969547	2639.969381	2784.353818	2640.972565
SCP2	1154.203046	1249.86512	1255.029464	748.2169629	745.0926233	625.3109525
ERCC6L	411.8609322	366.3742005	494.7172775	207.5873359	152.4574137	167.476309
MKI67	3937.985973	4366.541561	4942.496808	2414.330972	2064.479715	2833.223476
NDUFAF8	1306.045751	1093.132833	1065.185216	626.3722223	695.8018805	600.5363506
CDS1	416.8231121	512.1252448	486.3005374	842.0825409	847.1129979	858.1922106
FNDC10	195.5098883	224.6163355	232.863142	65.88641531	82.53333673	79.27872615
TRPM7	851.5100719	1044.216386	1106.333723	1789.763857	1654.105624	1775.843466
HSP90AA1	35764.41546	35341.63337	37478.80834	25333.77796	25785.93625	26494.95028

LRIG3	906.0940508	935.4022503	947.3508547	549.6551634	372.5463116	484.5912136
G6PD	1762.566303	1692.109727	1562.708073	979.2706933	1087.83523	1033.596392
SDSL	343.3828495	256.5617698	290.8451291	106.5013289	104.3129673	129.8189141
GNAI2	4458.022428	4073.042883	4054.998328	2868.31545	2843.961228	2718.269323
ANXA8L1	2601.174707	2331.018415	2263.167886	1514.484999	1576.157472	1480.530211
PRR11	1372.538962	1440.539431	1661.838567	911.5791708	798.9685514	886.9307488
PAWR	932.8898223	1056.195924	1157.769356	1767.200016	1814.587112	1682.690963
GAB2	275.8972028	296.4935628	303.0026426	537.9219661	550.2222449	579.725685
ELAC2	1969.985423	1850.838604	1796.506408	1218.447406	1140.564862	1219.901399
IER2	1117.482915	1013.269247	772.4696999	2122.806148	2027.798232	1627.195854
H2AFV	2835.389598	2513.706367	2362.29838	1667.919116	1583.03525	1492.42202
KMT5C	826.6991724	736.7415803	718.2284861	444.9589418	398.9111275	351.7993473

PLK2	1997.77363	1951.666381	1840.460496	2838.53118	2983.809382	2866.916935
MRPS26	835.6310962	726.7586321	664.9224656	418.7848864	365.6685336	319.0968728
PSMB8	1223.673565	1068.175462	943.6100813	564.0960215	621.2926182	585.6715895
SOX12	767.1530135	698.806377	707.006166	1358.34322	1384.725983	1107.920198
CUL4A	1497.585896	1453.517264	1518.753986	2267.21473	2211.205647	2242.596966
LAMTOR4	2992.194483	2466.786511	2057.425351	1344.804915	1432.870429	1245.666985
HMGN3	867.3890476	828.5847041	848.2203605	447.6666027	520.41854	384.5018218
TMEM107	465.4524752	384.3435073	363.7902097	188.6337096	174.2370442	181.3500861
FSCN1	29717.50302	28512.29848	26532.37028	19995.17322	19767.88044	18595.8162
PARP14	1684.16386	1861.819847	1979.804303	2878.24354	2798.109375	3208.806441
NUCB2	451.5583715	457.2190295	431.1241303	733.7761048	851.6981832	809.6339908
IER5	2045.410557	1984.610111	1874.127456	3151.717291	3336.868656	2822.322651

MAP4K3	572.6355612	648.8916358	681.7559458	1083.966915	1166.929678	1046.479185
RBM26	1357.652422	1476.478045	1525.30034	2272.630051	2176.816756	2300.074043
PIMREG	588.5145369	526.1013724	488.1709241	275.2788585	260.20927	272.5206212
ZMYM5	209.403992	224.6163355	191.714635	431.4206372	443.6166849	415.2223282
RPA3	1130.384583	1031.238553	923.9710211	542.4347343	627.0240999	529.1854971
VARS	3771.256729	3473.067694	3276.917468	2329.49093	2324.688985	2250.524839
TAF1A	317.579514	333.4304713	330.1232495	675.1101185	608.6833584	571.7978124
SLC16A9	186.5779645	210.6402079	232.863142	74.00939802	68.77778061	75.31478985
ADIPOR2	2875.087038	2864.107851	2916.868031	4112.034358	4216.077951	4114.565887
SMPD4	2134.729796	1973.628868	1974.193143	1317.728306	1335.43524	1306.117013
ZXDB	168.7141168	186.6811321	186.1034749	385.3904019	377.131497	381.5288696
CPA4	266.9652789	235.5975785	244.0854621	83.03493437	74.50926233	110.9902166

DMRTA1	99.24359812	122.7902634	123.445521	276.1814121	284.2814932	284.4124301
LPAR3	748.2967298	710.7859149	637.8018587	1112.848631	1350.337093	1221.883367
PODXL2	1416.206145	1244.873646	1317.687418	843.8876482	794.3833661	731.3462488
LMF2	1130.384583	1019.259016	1016.555162	650.7411704	623.5852109	593.5994621
NOD1	241.1619434	276.5276663	288.0395491	110.1115434	105.4592636	107.0262803
AATF	1567.056414	1465.496802	1348.548798	2310.537304	2472.561213	2203.948587
SGTB	296.7383584	279.5225508	315.160156	572.2190042	569.7092827	544.0502582
COMMD4	941.8217462	784.6597319	732.2563862	397.1235991	472.2740935	371.6190288
PGAM1	10014.67149	9608.587683	9285.534685	6857.602514	6853.705838	6545.449828
MMP24OS	926.9352065	811.6136921	770.5993132	440.4461736	490.614835	447.9248028
BDP1	916.0184107	1310.761104	1385.956532	2637.26172	2057.601937	2663.765199
KATNB1	1001.367905	858.5335489	879.0817408	526.1887689	505.5166875	531.1674652

SYNGR2	2532.696624	2327.025235	2194.898772	1557.807573	1450.064875	1485.485131
COX6B1	1783.407458	1620.2325	1448.614486	938.6557798	992.6926335	970.1734113
SPAG9	2669.652789	3171.582657	3211.453934	4719.452954	4572.576114	5043.117967
TIMM8A	619.2800523	561.0416912	674.274399	331.2371838	291.1592713	334.952618
GTPBP10	805.8580167	932.4073659	998.7864884	1546.976929	1517.696359	1508.277765
EIF3A	6882.54353	7276.570974	7205.664694	9798.122255	10243.30413	10126.86628
ATP6V1E1	2836.382034	2547.648392	2556.818595	1598.422487	1804.270445	1674.76309
CIAO2A	748.2967298	724.7620424	787.4327933	444.9589418	427.5685361	405.3124875
BAZ1B	2795.692159	2825.174353	3092.684379	4362.944269	4157.616838	4551.589865
TPT1	12356.8204	11142.96683	10566.74956	16700.85245	21721.16941	18143.92746
SNRPD2	7918.646694	6591.740725	5463.399499	3865.637216	3843.53164	3496.191823
TSEN54	1192.908049	1017.262426	930.5173745	616.4441323	550.2222449	493.5100703

COG3	601.4162046	652.8848151	649.0241788	1013.567731	1075.22597	1073.235755
RAI14	3501.314142	3835.448715	3935.29358	2502.781228	2333.859355	2567.639743
GEM	94.28141822	83.85676524	85.10259405	206.6847823	310.6463091	240.8091307
PARP6	549.8095336	591.9888308	556.440038	916.0919389	988.1074481	958.2816024
ALDH16A1	676.8413392	672.8507115	638.7370521	392.610831	341.5963104	346.8444269
ATXN7L3	1450.941405	1379.643447	1302.724324	2274.435159	2260.496389	2013.679644
LTBP1	686.765699	751.7160027	651.8297588	356.5086856	367.9611263	406.3034715
KIFC3	2146.639027	1934.695369	1737.589228	3195.942419	3036.539014	3047.276037
TMEM64	465.4524752	545.068974	533.9953978	264.4482149	260.20927	261.6197963
FAH	456.5205514	367.3724953	338.5399895	157.946886	143.2870429	185.3140224
ZBED2	387.0500327	392.3298659	429.2537436	204.879675	192.5777857	172.4312294
APOBEC3C	1472.774996	1490.454173	1460.771999	865.5489354	910.1592968	987.0201406

HPRT1	1339.788575	1333.721885	1304.594711	764.4629283	850.5518869	850.264338
CDC42BPA	482.3238869	598.9768946	638.7370521	1087.577129	1070.640785	989.9930928
CDK2	2622.015862	2662.452296	2554.013015	1763.589802	1717.151923	1801.609052
CSNK1G1	380.1029808	492.1593484	506.8747909	856.5233991	833.3574417	927.561096
KAT7	1112.520735	1182.979367	1141.87107	1688.67785	1852.414891	1828.365622
CRY2	328.4963098	313.4645748	302.0674492	136.2855988	150.164821	109.0082485
VAPB	2044.418121	2006.572597	1955.489277	2922.468668	3092.707535	2887.7276
MARVELD1	1728.823479	1807.911927	1653.421827	1057.79286	1161.198196	978.1012839
PYROXD1	263.987971	270.5378974	341.3455695	552.3628243	600.659284	599.5453665
EGR3	12.90166776	4.991474121	5.611160047	82.13238073	71.0703733	73.33282169
PKP2	1249.4769	1468.491686	1340.132058	2161.615954	2094.28342	2121.696909
ZNF281	1212.756769	1460.505328	1524.365146	2274.435159	2241.009352	2481.424129

ELK3	761.1983976	760.7006561	779.9512466	1224.765282	1221.951902	1214.946478
IQANK1	610.3481284	592.9871256	619.0979919	997.321766	989.2537445	1007.830806
ATXN1L	1093.664451	1437.544547	1393.438078	2486.535263	2124.087125	2275.299441
TIMM44	1231.613053	1220.91457	1066.120409	1796.984286	1934.948228	1891.788603
RRBP1	3602.542612	3494.031885	3398.492602	2401.695221	2448.48899	2264.398616
NDUFA8	1557.132055	1454.515559	1226.03847	774.3910183	851.6981832	813.5979271
LRRC8B	248.1089953	270.5378974	290.8451291	565.9011288	487.175946	538.1043538
TRMT2A	1092.672015	878.4994453	906.2023476	564.0960215	494.0537241	513.3297518
MT-ND1	22668.23025	20359.22465	20453.61357	29750.87545	38964.90531	34450.57045
ENC1	382.0878528	432.2616589	435.800097	805.0778419	699.2407695	760.084787
BUD31	1192.908049	1183.977662	1267.186977	1791.568964	2259.350093	2019.625549
TYMS	440.6415757	436.2548382	456.3743505	234.6639449	209.7722309	222.9714173

MN1	259.0257911	244.5822319	242.2150754	104.6962216	97.4351892	96.12545546
UBE2R2	1697.065528	1502.433711	1515.948406	2354.762432	2670.87048	2428.901973
HNRNPH3	2471.165593	2567.614288	2416.539594	1523.510535	1721.737108	1393.323612
ZC3HAV1	2118.85082	2281.103673	2670.912183	1446.793476	1373.26302	1456.746593
P4HA2	867.3890476	768.6870147	699.5246192	1303.287448	1495.916728	1281.342411
CCNI	3040.823846	2977.913461	3117.9346	4235.684206	4781.202049	4526.815263
RUNDC1	639.1287719	616.9462014	551.7640713	1040.64434	975.4981883	1052.42509
DHDDS	751.2740378	728.7552217	683.6263324	426.9078691	395.4722385	392.4296945
KIF2C	2453.301746	2303.06616	2409.058047	1547.879483	1584.181547	1640.078647
ZNF598	2147.631463	2072.460055	1953.61889	3041.605748	3187.850131	2996.735849
AVL9	1046.027524	1187.970841	1209.20499	1870.09113	1763.003776	1841.248415
MYNN	438.6567037	499.1474121	418.9666169	803.2727346	805.8463295	790.8052934

DSCC1	450.5659355	455.2224399	548.9584913	253.6175713	221.2351943	227.9263377
ATP5MC3	3816.908784	3223.493988	2987.942725	2004.571622	2135.550088	1859.086128
DEDD	500.1877345	408.3025831	426.4481636	789.7344301	875.7704064	775.9405322
EIF2A	7208.062532	7139.804583	7272.063421	9796.317148	11068.63749	10353.80164
DLGAP5	1194.892921	1261.844658	1319.557804	723.8480148	662.5592866	820.5348157
OPTN	1221.688693	1030.240259	1059.574056	1700.411047	1969.337118	1822.419717
LAMB2	1442.009481	1317.749168	1172.73245	2093.924432	2090.844531	2142.507574
NEFL	356.2845173	353.3963678	341.3455695	167.874976	157.0425991	165.4943408
NUDC	4368.703189	3808.494755	3624.809391	2408.91565	2646.798257	2269.353536
SLC27A4	1677.216808	1589.28536	1456.096032	999.1268733	944.5481871	995.9389973
PNRC2	1349.712934	1492.450762	1577.671167	2252.773871	2486.316769	2269.353536
AKAP13	1591.867314	1881.785744	1889.090549	2888.17163	2706.405667	2885.745632

RNF217	506.1423504	559.0451016	500.3284376	910.6766171	843.6741088	950.3537298
ANKRD13C	496.2179906	571.0246395	592.9125783	954.9017452	1025.935227	919.6332234
ZNF264	484.3087588	542.0740896	552.6992647	879.9897935	968.6204103	884.9487807
NCAPD3	1693.095784	1781.956261	1781.543315	1111.043524	888.3796662	1117.830039
FAM53B	467.4373472	385.3418022	416.1610368	217.5154259	187.9926003	151.6205638
LYSMD3	349.3374654	476.1866312	454.5039638	836.6672191	797.8222551	799.7241501
VOPP1	2437.42277	2305.062749	2237.917666	1578.566307	1546.353767	1467.647418
MEF2A	1353.682678	1580.300707	1555.226526	2333.101145	2283.422316	2423.947052
SERPINE1	2040.448377	1848.842015	1587.0231	2846.654163	3388.451991	3038.35718
ANXA1	15820.42198	15503.51862	14609.59038	21238.89212	23483.02689	21508.31841
CELF1	3621.398895	3734.620938	4046.581587	5747.461544	5397.909482	5589.150194
PODXL	676.8413392	800.6324491	758.4417997	435.9334054	369.1074226	406.3034715

DNAJB2	778.0698 093	626.9291 496	524.6434 644	1242.816 355	1508.525 988	1156.478 418
ZMYM4	548.8170 976	657.8762 892	603.1997 051	1068.623 503	968.6204 103	1092.064 453
UBXN2B	326.5114 378	381.3486 229	448.8928 038	720.2378 003	735.9222 525	765.0397 074
AKT1	2166.487 747	2116.385 027	1936.785 41	1363.758 542	1229.975 977	1361.612 122
IL7R	2031.516 454	2321.035 466	2355.752 027	1460.331 78	1326.264 869	1434.944 943
RCCD1	1402.312 041	1247.868 53	1157.769 356	792.4420 91	703.8259 549	732.3372 328
POLD3	788.9866 051	775.6750 784	787.4327 933	476.5483 19	401.2037 202	454.8616 913
ALDH2	129.0166 776	123.7885 582	119.7047 477	34.29703 811	26.36481 59	31.71149 046
MAML1	999.3830 331	1080.155	1187.695 543	1820.450 681	1680.470 44	1760.978 705
SNX11	510.1120 943	498.1491 173	466.6614 773	909.7740 635	934.2315 2	777.9225 004
SACS	701.6522 387	819.6000 507	880.0169 341	427.8104 227	365.6685 336	459.8166 117
ARL4A	461.4827 313	409.3008 779	404.9387 168	719.3352 466	815.0167 002	751.1659 303

UIMC1	676.8413392	658.874584	678.0151724	1042.449448	1105.029675	1129.721848
RCOR3	538.8927378	531.0928465	541.4769446	929.6302434	853.9907759	919.6332234
YY1	2975.323072	2963.937333	2897.228971	4219.438241	4169.079801	4183.934773
KPNA1	654.0153116	726.7586321	827.646107	1323.143628	1278.120423	1202.063685
KDM1A	3429.858751	3326.318354	3359.214482	2326.78327	2284.568613	2383.316705
SLC25A45	51.60667102	66.88575323	59.85237384	167.874976	185.7000076	186.3050065
NAA38	884.2604593	735.7432855	634.9962787	393.5133846	377.131497	373.600997
TOX2	278.8745107	293.4986783	274.9468423	547.8500561	638.4870633	495.4920385
CCDC174	265.972843	236.5958733	243.1502687	476.548319	585.7574315	464.7715321
RAB23	132.9864215	161.7237615	177.6867348	370.0469901	381.7166824	325.0427772
CDC42EP3	776.0849373	795.6409749	914.6190877	1283.431268	1429.43154	1508.277765
THEM4	88.32680233	97.83289278	114.0935876	12.63575088	20.63333418	21.80164969

LDAH	383.0802887	397.3213401	490.9765041	769.8782501	787.505588	788.8233252
UBE2C	3068.612054	2780.251086	2717.67185	1858.357933	1821.46489	1947.283711
RGS16	50.61423504	39.93179297	37.40773365	167.874976	127.2388941	162.5213886
EEF2KMT	253.0711752	215.631682	204.8073417	85.74259527	81.38704039	83.24266246
TRIM33	1008.314957	1196.955494	1195.17709	1951.320958	1720.590812	1944.310759
NDUFB7	1315.970111	1010.274362	977.2770416	593.8802914	600.659284	578.7347009
CIC	1406.281785	1477.47634	1255.964657	2213.964065	2087.405642	2195.02973
HENMT1	641.1136439	666.8609426	647.1537921	369.1444365	366.8148299	369.6370607
LNX2	390.0273406	468.2002726	474.143024	778.9037865	793.2370697	808.6430068
RCC1	3413.979775	3241.463294	2986.072339	2103.852522	1992.263045	2204.939571
CARD19	590.4994088	507.1337707	447.9576104	227.4435159	278.5500115	208.1066562
NF1	2323.292632	2845.140249	3020.674492	5000.147135	4096.863132	5331.494334

IST1	2889.973577	2916.019182	2779.39461	4056.076033	4288.294621	4048.169954
RAB7A	4614.827313	4302.650693	4392.603124	6137.364714	7095.574366	6550.404748
BCL7B	1141.301378	1032.236848	1053.027702	1574.053538	1941.826006	1856.113176
CCT5	15034.41268	13967.14289	13897.90824	9798.122255	10219.2319	10331.99999
RBX1	1099.619067	924.4210073	820.1645603	487.3789626	530.735207	514.3207359
JRK	701.6522387	703.7978511	704.2005859	403.4414746	342.7426067	418.1952805
ASXL1	2360.012763	2423.859833	2347.335286	3442.339562	3351.770508	3476.372142
TAF1D	2918.754221	3059.773636	3139.444046	4277.201673	4759.422418	4410.870126
NME4	1810.20323	1521.401312	1391.567692	845.6927554	973.2055956	875.0389399
SPIN1	1970.977859	2112.391848	2278.130979	3207.675617	3226.824207	3147.365428
CTIF	340.4055416	342.4151247	286.1691624	703.0892812	677.461139	547.0232105
SGMS2	570.6506892	687.8251339	825.7757203	1301.482341	1236.853755	1318.999806

CAPN8	17.86384766	12.97783272	16.83348014	101.9885607	79.0944477	92.16151915
GPATCH2	339.4131056	371.3656746	420.8370036	719.3352466	693.5092878	664.9503156
VAMP3	1911.4317	1857.826668	1882.544196	1208.519316	1264.364867	1275.396507
GPRIN1	357.2769532	363.379316	377.8181099	191.3413705	162.7740808	159.5484364
TUFM	6774.368008	6066.637647	5657.919714	4051.563265	4110.618688	4103.665062
GOLGA4	1660.345397	1907.741409	1955.489277	2813.259678	2778.622337	3047.276037
IPO7	5363.124042	6058.651288	6414.491127	8706.934911	8863.163328	9270.65604
CFH	1101.603939	1126.076562	1182.084383	675.1101185	672.8759536	742.2470736
KLHL22	154.8200131	191.6726063	172.0755748	59.56853987	41.26666837	58.46806054
ZNF740	736.3874981	740.7347596	712.617326	1209.42187	1208.196346	1108.911182
DEF6	317.579514	314.4628696	304.8730292	141.7009206	151.3111173	112.9721848
SSBP3	721.5009583	665.8626478	576.0790982	355.606132	333.572236	353.7813155

PTPRH	793.948785	669.8558271	644.3482121	1215.739746	1391.603761	1136.658736
JPH1	287.8064346	298.4901525	264.6597156	130.870277	102.0203746	121.8910415
SNX2	1194.892921	1312.757694	1256.899851	1855.650272	2231.838981	1973.049297
ZNF84	398.9592644	451.2292606	438.605677	685.0382085	949.1333724	872.0659877
GFM1	1737.755403	1921.717537	1916.211156	2797.013713	2691.503815	2914.48417
IPMK	344.3752855	430.2650693	437.6704837	824.9340218	811.5778112	682.788029
CBLL1	471.4070911	534.087731	533.9953978	882.6974545	839.0889235	909.7233826
BDNF	120.0847537	96.83459795	93.51933412	296.0375921	268.2333444	230.8992899
APPBP2	416.8231121	476.1866312	513.4211443	872.7693645	773.7500319	879.0028762
CSTB	1868.756953	1518.406428	1383.150952	876.379579	954.8648541	778.9134845
DDX59	330.4811817	347.4065988	311.4193826	600.1981669	593.7815059	593.5994621
NUDT22	696.6900588	585.9990618	593.8477717	342.0678274	345.0351994	316.1239205

UGDH	750.2816018	771.6818992	833.257267	440.4461736	459.6648338	467.7444843
ODR4	505.1499144	568.029755	537.7361712	889.0153299	891.8185553	915.6692871
RASSF1	404.9138803	347.4065988	338.5399895	667.8896895	665.9981756	647.1126022
FAM162A	796.9260929	740.7347596	719.1636794	1153.463545	1364.092649	1237.739112
ATP5MF	4167.238685	3400.192171	2804.64483	1843.014521	2041.553788	1825.39267
UPF3B	772.1151934	759.7023613	832.3220737	1204.909102	1282.705608	1302.153077
FXYD3	131.0015495	166.7152357	158.0476747	42.42002082	51.58333546	38.648379
MBLAC2	203.4493761	241.5873475	294.5859025	90.25536344	72.21666964	99.09840769
TRIM66	1046.027524	1155.027112	1215.751344	668.7922431	638.4870633	726.3913284
NFKBIZ	372.163493	392.3298659	457.3095439	743.7041948	721.0204001	730.3552647
MRPS2	1296.121391	1195.957199	1150.28781	743.7041948	755.4092904	762.0667551
NUP62	2098.009664	1905.74482	1898.442483	1213.934638	1236.853755	1342.783424

PIGX	411.8609322	445.2394916	430.188937	204.879675	208.6259345	232.8812581
GDF11	778.0698093	719.7705683	793.0439534	447.6666027	414.9592764	456.8436595
CYTH3	1364.599474	1468.491686	1391.567692	2211.256404	2117.209346	2048.364087
HIST2H2BE	163.7519369	173.7032994	171.1403814	341.1652738	507.8092802	359.7272199
HLCS	309.6400261	425.2735951	400.26275	686.8433158	735.9222525	757.1118348
BRPF1	555.7641495	502.1422966	513.4211443	883.6000081	900.988926	851.2553221
MEX3C	457.5129873	526.1013724	538.6713645	894.4306517	847.1129979	871.0750036
GUSB	636.151464	633.9172134	523.7082711	324.9193084	293.4518639	320.0878568
KDM5A	1140.308942	1270.829311	1300.853938	1911.608598	1861.585262	1984.941106
TRAF6	374.1483649	401.3145194	405.8739101	665.1820286	692.3629915	706.5716468
POLA2	363.2315691	370.3673798	357.2438563	194.951585	153.60371	149.6385956
SLC35B2	1665.307576	1591.28195	1457.031226	1018.0805	982.3759664	856.2102425

MSANTD3	1089.694707	1008.277772	951.091628	1603.837808	1805.416741	1546.926144
LCLAT1	472.3995271	498.1491173	521.8378844	917.8970462	777.1889209	1019.722615
ABHD11	516.0667102	451.2292606	431.1241303	208.4898895	259.0629736	214.0525606
BCL7C	526.983506	495.1542328	431.1241303	233.7613913	264.7944554	226.9353536
PRPF19	5413.738278	4769.85267	4762.939687	3313.274392	3371.257546	3425.831954
KCNG1	942.8141822	871.5113816	876.2761607	1424.229635	1427.138948	1355.666217
RABL6	3071.589362	2490.745587	2389.418987	1643.550168	1626.594511	1429.990023
ZBED1	442.6264476	415.2906469	382.4940766	202.1720141	202.8944528	211.0796084
ALOXE3	90.31167429	47.91815156	41.14850701	197.6592459	231.5518614	201.1697676
THRB	206.4266841	226.6129251	208.5481151	484.6713017	372.5463116	528.194513
TSR3	912.0486667	817.6034611	774.3400865	486.4764089	491.7611314	403.3305193
MAPK7	365.2164411	327.4407024	347.8919229	625.4696686	615.5611365	612.4281595

GLI4	377.1256729	319.4543438	288.9747424	133.5779379	154.7500064	140.7197389
TROAP	1012.284701	1000.291414	916.4894744	627.2747759	555.9537266	548.0141945
BCL2L11	204.4418121	214.6333872	224.4464019	74.91195166	85.97222576	86.21561469
CCND1	7396.625368	7599.020202	7443.203803	5519.115474	5284.426144	5353.295983
FAM168A	1073.815732	1185.974251	1173.667643	1713.949352	1743.516738	1837.284479
RELB	968.6175177	827.5864093	808.9422402	1580.371414	1718.298219	1315.03587
TBC1D22B	351.3223373	331.4338817	341.3455695	570.4138969	628.1703962	659.9953952
TRIM29	2201.223006	1948.671497	1949.878116	1280.723607	1307.924128	1358.639169
CYSTM1	656.0001836	633.9172134	513.4211443	1000.931981	1238.000051	1057.38001
PLCB3	4837.132972	4598.145961	4272.898376	3060.559374	3156.90013	3164.212158
ADAMTSL4	399.9517004	434.2582486	375.9477232	203.0745677	193.7240821	201.1697676
HPS3	1993.803886	2248.159944	2239.788052	1404.373455	1291.875979	1455.755609

GALNT1	853.4949438	859.5318437	918.3598611	553.2653779	514.6870582	494.5010544
ASXL2	821.7369924	1062.185693	1099.787369	1880.01922	1591.059325	1810.527909
CTCF	1209.779461	1212.928211	1219.492117	1830.378771	1758.418591	1890.797619
IDH1	2221.071726	2096.419131	2190.222805	3073.195125	3826.337195	3346.553228
NUP98	2624.000734	2856.121492	2862.626817	4235.684206	3853.848307	4416.816031
HSPB1	6608.631199	5681.295845	5022.923436	3243.777762	3840.092751	3278.175326
FBXO45	1648.436165	1735.036405	1552.420946	2590.328931	2384.296395	2465.568383
MRTO4	1850.893105	1532.382555	1632.847574	1022.593268	1035.105598	1063.325915
MSH5	208.4115561	194.6674907	179.5571215	71.30173712	69.92407695	71.35085354
UBE2D3	3505.283886	3603.844316	3413.455695	5080.474408	5524.002079	4830.056391
CEP350	796.9260929	1075.163526	1168.991677	1990.130764	1669.007476	2146.471511
ANGEL1	477.361707	506.1354759	511.5507577	281.5967339	210.9185272	256.6648759

RACGAP1	1719.891555	1673.142125	1720.755748	1114.653738	997.2778189	1161.433338
SMG9	1244.51472	1120.086793	1088.565049	1827.67111	1717.151923	1828.365622
ANXA8	1464.835508	1478.474635	1435.521779	907.9689562	994.9852262	928.5520801
SAT1	3959.819565	3649.765877	3494.817516	5335.897087	6442.185451	5510.862452
SDE2	727.4555742	818.6017559	905.2671543	1374.589185	1444.333393	1286.297332
10-Sep	1507.510255	1553.346747	1661.838567	2335.808806	2361.370468	2321.875692
SLTM	1609.731162	1573.312643	1697.375914	2294.291339	2620.433441	2525.027428
SLC12A7	1071.83086	1112.100434	923.9710211	1689.580404	1636.911179	1637.105695
SAPCD2	1074.808168	1057.194219	990.3697484	684.1356549	531.8815034	557.9240353
FBXL15	260.0182271	210.6402079	227.2519819	98.37834615	90.55741114	81.26069431
NUAK1	377.1256729	408.3025831	357.2438563	646.2284022	671.7296573	702.6077105
MYO1B	6148.140904	6882.244518	7207.535081	9943.43339	9585.330024	10291.36964

PLOD1	3063.649874	2804.210161	2702.708756	1923.341795	1910.876005	1919.536157
PSME4	1861.809901	2126.367976	2112.601758	3068.682357	2955.151974	3199.887584
POLR3K	573.6279971	531.0928465	466.6614773	298.745253	231.5518614	236.8451944
GAPVD1	1648.436165	1789.94262	1872.257069	2768.131997	2535.607512	3016.55553
DUS1L	2749.047668	2511.709778	2298.705233	1639.0374	1578.450065	1662.871281
BTN3A2	555.7641495	461.2122088	534.9305912	277.0839658	221.2351943	275.4935734
FAM102A	930.9049504	886.4858039	901.5263809	1374.589185	1603.668585	1390.35066
TGFBI	6748.564672	6458.967513	6198.461466	4444.174096	4516.407593	4707.174365
LAMC2	10778.84719	10723.683	9818.59489	14278.3985	15221.66915	14758.72586
SLC26A2	465.4524752	556.0502171	595.7181584	295.1350385	243.0148248	270.538653
CDH24	981.5191854	931.409071	914.6190877	600.1981669	539.9055778	572.7887965
B4GALT2	2543.61342	2098.415721	2068.647671	1393.542812	1404.213021	1378.458851

SLC9A3R2	722.4933943	620.9393807	599.4589317	337.5550593	372.5463116	348.8263951
RAB11FIP4	389.0349046	450.2309657	411.4850701	203.0745677	224.6740833	194.2328791
PHF19	984.4964934	916.4346487	934.2581479	604.7109351	533.0277997	570.8068283
THOP1	2312.375836	2078.449824	2014.406457	1448.598583	1234.561162	1351.702281
DLL1	549.8095336	511.12695	483.4949574	295.1350385	252.1851956	261.6197963
SPPL3	1451.933841	1506.42689	1530.9115	2246.455996	2440.464915	2132.597734
STAT3	2808.593827	2791.232329	2881.330684	4064.199016	3940.966829	3964.927292
PRDM10	258.0333551	341.4168299	329.1880561	588.4649696	566.2703937	641.1666978
CD55	3047.770898	2903.041349	2986.072339	4118.352234	4472.848332	4218.619215
HMCES	1516.442179	1431.554778	1382.215758	941.3634407	925.0611492	779.9044685
SH3RF1	503.1650425	470.1968622	497.5228575	780.7088938	862.0148503	829.4536724
SNX18	1042.05778	1169.003239	1053.027702	1767.200016	1613.985252	1695.573756

PLEKHN1	288.7988705	261.553244	200.131375	92.96302434	75.65555867	105.0443122
SNRPF	2095.032356	1757.997186	1724.496521	1092.992451	1223.098199	1004.857854
CLK3	784.0244252	823.59323	674.274399	1208.519316	1421.407466	1248.639937
VWA8	667.9094154	714.7790942	782.7568266	1241.913801	1094.713008	1335.846536
RAPGEF2	245.1316874	302.4833317	277.7524223	565.9011288	479.1518716	551.9781308
FGF21	5.954615887	1.996589649	1.870386682	56.86087897	69.92407695	52.52215608
HDAC9	421.785292	612.9530221	595.7181584	1003.639641	1106.175971	996.9299814
CLDND1	922.9654625	978.3289278	1121.296816	1534.341178	1830.635261	1663.862265
NR2F2	653.0228756	623.9342652	627.514732	1003.639641	1007.594486	1028.641472
NLRP2	3464.59401	3182.5639	3211.453934	2278.045373	2242.155648	2285.209281
AKR1A1	1731.800787	1526.392786	1368.187858	889.0153299	976.6444847	911.7053508
ADGRL1	1700.042836	1700.096086	1750.681935	2701.343028	2471.414917	2453.676574

KRAS	1643.473985	1764.985249	1951.748503	2847.556717	2659.407517	2700.43161
ARPIN	1029.156113	1083.149884	1199.853057	676.0126722	655.6815085	690.7159016
FOXN2	332.4660537	406.3059935	503.1340176	810.4931637	782.9204026	826.4807201
FZD4	216.3510439	222.6197458	229.1223686	84.84004163	96.28889286	88.19758285
LCAT	86.34193037	76.86870147	61.72276052	195.8541387	222.3814906	196.2148472
NDUFB3	823.7218644	721.7671579	730.3859995	410.6619037	448.2018703	309.187032
FAM111A	1097.634195	1104.114076	1379.410178	738.2888729	614.4148401	660.9863793
ITGB8	309.6400261	359.3861367	423.6425836	626.3722223	771.4574392	735.3101851
PTK2	1712.944504	1857.826668	1886.284969	2690.512384	2596.361218	2861.962014
ENO2	934.8746943	762.6972457	641.5426321	1380.004507	1557.816731	1358.639169
ZNF316	1056.94432	961.3579158	896.8504142	1731.097871	1728.614886	1424.044119
AKR1B10	139.9334734	128.7800323	151.5013213	36.10214538	46.99815008	41.62133123

MAGED2	1011.292265	982.3221071	918.3598611	592.0751842	599.5129877	602.5183188
PIM1	681.8035191	683.8319546	670.5336257	1167.904403	1313.65561	1010.803758
PRDX2	2397.725331	2103.407195	2200.509932	1487.40839	1514.25747	1382.422787
FDXR	789.979041	664.864353	670.5336257	408.8567964	387.4481641	397.3846148
VPS50	576.6053051	594.9837153	625.6443453	923.312368	1022.496338	994.9480132
TNIK	248.1089953	228.6095148	259.9837489	499.1121598	436.7389069	475.6723569
TRIM14	1688.133604	1791.93921	1779.672928	1211.226977	1052.300043	1139.631688
HMGN1	5505.042388	5240.049533	5381.102485	3887.298503	3871.042752	3562.587756
TMED9	3686.89967	3264.424075	3064.628579	2076.775913	2242.155648	1752.059848
GGH	1148.24843	1099.122602	1096.046596	725.6531221	696.9481769	600.5363506
PSD4	738.37237	722.7654528	751.8954463	423.2976545	448.2018703	332.9706498
CDIPT	1148.24843	901.4602263	877.2113541	543.3372879	538.7592815	548.0141945

TADA2A	307.6551542	357.3895471	315.160156	562.2909142	623.5852109	595.5814302
KLHDC10	641.1136439	606.9632531	704.2005859	1133.607365	1058.031525	1022.695567
ETFB	757.2286537	621.9376755	568.5975515	345.678042	342.7426067	335.9436021
ARHGEF19	1077.785476	966.3493899	918.3598611	602.9058278	516.9796509	606.4822551
GTF2H1	1007.322521	917.4329435	1000.656875	1426.937296	1556.670434	1603.412236
RFFL	251.0863032	271.5361922	299.2618692	505.4300353	530.735207	500.4469588
FAM206A	765.1681415	703.7978511	771.5345065	1223.862728	1125.663009	1214.946478
MTHFD1L	1716.914248	1536.375735	1639.393927	2334.003699	2533.314919	2448.721654
NDUFA4L2	2724.236768	2410.882001	1966.711597	1319.533414	1427.138948	1413.143294
PACSIN2	1616.678213	1540.368914	1484.151833	2185.984903	2442.757508	2345.65931
ARL6IP5	1008.314957	1006.281183	1109.139303	629.0798832	668.2907683	638.1937455
POLR2K	1011.292265	1020.25731	953.8972081	596.5879523	651.0963231	597.5633984

ARF6	2197.253262	2198.245203	2125.694465	3066.87725	3260.066801	3051.239973
HADH	609.3556925	648.8916358	644.3482121	334.8473984	373.692608	374.5919811
BTBD10	1175.044202	1081.153295	1136.25991	1627.304203	1791.661185	1771.87953
RNF10	4120.594194	3775.551025	3942.775127	5401.783502	5955.009505	5648.609238
HERC6	528.968378	486.1695794	549.8936846	283.4018412	292.3055676	276.4845575
CTSL	1409.259093	1197.953789	1246.612724	805.0778419	820.748182	749.1839621
MMP2	80.38731448	82.85847041	62.65795386	12.63575088	9.170370748	10.90082485
ADAM15	2552.545344	2268.125841	2122.888885	1557.807573	1444.333393	1447.827736
RFC2	639.1287719	547.0655637	611.6164452	342.9703811	332.4259396	324.0517932
RTKN	1106.566119	1090.137948	1010.008809	663.3769213	628.1703962	696.6618061
GLA	712.5690345	701.8012615	754.7010264	443.1538345	404.6426093	427.1141371
LONRF3	112.1452659	155.7339926	145.8901612	318.6014329	312.9389018	300.2681753

TPI1	30788.34145	26366.9629	23840.88385	16901.21936	18453.07854	15666.46727
HAUS5	865.4041756	753.7125923	789.30318	476.548319	490.614835	454.8616913
NDUFA2	683.7883911	598.9768946	568.5975515	354.7035783	342.7426067	291.3493186
TMEM177	304.6778462	236.5958733	261.8541355	115.5268652	119.2148197	103.062344
PARP11	107.183086	111.8090203	121.5751344	251.812464	287.7203822	248.7370033
FANCI	2847.29883	3216.505924	3297.491721	2040.673767	1765.296369	2135.570686
AURKAIP1	1849.900669	1739.029584	1498.179733	1066.818396	1076.372267	909.7233826
WDR46	1380.47845	1243.875351	1213.880957	825.8365755	821.8944783	739.2741214
DDB2	1658.360525	1525.394491	1454.225646	999.1268733	1027.081524	963.2365228
DTD2	385.0651607	401.3145194	455.4391572	213.0026577	190.285193	218.0164969
NDUFS6	2929.671017	2261.137777	1949.878116	1318.63086	1340.020426	1314.044886
PHF3	1301.083571	1556.341631	1637.52354	2389.962024	2279.983427	2499.261842

BRSK1	58.55372289	75.87040664	51.43563377	171.4851905	207.4796382	168.4672931
CPPED1	87.33436635	90.84482901	71.07469393	12.63575088	16.04814881	15.85574523
GRK5	614.3178724	606.9632531	525.5786578	331.2371838	319.8166798	312.1599842
NUP85	1608.738726	1570.317759	1417.753105	977.4655861	989.2537445	1008.82179
MUC1	161.7670649	154.7356978	137.4734212	304.1605748	331.2796433	326.0337613
UBE2B	1129.392147	1013.269247	1003.462455	1663.406348	2116.06305	1608.367157
SMIM30	1262.378568	1228.900929	1421.493879	837.5697727	833.3574417	732.3372328
C1orf198	533.9305579	476.1866312	501.2636309	285.2069485	270.5259371	265.5837326
PITRM1	1320.932291	1201.946968	1174.602837	709.4071566	758.8481794	820.5348157
CBARP	348.3450294	308.4731007	347.8919229	653.4488313	562.8315047	595.5814302
HELLS	1187.94587	1361.67414	1408.401172	861.0361672	762.2870684	806.6610386
MCTP1	64.50833878	99.82948243	100.0656875	222.9307477	234.9907504	235.8542103

ALYREF	2432.46059	2256.146303	2253.815952	1587.591843	1566.987102	1343.774408
OXCT1	1675.231936	1740.027879	1691.764754	1203.103995	1054.592636	1079.18166
TEAD2	618.2876163	675.845596	611.6164452	347.4831492	369.1074226	376.5739492
CAD	2890.966013	3020.840138	3002.905819	2065.945269	1720.590812	2047.373103
RBBP7	2157.555823	2101.410605	2058.360544	1450.40369	1411.090799	1469.629386
C12orf66	552.7868415	605.9649583	604.1348984	314.0886648	267.087048	355.7632836
SRGAP3	245.1316874	259.5566543	246.8910421	117.3319725	108.8981526	81.26069431
MEGF8	619.2800523	520.1116034	651.8297588	330.3346302	334.7185323	292.3403027
SKIV2L	1898.530032	1796.930684	1608.532547	1133.607365	1150.881529	1146.568577
ATRX	856.4722518	1026.247079	1136.25991	1754.564265	1550.938953	1946.292727
THEM6	408.8836243	328.4389972	306.7434159	157.0443324	144.4333393	170.4492612
ALG1	522.0213261	518.1150138	467.5966706	288.817163	239.5759358	271.5296371

THOC5	1451.933841	1345.701423	1350.419185	889.0153299	847.1129979	939.4529049
ATP5MC1	3023.952435	2377.938271	2147.203911	1514.484999	1486.746358	1380.440819
APOBEC3B	523.0137621	526.1013724	591.977385	303.2580212	309.5000127	232.8812581
FSTL3	1385.44063	1140.052689	1011.879195	655.2539386	671.7296573	684.7699971
SEMA3A	466.4449112	468.2002726	591.977385	879.0872399	860.868554	944.4078253
TUBA1C	7240.812919	6723.515641	6520.167975	4244.709743	4794.957605	4892.488388
ZNF638	1005.337649	1122.083382	1191.436317	1766.297463	1634.618586	1983.950122
PLAA	1200.847537	1338.713359	1302.724324	1859.260487	1932.655635	1966.112409
TAOK3	1167.104714	1264.839542	1180.213997	1742.831068	1801.977852	1982.959138
GDAP1	267.9577149	240.5890526	286.1691624	466.620229	505.5166875	514.3207359
DHRS1	580.575049	515.1201293	458.2447372	284.3043948	262.5018627	267.5657008
FAM83G	960.6780298	973.3374537	806.1366601	1460.33178	1589.913028	1403.233453

OGDH	3420.926827	3295.371215	3012.257752	2185.082349	2125.233421	2294.128138
PECAM1	77.41000653	76.86870147	83.23220737	14.44085815	3.438889031	15.85574523
SLC12A9	1603.776546	1374.651973	1363.511892	954.9017452	889.5259626	838.3725291
ZNF592	857.4646878	872.5096764	807.0718535	1278.015946	1274.681534	1426.026087
SLC25A25	574.6204331	565.0348705	461.9855106	987.393676	1014.472264	837.381545
LRRC58	1381.470886	1648.184755	1803.987955	2688.707277	2496.633436	2572.594664
CCNG2	458.5054233	524.1047827	461.9855106	859.23106	849.4055905	754.1388825
ZDHHC8	1537.283335	1561.333105	1368.187858	1003.639641	879.2092955	936.4799527
STAG1	557.7490214	626.9291496	730.3859995	1105.628202	1044.275969	1107.920198
TBCD	1176.036638	1142.049279	1125.972783	728.360783	692.3629915	773.9585641
FZD1	520.0364542	604.9666635	645.2834054	352.8984711	278.5500115	285.4034142
DENND4A	451.5583715	584.0024722	555.5048447	910.6766171	875.7704064	1051.434106

LACC1	181.6157846	189.6760166	226.3167886	422.3951009	369.1074226	413.2403601
MKKS	212.3813	176.6981839	198.2609883	396.2210455	426.4222398	359.7272199
MEGF9	318.57195	358.3878419	370.3365631	170.5826369	162.7740808	178.3771338
EYA2	396.9743925	356.3912523	340.4103762	649.8386168	607.5370621	655.0404748
C1orf61	180.6233486	208.6436183	255.3077822	76.71705892	90.55741114	72.34183761
HDHD5	1782.415022	1572.314348	1375.669405	981.9783542	976.6444847	886.9307488
AP4E1	432.7020878	565.0348705	554.5696513	993.7115515	858.5759613	908.7323985
HFE	323.5341299	335.427061	400.26275	174.1928514	175.3833406	132.7918663
NBEA	61.53103084	79.86358594	67.33392057	218.4179795	155.8963027	216.0345288
LUC7L	538.8927378	508.1320655	509.680371	870.0617036	870.0389247	802.6971023
GALK1	359.2618252	283.5157301	304.8730292	152.5315642	112.3370417	147.6566275
DELE1	1057.936756	1111.102139	1098.852176	1623.693988	1638.057475	1589.538459

EFL1	355.2920813	330.4355868	317.0305427	570.4138969	583.4648389	601.5273347
GSTO1	1202.832409	973.3374537	952.9620147	546.9475025	622.4389145	394.4116626
FZD5	374.1483649	393.3281608	411.4850701	210.2949968	201.7481565	197.2058313
CHD1	1195.885357	1552.348452	1574.865587	2351.152218	2262.788982	2600.342218
PPP1R12B	341.3979775	408.3025831	463.8558972	791.5395374	663.7055829	857.2012265
APC	446.5961915	587.9956515	622.8387653	996.4192124	928.5000382	1155.487434
DOLK	490.2633747	412.2957624	375.9477232	193.1464778	234.9907504	187.2959905
IRF1	642.1060798	574.0195239	642.4778254	1019.885607	1041.983376	946.3897935
TMEM246	926.9352065	979.3272226	995.0457151	592.0751842	616.7074328	618.374064
SIRT1	275.8972028	309.4713955	362.8550164	580.3419869	572.0018754	598.5543825
RPA2	1203.824845	1044.216386	1026.842289	602.0032741	649.9500268	711.5265672
COQ4	1265.355876	1308.764515	1258.770237	725.6531221	870.0389247	820.5348157

FOXL1	147.8729612	164.718646	127.1862944	40.61491355	53.87592815	43.60329938
PPP4R3B	1128.399711	1307.76622	1388.762112	1978.397567	2074.796382	1930.436982
CBX3	5203.34185	4916.602009	4903.218688	3526.27705	3681.903855	3230.608091
VPS8	529.960814	639.9069823	630.320312	1018.0805	935.3778163	1065.307883
TMEM268	400.9441364	391.3315711	405.8739101	682.3305476	685.4852134	647.1126022
CEP55	1528.351411	1692.109727	1670.255307	1111.043524	976.6444847	1077.199692
UBE3A	1090.687143	1277.817375	1359.771118	2011.792051	1879.926003	1984.941106
ITPKC	375.1408009	371.3656746	372.2069498	642.6181877	678.6074354	600.5363506
VSIR	252.0787392	234.5992837	248.7614288	458.4972463	596.0740986	431.0780735
IGFBP3	201.4645042	212.6367976	195.4554083	370.9495437	444.7629813	386.48379
CIRBP	3073.574234	2705.378974	2683.069696	1796.984286	1881.0723	1401.251485
TMEM38B	943.8066181	1027.245374	1067.055602	639.0079732	593.7815059	651.0765385

NCK1	369.186185	460.213914	450.7631905	788.8318765	800.1148478	689.7249175
RPP25L	787.0017331	633.9172134	606.0052851	333.0422911	403.4963129	334.952618
FLYWCH1	1198.862665	1136.05951	1178.34361	1770.810231	2024.359343	1677.736042
ZNF655	2419.558922	2720.353396	2796.22809	3711.300545	3928.357569	4055.106843
WFS1	299.7156663	264.5481284	287.1043558	120.942187	85.97222576	142.7017071
NPC2	1231.613053	1082.151589	972.6010749	662.4743677	678.6074354	579.725685
ERC1	926.9352065	1097.126012	1082.018696	1697.703386	1509.672284	1783.771338
COPRS	778.0698093	698.806377	662.1168856	411.5644573	421.8370544	419.1862645
RTN4	4260.527667	4284.681386	4128.878602	5862.085855	6239.290998	5650.591207
TMEM170A	347.3525934	455.2224399	476.0134107	787.0267692	813.8704039	733.3282169
SLU7	1194.892921	1132.066331	1219.492117	1663.406348	2036.968602	1862.059081
POMGNT1	1554.154747	1478.474635	1344.808025	981.9783542	886.0870735	930.5340482

GATD3B	223.2980958	188.6777218	241.279882	95.67068525	81.38704039	81.26069431
PBRM1	574.6204331	673.8490064	698.5894259	1036.131572	1045.422265	1105.93823
CHMP6	244.1392514	211.6385027	226.3167886	94.76813161	104.3129673	86.21561469
ARCN1	2668.660353	2732.332934	2872.913944	3772.674192	3897.407568	4039.251097
CEP170	463.4676032	586.9973567	634.9962787	1028.911143	925.0611492	1024.677536
RHOQ	575.6128691	611.9547273	665.857659	1024.398375	1126.809306	936.4799527
RASA2	381.0954168	411.2974676	400.26275	640.8130804	677.461139	696.6618061
ANKRD40	1165.119842	1172.996419	1284.955651	1705.826369	1891.388967	1947.283711
CNPY4	230.2451476	238.592463	232.863142	114.6243116	88.26481845	89.18856692
EDA2R	4.962179906	2.994884473	5.611160047	64.08130804	63.04629889	42.61231531
LZTFL1	165.7368089	186.6811321	214.1592751	355.606132	419.5444617	382.5198537
HILPDA	591.4918448	508.1320655	409.6146835	960.317067	1032.813006	833.4176087

TUSC1	597.4464607	639.9069823	577.0142915	352.8984711	358.7907555	281.4394778
TMED7	1822.112462	2210.224741	2317.4091	3349.376537	3472.131625	3135.473619
ZNF580	729.4404462	644.8984565	750.960253	384.4878483	450.494463	373.600997
CAPN15	2040.448377	1992.596469	1834.849335	1385.419829	1239.146347	1252.603873
PRAME	2461.241233	2294.081506	2190.222805	1464.844549	1460.381542	1660.889313
ZSCAN29	437.6642677	387.3383918	442.3464504	708.504603	683.1926207	767.0216755
NDRG4	198.4871962	200.6572597	233.7983353	98.37834615	69.92407695	73.33282169
KIAA0895	220.3207878	193.6691959	238.474302	442.2512809	463.1037228	382.5198537
GON4L	1016.254445	993.3033501	1090.435436	1509.97223	1546.353767	1615.304045
MOSMO	395.9819565	468.2002726	495.6524708	774.3910183	764.5796611	785.850373
CEP131	874.3360994	889.4806884	812.6830135	567.706236	491.7611314	460.8075958
ESCO1	393.0046486	375.3588539	417.0962302	671.499904	659.1203975	663.9593315

EIF6	4354.809086	3612.828969	3419.066856	2530.760391	2404.929729	2083.04853
STX1A	345.3677215	298.4901525	273.0764556	574.9266651	574.2944681	527.2035289
H2AFZ	6598.706839	5860.988913	5434.408506	3973.041099	4178.250172	3563.578741
KNOP1	488.2785028	521.1098983	550.828878	287.9146094	283.1351968	301.2591594
CARHSP1	723.4858303	810.6153973	701.3950059	475.6457653	410.374091	384.5018218
RPUSD2	423.770164	315.4611645	327.3176694	118.2345261	176.5296369	163.5123727
USP8	908.0789228	1041.221502	973.5362682	1475.675192	1443.187096	1577.64665
TRA2A	1546.215259	1438.542842	1537.457853	2199.523207	2437.026026	2125.660845
DDX10	937.8520022	963.3545054	933.3229545	1359.245773	1539.475989	1424.044119
LMBRD1	321.5492579	336.4253558	331.0584428	529.7989834	674.02225	590.6265098
IGF2BP3	680.8110831	874.506266	841.6740071	1328.55895	1246.024125	1534.043351
AGR2	90.31167429	85.85335489	97.26010749	194.951585	301.4759383	220.9894492

ZNF165	80.38731448	74.87211182	74.8154673	183.2183878	272.8185298	181.3500861
CAPG	2158.548259	1794.934094	1582.347133	1107.433309	1155.466714	972.1553795
SP100	1447.964097	1321.742347	1323.298578	1944.100529	2082.820456	2016.652597
RAC2	3956.842257	3637.78634	3271.306308	2226.599816	2527.583437	2396.199498
UBALD2	1722.868863	1489.455878	1277.474104	2371.008398	2559.679735	2362.506039
NAE1	1860.817465	1904.746525	1934.915023	1260.867427	1284.998201	1355.666217
CMAS	1872.726697	1873.799385	1898.442483	1287.041483	1329.703758	1148.550545
TIPIN	387.0500327	397.3213401	445.1520304	211.1975505	216.6500089	218.0164969
HES7	487.2860668	469.1985674	376.8829165	178.7056196	253.3314919	200.1787835
FAM234A	565.6885093	426.27189	415.2258435	255.4226785	214.3574162	202.1607517
COQ8A	643.0985158	643.9001616	582.6254516	368.2418828	354.2055701	369.6370607
JUNB	5382.972762	5122.250743	4110.174735	8376.600281	8178.824411	7000.311519

KRT18	5963.547811	5044.383747	4698.411346	7650.044605	9176.10223	7952.647217
VPS13B	467.4373472	587.9956515	633.125892	989.1987833	913.5981858	1101.974294
HEATR5B	537.9003018	611.9547273	519.0323044	993.7115515	833.3574417	1017.740647
SOX13	1229.628181	1234.890698	1151.223003	790.6369837	733.6296599	811.615959
AMOTL1	2496.968929	2863.109556	2781.264997	3855.709126	3849.263122	4076.908492
CTSB	5706.506892	5193.129676	5059.395976	3569.599624	3924.91868	3526.91233
ZNHIT6	791.963913	758.7040664	801.4606934	1212.129531	1192.148197	1169.361211
BCL9L	1948.151831	2156.31682	2013.471264	3255.510959	2772.890855	3093.852288
ZBTB17	758.2210896	701.8012615	628.4499253	1111.043524	1103.883379	1109.902166
CDC27	1898.530032	2210.224741	2498.836608	3600.286448	3260.066801	3593.308263
C9orf16	1047.01996	823.59323	789.30318	441.3487272	553.6611339	403.3305193
CENPB	2526.742008	2295.079801	2032.17513	1478.382853	1521.135248	1446.836752

CAST	3948.902769	4068.051409	4149.452855	5398.173287	5685.629864	5619.8707
SLAIN2	846.547892	936.4005452	905.2671543	1340.292147	1395.04265	1333.864568
HUS1	307.6551542	391.3315711	360.9846297	582.1470942	694.6555842	644.13965
FRS2	324.5265659	389.3349815	391.84601	685.9407621	620.1463218	630.2658729
TMED4	1964.030807	2030.531673	1932.109443	1376.394292	1383.579687	1354.675233
BICD2	3347.486565	3726.634579	3513.521383	2504.586335	2302.909354	2528.991364
TENM3	613.3254364	777.6716681	663.0520789	391.7082773	343.8889031	405.3124875
OAS2	646.0758238	602.9700739	707.9413593	338.4576129	413.81298	352.7903314
D2HGDH	470.4146551	438.2514278	394.65159	246.3971422	223.527787	214.0525606
ROS1	16.87141168	27.95225508	53.30602045	153.4341178	131.8240795	129.8189141
DMAC1	1064.883808	988.311876	763.1177664	468.4253363	496.3463167	567.8338761
PPP4R2	933.8822583	1101.119191	1088.565049	1563.222895	1630.0334	1601.430268

RFX1	245.1316874	257.5600647	209.4833084	432.3231909	480.2981679	432.0690575
EIF2S2	7216.994455	6627.679338	6848.420838	9220.487929	10670.87266	9647.229989
SP140L	537.9003018	599.9751894	609.7460585	913.384278	985.8148554	909.7233826
AC011462.1	1398.342298	1324.737232	1322.363384	843.8876482	930.7926309	897.8315737
LMTK2	1885.628364	2250.156534	2238.852859	3259.121174	3020.490865	3363.399957
ACD	948.768798	870.5130868	757.5066064	472.0355508	529.5889107	512.3387678
NCOA1	293.7610504	344.4117144	314.2249627	560.485807	523.857429	656.0314589
LYST	402.9290084	444.2411968	528.3842378	794.2471983	753.1166977	866.1200832
BMP1	1174.051766	1117.091908	1043.675769	677.8177794	723.3129928	732.3372328
PLEK2	1738.747839	1536.375735	1317.687418	2278.045373	2568.850106	2528.991364
FKBP1A	4002.494312	3687.701081	3759.477232	2637.26172	2795.816782	2715.296371
RP9	255.0560472	203.6521441	173.9459615	410.6619037	445.9092776	424.1411849

TAX1BP1	2029.531582	2058.483928	2200.509932	2821.382661	3332.283471	3185.022823
FAM168B	2420.551358	2552.639866	2662.495442	3703.177562	3616.564964	3479.345094
MTUS1	668.9018513	775.6750784	752.8306397	1143.535455	1102.737082	1280.351427
NFATC4	41.68231121	26.95396025	32.73176694	127.2600625	124.9463014	105.0443122
SP2	617.2951803	532.0911413	505.0044043	857.4259527	1000.716708	922.6061756
ROCK2	882.2755873	1090.137948	1180.213997	1721.169781	1627.740808	1866.023017
TMEM203	804.8655808	778.6699629	752.8306397	339.3601665	510.1018729	413.2403601
HIST3H2A	294.7534864	274.5310767	290.8451291	524.3836616	763.4333648	493.5100703
MSH3	287.8064346	340.4185351	324.5120894	571.3164506	561.6852083	538.1043538
PPP2CA	2700.418305	2549.644981	2718.607043	3597.578787	3924.91868	3721.145209
ZNHIT1	1700.042836	1554.345041	1574.865587	1034.326465	1130.248195	1064.316899
CRYBG2	393.9970845	395.3247504	331.9936361	194.0490314	194.8703784	138.7377708

ZNF496	573.6279971	578.0127032	572.3383248	902.5536344	935.3778163	870.0840195
RBM38	872.3512275	808.6188076	779.0160532	530.701537	469.9815008	500.4469588
METTL26	1120.460223	921.4261228	836.9980404	543.3372879	577.7333571	543.0592741
LY6D	293.7610504	281.5191404	237.5391087	129.0651697	127.2388941	109.9992325
AK3	1125.422403	1161.016881	1196.112283	740.0939802	762.2870684	785.850373
CHRM3	146.8805252	129.7783272	177.6867348	320.4065402	319.8166798	315.1329365
ADAR	6870.634298	7382.390225	7676.066945	10209.68671	9761.859661	10515.33204
ZNF33B	381.0954168	355.3929574	416.1610368	622.7620077	649.9500268	706.5716468
PHF20L1	818.7596845	905.4534056	949.2212413	1436.865386	1283.851905	1457.737577
TRMT10C	653.0228756	620.9393807	653.7001455	365.5342219	416.1055727	338.9165543
PIP5KL1	137.9486014	146.7493392	163.6588347	357.4112392	356.4981628	265.5837326
ACIN1	3518.185553	3312.342227	3055.276646	4612.951625	4635.622413	4819.155566

ZNF654	217.3434799	303.4816266	265.5949089	518.9683398	480.2981679	496.4830225
NBAS	572.6355612	582.0058825	575.1439049	924.2149216	859.7222576	936.4799527
EBNA1BP2	2552.545344	2286.095148	2208.926672	1483.798175	1683.909329	1389.359676
NIT2	838.6084041	795.6409749	887.4984808	500.9172671	558.2463193	483.6002295
TBC1D15	429.7247799	415.2906469	417.0962302	642.6181877	771.4574392	718.4634558
ZMIZ1	1162.142534	1267.834427	1287.761231	815.0059319	739.3611416	837.381545
PPIL4	480.3390149	505.1371811	553.634458	789.7344301	842.5278125	902.7864941
WDR45	468.4297831	371.3656746	359.114243	691.356084	923.9148529	689.7249175
MLF2	5640.013681	4935.569611	4745.171013	3371.037825	3655.539039	3007.636673
AREG	9973.981611	9063.518709	8731.900227	12787.37989	13706.26538	12622.16419
AC093323.1	551.7944056	519.1133086	549.8936846	330.3346302	310.6463091	270.538653
UBE2L3	2671.637661	2317.042287	2538.114728	1657.991026	1658.690809	1788.726259

GBP2	84.3570584	65.8874584	82.29701403	231.0537304	205.1870455	168.4672931
DGAT2	187.5704004	198.66067	174.8811548	83.937488	71.0703733	62.43199685
GADD45GIP1	1523.389231	1517.408133	1361.641505	975.6604788	981.2296701	808.6430068
ZNF296	265.972843	235.5975785	219.7704352	106.5013289	115.7759307	85.22463061
FRYL	1143.28625	1307.76622	1309.270678	2011.792051	1748.101924	2123.678877
RAB34	2460.248797	2110.395258	1956.42447	1306.897663	1460.381542	1398.278533
TNFRSF12A	3142.052317	2716.360217	2304.316393	4655.371646	4691.790934	3960.963355
INAFM2	467.4373472	431.2633641	469.4670573	258.1303394	247.6000102	255.6738918
KIF1C	2951.504608	2852.128313	2635.374836	1949.51585	1994.555638	1960.166504
PPP1R12A	810.8201967	970.3425692	1100.722563	1605.642916	1476.42969	1756.023784
CA2	666.9169794	736.7415803	642.4778254	415.1746718	419.5444617	376.5739492
IGF2BP1	2280.617885	2420.864949	2513.799701	3495.590226	3339.161249	3339.616339

SRF	1489.646408	1444.532611	1223.23289	2230.210031	2049.577862	2086.021482
TMEM160	434.6869598	406.3059935	321.7065094	213.9052114	166.2129698	141.710723
ACOT9	900.139435	924.4210073	832.3220737	1277.113393	1499.355617	1356.657201
EVC	202.4569402	202.6538493	223.5112086	413.3695646	395.4722385	372.6100129
DRAM1	281.8518187	274.5310767	306.7434159	494.5993917	514.6870582	504.4108951
EIF2AK4	1791.346946	1874.79768	1789.024862	1150.755884	1076.372267	1323.954727
CELSR2	1818.142718	2063.475402	1890.960936	1296.067019	1071.787081	1313.053902
SPATA13	241.1619434	227.6112199	268.4004889	121.8447406	81.38704039	106.0352962
XRN1	562.7112013	736.7415803	890.3040608	1537.951393	1221.951902	1350.711297
MXRA8	98.25116214	77.86699629	72.94508062	23.46639449	10.31666709	8.918856692
SLC41A2	35.72769532	60.89598428	61.72276052	164.2647615	177.6759332	140.7197389
RUSC1	1207.794589	1170.001534	1186.76035	833.9595582	746.2389196	687.7429494

CD109	1882.651056	2357.972375	2538.114728	1483.798175	1184.124123	1350.711297
TICAM1	698.6749308	592.9871256	542.4121379	976.5630324	1050.007451	1004.857854
GPR160	143.9032173	124.786853	143.0845812	283.4018412	295.7444566	275.4935734
MORC3	614.3178724	659.8728788	698.5894259	978.3681397	1064.909303	1058.370994
ZNFX1	2991.202047	3117.674736	3322.741941	2173.349152	2078.235271	2304.037979
ACAT2	955.7158499	862.5267282	853.8315205	513.553018	555.9537266	584.6806054
NOC3L	455.5281154	596.9803049	640.6074387	1008.15241	981.2296701	955.3086501
TAF3	288.7988705	268.5413077	224.4464019	483.768748	455.0796484	509.3658155
LSM12	480.3390149	600.9734842	503.1340176	1067.72095	876.9167028	839.3635131
SNAPC3	820.7445565	874.506266	944.5452746	1315.923199	1321.679684	1373.503931
MTHFD1	2084.115561	2173.287832	2143.463138	1473.870085	1297.607461	1535.034335
PHC2	1943.189651	1891.768692	1613.208514	2673.363865	2882.935304	2638.990597

TCIM	25.80333551	28.9505499	9.351933412	100.1834534	93.99630017	103.062344
HIST1H4I	431.7096518	338.4219454	420.8370036	177.803066	199.4555638	218.0164969
VANGL1	815.7823766	971.340864	926.7766012	581.2445406	483.737057	556.9330512
YIPF5	736.3874981	792.6460905	816.4237869	1201.298887	1315.948202	1137.64972
RANBP10	816.7748125	833.5761783	707.006166	1243.718908	1173.807456	1256.56781
TRMT13	275.8972028	321.4509334	331.0584428	579.4394333	497.4926131	594.5904461
TCP1	6089.587181	6037.687097	6116.164452	4570.531605	4578.307596	4343.483209
ZCCHC3	710.5841625	667.8592374	605.0700918	1024.398375	1077.518563	1018.731631
CSRP2	1115.498043	1058.192514	976.3418482	645.3258486	702.6796586	661.9773634
WASF1	436.6718317	444.2411968	458.2447372	728.360783	727.8981781	701.6167265
PAXX	691.7278789	592.9871256	563.9215848	361.9240074	351.9129775	347.835411
PLSCR1	3840.727247	3986.191233	4206.499649	5775.440707	6169.366921	5297.800875

PAPOLG	357.2769532	411.2974676	437.6704837	708.504603	668.2907683	666.9322838
KAT5	279.8669467	274.5310767	304.8730292	477.4508726	529.5889107	506.3928633
UBTD2	402.9290084	471.195157	463.8558972	737.3863193	789.7981807	703.5986946
PPP2R1A	7390.670752	6400.068118	6242.415553	4608.438857	4796.103901	4436.635712
PPP1R9B	1558.124491	1618.23591	1441.132939	2456.750993	2195.157498	2184.128906
BCLAF3	112.1452659	120.7936737	137.4734212	242.7869277	263.648159	328.0157295
VHL	1400.327169	1503.432005	1472.929512	2101.144861	2229.546388	2015.661612
IL23A	48.62936308	43.92497227	48.63005374	135.3830452	175.3833406	120.9000574
TMEM141	703.6371107	576.0161136	478.8189907	333.9448447	286.5740859	259.6378282
FBXL14	300.7081023	274.5310767	274.9468423	490.0866235	499.7852058	492.5190862
BAZ2B	352.3147733	372.3639694	445.1520304	663.3769213	641.9259524	781.8864367
TMCC1	278.8745107	317.4577541	300.1970625	516.2606789	499.7852058	548.0141945

CCNYL1	560.7263<br>294	601.9717<br>79	567.6623<br>581	358.3137<br>929	317.5240<br>872	338.9165<br>543
ABI1	1121.452<br>659	1232.894<br>108	1199.853<br>057	1648.062<br>936	2031.237<br>121	1815.482<br>829
SETD2	1322.917<br>163	1431.554<br>778	1510.337<br>246	2093.924<br>432	2019.774<br>157	2107.823<br>132
FPGS	2683.546<br>893	2423.859<br>833	2071.453<br>251	1527.120<br>749	1586.474<br>139	1496.385<br>956
CHTOP	1449.948<br>969	1380.641<br>742	1277.474<br>104	2102.047<br>415	2237.570<br>463	1888.815<br>651
CELSR3	402.9290<br>084	411.2974<br>676	411.4850<br>701	741.8990<br>875	629.3166<br>926	672.8781<br>882
PSMB5	2485.059<br>697	2188.262<br>255	2131.305<br>625	1484.700<br>729	1613.985<br>252	1406.206<br>405
PHF13	834.6386<br>602	819.6000<br>507	738.8027<br>396	482.8661<br>944	515.8333<br>546	480.6272<br>773
AGPAT2	1363.607<br>038	1214.924<br>801	1024.971<br>902	698.5765<br>13	773.7500<br>319	603.5093<br>028
PDE10A	97.25872<br>616	94.83800<br>83	119.7047<br>477	217.5154<br>259	247.6000<br>102	244.7730<br>67
SELENOI	1084.732<br>527	1271.827<br>606	1471.994<br>319	2097.534<br>646	1942.972<br>302	2133.588<br>718
TBC1D10<br>A	319.5643<br>86	324.4458<br>179	297.3914<br>825	122.7472<br>943	170.7981<br>552	156.5754<br>842

CIT	1992.81145	2330.02012	2444.595394	1475.675192	1226.537088	1522.151542
CEP135	359.2618252	472.1934519	474.143024	762.6578211	715.2889184	828.4626883
DIPK1A	339.4131056	411.2974676	402.1331367	173.2902978	201.7481565	212.0705925
TACC2	735.3950621	724.7620424	690.1726858	1028.00859	1168.075974	1132.6948
XRCC6	10820.5295	9784.287573	10067.35632	7214.1112	7762.718838	7290.669854
TIMM8B	800.8958368	702.7995563	649.9593722	377.2674192	464.2500191	334.952618
FAM185A	223.2980958	199.6589649	206.6777284	90.25536344	89.41111479	93.15250323
COX7A2L	1006.330085	1004.284593	973.5362682	1604.740362	1784.783407	1368.54901
NUDT4	831.6613523	968.3459795	1022.166322	1467.55221	1553.231545	1398.278533
POFUT1	2883.026525	3123.664505	3166.564653	2173.349152	2125.233421	2210.885476
IFI6	721.5009583	642.9018668	506.8747909	324.0167548	357.6444592	251.7099555
MON2	942.8141822	1208.935032	1337.326478	1939.58776	1829.488964	2062.237864

BCAT2	824.7143004	711.7842097	692.9782658	443.1538345	444.7629813	453.8707072
PDE8A	665.9245434	791.6477956	780.8864399	1139.92524	1141.711158	1277.378475
KIAA1109	945.7914901	1226.904339	1262.511011	1962.151601	1701.103774	2144.489542
PLIN3	3247.250531	2927.99872	2714.86627	2095.729539	1991.116749	1922.509109
VPS37B	1866.772081	1721.060277	1613.208514	2566.862536	3018.198272	2445.748702
RFWD3	2178.396979	2413.876885	2565.235335	1657.991026	1477.575987	1641.069631
XXYLT1	523.0137621	498.1491173	516.2267244	306.8682357	306.0611237	266.5747167
PPP1R14C	577.5977411	546.0672689	548.023298	332.1397375	324.4018652	333.9616339
BDH1	621.2649242	607.961548	591.0421917	378.1699728	312.9389018	369.6370607
DHX40	1354.675114	1550.351862	1428.975425	2067.750376	2106.892679	2125.660845
STK4	1569.041286	1892.766987	1924.627896	2823.187768	2539.046401	2941.24074
THOC2	3414.972211	3981.199759	4239.231416	5866.598624	5412.811334	6098.516009

POLR2L	2828.442546	2203.236677	1873.192262	924.2149216	883.7944809	666.9322838
SLC38A9	361.2466972	355.3929574	380.6236899	583.9522015	614.4148401	629.2748888
BSG	12565.23196	11466.41435	10564.87918	7926.226017	8455.08183	7164.814876
CLN6	465.4524752	422.2787107	499.3932442	250.0073567	276.2574188	223.9624014
PIGW	393.0046486	393.3281608	425.5129703	217.5154259	223.527787	222.9714173
SFI1	1013.277137	958.3630313	913.6838944	643.5207413	504.3703911	599.5453665
RC3H1	372.163493	424.2753003	520.9026911	787.9293228	739.3611416	796.7511978
DMXL1	554.7717135	581.0075877	623.7739586	999.1268733	849.4055905	998.9119495
TMEM132A	3085.483466	2769.269842	2599.837489	1920.634134	1970.483415	1886.833682
COPS7A	1465.827944	1294.788387	1314.881838	878.1846863	936.5241127	873.0569718
NCAPH	1129.392147	1081.153295	1160.574936	747.3144093	660.2666939	765.0397074
POLD2	4587.039105	3503.016538	3582.72569	2495.560799	2451.927879	2520.072508

SLC25A28	494.2331<br>186	447.2360<br>813	476.9486<br>04	240.0792<br>668	252.1851<br>956	291.3493<br>186
KIF11	1417.198<br>581	1602.263<br>193	1858.229<br>169	1053.280<br>091	922.7685<br>565	1028.641<br>472
CMTM7	757.2286<br>537	717.7739<br>786	680.8207<br>524	439.5436<br>2	463.1037<br>228	417.2042<br>964
EIF4B	6955.983<br>792	6978.080<br>822	6937.264<br>205	8991.239<br>306	10261.64<br>487	9510.474<br>186
PARK7	3652.164<br>411	3140.635<br>517	3042.183<br>939	2180.569<br>581	2317.811<br>207	1952.238<br>632
ADNP2	628.2119<br>761	742.7313<br>492	720.0988<br>727	1046.059<br>662	1111.907<br>453	1120.802<br>991
TSTD2	312.6173<br>341	371.3656<br>746	384.3644<br>632	645.3258<br>486	567.4166<br>9	653.0585<br>067
AFDN	1221.688<br>693	1435.547<br>957	1452.355<br>259	2155.298<br>079	1923.485<br>264	2230.705<br>157
RNF2	584.5447<br>929	671.8524<br>167	653.7001<br>455	933.2404<br>58	1058.031<br>525	1050.443<br>122
ZC3H15	1771.498<br>226	1693.108<br>022	1774.996<br>962	2377.326<br>273	2518.413<br>067	2480.433<br>145
SRSF6	3751.408<br>009	3876.378<br>803	3866.089<br>273	5038.956<br>941	5272.963<br>18	5244.287<br>735
PAICS	10622.04<br>231	10483.09<br>395	10894.06<br>723	7719.541<br>235	7966.759<br>587	8236.068<br>663

GMEB1	343.3828495	335.427061	303.0026426	517.1632325	677.461139	578.7347009
TENT2	736.3874981	763.6955406	794.91434	1142.632901	1117.638935	1220.892383
UBE2Q1	1414.221273	1246.870235	1199.853057	1856.552826	2035.822306	1904.671396
SF3A3	3188.696808	2919.014066	3063.693386	1996.448639	2273.105649	2119.714941
PALLD	2693.471253	2937.981668	3020.674492	4063.296462	3911.163124	4218.619215
AC087289.1	1007.322521	941.3920193	824.840527	586.6598624	575.4407644	546.0322264
PJA1	597.4464607	612.9530221	514.3563377	923.312368	996.1315225	877.0209081
MYL6	12640.65709	10671.77167	8920.809282	7045.33367	6682.907683	5356.268936
CELF2	100.2360341	108.8141358	105.6768476	229.2486231	222.3814906	236.8451944
ZMYND8	2477.120209	2621.522208	2660.625056	3468.513617	3605.102	3847.000187
GABRA2	13.89410374	12.97783272	22.44464019	75.81450529	74.50926233	88.19758285
TIMM17A	1217.718949	1211.929917	1218.556924	824.0314682	821.8944783	673.8691723

CERS2	1260.393696	1262.842953	1193.306703	1727.487656	1780.198221	1876.923842
WBP4	333.4584897	318.4560489	363.7902097	555.9730388	709.5574366	563.8699398
HLA-B	1579.958082	1230.897518	1127.84317	2047.894196	2385.442691	2144.489542
EEF1A2	1176.036638	985.3169915	935.1933412	1565.930556	1850.122298	1578.637635
PRPS2	1128.399711	1093.132833	1145.611843	706.6994957	687.7778061	792.7872615
CWC27	557.7490214	589.9922411	576.0790982	858.3285063	917.0370748	901.79551
CORO1A	313.6097701	249.5737061	221.6408219	118.2345261	122.6537088	94.14348731
RAB38	1006.330085	926.4175969	913.6838944	1412.496438	1699.957477	1379.449835
SHANK2	317.579514	327.4407024	314.2249627	606.5160423	486.0296497	604.5002869
ATP5F1D	2587.280603	2210.224741	2185.546838	1626.401649	1525.720433	1331.882599
PCNX1	906.0940508	1147.040753	1203.59383	1859.260487	1623.155622	1813.500861
MFGE8	1027.171241	1006.281183	935.1933412	657.9615995	635.0481743	636.2117774

FBXO8	216.3510439	192.6709011	227.2519819	393.5133846	405.7889056	380.5378855
SMCR8	645.0833878	686.8268391	686.4319125	1021.690714	1035.105598	1007.830806
USP15	1723.861299	1780.957966	1851.682816	2515.416979	2434.733434	2611.243043
FRY	15.8789757	12.97783272	6.546353389	67.69152258	66.48518792	71.35085354
GPR108	599.4313327	550.0604482	532.1250112	862.8412745	863.1611467	925.5791278
NKX2-1	117.1074458	100.8277772	114.0935876	22.56384086	26.3648159	41.62133123
EPB41L1	815.7823766	883.4909195	892.1744475	524.3836616	589.1963206	500.4469588
PYURF	942.8141822	944.3869037	802.3958868	577.634326	536.4666888	426.1231531
PRICKLE1	120.0847537	114.8039048	115.028781	261.740554	265.9407517	223.9624014
SCARA3	171.6914248	154.7356978	132.7974545	61.37364714	50.43703911	52.52215608
PPP1R21	344.3752855	315.4611645	321.7065094	530.701537	544.4907632	601.5273347
TRMT9B	27.78820747	27.95225508	23.37983353	92.96302434	106.6055599	92.16151915

NAT14	936.8595663	818.6017559	697.6542326	488.2815162	486.0296497	386.48379
ADGRD2	79.3948785	90.84482901	103.8064609	25.27150176	19.48703784	22.79263377
PAGR1	194.5174523	171.7067098	181.4275082	84.84004163	69.92407695	52.52215608
FAM210B	1178.02151	1090.137948	1136.25991	787.0267692	727.8981781	732.3372328
RRP1B	2093.047484	2092.425952	2209.861865	1437.76794	1335.43524	1574.673698
EXTL3	2183.359159	2142.340693	2002.248944	1537.951393	1351.483389	1442.872816
KIAA2013	1751.649507	1637.203512	1529.976306	1074.941379	1133.687084	1115.848071
PERP	10906.87143	10926.33685	11527.19312	7813.406813	8668.29295	7585.983109
CNOT3	2140.684411	1970.633983	2062.101317	2764.521782	2937.957528	3012.591594
KIAA0930	1312.000367	1251.86171	1095.111403	769.8782501	734.7759562	824.498752
SEC61B	1472.774996	1196.955494	1196.112283	768.0731429	867.746332	687.7429494
APEH	3057.695258	2798.220392	2588.615169	1972.079691	1876.487114	1961.157488

CAB39	2000.750938	2166.299769	2215.473025	2976.621886	2926.494565	3067.095718
OSBPL6	111.1528299	136.7663909	144.0197745	290.6222703	272.8185298	262.6107804
SPATA2L	260.0182271	251.5702957	241.279882	454.8870317	435.5926105	436.0329938
LINGO2	48.62936308	76.86870147	67.33392057	171.4851905	169.6518588	170.4492612
SPRYD3	1195.885357	1118.090203	983.823395	731.9709975	680.900028	659.0044111
PRELID1	1660.345397	1467.493392	1369.123052	1031.618804	951.4259651	885.9397648
MSH2	1103.588811	1160.018586	1271.862944	771.6833574	703.8259549	799.7241501
PPIH	485.3011948	415.2906469	391.84601	203.0745677	224.6740833	251.7099555
NFKBIB	769.1378854	733.7466958	656.5057255	1135.412472	1180.685234	1053.416074
ST5	317.579514	292.5003835	298.3266759	145.3111351	119.2148197	169.4582772
HAUS6	2087.092868	2335.011594	2521.281248	3306.956516	3317.381618	3493.218871
NAA80	271.9274589	188.6777218	207.6129218	70.39918348	111.1907453	62.43199685

LITAF	3323.668101	3218.502513	3368.566415	4504.645189	4886.661312	4369.248795
THAP6	198.4871962	226.6129251	256.2429755	427.8104227	432.1537215	406.3034715
CDC25C	436.6718317	394.3264556	386.2348499	209.3924432	222.3814906	232.8812581
NPRL3	887.2377672	775.6750784	808.9422402	534.3117516	516.9796509	498.4649907
ANKRD13D	1358.644858	1311.759399	1203.59383	1947.710743	1800.831556	1905.66238
ZNF260	178.6384766	152.7391081	198.2609883	349.2882565	349.6203848	334.952618
RALGAPA1	358.2693892	469.1985674	524.6434644	861.9387209	732.4833635	860.1741788
NIFK	555.7641495	585.000767	525.5786578	836.6672191	875.7704064	893.8676374
ACACA	2614.076375	2829.167532	2958.016538	4144.526289	3723.170524	4128.439664
ZNF140	161.7670649	181.689658	181.4275082	345.678042	308.3537164	362.7001721
WDR5B	124.0544977	110.8107255	115.9639743	240.9818204	270.5259371	235.8542103
ABHD14B	802.8807088	699.8046718	679.8855591	426.0053154	450.494463	449.9067709

YAF2	331.4736177	292.5003835	328.2528628	556.8755924	528.4426144	534.1404175
B4GAT1	230.2451476	193.6691959	161.788448	74.00939802	88.26481845	65.40494908
POLRMT	1368.569218	1242.877056	1168.056483	874.5744717	789.7981807	732.3372328
TMX4	424.7626	468.2002726	501.2636309	240.0792668	281.9889005	250.7189715
PI4KB	1260.393696	1217.919686	1205.464217	1801.497054	1812.294519	1683.681947
COL17A1	11951.90652	12953.87364	12219.2362	9386.557798	8965.183703	9327.142132
USP33	1085.724963	1241.878761	1357.900731	1834.891539	1818.026001	2026.562437
TMEM14A	492.2482467	444.2411968	470.4022506	263.5456612	278.5500115	264.5927485
RGP1	1206.802153	1239.882172	1212.945764	1835.794092	1663.275994	1779.807402
PDCD10	2109.918896	2165.301474	2126.629658	2836.726073	3248.603838	2995.744865
SHLD3	81.37975046	73.87381699	79.491434	181.4132805	177.6759332	200.1787835
POP7	1873.719133	1477.47634	1425.234652	913.384278	1064.909303	906.7504304

ZNF594	20.84115561	24.95737061	28.05580024	106.5013289	83.67963308	92.16151915
C5orf34	385.0651607	393.3281608	482.5597641	239.1767131	210.9185272	214.0525606
PLOD3	4203.958816	3712.658451	3558.410663	2525.345069	2698.381593	2715.296371
ZNF618	875.3285354	995.2999398	958.5731748	599.2956132	410.374091	576.7527328
DHX36	4298.240235	4770.850965	5051.914429	6533.585759	6552.2299	6724.817946
MSRB3	249.1014313	283.5157301	257.1781688	471.1329972	534.1740961	429.0961053
GSDMB	193.5250163	198.66067	196.3906017	365.5342219	377.131497	345.8534428
HS3ST3B1	60.53859485	81.86017559	67.33392057	13.53830452	6.877778061	15.85574523
BANP	161.7670649	207.6453234	197.325795	361.9240074	337.011125	422.1592168
MRPL17	1717.906683	1339.711654	1377.539792	881.7949008	952.5722615	937.4709368
NADK	1395.36499	1412.587176	1405.595592	913.384278	1024.788931	905.7594463
TMEM131L	416.8231121	419.2838262	446.0872238	228.3460695	206.3333418	262.6107804

TRIP12	2625.985606	3063.766816	3134.76808	4334.965106	4070.498316	4371.230763
OTUD1	617.2951803	590.990536	570.4679381	944.9736552	964.0352249	861.1651628
UBL4A	1079.770348	1043.218091	946.4156613	626.3722223	668.2907683	684.7699971
AGFG2	757.2286537	643.9001616	677.079979	407.0516891	412.6666837	439.9969301
RAB32	400.9441364	346.408304	298.3266759	627.2747759	652.2426195	571.7978124
MX1	822.7294284	876.5028557	722.9044528	1191.370797	1495.916728	1258.549778
POLR3C	742.3421139	659.8728788	672.4040123	1009.054963	1111.907453	1072.244771
C4orf46	417.8155481	459.2156192	528.3842378	258.1303394	241.8685285	269.5476689
MDC1	1402.312041	1500.437121	1488.827799	993.7115515	875.7704064	1038.551313
LRIG2	408.8836243	453.2258502	432.0593236	668.7922431	702.6796586	712.5175513
FAM133B	423.770164	385.3418022	512.485951	736.4837657	787.505588	730.3552647
INO80E	826.6991724	751.7160027	717.2932927	481.0610871	492.9074277	447.9248028

ANAPC2	1365.59191	1209.933327	1214.81615	865.5489354	761.1407721	844.3184335
PPP1CC	5496.110464	5524.563557	5796.328329	7247.505684	7902.566992	7604.811806
ATRIP	328.4963098	307.4748059	314.2249627	178.7056196	150.164821	157.5664682
CBWD1	1124.429967	1060.189103	968.8603015	691.356084	699.2407695	644.13965
CPSF4	3029.907051	2572.605762	2563.364948	3976.651313	4190.859432	3765.739492
NCOR2	5411.753406	5860.988913	5338.083592	7691.562072	7254.909558	7703.910214
TMCO6	613.3254364	612.9530221	634.9962787	376.3648655	404.6426093	346.8444269
SPSB2	386.0575967	328.4389972	342.2807629	192.2439241	184.5537113	127.8369459
UBLCP1	1007.322521	1017.262426	1135.324716	1499.141587	1588.766732	1572.69173
LAGE3	542.8624817	461.2122088	368.4661764	207.5873359	262.5018627	167.476309
CLCA2	333.4584897	379.3520332	392.7812033	200.3669068	132.9703758	197.2058313
CDYL	1069.845988	1141.050984	1110.074496	1614.668452	1542.914878	1624.222902

MRPL20	1319.939855	1217.919686	1193.306703	718.432693	888.3796662	780.8954526
GNG12	2372.914431	2755.293715	3077.721286	4163.479916	4001.720535	4113.574903
CEP250	1929.295547	1916.726063	1901.248063	1360.148327	1294.168572	1401.251485
AASS	237.1921995	264.5481284	276.817229	481.0610871	416.1055727	509.3658155
ARC	18.85628364	10.98124307	19.63906017	61.37364714	107.7518563	78.28774208
CCDC91	491.2558107	448.2343761	460.1151239	678.7203331	816.1629966	813.5979271
FBXO4	322.5416939	368.3707902	358.1790497	184.1209414	200.6018601	156.5754842
SPR	624.2422322	548.0638585	496.5876642	277.0839658	343.8889031	310.1780161
RAD51AP1	636.151464	687.8251339	663.9872723	415.1746718	375.9852007	427.1141371
DPH3	379.1105448	319.4543438	348.8271163	588.4649696	631.6092853	555.9420671
DCAF6	730.4328822	738.7381699	702.3301993	1056.890306	1117.638935	1070.262803
SLC2A8	391.0197766	365.3759057	318.9009294	189.5362632	199.4555638	146.6656434

UBA1	6746.5798	6287.260803	6046.960144	4519.086047	4691.790934	4742.849792
ADM	1762.566303	1700.096086	1096.046596	4019.071334	4871.75946	3796.459999
NAPRT	1031.140984	938.3971348	837.9332337	612.8339178	581.1722462	543.0592741
ZNF776	194.5174523	197.6623752	225.3815952	392.610831	366.8148299	376.5739492
TRIM7	883.2680233	894.4721625	853.8315205	578.5368797	589.1963206	499.4559748
PLEKHA8	552.7868415	688.8234287	711.6821327	1076.746486	1031.666709	1038.551313
AGAP3	1723.861299	1516.409838	1413.077139	2266.312176	2328.127874	2264.398616
NR4A3	3.969743925	4.991474121	6.546353389	60.47109351	57.31481718	35.67542677
ZNF37A	236.1997635	280.5208456	285.2339691	482.8661944	443.6166849	498.4649907
DENND5B	484.3087588	595.9820101	678.9503657	975.6604788	957.1574468	1029.632456
BRD3OS	588.5145369	574.0195239	567.6623581	342.9703811	343.8889031	365.6731244
STAT2	2705.380485	2689.406257	2603.578262	1943.197975	1979.653785	1820.437749

SUN2	1727.831043	1700.096086	1593.569453	1231.985711	1071.787081	1074.226739
SLC25A11	1374.523834	1220.91457	1102.592949	838.4723264	759.9944758	710.5355831
HOMEZ	460.4902953	437.253133	492.8468908	278.889073	257.9166773	259.6378282
NFAT5	990.4511093	1543.363798	1590.763873	4368.359591	3328.844582	4723.030111
ZNF703	39.69743925	37.93520332	47.6948604	120.0396334	140.9944503	114.9541529
GOLPH3	3379.244516	3456.096682	3528.484476	4683.350809	4851.126126	4507.986566
DNAJB6	2163.510439	2051.495864	1967.64679	2813.259678	3140.851981	2838.178396
BID	867.3890476	697.8080822	681.7559458	429.61553	452.7870557	447.9248028
SMIM29	326.5114378	242.5856423	253.4373955	131.7728306	135.2629685	101.0803758
COX6B2	228.2602757	213.6350924	229.1223686	92.96302434	114.6296344	100.0893918
RNF44	1739.740275	1652.177934	1460.771999	2492.853138	2316.66491	2303.046995
RHOV	389.0349046	298.4901525	288.0395491	163.3622078	162.7740808	147.6566275

NUDT18	331.4736177	264.5481284	252.5022021	138.0907061	143.2870429	110.9902166
MEA1	967.6250817	793.6443853	761.2473798	508.1376962	516.9796509	379.5469015
AHRR	70.46295467	64.88916358	57.04679381	176.9005123	155.8963027	154.593516
CMPK1	1766.536047	1962.647624	1964.84121	1341.194701	1321.679684	1299.180125
RBM7	357.2769532	337.4236506	378.7533032	609.2237032	660.2666939	548.0141945
ETV6	737.379934	761.6989509	789.30318	1112.848631	1154.320418	1124.766927
FXR1	4013.411108	3993.179297	4111.109928	5273.620886	5667.289122	5371.133697
EXT1	2589.265475	2506.718304	2462.364067	3314.176946	3507.666811	3529.885282
MRNIP	447.5886275	400.3162245	435.800097	723.8480148	691.2166951	656.0314589
PSMB6	2530.711752	2073.45835	1953.61889	1312.312984	1388.164872	1489.449068
C3orf62	81.37975046	89.84653418	80.42662735	178.7056196	207.4796382	195.2238632
SP1	2402.687511	2788.237444	2927.155158	3977.553867	3795.387193	3967.900244

AAMP	2262.754037	1971.632278	1873.192262	1345.707469	1424.846355	1152.514481
VGLL3	34.73525934	62.89257393	53.30602045	136.2855988	154.7500064	147.6566275
TARBP1	730.4328822	799.6341542	748.154673	488.2815162	431.0074252	499.4559748
CRK	1559.116926	1413.585471	1575.80078	2117.390826	2171.085275	2169.264144
CANX	12521.56477	13637.70559	14068.11343	17466.21793	18865.74522	18352.03412
INKA1	148.8653972	128.7800323	120.639941	56.86087897	36.68148299	31.71149046
ZNF587B	451.5583715	499.1474121	521.8378844	306.8682357	259.0629736	269.5476689
IDH2	643.0985158	581.0075877	596.6533517	320.4065402	380.570386	381.5288696
MALSU1	644.0909518	559.0451016	530.2546245	317.6988793	347.3277921	348.8263951
EHBP1	913.0411027	1023.252195	987.5641683	1440.475601	1368.677834	1509.268749
MTCH2	3126.173341	2740.319293	2780.329803	1975.689906	2126.379717	1859.086128
MAST4	715.5463425	856.5369592	953.8972081	1466.649656	1234.561162	1422.06215

CHAF1A	1756.611687	1548.355272	1487.892606	1069.526057	1062.61671	1093.055437
DDN	221.3132238	216.6299769	165.5292214	88.45025617	89.41111479	70.35986946
IFRD2	1936.242599	1744.021058	1687.088788	1250.939337	1193.294494	1238.730096
MME	674.8564672	734.7449907	685.4967191	1029.813697	1060.324118	1046.479185
ANKMY1	264.980407	297.4918576	290.8451291	141.7009206	142.1407466	148.6476115
RPS6KA1	2497.961365	2078.449824	2075.194024	1495.531372	1471.844505	1509.268749
SREBF2	3771.256729	3798.511806	3589.272044	5020.905868	4982.950205	4936.091687
NAAA	297.7307944	298.4901525	303.9378359	173.2902978	142.1407466	136.7558026
AKAP9	2141.676847	2660.455707	2889.747424	3968.52833	3716.292746	4429.698824
RAB3IP	283.8366906	298.4901525	309.5489959	479.2559799	494.0537241	563.8699398
ABCA10	263.987971	309.4713955	283.3635824	469.3278899	566.2703937	480.6272773
APOL1	524.9986341	409.3008779	460.1151239	235.5664986	277.4037151	250.7189715

UGGT2	245.1316874	264.5481284	287.1043558	503.624928	417.251869	505.4018792
RUBCN	486.2936308	512.1252448	531.1898178	777.0986792	789.7981807	807.6520227
CHDH	42.67474719	43.92497227	49.56524708	3.610214538	2.292592687	4.954920385
MARVELD2	577.5977411	575.0178188	649.0241788	933.240458	879.2092955	1013.776711
PXMP4	267.9577149	290.5037939	290.8451291	164.2647615	114.6296344	102.0713599
CTNNAL1	3061.665002	3248.451358	3319.001168	2301.511768	2404.929729	2289.173218
TERF2IP	830.6689163	851.5454851	787.4327933	1163.391635	1382.43339	1219.901399
ST6GALNAC4	886.2453312	845.5557161	796.7847267	508.1376962	586.9037279	487.5641658
LGALS9B	116.1150098	107.815841	118.7695543	38.80980628	13.75555612	37.65739492
EIF3I	4368.703189	3925.295249	3725.810271	2726.61453	2979.224197	2690.521769
PFN2	6483.584265	6211.390397	6324.712567	4704.109543	4760.568715	4881.587563
DNAJB5	123.0620617	144.7527495	150.5661279	55.0557717	53.87592815	39.63936308

ATP5IF1	2473.150465	2019.550429	1731.042875	1209.42187	1384.725983	1098.010357
ABHD13	200.4720682	219.6248613	267.4652956	427.8104227	443.6166849	402.3395352
RCAN1	290.7837425	268.5413077	255.3077822	449.4717099	466.5426118	488.5551499
MERTK	4.962179906	4.991474121	4.675966706	49.64044989	72.21666964	31.71149046
SOX4	583.552357	637.9103927	659.3113056	414.2721182	362.2296446	319.0968728
ICAM5	1032.13342	935.4022503	790.2383733	583.0496478	568.5629864	516.3027041
AP1G1	1620.647957	1886.777218	1843.266076	2505.488889	2508.0964	2693.494721
BRWD3	487.2860668	577.0144084	715.422906	1079.454147	913.5981858	1184.225972
ARF5	1581.942954	1363.67073	1300.853938	898.9434199	938.8167053	952.3356979
HDGFL3	1182.98369	1239.882172	1156.834163	809.5906101	837.9426271	783.8684048
DDB1	6227.535782	6312.218174	6200.331852	4783.534262	4602.379819	4784.471123
ARGLU1	1499.570768	1686.119958	1581.41194	2299.70666	2270.813057	2177.192017

ITGA6	10374.92575	12122.29405	12326.78343	8211.432966	7907.152178	8569.039313
AMFR	1182.98369	1146.042458	1145.611843	800.5650737	803.5537368	765.0397074
NRBP2	429.7247799	532.0911413	472.2726373	256.3252322	285.4277895	269.5476689
KLHL13	608.3632565	659.8728788	668.663239	429.61553	363.3759409	382.5198537
DPY19L1	700.6598027	818.6017559	836.9980404	493.696838	420.6907581	507.3838474
RHBDD3	605.3859485	586.9973567	587.3014183	391.7082773	350.7666811	303.2411275
RBBP8	1007.322521	1102.117486	1183.95477	682.3305476	691.2166951	761.0757711
DHX8	1553.162311	1609.251257	1534.652273	2158.00574	2124.087125	2282.236329
REX1BD	628.2119761	580.0092929	481.6245707	347.4831492	309.5000127	283.421446
FUS	5957.593195	5744.188419	5746.763082	4503.742636	4330.707586	4244.384801
MAP2K7	957.7007219	996.2982346	838.8684271	1392.640258	1432.870429	1352.693265
CLTCL1	262.995535	273.5327818	240.3446887	132.6753843	80.24074405	126.8459618

RAP1B	3032.884359	3131.650864	3264.759954	4134.598199	4674.596489	4280.060228
PCYT2	756.2362177	662.8677633	614.4220252	405.2465818	413.81298	414.2313441
ABRACL	673.8640312	612.9530221	616.2924119	368.2418828	426.4222398	353.7813155
BCHE	49.62179906	69.8806377	63.5931472	9.928089978	11.46296344	9.909840769
STMN3	427.7399079	333.4304713	311.4193826	175.9979587	194.8703784	166.4853249
TMEM184C	357.2769532	413.2940572	446.0872238	676.0126722	652.2426195	674.8601564
SELENON	798.9109649	777.6716681	681.7559458	494.5993917	460.8111301	444.9518505
FARSA	2265.731345	1894.763576	1875.997842	1370.076417	1382.43339	1281.342411
HCLS1	312.6173341	249.5737061	246.8910421	104.6962216	149.0185247	116.9361211
PTK2B	1166.112278	1095.129422	983.823395	1605.642916	1703.396366	1527.106463
C1orf43	3105.332185	2864.107851	2708.319916	3998.3126	4499.213148	3921.323992
SENP6	868.3814836	998.2948243	1082.018696	1451.306244	1458.088949	1620.258966

PTGES2	2911.807169	2553.63816	2355.752027	1868.286023	1721.737108	1606.385189
TBC1D2	2646.826762	2545.651802	2130.370431	1689.580404	1611.692659	1625.213886
MKNK2	5706.506892	5332.890951	4653.522066	7731.274432	8049.292924	7018.149233
IGSF9	426.7474719	352.398073	341.3455695	196.7566923	170.7981552	214.0525606
DYRK1B	97.25872616	105.8192514	86.97298073	226.5409622	199.4555638	210.0886243
FGFR2	209.403992	206.6470286	204.8073417	377.2674192	351.9129775	383.5108378
PTPN4	182.6082205	173.7032994	201.0665684	352.8984711	319.8166798	367.6550925
LTN1	607.3708205	850.5471903	880.9521274	1368.27131	1225.390791	1381.431803
CENPM	494.2331186	458.2173243	408.6794901	222.0281941	270.5259371	262.6107804
MAP3K2	739.364806	918.4312383	928.6469878	1480.18796	1223.098199	1453.773641
SNW1	1125.422403	1102.117486	1130.64875	1529.82841	1819.172297	1607.376173
KRT8	13330.4001	11673.06138	10830.47408	7799.868509	8865.455921	8146.880096

HNRNPM	7851.161047	7066.929061	6942.875365	5382.829876	5435.737261	5177.891802
SLC4A1AP	585.5372289	596.9803049	612.5516385	889.9178835	943.4018907	881.9758285
USP16	874.3360994	912.4414694	913.6838944	1286.138929	1283.851905	1326.927679
COMMD10	131.0015495	120.7936737	154.3069013	268.0584294	287.7203822	265.5837326
MRPL34	524.0061981	468.2002726	415.2258435	277.0839658	263.648159	198.1968154
PRSS23	4483.825763	4534.255092	4667.549966	3306.053963	3547.787183	3287.094183
ACBD3	1126.414839	1226.904339	1153.09339	1825.866002	1687.348218	1594.49338
GPBP1L1	1774.475534	1864.814732	1920.887123	2487.437816	2636.48159	2576.5586
SRP14	3006.088587	2747.307356	2658.754669	1851.137504	2035.822306	2035.481294
PHF7	108.175522	137.7646857	108.4824276	43.32257445	37.82777934	34.68444269
R3HCC1L	300.7081023	367.3724953	360.0494364	579.4394333	561.6852083	580.7166691
CYP2S1	274.9047668	193.6691959	205.7425351	100.1834534	92.85000382	100.0893918

BOD1L1	1007.322521	1299.779861	1321.428191	2068.65293	1699.957477	2192.056778
RHOT2	2826.457674	2508.714893	2427.761914	1783.445982	1882.218596	1752.059848
RNF11	1045.035088	1025.248785	1132.519136	1488.310943	1665.568587	1531.070399
NAA30	397.9668285	438.2514278	442.3464504	714.8224785	667.1444719	658.0134271
KLHL20	335.4433617	348.4048937	392.7812033	576.7317724	584.6111352	611.4371755
NMD3	3015.020511	3267.41896	3279.723048	4244.709743	4616.135375	4277.087276
ZDHHC11	18.85628364	29.94884473	13.09270678	86.6451489	82.53333673	87.20659877
ZCCHC9	550.8019696	577.0144084	581.6902582	824.9340218	941.109298	870.0840195
SEC24B	810.8201967	944.3869037	941.7396946	1304.190002	1315.948202	1449.809705
COX7B	1776.460406	1607.254667	1494.438959	1051.474984	1006.44819	1170.352195
UPF2	778.0698093	833.5761783	880.9521274	1179.6376	1229.975977	1306.117013
RPL39L	471.4070911	453.2258502	386.2348499	241.884374	251.0388992	245.7640511

MTA3	638.1363359	557.0485119	582.6254516	880.8923472	917.0370748	924.5881438
PPL	5300.600576	5305.936991	5246.434644	3932.426185	4115.203873	3900.513327
SLC20A1	1899.522468	1912.732883	1796.506408	2530.760391	2878.350119	2547.820062
MLST8	918.9957186	904.4551108	855.7019072	551.4602706	627.0240999	565.8519079
CDKN2C	86.34193037	74.87211182	106.6120409	26.1740554	24.07222321	19.81968154
PRDX3	2588.273039	2589.576774	2595.161522	1902.583061	1953.288969	1781.78937
C1orf52	500.1877345	482.1764001	484.4301508	778.0012329	778.3352172	718.4634558
TRIM23	219.3283518	207.6453234	249.6966221	422.3951009	468.8352045	364.6821403
KLHDC4	828.6840443	748.7211182	800.5255001	1201.298887	1239.146347	1107.920198
ALKBH8	365.2164411	436.2548382	411.4850701	713.9199248	602.9518767	693.6888538
COG5	607.3708205	635.9138031	698.5894259	1000.931981	939.9630017	1020.713599
WNK4	15.8789757	9.982948243	14.02790012	83.937488	56.16852083	63.42298092

SESTD1	181.6157846	223.6180406	202.936955	83.937488	104.3129673	74.32380577
KCTD11	956.7082859	793.6443853	530.2546245	2580.400841	2923.055676	2032.508342
SCAND1	972.5872616	867.5182023	787.4327933	537.9219661	588.0500242	477.6543251
CTNNBIP1	674.8564672	669.8558271	647.1537921	422.3951009	437.8852032	347.835411
SFMBT2	159.782193	188.6777218	204.8073417	360.1189001	347.3277921	332.9706498
CDKN2D	162.7595009	158.7288771	153.371708	64.08130804	72.21666964	55.49510831
NLGN4X	558.7414574	662.8677633	641.5426321	952.1940843	925.0611492	1002.875886
KIAA2026	480.3390149	657.8762892	678.9503657	995.5166588	969.7667066	1211.973526
FBXO38	662.9472355	664.864353	750.0250597	1006.347302	1062.61671	1069.271819
SUDS3	1780.43015	1765.983544	1911.535189	2646.287256	2418.685285	2555.747934
N6AMT1	90.31167429	86.85164971	98.19530083	179.6081732	212.0648236	225.9443695
CAV1	21849.47056	21966.47931	22222.99937	16576.30005	17618.5748	16377.00286

FAM122B	659.9699275	674.8473012	741.6083196	434.1282981	450.494463	425.132169
FOXD1	529.960814	583.0041774	503.1340176	1009.957517	1178.392641	744.2290418
KIF5B	3126.173341	3700.678914	3973.636507	5204.124256	5195.015029	5265.098401
AGTPBP1	835.6310962	933.4056607	923.0358278	1245.524015	1365.238945	1374.494915
STEAP3	804.8655808	808.6188076	795.8495334	539.7270734	479.1518716	538.1043538
GNB2	11037.87298	9542.700225	9089.144083	7143.712016	7037.113253	6925.987714
SMIM4	291.7761785	233.6009889	152.4365146	84.84004163	91.70370748	82.25167838
EAF1	383.0802887	447.2360813	394.65159	620.9569005	704.9722513	669.905236
CLINT1	1847.915797	2078.449824	2110.731371	2778.96264	2837.08345	2837.187412
CEP95	766.1605775	778.6699629	830.451687	1074.941379	1306.777832	1270.441587
BUB1B	1044.042652	1127.074857	1092.305823	656.1564922	643.0722487	793.7782456
BOD1	1197.870229	1005.282888	983.823395	1564.125448	1733.200071	1544.944176

PPP1R14B	6523.281705	6022.712675	5694.392255	4318.719141	4494.627963	3596.281215
ELOB	2624.99317	2140.344103	1746.941161	1286.138929	1320.533388	1340.801456
CEP68	238.1846355	248.5754112	271.206069	128.1626161	96.28889286	132.7918663
HIF1A	15258.70321	16587.6668	17819.17392	11883.02115	11269.23935	12642.97485
NOC2L	4510.621535	3984.194644	3787.533032	2898.09972	2981.516789	2784.665256
PLEKHA5	972.5872616	1147.040753	1169.92687	1658.89358	1538.329693	1771.87953
MRPS7	1636.526933	1374.651973	1418.688299	991.9064442	1030.520413	915.6692871
RNF111	514.0818383	602.9700739	591.977385	961.2196206	805.8463295	985.0381725
CDA	311.6248981	226.6129251	212.2888885	112.8192043	121.5074124	94.14348731
VPS33B	106.19065	92.84141866	129.9918744	36.10214538	35.53518665	31.71149046
TEX10	972.5872616	999.2931191	1086.694663	1452.208798	1545.207471	1440.890848
TNFSF10	233.2224556	236.5958733	292.7155158	119.1370797	123.8000051	124.8639937

NEPRO	688.750571	808.6188076	868.794614	1220.252514	1350.337093	1152.514481
PEX1	694.7051869	793.6443853	822.0349469	509.9428034	474.5666862	464.7715321
DSP	12329.03219	14491.24767	15367.09698	9784.583951	9439.750389	10285.42373
DNAJA1	3528.109913	3546.941511	3603.299944	2596.646806	2757.989003	2538.901205
ACSL3	3297.864766	3465.081335	3635.096517	4589.485231	5036.826133	4634.832528
PFDN2	1963.038371	1679.131894	1499.114926	2453.140778	2919.616787	2579.531552
VWA1	427.7399079	363.379316	360.0494364	236.4690522	177.6759332	147.6566275
MACF1	3539.026709	4505.304542	4495.474391	6541.708742	5685.629864	7148.959131
HMX3	2.977307944	0.998294824	3.740773365	46.93278899	52.7296318	28.73853823
NDUFV3	625.2346682	528.097962	519.0323044	277.9865194	339.3037177	342.8804906
USP9X	2046.402993	2531.675674	2510.058928	3531.692371	3262.359394	3718.172257
TNPO1	2622.015862	3175.575836	3449.928236	4986.60883	4260.783509	4809.245725

GPATCH11	285.8215626	294.4969732	333.8640228	543.3372879	490.614835	507.3838474
MSL1	1203.824845	1114.097024	1147.48223	1708.53403	1649.520438	1575.664682
PSMC3	4120.594194	3353.272315	3209.583547	2278.045373	2450.781582	2420.9741
KMT2D	1641.489113	2014.558955	2004.11933	3276.269693	2488.609362	3304.931896
SNX33	890.2150751	932.4073659	885.6280941	1440.475601	1257.487089	1288.2793
CCDC115	420.792856	390.3332763	394.65159	234.6639449	229.2592687	229.9083058
C3orf52	257.0409191	241.5873475	283.3635824	415.1746718	552.5148376	455.8526754
NOLC1	4614.827313	4772.847555	4901.348301	3665.270309	3418.255696	3571.506613
SMNDC1	593.4767168	624.93256	678.9503657	909.7740635	1074.079674	964.2275068
SDF4	2859.208062	2526.6842	2430.567494	1821.353234	1883.364892	1771.87953
TRADD	513.0894023	465.2053881	367.5309831	254.5201249	239.5759358	228.9173218
RIOX1	298.7232303	257.5600647	261.8541355	131.7728306	144.4333393	139.7287548

SPTBN1	4272.436899	4611.123793	4582.447372	5818.763281	6008.885433	6449.324373
XRCC2	541.8700457	683.8319546	720.0988727	360.1189001	320.9629762	414.2313441
CRKL	3673.005566	3950.25262	3840.839052	5142.750609	4987.535391	5136.270471
FAM217B	656.0001836	630.9223289	708.8765526	448.5691563	385.1555714	400.3575671
CLDN12	1807.225922	2169.294653	2287.482913	2995.575513	3216.50754	3017.546514
NOP16	852.5025079	764.6938354	767.7937331	507.2351425	543.3444668	469.7264525
AZI2	298.7232303	309.4713955	333.8640228	470.2304435	614.4148401	553.960099
CDR2L	420.792856	347.4065988	332.9288295	222.0281941	150.164821	172.4312294
PTDSS1	2343.141352	2207.229856	2229.500925	1614.668452	1688.494514	1616.295029
MED27	898.154563	829.582999	789.30318	579.4394333	510.1018729	522.2486085
UTP25	740.357242	771.6818992	814.5534002	1145.340562	1095.859304	1181.25302
DDX19B	349.3374654	279.5225508	284.2987757	494.5993917	555.9537266	522.2486085

SLC16A2	113.1377019	96.83459795	100.0656875	30.68682357	21.77963053	39.63936308
ST3GAL5	236.1997635	223.6180406	212.2888885	89.35280981	112.3370417	113.9631688
PCID2	594.4691527	572.0229343	633.125892	941.3634407	845.9667015	975.1283317
NELFB	3101.362441	2739.320998	2356.68722	1831.281324	1823.757483	1813.500861
CKS2	1048.012396	1047.211271	1064.250022	690.4535303	755.4092904	588.6445417
SCFD1	618.2876163	641.903572	685.4967191	944.9736552	1012.179671	960.2635705
DOLPP1	683.7883911	656.8779944	574.2087115	956.7068525	959.4500395	1011.794743
CCDC51	556.7565855	522.1081931	425.5129703	274.3763049	256.7703809	308.1960479
ICA1	167.7216808	175.6998891	129.0566811	305.0631284	398.9111275	283.421446
RAPH1	87.33436635	123.7885582	155.2420946	307.7707893	223.527787	353.7813155
RMND5A	845.555456	812.6119869	856.6371006	1252.744445	1210.488939	1175.307115
BEND3	186.5779645	201.6555545	175.8163482	92.06047071	73.36296599	82.25167838

MT-ATP6	68723.21439	61732.55534	59588.64932	41765.66943	47609.12604	47463.18236
UBE2W	494.2331186	547.0655637	577.9494849	946.7787625	872.3315174	771.9765959
GPD2	1795.31669	2134.354334	2131.305625	1400.763241	1169.22227	1409.179357
DENR	3141.059881	3160.601414	3320.871555	4120.157341	4500.359445	4382.131588
TAP2	1153.21061	1247.86853	1233.520017	813.2008246	798.9685514	872.0659877
PPFIA4	273.9123308	272.534487	218.8352418	461.2049072	422.9833508	461.7985798
SH3GLB1	1222.681129	1313.755989	1269.057364	1728.39021	1828.342668	1777.825434
GLIPR1	1108.550991	1226.904339	1181.14919	1582.176521	1704.542663	1878.90581
PTPN6	802.8807088	649.8899306	691.1078792	444.0563881	440.1777959	443.9608665
EHMT2	1292.151648	1248.866825	1255.029464	934.1430116	819.6018856	832.4266246
POMP	1982.88709	1872.80109	1885.349776	1299.677234	1405.359317	1063.325915
PEX5	1247.492028	1117.091908	1149.352616	841.1799873	770.3111428	739.2741214

ACBD6	961.6704658	894.4721625	863.183454	1279.821054	1362.946352	1304.135045
NAB1	1430.100249	1511.418364	1502.855699	2108.36529	1971.629711	2091.967386
AJAP1	125.0469336	124.786853	119.7047477	41.51746718	45.85185374	47.56723569
H6PD	858.4571238	1055.197629	992.240135	629.0798832	593.7815059	642.1576818
MYCL	163.7519369	151.7408133	148.6957413	48.73789626	58.46111352	74.32380577
ARL14EP	305.6702822	312.46628	367.5309831	536.1168588	581.1722462	532.1584493
KIF17	19.84871962	17.96930684	21.50944685	82.13238073	99.72778189	61.44101277
COQ10B	405.9063163	378.3537384	407.7442968	658.8641531	777.1889209	579.725685
RAB3B	84.3570584	82.85847041	89.77856076	184.1209414	176.5296369	213.0615765
PIP4K2A	550.8019696	533.0894362	585.4310316	823.1289146	836.7963308	871.0750036
UHRF2	1441.017045	1738.031289	1606.66216	2361.080308	2167.646386	2464.577399
UBE2S	8953.757423	7934.447263	7038.265086	5703.236416	5623.729861	5259.152496

TCOF1	4710.101167	4670.023188	4660.068419	3645.414129	3359.794583	3528.894298
RIN3	152.8351411	177.6964787	168.3348014	305.0631284	293.4518639	341.8895065
GAGE13	125.0469336	27.95225508	28.99099358	0.902553634	1.146296344	0.990984077
SMARCA2	1122.445095	1185.974251	1238.195984	805.9803955	721.0204001	843.3274495
TRRAP	3526.125041	4160.892828	4224.268322	6029.960831	5201.892807	6720.85401
ADA	864.4117396	799.6341542	731.3211928	477.4508726	541.0518741	502.428927
SLC30A7	705.6219826	780.6665526	836.0628471	1179.6376	1146.296344	1134.676768
TUBGCP3	842.5781481	807.6205128	861.3130673	1207.616763	1162.344492	1303.144061
SCN9A	9.924359812	20.96419131	35.53734697	83.03493437	126.0925978	87.20659877
THADA	595.4615887	695.8114925	567.6623581	936.8506725	977.790781	954.3176661
HTATSF1	1191.915613	1057.194219	1206.39941	719.3352466	812.7241076	770.9856118
TAGLN2	15297.40821	14113.89223	13316.21799	10139.28753	11034.2486	9285.520801

TSPO	3365.350412	2736.326113	2286.547719	1819.548127	1776.759332	1482.512179
NRAS	2040.448377	2311.052518	2413.734014	3095.758966	3334.576063	3135.473619
POMGNT2	333.4584897	296.4935628	266.5301022	153.4341178	132.9703758	167.476309
RCBTB1	394.9895205	472.1934519	522.7730777	738.2888729	805.8463295	736.3011691
ELMO3	1506.517819	1261.844658	1184.889963	916.0919389	819.6018856	772.96758
KMT2B	1286.197032	1297.783272	1180.213997	1817.74302	1689.64081	1832.329558
FBXO31	735.3950621	665.8626478	673.3392057	1087.577129	998.4241152	1005.848838
NTNG1	25.80333551	30.94713955	40.21331367	101.0860071	128.3851905	90.179551
VAMP8	795.9336569	665.8626478	630.320312	407.0516891	451.6407593	411.2583919
EIF2B2	412.8533682	450.2309657	381.5588832	644.423295	670.583361	660.9863793
GLUD1	2821.495495	2851.130018	2710.190303	2019.915034	2141.28157	2002.778819
VPS35	2968.37602	3082.734417	3142.249627	2269.019837	2333.859355	2202.957603

XKR9	128.0242416	112.8073151	120.639941	37.90725265	41.26666837	49.54920385
FAM86B1	200.4720682	148.7459288	173.0107681	83.937488	65.33889158	62.43199685
RAD1	1086.717399	1005.282888	1029.647869	716.6275857	712.9963257	700.6257424
SLC25A15	463.4676032	501.1440018	519.9674977	313.1861111	292.3055676	286.3943982
EXO1	577.5977411	546.0672689	634.0610854	384.4878483	335.8648287	349.8173791
NPEPL1	311.6248981	256.5617698	242.2150754	140.798367	105.4592636	139.7287548
UBC	17634.59495	14681.92198	13208.67075	20568.29477	23575.8769	28198.45191
TSPAN17	1054.959448	936.4005452	836.0628471	637.2028659	567.41669	577.7437168
PPFIA1	1594.844622	1741.026174	1760.033868	2285.265802	2341.88343	2600.342218
NUDT1	706.6144186	586.9973567	446.0872238	306.8682357	333.572236	272.5206212
TMEM54	826.6991724	747.7228234	667.7280456	426.9078691	489.4685387	470.7174365
AMPD3	265.972843	283.5157301	254.3725888	476.548319	441.3240923	438.014962

KLF2	37.71256 729	18.96760 166	23.37983 353	84.84004 163	102.0203 746	100.0893 918
REC8	157.7973 21	157.7305 822	123.4455 21	261.7405 54	317.5240 872	292.3403 027
CDKN2AIPNL	1178.021 51	1008.277 772	971.6658 815	633.5926 514	699.2407 695	708.5536 15
SF3B1	4389.544 345	4681.004 431	4792.865 874	6079.601 281	6055.883 583	6767.430 261
WDR1	4924.467 339	4578.180 064	4362.676 937	6157.220 894	6104.028 029	6445.360 436
FBXL8	138.9410 374	111.8090 203	121.5751 344	55.05577 17	33.24259 396	35.67542 677
DENND1B	198.4871 962	222.6197 458	260.9189 422	462.1074 608	372.5463 116	417.2042 964
EXOSC4	656.9926 196	530.0945 517	455.4391 572	249.1048 031	318.6703 835	313.1509 683
PRRC2B	4198.996 637	4880.663 396	4835.884 767	6663.553 483	6042.128 027	6792.204 863
MPV17L2	343.3828 495	296.4935 628	251.5670 088	169.6800 833	131.8240 795	125.8549 778
PPM1A	651.0380 037	759.7023 613	784.6272 133	1073.136 271	1130.248 195	1109.902 166
SET	13595.38 051	13243.37 914	13577.13 693	10093.25 729	10827.91 526	9907.858 801

NNMT	80.38731<br>448	58.89939<br>463	95.38972<br>081	188.6337<br>096	189.1388<br>967	179.3681<br>179
MFN2	5807.735<br>362	5479.640<br>29	5605.548<br>887	4346.698<br>303	4203.468<br>692	4285.015<br>149
TPST2	790.9714<br>77	751.7160<br>027	684.5615<br>258	507.2351<br>425	440.1777<br>959	457.8346<br>435
TMEM50B	342.3904<br>135	294.4969<br>732	346.9567<br>296	194.0490<br>314	161.6277<br>844	166.4853<br>249
PRKAG2	238.1846<br>355	230.6061<br>044	253.4373<br>955	406.1491<br>355	395.4722<br>385	440.9879<br>142
CROCC	997.3981<br>611	866.5199<br>075	853.8315<br>205	630.8849<br>905	553.6611<br>339	554.9510<br>831
NCLN	3306.796<br>689	2898.049<br>875	2885.071<br>458	2279.850<br>481	2042.700<br>084	2084.039<br>514
BHLHE41	107.1830<br>86	137.7646<br>857	125.3159<br>077	239.1767<br>131	256.7703<br>809	249.7279<br>874
SNRPB	3426.881<br>443	2961.940<br>744	2728.894<br>17	2155.298<br>079	2119.501<br>939	1919.536<br>157
UQCC3	436.6718<br>317	370.3673<br>798	273.0764<br>556	187.7311<br>56	181.1148<br>223	144.6836<br>752
LYPLA2	2421.543<br>794	2065.471<br>991	1930.239<br>056	1463.039<br>441	1499.355<br>617	1350.711<br>297
POP1	832.6537<br>882	899.4636<br>367	913.6838<br>944	607.4185<br>96	549.0759<br>485	591.6174<br>939

FLNA	26241.99222	27244.46405	24032.59848	35066.0138	33099.30692	35139.30438
FBXW11	1106.566119	1184.975956	1219.492117	1751.856604	1575.011176	1679.71801
NSMF	2328.254812	2255.148008	2209.861865	1709.436584	1654.105624	1566.745826
FLCN	392.0122126	412.2957624	367.5309831	755.437392	709.5574366	548.0141945
PPP2R2D	951.746106	846.554011	836.0628471	1248.231676	1366.385241	1258.549778
SF3B5	1337.803703	1088.141358	1062.379636	629.0798832	782.9204026	752.1569144
ALDH3A2	2514.832776	2636.496631	2796.22809	1957.638833	1877.633411	1951.247647
PACS2	580.575049	492.1593484	535.8657845	816.8110391	826.4796637	819.5438316
BST2	7190.198684	6438.003322	5904.810757	4728.478491	4760.568715	4429.698824
OSBP	2150.608771	2255.148008	2241.658439	2948.642724	3023.929754	2944.213693
REL	173.6762967	295.495268	318.9009294	605.6134887	445.9092776	631.256857
CCDC93	592.4842808	609.9581376	650.8945655	884.5025617	912.4518894	1035.57836

DEDD2	478.3541429	447.2360813	480.6893774	719.3352466	917.0370748	693.6888538
RMI1	562.7112013	692.816608	812.6830135	428.7129763	385.1555714	362.7001721
NEIL2	297.7307944	318.4560489	286.1691624	185.0234951	130.6777832	131.8008822
BLM	751.2740378	828.5847041	894.9800276	542.4347343	474.5666862	550.9871468
IL20RA	143.9032173	160.7254667	129.0566811	61.37364714	56.16852083	54.50412423
EID3	2.977307944	4.991474121	1.870386682	37.90725265	55.02222449	31.71149046
ZNF395	1356.659986	1303.77304	1201.723443	1945.003082	1922.338968	1704.492612
RAB14	1876.69644	1865.813027	2057.425351	2703.148135	2934.518639	2542.865141
CIB1	3572.769532	3051.787278	2844.858144	2155.298079	2236.424166	2147.462495
TMEM106C	1489.646408	1245.871941	1253.159077	857.4259527	892.9648516	916.6602711
BNIP2	1378.493578	1370.658794	1490.698186	1919.73158	2035.822306	1947.283711
DDX21	4758.73053	5805.084403	6062.858431	8113.05462	7703.111428	8050.754641

KNDC1	35.72769532	62.89257393	40.21331367	162.4596542	124.9463014	118.9180892
EFNA5	280.8593827	284.5140249	323.5768961	173.2902978	131.8240795	151.6205638
BCAS3	599.4313327	589.9922411	541.4769446	862.8412745	858.5759613	868.1020514
ZNF704	229.2527117	229.6078096	262.7893289	109.2089898	97.4351892	136.7558026
AL590327.1	163.7519369	122.7902634	121.5751344	48.73789626	51.58333546	52.52215608
MRM1	162.7595009	150.7425185	140.2790012	74.00939802	51.58333546	51.531172
KCNC4	112.1452659	93.83971348	125.3159077	34.29703811	40.12037202	37.65739492
CYTH2	915.0259747	815.6068714	711.6821327	1316.825753	1463.820431	1137.64972
HNRNPUL1	5043.559657	4998.462185	4956.524708	3694.152026	3967.331645	3534.840202
RABL3	187.5704004	191.6726063	221.6408219	348.3857029	395.4722385	342.8804906
ZBED3	296.7383584	318.4560489	311.4193826	534.3117516	464.2500191	529.1854971
C11orf24	1290.166776	1058.192514	1059.574056	1620.083774	1697.664885	1732.240166

PA2G4	7573.278973	6906.203594	7108.404587	4970.362865	5597.365045	5390.953378
SLC43A1	21.83359159	15.97271719	19.63906017	62.27620077	92.85000382	76.30577392
AP1AR	598.4388967	581.0075877	593.8477717	889.9178835	909.0130004	838.3725291
RPS6KB1	842.5781481	975.3340433	1038.999802	1452.208798	1352.629685	1442.872816
TYSND1	651.0380037	576.0161136	489.1061175	304.1605748	366.8148299	319.0968728
PAAF1	462.4751672	411.2974676	437.6704837	240.9818204	283.1351968	240.8091307
CNP	1358.644858	1182.979367	1202.658637	896.235759	834.5037381	772.96758
C2orf49	402.9290084	358.3878419	432.994517	649.8386168	628.1703962	627.2929207
DCXR	855.4798158	709.78762	634.9962787	443.1538345	420.6907581	459.8166117
CCDC43	420.792856	409.3008779	427.3833569	624.567115	677.461139	651.0765385
SPINT2	2810.578699	2667.44377	2451.141747	1939.58776	1938.387117	1783.771338
HMBS	1045.035088	910.4448797	902.4615743	629.0798832	608.6833584	650.0855545

SMIM14	296.7383584	344.4117144	334.7992162	162.4596542	186.846304	182.3410702
CUTC	352.3147733	354.3946626	323.5768961	203.0745677	192.5777857	180.359102
PDCD5	1713.93694	1415.582061	1429.910619	930.5327971	1103.883379	951.3447138
BABAM2	337.4282336	257.5600647	272.1412623	487.3789626	479.1518716	527.2035289
UHMK1	2042.433249	2646.479579	2844.858144	4283.519549	3567.274221	3912.405136
MR1	576.6053051	476.1866312	522.7730777	781.6114474	819.6018856	836.3905609
FAM120AOS	958.6931579	937.39884	938.9341146	1293.359358	1399.627835	1328.909647
ITPR2	261.0106631	297.4918576	282.4283891	485.5738553	422.9833508	538.1043538
CDK7	939.8368742	937.39884	977.2770416	1301.482341	1594.498214	1353.684249
WWC3	149.8578332	141.757865	153.371708	59.56853987	57.31481718	67.38691723
ACER3	897.162127	1045.214681	1043.675769	1459.429227	1417.968577	1450.800689
MRPS21	1210.771897	1049.20786	1018.425549	738.2888729	740.5074379	705.5806628

EXOC4	982.5116214	944.3869037	978.2122349	1315.923199	1404.213021	1389.359676
MTLN	205.4342481	177.6964787	149.6309346	77.61961256	83.67963308	61.44101277
COIL	718.5236504	741.7330544	769.6641198	1084.869469	1095.859304	1047.470169
SLC44A1	2580.333551	2891.061811	3091.749186	1981.105228	1971.629711	2101.877227
DDOST	5070.355428	4568.197116	4568.419472	3501.005548	3561.542739	3513.038553
FAM110C	320.5568219	342.4151247	311.4193826	566.8036824	521.5648363	498.4649907
UQCRH	4216.860484	3666.736889	3416.261275	2454.945886	2832.498265	2225.750237
LRAT	36.7201313	15.97271719	32.73176694	93.86557798	92.85000382	111.9812007
FAM129B	13508.04614	12314.96495	11250.37589	9255.687521	9080.959633	8688.948386
SMOX	1354.675114	1146.042458	1119.426429	1751.856604	1838.659335	1701.51966
AMD1	1743.710019	1939.686844	2144.398331	1377.296846	1312.509313	1324.945711
IFI27L1	208.4115561	210.6402079	180.4923149	91.15791708	82.53333673	103.062344

MT-ND2	37525.98932	34423.20213	35988.11016	45584.37386	61081.54696	50826.58232
SEMA4C	472.3995271	413.2940572	461.9855106	252.7150176	271.6722334	276.4845575
PKN2	1500.563204	1928.7056	2076.129218	2922.468668	2725.892705	2769.800495
SPCS3	1881.65862	2068.466876	2226.695345	1416.106652	1515.403766	1389.359676
FKBP11	818.7596845	725.7603372	689.2374925	484.6713017	495.2000204	433.0600416
APEX1	2947.534864	2679.423308	2552.142628	1791.568964	2083.966753	1849.176288
TMEM238	867.3890476	769.6853095	822.9701403	494.5993917	539.9055778	327.0247454
BCL2L12	848.5327639	772.680194	660.2464989	485.5738553	483.737057	447.9248028
THOC3	2442.38495	2641.488105	1923.692703	1574.053538	1464.966727	1517.196622
IFT88	130.0091135	133.7715065	139.3438078	250.9099104	249.8926029	272.5206212
GSTK1	872.3512275	732.748401	760.3121864	474.7432117	506.6629838	531.1674652
PDGFB	317.579514	335.427061	257.1781688	592.0751842	506.6629838	484.5912136

BCAR1	2249.852369	2123.373091	1915.275963	2904.417596	2966.614937	2861.962014
TIMM13	1211.764333	1112.100434	838.8684271	666.0845822	646.5111377	561.8879716
RBM4	476.369271	418.2855314	447.0224171	677.8177794	699.2407695	694.6798379
CEP192	679.8186471	759.7023613	759.3769931	466.620229	476.8592789	488.5551499
CNN3	693.7127509	696.8097873	664.9224656	443.1538345	441.3240923	459.8166117
TPX2	6544.12286	6217.380165	6533.260682	4622.879715	4631.037228	5144.198343
PIP4P2	178.6384766	174.7015942	204.8073417	325.821862	335.8648287	340.8985225
VAMP4	265.972843	282.5174353	334.7992162	508.1376962	510.1018729	472.6994047
ZNF469	43.66718317	62.89257393	61.72276052	171.4851905	145.5796356	123.8730096
NOMO1	2059.304661	1772.971608	1744.135581	1324.948735	1264.364867	1289.270284
UGCG	2829.434982	3065.763405	3102.036313	4088.567964	4030.377944	3926.278913
JAG2	3072.581798	2934.986783	2610.124615	2153.492972	1999.140823	1945.301743

ZNF317	618.2876163	601.971779	611.6164452	904.3587417	937.670409	862.1561469
USP22	3922.106998	3922.300365	4057.803908	5242.934062	5082.677987	5207.621324
WDR17	192.5325804	255.563475	249.6966221	430.5180836	369.1074226	526.2125448
EWSR1	7776.728349	7376.400456	7122.432487	5519.115474	5827.77061	5545.546894
RNF135	278.8745107	241.5873475	246.8910421	138.9932597	136.4092649	101.0803758
ADI1	713.5614705	654.8814047	655.5705322	404.3440282	472.2740935	338.9165543
MYCBP	483.3163229	476.1866312	497.5228575	292.4273775	284.2814932	316.1239205
DHFR	1868.756953	2265.130956	2288.418106	1470.25987	1427.138948	1514.22367
ZNF544	364.2240051	310.4696903	355.3734697	564.9985751	522.7111326	585.6715895
SPINDOC	885.2528952	878.4994453	821.0997536	617.3466859	549.0759485	538.1043538
IQGAP1	7045.303031	8039.26822	8247.470076	10185.31776	10708.70044	10822.5371
CCT6A	10450.35088	10041.84764	10412.44266	7853.119173	8253.333673	7600.84787

SERTAD1	231.2375836	192.6709011	127.1862944	385.3904019	492.9074277	329.0067135
GALNT10	299.7156663	270.5378974	277.7524223	456.692139	448.2018703	498.4649907
AKAP1	2018.614786	1979.618637	1957.359663	1478.382853	1310.216721	1477.557259
PANX2	260.0182271	245.5805268	185.1682816	92.96302434	113.483338	112.9721848
PPP5C	2967.383584	2753.297125	2711.125496	2070.458037	2063.333418	2101.877227
MAGED1	598.4388967	565.0348705	606.9404785	315.893772	410.374091	352.7903314
TMEM125	77.41000653	53.90792051	59.85237384	141.7009206	179.9685259	150.6295797
POLE3	2406.657254	2114.388438	2244.464019	1698.60594	1593.351917	1521.160558
TRAPPC8	225.2829677	260.5549491	303.9378359	459.3997999	440.1777959	471.7084206
DNAJC1	260.0182271	230.6061044	245.0206554	391.7082773	456.2259447	411.2583919
DDX54	3766.294549	3369.245032	3118.869793	2371.008398	2565.411217	2159.354304
MRI1	343.3828495	329.437292	362.8550164	197.6592459	152.4574137	208.1066562

SNX19	627.2195401	709.78762	735.0619662	1053.280091	1004.155597	1015.758679
CERK	882.2755873	828.5847041	862.2482606	527.9938761	593.7815059	593.5994621
RUNX3	306.6627182	302.4833317	289.9099358	518.9683398	453.933352	496.4830225
CHD7	1286.197032	1559.336515	1587.0231	2222.087048	2007.164897	2333.767501
GOPC	813.7975046	911.4431745	982.8882016	1359.245773	1307.924128	1296.207173
SSSCA1	1203.824845	1036.230028	945.480468	676.0126722	730.1907708	652.0675226
GATD3A	203.4493761	173.7032994	185.1682816	83.937488	89.41111479	88.19758285
CLUH	3702.778646	3225.490577	3145.055207	2512.709318	2284.568613	2376.379816
SREK1IP1	444.6113196	503.1405914	512.485951	772.5859111	757.7018831	714.4995195
MFAP3	338.4206696	424.2753003	440.4760637	725.6531221	583.4648389	730.3552647
MIER3	460.4902953	547.0655637	629.3851186	882.6974545	832.2111454	914.678303
MAPKAP1	1996.781194	1869.806206	1894.701709	1314.118092	1383.579687	1453.773641

SNTA1	337.4282336	277.5259611	288.9747424	157.946886	175.3833406	146.6656434
RRP9	780.0546812	733.7466958	632.1906987	435.9334054	469.9815008	449.9067709
RPF2	553.7792775	640.9052772	591.977385	824.9340218	994.9852262	939.4529049
SLC25A38	482.3238869	491.1610535	440.4760637	748.2169629	707.2648439	703.5986946
TRAPPC1	1060.914064	926.4175969	895.9152209	584.8547551	641.9259524	663.9593315
ATP5PF	1451.933841	1226.904339	1153.09339	810.4931637	888.3796662	830.4446565
GJA1	1880.666184	1939.686844	2052.749384	1485.603282	1274.681534	1387.377708
MTO1	456.5205514	476.1866312	443.2816437	752.7297311	699.2407695	663.9593315
DOCK9	510.1120943	588.9939463	620.0331852	859.23106	842.5278125	955.3086501
7-Mar	1270.318056	1545.360388	1589.82868	2210.353851	2033.529713	2210.885476
CASP6	305.6702822	289.505499	278.6876157	167.874976	161.6277844	139.7287548
OFD1	462.4751672	474.1900415	450.7631905	670.5973504	745.0926233	703.5986946

CANT1	2094.03992	2037.519736	1925.56309	2787.085623	2872.618637	2609.261075
AMER1	259.0257911	267.5430129	275.8820357	149.8239033	112.3370417	147.6566275
GOLIM4	1548.200131	1604.259783	1885.349776	1188.663137	978.9370774	1156.478418
ZNF407	148.8653972	170.7084149	174.8811548	297.8426994	298.0370493	307.2050638
ABCE1	2505.900853	2690.404551	2980.461178	2004.571622	1910.876005	1928.455014
E4F1	913.0411027	857.535254	791.1735667	540.629627	599.5129877	549.9961627
ZNF623	494.2331186	521.1098983	544.2825246	748.2169629	796.6759587	804.6790705
TUT7	572.6355612	676.8438908	682.6911391	981.9783542	933.0852236	993.9570291
DNPH1	1240.544977	978.3289278	730.3859995	578.5368797	557.1000229	474.6813728
CCN2	118.0998818	162.7220564	77.62104732	258.1303394	284.2814932	280.4484938
NEDD8	970.6023896	753.7125923	665.857659	450.3742636	506.6629838	439.9969301
AGPAT5	1053.967012	970.3425692	1080.148309	735.581212	679.7537317	705.5806628

EPHB4	6095.541797	5572.481709	5222.119617	4305.180836	4039.548315	4059.070779
TFR2	394.9895205	414.2923521	397.45717	262.6431076	213.2111199	221.9804332
TWSG1	675.8489032	700.8029666	744.4138996	491.8917308	434.4463142	448.9157868
DIDO1	2709.350229	2957.947564	2934.636705	3788.017604	3789.655712	3870.783804
NSMCE4A	655.0077476	628.9257393	642.4778254	409.75935	426.4222398	423.1502008
OVOL1	167.7216808	138.7629806	140.2790012	281.5967339	287.7203822	269.5476689
DUSP23	408.8836243	346.408304	282.4283891	165.1673151	193.7240821	181.3500861
MMRN2	106.19065	110.8107255	109.4176209	33.39448447	34.38889031	46.57625161
IRF2BP1	1053.967012	932.4073659	927.7117945	693.1611912	639.6333597	593.5994621
ZNF106	1172.066894	1468.491686	1516.883599	2148.07765	1926.924153	2163.31824
TPP2	669.8942873	769.6853095	822.9701403	1128.192043	1071.787081	1200.081717
SP3	1353.682678	1547.356978	1621.625254	2164.323615	2026.651935	2346.650294

CSF3	389.0349046	367.3724953	346.0215363	194.951585	232.6981577	202.1607517
MRPL14	1219.703821	961.3579158	838.8684271	611.9313641	612.1222474	611.4371755
ANKS6	868.3814836	745.7262337	793.9791467	553.2653779	502.0777985	521.2576245
EIF2B3	741.349678	683.8319546	615.3572185	439.54362	447.055574	409.2764238
RNF214	309.6400261	271.5361922	287.1043558	453.9844781	518.1259473	463.780548
CD2AP	1035.110728	1216.921391	1330.780125	1778.933213	1702.25007	1802.600036
CNOT6	1461.8582	1608.252962	1797.441602	2397.182453	2175.67046	2462.595431
RTF1	1171.074458	1206.938443	1220.42731	844.7902018	778.3352172	881.9758285
GCC1	387.0500327	416.2889417	386.2348499	623.6645614	644.2185451	583.6896213
LIMCH1	581.567485	689.8217236	609.7460585	948.5838698	882.6481845	1069.271819
EPDR1	362.2391331	395.3247504	399.3275567	236.4690522	215.5037126	225.9443695
HINT1	3507.268758	3005.865716	2567.105722	2018.109927	2065.626011	1504.313829

ZFAND5	3176.787576	3399.193877	3448.057849	4401.754075	4506.090926	4337.537305
IDH3B	1562.094234	1299.779861	1245.677531	817.7135928	981.2296701	878.0118921
RBM3	3254.197582	3075.746354	3006.646592	2302.414321	2384.296395	2016.652597
BEX4	6.947051869	6.98806377	6.546353389	39.71235991	63.04629889	39.63936308
TMEM138	757.2286537	703.7978511	668.663239	444.0563881	450.494463	489.546134
APLN	97.25872616	79.86358594	84.16740071	28.8817163	29.80370493	17.83771338
TNFRSF13C	43.66718317	32.9437292	44.88928038	4.512768172	4.585185374	3.963936308
CAMSAP1	1325.894471	1479.47293	1380.345372	1913.413705	1867.316744	2050.346055
SEMA4B	12042.2182	11268.75198	9916.79019	8185.25891	7456.657715	8299.491644
B3GNT9	109.1679579	101.8260721	128.1214877	35.19959174	43.55926105	43.60329938
DNPEP	1273.295364	1174.993008	1133.45433	853.8157381	805.8463295	830.4446565
DDX28	428.7323439	361.3827264	366.5957898	206.6847823	200.6018601	244.773067

RAB33B	96.26629018	126.7834427	116.8991677	223.8333013	244.1611212	219.007481
TMF1	474.384399	565.0348705	571.4031315	779.8063401	866.6000357	837.381545
ASPH	7959.336569	8592.323552	8997.495136	6535.390867	6179.683588	6535.539987
C19orf71	50.61423504	58.89939463	39.27812033	146.2136888	124.9463014	118.9180892
CETN2	394.9895205	346.408304	368.4661764	180.5107269	189.1388967	241.8001148
PIGS	1929.295547	1774.968198	1753.487515	1277.113393	1351.483389	1314.044886
NDFIP2	1551.177439	1750.010827	1802.117569	2406.207989	2395.759358	2288.182234
TUT1	300.7081023	259.5566543	253.4373955	130.870277	153.60371	143.6926912
UBA5	740.357242	781.6648474	792.10876	1074.941379	1148.588936	1092.064453
OSBPL11	315.594642	340.4185351	341.3455695	515.3581252	506.6629838	581.7076531
ARL4C	681.8035191	630.9223289	643.4130188	406.1491355	400.0574239	454.8616913
HADHB	1371.546526	1295.786682	1358.835925	1778.03066	2022.06675	1827.374638

HMGN4	1289.174<br>34	1439.541<br>137	1359.771<br>118	926.0200<br>289	1006.448<br>19	942.4258<br>571
PKM	51992.72<br>862	46336.85<br>256	44617.13<br>912	35388.22<br>545	36609.26<br>632	35573.35<br>541
TAF2	876.3209<br>714	1063.183<br>988	1122.232<br>009	1494.628<br>819	1482.161<br>172	1573.682<br>714
JOSD1	1518.427<br>051	1541.367<br>209	1528.105<br>92	2045.186<br>536	2274.251<br>946	2012.688<br>66
BAIAP2L1	3234.348<br>863	3014.850<br>369	2889.747<br>424	3975.748<br>76	4323.829<br>808	4030.332<br>241
MYCBP2	1475.752<br>304	1858.824<br>963	1862.905<br>136	2867.412<br>897	2253.618<br>611	3110.699<br>017
SHROOM2	86.34193<br>037	70.87893<br>252	87.90817<br>408	20.75873<br>359	26.36481<br>59	24.77460<br>192
NMT1	2204.200<br>314	2113.390<br>143	2028.434<br>357	2901.709<br>935	2959.737<br>159	2715.296<br>371
KAZALD1	55.57641<br>495	62.89257<br>393	68.26911<br>391	15.34341<br>178	12.60925<br>978	14.86476<br>115
TSPAN5	273.9123<br>308	291.5020<br>887	283.3635<br>824	163.3622<br>078	154.7500<br>064	146.6656<br>434
SLC1A3	1074.808<br>168	1115.095<br>319	1114.750<br>463	1693.190<br>618	1399.627<br>835	1805.572<br>988
THG1L	428.7323<br>439	368.3707<br>902	378.7533<br>032	590.2700<br>769	683.1926<br>207	599.5453<br>665

ELOVL6	704.6295467	713.7807993	707.006166	486.4764089	449.3481667	477.6543251
MCC	1124.429967	1247.86853	1319.557804	1872.798791	1616.277844	1855.122192
ANAPC5	4866.906052	4815.774232	4742.365433	3541.620461	3579.883481	3852.946091
NARS	2063.274405	2066.470286	2268.779046	2814.162232	2936.811232	2874.844807
DERL1	1207.794589	1201.946968	1200.78825	1667.016563	1871.901929	1579.628619
ZBTB45	480.3390149	454.224145	412.4202635	287.9146094	271.6722334	237.8361785
DTX3	33.74282336	43.92497227	52.37082711	131.7728306	152.4574137	93.15250323
BPHL	173.6762967	198.66067	191.714635	97.47579252	89.41111479	76.30577392
ZNF283	250.0938673	243.5839371	298.3266759	435.0308518	447.055574	444.9518505
FAM174A	391.0197766	351.3997781	301.1322559	212.1001041	175.3833406	178.3771338
ZBTB2	541.8700457	507.1337707	589.171805	760.8527138	994.9852262	849.2733539
DOCK7	960.6780298	1158.021996	1276.538911	1712.144244	1638.057475	1720.348358

ZNF282	887.2377672	846.554011	785.5624066	1216.642299	1274.681534	1144.586609
TMEM30A	2139.691975	2624.517093	2610.124615	1815.035359	1630.0334	1635.123727
ZMIZ2	2252.829677	2138.347514	1904.988836	2908.02781	2783.207522	3105.744097
POLK	297.7307944	362.3810212	403.0683301	663.3769213	515.8333546	653.0585067
TMEM219	861.4344317	779.6682577	750.0250597	514.4555716	565.1240974	484.5912136
PEG10	6929.188021	7997.339837	9043.31961	5622.909142	5074.653913	5854.733926
ISCA2	269.9425869	276.5276663	228.1871753	145.3111351	136.4092649	110.9902166
DAPK3	1383.455758	1140.052689	976.3418482	1758.17448	1922.338968	1742.150007
CD320	964.6477737	813.6102818	757.5066064	550.557717	565.1240974	497.4740066
NECAP1	770.1303214	649.8899306	738.8027396	1061.403074	1079.811156	1027.650488
TYW3	652.0304397	669.8558271	659.3113056	926.0200289	930.7926309	1036.569344
COL1A1	684.780827	640.9052772	621.9035719	1020.788161	872.3315174	1012.785727

WTAP	1402.312041	1403.602523	1342.002445	1762.687248	2071.357493	2001.787835
NMNAT1	230.2451476	182.6879528	198.2609883	104.6962216	102.0203746	78.28774208
ZBTB11	592.4842808	749.719413	796.7847267	1142.632901	1090.127823	1077.199692
TACC1	549.8095336	674.8473012	727.5804195	985.5885688	1017.911153	1002.875886
KCNQ2	50.61423504	67.88404805	78.55624066	10.83064361	18.3407415	15.85574523
ZNF277	412.8533682	387.3383918	401.1979434	582.1470942	710.703733	610.4461914
THRAP3	4560.243334	4645.065817	4757.328527	3583.137929	3631.466816	3558.62382
CEPT1	397.9668285	443.242902	476.0134107	718.432693	695.8018805	639.1847296
BUB1	1378.493578	1532.382555	1595.43984	1008.15241	848.2592942	1113.866102
C6orf136	376.1332369	341.4168299	297.3914825	182.3158342	184.5537113	198.1968154
CEP104	544.8473537	606.9632531	573.2735182	843.8876482	825.3333673	874.0479558
RAPGEF6	172.6838607	232.6026941	223.5112086	370.0469901	409.2277946	351.7993473

PCDHGC3	184.5930925	210.6402079	199.1961817	112.8192043	76.80185502	81.26069431
CALU	1800.27887	2105.403784	2164.037392	1344.804915	1400.774132	1491.431036
MED15	3547.958633	3205.524681	3009.452172	2231.112584	2343.029726	2465.568383
AKIRIN2	901.1318709	863.525023	838.8684271	1240.108694	1321.679684	1163.415306
NCBP2-AS2	623.2497962	599.9751894	616.2924119	418.7848864	404.6426093	357.7452518
CPNE3	1510.487563	1800.923863	2000.378557	1225.667836	1053.44634	1209.991558
WDHD1	826.6991724	929.4124814	885.6280941	574.0241115	511.2481692	638.1937455
RANBP6	1004.345213	1031.238553	1140.935876	739.1914266	750.824105	684.7699971
DCBLD1	883.2680233	903.456816	852.8963272	1330.364057	1168.075974	1265.486666
ABCB10	550.8019696	516.1184241	552.6992647	364.6316683	331.2796433	329.0067135
HAPLN3	239.1770715	265.5464233	257.1781688	145.3111351	124.9463014	131.8008822
ICAM2	11.90923177	10.98124307	3.740773365	43.32257445	63.04629889	51.531172

TRERF1	957.7007219	989.3101708	1031.518255	663.3769213	497.4926131	702.6077105
HIST1H2BD	431.7096518	367.3724953	431.1241303	222.0281941	263.648159	233.8722422
NOTCH1	1658.360525	1823.884644	1649.681054	1222.057621	1135.979676	1286.297332
RHNO1	712.5690345	595.9820101	606.9404785	390.8057237	408.0814983	418.1952805
C1orf216	152.8351411	160.7254667	157.1124813	64.98386168	75.65555867	70.35986946
NPC1	570.6506892	531.0928465	526.5138511	806.8829492	770.3111428	834.4085928
ALKBH5	1345.743191	1301.776451	1312.076258	1889.044757	1821.46489	1717.375405
NEK1	154.8200131	224.6163355	215.0944685	380.8776337	351.9129775	358.7362358
CZIB	341.3979775	316.4594593	299.2618692	182.3158342	187.9926003	170.4492612
NOCT	207.4191201	180.6913632	201.0665684	319.5039866	334.7185323	397.3846148
SCYL3	185.5855285	203.6521441	217.9000485	352.8984711	340.450014	354.7722995
PMP22	92.29654625	102.8243669	82.29701403	22.56384086	16.04814881	39.63936308

PAN3	525.9910<br>7	615.9479<br>066	653.7001<br>455	820.4212<br>537	1015.618<br>56	999.9029<br>336
SKA3	581.5674<br>85	551.0587<br>43	541.4769<br>446	370.9495<br>437	299.1833<br>457	365.6731<br>244
SOX6	20.84115<br>561	27.95225<br>508	51.43563<br>377	101.9885<br>607	127.2388<br>941	102.0713<br>599
PFAS	1132.369<br>455	1072.168<br>641	1049.286<br>929	796.9548<br>592	656.8278<br>048	747.2019<br>94
RNF167	1515.449<br>743	1340.709<br>949	1327.039<br>351	955.8042<br>988	1036.251<br>895	874.0479<br>558
DNAJB12	670.8867<br>233	737.7398<br>751	679.8855<br>591	1008.152<br>41	1060.324<br>118	964.2275<br>068
HIRIP3	569.6582<br>532	511.1269<br>5	447.0224<br>171	321.3090<br>938	315.2314<br>945	252.7009<br>396
ORC6	807.8428<br>887	866.5199<br>075	814.5534<br>002	556.8755<br>924	551.3685<br>412	584.6806<br>054
GCAT	801.8882<br>728	709.7876<br>2	648.0889<br>855	418.7848<br>864	499.7852<br>058	442.9698<br>824
DLGAP1	50.61423<br>504	62.89257<br>393	42.08370<br>036	141.7009<br>206	116.9222<br>27	136.7558<br>026
SEC14L1	1183.976<br>126	1232.894<br>108	1154.963<br>776	1596.617<br>379	1654.105<br>624	1642.060<br>615
FAM173A	286.8139<br>986	272.5344<br>87	237.5391<br>087	148.0187<br>96	146.7259<br>32	103.0623<br>44

NBPF12	313.6097701	350.4014833	389.9756233	625.4696686	511.2481692	594.5904461
SENP7	105.198214	157.7305822	154.3069013	303.2580212	249.8926029	282.4304619
KDM5C	2122.820564	2180.275896	1996.637784	2996.478066	2762.574188	2752.953766
PLEKHA2	701.6522387	863.525023	805.2014668	1119.166507	1195.587086	1176.298099
FREM2	18.85628364	16.97101201	17.76867348	78.52216619	63.04629889	63.42298092
ATP5MPL	1147.255994	990.3084657	952.9620147	659.7667068	730.1907708	677.8331086
CLEC11A	126.0393696	104.8209565	129.9918744	46.03023535	49.29074277	44.59428346
SLCO1B3	180.6233486	187.679427	158.0476747	292.4273775	317.5240872	332.9706498
ZNF322	100.2360341	113.80561	131.8622611	231.956284	221.2351943	230.8992899
MAP2K3	1619.655521	1454.515559	1442.068132	2169.738937	2174.524164	1956.202568
CALD1	1629.579881	1711.077329	1785.284088	2273.532605	2390.027876	2268.362552
TOM1L2	823.7218644	838.5676524	767.7937331	568.6087897	529.5889107	544.0502582

FBXO30	391.0197766	440.2480175	446.0872238	676.9152258	623.5852109	661.9773634
NR2C2AP	369.186185	294.4969732	269.3356823	173.2902978	169.6518588	147.6566275
FMNL2	1436.054865	1695.104612	1660.903374	2463.971422	2056.45564	2425.92902
NLGN2	513.0894023	600.9734842	584.4958383	360.1189001	369.1074226	345.8534428
LTB	2.977307944	8.984653418	7.48154673	48.73789626	42.41296471	42.61231531
TMEM171	83.36462242	84.85506006	82.29701403	25.27150176	20.63333418	30.72050638
TNS4	10351.10728	10460.13317	10003.76317	8089.588225	7393.611416	8166.699778
RABGAP1	1153.21061	1302.774746	1309.270678	1729.292764	1704.542663	1822.419717
CNOT4	227.2678397	315.4611645	279.622809	472.0355508	436.7389069	508.3748315
CHCHD7	973.5796976	851.5454851	865.0538406	577.634326	590.3426169	639.1847296
ZBTB24	335.4433617	369.369085	430.188937	212.1001041	228.1129724	209.0976402
XRCC5	6854.755322	6867.270096	7167.321767	5273.620886	5409.372445	5543.564926

AP2A2	1375.51627	1307.76622	1287.761231	1843.014521	1722.883404	1876.923842
NID2	725.4707023	796.6392698	777.1456666	510.8453571	390.8870531	530.1764811
MKLN1	483.3163229	645.8967513	678.9503657	991.0038906	886.0870735	1057.38001
FBXO5	473.391963	485.1712846	537.7361712	312.2835575	263.648159	329.9976976
SERPINH1	4497.719867	4197.829736	3928.747226	3027.16489	3303.626062	2872.862839
PHF8	489.2709387	502.1422966	481.6245707	731.9709975	687.7778061	809.6339908
BOLA1	135.9637294	126.7834427	107.5472342	40.61491355	44.7055574	56.48609238
AEBP2	1107.558555	1182.979367	1265.316591	1699.508494	1577.303769	1696.56474
RHOBTB1	137.9486014	141.757865	151.5013213	64.98386168	40.12037202	67.38691723
TOMM34	2672.630097	2498.731945	2404.38208	1823.158342	1905.144523	1889.806635
AKAP8L	616.3027443	548.0638585	582.6254516	808.6880564	891.8185553	886.9307488
ACO1	1237.567669	1290.795208	1270.927751	931.4353507	798.9685514	920.6242075

KBTBD7	131.9939855	133.7715065	146.8253546	291.5248239	238.4296395	249.7279874
GOLGA6L9	188.5628364	197.6623752	146.8253546	317.6988793	374.8389043	300.2681753
CDC6	1903.492212	1953.662971	1997.572977	1444.988369	1424.846355	1479.539227
C19orf48	5042.567221	4303.648987	4080.248548	3204.967956	3296.748284	3137.455588
GNL3	2740.115744	2695.396025	2582.068815	1855.650272	2010.603787	2074.129673
TOR2A	389.0349046	337.4236506	391.84601	227.4435159	224.6740833	164.5033568
SPTY2D1	632.18172	679.8387753	731.3211928	957.6094061	986.9611518	1040.533281
EVI5	362.2391331	375.3588539	384.3644632	617.3466859	519.2722436	652.0675226
NXT1	975.5645695	820.5983455	700.4598126	510.8453571	551.3685412	475.6723569
PABPC4	3560.860301	3381.22457	3261.954374	2536.175713	2636.48159	2542.865141
TMIE	3.969743925	7.986358594	2.805580024	27.97916267	56.16852083	43.60329938
ANKH	1631.564753	1664.157472	1625.366027	1237.401033	1131.394491	1217.919431

SLC25A17	492.2482467	458.2173243	440.4760637	289.7197166	299.1833457	273.5116052
ZNF711	97.25872616	131.7749168	147.7605479	249.1048031	237.2833431	268.5566848
IL17RA	696.6900588	695.8114925	663.9872723	466.620229	465.3963155	443.9608665
CYB5R1	822.7294284	717.7739786	625.6443453	464.8151217	465.3963155	433.0600416
PDLIM1	3286.94797	2910.029413	2869.173171	2027.135463	2271.959353	2254.488775
CASTOR3	244.1392514	286.5106146	278.6876157	152.5315642	112.3370417	152.6115478
SAMD9	2535.673932	3224.492282	3521.938123	1965.761816	2025.505639	2183.137921
TMCC3	227.2678397	238.592463	262.7893289	394.4159382	541.0518741	382.5198537
RCE1	786.0092971	732.748401	693.9134592	1136.315026	1126.809306	985.0381725
TMA7	377.1256729	315.4611645	238.474302	159.7519933	151.3111173	75.31478985
FAM241A	493.2406827	524.1047827	581.6902582	818.6161464	831.064849	758.1028188
SPX	1.984871962	0.998294824	0	41.51746718	49.29074277	23.78361785

SATB1	212.3813	214.6333872	211.3536951	353.8010247	338.1574213	393.4206785
ZNF519	100.2360341	126.7834427	116.8991677	49.64044989	30.95000127	43.60329938
KANK1	500.1877345	459.2156192	459.1799305	301.4529139	254.4777883	309.187032
HDAC5	1259.40126	1043.218091	952.9620147	1586.689289	1803.124148	1542.962208
STX7	670.8867233	607.961548	658.3761122	916.0919389	907.8667041	1002.875886
FAAP100	949.761234	798.6358594	747.2194796	527.9938761	554.8074303	535.1314015
TOE1	328.4963098	337.4236506	275.8820357	180.5107269	183.407415	154.593516
TEAD3	1097.634195	1045.214681	999.7216818	720.2378003	701.5333622	767.0216755
PIN1	1071.83086	790.6495008	850.0907472	592.9777378	577.7333571	535.1314015
OTUD7B	608.3632565	648.8916358	657.4409189	958.5119597	912.4518894	895.8496055
DNMT1	4925.459775	4631.08969	4708.698473	3577.722607	3303.626062	3794.47803
ZNF146	1489.646408	1655.172819	1712.339008	2148.07765	2313.226021	2222.777285

AAGAB	1081.75522	1128.073151	1096.046596	1486.505836	1580.742658	1476.566275
DENND4B	1032.13342	998.2948243	1061.444442	1481.090514	1342.313018	1495.394972
INTS10	1114.505607	1140.052689	1130.64875	805.9803955	773.7500319	829.4536724
GPRC5C	322.5416939	306.476511	290.8451291	166.9724224	193.7240821	148.6476115
KLHL17	600.4237686	593.9854204	456.3743505	342.0678274	288.8666786	342.8804906
AKAP17A	477.361707	463.2087985	472.2726373	680.5254403	699.2407695	716.4814876
PVR	2545.598292	2322.033761	2280.936559	3181.501561	3203.89828	3141.419524
C1GALT1C1	392.0122126	426.27189	377.8181099	254.5201249	243.0148248	207.1156721
BARX2	615.3103084	610.9564324	594.782965	417.8823327	379.4240897	397.3846148
CYP1B1	62.52346682	73.87381699	83.23220737	22.56384086	12.60925978	22.79263377
COL18A1	514.0818383	529.0962569	447.9576104	331.2371838	296.890753	286.3943982
ACADS	350.3299014	335.427061	314.2249627	221.1256404	160.4814881	149.6385956

BTBD7	526.983506	651.8865202	699.5246192	979.2706933	896.4037406	1055.398042
SESN3	454.5356794	551.058743	573.2735182	347.4831492	281.9889005	316.1239205
MRTFA	488.2785028	393.3281608	428.3185503	737.3863193	731.3370672	614.4101277
FAM126B	193.5250163	240.5890526	266.5301022	469.3278899	363.3759409	419.1862645
RECQL	851.5100719	974.3357485	1050.222122	614.639025	640.779656	666.9322838
ABCB9	330.4811817	363.379316	337.6047962	196.7566923	213.2111199	195.2238632
SOCS5	240.1695075	300.4867421	331.0584428	500.9172671	496.3463167	467.7444843
SOGA1	1540.260643	1800.923863	1829.238175	1226.570389	970.913003	1236.748128
MICB	262.003099	299.4884473	252.5022021	146.2136888	152.4574137	145.6746593
SMAD2	550.8019696	523.1064879	570.4679381	790.6369837	776.0426246	858.1922106
MMACHC	382.0878528	307.4748059	310.4841893	203.9771214	176.5296369	174.4131975
ASNA1	1441.017045	1193.96061	1052.092509	802.370181	804.7000331	797.7421819

BAZ2A	6018.13179	6638.660581	6764.253437	8839.610295	8114.631816	8711.74102
RFC5	1627.595009	1640.198396	1769.385802	1282.528714	1160.0519	1200.081717
GEMIN6	253.0711752	211.6385027	256.2429755	127.2600625	124.9463014	125.8549778
ERI3	1092.672015	1036.230028	1003.462455	731.0684439	753.1166977	722.4273921
VAV1	435.6793958	418.2855314	381.5588832	596.5879523	739.3611416	621.3470162
WDR91	173.6762967	140.7595702	153.371708	74.00939802	52.7296318	74.32380577
MAPK3	1078.777912	914.438059	878.1465474	624.567115	617.8537292	668.9142519
PLEKHM3	185.5855285	200.6572597	219.7704352	335.749952	330.1333469	387.4747741
SLC38A10	1780.43015	1718.065393	1630.041994	1274.405732	1254.0482	1234.76616
IFT20	144.8956533	163.7203512	175.8163482	270.7660903	314.0851981	293.3312868
CHST14	173.6762967	137.7646857	126.2511011	59.56853987	48.14444643	70.35986946
RAB11FIP2	154.8200131	218.6265665	244.0854621	409.75935	367.9611263	366.6641085

GTF2IRD1	381.0954168	424.2753003	366.5957898	569.5113433	606.3907657	646.1216181
STMP1	867.3890476	871.5113816	951.091628	481.9636408	665.9981756	568.8248601
ZBTB34	216.3510439	239.5907578	275.8820357	461.2049072	410.374091	382.5198537
ACTR3B	171.6914248	172.7050046	145.8901612	82.13238073	58.46111352	76.30577392
SAP30BP	1319.939855	1111.102139	1082.953889	1634.524632	1752.687109	1648.00652
P3H4	330.4811817	281.5191404	225.3815952	152.5315642	137.5555612	138.7377708
CDC5L	1087.709835	1203.943558	1248.483111	1546.976929	1701.103774	1746.113944
WDR5	2497.961365	2313.049108	2269.714239	1829.476217	1673.592662	1716.384421
HNRNPU	10197.27971	10309.39065	10758.4642	13221.50819	13634.04871	12928.37827
CHCHD2	8535.941874	7576.059421	6840.004098	5297.989834	5929.790985	5005.460572
SPON1	35.72769532	32.9437292	29.92618692	98.37834615	104.3129673	82.25167838
FBXL2	233.2224556	240.5890526	243.1502687	385.3904019	377.131497	417.2042964

CIART	0.992435981	4.991474121	0	44.22512809	33.24259396	26.75657008
VDAC3	1767.528483	1616.23932	1671.190501	1177.832493	1209.342642	1287.288316
CALCB	0	0	0.935193341	40.61491355	44.7055574	37.65739492
PARS2	147.8729612	185.6828373	170.2051881	85.74259527	75.65555867	67.38691723
ZNF384	563.7036373	520.1116034	529.3194311	763.5603747	777.1889209	829.4536724
ZNF160	472.3995271	454.224145	506.8747909	678.7203331	725.6055854	741.2560895
FERMT1	1694.08822	1792.937504	1923.692703	1314.118092	1348.0445	1271.432571
ANP32A	3628.345947	3338.297892	3535.03083	2544.298695	2761.427892	2627.098788
SPTSSA	986.4813653	1049.20786	1007.203229	711.2122639	732.4833635	707.5626309
RIDA	440.6415757	432.2616589	432.0593236	272.5711976	263.648159	279.4575097
NAT8L	103.213342	110.8107255	88.84336742	32.49193084	40.12037202	36.66641085
DOP1A	124.0544977	205.6487338	223.5112086	413.3695646	304.9148274	429.0961053

STRN	831.6613523	1115.095319	1105.398529	1547.879483	1452.357467	1647.015536
GLE1	1606.753854	1643.193281	1574.865587	1168.806957	1189.855605	1205.036638
ZNF362	167.7216808	145.7510443	160.8532547	81.2298271	68.77778061	63.42298092
MATR3	56.56885093	53.90792051	69.20430725	127.2600625	144.4333393	149.6385956
LYPD6B	108.175522	90.84482901	101.9360742	195.8541387	232.6981577	184.3230383
PRDX4	1027.171241	902.4585211	883.7577075	675.1101185	631.6092853	553.960099
METTL21A	275.8972028	288.5072042	255.3077822	165.1673151	144.4333393	137.7467867
IL18R1	28.78064346	32.9437292	43.0188937	116.4294188	88.26481845	97.11643954
GALNT9	26.79577149	16.97101201	29.92618692	83.03493437	93.99630017	70.35986946
CENPH	1425.138069	1340.709949	1376.604598	987.393676	1047.714858	864.1381151
NSD1	1187.94587	1623.227384	1571.124813	2360.177754	1981.946378	2394.21753
COA4	1210.771897	1040.223207	953.8972081	643.5207413	714.142622	753.1478985

PHB2	6183.868599	5314.921644	5310.962985	3963.113009	4370.827958	4002.584687
RBM5	1077.785476	912.4414694	1030.583062	1402.568348	1413.383392	1441.881832
FAM91A1	1956.091319	2337.008184	2486.679094	3307.85907	3086.976053	3182.049871
MAGI1	276.8896388	317.4577541	297.3914825	497.3070526	433.3000178	511.3477837
CD3EAP	356.2845173	353.3963678	360.9846297	203.0745677	234.9907504	194.2328791
CITED4	991.4435452	982.3221071	803.3310801	609.2237032	652.2426195	529.1854971
STXBP6	59.54615887	54.90621533	57.04679381	119.1370797	139.8481539	144.6836752
SOWAHC	1685.156296	1896.760166	1750.681935	2463.971422	2467.976028	2297.10109
HMBOX1	154.8200131	195.6657856	201.0665684	365.5342219	309.5000127	316.1239205
SEC61A1	3977.683413	3725.636284	3668.763478	5112.063785	5340.594664	4692.309604
EXOC6B	794.941221	852.5437799	826.7109136	1137.217579	1118.785231	1238.730096
EI24	2863.177806	2697.392615	2863.562011	2167.031276	2091.990827	2136.56167

TMEM14B	951.746106	872.5096764	879.0817408	564.9985751	680.900028	555.9420671
TBC1D3L	21.83359159	39.93179297	24.31502687	81.2298271	115.7759307	83.24266246
ZNF76	725.4707023	658.874584	616.2924119	456.692139	406.9352019	439.0059461
USP13	733.4101901	819.6000507	860.3778739	514.4555716	432.1537215	580.7166691
MAD2L2	1237.567669	938.3971348	812.6830135	586.6598624	613.2685438	615.4011118
CAMKK2	2136.714668	2190.258844	2298.705233	1711.241691	1647.227846	1537.016303
ABTB1	160.774629	140.7595702	140.2790012	250.9099104	310.6463091	258.6468441
TBC1D9B	3107.317057	3188.553669	2894.423391	4155.356933	3850.409418	4153.214266
ILF2	4655.517188	4154.903059	4259.805669	3009.113817	3405.646437	3295.022056
COMT	2678.584713	2195.250319	2034.045517	1453.111351	1640.350068	1597.466332
BLOC1S3	397.9668285	394.3264556	417.0962302	214.807765	281.9889005	204.1427198
CPEB3	31.7579514	49.91474121	40.21331367	100.1834534	104.3129673	127.8369459

NUCKS1	6408.159131	6704.54804	7550.751037	5315.138353	4862.589089	5161.045073
TMEM87A	723.4858303	713.7807993	776.2104732	1074.941379	1134.83338	982.0652202
YJEFN3	42.67474719	33.94202402	16.83348014	109.2089898	95.14259651	93.15250323
MARS2	311.6248981	294.4969732	295.5210958	185.0234951	154.7500064	171.4402453
CTSV	396.9743925	355.3929574	377.8181099	225.6384086	240.7222321	213.0615765
SLC39A3	349.3374654	300.4867421	322.6417027	180.5107269	177.6759332	201.1697676
SMG8	463.4676032	522.1081931	513.4211443	297.8426994	320.9629762	329.9976976
SINHCAF	3125.180905	3506.011423	3651.929997	4786.241923	4861.442793	4342.492225
LRBA	1368.569218	1672.143831	1787.154475	2243.748335	2288.007502	2473.496256
CKMT1B	236.1997635	290.5037939	204.8073417	112.8192043	143.2870429	97.11643954
NFKBIL1	266.9652789	253.5668854	229.1223686	398.9287064	442.4703886	394.4116626
WWP2	1018.239317	983.3204019	917.4246677	1367.368756	1311.363017	1366.567042

ASAP2	355.2920813	474.1900415	453.5687705	691.356084	624.7315072	762.0667551
KRBOX4	130.0091135	120.7936737	131.8622611	256.3252322	237.2833431	221.9804332
LMX1B	44.65961915	46.91985674	38.34292699	98.37834615	116.922227	120.9000574
ATP6V1B2	1701.035272	1626.222269	1674.931274	2181.472134	2333.859355	2166.291192
RNPS1	4431.226656	4109.979791	4249.518543	3366.525056	3217.653836	3256.373677
THOC1	493.2406827	531.0928465	673.3392057	791.5395374	944.5481871	1055.398042
SLIRP	1364.599474	1164.011765	1207.334604	763.5603747	891.8185553	896.8405896
MAPK1	3411.002467	3795.516922	3779.116292	2707.660903	2732.770483	2846.106269
PCTP	368.193749	370.3673798	355.3734697	209.3924432	182.2611186	242.7910988
RNF5	1180.998818	1001.289709	871.600194	639.0079732	710.703733	610.4461914
TBC1D3D	33.74282336	21.96248613	32.73176694	74.91195166	105.4592636	93.15250323
PER2	618.2876163	661.8694685	645.2834054	953.9991916	874.6241101	931.5250323

BRAP	833.6462242	850.5471903	931.4525679	1155.268652	1350.337093	1247.648953
CMTM6	2009.682862	2151.325346	2335.177773	1570.443324	1639.203771	1528.097447
TMEM156	300.7081023	298.4901525	317.965736	441.3487272	530.735207	497.4740066
DHRS11	378.1181088	306.476511	303.0026426	189.5362632	176.5296369	192.2509109
HSD17B10	1133.361891	966.3493899	911.8135077	685.9407621	670.583361	675.8511405
SYNE2	1404.296913	1791.93921	1867.581102	1176.929939	954.8648541	1157.469402
TEX264	482.3238869	418.2855314	342.2807629	265.3507685	215.5037126	208.1066562
POP5	1379.486014	1203.943558	1073.601956	784.3191083	829.9185527	853.2372902
SLC10A6	41.68231121	29.94884473	27.1206069	0.902553634	2.292592687	2.972952231
SQLE	691.7278789	738.7381699	770.5993132	480.1585335	496.3463167	513.3297518
NDUFC1	656.0001836	565.0348705	585.4310316	415.1746718	387.4481641	358.7362358
PHLDA1	15958.37058	16904.12626	17163.60339	21294.85045	22497.21204	20488.59579

HIST2H2BF	11.90923177	14.97442236	17.76867348	50.54300353	63.04629889	69.36888538
RGMA	67.48564672	89.84653418	74.8154673	21.66128723	21.77963053	26.75657008
ZKSCAN1	2306.42122	2516.701252	2746.662843	3488.369797	3334.576063	3424.84097
TMEM67	308.6475902	365.3759057	370.3365631	548.7526097	567.41669	524.2305767
ATP5F1E	5292.661088	4385.509163	3818.394412	2856.582253	3315.089025	2928.357947
RBM41	358.2693892	468.2002726	505.0044043	722.9454612	669.4370646	759.0938029
PRDM15	274.9047668	285.5123197	277.7524223	178.7056196	142.1407466	135.7648185
SNX9	620.2724883	685.8285443	571.4031315	873.6719181	1008.740782	911.7053508
UNC119	677.8337752	662.8677633	610.6812518	411.5644573	367.9611263	469.7264525
CA13	135.9637294	119.7953789	144.0197745	230.1511768	302.6222347	236.8451944
YIPF6	647.0682598	735.7432855	659.3113056	963.0247279	1027.081524	952.3356979
ENTPD7	348.3450294	408.3025831	440.4760637	254.5201249	231.5518614	217.0255128

RPRD1A	668.9018513	719.7705683	815.4885935	1059.597967	1060.324118	1074.226739
NBEAL2	1760.581431	1864.814732	1607.597354	1288.84659	966.3278176	1236.748128
HELZ2	4074.942139	4427.437546	3928.747226	5964.97697	5529.733561	5191.765579
CENPI	382.0878528	399.3179297	476.0134107	271.668644	233.8444541	239.8181466
NELFCD	4117.616886	3859.407791	3897.885846	3035.287873	3033.100125	3103.762129
ZKSCAN8	280.8593827	412.2957624	479.754184	705.7969421	652.2426195	703.5986946
AGO3	494.2331186	571.0246395	590.1069983	818.6161464	772.6037355	906.7504304
CSTF2T	875.3285354	788.6529112	873.4705807	583.9522015	569.7092827	595.5814302
RTTN	213.373736	237.5941682	240.3446887	337.5550593	443.6166849	407.2944556
NEK6	1141.301378	1064.182283	1050.222122	759.0476065	636.1944707	805.6700545
CTHRC1	89.31923831	81.86017559	81.36182069	36.10214538	21.77963053	18.82869746
CXADR	334.4509257	388.3366866	458.2447372	633.5926514	662.5592866	599.5453665

C1orf159	623.2497962	501.1440018	531.1898178	355.606132	343.8889031	345.8534428
TAX1BP3	88.32680233	71.87722735	62.65795386	19.85617996	27.51111224	15.85574523
KIDINS220	954.7234139	1155.027112	1181.14919	1592.104611	1486.746358	1678.727026
CHD4	858.4571238	681.835365	719.1636794	483.768748	526.1500217	422.1592168
TMBIM1	2481.089953	2423.859833	2071.453251	3149.00963	3629.174224	3074.032607
KIFC2	430.7172158	447.2360813	419.9018102	280.6941803	278.5500115	250.7189715
P2RX4	650.0455677	587.9956515	607.8756718	880.8923472	1005.301893	842.3364654
TBRG4	3216.485015	2805.208456	2626.958096	2182.374688	2001.433416	2025.571453
MYB	188.5628364	220.6231562	241.279882	116.4294188	96.28889286	114.9541529
PALB2	522.0213261	637.9103927	683.6263324	370.9495437	372.5463116	409.2764238
ZDHHC21	228.2602757	300.4867421	395.5867833	561.3883606	544.4907632	553.960099
SERPINB13	103.213342	81.86017559	97.26010749	30.68682357	17.19444515	40.63034715

ZNF548	221.3132238	215.631682	221.6408219	361.9240074	386.3018678	342.8804906
HNRNPA0	4714.070911	4492.326709	4470.224171	3569.599624	3380.427917	2747.007861
CARD6	531.9456859	520.1116034	545.2177179	314.9912184	364.5222372	352.7903314
PEX10	398.9592644	373.3622643	345.0863429	214.807765	247.6000102	191.2599268
POLR3A	888.2302032	886.4858039	799.5903067	1254.549552	1233.414866	1141.613657
HMGCL	583.552357	541.0757947	456.3743505	324.0167548	353.0592738	275.4935734
NAGPA	356.2845173	276.5276663	266.5301022	170.5826369	154.7500064	163.5123727
NVL	505.1499144	476.1866312	494.7172775	749.1195166	661.4129902	854.2282743
SLC25A19	250.0938673	232.6026941	267.4652956	132.6753843	82.53333673	146.6656434
ANO8	472.3995271	427.2701848	351.6326963	254.5201249	209.7722309	254.6829078
ANTXR2	402.9290084	383.3452125	430.188937	616.4441323	652.2426195	580.7166691
PRRC2C	4988.975678	6080.613775	6422.907868	8437.071374	7515.118828	9539.212724

LAMA3	2406.657254	2563.621109	2520.346055	1814.132805	1899.413041	1945.301743
HDHD3	359.2618252	320.4526386	285.2339691	191.3413705	176.5296369	182.3410702
COG6	455.5281154	425.2735951	484.4301508	738.2888729	608.6833584	783.8684048
NLRX1	302.6929743	247.5771164	211.3536951	143.5060279	128.3851905	112.9721848
EEF2	45195.53458	41020.93262	37922.08999	52877.90978	59591.36171	54064.1273
SEM1	4097.768166	3317.333701	2900.969744	2253.676425	2503.511214	2007.73374
CBR3	82.37218644	78.86529112	74.8154673	29.78426994	24.07222321	19.81968154
UBXN11	609.3556925	554.0536275	497.5228575	333.9448447	365.6685336	356.7542677
AGAP2	130.0091135	147.747634	144.0197745	245.4945886	267.087048	251.7099555
UTP11	822.7294284	788.6529112	752.8306397	529.7989834	566.2703937	530.1764811
ATF4	9926.344684	9070.506773	8761.826414	11776.51982	13753.26353	11906.67368
ARHGAP17	689.7430069	644.8984565	652.7649522	435.0308518	452.7870557	455.8526754

ATOX1	988.4662373	801.6307439	770.5993132	552.3628243	584.6111352	535.1314015
IVD	1280.242416	1206.938443	1207.334604	936.8506725	860.868554	852.2463061
ZYX	3100.370005	2694.397731	2276.260593	3825.924856	3979.940905	3794.47803
OTUD4	1081.75522	1310.761104	1436.456972	1969.37203	1704.542663	2022.598501
PLCB4	88.32680233	101.8260721	76.68585398	213.9052114	158.1888954	190.2689428
ESYT1	5186.470438	4973.504814	4916.311395	3791.627818	4086.546465	3735.018986
RTCA	830.6689163	891.4772781	834.1924604	1218.447406	1346.898204	1108.911182
NXPH4	1212.756769	1080.155	1004.397648	655.2539386	834.5037381	684.7699971
FBF1	180.6233486	191.6726063	185.1682816	88.45025617	95.14259651	97.11643954
KDM4D	63.5159028	87.84994453	81.36182069	26.1740554	26.3648159	16.84672931
NUMA1	4008.448928	4010.150309	3776.310712	3050.631284	2862.30197	3113.67197
CMIP	3611.474536	3670.730069	3285.334208	4527.20903	4580.600189	4713.12027

AL135905.2	153.8275771	192.6709011	189.8442483	371.8520974	290.0129749	305.2230957
NAV2	527.975942	582.0058825	567.6623581	740.9965338	860.868554	937.4709368
WAPL	1074.808168	1277.817375	1344.808025	1885.434542	1638.057475	1805.572988
TMEM164	895.1772551	937.39884	967.9251082	710.3097103	582.3185425	606.4822551
ARF3	3515.208245	3343.289366	3403.168569	2720.296654	2602.0927	2586.468441
NR4A1	383.0802887	336.4253558	287.1043558	594.7828451	503.2240948	539.0953378
HAX1	1714.929376	1712.075624	1540.263433	2140.857221	2426.709359	2223.768269
COPE	1449.948969	1265.837837	1142.806263	817.7135928	890.6722589	919.6332234
TAF6L	357.2769532	380.350328	330.1232495	200.3669068	230.405565	205.1337039
PTP4A2	2155.570951	2135.352629	2141.592751	2748.275817	3097.29272	2741.061957
CSRNP2	349.3374654	290.5037939	310.4841893	500.0147135	512.3944656	482.6092455
CLPTM1	2583.310859	2505.720009	2375.391087	1740.123407	1851.268595	1962.148472

CAMK1D	130.0091135	113.80561	100.0656875	223.8333013	205.1870455	231.890274
YWHAH	3092.430517	2895.05499	2980.461178	2134.539345	2412.953803	2177.192017
ARID1B	887.2377672	892.4755729	1010.944002	1281.626161	1272.388941	1356.657201
HADHA	2506.893289	2283.100263	2375.391087	3007.30871	3332.283471	3176.103967
HELQ	181.6157846	174.7015942	143.0845812	308.673343	269.3796407	313.1509683
CEACAM19	88.32680233	59.89768946	89.77856076	166.0698687	169.6518588	178.3771338
SLC39A6	1009.307393	1025.248785	1098.852176	756.3399456	735.9222525	743.2380577
DLG4	169.7065528	151.7408133	137.4734212	277.9865194	284.2814932	258.6468441
LCN2	337.4282336	297.4918576	250.6318154	617.3466859	1144.003751	793.7782456
FHL1	1245.507156	1239.882172	1238.195984	1620.083774	1750.394517	1641.069631
IL22RA1	84.3570584	86.85164971	94.45452746	32.49193084	33.24259396	27.74755415
UBAC1	2292.527117	2039.516326	2040.591871	1471.162424	1616.277844	1585.574523

NATD1	125.0469336	97.83289278	86.97298073	217.5154259	240.7222321	180.359102
MAP3K1	320.5568219	389.3349815	417.0962302	646.2284022	531.8815034	639.1847296
B3GALT6	666.9169794	593.9854204	615.3572185	396.2210455	453.933352	342.8804906
ERCC6	257.0409191	343.4134195	316.0953493	503.624928	495.2000204	484.5912136
PLK3	537.9003018	451.2292606	404.9387168	723.8480148	765.7259575	677.8331086
MAP3K14	252.0787392	215.631682	184.2330882	376.3648655	369.1074226	366.6641085
KATNAL1	307.6551542	359.3861367	349.7623096	215.7103186	146.725932	198.1968154
NT5C2	273.9123308	346.408304	324.5120894	482.8661944	504.3703911	516.3027041
NDUFA6	1203.824845	1031.238553	871.600194	728.360783	610.9759511	649.0945704
PRMT7	773.1076294	689.8217236	592.9125783	471.1329972	411.5203873	426.1231531
ABTB2	514.0818383	456.2207347	485.3653441	713.0173712	721.0204001	700.6257424
ITGA3	23801.59214	23575.73057	21057.74846	29140.74919	29838.09382	29037.81542

CHML	695.6976228	945.3851986	998.7864884	1444.085815	1275.82783	1368.54901
COMMD2	2140.684411	2325.028646	2259.427112	2831.310751	3285.285321	2954.123533
SLC16A5	465.4524752	382.3469177	446.0872238	268.0584294	283.1351968	216.0345288
FBRS	1441.017045	1479.47293	1361.641505	2032.550785	2054.163048	1787.735275
SYCE1L	69.47051869	63.89086875	72.94508062	15.34341178	21.77963053	22.79263377
EGR4	18.85628364	4.991474121	3.740773365	47.83534262	72.21666964	45.58526754
SLC35C1	647.0682598	678.8404805	550.828878	411.5644573	427.5685361	350.8083632
CENPV	126.0393696	160.7254667	128.1214877	54.15321806	42.41296471	72.34183761
MZT2A	1111.528299	970.3425692	776.2104732	586.6598624	630.4629889	604.5002869
ATF7	618.2876163	625.9308548	639.6722454	883.6000081	926.2074456	861.1651628
RHPN1	264.980407	234.5992837	234.7335286	421.4925473	362.2296446	419.1862645
ASCC3	1344.750755	1642.194986	1702.051881	2302.414321	2056.45564	2378.361785

TMEM256	156.804885	140.7595702	124.3807144	73.10684439	55.02222449	52.52215608
CDC14B	468.4297831	560.0433964	558.3104247	806.8829492	762.2870684	781.8864367
WDR60	344.3752855	437.253133	420.8370036	613.7364714	594.9278023	662.9683475
ASCC1	443.6188836	426.27189	433.9297103	633.5926514	663.7055829	625.3109525
PDCD6	442.6264476	573.0212291	518.097111	315.893772	262.5018627	341.8895065
LONRF1	242.1543794	275.5293715	259.9837489	407.9542428	400.0574239	439.0059461
PAFAH1B3	620.2724883	509.1303604	482.5597641	342.9703811	331.2796433	334.952618
FUCA2	638.1363359	737.7398751	648.0889855	446.764049	435.5926105	468.7354684
IL13RA1	1243.522284	1309.762809	1309.270678	932.3379043	956.0111505	945.3988094
IRX3	258.0333551	227.6112199	237.5391087	139.8958133	136.4092649	114.9541529
NDUFAF6	296.7383584	315.4611645	328.2528628	175.9979587	187.9926003	191.2599268
DIS3L	498.2028626	524.1047827	491.9116975	342.9703811	290.0129749	335.9436021

DHRS2	452.5508074	372.3639694	388.1052366	241.884374	264.7944554	225.9443695
COPS2	1028.163677	1130.069741	1161.51013	1454.013905	1604.814881	1524.13351
RRP15	665.9245434	619.9410859	652.7649522	965.7323888	931.9389273	866.1200832
TP63	8608.389701	9572.64907	9584.796554	12081.58295	11497.35233	12310.0042
BAG3	2298.481732	2334.013299	2185.546838	1519.90032	1823.757483	1636.114711
LRR1	207.4191201	241.5873475	225.3815952	130.870277	116.922227	109.9992325
GUK1	2918.754221	2541.658623	2180.870872	1632.719525	1823.757483	1813.500861
ZNF621	306.6627182	454.224145	560.1808114	773.4884647	725.6055854	860.1741788
KLF15	6.947051869	13.97612754	10.28712675	44.22512809	48.14444643	57.47707646
BCL6	227.2678397	193.6691959	220.7056285	392.610831	315.2314945	383.5108378
FKBP7	69.47051869	60.89598428	84.16740071	21.66128723	20.63333418	21.80164969
MCMBP	2075.183637	2167.298063	2069.582864	1630.011864	1576.157472	1584.583539

KPNA5	128.0242416	151.7408133	187.9738616	291.5248239	299.1833457	270.538653
TMEM200B	130.0091135	124.786853	123.445521	49.64044989	45.85185374	64.413965
LAT2	23.81846355	24.95737061	27.1206069	74.91195166	97.4351892	66.39593315
KYNU	2289.549809	2262.136072	2396.900534	3013.626585	3119.072351	2921.421059
WDR66	1520.411923	1563.329695	1518.753986	1972.982245	2205.474165	1995.841931
FOXJ3	1515.449743	1690.113137	1632.847574	2166.128723	2087.405642	2205.930555
NKD2	1116.490479	1102.117486	972.6010749	787.0267692	678.6074354	745.2200258
BTBD9	184.5930925	149.7442236	207.6129218	324.0167548	322.1092725	307.2050638
RASSF5	224.2905318	233.6009889	179.5571215	100.1834534	102.0203746	120.9000574
MSANTD4	475.376835	519.1133086	517.1619177	686.8433158	778.3352172	758.1028188
SRRT	3131.135521	3022.836728	2702.708756	3761.843548	3994.842757	4021.413384
PQLC2	232.2300196	181.689658	212.2888885	111.014097	106.6055599	106.0352962

SVIL	725.4707023	755.709182	771.5345065	1037.93668	1005.301893	1107.920198
GTF3C6	235.2073275	208.6436183	232.863142	81.2298271	137.5555612	115.945137
NIPSNAP1	2101.979408	1996.589649	1805.858342	1415.204099	1486.746358	1439.899864
NAGLU	509.1196584	386.340097	348.8271163	259.0328931	210.9185272	228.9173218
RNPEP	1879.673748	1577.305822	1561.77288	1238.303586	1173.807456	1152.514481
ANAPC7	2154.578515	2025.540198	2136.916785	1620.083774	1556.670434	1609.358141
MAU2	890.2150751	929.4124814	893.1096409	1236.498479	1179.538937	1358.639169
FCHO1	436.6718317	415.2906469	317.0305427	253.6175713	183.407415	210.0886243
MYO9A	212.3813	227.6112199	259.0485555	447.6666027	324.4018652	448.9157868
ZNF426	60.53859485	57.90109981	58.9171805	111.9166507	145.5796356	149.6385956
GATD1	1974.947603	1807.911927	1694.570334	1396.250472	1185.270419	1327.918663
ZYG11A	50.61423504	41.92838262	38.34292699	9.025536344	8.024074405	6.936888538

DNAH1	58.55372289	39.93179297	43.95408704	121.8447406	98.58148554	137.7467867
SEZ6L2	411.8609322	401.3145194	366.5957898	248.2022495	244.1611212	242.7910988
INTS1	3261.144634	3351.275725	3131.027306	2527.150176	2260.496389	2562.684823
TRUB2	1090.687143	1072.168641	943.6100813	730.1658902	754.262994	696.6618061
FAM98A	963.6553378	999.2931191	991.3049417	718.432693	726.7518818	665.9412997
LRP1	2404.672382	2750.302241	2396.900534	1775.322999	1493.624136	1966.112409
DCAF16	1013.277137	1170.001534	1345.743218	787.0267692	727.8981781	841.3454813
KHDC4	1334.826395	1428.559894	1527.170726	1892.654971	1985.385267	1909.626316
WRAP53	575.6128691	460.213914	468.531864	322.2116475	323.2555689	293.3312868
PPP1R3E	126.0393696	152.7391081	200.131375	64.08130804	55.02222449	83.24266246
ZNF74	339.4131056	377.3554436	337.6047962	226.5409622	213.2111199	194.2328791
USP6NL	366.2088771	400.3162245	443.2816437	625.4696686	584.6111352	622.3380003

SLC35E1	829.6764803	817.6034611	793.0439534	1075.843932	1144.003751	1143.595625
PGAM5	2545.598292	2333.015004	2341.724126	1916.121366	1622.009326	1762.960673
ZNF791	281.8518187	268.5413077	319.8361227	490.0866235	431.0074252	459.8166117
COPA	4367.710753	4368.538151	4596.475272	5546.192083	5568.707637	5961.760207
ATG5	656.0001836	644.8984565	629.3851186	861.0361672	964.0352249	910.7143667
CENPC	343.3828495	413.2940572	436.7352904	262.6431076	230.405565	214.0525606
GINS4	406.8987523	428.2684796	531.1898178	264.4482149	259.0629736	301.2591594
ADRA1B	44.65961915	25.95566543	30.86138026	89.35280981	113.483338	86.21561469
ODF3B	185.5855285	171.7067098	190.7794416	98.37834615	83.67963308	95.13447138
JADE2	3382.221824	3531.967088	3344.251388	4517.28094	4628.744635	4170.060996
IP6K1	855.4798158	848.5506006	810.8126268	1148.950777	1216.22042	1107.920198
TGS1	1002.360341	1073.166936	1112.880076	1451.306244	1440.894504	1429.990023

SNX21	211.388864	200.6572597	194.520215	106.5013289	119.2148197	81.26069431
SEL1L	860.4419957	1208.935032	1278.409297	1640.842507	1791.661185	1724.312294
SLK	2333.216992	2720.353396	2835.506211	3413.457845	3609.687186	3707.271432
YBX3	7482.967298	7314.506177	7301.989608	9190.703659	9395.044831	9037.774781
UBN2	300.7081023	422.2787107	454.5039638	675.1101185	593.7815059	680.8060608
SHROOM3	1050.989704	1167.00665	1091.370629	1567.735663	1396.188946	1622.240934
CSNK1E	5115.015047	4721.934519	4125.137828	6309.752458	6728.759536	5988.516777
TRIM11	1063.891372	954.369852	953.8972081	1426.937296	1549.792656	1260.531746
PTPRA	989.4586733	987.3135812	956.7027881	650.7411704	708.4111403	730.3552647
SLCO1B7	17.86384766	21.96248613	40.21331367	83.03493437	91.70370748	81.26069431
DYNC1I1	205.4342481	180.6913632	175.8163482	332.1397375	303.768531	311.1690002
DMPK	402.9290084	357.3895471	327.3176694	549.6551634	541.0518741	578.7347009

TIAL1	1457.888456	1610.249552	1692.699948	2047.894196	2229.546388	2171.246113
XIAP	2032.50889	2373.945092	2529.697988	3371.940378	3240.579763	2992.771912
PI3	76.41757055	61.8942791	75.75066064	27.07660903	21.77963053	12.882793
P2RY2	383.0802887	340.4185351	359.114243	218.4179795	169.6518588	239.8181466
USP10	2125.797872	2173.287832	2163.102198	2807.844357	2676.601962	3114.662954
RBBP9	385.0651607	451.2292606	503.1340176	263.5456612	267.087048	290.3583345
RNF43	107.183086	98.8311876	99.13049417	185.9260487	228.1129724	181.3500861
CDKN3	427.7399079	429.2667744	422.7073902	237.3716058	259.0629736	300.2681753
RABGAP1L	459.4978593	460.213914	440.4760637	650.7411704	679.7537317	648.1035863
RFC3	1511.479999	1642.194986	1710.468621	1238.303586	1135.979676	1193.144829
ELL	544.8473537	516.1184241	493.7820842	787.9293228	787.505588	695.670822
BMP7	366.2088771	361.3827264	366.5957898	228.3460695	193.7240821	241.8001148

KIF5A	0.992435981	5.989768946	7.48154673	40.61491355	43.55926105	28.73853823
CCT7	7997.049137	7014.019435	6843.744871	5454.131613	5525.148376	5669.419904
OSER1	951.746106	943.3886089	808.9422402	1180.540154	1514.25747	1293.23422
ERMP1	1643.473985	1910.736294	1986.350657	1388.12749	1197.879679	1343.774408
EPG5	202.4569402	238.592463	249.6966221	344.7754883	387.4481641	423.1502008
ARHGAP39	444.6113196	460.213914	389.9756233	671.499904	616.7074328	644.13965
MRPL37	2484.067261	2212.221331	2119.148111	1640.842507	1727.46859	1676.745058
DNAJC2	1740.732711	1596.273424	1832.043755	2241.943228	2300.616761	2360.524071
KAT14	171.6914248	153.7374029	176.7515415	81.2298271	80.24074405	87.20659877
CCDC124	1721.876427	1522.399607	1361.641505	1030.71625	1156.613011	839.3635131
PPP2R5E	745.3194219	831.5795886	886.5632875	1157.073759	1144.003751	1153.505466
SEC63	1300.091135	1411.588881	1458.901612	1755.466819	1989.970452	1942.328791

CNPY2	215.3586079	136.7663909	177.6867348	81.2298271	75.65555867	88.19758285
VPS37A	331.4736177	398.3196349	390.9108166	582.1470942	521.5648363	657.022443
FGD6	598.4388967	785.6580267	792.10876	1149.85333	982.3759664	1194.135813
C9orf78	1245.507156	1116.093614	1073.601956	810.4931637	858.5759613	752.1569144
TANC2	1379.486014	1484.464404	1565.513653	2084.898895	1838.659335	2248.542871
ITPR1	202.4569402	254.5651802	216.9648552	377.2674192	338.1574213	416.2133123
P3H1	326.5114378	294.4969732	250.6318154	172.3877442	152.4574137	166.4853249
SERPINB6	1898.530032	1738.031289	1776.867348	1354.733005	1365.238945	1351.702281
RABEP2	383.0802887	353.3963678	337.6047962	225.6384086	231.5518614	188.2869746
MTMR4	1301.083571	1388.628101	1328.909738	957.6094061	950.2796688	1038.551313
FAM110A	511.1045303	396.3230452	405.8739101	658.8641531	675.1685463	675.8511405
CCL22	46.64449112	63.89086875	44.88928038	13.53830452	8.024074405	12.882793

PLEKHO2	194.5174523	191.6726063	152.4365146	92.06047071	81.38704039	92.16151915
MYH9	34655.86446	34872.4348	31761.03625	41855.9248	42979.2351	42582.58578
MAPRE2	248.1089953	214.6333872	288.0395491	150.7264569	99.72778189	132.7918663
TBC1D23	640.1212079	662.8677633	684.5615258	983.7834615	970.913003	866.1200832
DMBT1	24.81089953	33.94202402	32.73176694	93.86557798	88.26481845	76.30577392
PUSL1	624.2422322	575.0178188	469.4670573	378.1699728	333.572236	328.0157295
CMTM3	37.71256729	44.92326709	39.27812033	99.28089978	107.7518563	98.10742361
DENND1C	241.1619434	233.6009889	188.9090549	477.4508726	363.3759409	334.952618
GAGE2A	1726.838607	1645.18987	1548.680173	1052.377538	1314.801906	914.678303
TMEM63A	827.6916083	837.5693576	771.5345065	1151.658438	1045.422265	1235.757144
KMT2A	1432.085121	1810.906811	1885.349776	2611.087664	2191.718609	2810.430842
CRCP	621.2649242	619.9410859	656.5057255	908.8715098	988.1074481	830.4446565

UTP23	421.785292	435.2565434	442.3464504	667.8896895	605.2444694	631.256857
RACK1	24535.00233	19860.07723	19074.20339	27308.56532	32167.36799	29230.06633
DCAF8	644.0909518	636.9120979	652.7649522	878.1846863	930.7926309	891.8856692
COA3	552.7868415	488.1661691	465.7262839	314.0886648	351.9129775	293.3312868
CCDC28A	217.3434799	173.7032994	179.5571215	301.4529139	348.4740884	324.0517932
FLRT2	198.4871962	202.6538493	211.3536951	358.3137929	306.0611237	346.8444269
NLE1	1216.726513	970.3425692	923.9710211	1583.981628	1636.911179	1382.422787
RPUSD3	724.4782663	604.9666635	562.9863914	444.9589418	390.8870531	363.6911562
SLC39A4	621.2649242	511.12695	435.800097	300.5503603	322.1092725	331.9796658
ATP5MD	1352.690242	1172.996419	1110.074496	739.1914266	930.7926309	796.7511978
TMEM102	171.6914248	171.7067098	134.6678411	85.74259527	73.36296599	62.43199685
LYRM2	545.8397897	565.0348705	541.4769446	743.7041948	839.0889235	785.850373

GPAA1	2495.976<br>493	2439.832<br>55	2086.416<br>344	1741.025<br>961	1732.053<br>775	1692.600<br>803
TFG	2443.377<br>386	2430.847<br>897	2522.216<br>441	3059.656<br>821	3255.481<br>616	3203.851<br>521
MANEAL	239.1770<br>715	208.6436<br>183	200.1313<br>75	132.6753<br>843	89.41111<br>479	107.0262<br>803
C1orf122	511.1045<br>303	411.2974<br>676	378.7533<br>032	224.7358<br>55	248.7463<br>065	290.3583<br>345
SELENOO	683.7883<br>911	632.9189<br>186	627.5147<br>32	473.8406<br>581	432.1537<br>215	390.4477<br>263
KLHL18	373.1559<br>289	436.2548<br>382	441.4112<br>571	654.3513<br>849	589.1963<br>206	635.2207<br>933
PPP1R3D	249.1014<br>313	195.6657<br>856	253.4373<br>955	142.6034<br>742	115.7759<br>307	95.13447<br>138
SAA1	174.6687<br>327	127.7817<br>375	104.7416<br>542	59.56853<br>987	61.90000<br>255	41.62133<br>123
LRPAP1	1623.625<br>265	1626.222<br>269	1304.594<br>711	1045.157<br>109	1101.590<br>786	1052.425<br>09
SKP1	2364.974<br>943	2165.301<br>474	2214.537<br>832	1694.093<br>172	1780.198<br>221	1598.457<br>316
VRK1	698.6749<br>308	741.7330<br>544	741.6083<br>196	472.0355<br>508	523.8574<br>29	516.3027<br>041
ST3GAL2	431.7096<br>518	407.3042<br>883	408.6794<br>901	280.6941<br>803	252.1851<br>956	261.6197<br>963

GLRX5	950.75367	788.6529112	742.5435129	548.7526097	572.0018754	542.0682901
INPP5J	86.34193037	95.83630313	85.10259405	166.0698687	173.0907479	186.3050065
FAM45A	279.8669467	283.5157301	275.8820357	177.803066	163.9203771	117.9271052
HELZ	1055.951884	1529.387671	1546.809786	2242.845782	1851.268595	2435.838861
ING3	186.5779645	182.6879528	218.8352418	306.8682357	354.2055701	329.0067135
P2RY1	729.4404462	837.5693576	876.2761607	585.7573087	563.977801	498.4649907
CEACAM1	131.0015495	97.83289278	115.9639743	45.12768172	48.14444643	50.54018792
KRI1	943.8066181	908.4482901	851.0259405	634.495205	676.3148427	564.8609238
IGSF11	73.44026261	97.83289278	101.9360742	172.3877442	184.5537113	190.2689428
TOMM22	1326.886907	1219.916275	1250.353497	889.0153299	988.1074481	879.0028762
ZHX2	233.2224556	226.6129251	261.8541355	368.2418828	386.3018678	403.3305193
PML	944.7990541	891.4772781	893.1096409	692.2586376	620.1463218	614.4101277

ALCAM	3006.088587	3503.016538	3622.939004	4341.282981	4608.111301	4614.021862
VPS54	410.8684962	405.3076986	457.3095439	620.9569005	625.8778036	626.3019366
PRX	83.36462242	73.87381699	76.68585398	29.78426994	22.92592687	26.75657008
NR2F1	128.0242416	134.7698013	115.028781	52.3481108	59.60740986	56.48609238
CHRNB1	251.0863032	219.6248613	221.6408219	352.8984711	436.7389069	357.7452518
AC003665.1	292.7686145	276.5276663	280.5580024	177.803066	129.5314868	171.4402453
THUMPD2	288.7988705	290.5037939	286.1691624	406.1491355	473.4203899	473.6903888
SDHAF3	859.4495597	779.6682577	812.6830135	595.6853987	585.7574315	525.2215608
JUP	14159.08414	13396.11825	12392.24696	16579.91026	17546.35813	17013.21463
UXS1	635.159028	732.748401	670.5336257	463.0100145	471.1277972	460.8075958
TMEM143	139.9334734	117.7987893	136.5382278	55.0557717	66.48518792	56.48609238
G6PC3	961.6704658	819.6000507	838.8684271	651.643724	546.7833559	583.6896213

TNRC6A	1280.242416	1423.568419	1471.994319	1960.346494	1751.540813	2012.68866
PRKG1	11.90923177	18.96760166	33.66696028	80.32727346	75.65555867	69.36888538
ZDHHC16	863.4193037	776.6733733	772.4696999	562.2909142	562.8315047	566.842892
SSNA1	1240.544977	994.301645	929.5821812	725.6531221	731.3370672	647.1126022
RNF212	1217.718949	1237.885582	1196.112283	875.4770254	900.988926	923.5971597
DEGS1	683.7883911	587.9956515	709.811746	455.7895854	443.6166849	430.0870894
EMG1	1900.514904	1676.13701	1506.596473	1198.591226	1276.974127	1061.343946
CYBA	4622.7668	3873.383918	3367.631222	2633.651505	3011.320494	2529.982348
SLC35G1	280.8593827	274.5310767	305.8082226	183.2183878	157.0425991	162.5213886
ZDHHC17	302.6929743	435.2565434	428.3185503	643.5207413	578.8796535	666.9322838
MTR	986.4813653	1118.090203	1294.307584	768.0731429	730.1907708	815.5798953
ZNF675	88.32680233	78.86529112	101.0008809	27.07660903	20.63333418	42.61231531

HIST1H4H	69.47051869	107.815841	83.23220737	158.8494397	209.7722309	179.3681179
TAF1	544.8473537	674.8473012	635.931472	919.7021535	824.187071	1045.488201
ZNF324B	210.396428	175.6998891	155.2420946	100.1834534	74.50926233	87.20659877
PIGL	233.2224556	229.6078096	244.0854621	125.4549552	127.2388941	143.6926912
NSMCE1	864.4117396	744.7279389	680.8207524	544.2398415	505.5166875	464.7715321
FAM83F	44.65961915	24.95737061	26.18541355	95.67068525	132.9703758	68.37790131
PUM1	2180.381851	2315.045697	2316.473906	2917.9559	2837.08345	3242.4999
DPM3	540.8776098	415.2906469	304.8730292	239.1767131	233.8444541	201.1697676
CR2	138.9410374	110.8107255	156.177288	250.0073567	226.966676	272.5206212
PIR	403.9214444	386.340097	378.7533032	231.956284	261.3555663	246.7550352
SDF2L1	736.3874981	536.0843206	476.0134107	356.5086856	367.9611263	276.4845575
MICOS13	643.0985158	512.1252448	432.0593236	340.2627202	311.7926054	298.2862072

HOMER3	977.5494415	876.5028557	847.2851672	634.495205	665.9981756	575.7617487
ARHGEF16	1114.505607	1160.018586	964.1843348	738.2888729	792.0907734	752.1569144
OPA1	2696.448561	2888.066927	3205.842774	2209.451297	2183.694534	2206.921539
LRRC20	207.4191201	171.7067098	151.5013213	91.15791708	74.50926233	92.16151915
PTP4A1	12.90166776	22.96078096	14.02790012	71.30173712	59.60740986	52.52215608
AFMID	609.3556925	522.1081931	497.5228575	370.9495437	350.7666811	334.952618
ADPRH	13.89410374	3.993179297	7.48154673	42.42002082	51.58333546	40.63034715
LDLRAD3	403.9214444	407.3042883	462.9207039	629.0798832	621.2926182	625.3109525
UBE2Q2	235.2073275	278.524256	258.1133622	152.5315642	152.4574137	132.7918663
HS1BP3	354.2996453	324.4458179	341.3455695	537.0194125	637.340767	458.8256276
PPARD	774.1000653	781.6648474	769.6641198	555.0704852	472.2740935	571.7978124
LCA5	83.36462242	91.84312383	118.7695543	171.4851905	201.7481565	215.0435447

UBB	2782.790491	2468.7831	2266.908659	1760.882141	1964.751933	1702.510644
MELK	2144.654155	2195.250319	2260.362306	1607.448023	1678.177847	1753.050832
DSE	900.139435	987.3135812	981.0178149	1280.723607	1388.164872	1257.558794
DDX17	5860.334469	6536.834509	6651.095043	8231.289146	8104.315149	8077.511211
TRAPPC6B	110.1603939	113.80561	104.7416542	207.5873359	196.0166747	202.1607517
VKORC1L1	760.2059616	782.6631422	836.0628471	585.7573087	489.4685387	559.9060035
RRS1	900.139435	817.6034611	801.4606934	608.3211496	607.5370621	535.1314015
EIF3L	3836.757503	3596.856252	3772.569939	4536.234567	4977.218724	4865.731818
PYM1	876.3209714	845.5557161	827.646107	587.562416	648.8037304	555.9420671
PAG1	104.205778	146.7493392	102.8712675	237.3716058	192.5777857	264.5927485
EIF3B	9608.76517	8878.834167	8720.677907	7162.665643	7339.735488	6916.077873
C9orf64	172.6838607	193.6691959	181.4275082	345.678042	327.8407542	266.5747167

CDPF1	122.0696257	148.7459288	119.7047477	64.08130804	55.02222449	55.49510831
PPFIBP1	2230.996086	2461.795037	2504.447768	3083.123215	3080.098275	3231.599075
CDK5R1	155.8124491	193.6691959	178.6219282	96.57323888	89.41111479	79.27872615
ANKRD36	329.4887458	277.5259611	318.9009294	175.9979587	102.0203746	193.241895
METTL9	1129.392147	1194.958905	1151.223003	814.1033782	906.7204077	807.6520227
ODF2L	669.8942873	742.7313492	744.4138996	994.6141051	969.7667066	1036.569344
ILVBL	822.7294284	745.7262337	646.2185988	500.0147135	520.41854	393.4206785
TIMM10	756.2362177	634.9155082	555.5048447	443.1538345	414.9592764	383.5108378
FLRT3	157.797321	128.7800323	146.8253546	83.03493437	56.16852083	50.54018792
STOM	2157.555823	2359.968965	2524.086828	1759.077033	1812.294519	1699.537692
TCTN3	471.4070911	571.0246395	490.0413108	334.8473984	331.2796433	331.9796658
ACTL10	56.56885093	42.92667744	61.72276052	10.83064361	14.90185247	14.86476115

PNISR	1581.942954	1588.287065	1797.441602	2092.119325	2245.594537	2376.379816
AURKA	2198.245698	2178.279307	2200.509932	1761.784694	1636.911179	1651.970456
MCUB	252.0787392	304.4799214	239.4094954	162.4596542	145.5796356	137.7467867
EFS	162.7595009	184.6845425	168.3348014	100.1834534	46.99815008	79.27872615
FIS1	2137.707104	1707.084149	1693.635141	1365.563649	1315.948202	1184.225972
LVRN	20.84115561	32.9437292	24.31502687	74.91195166	76.80185502	78.28774208
CENPL	342.3904135	333.4304713	414.2906502	616.4441323	530.735207	542.0682901
SNRPD1	1488.653972	1390.62469	1474.799899	1074.038825	1125.663009	1054.407058
SWI5	443.6188836	454.224145	402.1331367	261.740554	295.7444566	277.4755415
ZNF512	230.2451476	270.5378974	259.9837489	362.826561	413.81298	459.8166117
NQO1	2233.973394	2009.567481	2137.851978	1585.786736	1683.909329	1554.854017
ZNF133	270.9350229	270.5378974	239.4094954	162.4596542	138.7018576	145.6746593

YIPF3	862.4268677	740.7347596	748.154673	1068.623503	1139.418565	1080.172644
MRPL24	1086.717399	972.3391588	808.9422402	660.6692604	669.4370646	539.0953378
WDSUB1	100.2360341	118.7970841	95.38972081	207.5873359	193.7240821	190.2689428
RNF13	1989.834142	2003.577712	2131.305625	2524.442515	2801.548264	2689.530785
GAMT	697.6824948	533.0894362	543.3473313	421.4925473	346.1814957	325.0427772
GLI1	42.67474719	36.9369085	46.75966706	88.45025617	114.6296344	109.9992325
DNAJC13	1168.09715	1419.57524	1509.402053	1945.905636	1788.222296	2139.534622
APCDD1L	70.46295467	59.89768946	59.85237384	25.27150176	5.731481718	14.86476115
HACD3	1536.290899	1516.409838	1503.790893	1206.714209	1090.127823	1108.911182
DMXL2	935.8671303	1219.916275	1367.252665	1799.691947	1584.181547	2023.589485
RNF34	1125.422403	1037.228322	1148.417423	796.9548592	810.4315149	811.615959
ADGRA2	11.90923177	13.97612754	13.09270678	59.56853987	50.43703911	44.59428346

PSMB10	248.1089953	235.5975785	161.788448	116.4294188	96.28889286	109.0082485
FBXW9	296.7383584	285.5123197	298.3266759	186.8286023	182.2611186	151.6205638
CCNA1	155.8124491	116.8004944	145.8901612	247.2996958	298.0370493	225.9443695
UNK	111.1528299	82.85847041	87.90817408	170.5826369	222.3814906	173.4222135
NCAPG2	1740.732711	1787.94603	1895.636903	1372.784078	1290.729683	1416.116246
POLR2E	3183.734628	2842.145365	2571.781688	2191.400224	2137.842681	1931.427966
LIPA	961.6704658	1121.085088	1077.342729	714.8224785	795.5296624	738.2831373
GATAD2B	979.5343135	1042.219797	1234.45521	1452.208798	1578.450065	1551.881064
HSPD1	10808.62027	10434.1775	10810.83502	13123.12984	14459.38208	12988.8283
OGFR	2094.03992	1835.864182	1765.645028	1406.178562	1458.088949	1265.486666
LZTS2	841.5857121	798.6358594	793.9791467	615.5415787	562.8315047	521.2576245
REXO5	164.7443729	146.7493392	196.3906017	70.39918348	83.67963308	93.15250323

TYW1	331.4736177	315.4611645	330.1232495	484.6713017	494.0537241	483.6002295
C1GALT1	571.6431252	614.9496117	593.8477717	814.1033782	844.8204052	829.4536724
COL12A1	1273.295364	1493.449057	1577.671167	1104.725649	851.6981832	1004.857854
MAGEA4	5842.470621	5448.693151	5439.084473	4252.832725	4620.720561	4069.971604
GCLC	1289.17434	1362.672435	1545.874593	1007.249856	1040.83708	993.9570291
TRAM1	2170.457491	2308.057634	2518.475668	3066.87725	3105.316795	3003.672737
FRMD5	189.5552724	227.6112199	177.6867348	320.4065402	311.7926054	371.6190288
UBXN7	1402.312041	1770.975018	1855.423589	2453.140778	2268.520464	2380.343753
RAC1	5934.767168	5527.558442	5505.4832	6908.145518	7753.548468	7136.076338
LAD1	4409.393065	4076.037767	3788.468225	2990.160191	3327.698285	2847.097253
CT45A10	92.29654625	69.8806377	102.8712675	148.018796	190.285193	219.007481
RAB3A	104.205778	129.7783272	90.7137541	43.32257445	37.82777934	49.54920385

RPE	931.8973864	1021.255605	1166.186097	1492.823711	1462.674134	1399.269517
TMUB2	709.5917266	616.9462014	580.7550649	891.7229908	1061.470414	867.1110673
SYBU	532.9381219	502.1422966	505.0044043	712.1148175	813.8704039	700.6257424
ASB7	424.7626	472.1934519	520.9026911	685.0382085	661.4129902	755.1298666
OSR2	108.175522	76.86870147	85.10259405	39.71235991	27.51111224	30.72050638
EPB41L4B	239.1770715	289.505499	302.0674492	400.7338137	468.8352045	458.8256276
ALAD	833.6462242	755.709182	727.5804195	574.9266651	495.2000204	526.2125448
MYEF2	44.65961915	29.94884473	34.60215363	95.67068525	84.82592942	102.0713599
TRIM3	177.6460406	250.5720009	210.4185018	111.014097	114.6296344	110.9902166
PCDH18	40.68987523	52.90962569	44.88928038	8.12298271	5.731481718	14.86476115
TRIM36	137.9486014	143.7544547	188.9090549	296.9401457	257.9166773	279.4575097
THRA	529.960814	446.2377864	491.9116975	344.7754883	287.7203822	312.1599842

SPCS1	1324.902035	1159.020291	1089.500243	846.5953091	888.3796662	800.7151341
CD163L1	186.5779645	221.621451	169.2699948	81.2298271	118.0685234	90.179551
CUTA	1584.920262	1275.820785	1202.658637	940.4608871	959.4500395	914.678303
SECISBP2	855.4798158	1033.235143	1007.203229	1306.897663	1322.82598	1388.368692
CES2	811.8126326	783.661437	775.2752799	509.9428034	529.5889107	609.4552073
ATG13	1702.027708	1594.276834	1524.365146	2016.304819	2355.638986	2124.669861
PSMG3	1150.233302	1011.272657	969.7954949	752.7297311	740.5074379	743.2380577
FKBP3	874.3360994	845.5557161	948.286048	588.4649696	691.2166951	580.7166691
B4GALT7	461.4827313	413.2940572	332.9288295	255.4226785	240.7222321	232.8812581
ARL6IP6	701.6522387	778.6699629	759.3769931	543.3372879	529.5889107	496.4830225
ABCA2	1328.871779	1435.547957	1315.817031	1043.352001	965.1815212	1017.740647
GALNT12	135.9637294	95.83630313	97.26010749	52.3481108	42.41296471	35.67542677

ERCC6L2	220.3207878	301.4850369	356.308663	438.6410663	495.2000204	629.2748888
C3orf38	255.0560472	291.5020887	315.160156	440.4461736	456.2259447	440.9879142
DIAPH2	218.3359159	254.5651802	254.3725888	356.5086856	406.9352019	397.3846148
FDFT1	2282.602757	2037.519736	2002.248944	1424.229635	1604.814881	1638.096679
C8orf82	542.8624817	490.1627587	437.6704837	333.0422911	323.2555689	286.3943982
KCTD5	1739.740275	1622.229089	1659.968181	1252.744445	1331.996351	1171.343179
KHSRP	6369.454127	6412.047656	6174.146439	5237.51874	5004.729836	4726.994047
CYP24A1	212.3813	245.5805268	206.6777284	385.3904019	323.2555689	372.6100129
AZIN2	84.3570584	72.87552217	95.38972081	34.29703811	27.51111224	30.72050638
GOLGA6L10	51.60667102	62.89257393	51.43563377	107.4038825	143.2870429	123.8730096
ZNF800	258.0333551	273.5327818	342.2807629	492.7942844	417.251869	499.4559748
ABR	858.4571238	901.4602263	878.1465474	1148.950777	1200.172272	1198.099749

RDH10	340.4055416	379.3520332	392.7812033	257.2277858	213.2111199	215.0435447
SLC37A1	387.0500327	430.2650693	343.2159562	238.2741595	200.6018601	261.6197963
PLPP5	414.8382401	426.27189	409.6146835	251.812464	301.4759383	245.7640511
MRPL50	717.5312144	702.7995563	736.9323529	495.5019453	521.5648363	511.3477837
STAG3	98.25116214	104.8209565	121.5751344	55.0557717	42.41296471	33.69345861
ALG9	99.24359812	110.8107255	107.5472342	175.0954051	210.9185272	207.1156721
KNSTRN	1056.94432	974.3357485	1069.861182	708.504603	761.1407721	778.9134845
MMP7	23.81846355	36.9369085	23.37983353	2.707660903	2.292592687	1.981968154
G3BP1	5901.024344	6336.17725	6661.38217	4886.425377	5064.337246	4881.587563
SURF6	1455.903584	1335.718475	1263.446204	1000.029427	1036.251895	869.0930355
RASGEF1A	479.3465789	497.1508225	453.5687705	283.4018412	308.3537164	341.8895065
KLHL26	281.8518187	241.5873475	230.9927553	138.0907061	162.7740808	106.0352962

CD81	3261.144 634	2783.245 97	2732.634 943	2237.430 46	2199.742 683	2126.651 829
PROCA1	90.31167 429	71.87722 735	88.84336 742	25.27150 176	33.24259 396	33.69345 861
PELI3	449.5734 995	433.2599 537	390.9108 166	234.6639 449	311.7926 054	240.8091 307
SLC35D1	369.1861 85	415.2906 469	462.9207 039	290.6222 703	247.6000 102	222.9714 173
HRK	58.55372 289	52.90962 569	53.30602 045	110.1115 434	130.6777 832	122.8820 255
KEAP1	1610.723 598	1563.329 695	1348.548 798	1065.013 289	1176.100 048	940.4438 89
TRIM2	73.44026 261	120.7936 737	125.3159 077	215.7103 186	196.0166 747	225.9443 695
MRPL52	968.6175 177	800.6324 491	694.8486 525	555.9730 388	567.4166 9	444.9518 505
NDC1	1568.048 85	1819.891 465	1835.784 529	1304.190 002	1232.268 569	1318.008 822
FAM184A	123.0620 617	139.7612 754	108.4824 276	231.0537 304	205.1870 455	234.8632 262
SMYD4	437.6642 677	467.2019 778	471.3374 44	308.6733 43	263.6481 59	320.0878 568
ZNF780B	138.9410 374	159.7271 719	157.1124 813	256.3252 322	270.5259 371	250.7189 715

MTREX	1609.731162	1861.819847	1925.56309	2378.228827	2360.224171	2481.424129
TOR1AIP1	871.3587915	900.4619315	962.3139481	1191.370797	1282.705608	1227.829271
SLC2A11	153.8275771	177.6964787	180.4923149	78.52216619	98.58148554	85.22463061
ABHD12	1317.954983	1191.96402	1080.148309	878.1846863	790.944477	880.9848444
STRADA	228.2602757	208.6436183	222.5760152	120.942187	51.58333546	121.8910415
OLAH	1.984871962	0	1.870386682	22.56384086	29.80370493	25.765586
TMC5	126.0393696	156.7322874	125.3159077	234.6639449	240.7222321	243.7820829
EPHA3	77.41000653	70.87893252	77.62104732	167.874976	127.2388941	167.476309
KIF2A	885.2528952	1007.279478	1124.102396	1473.870085	1318.240795	1457.737577
ADCK5	314.602206	228.6095148	271.206069	163.3622078	147.8722283	149.6385956
TMEM185B	739.364806	744.7279389	787.4327933	1031.618804	1008.740782	1046.479185
PTAFR	296.7383584	328.4389972	296.4562892	185.0234951	178.8222296	198.1968154

RICTOR	2223.056598	3176.574131	3160.0183	4191.459078	4002.866832	4258.258578
GPN3	1116.490479	1014.267541	993.1753284	780.7088938	768.0185502	702.6077105
ARIH2	1591.867314	1668.150651	1671.190501	2172.446598	2130.964903	2071.156721
SLCO4C1	1.984871962	2.994884473	4.675966706	25.27150176	37.82777934	25.765586
ANO1	782.0395532	800.6324491	785.5624066	582.1470942	469.9815008	579.725685
FKBP15	1247.492028	1203.943558	1162.445323	1524.413089	1580.742658	1782.780354
SPATA33	213.373736	192.6709011	210.4185018	122.7472943	114.6296344	99.09840769
SUMF1	410.8684962	317.4577541	342.2807629	231.0537304	205.1870455	213.0615765
CHEK1	999.3830331	974.3357485	1003.462455	713.9199248	687.7778061	768.0126596
SLC20A2	1356.659986	1329.728706	1175.53803	1680.554867	1748.101924	1733.231151
EME2	467.4373472	468.2002726	454.5039638	342.9703811	277.4037151	272.5206212
MEN1	963.6553378	911.4431745	927.7117945	687.7458694	641.9259524	703.5986946

IER5L	549.8095336	585.9990618	584.4958383	404.3440282	410.374091	344.8624588
CYB5R2	237.1921995	205.6487338	253.4373955	129.0651697	144.4333393	112.9721848
CRTC2	987.4738013	796.6392698	762.1825731	1180.540154	1266.65746	1198.099749
SZRD1	2861.192934	2619.525619	2499.771801	2060.529947	2083.966753	1770.888545
SDR42E1	91.30411027	121.7919686	101.9360742	40.61491355	45.85185374	45.58526754
TOR3A	454.5356794	413.2940572	433.9297103	302.3554675	283.1351968	261.6197963
USP18	361.2466972	379.3520332	311.4193826	185.9260487	213.2111199	236.8451944
FUT6	9.924359812	2.994884473	7.48154673	37.90725265	42.41296471	34.68444269
RBBP6	1164.127406	1153.030522	1329.844931	861.0361672	821.8944783	940.443889
PAK6	102.2209061	100.8277772	93.51933412	40.61491355	27.51111224	48.55821977
SMPD3	67.48564672	78.86529112	57.98198716	24.36894813	13.75555612	24.77460192
NAXE	2354.058147	1940.685138	1918.081543	1506.362016	1409.944503	1579.628619

C12orf60	82.37218644	99.82948243	88.84336742	36.10214538	36.68148299	34.68444269
TLE3	710.5841625	649.8899306	649.9593722	494.5993917	439.0314996	459.8166117
SEMA3B	452.5508074	440.2480175	410.5498768	249.1048031	325.5481616	230.8992899
SIRT3	405.9063163	392.3298659	358.1790497	221.1256404	263.648159	247.7460192
KCNK5	174.6687327	149.7442236	122.5103277	70.39918348	81.38704039	52.52215608
MTFR2	263.987971	262.5515388	298.3266759	167.874976	155.8963027	167.476309
POU2F2	11.90923177	9.982948243	12.15751344	54.15321806	44.7055574	41.62133123
HNRNPLL	648.0606957	687.8251339	692.9782658	937.7532261	892.9648516	957.2906183
ITFG1	520.0364542	566.0331654	503.1340176	324.0167548	392.0333495	336.9345861
DPM2	1500.563204	1290.795208	1112.880076	899.8459735	952.5722615	832.4266246
ABCA1	923.9578985	1149.037343	1332.650511	1584.884182	1779.051925	1645.033568
SCRN3	263.987971	279.5225508	260.9189422	413.3695646	417.251869	398.3755989

TUSC2	549.8095336	532.0911413	471.337444	365.5342219	324.4018652	339.9075384
FAM189B	765.1681415	746.7245285	717.2932927	547.8500561	466.5426118	540.0863219
NUP155	2650.796506	3041.80433	3347.056968	2266.312176	2055.309344	2293.137154
PROCR	1167.104714	1084.148179	909.0079277	704.8943885	789.7981807	683.7790131
PSMA3	1735.770531	1729.046636	1673.996081	1300.579787	1350.337093	1294.225204
SNRK	180.6233486	248.5754112	274.9468423	411.5644573	394.3259422	377.5649333
CENPF	3993.562388	4658.04365	5021.053049	3279.879907	3140.851981	3619.073849
COMTD1	632.18172	569.0280498	473.2078307	383.5852946	343.8889031	351.7993473
OSBPL3	1439.032173	1573.312643	1586.087907	2082.191235	1929.216746	2024.580469
TCF20	1793.331818	1843.85054	1715.144588	2302.414321	2211.205647	2500.252826
PPHLN1	1931.280419	1933.697075	1934.915023	1454.013905	1510.818581	1527.106463
CCDC127	652.0304397	613.9513169	647.1537921	449.4717099	440.1777959	451.8887391

NUSAP1	248.1089953	200.6572597	215.0944685	120.942187	118.0685234	130.8098982
ENDOG	160.774629	146.7493392	125.3159077	74.00939802	75.65555867	43.60329938
PLS1	751.2740378	713.7807993	852.8963272	1197.688673	959.4500395	1218.910415
FASTK	1006.330085	998.2948243	880.0169341	733.7761048	624.7315072	677.8331086
STRBP	858.4571238	915.4363538	997.8512951	1225.667836	1229.975977	1337.828504
MEGF6	162.7595009	161.7237615	127.1862944	83.03493437	67.63148427	66.39593315
GLB1L2	171.6914248	164.718646	196.3906017	105.5987752	89.41111479	78.28774208
CCDC112	289.7913065	310.4696903	277.7524223	405.2465818	486.0296497	462.7895639
ESRRA	3130.143085	2813.194815	2462.364067	2167.031276	2009.45749	1585.574523
AC011481.1	3162.893472	2764.278368	2625.087709	2220.281941	2158.476015	1969.085361
CKB	7143.554193	6223.369934	5469.945853	4576.84948	4611.55019	4743.840776
AP3D1	3384.206696	3174.577541	3089.878799	4035.317299	4068.205723	4106.638015

DIAPH3	724.4782663	946.3834934	785.5624066	1207.616763	1098.151897	1217.919431
STXBP5	129.0166776	144.7527495	173.0107681	268.0584294	232.6981577	287.3853823
TBC1D24	354.2996453	364.3776109	351.6326963	238.2741595	210.9185272	230.8992899
WASF2	5298.615704	5330.894362	5251.110611	6435.207413	6562.546567	6614.818713
PHKG2	769.1378854	646.8950461	686.4319125	477.4508726	497.4926131	484.5912136
PCGF1	413.8458042	401.3145194	343.2159562	550.557717	590.3426169	575.7617487
ADPGK	964.6477737	1034.233438	978.2122349	687.7458694	700.3870659	772.96758
RAB20	153.8275771	157.7305822	125.3159077	251.812464	316.3777908	223.9624014
L3MBTL1	380.1029808	418.2855314	420.8370036	265.3507685	199.4555638	281.4394778
RRM1	3378.25208	3397.197287	3792.208999	2772.644765	2578.020477	2788.629192
SIN3A	1759.588995	1729.046636	1850.747622	1291.554251	1334.288944	1428.999039
C9orf40	350.3299014	323.4475231	299.2618692	204.879675	187.9926003	207.1156721

HSD17B8	421.785292	314.4628696	299.2618692	213.9052114	199.4555638	192.2509109
ANP32E	2406.657254	2393.910989	2531.568375	1981.105228	1837.513039	1868.995969
VRK2	297.7307944	330.4355868	299.2618692	461.2049072	447.055574	485.5821977
ZNF24	565.6885093	748.7211182	728.5156128	1076.746486	977.790781	946.3897935
AIG1	942.8141822	858.5335489	982.8882016	1174.222278	1359.507463	1304.135045
SFRP1	89.31923831	79.86358594	85.10259405	35.19959174	34.38889031	28.73853823
ARSJ	276.8896388	336.4253558	381.5588832	545.1423952	459.6648338	587.6535576
TRMT112	4381.604857	3696.685734	3072.110126	2653.507685	2735.063076	2334.758485
CABIN1	1726.838607	1818.89317	1677.736854	1349.317683	1172.661159	1363.59409
PDXK	3122.203597	2760.285189	2679.328923	2221.184494	2141.28157	2165.300208
B3GALT4	166.7292448	170.7084149	119.7047477	62.27620077	89.41111479	56.48609238
KRT17	52604.06918	44317.30213	37630.30966	59573.95519	71504.81961	59987.23913

MRPS15	1546.215259	1397.612754	1379.410178	1000.029427	1165.783381	915.6692871
PARD6G	232.2300196	235.5975785	223.5112086	146.2136888	114.6296344	130.8098982
RBBP5	485.3011948	504.1388862	519.9674977	685.9407621	766.8722538	692.6978698
MIS18A	396.9743925	360.3844316	415.2258435	276.1814121	241.8685285	210.0886243
PLEKHB1	12.90166776	22.96078096	16.83348014	45.12768172	72.21666964	67.38691723
WRB	185.5855285	195.6657856	204.8073417	101.9885607	115.7759307	105.0443122
ANKZF1	1634.542061	1470.488276	1365.382278	1945.905636	2281.129724	1932.41895
RUVBL2	3585.6712	3067.759995	2728.89417	2262.701961	2401.49084	2063.228848
AGPAT1	512.0969663	509.1303604	519.9674977	344.7754883	367.9611263	338.9165543
MAP3K21	454.5356794	503.1405914	508.7451776	351.0933638	271.6722334	325.0427772
IL1RL1	10.91679579	4.991474121	11.22232009	40.61491355	43.55926105	42.61231531
MOAP1	298.7232303	304.4799214	299.2618692	513.553018	496.3463167	396.3936308

RPS6KC1	513.0894023	510.1286552	584.4958383	778.0012329	710.703733	813.5979271
EIF3J	1634.542061	1652.177934	1785.284088	2141.759774	2231.838981	2196.020714
CLCN2	222.3056598	211.6385027	215.0944685	136.2855988	102.0203746	120.9000574
HMMR	1415.213709	1508.423479	1708.598234	1037.93668	1166.929678	1160.442354
MEF2C	181.6157846	165.7169408	182.3627015	82.13238073	81.38704039	109.9992325
GDPGP1	83.36462242	80.86188076	77.62104732	30.68682357	30.95000127	30.72050638
BBX	1380.47845	1690.113137	1842.330882	2511.806765	2089.698234	2397.190482
STK38	413.8458042	404.3094038	415.2258435	604.7109351	660.2666939	545.0412423
RAB3GAP2	1217.718949	1467.493392	1433.651392	1842.111968	1799.685259	1920.527141
STX5	812.8050686	717.7739786	678.9503657	1046.962216	1077.518563	967.2004591
C16orf91	359.2618252	296.4935628	271.206069	192.2439241	189.1388967	165.4943408
INPP5B	302.6929743	269.5396025	329.1880561	196.7566923	181.1148223	159.5484364

CDK8	300.7081023	332.4321765	330.1232495	490.9891771	444.7629813	510.3567996
PI4K2A	777.0773733	707.7910304	680.8207524	945.8762089	1022.496338	1019.722615
STN1	417.8155481	406.3059935	393.7163967	247.2996958	270.5259371	278.4665256
ACAT1	954.7234139	940.3937244	949.2212413	676.0126722	695.8018805	722.4273921
5-Mar	722.4933943	711.7842097	690.1726858	1032.521358	961.7426322	917.6512552
YPEL2	26.79577149	35.93861367	21.50944685	71.30173712	107.7518563	67.38691723
SOCS4	666.9169794	760.7006561	769.6641198	1075.843932	921.6222602	1121.793975
MDFIC	504.1574785	556.0502171	621.9035719	395.3184919	340.450014	379.5469015
SF3B2	7140.576885	6444.991385	6504.269688	8169.012945	9004.157778	8390.662179
MRPL13	1152.218174	1122.083382	1129.713556	743.7041948	917.0370748	797.7421819
ELOC	1680.194116	1592.280245	1447.679292	1127.289489	1236.853755	1142.604641
MRPL4	1205.809717	1004.284593	931.4525679	714.8224785	780.6278099	670.8962201

1-Mar	266.9652789	306.476511	281.4931957	174.1928514	181.1148223	163.5123727
NDUFC2	357.2769532	311.4679852	278.6876157	153.4341178	199.4555638	202.1607517
SNTB1	26.79577149	20.96419131	35.53734697	88.45025617	85.97222576	63.42298092
PEX12	79.3948785	71.87722735	70.13950059	143.5060279	135.2629685	163.5123727
CORO1C	8106.217095	8100.164204	7835.049813	9505.694878	10095.4319	10147.67695
MAGEA3	1826.082205	1577.305822	1601.986194	1213.032085	1327.411166	1083.145596
GPATCH2L	554.7717135	623.9342652	681.7559458	1011.762624	817.3092929	879.9938603
ZNF416	53.59154299	56.90280498	65.46353389	128.1626161	143.2870429	109.0082485
TRMO	188.5628364	167.7135305	162.7236414	256.3252322	306.0611237	305.2230957
C12orf4	270.9350229	289.505499	366.5957898	470.2304435	467.6889082	516.3027041
SLC30A6	625.2346682	672.8507115	712.617326	894.4306517	929.6463346	949.3627457
CNOT9	1360.62973	1424.566714	1362.576698	1029.813697	999.5704115	1098.010357

POLR3D	783.0319892	844.5574213	724.7748395	1063.208181	1035.105598	1134.676768
PXK	241.1619434	229.6078096	265.5949089	426.9078691	328.9870506	433.0600416
KLHL28	152.8351411	177.6964787	167.3996081	278.889073	248.7463065	310.1780161
C4orf48	412.8533682	371.3656746	234.7335286	205.7822286	178.8222296	153.6025319
DNMT3B	370.178621	443.242902	463.8558972	252.7150176	298.0370493	261.6197963
RASSF6	75.42513457	51.91133086	64.52834054	114.6243116	136.4092649	169.4582772
SH3RF3	22.82602757	18.96760166	27.1206069	88.45025617	71.0703733	52.52215608
DHODH	498.2028626	472.1934519	471.337444	328.5295229	334.7185323	314.1419524
SETDB1	567.6733813	582.0058825	543.3473313	760.8527138	809.2852185	776.9315163
PAX9	111.1528299	126.7834427	103.8064609	49.64044989	41.26666837	59.45904461
TRIP13	1955.098883	1831.871003	1857.293976	1404.373455	1325.118573	1536.025319
CKMT1A	142.9107813	115.8021996	111.2880076	53.25066443	58.46111352	59.45904461

STX16	3006.088587	2763.280074	2911.256871	2288.876017	2322.396392	2186.110874
ERCC2	1849.900669	1726.051751	1663.708954	1360.148327	1281.559312	1347.738345
KCNS3	225.2829677	215.631682	202.936955	122.7472943	108.8981526	131.8008822
FBXL19	1117.482915	1009.276067	938.9341146	790.6369837	708.4111403	673.8691723
RPN2	8266.991724	7483.218003	7418.888776	5862.988409	6006.59284	6376.982535
KHDC1	177.6460406	142.7561599	137.4734212	89.35280981	63.04629889	67.38691723
ZBTB18	193.5250163	158.7288771	173.9459615	297.8426994	280.8426042	285.4034142
BTN2A1	449.5734995	371.3656746	354.4382763	593.8802914	592.6352096	562.8789557
SETX	1475.752304	1741.026174	1842.330882	2191.400224	2271.959353	2386.289657
CHMP2B	899.146999	957.3647365	1056.768476	1304.190002	1424.846355	1257.558794
FAM76A	127.0318056	127.7817375	156.177288	83.03493437	43.55926105	50.54018792
KAT2B	130.0091135	97.83289278	130.9270678	242.7869277	198.3092674	213.0615765

COPB2	2624.99317	2706.377269	2755.079583	3370.135271	3350.624212	3421.868018
MFSD10	1361.622166	1210.931622	1046.481349	1665.211455	1812.294519	1586.565507
CHCHD1	532.9381219	489.1644639	435.800097	302.3554675	355.3518665	288.3763664
IGFBP7	2051.365173	1844.848835	1702.051881	1372.784078	1417.968577	1395.30558
DEK	6089.587181	6481.928294	6921.365918	5036.24928	5062.044653	5285.909066
LUZP2	53.59154299	69.8806377	65.46353389	19.85617996	24.07222321	16.84672931
SF3B6	1782.415022	1646.188165	1688.023981	2093.924432	2307.494539	2242.596966
ATP5F1C	2565.447011	2454.806973	2471.716001	1930.562224	2022.06675	1938.364854
CDC123	1326.886907	1204.941853	1193.306703	954.9017452	899.8426297	920.6242075
EPHB3	157.797321	124.786853	101.0008809	63.17875441	56.16852083	51.531172
CFAP74	31.7579514	38.93349815	28.05580024	91.15791708	75.65555867	86.21561469
NACC1	3092.430517	2973.920281	2787.81135	2439.602474	2166.500089	2189.083826

ECD	645.0833878	672.8507115	722.9044528	911.5791708	964.0352249	920.6242075
FBXL16	170.6989888	124.786853	136.5382278	78.52216619	63.04629889	65.40494908
NOTCH3	1528.351411	1693.108022	1579.541553	1278.9185	1161.198196	1180.262036
RO60	637.1438999	664.864353	750.0250597	903.456188	990.4000408	962.2455387
NTNG2	67.48564672	74.87211182	57.04679381	27.97916267	19.48703784	16.84672931
PRDX6	5273.804804	4997.46389	4675.031513	3888.201057	4089.985354	3684.478798
ATP13A3	4983.021062	6818.35365	6941.004979	9345.040331	8161.629966	9028.855925
PLAG1	246.1241233	303.4816266	393.7163967	484.6713017	516.9796509	544.0502582
HKR1	46.64449112	18.96760166	27.1206069	3.610214538	2.292592687	4.954920385
ELOVL7	263.987971	322.4492282	338.5399895	478.3534262	486.0296497	450.897755
TKFC	988.4662373	841.5625368	803.3310801	635.3977586	613.2685438	608.4642232
RCN1	1146.263558	1163.01347	1124.102396	1492.823711	1632.325993	1428.999039

GAN	386.0575967	397.3213401	433.9297103	560.485807	593.7815059	602.5183188
TULP4	505.1499144	613.9513169	596.6533517	820.4212537	762.2870684	872.0659877
NQO2	812.8050686	744.7279389	706.0709726	486.4764089	578.8796535	516.3027041
TRAP1	2567.431883	2398.902463	2299.640426	1862.870701	1701.103774	1959.17552
ADGRA3	768.1454495	831.5795886	870.6650007	586.6598624	456.2259447	622.3380003
CHKA	346.3601574	365.3759057	307.6786093	491.8917308	619.0000255	472.6994047
TDRKH	182.6082205	193.6691959	187.0386682	332.1397375	277.4037151	301.2591594
MPG	384.0727247	379.3520332	323.5768961	226.5409622	243.0148248	220.9894492
RAPGEF5	634.166592	771.6818992	864.1186473	526.1887689	406.9352019	530.1764811
SLC25A40	710.5841625	821.5966404	903.3967676	583.0496478	537.6129851	569.8158442
PHETA1	265.972843	226.6129251	250.6318154	144.4085815	155.8963027	138.7377708
CLP1	543.8549177	499.1474121	432.0593236	296.0375921	339.3037177	330.9886817

ZFPM2	41.68231121	64.88916358	51.43563377	138.9932597	98.58148554	121.8910415
PRADC1	185.5855285	174.7015942	156.177288	101.0860071	93.99630017	62.43199685
PSME2	2121.828128	1846.845425	1760.033868	1350.220237	1531.451915	1308.098982
MCM8	261.0106631	286.5106146	346.9567296	167.874976	167.3592662	194.2328791
MRPL43	852.5025079	804.6256284	686.4319125	506.3325889	580.0259498	535.1314015
NEK9	738.37237	808.6188076	807.0718535	588.4649696	565.1240974	536.1223856
IQGAP3	2132.744924	2184.269075	2349.205673	1704.923815	1450.064875	1787.735275
PUS7	1221.688693	1376.648563	1490.698186	1015.372839	865.4537394	1037.560329
CSNK2A3	89.31923831	104.8209565	137.4734212	178.7056196	232.6981577	223.9624014
CTRB1	0	2.994884473	0	36.10214538	26.3648159	14.86476115
TTPAL	740.357242	811.6136921	880.9521274	1125.484382	1108.468564	1086.118548
ATP1A1	11238.34505	10538.99846	10345.10874	8716.863001	8244.163303	8834.623046

ARID4A	210.396428	217.6282717	268.4004889	373.6572046	334.7185323	420.1772486
C17orf80	418.8079841	459.2156192	461.0503172	665.1820286	599.5129877	645.1306341
RALY	4977.066446	4357.556908	4334.621137	3477.539153	3660.124225	3484.300014
PHLDA2	1032.13342	886.4858039	814.5534002	1241.011247	1479.868579	1200.081717
CHURC1	258.0333551	296.4935628	303.9378359	402.5389209	451.6407593	456.8436595
C12orf57	475.376835	368.3707902	283.3635824	203.0745677	237.2833431	197.2058313
SETD6	617.2951803	652.8848151	594.782965	410.6619037	479.1518716	389.4567422
TMEM208	656.9926196	550.0604482	539.6065579	415.1746718	385.1555714	378.5559174
PPM1D	232.2300196	253.5668854	249.6966221	371.8520974	363.3759409	391.4387104
LMAN2	2381.846355	2112.391848	2017.212037	1555.099912	1741.224146	1582.601571
HINT3	377.1256729	396.3230452	375.0125298	572.2190042	569.7092827	518.2846722
CD63	8573.654442	7130.81993	6505.204882	5170.729772	5955.009505	4372.221747

JMJD8	408.8836243	404.3094038	337.6047962	247.2996958	236.1370468	252.7009396
ZNF268	254.0636112	257.5600647	290.8451291	381.7801874	441.3240923	410.2674078
UNC119B	2202.215442	2364.960439	2328.63142	1918.829027	1658.690809	1639.087663
LSM6	358.2693892	299.4884473	279.622809	204.879675	193.7240821	155.5845001
GTF3C5	2261.761601	2007.570892	1892.831323	1547.879483	1600.229696	1481.521195
TINAGL1	8991.46999	8747.05925	7561.973357	6740.270542	6578.594715	6264.01035
HYLS1	192.5325804	209.6419131	209.4833084	113.7217579	96.28889286	128.82793
CXXC5	299.7156663	273.5327818	282.4283891	180.5107269	174.2370442	175.4041816
SCRN2	813.7975046	717.7739786	614.4220252	507.2351425	481.4444643	465.7625161
EIF5	4508.636663	4644.067522	5114.572383	6228.522631	5929.790985	5916.174939
CASP1	1312.000367	1214.924801	1237.26079	932.3379043	974.351892	939.4529049
PDCD6IP	2143.661719	2300.071275	2384.74302	2775.352426	2986.101975	3021.510451

BTBD11	417.8155481	369.369085	320.771316	248.2022495	224.6740833	218.0164969
HNRNPA2B1	24517.13848	24199.66483	25178.21033	20026.76259	20874.05642	18484.82599
SCRIB	3106.324621	2993.886178	2641.921189	2355.664986	2182.548238	2075.120657
NDUFA4	2805.616519	2346.991132	2267.843852	1727.487656	1993.409341	1662.871281
SLC29A4	90.31167429	87.84994453	71.07469393	150.7264569	150.164821	183.3320542
CDK4	4135.480734	3752.590244	3536.901217	2985.647423	2973.492715	2926.375979
PCGF6	453.5432434	479.1815156	504.0692109	316.7963257	304.9148274	346.8444269
DPYSL5	27.78820747	17.96930684	26.18541355	79.42471983	49.29074277	89.18856692
ACOT13	369.186185	359.3861367	406.8091034	234.6639449	253.3314919	251.7099555
FMNL1	164.7443729	124.786853	131.8622611	238.2741595	279.6963078	222.9714173
PKD2	276.8896388	253.5668854	323.5768961	183.2183878	142.1407466	175.4041816
KNTC1	2236.950702	2757.290305	2981.396372	1908.900937	1539.475989	2050.346055

FAM135A	256.0484832	326.4424075	408.6794901	551.4602706	461.9574264	670.8962201
APOL3	203.4493761	183.6862477	177.6867348	99.28089978	119.2148197	88.19758285
LAMP2	2700.418305	2882.077158	3076.786093	2283.460695	2266.227871	2193.047762
C16orf58	515.0742743	437.253133	471.337444	285.2069485	328.9870506	330.9886817
FOXP2	146.8805252	198.66067	230.0575619	322.2116475	322.1092725	332.9706498
HSPBP1	2292.527117	1922.715832	1851.682816	1531.633518	1544.061175	1324.945711
CTDSP1	1526.366539	1397.612754	1350.419185	1907.998383	1923.485264	1775.843466
TSHZ1	90.31167429	103.8226617	102.8712675	42.42002082	30.95000127	50.54018792
PTPN2	820.7445565	830.5812938	831.3868803	1008.15241	1223.098199	1183.234988
TBPL1	268.9501509	315.4611645	280.5580024	395.3184919	484.8833533	449.9067709
PFKM	2132.744924	2045.506095	2024.693584	1686.872743	1557.816731	1573.682714
GNA12	1645.458857	1547.356978	1533.71708	1267.185303	1135.979676	1188.189908

RPL36AL	1659.352961	1408.593997	1385.956532	1024.398375	1184.124123	832.4266246
SLC39A7	3335.577333	3000.874242	2827.089471	2389.05947	2330.420466	2342.686358
PLEKHO1	189.5552724	194.6674907	168.3348014	91.15791708	96.28889286	112.9721848
UBASH3B	471.4070911	430.2650693	465.7262839	334.8473984	277.4037151	293.3312868
POLR2J	1251.461772	1025.248785	909.0079277	725.6531221	735.9222525	739.2741214
SUN3	291.7761785	291.5020887	285.2339691	459.3997999	527.296318	377.5649333
GGCT	1110.535863	975.3340433	949.2212413	755.437392	740.5074379	714.4995195
ATP1B1	1312.000367	1213.926506	1405.595592	952.1940843	1011.033375	973.1463635
PIK3C2A	1375.51627	1690.113137	1817.080662	2359.2752	2101.161198	2301.065027
VPS18	346.3601574	336.4253558	352.5678896	196.7566923	205.1870455	249.7279874
RPL19	17259.45415	15057.28083	13906.32498	19013.19486	22316.09722	20016.88737
EMP2	1184.968562	1393.619575	1384.086145	1003.639641	981.2296701	942.4258571

LTB4R	321.5492579	320.4526386	300.1970625	189.5362632	202.8944528	205.1337039
RAP2A	397.9668285	472.1934519	480.6893774	682.3305476	641.9259524	625.3109525
FRA10AC1	283.8366906	268.5413077	308.6138026	429.61553	442.4703886	418.1952805
FLOT2	2791.722415	2412.87859	2453.012134	1964.859262	2025.505639	1859.086128
LAS1L	1114.505607	1029.241964	983.823395	787.0267692	749.6778087	774.9495481
SSTR2	38.70500327	25.95566543	29.92618692	4.512768172	4.585185374	5.945904461
DLG5	1452.926276	1512.416659	1528.10592	2033.453338	1790.514889	2079.084593
GJB5	557.7490214	480.1798105	418.9666169	669.6947967	746.2389196	722.4273921
SNRPD3	672.8715953	660.8711737	663.0520789	487.3789626	497.4926131	403.3305193
TLE2	52.599107	56.90280498	44.88928038	111.9166507	97.4351892	130.8098982
EZH1	530.9532499	480.1798105	513.4211443	722.9454612	671.7296573	738.2831373
TRIM26	1358.644858	1178.986187	1201.723443	913.384278	964.0352249	897.8315737

PEX6	267.9577149	162.7220564	165.5292214	93.86557798	110.044449	91.17053508
FBXO32	319.564386	307.4748059	340.4103762	170.5826369	226.966676	201.1697676
FAM234B	237.1921995	302.4833317	284.2987757	427.8104227	429.8611288	406.3034715
CYB5R4	478.3541429	524.1047827	542.4121379	702.1867276	798.9685514	692.6978698
MED23	391.0197766	422.2787107	489.1061175	640.8130804	593.7815059	666.9322838
SRSF3	6225.55091	5993.762125	6026.385891	7470.436432	7837.228101	7247.066554
HMGA1	20409.44595	18737.99385	18062.32419	14607.83057	16057.31918	12834.23478
ITPA	1065.876244	896.4687522	867.8594207	706.6994957	677.461139	599.5453665
MYLK	128.0242416	130.776622	159.9180613	64.08130804	69.92407695	74.32380577
DEPDC7	172.6838607	220.6231562	208.5481151	96.57323888	102.0203746	127.8369459
NDUFAF7	282.8442546	288.5072042	291.7803225	426.0053154	405.7889056	452.8797231
CEP78	986.4813653	1162.015175	1251.288691	820.4212537	668.2907683	854.2282743

STARD7	2418.566486	2365.958733	2471.716001	1953.126065	1885.657485	1902.689428
HIST1H2AC	528.968378	472.1934519	476.948604	657.9615995	824.187071	673.8691723
HSBP1	1122.445095	963.3545054	890.3040608	762.6578211	661.4129902	659.9953952
TGDS	175.6611687	176.6981839	163.6588347	274.3763049	298.0370493	263.6017645
AP3M1	936.8595663	1069.173757	1081.083502	1340.292147	1338.874129	1475.575291
NIPSNAP3B	49.62179906	44.92326709	35.53734697	13.53830452	3.438889031	10.90082485
PBXIP1	622.2573602	534.087731	526.5138511	411.5644573	374.8389043	352.7903314
JOSD2	351.3223373	275.5293715	225.3815952	179.6081732	155.8963027	136.7558026
ERGIC1	2914.784477	2637.494926	2547.466661	3345.766323	3579.883481	3458.534428
FRRS1	239.1770715	289.505499	285.2339691	418.7848864	401.2037202	417.2042964
TMEM158	166.7292448	130.776622	139.3438078	71.30173712	85.97222576	44.59428346
REPS1	1026.178805	1040.223207	1075.472342	1313.215538	1396.188946	1404.224437

TBRG1	353.3072<br>093	378.3537<br>384	399.3275<br>567	549.6551<br>634	562.8315<br>047	520.2666<br>404
TSPAN13	819.7521<br>205	858.5335<br>489	888.4336<br>742	675.1101<br>185	602.9518<br>767	560.8969<br>875
USP54	392.0122<br>126	467.2019<br>778	476.0134<br>107	644.4232<br>95	621.2926<br>182	653.0585<br>067
TIMM50	1210.771<br>897	1068.175<br>462	982.8882<br>016	774.3910<br>183	801.2611<br>441	792.7872<br>615
NABP2	1835.014<br>129	1557.339<br>926	1435.521<br>779	1167.001<br>849	1200.172<br>272	1160.442<br>354
SMARCA<br>D1	633.1741<br>56	697.8080<br>822	714.4877<br>127	944.9736<br>552	874.6241<br>101	988.0111<br>247
HSD11B2	94.28141<br>822	89.84653<br>418	117.8343<br>61	39.71235<br>991	32.09629<br>762	53.51314<br>015
MAP3K15	7.939487<br>85	14.97442<br>236	14.02790<br>012	46.93278<br>899	43.55926<br>105	49.54920<br>385
CFLAR	448.5810<br>635	485.1712<br>846	455.4391<br>572	650.7411<br>704	676.3148<br>427	623.3289<br>844
SLC2A9	184.5930<br>925	180.6913<br>632	173.0107<br>681	260.8380<br>003	310.6463<br>091	299.2771<br>912
BTF3L4	929.9125<br>144	985.3169<br>915	996.9161<br>017	1257.257<br>213	1407.651<br>91	1230.802<br>224
BCL7A	1411.243<br>965	1334.720<br>18	1281.214<br>877	1030.716<br>25	957.1574<br>468	1051.434<br>106

TMEM167B	742.3421139	728.7552217	749.0898663	1030.71625	1122.22412	911.7053508
IRAK1	4723.002835	4348.572254	4088.665288	3590.358358	3412.524215	3206.824473
PMS2	368.193749	415.2906469	439.5408704	275.2788585	254.4777883	273.5116052
GRHL1	113.1377019	112.8073151	95.38972081	188.6337096	185.7000076	197.2058313
BTC	166.7292448	177.6964787	176.7515415	74.00939802	80.24074405	114.9541529
COL4A6	1073.815732	1185.974251	1198.917863	1658.89358	1525.720433	1433.953959
TLK1	608.3632565	677.8421857	731.3211928	892.6255444	967.4741139	930.5340482
KCTD3	1306.045751	1410.590587	1501.920506	1040.64434	942.2555944	1123.775943
DDX50	873.3436635	795.6409749	798.6551134	1114.653738	1126.809306	1058.370994
AIFM1	829.6764803	733.7466958	770.5993132	578.5368797	565.1240974	543.0592741
MARK2	1676.224372	1792.937504	1777.802542	2612.892772	2340.737133	2077.102625
ISL2	45.65205514	36.9369085	63.5931472	136.2855988	89.41111479	118.9180892

SUPT6H	3750.415573	3649.765877	3464.891329	4365.65193	4507.237223	4857.803945
STOML2	3446.730163	3122.66621	2938.377478	2195.912993	2516.120474	2504.216762
GBP3	321.5492579	320.4526386	350.697503	479.2559799	476.8592789	488.5551499
PRDM11	321.5492579	307.4748059	294.5859025	444.9589418	440.1777959	472.6994047
WDR36	1349.712934	1551.350157	1627.236414	2065.042716	1894.827856	2068.183769
SLC49A4	350.3299014	398.3196349	421.7721969	530.701537	637.340767	561.8879716
AGPAT4	433.6945238	429.2667744	410.5498768	577.634326	610.9759511	606.4822551
DAP3	2230.996086	2013.560661	2164.972585	2611.087664	2942.542714	2720.251291
PNPLA3	93.28898223	138.7629806	118.7695543	54.15321806	42.41296471	59.45904461
TSPYL4	557.7490214	591.9888308	618.1627986	429.61553	424.1296471	382.5198537
DUSP11	571.6431252	549.0621533	554.5696513	748.2169629	772.6037355	767.0216755
MCF2L	350.3299014	308.4731007	336.6696028	483.768748	500.9315021	469.7264525

AMPD2	1050.989704	890.4789832	874.405774	721.1403539	627.0240999	652.0675226
DAAM1	463.4676032	490.1627587	524.6434644	692.2586376	672.8759536	696.6618061
KLF16	968.6175177	864.5233178	810.8126268	683.2331012	577.7333571	611.4371755
CCDC117	602.4086406	686.8268391	723.8396461	483.768748	440.1777959	482.6092455
DOCK1	614.3178724	726.7586321	626.5795386	906.1638489	867.746332	948.3717616
HAUS1	237.1921995	259.5566543	212.2888885	134.4804915	146.725932	137.7467867
PWWP2A	451.5583715	408.3025831	449.8279971	620.9569005	601.8055803	622.3380003
NBEAL1	190.5477084	335.427061	334.7992162	535.2143052	412.6666837	583.6896213
ARF4	2172.442363	2169.294653	2117.277725	2720.296654	3051.440866	2590.432377
L3HYPDH	234.2148916	292.5003835	316.0953493	166.0698687	178.8222296	158.5574523
GCC2	395.9819565	467.2019778	528.3842378	700.3816203	612.1222474	775.9405322
MRPS23	1215.734077	1033.235143	952.9620147	788.8318765	785.2129953	690.7159016

ZNF26	420.792856	454.224145	446.0872238	638.1054195	613.2685438	602.5183188
CYB5R3	2562.469703	2277.110494	2171.518938	1787.95875	1763.003776	1827.374638
HGH1	485.3011948	456.2207347	437.6704837	314.0886648	283.1351968	331.9796658
STRAP	3533.072093	3619.817033	3787.533032	2616.502986	3122.51124	2673.67504
EIF4EBP2	1023.201497	1177.987893	1331.715318	886.307669	786.3592917	842.3364654
NLGN3	0.992435981	2.994884473	4.675966706	24.36894813	24.07222321	28.73853823
C11orf68	1106.566119	887.4840988	916.4894744	1439.573047	1509.672284	1192.153845
MGME1	692.7203149	730.7518114	767.7937331	531.6040907	502.0777985	547.0232105
DCUN1D1	1080.762784	1053.20104	1170.862063	1440.475601	1491.331543	1400.260501
MPDZ	671.8791593	853.5420747	849.1555538	1185.955476	1007.594486	1185.216956
LRWD1	1768.520919	1548.355272	1523.429953	1249.13423	1254.0482	1094.046421
MTRF1L	318.57195	302.4833317	308.6138026	450.3742636	476.8592789	434.0510257

UNC13B	557.7490214	542.0740896	505.9395976	753.6322847	698.0944732	769.9946278
BRWD1	963.6553378	1145.044163	1216.686537	1541.561608	1400.774132	1653.952424
CASZ1	93.28898223	80.86188076	84.16740071	172.3877442	145.5796356	163.5123727
ACOX1	532.9381219	547.0655637	635.931472	379.9750801	384.0092751	417.2042964
MPP3	329.4887458	368.3707902	378.7533032	234.6639449	246.4537139	219.007481
UBR5	3275.038738	4087.019011	4276.639149	5297.08728	4862.589089	5634.735461
ZNF768	835.6310962	831.5795886	698.5894259	1087.577129	1164.637085	1015.758679
SLC10A5	10.91679579	8.984653418	11.22232009	34.29703811	40.12037202	52.52215608
ERI1	426.7474719	437.253133	456.3743505	610.1262569	624.7315072	610.4461914
PIM3	2925.701273	2711.368743	2569.911302	3593.968572	3822.898306	3260.337613
ROGDI	257.0409191	251.5702957	216.9648552	163.3622078	137.5555612	115.945137
MIA3	1018.239317	1126.076562	1190.501123	1425.132189	1483.307469	1501.340877

ENPP4	50.61423504	68.88234287	72.94508062	120.942187	126.0925978	150.6295797
RAD17	507.1347864	498.1491173	534.9305912	698.576513	714.142622	705.5806628
PPCS	581.567485	563.0382809	566.7271648	381.7801874	440.1777959	374.5919811
SETBP1	120.0847537	109.8124307	116.8991677	177.803066	209.7722309	221.9804332
REV3L	451.5583715	614.9496117	634.9962787	800.5650737	811.5778112	920.6242075
ZNF444	682.7959551	643.9001616	596.6533517	903.456188	941.109298	813.5979271
HRH1	351.3223373	376.3571487	338.5399895	546.9475025	482.5907606	513.3297518
SERPINB1	11606.5388	10663.78531	10424.60017	8426.240731	9266.659641	8201.384221
CCDC88A	824.7143004	1095.129422	1197.047477	1641.745061	1303.338943	1727.285246
MT-ND4	126652.6951	121286.8314	121750.9507	90925.05824	100384.6097	105350.5262
UBR7	606.3783845	573.0212291	607.8756718	412.4670109	393.1796458	452.8797231
PPP1R10	1597.82193	1558.338221	1408.401172	1954.931172	1977.361193	1954.2206

PWWP3A	623.2497962	595.9820101	606.0052851	435.0308518	365.6685336	461.7985798
SPRED3	65.50077476	89.84653418	64.52834054	140.798367	135.2629685	159.5484364
ZDHHC6	468.4297831	444.2411968	449.8279971	650.7411704	602.9518767	646.1216181
TMEM147	885.2528952	748.7211182	655.5705322	504.5274816	480.2981679	566.842892
NECAB2	22.82602757	17.96930684	13.09270678	53.25066443	57.31481718	58.46806054
DDAH1	515.0742743	512.1252448	497.5228575	716.6275857	649.9500268	756.1208507
SYNE1	54.58397897	44.92326709	48.63005374	104.6962216	90.55741114	133.7828504
ZBTB47	160.774629	136.7663909	120.639941	83.937488	57.31481718	59.45904461
FAAP20	527.975942	478.1832208	418.9666169	301.4529139	351.9129775	279.4575097
PFKL	4063.032907	3716.651631	3261.954374	2963.986135	2736.209372	2642.954533
ETFDH	231.2375836	218.6265665	249.6966221	378.1699728	312.9389018	408.2854397
TSC1	889.2226392	936.4005452	874.405774	1134.509918	1172.661159	1288.2793

MRPL45	816.7748125	756.7074768	778.0808599	963.0247279	1122.22412	1105.93823
ADPRM	80.38731448	72.87552217	74.8154673	139.8958133	140.9944503	152.6115478
P2RY11	43.66718317	42.92667744	32.73176694	93.86557798	107.7518563	79.27872615
MECR	431.7096518	460.213914	389.9756233	287.9146094	268.2333444	299.2771912
PAPLN	159.782193	163.7203512	172.0755748	92.06047071	72.21666964	100.0893918
SLC35B3	247.1165593	240.5890526	243.1502687	138.0907061	162.7740808	143.6926912
MAP2	237.1921995	254.5651802	233.7983353	359.2163465	377.131497	358.7362358
MUTYH	534.9229939	481.1781053	445.1520304	297.8426994	326.6944579	355.7632836
URM1	1737.755403	1589.28536	1433.651392	1211.226977	1236.853755	1036.569344
IPP	215.3586079	262.5515388	263.7245222	163.3622078	122.6537088	145.6746593
LRRC75B	109.1679579	104.8209565	80.42662735	42.42002082	48.14444643	37.65739492
ARSA	568.6658172	596.9803049	519.9674977	399.83126	390.8870531	391.4387104

AGO1	947.7763621	1023.252195	1046.481349	769.8782501	722.1666964	762.0667551
MFAP5	318.57195	306.476511	288.9747424	422.3951009	469.9815008	449.9067709
LSM5	1185.960998	1140.052689	1097.916983	781.6114474	910.1592968	864.1381151
NPR2	80.38731448	81.86017559	71.07469393	157.946886	134.1166722	150.6295797
PLSCR4	187.5704004	205.6487338	226.3167886	125.4549552	103.1666709	124.8639937
DPP7	1608.738726	1424.566714	1306.465098	1156.171206	1036.251895	939.4529049
EIF4E2	1098.626631	942.3903141	967.9251082	700.3816203	782.9204026	720.4454239
MLH1	892.1999471	923.4227124	934.2581479	651.643724	702.6796586	698.6437742
TMSB4X	14477.65609	13603.76357	12386.6358	10469.62216	11319.67639	9802.814489
PRKCD	621.2649242	505.1371811	490.0413108	342.9703811	357.6444592	385.4928059
IL17RC	410.8684962	367.3724953	369.4013698	245.4945886	276.2574188	233.8722422
CRIM1	1464.835508	1652.177934	1628.171607	2210.353851	1866.170447	2260.434679

CIR1	278.8745107	266.5447181	262.7893289	359.2163465	453.933352	418.1952805
LCMT2	226.2754037	270.5378974	252.5022021	175.0954051	123.8000051	133.7828504
MRPL16	1192.908049	1153.030522	1030.583062	781.6114474	917.0370748	735.3101851
DYNLT1	1033.125856	921.4261228	879.0817408	678.7203331	733.6296599	651.0765385
HID1	76.41757055	81.86017559	83.23220737	138.9932597	165.0666735	150.6295797
ME3	53.59154299	56.90280498	67.33392057	130.870277	99.72778189	142.7017071
PPIE	1096.641759	932.4073659	947.3508547	703.9918348	778.3352172	675.8511405
SYVN1	1543.237951	1537.374029	1400.919625	1820.450681	1972.776007	2017.643581
IRF6	5510.997004	5567.490235	5492.390493	6576.00578	6843.389171	6750.583532
CHIC1	104.205778	97.83289278	112.2232009	211.1975505	173.0907479	176.3951657
HMOX2	1299.098699	1124.079972	984.7585883	799.6625201	875.7704064	685.7609812
ACVR2B	198.4871962	210.6402079	223.5112086	340.2627202	330.1333469	308.1960479

TP53RK	482.3238869	494.155938	536.8009779	721.1403539	674.02225	697.6527901
PDE9A	147.8729612	139.7612754	123.445521	213.0026577	229.2592687	250.7189715
TRIM65	1049.997268	1011.272657	974.4714616	769.8782501	787.505588	716.4814876
PPT2	213.373736	226.6129251	202.0017617	123.6498479	143.2870429	95.13447138
PKN1	1268.333184	1182.979367	1014.684775	1528.023303	1672.446365	1497.37694
CECR2	50.61423504	44.92326709	37.40773365	93.86557798	82.53333673	136.7558026
SLC10A3	465.4524752	437.253133	437.6704837	277.9865194	316.3777908	318.1058887
GPX8	582.559921	530.0945517	619.0979919	754.5348384	840.2352198	802.6971023
HIPK2	673.8640312	799.6341542	805.2014668	1104.725649	944.5481871	1121.793975
COPS6	4902.633747	4187.846788	4034.424074	3317.78716	3496.203848	3295.022056
C19orf81	77.41000653	86.85164971	94.45452746	24.36894813	50.43703911	15.85574523
TAP1	1060.914064	999.2931191	937.9989212	766.2680356	762.2870684	687.7429494

AGPAT3	1558.124491	1507.425185	1411.206752	1167.001849	1079.811156	1179.271052
WIPI1	80.38731448	82.85847041	87.90817408	128.1626161	249.8926029	152.6115478
TMEM14C	1451.933841	1417.57865	1399.984432	1055.085199	1181.83153	975.1283317
CAVIN2	105.198214	102.8243669	105.6768476	42.42002082	50.43703911	53.51314015
GBF1	1557.132055	1471.486571	1681.477628	2039.771214	1914.314894	2264.398616
MYSM1	684.780827	914.438059	1001.592068	1270.795517	1168.075974	1355.666217
TARS2	972.5872616	862.5267282	862.2482606	678.7203331	643.0722487	665.9412997
RPL22	2997.156663	2603.552902	2519.410861	2119.195934	2141.28157	1734.222135
ESS2	464.4600392	452.2275554	400.26275	280.6941803	331.2796433	252.7009396
HDAC4	140.9259093	113.80561	142.1493879	240.0792668	225.8203797	209.0976402
CALB1	7583.203332	8235.9323	8345.665377	9501.182109	11238.28935	10056.50641
TTLL7	97.25872616	117.7987893	104.7416542	196.7566923	173.0907479	193.241895

ZNF584	458.5054233	420.282121	360.9846297	282.4992876	257.9166773	273.5116052
ANKRD9	624.2422322	580.0092929	539.6065579	436.8359591	409.2277946	344.8624588
ALG13	671.8791593	689.8217236	664.9224656	852.0106309	945.6944834	927.561096
DHX9	4920.497595	5283.974505	5559.724414	4297.057853	4001.720535	4255.285626
CCDC58	306.6627182	320.4526386	355.3734697	443.1538345	488.3222423	512.3387678
SPANXB1	33.74282336	24.95737061	28.99099358	3.610214538	6.877778061	3.963936308
ZNF649	8.931923831	12.97783272	15.8982868	47.83534262	37.82777934	52.52215608
CYC1	5318.464423	4455.389801	4251.388929	3605.701769	3688.781633	3348.535196
L2HGDH	398.9592644	431.2633641	447.9576104	311.3810039	283.1351968	262.6107804
BMT2	245.1316874	267.5430129	275.8820357	382.682741	433.3000178	372.6100129
VAPA	3012.043203	2996.881062	2941.183058	3503.713209	4062.474241	3767.72146
SLC35E2B	854.4873798	914.438059	904.331961	1217.544853	1221.951902	1099.001341

VPS52	931.8973864	903.456816	977.2770416	710.3097103	682.0463244	715.4905035
POLM	489.2709387	427.2701848	419.9018102	303.2580212	307.2074201	297.2952231
HNRNPL	3274.046302	3111.684967	2902.840131	2557.837	2426.709359	2184.128906
COX7A2	2088.085304	1844.848835	1652.486634	1386.322382	1390.457465	1405.215421
TMEM216	73.44026261	51.91133086	66.39872723	123.6498479	131.8240795	129.8189141
DCAF13	1412.236401	1438.542842	1411.206752	1112.848631	1100.44449	1108.911182
C22orf39	445.6037556	376.3571487	412.4202635	255.4226785	285.4277895	283.421446
NEURL1B	156.804885	184.6845425	197.325795	300.5503603	345.0351994	250.7189715
SIRPA	146.8805252	161.7237615	127.1862944	86.6451489	74.50926233	56.48609238
SLC17A5	256.0484832	275.5293715	299.2618692	188.6337096	144.4333393	173.4222135
SEC22B	732.4177541	786.6563215	850.0907472	1087.577129	1012.179671	1077.199692
SUGT1	925.9427705	853.5420747	877.2113541	1114.653738	1239.146347	1159.45137

RRAGC	320.5568219	316.4594593	311.4193826	431.4206372	534.1740961	442.9698824
AP2S1	2604.152015	2143.338988	1883.479389	1546.074376	1718.298219	1473.593322
DNAJC25	284.8291266	259.5566543	271.206069	395.3184919	410.374091	397.3846148
AFAP1	987.4738013	967.3476847	958.5731748	1361.050881	1205.903753	1253.594857
KAT6B	309.6400261	353.3963678	423.6425836	540.629627	495.2000204	620.3560321
TMED5	734.4026261	881.4943298	1010.008809	1162.489081	1284.998201	1253.594857
SOD2	3585.6712	3716.651631	4285.055889	5344.922623	5440.322446	4542.671009
IQCE	428.7323439	432.2616589	407.7442968	592.9777378	572.0018754	603.5093028
SS18L2	310.6324621	279.5225508	253.4373955	157.0443324	184.5537113	180.359102
TDP1	271.9274589	309.4713955	330.1232495	203.9771214	194.8703784	174.4131975
GPR107	2517.810084	2535.668854	2461.428874	3140.886648	3064.050126	3087.906384
SSBP1	1597.82193	1530.385966	1398.114045	1089.382237	1241.43894	1068.280835

TCAF1	406.8987523	412.2957624	362.8550164	281.5967339	243.0148248	259.6378282
TBX18	271.9274589	350.4014833	340.4103762	214.807765	145.5796356	208.1066562
MORF4L2	7454.186655	7466.246991	7719.085838	5689.698111	6557.961381	5806.175707
POLR2G	1338.796139	1170.001534	1091.370629	886.307669	938.8167053	766.0306915
IKBKB	617.2951803	626.9291496	533.9953978	839.37488	804.7000331	796.7511978
TMEM131	821.7369924	1017.262426	964.1843348	1224.765282	1232.268569	1353.684249
PI4K2B	565.6885093	601.971779	689.2374925	406.1491355	481.4444643	322.069825
UVRAG	545.8397897	528.097962	484.4301508	699.4790667	710.703733	730.3552647
FAM114A2	303.6854103	313.4645748	293.6507091	445.8614954	437.8852032	435.0420098
ANKRD28	1211.764333	1403.602523	1377.539792	1758.17448	1664.422291	1792.690195
GRK2	4711.093603	4482.343761	4369.22329	5716.77472	5514.831709	5459.33128
STARD13	184.5930925	190.6743114	219.7704352	324.9193084	323.2555689	287.3853823

GPR3	55.57641495	91.84312383	72.00988727	29.78426994	27.51111224	23.78361785
MTX1	398.9592644	406.3059935	349.7623096	257.2277858	273.9648261	227.9263377
PTPRJ	242.1543794	219.6248613	231.9279486	133.5779379	128.3851905	154.593516
EEF1AKMT3	293.7610504	264.5481284	280.5580024	156.1417788	205.1870455	150.6295797
GDAP2	229.2527117	309.4713955	307.6786093	416.9797791	419.5444617	469.7264525
BEX3	2126.790308	1861.819847	1690.829561	1370.978971	1528.013026	1159.45137
DCLRE1B	523.0137621	452.2275554	497.5228575	350.1908101	345.0351994	322.069825
CEP57	1329.864215	1457.510443	1603.85658	1900.777954	1900.559338	1935.391902
MYOF	7215.009583	7854.583677	8005.255001	9428.075265	9205.905935	10045.60559
TXNRD2	709.5917266	604.9666635	546.1529113	425.1027618	416.1055727	440.9879142
FUCA1	180.6233486	181.689658	149.6309346	95.67068525	103.1666709	80.26971023
DPYSL3	1353.682678	1329.728706	1509.402053	1049.669877	946.8407797	1128.730864

NIPAL4	209.403992	158.7288771	192.6498283	101.0860071	104.3129673	109.0082485
SLC9A3	37.71256729	41.92838262	40.21331367	10.83064361	6.877778061	11.89180892
PYGM	12.90166776	9.982948243	0.935193341	36.10214538	49.29074277	35.67542677
PARG	383.0802887	459.2156192	416.1610368	564.9985751	629.3166926	598.5543825
NDE1	767.1530135	798.6358594	783.6920199	1000.029427	1052.300043	1038.551313
DHX57	539.8851738	456.2207347	527.4490445	719.3352466	656.8278048	773.9585641
TBCEL	136.9561654	142.7561599	147.7605479	238.2741595	253.3314919	214.0525606
TUBB2A	183.6006565	165.7169408	135.6030345	89.35280981	72.21666964	93.15250323
NFXL1	293.7610504	339.4202402	331.9936361	455.7895854	451.6407593	500.4469588
ATP5F1A	4407.408193	3891.353225	3751.995685	2949.545277	3294.455691	3122.590826
RCAN3	406.8987523	488.1661691	473.2078307	316.7963257	328.9870506	253.6919237
CNBP	7011.560207	6546.817457	6587.501896	8141.033782	8714.144803	7994.268548

ADTRP	65.50077476	77.86699629	65.46353389	140.798367	134.1166722	127.8369459
BORCS8	248.1089953	176.6981839	206.6777284	120.942187	115.7759307	122.8820255
ZNF41	117.1074458	135.7680961	137.4734212	202.1720141	221.2351943	234.8632262
SLC41A3	508.1272224	478.1832208	435.800097	625.4696686	690.0703988	673.8691723
MRPL2	955.7158499	853.5420747	788.3679867	560.485807	695.8018805	574.7707646
SMARCB1	2449.332002	2166.299769	2015.34165	1577.663753	1721.737108	1735.213119
API5	3045.786026	3366.250147	3616.392651	2627.33363	2445.050101	2700.43161
KLHL23	345.3677215	398.3196349	418.0314235	248.2022495	213.2111199	283.421446
GMPPB	488.2785028	552.0570378	493.7820842	349.2882565	369.1074226	353.7813155
PIGT	2067.244149	1964.644214	1933.97983	1524.413089	1467.25932	1654.943408
RMND5B	553.7792775	596.9803049	506.8747909	754.5348384	764.5796611	755.1298666
PPIL2	1227.643309	1179.984482	1070.796376	934.1430116	823.0407746	828.4626883

CCDC86	884.2604593	816.6051662	805.2014668	629.0798832	615.5611365	617.3830799
SIKE1	918.0032826	1116.093614	1165.250903	1456.721566	1389.311168	1484.494147
SFR1	374.1483649	324.4458179	345.0863429	196.7566923	259.0629736	212.0705925
ABLIM3	257.0409191	281.5191404	280.5580024	405.2465818	396.6185349	401.3485511
ATP5PD	2235.958266	2004.576007	1970.45237	1384.517275	1777.905629	1317.017838
ZFC3H1	659.9699275	849.5488954	943.6100813	1161.586527	1080.957452	1331.882599
SNX25	193.5250163	251.5702957	194.520215	321.3090938	324.4018652	366.6641085
ERAP2	2685.531765	3155.609939	3173.111007	2313.244965	2235.27787	2416.01918
PLD2	852.5025079	806.622218	750.0250597	600.1981669	617.8537292	512.3387678
RPAP2	366.2088771	389.3349815	403.0683301	554.1679315	553.6611339	525.2215608
PSMD6	1501.55564	1454.515559	1412.141945	975.6604788	1205.903753	1098.010357
COBLL1	262.995535	321.4509334	332.9288295	428.7129763	442.4703886	514.3207359

BAX	1144.278686	1012.270952	874.405774	703.9918348	769.1648465	692.6978698
AES	3241.295915	2808.203341	2634.439642	2223.892155	2320.103799	2032.508342
DCAF1	817.7672485	921.4261228	860.3778739	1104.725649	1117.638935	1243.685017
GINM1	444.6113196	397.3213401	428.3185503	265.3507685	288.8666786	307.2050638
ECT2	2869.132422	3407.180235	3492.011936	4305.180836	4000.574239	4376.185684
GOLGA3	2060.297097	2061.478812	2172.454132	2602.964682	2603.238996	2631.062724
TMEM168	467.4373472	480.1798105	489.1061175	634.495205	665.9981756	669.905236
FAM86C1	173.6762967	162.7220564	188.9090549	95.67068525	96.28889286	105.0443122
EMC2	707.6068546	705.7944407	744.4138996	550.557717	463.1037228	540.0863219
VEPH1	40.68987523	49.91474121	55.17640713	87.54770254	105.4592636	128.82793
CDK5	178.6384766	161.7237615	139.3438078	74.00939802	104.3129673	73.33282169
MGST2	335.4433617	321.4509334	356.308663	234.6639449	201.7481565	225.9443695

MPLKIP	1184.968562	1079.156705	967.9251082	849.30297	782.9204026	654.0494908
TAPT1	170.6989888	189.6760166	182.3627015	266.2533222	308.3537164	281.4394778
SERPINE2	1382.463322	1431.554778	1481.346253	1754.564265	1976.214896	1800.618068
ZNF688	116.1150098	107.815841	105.6768476	60.47109351	55.02222449	37.65739492
DHX30	2199.238134	1965.642509	1919.95193	1605.642916	1540.622286	1584.583539
TYW5	199.4796322	200.6572597	204.8073417	305.0631284	290.0129749	338.9165543
SLC35A5	244.1392514	247.5771164	287.1043558	375.4623119	417.251869	377.5649333
GMDS	519.0440182	492.1593484	418.0314235	649.8386168	694.6555842	669.905236
ZBTB41	373.1559289	505.1371811	489.1061175	771.6833574	636.1944707	631.256857
IFI27L2	272.9198948	192.6709011	133.7326478	111.9166507	96.28889286	83.24266246
CMPK2	87.33436635	85.85335489	106.6120409	50.54300353	38.97407568	28.73853823
ABO	32.75038738	29.94884473	31.7965736	8.12298271	1.146296344	6.936888538

NR6A1	230.2451476	241.5873475	207.6129218	140.798367	124.9463014	142.7017071
OLFML2A	280.8593827	294.4969732	298.3266759	192.2439241	131.8240795	200.1787835
PPP1R35	804.8655808	685.8285443	662.1168856	515.3581252	541.0518741	463.780548
MGA	524.0061981	625.9308548	679.8855591	875.4770254	793.2370697	913.6873189
HPS4	1265.355876	1264.839542	1315.817031	946.7787625	1039.690784	978.1012839
ZBED5	972.5872616	1072.168641	1257.835044	1397.153026	1552.085249	1559.808937
DDX42	3504.29145	3442.120554	3576.179337	4286.22721	4418.972404	4196.817566
PHLDB3	506.1423504	448.2343761	447.9576104	328.5295229	318.6703835	324.0517932
BMP4	102.2209061	88.84823936	77.62104732	34.29703811	25.21851956	49.54920385
ANXA7	2201.223006	2158.31341	2106.990598	1646.257829	1827.196372	1539.989256
ACAA1	709.5917266	659.8728788	573.2735182	455.7895854	460.8111301	458.8256276
NUDT8	237.1921995	219.6248613	203.8721484	141.7009206	124.9463014	128.82793

ELMOD1	5.954615887	16.97101201	10.28712675	39.71235991	49.29074277	40.63034715
AC005726.1	94.28141822	88.84823936	95.38972081	35.19959174	46.99815008	43.60329938
SERF2	2024.569402	1748.014237	1447.679292	1187.760583	1381.287094	947.3807775
ALOX12	390.0273406	370.3673798	316.0953493	240.9818204	248.7463065	205.1337039
GLT8D1	814.7899406	822.5949352	766.8585398	561.3883606	628.1703962	588.6445417
HMGA2	12527.51939	13450.02617	13627.63737	11068.91777	10323.54487	10948.39208
LY6K	425.7550359	410.2991728	346.9567296	270.7660903	267.087048	246.7550352
PPP4C	2464.218541	2063.475402	1818.015855	1541.561608	1656.398216	1353.684249
NR3C2	9.924359812	11.97953789	4.675966706	33.39448447	36.68148299	43.60329938
ATF7IP2	124.0544977	107.815841	146.8253546	63.17875441	59.60740986	66.39593315
DTWD2	148.8653972	213.6350924	212.2888885	331.2371838	332.4259396	281.4394778
RIN1	1561.101798	1475.47975	1258.770237	1100.21288	1102.737082	946.3897935

PNPLA2	1438.039737	1205.940148	1179.278803	1621.888881	1783.637111	1683.681947
PBDC1	231.2375836	227.6112199	206.6777284	323.1142011	366.8148299	325.0427772
GRIN2D	813.7975046	822.5949352	764.0529598	593.8802914	622.4389145	559.9060035
ZNF263	650.0455677	660.8711737	655.5705322	844.7902018	887.2333699	878.0118921
FAM219A	303.6854103	343.4134195	290.8451291	217.5154259	184.5537113	197.2058313
ZMYM1	466.4449112	525.1030776	573.2735182	345.678042	353.0592738	381.5288696
PHF11	330.4811817	347.4065988	345.0863429	425.1027618	541.0518741	551.9781308
C11orf54	68.4780827	87.84994453	96.32491415	170.5826369	158.1888954	143.6926912
ARFGEF1	1005.337649	1344.703128	1477.605479	1903.485615	1644.935253	1948.274695
FNDC3B	1968.992987	2508.714893	2414.669207	3122.835575	2827.913079	3268.265486
GCOM1	20.84115561	22.96078096	36.47254031	65.88641531	59.60740986	96.12545546
USP24	1583.927826	1889.772102	1867.581102	2480.217387	2097.722309	2628.089772

TMEM134	649.0531317	609.9581376	566.7271648	483.768748	401.2037202	347.835411
POLDIP3	1369.561654	1349.694602	1350.419185	1683.262528	1822.611186	1676.745058
SOX2	260.0182271	277.5259611	382.4940766	207.5873359	162.7740808	171.4402453
PSRC1	799.9034009	708.7893252	630.320312	544.2398415	466.5426118	485.5821977
IL17C	10.91679579	12.97783272	8.416740071	44.22512809	36.68148299	40.63034715
MDGA1	89.31923831	91.84312383	70.13950059	27.97916267	42.41296471	34.68444269
DPY30	683.7883911	570.0263447	614.4220252	431.4206372	464.2500191	432.0690575
TAB3	777.0773733	957.3647365	998.7864884	1264.477642	1172.661159	1312.062918
FAM181B	149.8578332	186.6811321	175.8163482	97.47579252	106.6055599	72.34183761
SLC25A39	4882.785028	4099.996843	3811.848059	3287.100337	3235.994578	3246.463836
LAMC1	5272.812368	5492.618123	5826.254516	6693.337753	6726.466944	6959.681172
PPOX	347.3525934	301.4850369	279.622809	222.0281941	176.5296369	179.3681179

NPM3	1263.371 004	997.2965 294	1042.740 575	818.6161 464	805.8463 295	785.8503 73
TIMM21	295.7459 224	257.5600 647	288.9747 424	166.0698 687	194.8703 784	172.4312 294
C1orf115	233.2224 556	228.6095 148	218.8352 418	339.3601 665	357.6444 592	326.0337 613
SYNM	225.2829 677	212.6367 976	205.7425 351	136.2855 988	91.70370 748	137.7467 867
PFN1	12254.59 95	9782.290 983	8940.448 342	7375.668 3	7974.783 662	7719.765 959
GPANK1	552.7868 415	414.2923 521	412.4202 635	295.1350 385	326.6944 579	273.5116 052
MB21D2	186.5779 645	200.6572 597	207.6129 218	328.5295 229	319.8166 798	276.4845 575
GATA6	200.4720 682	186.6811 321	160.8532 547	298.7452 53	306.0611 237	264.5927 485
CFL2	383.0802 887	404.3094 038	439.5408 704	549.6551 634	593.7815 059	579.7256 85
CD99	998.3905 971	881.4943 298	784.6272 133	644.4232 95	680.9000 28	560.8969 875
EIF2AK2	2001.743 374	2287.093 442	2635.374 836	3115.615 146	3037.685 31	3021.510 451
ZNF227	113.1377 019	129.7783 272	119.7047 477	185.0234 951	198.3092 674	238.8271 625

RFC1	1830.051949	1929.703895	1999.443364	2282.558141	2537.900105	2492.324953
POLR2M	391.0197766	373.3622643	456.3743505	548.7526097	662.5592866	562.8789557
CCDC189	37.71256729	38.93349815	36.47254031	11.73319725	9.170370748	6.936888538
CDK20	227.2678397	181.689658	178.6219282	326.7244157	300.329642	296.304239
LIG3	564.6960733	591.9888308	608.8108651	795.1497519	743.9463269	855.2192584
ZC3H13	1297.113827	1351.691192	1573.930393	1778.933213	1827.196372	1962.148472
KIAA0100	3875.462507	4088.017305	4103.628381	3246.485423	2898.983453	3395.111447
TRABD	2263.746473	2021.547019	1800.247182	1506.362016	1594.498214	1508.277765
COX14	463.4676032	353.3963678	329.1880561	249.1048031	261.3555663	199.1877995
CCNY	1173.05933	1220.91457	1134.389523	1474.772639	1528.013026	1500.349892
OSBPL2	768.1454495	847.5523058	766.8585398	1045.157109	1076.372267	1017.740647
SLC35D2	149.8578332	107.815841	130.9270678	200.3669068	234.9907504	227.9263377

PRKCZ	384.0727247	323.4475231	313.2897693	481.0610871	541.0518741	470.7174365
DOCK5	1853.870413	2249.158239	2301.510813	2789.793284	2668.577888	2968.988294
FAM207A	523.0137621	466.2036829	453.5687705	369.1444365	314.0851981	235.8542103
BEND7	29.77307944	19.96589649	21.50944685	61.37364714	60.75370621	69.36888538
EID1	2206.185186	2120.378207	2096.703471	1744.636175	1746.955628	1564.763857
C22orf23	105.198214	107.815841	99.13049417	199.4643532	205.1870455	150.6295797
NECAB3	457.5129873	424.2753003	376.8829165	296.0375921	270.5259371	284.4124301
LRP4	811.8126326	839.5659472	804.2662735	612.8339178	395.4722385	614.4101277
STARD5	130.0091135	136.7663909	112.2232009	55.95832533	43.55926105	80.26971023
CAPNS1	4291.293183	3728.631169	3388.205475	2578.595734	2943.68901	3055.203909
ARPC5	3602.542612	3510.004602	3403.168569	2763.619229	2948.274196	2272.326488
LDHB	1436.054865	1233.892403	977.2770416	766.2680356	964.0352249	707.5626309

GNGT1	115.1225738	150.7425185	113.1583943	227.4435159	196.0166747	222.9714173
ERG28	535.9154299	451.2292606	483.4949574	295.1350385	314.0851981	380.5378855
CDKL5	106.19065	165.7169408	170.2051881	274.3763049	233.8444541	257.65586
ALS2	408.8836243	342.4151247	420.8370036	524.3836616	581.1722462	574.7707646
MZT2B	635.159028	510.1286552	394.65159	317.6988793	363.3759409	215.0435447
SECISBP2L	251.0863032	361.3827264	354.4382763	492.7942844	505.5166875	473.6903888
GULP1	221.3132238	266.5447181	281.4931957	399.83126	379.4240897	376.5739492
EIF3M	3786.143268	3691.69426	3621.068617	4375.58002	4561.113151	4637.80548
RHPN2	975.5645695	905.4534056	907.137541	687.7458694	740.5074379	669.905236
MAFK	1805.24105	1822.886349	1418.688299	2513.611872	2407.222321	2028.544405
RPL26L1	714.5539065	570.0263447	535.8657845	389.9031701	445.9092776	406.3034715
DBP	161.7670649	149.7442236	173.9459615	88.45025617	84.82592942	98.10742361

NUDT11	238.1846<br>355	208.6436<br>183	208.5481<br>151	136.2855<br>988	130.6777<br>832	130.8098<br>982
NPLOC4	4482.833<br>327	4349.570<br>549	4300.954<br>176	3656.244<br>773	3568.420<br>517	3554.659<br>884
THNSL2	7.939487<br>85	10.98124<br>307	14.02790<br>012	43.32257<br>445	41.26666<br>837	37.65739<br>492
PLA2G4A	611.3405<br>644	594.9837<br>153	561.1160<br>047	405.2465<br>818	381.7166<br>824	463.7805<br>48
NDUFA12	882.2755<br>873	748.7211<br>182	666.7928<br>523	571.3164<br>506	506.6629<br>838	535.1314<br>015
TNF	16.87141<br>168	18.96760<br>166	25.25022<br>021	55.05577<br>17	63.04629<br>889	55.49510<br>831
FGFRL1	975.5645<br>695	834.5744<br>731	808.0070<br>468	647.1309<br>559	566.2703<br>937	670.8962<br>201
ZFP1	238.1846<br>355	251.5702<br>957	252.5022<br>021	360.1189<br>001	375.9852<br>007	357.7452<br>518
STARD3	679.8186<br>471	661.8694<br>685	580.7550<br>649	862.8412<br>745	857.4296<br>65	866.1200<br>832
NF2	1043.050<br>216	942.3903<br>141	903.3967<br>676	729.2633<br>366	723.3129<br>928	716.4814<br>876
PDE8B	80.38731<br>448	81.86017<br>559	94.45452<br>746	145.3111<br>351	170.7981<br>552	150.6295<br>797
SCART1	123.0620<br>617	172.7050<br>046	140.2790<br>012	77.61961<br>256	72.21666<br>964	79.27872<br>615

KCTD20	938.8444382	1061.187398	1013.749582	1267.185303	1279.266719	1394.314596
UQCC2	518.0515822	488.1661691	408.6794901	299.6478066	362.2296446	266.5747167
GBP1	430.7172158	489.1644639	463.8558972	676.9152258	598.3666913	642.1576818
ATAD1	911.0562308	929.4124814	997.8512951	1294.261912	1250.609311	1163.415306
VPS29	2460.248797	2239.175291	2084.545958	1748.24639	1820.318594	1717.375405
SLC27A1	287.8064346	304.4799214	270.2708756	186.8286023	183.407415	188.2869746
CLPP	829.6764803	815.6068714	671.468819	567.706236	570.8555791	522.2486085
PNKD	1094.656887	836.5710627	837.9332337	659.7667068	683.1926207	610.4461914
RNF114	2373.906867	2346.991132	2340.788933	2949.545277	2973.492715	2796.557065
XPNPEP3	338.4206696	341.4168299	337.6047962	222.0281941	213.2111199	243.7820829
CAAP1	628.2119761	673.8490064	713.5525194	890.8204372	931.9389273	869.0930355
CENPU	656.0001836	635.9138031	696.7190392	426.0053154	471.1277972	525.2215608

ATP11B	1021.216625	1317.749168	1250.353497	1653.478258	1549.792656	1614.313061
PIEZO1	4751.783478	4728.922583	4650.716486	5979.417828	5436.883557	5900.319194
FAM81A	280.8593827	232.6026941	249.6966221	147.1162424	146.725932	176.3951657
DPP3	1409.259093	1249.86512	1213.880957	947.6813161	1040.83708	945.3988094
IL1RN	84.3570584	91.84312383	104.7416542	37.90725265	49.29074277	41.62133123
IL33	45.65205514	98.8311876	54.24121379	16.24596542	22.92592687	25.765586
PPP2R1B	760.2059616	714.7790942	870.6650007	591.1726305	515.8333546	579.725685
PCED1B	203.4493761	235.5975785	152.4365146	77.61961256	120.3611161	114.9541529
RHOD	1369.561654	1135.061215	1016.555162	1628.206756	1688.494514	1505.304813
PSMD12	2299.474168	2400.899052	2532.503568	1794.276625	2000.287119	1920.527141
THYN1	326.5114378	325.4441127	347.8919229	201.2694605	212.0648236	244.773067
AGFG1	1471.78256	1638.201807	1642.199507	1972.982245	1961.313044	2144.489542

TUT4	542.8624817	614.9496117	681.7559458	845.6927554	821.8944783	853.2372902
TPM3	12391.55566	11573.2319	11740.41721	9505.694878	10133.25968	9824.616139
MESD	787.9941691	872.5096764	846.3499738	579.4394333	676.3148427	592.608478
WNK1	2167.480183	2672.435245	2754.14439	3575.014946	2974.639011	3817.270664
SESN1	181.6157846	171.7067098	188.9090549	101.0860071	116.922227	97.11643954
AK7	41.68231121	35.93861367	51.43563377	13.53830452	8.024074405	13.87377708
COL6A1	613.3254364	631.9206238	399.3275567	354.7035783	351.9129775	336.9345861
DUOX1	177.6460406	184.6845425	169.2699948	99.28089978	103.1666709	107.0262803
BABAM1	430.7172158	338.4219454	280.5580024	220.2230868	226.966676	199.1877995
ARL15	219.3283518	242.5856423	254.3725888	330.3346302	384.0092751	362.7001721
RNF115	647.0682598	650.8882254	640.6074387	826.7391291	981.2296701	824.498752
EEF1AKNMT	974.5721336	875.5045609	803.3310801	646.2284022	669.4370646	642.1576818

NDUFAF1	272.9198948	248.5754112	260.9189422	158.8494397	182.2611186	154.593516
DPYD	281.8518187	299.4884473	304.8730292	453.0819245	417.251869	408.2854397
ZFAT	164.7443729	175.6998891	164.5940281	245.4945886	253.3314919	306.2140798
TAF9	1718.899119	1685.121663	1614.143707	1994.643532	2226.107499	2126.651829
LAMTOR1	1613.700905	1327.732116	1318.622611	1024.398375	1154.320418	924.5881438
ERCC1	2331.23212	2146.333872	2027.499164	2918.858454	3124.803832	2508.180699
PRELID3B	2419.558922	2529.679085	2531.568375	2942.324848	3342.600138	3089.888352
OSTF1	952.738542	812.6119869	810.8126268	601.1007205	660.2666939	633.2388251
UFSP1	74.43269859	53.90792051	57.04679381	22.56384086	14.90185247	27.74755415
CTXN1	276.8896388	245.5805268	210.4185018	169.6800833	134.1166722	131.8008822
APEX2	812.8050686	742.7313492	729.4508062	572.2190042	550.2222449	573.7797805
LETM2	102.2209061	99.82948243	120.639941	203.9771214	163.9203771	191.2599268

NAA16	482.3238869	510.1286552	617.2276052	741.8990875	710.703733	815.5798953
11-Sep	877.3134074	1047.211271	1022.166322	728.360783	709.5574366	755.1298666
IMPACT	367.201313	386.340097	348.8271163	251.812464	267.087048	219.9984651
ARL13B	248.1089953	262.5515388	251.5670088	364.6316683	348.4740884	417.2042964
ZNRF1	537.9003018	540.0774999	479.754184	732.8735511	781.7741063	647.1126022
CCT3	10734.18757	9708.417166	9559.546334	7817.919581	8205.189227	8343.094944
NPAS2	522.0213261	564.0365757	544.2825246	687.7458694	750.824105	769.0036437
ISOC2	1920.363624	1662.160882	1563.643267	1267.185303	1275.82783	1365.576058
C19orf53	1131.377019	944.3869037	814.5534002	668.7922431	717.581511	659.0044111
VPS72	964.6477737	798.6358594	749.0898663	1116.458846	1290.729683	1078.190676
FSTL1	3.969743925	4.991474121	1.870386682	22.56384086	24.07222321	26.75657008
SUV39H1	366.2088771	362.3810212	325.4472827	244.5920349	249.8926029	197.2058313

MOV10L1	2.977307944	0.998294824	0.935193341	15.34341178	19.48703784	25.765586
GGACT	70.46295467	68.88234287	77.62104732	144.4085815	124.9463014	134.7738345
HOMER1	626.2271041	712.7825045	762.1825731	1021.690714	853.9907759	1018.731631
LPAR5	62.52346682	73.87381699	69.20430725	21.66128723	33.24259396	26.75657008
OSBPL9	1764.551175	1572.314348	1745.070775	1321.338521	1327.411166	1337.828504
PXN	9539.294651	9129.406168	8671.11266	11226.86466	11164.92639	10784.87971
6-Sep	685.773263	726.7586321	628.4499253	485.5738553	504.3703911	503.4199111
GPS1	2630.947786	2221.205984	2152.815072	1790.666411	1681.616736	1875.932858
MED25	792.956349	794.6426801	818.2941736	602.9058278	623.5852109	588.6445417
WDR4	589.5069728	590.990536	636.8666654	469.3278899	426.4222398	419.1862645
PPP1R26	786.0092971	801.6307439	764.9881531	591.1726305	583.4648389	597.5633984
ANKRD39	88.32680233	90.84482901	68.26911391	34.29703811	34.38889031	38.648379

P2RX6	30.76551542	18.96760166	25.25022021	3.610214538	2.292592687	4.954920385
HS3ST3A1	96.26629018	78.86529112	99.13049417	46.93278899	28.65740859	44.59428346
ZFYVE16	654.0153116	800.6324491	1001.592068	1243.718908	1039.690784	1362.603106
MRPS18B	1921.35606	1744.021058	1716.079781	1321.338521	1513.111173	1309.089966
MND1	229.2527117	190.6743114	247.8262354	139.8958133	107.7518563	143.6926912
ASB8	227.2678397	199.6589649	256.2429755	344.7754883	366.8148299	328.0157295
MBP	462.4751672	481.1781053	466.6614773	629.9824368	643.0722487	636.2117774
TMEM255B	20.84115561	12.97783272	17.76867348	48.73789626	56.16852083	49.54920385
DCAKD	291.7761785	275.5293715	294.5859025	162.4596542	176.5296369	211.0796084
MLLT3	365.2164411	391.3315711	349.7623096	521.6760007	475.7129826	576.7527328
ZNF721	223.2980958	253.5668854	286.1691624	410.6619037	342.7426067	400.3575671
IL17RE	215.3586079	206.6470286	173.9459615	106.5013289	132.9703758	109.9992325

KDM4B	698.6749<br>308	764.6938<br>354	707.0061<br>66	972.0502<br>643	957.1574<br>468	921.6151<br>915
ATL3	1980.902<br>219	2130.361<br>155	2204.250<br>705	1701.313<br>601	1712.566<br>737	1567.736<br>81
RC3H2	1495.601<br>024	1745.019<br>353	1876.933<br>036	2301.511<br>768	2058.748<br>233	2443.766<br>734
ERBB3	1639.504<br>241	1762.988<br>66	1816.145<br>469	1398.958<br>133	1125.663<br>009	1399.269<br>517
KIAA0232	408.8836<br>243	511.1269<br>5	526.5138<br>511	716.6275<br>857	653.3889<br>158	669.9052<br>36
CSTF2	1289.174<br>34	1344.703<br>128	1234.455<br>21	1018.983<br>053	990.4000<br>408	1007.830<br>806
RPL35	17025.23<br>926	14907.53<br>661	12938.39<br>988	10423.59<br>192	12674.59<br>867	10436.05<br>331
OPLAH	379.1105<br>448	352.3980<br>73	309.5489<br>959	522.5785<br>543	534.1740<br>961	450.8977<br>55
ELK4	1108.550<br>991	1251.861<br>71	1363.511<br>892	1608.350<br>577	1611.692<br>659	1640.078<br>647
MARCKS	2139.691<br>975	2189.260<br>55	2404.382<br>08	2928.786<br>544	3385.013<br>102	2603.315<br>17
CD44	6712.836<br>977	6830.333<br>188	6653.900<br>623	5513.700<br>153	5362.374<br>295	5797.256<br>85
PINK1	209.4039<br>92	200.6572<br>597	187.9738<br>616	135.3830<br>452	114.6296<br>344	102.0713<br>599

ZNF16	136.9561654	130.776622	129.0566811	202.1720141	236.1370468	210.0886243
TUBG1	1631.564753	1313.755989	1234.45521	1027.106036	997.2778189	1045.488201
FBXW4	242.1543794	187.679427	207.6129218	123.6498479	127.2388941	131.8008822
ZNF202	274.9047668	285.5123197	287.1043558	411.5644573	373.692608	446.9338187
MARF1	887.2377672	1000.291414	948.286048	1218.447406	1182.977827	1280.351427
SYNJ1	163.7519369	213.6350924	259.9837489	367.3393292	295.7444566	382.5198537
CNTNAP3C	107.183086	33.94202402	57.04679381	24.36894813	19.48703784	14.86476115
C12orf75	1346.735627	1229.899223	1229.779244	969.3426034	994.9852262	993.9570291
CRHR2	10.91679579	8.984653418	9.351933412	48.73789626	29.80370493	34.68444269
CLCN5	222.3056598	229.6078096	270.2708756	380.8776337	315.2314945	410.2674078
CT45A1	52.599107	34.94031885	29.92618692	81.2298271	96.28889286	91.17053508
RGS19	593.4767168	568.029755	490.0413108	383.5852946	397.7648312	390.4477263

COPG1	2361.005199	2292.084916	2153.750265	2706.75835	2884.0816	2880.790712
ZFYVE9	393.0046486	457.2190295	444.2168371	615.5415787	570.8555791	605.491271
STRIP1	733.4101901	727.7569269	676.1447857	939.5583334	945.6944834	914.678303
ZBTB26	326.5114378	304.4799214	370.3365631	489.1840698	469.9815008	471.7084206
BCAP31	3264.121942	2777.256201	2666.236216	2132.734238	2437.026026	2014.670628
ALDH1L1	17.86384766	22.96078096	28.99099358	4.512768172	1.146296344	2.972952231
RNF223	81.37975046	73.87381699	46.75966706	132.6753843	184.5537113	109.0082485
TMEM186	232.2300196	255.563475	217.9000485	146.2136888	155.8963027	137.7467867
PSMG1	868.3814836	808.6188076	885.6280941	594.7828451	668.2907683	658.0134271
ICE1	1936.242599	2383.92804	2441.789814	3030.775104	2738.501965	3163.221174
FARSB	1644.466421	1656.171113	1734.783648	1354.733005	1337.727833	1316.026854
ANAPC13	588.5145369	559.0451016	600.3941251	757.2424993	792.0907734	774.9495481

BBS2	556.7565855	628.9257393	580.7550649	420.5899936	437.8852032	425.132169
MAGEA6	1350.70537	1186.972546	1197.047477	968.4400497	969.7667066	933.5070005
WRNIP1	2129.767616	2014.558955	1949.878116	1655.283366	1658.690809	1453.773641
KIF4A	812.8050686	853.5420747	873.4705807	675.1101185	601.8055803	631.256857
TXNDC11	714.5539065	668.8575323	607.8756718	443.1538345	531.8815034	447.9248028
WHAMM	284.8291266	318.4560489	270.2708756	408.8567964	429.8611288	422.1592168
SBF1	2563.462139	2623.518798	2345.4649	2074.970806	1971.629711	1956.202568
RAB11A	1962.045935	1925.710716	2052.749384	1492.823711	1635.764882	1595.484364
RALGPS1	57.56128691	66.88575323	73.88027396	121.8447406	130.6777832	124.8639937
KLF9	286.8139986	310.4696903	282.4283891	442.2512809	471.1277972	376.5739492
S100A9	106.19065	78.86529112	65.46353389	27.97916267	44.7055574	30.72050638
CYHR1	761.1983976	765.6921302	712.617326	981.9783542	974.351892	960.2635705

HAT1	1309.023059	1308.764515	1338.261671	906.1638489	1038.544487	1084.13658
SLC2A1	6387.317975	5906.910475	4700.281733	7282.705276	8292.307749	7178.688653
SERPINB2	292.7686145	312.46628	288.0395491	190.4388169	218.9426016	168.4672931
CFL1	20614.8802	18148.00161	16718.45136	15026.61546	15062.33395	13250.44809
HSPA14	462.4751672	413.2940572	514.3563377	667.8896895	609.8296548	659.9953952
GPN1	631.1892841	596.9803049	603.1997051	750.9246238	863.1611467	843.3274495
USP43	163.7519369	163.7203512	161.788448	240.0792668	270.5259371	253.6919237
ANKRD16	119.0923177	124.786853	98.19530083	65.88641531	44.7055574	56.48609238
EXPH5	355.2920813	383.3452125	459.1799305	256.3252322	256.7703809	286.3943982
CNPPD1	1001.367905	890.4789832	756.5714131	647.1309559	671.7296573	560.8969875
SLC27A2	21.83359159	18.96760166	28.05580024	66.78896895	51.58333546	65.40494908
SPAST	355.2920813	372.3639694	405.8739101	580.3419869	495.2000204	524.2305767

OR51B4	28.78064346	25.95566543	19.63906017	5.415321806	1.146296344	3.963936308
ZNF134	391.0197766	418.2855314	405.8739101	581.2445406	539.9055778	550.9871468
DHRS4	149.8578332	113.80561	118.7695543	57.7634326	82.53333673	48.55821977
ERAP1	990.4511093	1083.149884	1205.464217	1447.69603	1456.942653	1385.39574
ARHGEF35	91.30411027	75.87040664	81.36182069	146.2136888	157.0425991	142.7017071
AK6	238.1846355	222.6197458	246.8910421	344.7754883	339.3037177	357.7452518
NEK7	1055.951884	1256.853184	1349.483991	1663.406348	1587.620436	1595.484364
LSM14A	2319.322888	2423.859833	2571.781688	2968.498904	3184.411242	2961.060422
HIC2	423.770164	394.3264556	411.4850701	300.5503603	279.6963078	275.4935734
KIF3A	441.6340116	506.1354759	550.828878	720.2378003	670.583361	680.8060608
PIGH	418.8079841	412.2957624	432.0593236	543.3372879	629.3166926	575.7617487
DPYSL2	259.0257911	240.5890526	252.5022021	361.0214538	377.131497	357.7452518

SSH2	506.1423504	541.0757947	623.7739586	796.0523055	696.9481769	827.4717042
KLHDC3	1540.260643	1263.841248	1170.862063	1030.71625	938.8167053	946.3897935
PLBD2	1572.018594	1587.288771	1509.402053	1249.13423	1062.61671	1273.414539
NRTN	97.25872616	82.85847041	80.42662735	40.61491355	40.12037202	38.648379
IMPA1	446.5961915	518.1150138	579.8198716	733.7761048	737.0685489	682.788029
NCL	15876.99083	15120.17341	15954.3984	12697.12453	13552.66167	12803.51427
STIP1	8576.63175	7535.129333	7597.510704	5890.065018	6568.278048	6508.783417
MTURN	519.0440182	547.0655637	535.8657845	400.7338137	384.0092751	375.5829651
RAB9A	326.5114378	349.4031885	376.8829165	496.4044989	539.9055778	461.7985798
ZNF586	239.1770715	233.6009889	230.9927553	374.5597583	369.1074226	308.1960479
MAMDC4	99.24359812	88.84823936	75.75066064	170.5826369	132.9703758	174.4131975
NGEF	324.5265659	321.4509334	282.4283891	209.3924432	209.7722309	194.2328791

SULF2	503.1650425	521.1098983	471.337444	341.1652738	342.7426067	379.5469015
COX18	285.8215626	298.4901525	254.3725888	193.1464778	176.5296369	171.4402453
AD000864.1	199.4796322	171.7067098	222.5760152	112.8192043	134.1166722	98.10742361
HIST1H2BH	41.68231121	48.91644639	62.65795386	93.86557798	108.8981526	115.945137
TDG	610.3481284	684.8302494	687.3671058	922.4098144	837.9426271	877.0209081
SLC38A5	1.984871962	2.994884473	0	19.85617996	26.3648159	13.87377708
SIK1B	526.983506	484.1729898	420.8370036	333.9448447	341.5963104	319.0968728
ZNF234	50.61423504	77.86699629	70.13950059	126.3575088	146.725932	115.945137
SAE1	2928.678581	2611.53926	2555.883402	2202.230868	2149.305644	2079.084593
MID1IP1	1037.0956	977.3306329	956.7027881	1272.600625	1466.113023	1194.135813
PCF11	725.4707023	709.78762	813.6182069	989.1987833	943.4018907	1044.497217
PYCR2	1356.659986	1098.124307	1044.610962	889.0153299	867.746332	803.6880864

UQCRQ	3031.891923	2687.409667	2370.71512	2028.94057	2144.720459	2035.481294
TFAM	979.5343135	1120.086793	1158.70455	1434.157725	1459.235245	1350.711297
PEX16	862.4268677	727.7569269	717.2932927	572.2190042	594.9278023	484.5912136
GABRE	275.8972028	277.5259611	292.7155158	181.4132805	197.1629711	177.3861498
AGPS	1542.245515	1702.092675	1835.784529	2189.595117	2182.548238	2103.859195
ARHGEF5	407.8911883	480.1798105	388.1052366	537.0194125	649.9500268	636.2117774
ARL3	387.0500327	401.3145194	376.8829165	284.3043948	272.8185298	244.773067
STX4	776.0849373	655.8796995	529.3194311	473.8406581	456.2259447	381.5288696
APRT	2438.415206	2089.431067	1953.61889	1673.334438	1742.370442	1461.701513
BCAS4	522.0213261	597.9785997	517.1619177	416.0772255	354.2055701	387.4747741
LRRCC1	370.178621	391.3315711	427.3833569	271.668644	247.6000102	292.3403027
PSMC4	2006.705554	1816.89658	1625.366027	1361.050881	1409.944503	1421.071166

GCDH	488.2785028	417.2872365	380.6236899	318.6014329	262.5018627	280.4484938
NAT10	2683.546893	2634.500041	2594.226329	3193.234759	3160.339019	3297.004024
ARHGAP11A	1118.475351	1337.715065	1268.122171	968.4400497	831.064849	981.0742361
ENO1	71458.36796	63646.28652	59938.41163	50710.8785	55027.95597	50878.11349
EIF2AK1	2955.474352	2816.189699	2943.988638	2278.045373	2247.88713	2491.333969
RAB36	110.1603939	93.83971348	99.13049417	44.22512809	57.31481718	46.57625161
NFKBIE	196.5023243	189.6760166	164.5940281	292.4273775	306.0611237	256.6648759
EXT2	1427.122941	1449.524085	1360.706311	1080.3567	1056.885229	1174.316131
ALKBH6	46.64449112	37.93520332	35.53734697	10.83064361	14.90185247	7.927872615
CCDC34	598.4388967	569.0280498	589.171805	429.61553	401.2037202	450.897755
MMP11	60.53859485	40.93008779	28.99099358	11.73319725	10.31666709	13.87377708
PCNX4	673.8640312	779.6682577	788.3679867	987.393676	961.7426322	1016.749663

ARNT	874.3360994	976.3323381	989.434555	1204.006548	1178.392641	1335.846536
CERS6	494.2331186	759.7023613	742.5435129	1034.326465	923.9148529	975.1283317
SMYD3	259.0257911	220.6231562	222.5760152	333.0422911	354.2055701	353.7813155
TRPM2	563.7036373	501.1440018	462.9207039	368.2418828	359.9370519	350.8083632
REEP3	608.3632565	622.9359703	775.2752799	508.1376962	466.5426118	439.0059461
ZNF44	27.78820747	18.96760166	12.15751344	1.805107269	2.292592687	0
RRN3	990.4511093	1095.129422	1083.889082	1378.1994	1404.213021	1290.261268
LARP1B	455.5281154	496.1525277	432.994517	595.6853987	668.2907683	636.2117774
RBFOX2	1227.643309	1318.747463	1326.104158	1662.503795	1584.181547	1634.132743
RBMS1	719.5160864	834.5744731	909.0079277	1126.386936	1032.813006	1155.487434
CDC37	5654.900221	4952.540623	4581.512179	3909.862344	4141.568689	3892.585454
ZNF589	577.5977411	511.12695	518.097111	405.2465818	374.8389043	372.6100129

TSG101	1333.833959	1135.061215	1188.630737	1456.721566	1801.977852	1603.412236
FADS2	355.2920813	325.4441127	326.3824761	219.3205332	251.0388992	202.1607517
TECR	1785.39233	1548.355272	1449.549679	1167.904403	1251.755607	1233.775176
GNB3	33.74282336	38.93349815	51.43563377	16.24596542	4.585185374	10.90082485
PTBP2	323.5341299	293.4986783	357.2438563	447.6666027	463.1037228	479.6362932
PCBP2	5000.884909	4389.502342	4466.483398	5658.108734	6279.41137	5484.105882
FAM76B	392.0122126	439.2497227	489.1061175	599.2956132	623.5852109	611.4371755
MRPS25	845.555456	891.4772781	889.3688675	714.8224785	649.9500268	607.4732391
GAREM1	56.56885093	79.86358594	97.26010749	132.6753843	151.3111173	162.5213886
LRRC38	44.65961915	55.90451016	49.56524708	16.24596542	21.77963053	2.972952231
NR4A2	84.3570584	84.85506006	97.26010749	160.6545469	137.5555612	173.4222135
ABHD4	277.8820747	212.6367976	215.0944685	314.0886648	411.5203873	371.6190288

PSMB7	4204.951252	3572.897176	3381.659122	2782.572855	3112.194573	2666.738151
SNRPE	2029.531582	1848.842015	1605.726967	1399.860687	1439.748207	1346.747361
SMARCD1	1666.300012	1646.188165	1626.30122	2026.232909	2133.257495	1978.995202
SMAD3	3395.123492	3528.972204	3446.187462	2745.568156	2751.111224	2966.015342
METTL1	577.5977411	456.2207347	494.7172775	370.9495437	358.7907555	337.9255702
GPAT3	153.8275771	186.6811321	179.5571215	256.3252322	277.4037151	272.5206212
C5orf30	327.5038738	352.398073	401.1979434	228.3460695	255.6240846	244.773067
PQLC1	412.8533682	340.4185351	317.965736	235.5664986	244.1611212	231.890274
TXNL1	408.8836243	417.2872365	418.0314235	519.8708934	668.2907683	568.8248601
ANAPC15	179.6309126	178.6947735	176.7515415	103.793668	122.6537088	82.25167838
VAMP1	113.1377019	79.86358594	142.1493879	51.44555716	63.04629889	38.648379
ERLIN1	1044.042652	1051.20445	1063.314829	830.3493437	786.3592917	837.381545

UBE2E2	514.0818383	497.1508225	448.8928038	609.2237032	755.4092904	666.9322838
PNPLA6	1295.128955	1221.912865	1178.34361	1000.931981	880.3555918	966.209475
HEG1	290.7837425	300.4867421	288.9747424	194.951585	163.9203771	213.0615765
RABEPK	818.7596845	719.7705683	621.9035719	528.8964298	491.7611314	524.2305767
STIM2	691.7278789	631.9206238	617.2276052	444.9589418	455.0796484	514.3207359
PPM1H	83.36462242	91.84312383	105.6768476	174.1928514	142.1407466	178.3771338
TRPS1	275.8972028	377.3554436	414.2906502	519.8708934	482.5907606	642.1576818
METTL6	195.5098883	197.6623752	202.0017617	298.745253	290.0129749	300.2681753
COL21A1	0.992435981	1.996589649	0.935193341	15.34341178	25.21851956	14.86476115
ZNF222	17.86384766	31.94543438	27.1206069	61.37364714	61.90000255	73.33282169
TMEM260	166.7292448	173.7032994	191.714635	308.673343	253.3314919	264.5927485
RAG1	188.5628364	208.6436183	214.1592751	334.8473984	278.5500115	312.1599842

NUCB1	1842.953617	1791.93921	1702.051881	1321.338521	1508.525988	1368.54901
TNS3	1382.463322	1448.52579	1483.216639	1859.260487	1681.616736	1956.202568
SNX16	165.7368089	204.650439	241.279882	310.4784502	318.6703835	324.0517932
ARL16	552.7868415	497.1508225	495.6524708	367.3393292	389.7407568	352.7903314
LARP4	1102.596375	1232.894108	1316.752224	1630.011864	1464.966727	1667.826201
ABCC5	1196.877793	1194.958905	1212.01057	974.7579252	827.62596	962.2455387
PRCP	1193.900485	1166.008355	1105.398529	838.4723264	942.2555944	899.8135418
SUB1	4462.984608	4130.943983	4517.919031	3212.188385	3788.509415	3449.615572
TIPARP	5111.045303	5903.915591	5134.211443	6725.829684	6478.866934	6815.988481
ABT1	994.4208532	908.4482901	832.3220737	663.3769213	637.340767	731.3462488
MAGEE1	20.84115561	25.95566543	32.73176694	2.707660903	5.731481718	5.945904461
TNPO3	1319.939855	1250.863415	1313.946644	1627.304203	1604.814881	1626.20487

SLFN13	389.0349046	447.2360813	421.7721969	308.673343	269.3796407	295.3132549
ZNF771	124.0544977	108.8141358	116.8991677	54.15321806	66.48518792	62.43199685
CLASP2	666.9169794	837.5693576	914.6190877	1182.345261	998.4241152	1203.054669
SOX7	604.3935126	681.835365	612.5516385	789.7344301	992.6926335	824.498752
NXT2	422.777728	527.0996672	539.6065579	329.4320766	337.011125	365.6731244
MIB2	2294.511989	1981.615226	1868.516296	1490.11605	1632.325993	1612.331093
TMCO3	408.8836243	457.2190295	481.6245707	597.490506	674.02225	593.5994621
CRB3	309.6400261	312.46628	256.2429755	481.0610871	510.1018729	354.7722995
NANS	1175.044202	1090.137948	983.823395	795.1497519	832.2111454	842.3364654
SMUG1	1111.528299	957.3647365	887.4984808	745.509302	731.3370672	726.3913284
IKZF5	287.8064346	345.4100092	366.5957898	495.5019453	542.1981705	429.0961053
ZNF274	466.4449112	469.1985674	442.3464504	600.1981669	660.2666939	607.4732391

SERP1	6557.024528	6493.907832	6462.185988	8167.207838	8486.031831	7319.408392
EYA3	382.0878528	454.224145	439.5408704	566.8036824	554.8074303	690.7159016
UBE2D2	1688.133604	1644.191576	1615.0789	2034.355892	2281.129724	1955.211584
SNRPA	1837.991437	1619.234205	1595.43984	1333.974272	1370.970427	1195.126797
PPAT	1114.505607	1335.718475	1393.438078	1002.737088	895.2574443	981.0742361
EPS8L1	441.6340116	423.2770055	369.4013698	577.634326	629.3166926	527.2035289
TBKBP1	137.9486014	121.7919686	115.028781	213.9052114	198.3092674	198.1968154
CORO1B	3131.135521	2751.300536	2570.846495	3550.645998	3645.222372	3496.191823
TBL1XR1	4254.573051	4733.914057	5019.182662	5732.118132	5772.748386	5846.806054
B4GALNT1	1087.709835	1075.163526	995.9809084	837.5697727	788.6518843	814.5889112
ZNF318	824.7143004	878.4994453	906.2023476	1148.048223	1051.153747	1192.153845
ARHGEF11	820.7445565	911.4431745	940.8045013	1136.315026	1107.322268	1261.52273

SNTB2	873.3436635	1046.212976	1205.464217	794.2471983	568.5629864	772.96758
SECTM1	310.6324621	241.5873475	224.4464019	146.2136888	176.5296369	155.5845001
PELO	956.7082859	849.5488954	808.0070468	1204.909102	1101.590786	1121.793975
CLDN5	32.75038738	25.95566543	24.31502687	6.317875441	2.292592687	6.936888538
PXMP2	133.9788575	132.7732116	147.7605479	83.03493437	79.0944477	66.39593315
MRPL55	636.151464	456.2207347	509.680371	366.4367756	371.4000153	361.7091881
TP73	37.71256729	50.91303604	54.24121379	18.95362632	16.04814881	10.90082485
TERF1	465.4524752	518.1150138	523.7082711	665.1820286	686.6315098	673.8691723
USP21	635.159028	616.9462014	595.7181584	814.1033782	756.5555867	894.8586215
RAB35	2396.732895	2222.204279	2119.148111	2770.839658	2894.398267	2716.287355
HSPA14	304.6778462	342.4151247	317.0305427	191.3413705	228.1129724	224.9533855
PIK3AP1	466.4449112	610.9564324	543.3473313	388.0980628	378.2777934	372.6100129

LCK	61.53103084	55.90451016	45.82447372	15.34341178	22.92592687	20.81066562
KIF18B	1072.823296	1088.141358	1106.333723	835.7646655	683.1926207	901.79551
RASSF7	1188.938305	1015.265836	923.9710211	778.0012329	804.7000331	729.3642806
ZNF211	137.9486014	173.7032994	148.6957413	259.0328931	268.2333444	213.0615765
TANGO2	241.1619434	292.5003835	249.6966221	161.5571006	165.0666735	176.3951657
PIGQ	654.0153116	604.9666635	606.0052851	460.3023535	434.4463142	480.6272773
FARP2	559.7338934	676.8438908	686.4319125	494.5993917	382.8629787	466.7535002
ANXA4	724.4782663	670.8541219	699.5246192	531.6040907	508.9555765	532.1584493
TP53BP1	877.3134074	987.3135812	966.0547215	1259.964874	1114.200046	1368.54901
PAQR8	60.53859485	61.8942791	70.13950059	18.95362632	32.09629762	25.765586
NUP214	1598.814366	1459.507033	1622.560447	1218.447406	1256.340792	1228.820255
UBL7	522.0213261	449.2326709	422.7073902	307.7707893	332.4259396	333.9616339

TAF7	1265.355876	1234.890698	1202.658637	1546.976929	1938.387117	1470.62037
FANCB	118.0998818	117.7987893	162.7236414	66.78896895	64.19259524	78.28774208
RAD52	251.0863032	215.631682	188.9090549	352.8984711	302.6222347	340.8985225
FBXL6	495.2255546	463.2087985	405.8739101	323.1142011	316.3777908	318.1058887
UBR3	407.8911883	487.1678742	480.6893774	657.9615995	575.4407644	677.8331086
EEF1AKMT4	194.5174523	185.6828373	131.8622611	101.0860071	83.67963308	99.09840769
ZNF451	613.3254364	698.806377	784.6272133	941.3634407	920.4759638	959.2725865
HSCB	150.8502691	135.7680961	139.3438078	75.81450529	88.26481845	75.31478985
GOLT1B	874.3360994	986.3152864	1039.934995	1290.651697	1256.340792	1219.901399
CHCHD4	353.3072093	329.437292	400.26275	253.6175713	254.4777883	224.9533855
TAF5L	799.9034009	770.6836043	837.9332337	1015.372839	1030.520413	1056.389026
MYO15B	66.49321074	44.92326709	45.82447372	102.8911143	105.4592636	109.0082485

CRACR2A	317.579514	350.4014833	372.2069498	481.0610871	450.494463	551.9781308
ARHGEF1	1555.147183	1441.537726	1332.650511	1128.192043	1100.44449	1147.559561
DERL2	365.2164411	407.3042883	381.5588832	479.2559799	590.3426169	554.9510831
ATP2B4	571.6431252	625.9308548	661.1816922	882.6974545	761.1407721	849.2733539
OSTM1	172.6838607	186.6811321	235.668722	329.4320766	324.4018652	275.4935734
PPP1R12C	1776.460406	1559.336515	1438.327359	2196.815546	2043.84638	1951.247647
FBN1	77.41000653	79.86358594	92.58414078	41.51746718	32.09629762	40.63034715
STAG2	5453.435717	6736.493474	7175.738507	8262.878523	8042.415146	8775.164001
LRRC40	479.3465789	564.0365757	533.0602045	744.6067484	669.4370646	715.4905035
CRBN	342.3904135	349.4031885	317.965736	471.1329972	497.4926131	443.9608665
FAM200A	398.9592644	357.3895471	346.0215363	474.7432117	530.735207	536.1223856
MTMR6	470.4146551	540.0774999	527.4490445	766.2680356	722.1666964	626.3019366

AHDC1	1466.82038	1418.576945	1333.585705	1147.145669	1003.009301	1128.730864
UBE2E1	1128.399711	1102.117486	1182.084383	1392.640258	1586.474139	1396.296564
RABAC1	105.198214	92.84141866	72.94508062	39.71235991	42.41296471	44.59428346
RNF169	462.4751672	600.9734842	673.3392057	893.5280981	753.1166977	840.3544972
CPNE7	270.9350229	259.5566543	243.1502687	421.4925473	356.4981628	351.7993473
PSMD1	2245.882625	2308.057634	2416.539594	1816.840466	1928.07045	1901.698444
FAM120C	110.1603939	87.84994453	127.1862944	46.93278899	59.60740986	55.49510831
COL4A5	1043.050216	1216.921391	1356.030345	1796.081732	1454.65006	1631.159791
COX5A	1897.537596	1638.201807	1560.837687	1211.226977	1452.357467	1034.587376
C2CD4C	77.41000653	92.84141866	61.72276052	43.32257445	22.92592687	27.74755415
ZIC2	223.2980958	228.6095148	213.2240818	144.4085815	136.4092649	141.710723
ATP6V1F	851.5100719	836.5710627	673.3392057	561.3883606	586.9037279	577.7437168

AC099489.1	53.59154299	55.90451016	46.75966706	111.9166507	95.14259651	101.0803758
SYK	1054.959448	1163.01347	1096.046596	819.5187	918.1833712	816.5708794
ZSCAN22	142.9107813	116.8004944	142.1493879	201.2694605	210.9185272	238.8271625
PAM16	107.183086	136.7663909	109.4176209	57.7634326	42.41296471	74.32380577
PAFAH1B2	1959.068627	1966.640804	2106.055404	2446.822903	2578.020477	2420.9741
TTYH1	10.91679579	7.986358594	7.48154673	43.32257445	25.21851956	34.68444269
RAP1A	810.8201967	838.5676524	851.0259405	1002.737088	1150.881529	1088.100516
DDX60L	693.7127509	783.661437	812.6830135	604.7109351	526.1500217	563.8699398
GTF3C1	2261.761601	2398.902463	2421.21556	1973.884798	1819.172297	1927.46403
HNRNPD	7166.38022	6574.769713	6544.483002	5653.595966	5732.628014	5093.658155
CTSH	518.0515822	536.0843206	519.0323044	388.0980628	375.9852007	389.4567422
TAPBPL	320.5568219	291.5020887	343.2159562	217.5154259	205.1870455	220.9894492

ZBTB39	472.3995271	489.1644639	556.440038	658.8641531	676.3148427	721.436408
UBR4	4772.624634	5420.740896	5767.337335	7087.753691	6015.763211	7708.865134
TCN1	19.84871962	22.96078096	17.76867348	0.902553634	2.292592687	3.963936308
CENPK	646.0758238	682.8336598	701.3950059	515.3581252	482.5907606	522.2486085
ENOX1	45.65205514	32.9437292	30.86138026	72.20429075	90.55741114	81.26069431
LRRC8D	742.3421139	753.7125923	741.6083196	907.0664026	962.8889286	1048.461153
KIF13B	428.7323439	473.1917467	462.9207039	662.4743677	585.7574315	603.5093028
TMCO1	1152.218174	1153.030522	1096.046596	1384.517275	1625.448215	1382.422787
NDUFS4	867.3890476	788.6529112	706.0709726	1019.885607	1181.83153	983.0562043
TANK	477.361707	488.1661691	461.9855106	647.1309559	644.2185451	615.4011118
DOK3	37.71256729	54.90621533	41.14850701	86.6451489	113.483338	84.23364654
ENKUR	39.69743925	34.94031885	36.47254031	90.25536344	81.38704039	70.35986946

RBM4B	552.7868415	481.1781053	509.680371	657.0590458	745.0926233	682.788029
AAAS	1601.791674	1431.554778	1320.492998	1107.433309	1134.83338	1128.730864
RBMXL1	533.9305579	592.9871256	642.4778254	879.0872399	765.7259575	755.1298666
HOXB7	74.43269859	55.90451016	48.63005374	112.8192043	145.5796356	100.0893918
PTMS	3170.83296	2773.263022	2459.558487	1943.197975	2409.514914	1882.869746
RBSN	578.590177	579.0109981	621.9035719	883.6000081	739.3611416	764.0487233
FAM192A	1382.463322	1257.851479	1342.002445	916.9944926	1103.883379	1044.497217
ANAPC11	1772.490662	1325.735527	1088.565049	922.4098144	1008.740782	959.2725865
TMED3	1284.21216	1198.952084	1140.000683	988.2962297	910.1592968	916.6602711
ATP5F1B	28589.10331	26034.53072	25517.68551	22617.09152	21956.16016	22029.57603
TOMM7	2543.61342	1988.60329	1635.653154	1370.076417	1634.618586	1309.089966
GRIN1	203.4493761	175.6998891	217.9000485	111.9166507	128.3851905	124.8639937

OSGEPL1	166.7292448	163.7203512	183.2978949	105.5987752	102.0203746	100.0893918
SENP2	1497.585896	1543.363798	1558.9673	1840.306861	1946.411191	1901.698444
SERTAD2	532.9381219	584.0024722	506.8747909	734.6786584	716.4352147	709.5445991
TRIQK	304.6778462	357.3895471	379.6884965	480.1585335	492.9074277	491.5281021
PDK2	658.9774915	603.9683687	540.5417512	424.2002082	402.3500166	470.7174365
GABARAPL2	1154.203046	1010.274362	1050.222122	1334.876825	1454.65006	1332.873583
CCDC188	30.76551542	33.94202402	26.18541355	1.805107269	12.60925978	3.963936308
RPS6	28990.04745	24462.21637	25037.93132	30800.54533	35773.61629	32017.70454
GVQW3	88.32680233	90.84482901	101.0008809	51.44555716	27.51111224	49.54920385
NUMBL	408.8836243	430.2650693	391.84601	294.2324848	270.5259371	303.2411275
PILRB	1469.797688	1474.481455	1477.605479	1252.744445	1058.031525	1150.532513
RAPGEFL1	1218.711385	1166.008355	1142.806263	906.1638489	879.2092955	973.1463635

DNAJB11	1535.298463	1438.542842	1456.096032	1778.933213	2117.209346	1786.744291
RPL24	7920.631566	6874.25816	6592.177862	5513.700153	6134.978031	5087.712251
RCOR2	36.7201313	29.94884473	34.60215363	71.30173712	91.70370748	66.39593315
GRAMD1A	244.1392514	209.6419131	205.7425351	309.5758966	307.2074201	373.600997
MRPL57	738.37237	769.6853095	729.4508062	575.8292188	572.0018754	557.9240353
PIAS2	209.403992	219.6248613	243.1502687	321.3090938	347.3277921	319.0968728
IPO5	3114.264109	3327.316649	3547.188343	4084.055196	3984.52609	4188.889693
COPS9	475.376835	440.2480175	372.2069498	303.2580212	319.8166798	246.7550352
EXOC5	903.1167429	1039.224912	1148.417423	1414.301545	1305.631535	1342.783424
GSDMD	1440.024609	1273.824196	1152.158196	1004.542195	1012.179671	915.6692871
FTSJ3	2356.043019	2109.396964	2224.824959	1797.88684	1818.026001	1789.717243
ROBO4	0	0	2.805580024	18.95362632	12.60925978	20.81066562

ZBTB44	579.582613	580.0092929	672.4040123	807.7855028	772.6037355	865.1290991
CLCN6	491.2558107	466.2036829	470.4022506	627.2747759	602.9518767	690.7159016
EDRF1	261.0106631	335.427061	374.0773365	514.4555716	411.5203873	531.1674652
POPDC3	145.8880892	146.7493392	159.9180613	84.84004163	80.24074405	96.12545546
PHKB	1178.02151	1184.975956	1291.502004	944.9736552	896.4037406	1004.857854
SLC5A5	135.9637294	164.718646	136.5382278	83.03493437	93.99630017	65.40494908
GAP43	37.71256729	39.93179297	65.46353389	95.67068525	104.3129673	101.0803758
ADARB1	384.0727247	419.2838262	386.2348499	568.6087897	525.0037253	528.194513
R3HDM4	1435.062429	1282.808849	1147.48223	1686.872743	1920.046375	1541.971224
C19orf54	232.2300196	210.6402079	187.9738616	274.3763049	348.4740884	338.9165543
MIOS	404.9138803	479.1815156	552.6992647	669.6947967	635.0481743	708.553615
RSPO4	11.90923177	20.96419131	22.44464019	0.902553634	2.292592687	1.981968154

YWHAG	3153.961548	3332.308123	3262.889568	3825.924856	4005.159424	3932.224817
ABHD17A	1222.681129	1214.924801	1017.490355	933.240458	880.3555918	724.4093602
RND1	9.924359812	10.98124307	12.15751344	36.10214538	38.97407568	36.66641085
C6orf203	194.5174523	176.6981839	228.1871753	136.2855988	103.1666709	118.9180892
ZNHIT2	424.7626	343.4134195	294.5859025	244.5920349	233.8444541	215.0435447
SH2D2A	129.0166776	110.8107255	81.36182069	44.22512809	61.90000255	50.54018792
RRAGD	50.61423504	43.92497227	40.21331367	85.74259527	91.70370748	97.11643954
SVIP	468.4297831	544.0706792	584.4958383	425.1027618	353.0592738	330.9886817
NSD3	1057.936756	1206.938443	1285.890844	1548.782037	1434.016726	1625.213886
HM13	3279.008482	2852.128313	2880.395491	3810.581444	3769.022377	3589.344327
PRKRIP1	1032.13342	965.3510951	921.1654411	1398.958133	1416.822281	1098.010357
MRPL18	1198.862665	1067.177167	1032.453449	886.307669	842.5278125	805.6700545

CYBRD1	190.5477084	207.6453234	216.0296618	120.0396334	142.1407466	120.9000574
UBA6	1497.585896	1784.951146	1688.959174	2037.063553	2058.748233	2214.849412
ATPSCKMT	384.0727247	437.253133	409.6146835	267.1558758	259.0629736	324.0517932
TAOK2	1329.864215	1336.71677	1299.918744	1116.458846	990.4000408	1016.749663
COP1	596.4540247	573.0212291	582.6254516	766.2680356	796.6759587	732.3372328
ATG101	312.6173341	275.5293715	248.7614288	420.5899936	419.5444617	370.6280448
MRPL23	436.6718317	396.3230452	328.2528628	274.3763049	279.6963078	201.1697676
FAM214A	238.1846355	262.5515388	284.2987757	365.5342219	373.692608	388.4657581
ARL2	668.9018513	542.0740896	497.5228575	791.5395374	855.1370723	730.3552647
SAPCD1	45.65205514	32.9437292	28.05580024	12.63575088	3.438889031	9.909840769
LAMTOR2	587.5221009	504.1388862	370.3365631	307.7707893	338.1574213	317.1149046
CDK9	2068.236585	1942.681728	1789.024862	1486.505836	1607.107474	1510.259733

UBAC2	672.8715953	609.9581376	593.8477717	794.2471983	892.9648516	798.733166
PPFIA3	325.5190018	311.4679852	241.279882	209.3924432	191.4314894	141.710723
IKBKE	657.9850555	595.9820101	575.1439049	808.6880564	820.748182	776.9315163
TNNI3	59.54615887	66.88575323	73.88027396	22.56384086	34.38889031	27.74755415
CCNO	104.205778	94.8380083	104.7416542	57.7634326	51.58333546	45.58526754
ZBED6CL	186.5779645	199.6589649	173.9459615	132.6753843	80.24074405	109.0082485
NGLY1	443.6188836	429.2667744	383.4292699	560.485807	563.977801	580.7166691
SLC25A46	954.7234139	981.3238122	960.4435614	1187.760583	1271.242645	1199.090733
CCDC71	371.171057	348.4048937	314.2249627	234.6639449	239.5759358	237.8361785
VANGL2	70.46295467	95.83630313	83.23220737	31.5893772	41.26666837	42.61231531
PNMA2	0.992435981	3.993179297	2.805580024	20.75873359	25.21851956	14.86476115
ZNF561	230.2451476	195.6657856	191.714635	296.9401457	306.0611237	313.1509683

PITPNM1	2481.089953	2263.134367	2108.860984	2946.837616	2747.672335	2857.007094
UNKL	326.5114378	271.5361922	303.9378359	217.5154259	178.8222296	201.1697676
KCTD7	97.25872616	122.7902634	113.1583943	180.5107269	178.8222296	188.2869746
LGALS8	1046.027524	1151.033932	1184.889963	1382.712168	1443.187096	1454.764625
DNAAF3	246.1241233	242.5856423	187.9738616	136.2855988	151.3111173	133.7828504
TRAPPC10	741.349678	822.5949352	825.7757203	1114.653738	942.2555944	1075.217723
MYD88	694.7051869	608.9598428	692.0430725	774.3910183	1007.594486	944.4078253
ENTPD6	1681.186552	1582.297296	1514.078019	2118.29338	2083.966753	1838.275463
TMEM18	321.5492579	311.4679852	297.3914825	493.696838	460.8111301	380.5378855
PLEKHJ1	906.0940508	812.6119869	665.857659	592.0751842	557.1000229	576.7527328
TMTC1	13.89410374	15.97271719	20.57425351	58.66598624	38.97407568	47.56723569
EXOSC7	658.9774915	572.0229343	514.3563377	423.2976545	433.3000178	397.3846148

TIFA	260.0182271	302.4833317	329.1880561	414.2721182	467.6889082	403.3305193
CDC37L1	255.0560472	270.5378974	247.8262354	372.754651	367.9611263	358.7362358
ADPRHL2	843.570584	800.6324491	637.8018587	503.624928	620.1463218	486.5731818
GNPAT	754.2513457	755.709182	819.2293669	979.2706933	1014.472264	993.9570291
SWT1	67.48564672	67.88404805	74.8154673	143.5060279	112.3370417	125.8549778
ELP6	438.6567037	424.2753003	394.65159	325.821862	291.1592713	262.6107804
DHFR2	105.198214	150.7425185	155.2420946	80.32727346	79.0944477	58.46806054
ORC2	574.6204331	498.1491173	590.1069983	727.4582293	737.0685489	749.1839621
PPP1R18	2582.318423	2135.352629	2122.888885	2833.115858	3112.194573	2808.448874
HSPA4	4739.874246	4950.544033	5065.007136	5709.554291	5896.548391	6132.209468
TIMM9	283.8366906	234.5992837	238.474302	361.0214538	401.2037202	342.8804906
MTERF2	183.6006565	189.6760166	210.4185018	123.6498479	132.9703758	99.09840769

CDC7	859.4495597	907.4499952	1041.805382	744.6067484	665.9981756	705.5806628
MED21	527.975942	525.1030776	596.6533517	717.5301394	702.6796586	775.9405322
ARL6	113.1377019	113.80561	141.2141945	196.7566923	208.6259345	191.2599268
SEPHS2	1526.366539	1511.418364	1490.698186	1970.274584	2181.401942	1700.528676
OTX1	110.1603939	90.84482901	99.13049417	37.90725265	51.58333546	59.45904461
GABPB1	512.0969663	464.2070933	461.9855106	614.639025	652.2426195	654.0494908
PDS5B	753.2589097	936.4005452	993.1753284	1185.052922	1111.907453	1346.747361
HKDC1	8.931923831	16.97101201	14.96309346	39.71235991	44.7055574	42.61231531
GRK6	1634.542061	1473.483161	1420.558685	1230.180604	1216.22042	1086.118548
PFDN1	1134.354327	1039.224912	1017.490355	1359.245773	1464.966727	1267.468634
HACD4	163.7519369	172.7050046	237.5391087	334.8473984	270.5259371	299.2771912
MIGA2	878.3058434	707.7910304	663.9872723	580.3419869	518.1259473	523.2395926

ERP44	1051.98214	998.2948243	989.434555	1222.960175	1362.946352	1269.450603
UBR1	505.1499144	648.8916358	698.5894259	871.8668108	825.3333673	852.2463061
MASTL	761.1983976	737.7398751	836.0628471	542.4347343	622.4389145	595.5814302
EDARADD	135.9637294	152.7391081	133.7326478	74.00939802	85.97222576	82.25167838
ARFIP1	636.151464	651.8865202	718.2284861	907.9689562	949.1333724	801.7061182
GTF2H5	356.2845173	291.5020887	285.2339691	198.5617996	231.5518614	185.3140224
MED19	336.4357976	308.4731007	298.3266759	444.9589418	463.1037228	411.2583919
ELAVL1	1849.900669	1727.050046	1759.098675	1487.40839	1434.016726	1376.476883
CIAPIN1	1453.918712	1387.629806	1305.529904	1123.679275	1088.981526	1080.172644
NCOA5	670.8867233	646.8950461	570.4679381	797.8574128	849.4055905	839.3635131
KANSL3	977.5494415	994.301645	986.628975	1275.308285	1171.514863	1287.288316
CTDSPL2	925.9427705	1060.189103	1129.713556	1395.347919	1315.948202	1312.062918

DCTN3	1171.074458	887.4840988	830.451687	666.9871358	687.7778061	721.436408
SVEP1	264.980407	330.4355868	262.7893289	444.0563881	418.3981654	379.5469015
HDDC3	232.2300196	183.6862477	157.1124813	119.1370797	116.922227	101.0803758
RNF103	111.1528299	115.8021996	136.5382278	165.1673151	231.5518614	210.0886243
PRKCI	1940.212343	2247.161649	2483.873514	2771.742211	2760.281595	3056.194893
SOX15	1776.460406	1740.027879	1596.375033	1333.071718	1448.918578	1256.56781
AC007325.4	30.76551542	88.84823936	21.50944685	13.53830452	13.75555612	5.945904461
ZNF777	372.163493	361.3827264	343.2159562	447.6666027	516.9796509	527.2035289
IL36G	90.31167429	64.88916358	82.29701403	30.68682357	33.24259396	43.60329938
DNAJC8	2220.07929	1888.773807	1962.03563	1458.526673	1636.911179	1664.853249
EID2B	37.71256729	38.93349815	53.30602045	11.73319725	19.48703784	10.90082485
PCDHAC1	8.931923831	19.96589649	20.57425351	38.80980628	72.21666964	42.61231531

NUP88	960.6780298	1063.183988	1027.777482	1198.591226	1326.264869	1388.368692
RHEBL1	129.0166776	89.84653418	96.32491415	48.73789626	58.46111352	52.52215608
AC013394.1	16.87141168	21.96248613	17.76867348	51.44555716	42.41296471	58.46806054
HEPHL1	38.70500327	42.92667744	40.21331367	18.05107269	10.31666709	8.918856692
TECPR1	535.9154299	486.1695794	436.7352904	666.0845822	660.2666939	639.1847296
FAM204A	638.1363359	569.0280498	639.6722454	787.9293228	844.8204052	788.8233252
ECE1	2635.909966	2487.750702	2617.606162	3201.357741	3132.827907	3037.366196
PCM1	1212.756769	1482.467814	1610.402934	1853.845165	1783.637111	2061.24688
AGO4	282.8442546	332.4321765	315.160156	416.0772255	436.7389069	448.9157868
SUCO	517.0591462	649.8899306	675.2095924	838.4723264	795.5296624	863.147131
TNFSF9	222.3056598	175.6998891	186.1034749	136.2855988	108.8981526	107.0262803
MAGOH	772.1151934	766.690425	746.2842863	503.624928	665.9981756	434.0510257

ZNF419	194.5174523	214.6333872	210.4185018	269.8635367	309.5000127	348.8263951
NADSYN1	980.5267494	889.4806884	845.4147805	694.0637449	717.581511	671.8872041
AMZ2	1047.01996	1044.216386	1023.101515	833.9595582	843.6741088	763.0577392
CCDC106	615.3103084	547.0655637	534.9305912	432.3231909	435.5926105	355.7632836
KCTD17	379.1105448	312.46628	267.4652956	237.3716058	201.7481565	165.4943408
GANAB	13408.80254	13243.37914	12902.86253	11456.11328	10882.93749	11178.30039
CBX7	198.4871962	207.6453234	202.936955	148.018796	114.6296344	113.9631688
ANKRD52	2957.459224	3271.412139	3248.861667	2722.101761	2300.616761	2547.820062
VMAC	51.60667102	49.91474121	58.9171805	14.44085815	22.92592687	23.78361785
TENT4A	1816.157846	1865.813027	1844.201269	2247.35855	2279.983427	2195.02973
SLC35B1	943.8066181	821.5966404	812.6830135	1058.695413	1121.077824	1153.505466
RIC1	862.4268677	1074.165231	1146.547036	1431.450064	1298.753757	1366.567042

RBFA	162.7595009	124.786853	125.3159077	80.32727346	83.67963308	64.413965
DDAH2	593.4767168	569.0280498	474.143024	372.754651	426.4222398	372.6100129
CTAGE8	9.924359812	15.97271719	14.96309346	41.51746718	45.85185374	37.65739492
ARHGAP35	2393.755587	2543.655212	2448.336167	3182.404115	2895.544564	2940.249756
IL4R	2819.510623	2730.336344	2457.688101	2145.369989	2118.355643	2213.858428
HDDC2	603.4010766	488.1661691	560.1808114	376.3648655	410.374091	411.2583919
NR2C2	780.0546812	997.2965294	1033.388642	1248.231676	1166.929678	1374.494915
DSC3	784.0244252	853.5420747	993.1753284	676.9152258	610.9759511	671.8872041
PRR15	125.0469336	117.7987893	143.0845812	72.20429075	81.38704039	59.45904461
PRKACA	868.3814836	827.5864093	837.9332337	694.0637449	635.0481743	622.3380003
GPR146	5.954615887	9.982948243	11.22232009	32.49193084	37.82777934	29.72952231
DNM1L	2221.071726	2299.07298	2437.113847	2795.208606	2808.426042	2859.980046

PRR5	165.7368089	180.6913632	149.6309346	99.28089978	87.11852211	108.0172644
RITA1	950.75367	930.4107762	879.0817408	783.4165547	664.8518792	635.2207933
NKIRAS2	923.9578985	891.4772781	894.9800276	1165.196742	1202.464864	1075.217723
TNNT2	1.984871962	2.994884473	0	15.34341178	13.75555612	23.78361785
TBCB	893.1923831	917.4329435	716.3580994	602.0032741	641.9259524	629.2748888
GALNT2	2487.044569	2344.994542	2197.704352	2010.889497	1766.442665	1838.275463
SH3GL2	0	0	0	10.83064361	5.731481718	10.90082485
CD164	2072.206329	2217.212805	2494.160641	2825.895429	2945.981603	2731.152116
SLC39A9	993.4284172	1073.166936	1114.750463	1334.876825	1334.288944	1330.891615
FAM221A	9.924359812	13.97612754	12.15751344	41.51746718	35.53518665	37.65739492
ADH5	3041.816282	2692.401141	2758.820357	2353.859879	2326.981577	2218.813348
AMN1	102.2209061	133.7715065	114.0935876	158.8494397	239.5759358	197.2058313

FAM104A	562.7112013	594.9837153	576.0790982	702.1867276	797.8222551	777.9225004
FTL	11742.50253	10420.20138	10689.25989	7898.246855	9838.661516	8324.266246
POLR3G	577.5977411	699.8046718	676.1447857	456.692139	456.2259447	520.2666404
RIBC2	50.61423504	55.90451016	42.08370036	8.12298271	19.48703784	22.79263377
TIMP1	767.1530135	666.8609426	612.5516385	416.9797791	566.2703937	468.7354684
MTOR	1915.401444	2030.531673	2018.14723	1546.074376	1409.944503	1737.195087
LDOC1	586.5296649	538.0809103	444.2168371	355.606132	402.3500166	351.7993473
NUDT12	408.8836243	459.2156192	501.2636309	304.1605748	291.1592713	362.7001721
KCTD10	2141.676847	2205.233267	2126.629658	2538.883374	2646.798257	2653.855358
LYAR	1031.140984	983.3204019	978.2122349	823.1289146	764.5796611	753.1478985
CNIH1	1157.180354	1143.047574	1233.520017	1444.085815	1572.718583	1423.053134
SAMD8	648.0606957	711.7842097	764.9881531	973.8553715	884.9407772	914.678303

CYP4F11	131.0015495	147.747634	137.4734212	78.52216619	88.26481845	73.33282169
CYLD	447.5886275	477.184926	543.3473313	633.5926514	655.6815085	687.7429494
MEPCE	1735.770531	1634.208627	1575.80078	2162.518508	2273.105649	1862.059081
EML1	61.53103084	54.90621533	63.5931472	97.47579252	119.2148197	119.9090733
FKRP	557.7490214	542.0740896	452.6335772	740.9965338	727.8981781	641.1666978
TLE1	1885.628364	1733.039815	1663.708954	1383.614722	1405.359317	1447.827736
CLASP1	1092.672015	1264.839542	1288.696424	1606.545469	1445.479689	1615.304045
MAEA	1614.693341	1447.527495	1462.642386	1205.811656	1168.075974	1233.775176
MED9	293.7610504	298.4901525	281.4931957	181.4132805	212.0648236	201.1697676
ZNF428	319.564386	289.505499	253.4373955	210.2949968	184.5537113	173.4222135
MIIP	612.3330004	564.0365757	487.2357308	367.3393292	401.2037202	428.1051212
NRIP1	761.1983976	1021.255605	1111.944883	1335.779379	1279.266719	1341.79244

SDHAF4	97.25872616	109.8124307	97.26010749	61.37364714	37.82777934	53.51314015
ZADH2	237.1921995	242.5856423	304.8730292	171.4851905	183.407415	152.6115478
NDUFAF3	931.8973864	826.5881145	751.8954463	609.2237032	668.2907683	607.4732391
RALGAPA2	326.5114378	353.3963678	339.4751829	489.1840698	413.81298	522.2486085
METTL27	101.2284701	88.84823936	112.2232009	60.47109351	50.43703911	42.61231531
ZNF624	81.37975046	84.85506006	72.94508062	116.4294188	162.7740808	145.6746593
TNKS1BP1	5085.241968	4461.37957	4310.30611	5955.04888	5880.500242	5356.268936
FKBPL	188.5628364	144.7527495	161.788448	105.5987752	93.99630017	94.14348731
MCUR1	895.1772551	917.4329435	987.5641683	743.7041948	734.7759562	702.6077105
DUS2	387.0500327	302.4833317	356.308663	236.4690522	210.9185272	259.6378282
ACVR2A	86.34193037	67.88404805	101.0008809	143.5060279	166.2129698	139.7287548
SPPL2B	748.2967298	645.8967513	695.7838459	582.1470942	452.7870557	490.5371181

HIVEP1	425.7550359	531.0928465	606.9404785	702.1867276	704.9722513	774.9495481
PGGT1B	426.7474719	499.1474121	534.9305912	685.9407621	714.142622	601.5273347
PTPN11	6381.363359	7222.663053	7410.472036	8387.430925	8516.981832	8612.642612
RIOX2	506.1423504	594.9837153	561.1160047	767.1705892	726.7518818	703.5986946
YTHDC2	837.6159681	977.3306329	1026.842289	1328.55895	1107.322268	1317.017838
SPIN3	250.0938673	234.5992837	313.2897693	447.6666027	405.7889056	337.9255702
SAMM50	764.1757055	654.8814047	668.663239	508.1376962	530.735207	534.1404175
ATAD5	627.2195401	695.8114925	822.0349469	541.5321806	452.7870557	549.9961627
MOB2	421.785292	403.311109	274.011649	555.0704852	558.2463193	503.4199111
CFAP36	497.2104266	465.2053881	420.8370036	587.562416	678.6074354	606.4822551
TENT5A	207.4191201	259.5566543	279.622809	393.5133846	332.4259396	372.6100129
GPRC5B	299.7156663	239.5907578	228.1871753	170.5826369	150.164821	173.4222135

SLC5A6	600.4237686	545.068974	591.0421917	444.9589418	409.2277946	441.9788983
RAET1G	45.65205514	29.94884473	30.86138026	8.12298271	14.90185247	6.936888538
AGMAT	67.48564672	71.87722735	60.78756718	28.8817163	33.24259396	26.75657008
UQCRFS1	1666.300012	1565.326284	1548.680173	1275.308285	1329.703758	1252.603873
CLOCK	608.3632565	762.6972457	822.0349469	1046.962216	889.5259626	1058.370994
SPTB	16.87141168	11.97953789	13.09270678	44.22512809	40.12037202	39.63936308
CASTOR2	24.81089953	32.9437292	40.21331367	5.415321806	12.60925978	7.927872615
SENP1	402.9290084	410.2991728	410.5498768	554.1679315	511.2481692	576.7527328
GLMP	158.789757	203.6521441	180.4923149	259.9354467	270.5259371	286.3943982
S100A2	12978.08533	9450.857101	7257.100328	4953.214346	5028.802059	3279.166311
APP	13547.74358	13422.07391	12453.96973	10892.01726	11166.07268	11190.1922
LGALS2	26.79577149	21.96248613	22.44464019	47.83534262	73.36296599	55.49510831

TMEM65	574.6204331	611.9547273	616.2924119	443.1538345	472.2740935	444.9518505
TJP3	252.0787392	273.5327818	212.2888885	339.3601665	358.7907555	369.6370607
SLC45A4	315.594642	293.4986783	331.0584428	245.4945886	177.6759332	202.1607517
MAST3	131.0015495	145.7510443	139.3438078	76.71705892	60.75370621	95.13447138
USP38	317.579514	334.4287661	404.0035234	503.624928	458.5185374	516.3027041
MCRIP2	724.4782663	576.0161136	493.7820842	435.0308518	397.7648312	419.1862645
POU2F1	778.0698093	1040.223207	1071.731569	1396.250472	1155.466714	1414.134278
ZNF175	27.78820747	23.95907578	31.7965736	6.317875441	5.731481718	7.927872615
NOP14	1451.933841	1427.561599	1439.262552	1707.631476	1819.172297	1770.888545
DNMT3A	356.2845173	322.4492282	346.9567296	487.3789626	474.5666862	441.9788983
CCDC78	84.3570584	79.86358594	66.39872723	35.19959174	41.26666837	31.71149046
JAK3	77.41000653	88.84823936	85.10259405	26.1740554	40.12037202	50.54018792

AKR1E2	129.0166776	137.7646857	143.0845812	83.03493437	83.67963308	70.35986946
SMPDL3B	12.90166776	7.986358594	6.546353389	29.78426994	38.97407568	30.72050638
ABCB7	428.7323439	402.3128142	472.2726373	546.0449488	612.1222474	605.491271
CDC40	444.6113196	518.1150138	522.7730777	674.2075649	678.6074354	630.2658729
SERINC5	902.1243069	1070.172052	1120.361623	752.7297311	857.429665	711.5265672
DOK1	112.1452659	86.85164971	69.20430725	42.42002082	44.7055574	41.62133123
TRIM45	115.1225738	112.8073151	98.19530083	72.20429075	43.55926105	50.54018792
XRN2	2074.191201	2238.176996	2287.482913	2643.579595	2637.627886	2763.854591
LRRK1	527.975942	565.0348705	562.9863914	393.5133846	339.3037177	456.8436595
DUSP12	508.1272224	507.1337707	459.1799305	608.3211496	784.066699	632.2478411
HAUS4	344.3752855	316.4594593	360.0494364	254.5201249	239.5759358	213.0615765
TANC1	1058.929192	1175.991303	1214.81615	888.1127763	794.3833661	965.2184909

FAM167A	68.4780827	51.91133086	97.26010749	121.8447406	160.4814881	131.8008822
PRPF8	6205.702191	6657.628183	6591.242669	5188.780844	4683.76686	5784.374057
SLC37A2	1228.635745	1167.00665	1200.78825	985.5885688	958.3037432	926.5701119
ZBTB22	393.0046486	316.4594593	306.7434159	242.7869277	244.1611212	185.3140224
SUPV3L1	579.582613	580.0092929	613.4868318	790.6369837	771.4574392	735.3101851
EIF4G2	18715.35773	19842.10793	20971.71068	23215.48458	23822.33061	23725.14979
ZBTB40	994.4208532	1136.05951	981.9530083	1346.610023	1254.0482	1357.648185
PIK3CB	900.139435	945.3851986	931.4525679	1175.124832	1111.907453	1202.063685
TRA2B	4132.503426	4133.938867	4181.249429	4819.636408	4945.122426	4982.667939
TRMT61B	180.6233486	209.6419131	191.714635	287.9146094	264.7944554	305.2230957
RB1	1434.069993	1782.954556	1911.535189	2232.917692	2110.331568	2341.695374
MAP7D1	3739.498777	3365.251853	3091.749186	2806.941803	2819.889005	2375.388832

KRT16	318.57195	237.5941682	190.7794416	153.4341178	152.4574137	149.6385956
AC068831.7	128.0242416	160.7254667	212.2888885	88.45025617	72.21666964	112.9721848
LBR	1632.557189	1823.884644	1766.580222	2211.256404	2044.992677	2187.101858
SAR1B	656.9926196	665.8626478	710.7469393	869.1591499	946.8407797	822.5167838
EXOC6	113.1377019	132.7732116	144.9549679	192.2439241	222.3814906	205.1337039
RDH11	1973.955167	2005.574302	2017.212037	2346.639449	2420.977878	2488.361017
ADD3	1007.322521	1162.015175	1166.186097	857.4259527	884.9407772	866.1200832
SCLY	84.3570584	98.8311876	90.7137541	55.95832533	27.51111224	45.58526754
ELP3	648.0606957	635.9138031	647.1537921	801.4676274	821.8944783	852.2463061
SNAP29	516.0667102	481.1781053	485.3653441	373.6572046	369.1074226	355.7632836
FP565260.1	527.975942	657.8762892	538.6713645	361.9240074	439.0314996	435.0420098
TRIP11	481.3314509	535.0860258	519.0323044	656.1564922	640.779656	744.2290418

SFXN3	931.8973 864	836.5710 627	788.3679 867	669.6947 967	676.3148 427	597.5633 984
ORAI3	216.3510 439	164.7186 46	157.1124 813	119.1370 797	103.1666 709	98.10742 361
PHF10	596.4540 247	662.8677 633	629.3851 186	504.5274 816	434.4463 142	474.6813 728
DSTYK	385.0651 607	410.2991 728	459.1799 305	642.6181 877	525.0037 253	556.9330 512
MPRIP	5611.233 038	6316.211 353	6017.033 957	7622.967 996	6912.166 951	7181.661 605
GPM6A	63.51590 28	49.91474 121	54.24121 379	118.2345 261	90.55741 114	108.0172 644
HUWE1	4530.470 254	5061.354 759	5318.444 532	6236.645 614	5638.631 714	6801.123 72
RPL5	19577.78 46	17579.97 186	16305.09 59	20600.78 671	24677.46 768	21625.25 453
KCND1	6.947051 869	10.98124 307	10.28712 675	34.29703 811	24.07222 321	42.61231 531
AP002990. 1	63.51590 28	46.91985 674	58.91718 05	27.07660 903	12.60925 978	24.77460 192
SNCG	186.5779 645	176.6981 839	160.8532 547	106.5013 289	95.14259 651	118.9180 892
FDPS	1467.812 816	1322.740 642	1254.094 271	1091.187 344	1041.983 376	1061.343 946

SCD	12474.92028	13416.08414	13161.91108	15207.12619	16161.63215	14989.62515
TRIM68	226.2754037	245.5805268	251.5670088	333.0422911	324.4018652	377.5649333
ACSF2	360.2542612	321.4509334	320.771316	238.2741595	245.3074175	215.0435447
COPB1	3509.25363	3585.875009	3670.633864	4119.254787	4291.73351	4516.905423
MATK	419.8004201	370.3673798	357.2438563	310.4784502	223.527787	242.7910988
CYB5B	2969.368456	2897.05158	3023.480072	2520.832301	2369.394542	2490.342985
TM9SF4	2077.168509	2004.576007	1967.64679	2368.300737	2410.66121	2600.342218
CYGB	25.80333551	23.95907578	27.1206069	5.415321806	3.438889031	7.927872615
CTTN	8141.94479	7692.859916	7170.127347	6381.956749	6561.40027	6154.011118
C21orf91	230.2451476	271.5361922	300.1970625	427.8104227	359.9370519	371.6190288
MTMR1	807.8428887	861.5284333	885.6280941	1045.157109	1163.490789	1049.452137
NOG	4.962179906	2.994884473	3.740773365	21.66128723	24.07222321	18.82869746

ALDOC	1560.109362	1391.622985	1331.715318	1074.941379	1153.174122	1154.49645
HLA-F	211.388864	179.6930684	159.9180613	280.6941803	288.8666786	258.6468441
ATG10	228.2602757	212.6367976	214.1592751	163.3622078	129.5314868	123.8730096
MGAT5	736.3874981	889.4806884	925.8414078	1133.607365	1130.248195	1065.307883
PEPD	610.3481284	563.0382809	533.0602045	426.0053154	381.7166824	450.897755
RAVER1	825.7067364	839.5659472	766.8585398	679.6228867	593.7815059	580.7166691
BRD9	2342.148916	2335.011594	2160.296618	1823.158342	1824.903779	1942.328791
CADM4	268.9501509	200.6572597	175.8163482	138.0907061	139.8481539	110.9902166
PRKG2	56.56885093	80.86188076	78.55624066	135.3830452	124.9463014	124.8639937
N4BP2	239.1770715	300.4867421	368.4661764	447.6666027	419.5444617	468.7354684
KLHL31	40.68987523	30.94713955	36.47254031	14.44085815	8.024074405	9.909840769
CEMIP2	894.1848191	1004.284593	1150.28781	1350.220237	1255.194496	1358.639169

SSBP2	418.8079841	404.3094038	383.4292699	581.2445406	483.737057	573.7797805
RTN3	1725.846171	1831.871003	1832.978949	2165.226169	2516.120474	2089.985418
AC011529.1	217.3434799	204.650439	196.3906017	131.7728306	130.6777832	137.7467867
MAP3K20	1071.83086	1240.880467	1336.391285	1609.25313	1481.014876	1581.610587
TGFBR3L	58.55372289	57.90109981	52.37082711	25.27150176	26.3648159	17.83771338
TRIM37	839.6008401	962.3562106	887.4984808	1211.226977	1051.153747	1190.171876
TYRO3	662.9472355	591.9888308	594.782965	453.0819245	487.175946	454.8616913
IP6K2	1113.513171	932.4073659	1023.101515	1283.431268	1375.555612	1255.576825
ZCCHC4	252.0787392	221.621451	231.9279486	348.3857029	308.3537164	351.7993473
DROSHA	2553.53778	2833.160711	2735.440523	2044.283982	1921.192672	2421.965084
POLR2C	1562.094234	1515.411543	1395.308465	1127.289489	1255.194496	1186.20794
LAMTOR5	980.5267494	833.5761783	714.4877127	588.4649696	667.1444719	596.5724143

MIF4GD	430.7172158	346.408304	397.45717	260.8380003	286.5740859	282.4304619
METTL3	819.7521205	739.7364648	786.4976	567.706236	572.0018754	652.0675226
INPP5F	389.0349046	466.2036829	431.1241303	301.4529139	243.0148248	341.8895065
C3orf14	182.6082205	185.6828373	183.2978949	114.6243116	129.5314868	102.0713599
KRT86	6.947051869	15.97271719	8.416740071	37.00469901	33.24259396	35.67542677
CRYBB3	15.8789757	22.96078096	30.86138026	6.317875441	2.292592687	3.963936308
STAC3	46.64449112	47.91815156	66.39872723	97.47579252	102.0203746	108.0172644
AFAP1L2	297.7307944	359.3861367	323.5768961	231.956284	217.7963053	230.8992899
PPIG	1149.240866	1195.957199	1246.612724	1469.357317	1575.011176	1428.999039
LUC7L3	3633.308127	3881.370277	4241.101802	4628.295037	4690.644638	5122.396694
ZBTB32	14.88653972	18.96760166	14.02790012	2.707660903	0	0.990984077
CDC42BPG	1039.080472	1050.206155	1016.555162	1381.809614	1199.025975	1323.954727

TTC27	510.1120943	465.2053881	551.7640713	660.6692604	675.1685463	679.8150768
BROX	806.8504527	935.4022503	949.2212413	1123.679275	1156.613011	1152.514481
ODC1	3239.311043	3067.759995	2970.174052	2435.089706	2706.405667	2465.568383
HECTD1	1625.610137	1934.695369	2077.064411	2431.479491	2301.763058	2432.865909
CWF19L2	407.8911883	472.1934519	496.5876642	625.4696686	591.4889133	627.2929207
STXBP1	934.8746943	897.467047	813.6182069	713.0173712	582.3185425	701.6167265
ING1	211.388864	180.6913632	159.9180613	314.9912184	255.6240846	265.5837326
BCL11B	135.9637294	131.7749168	153.371708	94.76813161	63.04629889	81.26069431
OBSL1	272.9198948	241.5873475	213.2240818	148.018796	179.9685259	142.7017071
SBDS	1183.976126	1105.11237	1155.89897	1334.876825	1618.570437	1430.981007
ABHD3	252.0787392	233.6009889	225.3815952	163.3622078	171.9444515	118.9180892
MBOAT2	471.4070911	497.1508225	529.3194311	351.0933638	349.6203848	399.366583

CHST12	519.0440182	424.2753003	405.8739101	295.1350385	324.4018652	335.9436021
SMAP1	76.41757055	111.8090203	94.45452746	144.4085815	189.1388967	152.6115478
RANBP2	1635.534497	1896.760166	2197.704352	2466.679083	2344.176022	2614.215995
CBFA2T2	957.7007219	997.2965294	928.6469878	760.8527138	738.2148452	776.9315163
ZNF443	4.962179906	13.97612754	7.48154673	0	0	0
VPS41	1033.125856	1017.262426	1108.204109	1329.461503	1264.364867	1345.756376
GZF1	680.8110831	759.7023613	727.5804195	910.6766171	896.4037406	964.2275068
MRPL10	1218.711385	1074.165231	1057.703669	1335.779379	1585.327843	1383.413771
CNNM2	280.8593827	258.5583595	274.011649	373.6572046	354.2055701	411.2583919
EEA1	468.4297831	566.0331654	581.6902582	723.8480148	687.7778061	742.2470736
TMEM237	571.6431252	591.9888308	587.3014183	437.7385127	447.055574	446.9338187
BCS1L	684.780827	591.9888308	560.1808114	440.4461736	436.7389069	482.6092455

TCERG1	2086.100433	2260.139482	2300.575619	2712.173671	2557.387142	2899.619409
NUF2	799.9034009	742.7313492	910.8783144	602.9058278	621.2926182	643.1486659
RXYLT1	396.9743925	415.2906469	367.5309831	300.5503603	262.5018627	280.4484938
RAB5IF	759.2135256	799.6341542	750.960253	648.0335095	577.7333571	449.9067709
UBE2G2	1910.439264	1995.591354	1948.942923	1602.935255	1549.792656	1653.952424
SERTAD4	147.8729612	153.7374029	183.2978949	218.4179795	255.6240846	269.5476689
PCCA	105.198214	112.8073151	110.3528143	170.5826369	170.7981552	181.3500861
IL27RA	393.0046486	354.3946626	392.7812033	255.4226785	292.3055676	267.5657008
SEC13	1485.676664	1336.71677	1298.983551	1097.505219	996.1315225	1147.559561
EXOSC8	1013.277137	1032.236848	1022.166322	1232.888265	1403.066724	1228.820255
ST6GALNAC5	61.53103084	71.87722735	57.98198716	129.9677234	107.7518563	108.0172644
ANKRD33B	846.547892	952.3732623	1027.777482	1301.482341	1138.272269	1205.036638

APTX	1215.734077	1074.165231	1111.944883	849.30297	931.9389273	901.79551
NBPF15	423.770164	454.224145	446.0872238	591.1726305	558.2463193	597.5633984
RELL1	213.373736	200.6572597	207.6129218	122.7472943	159.3351917	109.9992325
AC008403.1	92.29654625	86.85164971	98.19530083	55.0557717	48.14444643	38.648379
TMEM9	1249.4769	1160.018586	1098.852176	942.2659943	927.3537419	914.678303
TAF11	443.6188836	410.2991728	432.0593236	296.9401457	350.7666811	271.5296371
TM2D2	307.6551542	286.5106146	273.0764556	209.3924432	209.7722309	167.476309
GCNT3	29.77307944	37.93520332	24.31502687	7.220429075	11.46296344	5.945904461
SRPRA	2789.737543	2625.515388	2574.587268	2146.272543	2210.05935	2252.506807
PLCD3	1754.626815	1709.080739	1538.393046	1354.733005	1356.068574	1318.999806
KLHL35	103.213342	121.7919686	101.0008809	34.29703811	60.75370621	69.36888538
WNT10B	187.5704004	170.7084149	146.8253546	120.0396334	93.99630017	85.22463061

BMP6	49.62179906	57.90109981	59.85237384	24.36894813	24.07222321	21.80164969
GLRX2	335.4433617	297.4918576	317.965736	412.4670109	539.9055778	406.3034715
TXNDC5	161.7670649	166.7152357	142.1493879	120.942187	72.21666964	54.50412423
ENPP5	30.76551542	51.91133086	35.53734697	9.025536344	11.46296344	16.84672931
NDUFB1	775.0925013	572.0229343	530.2546245	348.3857029	468.8352045	460.8075958
FSIP2	18.85628364	37.93520332	43.0188937	67.69152258	65.33889158	100.0893918
GIN1	163.7519369	180.6913632	190.7794416	252.7150176	264.7944554	271.5296371
ECI1	1770.50579	1657.169408	1466.383159	1344.804915	1315.948202	1178.280067
STARD9	182.6082205	186.6811321	192.6498283	129.0651697	105.4592636	120.9000574
SMG1	2120.835692	2796.223803	2962.692505	3599.383894	3059.464941	3893.576438
LNP1	35.72769532	38.93349815	41.14850701	9.928089978	11.46296344	16.84672931
CAMKMT	125.0469336	94.8380083	110.3528143	170.5826369	189.1388967	173.4222135

ABCA3	24.81089953	18.96760166	29.92618692	50.54300353	67.63148427	58.46806054
AL590560.1	18.85628364	26.95396025	22.44464019	5.415321806	1.146296344	5.945904461
PPP6C	1171.074458	1248.866825	1313.946644	1542.464161	1639.203771	1476.566275
TCEANC2	240.1695075	250.5720009	299.2618692	175.0954051	166.2129698	186.3050065
ANO10	333.4584897	315.4611645	347.8919229	255.4226785	224.6740833	218.0164969
LDB1	1500.563204	1564.32799	1296.177971	1159.78142	994.9852262	1206.027622
SPRTN	271.9274589	368.3707902	356.308663	472.9381044	476.8592789	452.8797231
NHP2	1174.051766	1076.161821	1001.592068	861.9387209	889.5259626	629.2748888
FICD	71.45539065	81.86017559	97.26010749	37.90725265	48.14444643	37.65739492
THAP11	611.3405644	557.0485119	531.1898178	437.7385127	397.7648312	434.0510257
ACAD8	419.8004201	371.3656746	398.3923634	280.6941803	312.9389018	256.6648759
RNGTT	472.3995271	437.253133	438.605677	628.1773295	544.4907632	618.374064

E2F6	475.376835	467.2019778	490.0413108	665.1820286	621.2926182	593.5994621
HDAC6	438.6567037	457.2190295	496.5876642	342.9703811	325.5481616	355.7632836
IFITM10	0	0.998294824	0	9.025536344	12.60925978	14.86476115
BFAR	1074.808168	1033.235143	976.3418482	1248.231676	1432.870429	1232.784192
KCNJ12	53.59154299	41.92838262	51.43563377	101.0860071	84.82592942	95.13447138
RAB8A	464.4600392	434.2582486	444.2168371	308.673343	356.4981628	321.0788409
SLC6A8	3629.338383	3339.296187	2924.349578	4000.117708	4407.509441	3961.954339
ADAMTS6	74.43269859	101.8260721	80.42662735	136.2855988	124.9463014	196.2148472
ARMC8	534.9229939	575.0178188	594.782965	780.7088938	708.4111403	723.4183761
GPR176	315.594642	298.4901525	300.1970625	395.3184919	508.9555765	391.4387104
SHPRH	156.804885	228.6095148	273.0764556	399.83126	265.9407517	412.249376
UGP2	1094.656887	1174.993008	1060.509249	1336.681933	1498.209321	1344.765392

WDR35	317.579514	361.3827264	345.0863429	496.4044989	464.2500191	435.0420098
LPGAT1	1348.720498	1505.428595	1682.412821	1952.223511	1847.829706	1922.509109
TDRD5	309.6400261	322.4492282	320.771316	410.6619037	486.0296497	416.2133123
PLXNA3	1005.337649	965.3510951	952.0268214	1206.714209	1156.613011	1292.243236
MISP	1849.900669	1636.205217	1700.181494	2183.277242	2456.513064	1954.2206
ZDHHC24	515.0742743	465.2053881	439.5408704	333.0422911	374.8389043	330.9886817
ATP23	200.4720682	207.6453234	204.8073417	139.8958133	145.5796356	86.21561469
VPS36	899.146999	904.4551108	1051.157316	1206.714209	1216.22042	1193.144829
PSD3	747.3042939	925.4193021	952.9620147	1250.939337	1022.496338	1216.928446
TNPO2	2108.92646	2121.376502	2093.897891	1839.404307	1492.477839	1721.339342
MTF1	420.792856	410.2991728	468.531864	621.8594541	520.41854	607.4732391
MYL5	46.64449112	37.93520332	36.47254031	16.24596542	8.024074405	15.85574523

ACTL8	62.52346682	47.91815156	50.50044043	23.46639449	22.92592687	19.81968154
ZKSCAN2	88.32680233	117.7987893	142.1493879	164.2647615	231.5518614	193.241895
FAHD2A	355.2920813	312.46628	330.1232495	237.3716058	228.1129724	240.8091307
PDXP	217.3434799	258.5583595	215.0944685	170.5826369	113.483338	150.6295797
NUDCD3	1838.983873	1665.155767	1561.77288	1362.855988	1349.190796	1357.648185
THNSL1	189.5552724	187.679427	212.2888885	108.3064361	150.164821	111.9812007
RPL36	5559.626367	4872.677037	4380.44561	3934.231292	4222.95573	3404.030304
KRTAP5-1	0.992435981	1.996589649	1.870386682	18.95362632	14.90185247	12.882793
RASSF3	657.9850555	726.7586321	717.2932927	539.7270734	489.4685387	571.7978124
DYM	344.3752855	317.4577541	340.4103762	482.8661944	433.3000178	442.9698824
TOMM5	61.53103084	84.85506006	63.5931472	28.8817163	30.95000127	36.66641085
PDS5A	2266.723781	2669.44036	2801.83925	3195.039866	3039.977903	3532.858234

NDC80	795.9336569	720.7688631	903.3967676	552.3628243	589.1963206	667.9232678
AUNIP	352.3147733	362.3810212	346.0215363	251.812464	253.3314919	258.6468441
NDUFB9	2591.250347	2270.12243	2085.481151	1781.640874	1995.701934	1639.087663
NCK2	432.7020878	446.2377864	427.3833569	587.562416	551.3685412	575.7617487
RNF168	902.1243069	944.3869037	899.6559943	730.1658902	751.9704013	684.7699971
SYF2	731.4253182	675.845596	736.9323529	861.9387209	1093.566712	879.0028762
C1orf21	499.1952986	464.2070933	454.5039638	333.9448447	361.0833482	357.7452518
SNX8	857.4646878	745.7262337	663.9872723	562.2909142	578.8796535	563.8699398
MPP5	949.761234	984.3186967	1096.981789	1322.241074	1251.755607	1238.730096
TMX1	1506.517819	1569.319464	1567.38404	1331.266611	1248.316718	1166.388259
PCOLCE2	204.4418121	194.6674907	159.9180613	129.9677234	116.922227	98.10742361
CAB39L	85.34949438	92.84141866	81.36182069	135.3830452	142.1407466	153.6025319

SAFB	2005.713118	1942.681728	1964.84121	2361.982861	2440.464915	2308.992899
TMEM187	136.9561654	91.84312383	105.6768476	41.51746718	77.94815136	50.54018792
DNM1	947.7763621	952.3732623	1007.203229	801.4676274	694.6555842	781.8864367
ATIC	2383.831227	2408.885411	2343.594513	1984.715442	1946.411191	2017.643581
LAPTM5	87.33436635	58.89939463	39.27812033	13.53830452	29.80370493	26.75657008
CBX5	3073.574234	3393.204108	3724.875078	2816.869893	2618.140849	2808.448874
KIAA0895L	472.3995271	404.3094038	414.2906502	348.3857029	301.4759383	259.6378282
RWDD4	351.3223373	308.4731007	335.7344095	217.5154259	245.3074175	240.8091307
PPP2CB	1461.8582	1407.595702	1510.337246	1738.3183	1840.951928	1764.942641
POM121	1573.01103	1685.121663	1660.903374	2044.283982	1860.438966	2179.173985
SAV1	1255.431516	1385.633216	1335.456091	1596.617379	1704.542663	1597.466332
PERM1	61.53103084	55.90451016	56.11160047	24.36894813	28.65740859	22.79263377

NUP37	848.5327639	792.6460905	825.7757203	639.9105268	662.5592866	638.1937455
NSUN6	191.5401444	226.6129251	215.0944685	309.5758966	283.1351968	317.1149046
AQP3	4294.270491	4038.102564	3805.301705	3101.174288	3652.10015	2969.979279
NCOA3	2445.362258	3069.756585	3068.369353	3683.321382	3363.233472	3840.063298
POM121C	630.1968481	747.7228234	661.1816922	824.9340218	894.1111479	923.5971597
PHC1	275.8972028	299.4884473	327.3176694	417.8823327	390.8870531	436.0329938
SASH1	286.8139986	319.4543438	304.8730292	420.5899936	378.2777934	460.8075958
MTUS2	4.962179906	2.994884473	6.546353389	21.66128723	27.51111224	19.81968154
PEAK1	376.1332369	472.1934519	454.5039638	642.6181877	511.2481692	646.1216181
C2orf42	111.1528299	75.87040664	111.2880076	184.1209414	154.7500064	159.5484364
BMF	48.62936308	44.92326709	56.11160047	18.95362632	21.77963053	19.81968154
CUEDC1	774.1000653	709.78762	580.7550649	932.3379043	960.5963359	853.2372902

ZNF112	48.62936308	49.91474121	65.46353389	93.86557798	126.0925978	92.16151915
SIAH1	228.2602757	218.6265665	221.6408219	314.0886648	363.3759409	285.4034142
VIP	0	0	0.935193341	5.415321806	18.3407415	12.882793
NBN	899.146999	1034.233438	1106.333723	1250.939337	1272.388941	1349.720313
MICAL1	257.0409191	263.5498336	231.9279486	319.5039866	371.4000153	371.6190288
FLOT1	1886.6208	1579.302412	1612.27332	2039.771214	2224.961203	2082.057546
FOXO4	90.31167429	119.7953789	89.77856076	153.4341178	168.5055625	169.4582772
GNL3L	558.7414574	531.0928465	636.8666654	753.6322847	716.4352147	787.8323411
DENND2D	371.171057	424.2753003	383.4292699	564.0960215	504.3703911	510.3567996
OPN3	33.74282336	36.9369085	30.86138026	11.73319725	9.170370748	10.90082485
BDH2	178.6384766	167.7135305	158.982868	95.67068525	121.5074124	97.11643954
RTN4RL1	53.59154299	44.92326709	54.24121379	10.83064361	28.65740859	18.82869746

IFNE	17.86384766	22.96078096	30.86138026	55.0557717	59.60740986	55.49510831
PRR13	2211.147366	1982.613521	1919.016736	1482.895621	1814.587112	1513.232685
NEIL3	425.7550359	422.2787107	446.0872238	326.7244157	284.2814932	334.952618
PSMA4	2651.788942	2495.737061	2574.587268	2022.622695	2202.035276	2174.219065
SCMH1	508.1272224	446.2377864	472.2726373	601.1007205	622.4389145	646.1216181
PDE12	522.0213261	590.990536	558.3104247	765.365482	686.6315098	716.4814876
RPS2	48480.49768	40214.31041	37742.53287	33365.60276	36101.45704	31329.96159
JMJD4	195.5098883	172.7050046	139.3438078	95.67068525	123.8000051	78.28774208
PSMD4	3677.967746	3104.696903	2915.932838	2499.171014	2731.624187	2474.48724
OR52E6	8.931923831	4.991474121	10.28712675	0	0	0
ORAI2	982.5116214	912.4414694	952.9620147	789.7344301	758.8481794	689.7249175
KCTD13	463.4676032	480.1798105	437.6704837	342.0678274	275.1111224	371.6190288

CCDC85B	3149.991804	2539.662033	2277.195786	2086.704003	2184.840831	1641.069631
SCX	45.65205514	41.92838262	36.47254031	84.84004163	97.4351892	67.38691723
DPP8	550.8019696	633.9172134	692.0430725	804.1752883	790.944477	866.1200832
XRRA1	258.0333551	238.592463	299.2618692	370.0469901	351.9129775	400.3575671
NOTCH2	1779.437714	2047.502685	2282.806946	2856.582253	2241.009352	2938.267788
CLK2	1054.959448	1049.20786	1043.675769	1262.672535	1267.803756	1350.711297
ARL5A	430.7172158	426.27189	461.0503172	370.0469901	287.7203822	277.4755415
HSDL2	1455.903584	1506.42689	1639.393927	1246.426569	1168.075974	1289.270284
GATB	390.0273406	393.3281608	346.9567296	282.4992876	255.6240846	275.4935734
CNDP2	697.6824948	663.8660581	632.1906987	537.9219661	503.2240948	490.5371181
BORCS5	169.7065528	185.6828373	188.9090549	267.1558758	264.7944554	257.65586
DYNC1H1	5679.71112	6491.911242	6372.407427	7607.624584	7035.966957	7762.378274

OGFOD1	882.2755873	927.4158917	986.628975	742.8016411	759.9944758	702.6077105
FO681492.1	57.56128691	26.95396025	57.04679381	18.05107269	10.31666709	19.81968154
AK4	1500.563204	1514.413248	1444.873712	1813.230252	1884.511189	1733.231151
RPS6KA3	1070.838424	1145.044163	1152.158196	1348.41513	1368.677834	1436.926912
UBE2E3	1502.548076	1292.791797	1396.243658	1055.987752	1227.683384	964.2275068
FIBIN	0	0	0.935193341	7.220429075	14.90185247	12.882793
CTRB2	0.992435981	0.998294824	0.935193341	15.34341178	10.31666709	15.85574523
MTMR9	184.5930925	195.6657856	212.2888885	312.2835575	262.5018627	283.421446
MAP3K11	2263.746473	2032.528262	2018.14723	2567.76509	2630.750108	2478.451176
THOC6	1061.9065	842.5608317	779.9512466	653.4488313	668.2907683	683.7790131
CCDC142	263.987971	234.5992837	271.206069	385.3904019	356.4981628	335.9436021
PJA2	1589.882442	1855.830078	1763.774642	2225.697262	2044.992677	2151.426431

ANKRD18B	166.7292448	301.4850369	246.8910421	162.4596542	120.3611161	143.6926912
ZNF91	16.87141168	16.97101201	16.83348014	1.805107269	2.292592687	2.972952231
ELF2	379.1105448	433.2599537	424.5777769	581.2445406	518.1259473	546.0322264
TRIOBP	3849.659171	3552.93128	3415.326082	2914.345686	3061.757534	3013.582578
AC011604.2	11.90923177	6.98806377	13.09270678	31.5893772	32.09629762	38.648379
RPL23	10841.37066	9339.048081	9060.15309	11338.78131	13733.77649	11538.02761
MECOM	539.8851738	607.961548	639.6722454	434.1282981	356.4981628	498.4649907
URI1	986.4813653	1026.247079	1068.925989	798.7599665	833.3574417	832.4266246
PRKD1	35.72769532	39.93179297	42.08370036	78.52216619	76.80185502	78.28774208
SLC35F3	36.7201313	48.91644639	41.14850701	89.35280981	91.70370748	70.35986946
LRP6	806.8504527	1011.272657	1079.213116	1433.255171	1099.298193	1375.485899
ABCD4	659.9699275	675.845596	616.2924119	527.0913225	481.4444643	490.5371181

DDIAS	522.0213261	624.93256	572.3383248	440.4461736	371.4000153	455.8526754
IFT22	1037.0956	864.5233178	796.7847267	677.8177794	695.8018805	678.8240927
COQ3	333.4584897	323.4475231	314.2249627	240.0792668	236.1370468	215.0435447
RHBDD2	447.5886275	442.2446071	415.2258435	317.6988793	334.7185323	316.1239205
MT-CO3	97176.35397	88027.64101	90894.24641	63124.60119	80721.04221	78144.04938
DNAJA4	650.0455677	650.8882254	707.9413593	846.5953091	841.3815161	857.2012265
UBE2A	1683.171424	1641.196691	1740.394808	1318.63086	1405.359317	1427.017071
SLAMF9	66.49321074	73.87381699	36.47254031	22.56384086	17.19444515	29.72952231
UTP18	1527.358975	1476.478045	1477.605479	1795.179179	1905.144523	1749.086896
RNF157	102.2209061	110.8107255	78.55624066	166.0698687	139.8481539	176.3951657
CD58	436.6718317	436.2548382	504.0692109	579.4394333	631.6092853	607.4732391
CYFIP2	2.977307944	5.989768946	4.675966706	20.75873359	20.63333418	23.78361785

HTRA2	362.2391331	303.4816266	316.0953493	237.3716058	231.5518614	224.9533855
ONECUT3	54.58397897	51.91133086	38.34292699	72.20429075	131.8240795	90.179551
IER3	8781.073562	8525.437799	7418.888776	9961.484463	10329.27635	9626.419323
SOAT1	1055.951884	1156.025406	1269.992557	911.5791708	898.6963333	940.443889
TAF12	458.5054233	428.2684796	424.5777769	299.6478066	363.3759409	291.3493186
DCK	552.7868415	593.9854204	565.7919714	467.5227826	425.2759434	402.3395352
TMEM159	471.4070911	503.1405914	457.3095439	353.8010247	364.5222372	355.7632836
RBM43	144.8956533	187.679427	206.6777284	126.3575088	89.41111479	109.0082485
TMEM116	130.0091135	147.747634	138.4086145	88.45025617	85.97222576	76.30577392
GRN	4407.408193	3730.627758	3538.771603	3024.457229	3077.805682	3284.121231
ATN1	2765.91908	2660.455707	2669.976989	3107.492163	3237.140874	3281.148279
SASS6	385.0651607	420.282121	555.5048447	583.9522015	635.0481743	682.788029

ZNF71	15.8789757	19.96589649	13.09270678	3.610214538	1.146296344	0.990984077
PDE6D	337.4282336	288.5072042	286.1691624	185.0234951	202.8944528	237.8361785
CCDC82	434.6869598	521.1098983	660.2464989	689.5509767	746.2389196	828.4626883
CDC42SE2	936.8595663	961.3579158	1070.796376	1231.083157	1240.292644	1229.811239
HINFP	320.5568219	302.4833317	324.5120894	426.0053154	440.1777959	412.249376
PDHA1	1632.557189	1538.372324	1482.281446	1251.841891	1259.779682	1289.270284
TMEM92	51.60667102	60.89598428	85.10259405	118.2345261	118.0685234	119.9090733
ACTR5	417.8155481	388.3366866	370.3365631	282.4992876	264.7944554	306.2140798
RAB27B	163.7519369	197.6623752	258.1133622	144.4085815	111.1907453	122.8820255
SRSF9	4694.222191	4135.935457	3588.33685	3498.297887	3310.50384	2921.421059
FBXL5	468.4297831	500.145707	543.3473313	671.499904	669.4370646	629.2748888
FYTTD1	1287.189468	1457.510443	1613.208514	1788.861303	1863.877855	1815.482829

MXD4	358.2693892	276.5276663	239.4094954	457.5946926	442.4703886	373.600997
SLC1A1	255.0560472	272.534487	302.0674492	194.951585	190.285193	190.2689428
SRSF2	3090.445646	3011.855485	3069.304546	2614.697879	2490.901954	2615.206979
IFITM3	9739.76672	8226.947647	7141.136354	9952.458927	11142.00046	10768.03298
USPL1	361.2466972	399.3179297	393.7163967	508.1376962	500.9315021	517.2936881
OAZ1	9020.250633	8255.898197	7604.992251	6539.903635	7397.050305	6284.821016
SAA2	43.66718317	34.94031885	35.53734697	16.24596542	13.75555612	8.918856692
EEF1A1	126940.5015	114442.5221	112849.7805	133695.2699	148453.4006	139870.4656
SLC25A3	7326.162413	6904.207005	6465.926761	5898.188001	5963.033579	5148.16228
RAN	16958.74605	15406.68402	15081.86301	13146.59624	13779.62835	13132.52099
HSF1	2333.216992	2152.323641	1982.609883	2567.76509	2707.551963	2622.143868
HEBP1	470.4146551	417.2872365	439.5408704	320.4065402	343.8889031	321.0788409

TMEM43	912.0486667	857.535254	880.9521274	1100.21288	1092.420415	1088.100516
ITGB4	29103.18515	27955.24996	23996.12594	32491.93084	32950.28839	32435.89982
URB1	1221.688693	1430.556483	1393.438078	1128.192043	899.8426297	1099.992325
OXNAD1	224.2905318	209.6419131	223.5112086	320.4065402	279.6963078	330.9886817
CTF1	81.37975046	79.86358594	65.46353389	35.19959174	44.7055574	31.71149046
UQCRC1	6457.78093	5783.121917	5248.305031	4686.05847	4678.035378	4930.145783
MAPKBP1	781.0471172	789.651206	701.3950059	564.0960215	551.3685412	635.2207933
HS3ST2	33.74282336	23.95907578	27.1206069	7.220429075	10.31666709	5.945904461
NPTXR	145.8880892	119.7953789	113.1583943	73.10684439	64.19259524	81.26069431
DIP2C	399.9517004	408.3025831	400.26275	552.3628243	489.4685387	552.9691149
PSME3	3425.889007	3290.379741	3428.418789	3835.852946	4017.768684	4223.574136
SLC16A3	9332.867967	8338.756667	7796.706886	7494.80538	6867.461394	5737.797805

ZCCHC10	352.3147733	375.3588539	346.0215363	462.1074608	482.5907606	482.6092455
ZC3H11B	77.41000653	67.88404805	100.0656875	130.870277	154.7500064	133.7828504
PRKCG	24.81089953	21.96248613	15.8982868	40.61491355	56.16852083	57.47707646
KITLG	272.9198948	330.4355868	420.8370036	235.5664986	213.2111199	237.8361785
SENP3	478.3541429	499.1474121	500.3284376	396.2210455	381.7166824	285.4034142
PDAP1	8663.966116	7223.661348	6824.105811	6035.376153	6518.987306	5508.880484
RAB27A	214.3661719	222.6197458	237.5391087	142.6034742	155.8963027	156.5754842
VSIG10L	30.76551542	43.92497227	43.95408704	18.95362632	6.877778061	12.882793
PSMA1	651.0380037	646.8950461	630.320312	500.0147135	523.857429	467.7444843
MIS18BP1	728.4480102	909.4465849	950.1564347	657.9615995	569.7092827	700.6257424
FBXO36	133.9788575	162.7220564	129.0566811	76.71705892	82.53333673	95.13447138
ZNF630	19.84871962	16.97101201	12.15751344	0.902553634	1.146296344	3.963936308

WDR6	2342.148916	2130.361155	1957.359663	1833.086431	1742.370442	1518.187606
DLX2	7.93948785	9.982948243	7.48154673	27.07660903	27.51111224	32.70247454
PISD	1558.124491	1314.754284	1304.594711	1722.072334	1901.705634	1650.979472
TXNRD3	172.6838607	191.6726063	182.3627015	127.2600625	111.1907453	113.9631688
EPHX1	455.5281154	367.3724953	372.2069498	301.4529139	265.9407517	287.3853823
ZNF577	11.90923177	9.982948243	13.09270678	30.68682357	35.53518665	38.648379
FBXO44	232.2300196	194.6674907	177.6867348	129.9677234	134.1166722	127.8369459
CLEC2B	87.33436635	84.85506006	70.13950059	126.3575088	161.6277844	123.8730096
LAMA1	772.1151934	803.6273335	783.6920199	649.8386168	527.296318	636.2117774
CCDC71L	429.7247799	461.2122088	420.8370036	598.3930596	644.2185451	512.3387678
TPGS2	658.9774915	676.8438908	763.1177664	549.6551634	465.3963155	570.8068283
MEAK7	1914.409008	1817.894875	1815.210275	2182.374688	2440.464915	2142.507574

MED7	251.0863032	213.6350924	219.7704352	315.893772	322.1092725	323.0608091
USP12	609.3556925	751.7160027	722.9044528	879.9897935	922.7685565	882.9668125
PHPT1	1164.127406	935.4022503	886.5632875	747.3144093	818.4555893	684.7699971
IL20	42.67474719	55.90451016	49.56524708	111.014097	103.1666709	71.35085354
TMEM161B	467.4373472	629.9240341	676.1447857	787.0267692	773.7500319	853.2372902
CHERP	929.9125144	931.409071	897.7856076	661.571814	699.2407695	798.733166
BMI1	484.3087588	473.1917467	534.9305912	709.4071566	687.7778061	579.725685
DAZAP1	4319.08139	4117.96615	4000.757114	3447.754883	3624.589038	3412.949161
DPH7	835.6310962	770.6836043	759.3769931	652.5462777	600.659284	598.5543825
CRIP2	109.1679579	98.8311876	66.39872723	48.73789626	43.55926105	47.56723569
PAQR7	408.8836243	380.350328	358.1790497	298.745253	285.4277895	236.8451944
CLPTM1L	4131.51099	3731.626053	3535.966023	3065.974696	3200.459391	3166.194126

BOK	540.8776098	421.2804158	490.9765041	371.8520974	350.7666811	342.8804906
CHST7	114.1301378	107.815841	86.03778739	54.15321806	56.16852083	59.45904461
ZNF646	478.3541429	473.1917467	480.6893774	382.682741	346.1814957	348.8263951
ENDOV	306.6627182	279.5225508	241.279882	364.6316683	418.3981654	373.600997
SNX14	751.2740378	748.7211182	705.1357793	935.9481189	944.5481871	884.9487807
LRRC37B	84.3570584	98.8311876	101.0008809	148.9213497	142.1407466	168.4672931
BMPR2	434.6869598	598.9768946	560.1808114	792.442091	629.3166926	768.0126596
NOL4L	547.8246616	526.1013724	521.8378844	390.8057237	421.8370544	406.3034715
MCU	877.3134074	1047.211271	986.628975	1145.340562	1280.413016	1255.576825
B3GNT2	313.6097701	333.4304713	424.5777769	528.8964298	487.175946	468.7354684
SATB2	86.34193037	81.86017559	99.13049417	151.6290106	128.3851905	158.5574523
SLFNL1	55.57641495	47.91815156	55.17640713	12.63575088	28.65740859	23.78361785

MMP17	357.2769532	323.4475231	274.011649	446.764049	426.4222398	437.0239779
L3MBTL4	11.90923177	3.993179297	13.09270678	24.36894813	42.41296471	33.69345861
HCFC1	2768.896388	2922.008951	3008.516979	2444.115242	2236.424166	2518.090539
TMC8	64.50833878	60.89598428	55.17640713	21.66128723	28.65740859	31.71149046
SEPHS1	1259.40126	1295.786682	1176.473223	1019.885607	947.9870761	1038.551313
IKBKG	129.0166776	136.7663909	186.1034749	58.66598624	97.4351892	99.09840769
RIPPLY2	44.65961915	28.9505499	32.73176694	15.34341178	11.46296344	6.936888538
CCDC28B	174.6687327	188.6777218	189.8442483	140.798367	100.8740782	107.0262803
QDPR	202.4569402	228.6095148	188.9090549	135.3830452	144.4333393	129.8189141
FLNC	29.77307944	43.92497227	32.73176694	68.59407621	80.24074405	69.36888538
LHFPL6	225.2829677	188.6777218	224.4464019	356.5086856	292.3055676	277.4755415
KCNAB2	503.1650425	526.1013724	396.5219767	389.0006164	254.4777883	329.9976976

BRCA1	1203.824845	1403.602523	1458.901612	1041.546894	970.913003	1164.40629
NPIPB5	1078.777912	1138.0561	1123.167203	887.2102226	870.0389247	939.4529049
CASP2	689.7430069	641.903572	676.1447857	479.2559799	452.7870557	581.7076531
ZNF830	212.3813	181.689658	163.6588347	280.6941803	339.3037177	228.9173218
EPHA4	84.3570584	95.83630313	77.62104732	46.93278899	37.82777934	49.54920385
BSPRY	59.54615887	56.90280498	43.0188937	92.96302434	104.3129673	95.13447138
DNAH17	158.789757	142.7561599	170.2051881	257.2277858	187.9926003	280.4484938
GABPA	456.5205514	523.1064879	601.3293184	671.499904	738.2148452	690.7159016
MAL2	3540.019145	3783.537384	3972.701314	4433.343452	4617.281672	4401.95127
MAP4K5	962.6629018	1012.270952	1027.777482	1249.13423	1200.172272	1237.739112
ITGAV	1441.017045	1813.901696	2060.230931	2598.451913	2081.67416	2353.587183
ATP6V0E1	1674.2395	1481.469519	1443.938519	1088.479683	1340.020426	1200.081717

ZFYVE1	211.3888<br>64	245.5805<br>268	213.2240<br>818	283.4018<br>412	370.2537<br>19	312.1599<br>842
UFSP2	377.1256<br>729	376.3571<br>487	388.1052<br>366	463.0100<br>145	575.4407<br>644	495.4920<br>385
TRAF3	1124.429<br>967	1134.062<br>92	1073.601<br>956	1457.624<br>12	1295.314<br>868	1350.711<br>297
PHKA2	428.7323<br>439	456.2207<br>347	456.3743<br>505	314.9912<br>184	294.5981<br>603	373.6009<br>97
HSH2D	284.8291<br>266	307.4748<br>059	239.4094<br>954	364.6316<br>683	482.5907<br>606	354.7722<br>995
CENPN	539.8851<br>738	573.0212<br>291	509.6803<br>71	418.7848<br>864	425.2759<br>434	390.4477<br>263
SMIM11A	10.91679<br>579	50.91303<br>604	34.60215<br>363	12.63575<br>088	4.585185<br>374	3.963936<br>308
TXLNG	609.3556<br>925	685.8285<br>443	623.7739<br>586	835.7646<br>655	779.4815<br>136	821.5257<br>998
KIF7	42.67474<br>719	38.93349<br>815	57.98198<br>716	22.56384<br>086	11.46296<br>344	18.82869<br>746
DNAH14	452.5508<br>074	532.0911<br>413	497.5228<br>575	655.2539<br>386	685.4852<br>134	599.5453<br>665
PIK3CD	319.5643<br>86	334.4287<br>661	277.7524<br>223	424.2002<br>082	414.9592<br>764	423.1502<br>008
KCTD12	1024.193<br>933	1256.853<br>184	1313.011<br>451	993.7115<br>515	834.5037<br>381	931.5250<br>323

KLHDC9	22.82602757	20.96419131	28.99099358	2.707660903	6.877778061	7.927872615
ABRAXAS2	387.0500327	405.3076986	426.4481636	533.4091979	551.3685412	513.3297518
PATL2	31.7579514	44.92326709	54.24121379	11.73319725	5.731481718	24.77460192
DIPK1B	238.1846355	199.6589649	204.8073417	150.7264569	138.7018576	138.7377708
DCTN1	2898.905501	2869.099325	2608.254229	2441.407581	2213.498239	2266.380584
PLCG1	2275.655705	2187.26396	2056.490157	1785.251089	1522.281544	1904.671396
RIPK1	562.7112013	554.0536275	606.0052851	423.2976545	451.6407593	449.9067709
TMEM161A	710.5841625	658.874584	624.7091519	538.8245197	515.8333546	484.5912136
NSMAF	849.5251999	837.5693576	895.9152209	680.5254403	693.5092878	687.7429494
NKX3-1	122.0696257	109.8124307	124.3807144	66.78896895	63.04629889	78.28774208
EIF2S3	4282.361259	4272.701848	4647.910906	5131.017412	5514.831709	5060.955681
CDC26	305.6702822	243.5839371	280.5580024	183.2183878	204.0407491	187.2959905

ADAM28	120.0847537	159.7271719	129.9918744	90.25536344	65.33889158	84.23364654
SETMAR	96.26629018	108.8141358	134.6678411	62.27620077	72.21666964	57.47707646
EP400	2701.410741	3154.611645	3131.9625	3791.627818	3341.453841	4003.575671
MTRF1	309.6400261	322.4492282	332.9288295	394.4159382	431.0074252	488.5551499
HSD17B1	63.5159028	57.90109981	73.88027396	116.4294188	107.7518563	113.9631688
TRAF3IP2	1016.254445	931.409071	950.1564347	742.8016411	825.3333673	736.3011691
MBNL1	5288.691344	6647.645235	7014.885253	8095.906101	7405.074379	8361.923641
PIKFYVE	535.9154299	656.8779944	740.6731263	863.7438281	779.4815136	957.2906183
TMEM258	1066.86868	888.4823936	745.349093	638.1054195	731.3370672	630.2658729
LCP1	11491.41623	11801.84141	11211.09777	12891.17356	13792.23761	13517.02281
RIOK1	711.5765985	643.9001616	643.4130188	834.8621118	833.3574417	852.2463061
CASKIN1	7.93948785	9.982948243	9.351933412	32.49193084	29.80370493	26.75657008

PKD1	1142.293814	1339.711654	1204.529023	1799.691947	1305.631535	1738.186071
ALDH4A1	205.4342481	209.6419131	171.1403814	132.6753843	138.7018576	106.0352962
PTPRR	108.175522	124.786853	115.028781	67.69152258	71.0703733	65.40494908
RDH14	163.7519369	145.7510443	161.788448	240.0792668	231.5518614	219.007481
PPP1R3G	164.7443729	163.7203512	148.6957413	222.0281941	293.4518639	209.0976402
ZNF70	139.9334734	134.7698013	158.0476747	99.28089978	85.97222576	79.27872615
PDGFC	187.5704004	240.5890526	246.8910421	310.4784502	349.6203848	308.1960479
SYDE2	87.33436635	84.85506006	79.491434	135.3830452	124.9463014	151.6205638
CDADC1	121.0771897	178.6947735	124.3807144	231.0537304	224.6740833	203.1517358
NDN	13.89410374	17.96930684	20.57425351	0	5.731481718	1.981968154
RGL3	118.0998818	74.87211182	86.97298073	162.4596542	149.0185247	151.6205638
PSMB3	3172.817832	2779.252791	2438.049041	2132.734238	2260.496389	2298.092074

JAZF1	72.44782663	84.85506006	78.55624066	112.8192043	136.4092649	146.6656434
STT3A	2623.008298	2668.442065	2535.309148	2946.837616	3263.50569	3134.482635
PARVA	682.7959551	743.7296441	763.1177664	921.5072607	911.3055931	910.7143667
RARG	1190.923177	1109.10555	1171.797257	938.6557798	960.5963359	922.6061756
SPTBN5	14.88653972	18.96760166	12.15751344	39.71235991	43.55926105	38.648379
CD46	3907.220458	4674.016367	4692.800186	5446.911184	5290.157625	5390.953378
SLC25A29	330.4811817	305.4782162	311.4193826	257.2277858	183.407415	215.0435447
ATRAID	950.75367	895.4704574	797.7199201	648.9360631	748.5315123	656.0314589
EPOP	90.31167429	67.88404805	72.94508062	151.6290106	131.8240795	110.9902166
ASL	469.4222191	466.2036829	373.1421431	333.9448447	312.9389018	305.2230957
AFG3L2	1055.951884	1088.141358	1078.277922	931.4353507	816.1629966	833.4176087
STK10	1618.663085	1551.350157	1428.975425	1795.179179	1971.629711	1854.131208

ZIC5	83.36462242	109.8124307	97.26010749	51.44555716	55.02222449	53.51314015
ZCCHC14	772.1151934	930.4107762	916.4894744	1073.136271	1062.61671	1206.027622
TMEM59	1879.673748	1744.021058	1631.91238	1377.296846	1546.353767	1280.351427
GXYLT1	400.9441364	440.2480175	488.1709241	611.9313641	535.3203924	615.4011118
FDXACB1	35.72769532	39.93179297	21.50944685	10.83064361	10.31666709	8.918856692
C19orf57	41.68231121	25.95566543	23.37983353	6.317875441	11.46296344	8.918856692
PRRT1	56.56885093	52.90962569	32.73176694	12.63575088	26.3648159	14.86476115
SCAPER	176.6536047	163.7203512	189.8442483	220.2230868	269.3796407	295.3132549
ELOVL4	136.9561654	155.7339926	189.8442483	247.2996958	286.5740859	207.1156721
DLK2	311.6248981	259.5566543	252.5022021	172.3877442	209.7722309	184.3230383
AC106886.5	42.67474719	25.95566543	44.88928038	66.78896895	85.97222576	79.27872615
ZNF622	1079.770348	959.3613261	964.1843348	1176.929939	1358.361167	1229.811239

CD177	71.45539065	81.86017559	50.50044043	30.68682357	35.53518665	29.72952231
ANKIB1	1976.932475	2468.7831	2525.022021	2996.478066	2712.137149	3070.06867
IFT46	113.1377019	77.86699629	106.6120409	138.9932597	165.0666735	186.3050065
FOPNL	830.6689163	783.661437	913.6838944	626.3722223	709.5574366	641.1666978
ING5	664.9321074	737.7398751	737.8675462	604.7109351	535.3203924	509.3658155
PLEKHA7	239.1770715	249.5737061	230.9927553	312.2835575	328.9870506	354.7722995
BAHCC1	240.1695075	211.6385027	214.1592751	166.0698687	135.2629685	144.6836752
GSDME	1252.454208	1258.849773	1212.945764	972.0502643	1028.22782	1041.524265
HSDL1	482.3238869	565.0348705	521.8378844	637.2028659	710.703733	685.7609812
MT-ND6	11157.95774	8548.39858	7029.848346	4406.266843	5929.790985	5088.703235
SMAD5	1013.277137	1211.929917	1279.344491	1632.719525	1417.968577	1423.053134
CPT1A	2057.319789	1920.719242	1874.127456	1634.524632	1564.694509	1647.015536

SUFU	306.6627182	298.4901525	273.0764556	221.1256404	173.0907479	216.0345288
AC129492.1	630.1968481	643.9001616	575.1439049	774.3910183	801.2611441	767.0216755
ERI2	309.6400261	392.3298659	435.800097	302.3554675	230.405565	249.7279874
DDX31	531.9456859	577.0144084	555.5048447	677.8177794	696.9481769	752.1569144
TAF4B	145.8880892	169.7101201	230.0575619	283.4018412	265.9407517	273.5116052
ATP6V1C1	1239.552541	1403.602523	1362.576698	1509.97223	1773.320443	1696.56474
NENF	777.0773733	579.0109981	581.6902582	466.620229	490.614835	474.6813728
CSF2RA	14.88653972	9.982948243	10.28712675	31.5893772	37.82777934	33.69345861
STK35	766.1605775	812.6119869	759.3769931	1014.470285	982.3759664	920.6242075
ETV4	664.9321074	674.8473012	624.7091519	906.1638489	857.429665	752.1569144
ESCO2	246.1241233	252.5685905	339.4751829	213.0026577	163.9203771	179.3681179
SLC39A10	733.4101901	908.4482901	941.7396946	652.5462777	615.5611365	703.5986946

ELMO2	622.2573602	603.9683687	547.0881046	436.8359591	455.0796484	471.7084206
NOC4L	963.6553378	832.5778834	835.1276537	614.639025	707.2648439	722.4273921
GJB7	1.984871962	0.998294824	1.870386682	18.05107269	12.60925978	11.89180892
MFNG	107.183086	129.7783272	105.6768476	202.1720141	173.0907479	160.5394205
HIRA	319.564386	315.4611645	272.1412623	227.4435159	196.0166747	216.0345288
DNA2	425.7550359	450.2309657	437.6704837	330.3346302	278.5500115	356.7542677
RCHY1	214.3661719	233.6009889	257.1781688	331.2371838	387.4481641	290.3583345
HIST1H3E	68.4780827	70.87893252	59.85237384	100.1834534	130.6777832	113.9631688
RBL2	1201.839973	1425.565009	1547.74498	1123.679275	984.6685591	1141.613657
SPTAN1	4124.563938	4280.688206	4276.639149	4830.467051	4968.048353	5024.28927
ABCB1	10.91679579	24.95737061	15.8982868	50.54300353	44.7055574	39.63936308
TRIM9	59.54615887	62.89257393	69.20430725	124.5524015	111.1907453	97.11643954

DEPDC1B	720.5085224	835.5727679	791.1735667	634.495205	627.0240999	572.7887965
MOB1A	2068.236585	2206.231562	2411.863627	1928.757117	1790.514889	1744.131975
NIN	1031.140984	1244.873646	1445.808906	987.393676	903.2815187	951.3447138
MOSPD1	106.19065	117.7987893	115.9639743	174.1928514	196.0166747	157.5664682
ECPAS	3272.06143	3314.338817	3505.104643	3983.871742	3896.261272	3996.638782
HERC2	1865.779645	2183.270781	2231.371312	1719.364674	1464.966727	1791.699211
CCN4	9.924359812	11.97953789	4.675966706	22.56384086	33.24259396	34.68444269
NEU3	570.6506892	634.9155082	591.0421917	473.8406581	448.2018703	467.7444843
DNAJB14	567.6733813	689.8217236	807.0718535	923.312368	895.2574443	913.6873189
CCAR1	1668.284884	1863.816437	1913.405576	2152.590418	2151.598237	2325.839629
NHSL1	367.201313	312.46628	337.6047962	468.4253363	442.4703886	445.9428346
ZNF653	94.28141822	77.86699629	59.85237384	146.2136888	136.4092649	114.9541529

CCDC178	1.984871962	0	0.935193341	9.928089978	16.04814881	12.882793
SNRNP40	1289.17434	1202.945263	1169.92687	1046.962216	968.6204103	940.443889
CHRNA5	61.53103084	64.88916358	73.88027396	44.22512809	24.07222321	21.80164969
LGMN	899.146999	790.6495008	814.5534002	609.2237032	657.9741012	697.6527901
ZNF503	108.175522	90.84482901	101.9360742	64.08130804	50.43703911	53.51314015
INSIG1	498.2028626	468.2002726	442.3464504	601.1007205	656.8278048	574.7707646
LOXL3	79.3948785	85.85335489	90.7137541	38.80980628	36.68148299	57.47707646
GABRA3	64.50833878	77.86699629	74.8154673	116.4294188	115.7759307	132.7918663
PSPC1	1481.70692	1452.518969	1496.309346	1765.394909	1844.390817	1719.357373
IGSF1	35.72769532	40.93008779	38.34292699	67.69152258	74.50926233	82.25167838
EDC3	636.151464	659.8728788	615.3572185	842.0825409	795.5296624	769.9946278
SNX30	840.5932761	974.3357485	914.6190877	1182.345261	1077.518563	1144.586609

UBE2O	1520.411923	1499.438826	1368.187858	1214.837192	1173.807456	1205.036638
NDUFA1	1632.557189	1353.687782	1195.17709	814.1033782	876.9167028	504.4108951
SS18	883.2680233	851.5454851	1001.592068	688.6484231	724.4592891	746.2110099
EMC4	1050.989704	914.438059	952.0268214	749.1195166	842.5278125	691.7068857
KATNA1	322.5416939	310.4696903	307.6786093	419.68744	441.3240923	396.3936308
BBIP1	125.0469336	121.7919686	133.7326478	166.9724224	207.4796382	207.1156721
TNRC6C	153.8275771	199.6589649	162.7236414	264.4482149	251.0388992	238.8271625
PRMT2	1253.446644	1115.095319	1023.101515	881.7949008	923.9148529	885.9397648
SLC4A11	105.198214	96.83459795	82.29701403	46.03023535	56.16852083	54.50412423
ZNF182	88.32680233	121.7919686	111.2880076	149.8239033	183.407415	178.3771338
NME2	93.28898223	81.86017559	82.29701403	51.44555716	51.58333546	28.73853823
ZSWIM4	152.8351411	152.7391081	130.9270678	214.807765	220.088898	209.0976402

USB1	1288.181904	1123.081677	1111.944883	938.6557798	931.9389273	959.2725865
AKAP11	1144.278686	1438.542842	1614.143707	1878.214113	1695.372292	1866.023017
HIST1H2BF	4.962179906	8.984653418	14.02790012	25.27150176	40.12037202	28.73853823
MRPS31	511.1045303	449.2326709	408.6794901	592.9777378	594.9278023	606.4822551
GTSF1	1296.121391	1247.86853	1243.807144	1373.686632	1722.883404	1658.907345
USH1G	75.42513457	63.89086875	57.04679381	26.1740554	42.41296471	22.79263377
PCMTD2	925.9427705	1013.269247	1110.074496	1261.769981	1252.901903	1275.396507
ABCF2	616.3027443	466.2036829	533.9953978	419.68744	404.6426093	373.600997
DHX34	600.4237686	616.9462014	569.5327448	787.0267692	691.2166951	801.7061182
PSMB1	3510.246066	3234.475231	3063.693386	2754.593692	2795.816782	2623.134852
NME6	434.6869598	388.3366866	423.6425836	506.3325889	562.8315047	563.8699398
ZNF774	130.0091135	116.8004944	131.8622611	203.9771214	191.4314894	176.3951657

PLCB1	237.1921995	281.5191404	291.7803225	361.9240074	384.0092751	366.6641085
DDX39B	1919.371188	1985.608405	1934.915023	2360.177754	2215.790832	2381.334737
NDRG3	1679.20168	1671.145536	1628.171607	1360.148327	1326.264869	1439.899864
ST6GALNAC6	16.87141168	15.97271719	14.02790012	2.707660903	2.292592687	1.981968154
MOSPD2	227.2678397	264.5481284	235.668722	323.1142011	327.8407542	352.7903314
SLC47A1	160.774629	170.7084149	150.5661279	250.0073567	209.7722309	242.7910988
RPN1	3746.445829	3394.202402	3332.093875	3971.235991	4502.652037	4135.376553
VIPAS39	236.1997635	237.5941682	215.0944685	324.9193084	302.6222347	323.0608091
SCAF11	1475.752304	1764.985249	1755.357901	2124.611255	1936.094524	2113.769036
COQ6	112.1452659	123.7885582	122.5103277	81.2298271	71.0703733	58.46806054
BRD3	807.8428887	777.6716681	709.811746	655.2539386	545.6370595	575.7617487
LIN7C	764.1757055	838.5676524	960.4435614	1026.203482	1098.151897	1140.622673

DTNB	270.9350229	293.4986783	263.7245222	371.8520974	364.5222372	381.5288696
NEK4	378.1181088	378.3537384	406.8091034	494.5993917	475.7129826	568.8248601
NFYB	340.4055416	353.3963678	389.9756233	490.0866235	442.4703886	506.3928633
SPTBN2	1470.790124	1411.588881	1437.392165	1209.42187	1097.005601	1230.802224
PPIA	37390.02559	33183.31996	31759.16587	26334.70994	30762.00867	27480.97944
CEBPZ	787.0017331	962.3562106	954.8324014	1113.751185	1094.713008	1225.847303
CEBPD	997.3981611	992.3050553	924.9062145	1324.046182	1342.313018	1054.407058
GFM2	1039.080472	1145.044163	1143.741456	1341.194701	1329.703758	1395.30558
DOK4	161.7670649	160.7254667	166.4644147	113.7217579	110.044449	89.18856692
CLTB	2552.545344	2099.414015	1856.358782	1635.427186	1846.683409	1558.817953
ACP6	416.8231121	410.2991728	466.6614773	304.1605748	324.4018652	338.9165543
LEPROTL1	564.6960733	613.9513169	611.6164452	747.3144093	769.1648465	740.2651055

CLIC1	8175.687613	6942.142208	6279.823286	5341.312408	6032.957656	5853.742942
KDM3B	1898.530032	2001.581123	1919.95193	2466.679083	2220.376017	2288.182234
ARNT2	243.1468154	244.5822319	220.7056285	331.2371838	311.7926054	329.9976976
BZW1	6117.375388	6653.635004	6786.698077	7691.562072	8421.839236	7284.723949
RTL6	283.8366906	323.4475231	288.0395491	411.5644573	388.5944605	401.3485511
CCT2	4723.995271	4474.357402	4540.363672	3752.818012	4089.985354	3826.189521
PRELID2	191.5401444	169.7101201	181.4275082	222.9307477	317.5240872	261.6197963
WDR7	95.2738542	85.85335489	100.0656875	153.4341178	155.8963027	136.7558026
WDR12	1152.218174	1210.931622	1224.168084	972.9528179	904.427815	1029.632456
PIGZ	23.81846355	24.95737061	19.63906017	7.220429075	3.438889031	5.945904461
RHOBTB2	1420.175889	1224.907749	1212.01057	1560.515234	1627.740808	1558.817953
MCTP2	750.2816018	798.6358594	777.1456666	615.5415787	610.9759511	629.2748888

ABHD6	223.2980958	258.5583595	226.3167886	165.1673151	147.8722283	172.4312294
TUBB2B	14.88653972	15.97271719	12.15751344	41.51746718	40.12037202	32.70247454
MUL1	727.4555742	560.0433964	544.2825246	446.764049	497.4926131	396.3936308
HERC1	682.7959551	828.5847041	788.3679867	991.9064442	881.5018882	1094.046421
RGS17	105.198214	90.84482901	137.4734212	226.5409622	158.1888954	161.5304045
MCPH1	363.2315691	411.2974676	392.7812033	486.4764089	490.614835	565.8519079
DTNA	201.4645042	176.6981839	202.0017617	274.3763049	267.087048	276.4845575
SELENOS	767.1530135	680.8370701	670.5336257	866.451489	1039.690784	833.4176087
C7orf61	14.88653972	6.98806377	14.02790012	33.39448447	42.41296471	29.72952231
GNS	2338.179172	2511.709778	2583.939202	2940.519741	3013.613087	2880.790712
NPHP4	496.2179906	448.2343761	454.5039638	379.9750801	299.1833457	357.7452518
PSMA5	2429.483282	2062.477107	2066.777284	1838.501753	1784.783407	1737.195087

RAB3D	510.1120943	476.1866312	498.4580509	365.5342219	386.3018678	387.4747741
CYTH1	1235.582797	1230.897518	1106.333723	1486.505836	1499.355617	1386.386724
GIPC3	7.93948785	0.998294824	2.805580024	19.85617996	26.3648159	14.86476115
PDRG1	758.2210896	643.9001616	600.3941251	952.1940843	982.3759664	739.2741214
DDX46	2304.436348	2467.784806	2730.764556	2156.200633	1818.026001	2088.994434
EIF3CL	122.0696257	101.8260721	105.6768476	73.10684439	50.43703911	64.413965
TNFRSF1A	2436.430334	2135.352629	2150.944685	2684.194509	2747.672335	2647.909454
QTRT1	794.941221	693.8149029	637.8018587	532.5066443	572.0018754	536.1223856
MAD1L1	29.77307944	24.95737061	25.25022021	49.64044989	64.19259524	58.46806054
SLC52A2	921.9730265	747.7228234	767.7937331	707.6020494	588.0500242	529.1854971
AKAP6	49.62179906	73.87381699	57.98198716	111.9166507	85.97222576	127.8369459
B3GNT3	971.5948256	946.3834934	860.3778739	759.0476065	754.262994	707.5626309

SENP5	757.2286537	752.7142975	826.7109136	968.4400497	960.5963359	965.2184909
MED8	707.6068546	567.0314602	525.5786578	752.7297311	922.7685565	743.2380577
RNASEH2A	47.6369271	47.91815156	46.75966706	16.24596542	19.48703784	24.77460192
PRDM16	46.64449112	40.93008779	28.99099358	71.30173712	74.50926233	82.25167838
EIF2AK3	230.2451476	249.5737061	277.7524223	347.4831492	340.450014	349.8173791
ZSCAN21	134.9712934	142.7561599	151.5013213	191.3413705	220.088898	222.9714173
FAHD2B	377.1256729	325.4441127	306.7434159	203.9771214	276.2574188	230.8992899
PSENEN	125.0469336	112.8073151	90.7137541	75.81450529	61.90000255	37.65739492
ZNF14	44.65961915	30.94713955	28.05580024	63.17875441	79.0944477	68.37790131
CCDC190	5.954615887	20.96419131	11.22232009	0.902553634	1.146296344	0
OSBPL10	612.3330004	655.8796995	565.7919714	774.3910183	805.8463295	744.2290418
WDR24	267.9577149	244.5822319	202.936955	137.1881524	163.9203771	178.3771338

POFUT2	565.6885093	546.0672689	539.6065579	686.8433158	711.8500293	683.7790131
SRP19	216.3510439	253.5668854	226.3167886	315.893772	341.5963104	306.2140798
KLC2	1967.008115	1883.782333	1837.654916	2374.618612	2216.937128	2200.975635
SMIM12	640.1212079	631.9206238	564.8567781	489.1840698	497.4926131	424.1411849
DAGLA	96.26629018	78.86529112	101.9360742	151.6290106	136.4092649	153.6025319
DAZAP2	3030.899487	3029.824792	3047.795099	2433.284598	2702.966778	2557.729903
MAT2B	1130.384583	1198.952084	1129.713556	1386.322382	1487.892654	1341.79244
ARAP1	3786.143268	3684.706196	3336.769841	4322.329355	4211.492766	4272.132356
TMEM123	5178.53095	5986.774061	6066.599205	4719.452954	4883.222423	4816.182614
GK5	665.9245434	690.8200184	801.4606934	554.1679315	560.538912	567.8338761
SRR	65.50077476	73.87381699	74.8154673	28.8817163	48.14444643	29.72952231
MRFAP1	4585.054233	4181.857019	4076.507774	3473.928939	3871.042752	3243.490884

ATP7B	91.30411027	108.8141358	84.16740071	149.8239033	146.725932	152.6115478
TRIR	2352.073275	2078.449824	1950.81331	1507.264569	1949.85008	1539.989256
ZBED4	687.758135	823.59323	735.9971595	1002.737088	874.6241101	972.1553795
FBXO33	159.782193	188.6777218	192.6498283	222.0281941	271.6722334	300.2681753
PAK3	28.78064346	27.95225508	28.99099358	65.88641531	49.29074277	64.413965
TPRA1	563.7036373	458.2173243	498.4580509	700.3816203	718.7278074	586.6625735
BCKDK	1464.835508	1262.842953	1114.750463	972.9528179	1011.033375	1036.569344
ZNF485	42.67474719	45.92156192	43.0188937	21.66128723	19.48703784	11.89180892
INTS7	794.941221	892.4755729	858.5074872	1046.059662	1001.863004	1111.884134
RCC1L	527.975942	472.1934519	426.4481636	374.5597583	369.1074226	313.1509683
MINDY3	241.1619434	291.5020887	297.3914825	399.83126	377.131497	357.7452518
MTMR3	134.9712934	143.7544547	115.9639743	203.9771214	220.088898	173.4222135

GFI1	99.24359812	97.83289278	79.491434	47.83534262	60.75370621	43.60329938
COX10	340.4055416	317.4577541	377.8181099	483.768748	432.1537215	460.8075958
FAAH2	34.73525934	31.94543438	27.1206069	64.98386168	53.87592815	73.33282169
CENPP	198.4871962	231.6043992	237.5391087	166.9724224	132.9703758	150.6295797
ERO1A	2905.852553	3248.451358	2865.432398	3601.189001	3957.014978	3382.228654
POLR2H	2364.974943	1864.814732	1759.098675	1518.095213	1627.740808	1562.781889
DENND3	775.0925013	771.6818992	822.0349469	616.4441323	647.6574341	632.2478411
LUM	101.2284701	78.86529112	93.51933412	38.80980628	53.87592815	56.48609238
URGCP	838.6084041	742.7313492	653.7001455	905.2612953	1032.813006	935.4889686
OSCP1	122.0696257	96.83459795	85.10259405	62.27620077	48.14444643	58.46806054
EVA1B	154.8200131	140.7595702	115.028781	92.06047071	87.11852211	68.37790131
MTHFD2L	222.3056598	259.5566543	277.7524223	176.9005123	193.7240821	150.6295797

TTC9C	341.3979775	291.5020887	324.5120894	406.1491355	435.5926105	435.0420098
RNF187	3691.86185	3616.822148	3270.371114	4290.739978	4750.252048	3877.720693
EPB41L5	296.7383584	362.3810212	387.1700433	458.4972463	441.3240923	520.2666404
ENTR1	2492.999185	2430.847897	2334.24258	1946.808189	2148.159348	1984.941106
ZC3H12A	1566.063978	1478.474635	1525.30034	1225.667836	1302.192646	1267.468634
TTC8	331.4736177	347.4065988	354.4382763	214.807765	254.4777883	278.4665256
SLC2A13	200.4720682	212.6367976	220.7056285	158.8494397	116.922227	147.6566275
FRAS1	700.6598027	842.5608317	839.8036204	1134.509918	852.8444796	1163.415306
CFDP1	559.7338934	509.1303604	466.6614773	647.1309559	849.4055905	593.5994621
TSSC4	604.3935126	494.155938	377.8181099	687.7458694	679.7537317	663.9593315
C1QTNF1	3.969743925	5.989768946	7.48154673	9.928089978	37.82777934	27.74755415
HTATIP2	1682.178988	1631.213743	1597.310227	1283.431268	1462.674134	1296.207173

MYO19	2563.462139	2593.569953	2610.124615	2164.323615	1934.948228	2322.866676
THBD	620.2724883	703.7978511	636.8666654	800.5650737	974.351892	767.0216755
CENPS	54.58397897	25.95566543	35.53734697	18.05107269	13.75555612	7.927872615
SP8	58.55372289	49.91474121	46.75966706	17.14851905	19.48703784	30.72050638
PDSS1	300.7081023	293.4986783	317.0305427	232.8588377	204.0407491	221.9804332
PCNP	1372.538962	1479.47293	1678.672048	1765.394909	1969.337118	1865.032033
TMC4	393.0046486	327.4407024	249.6966221	454.8870317	502.0777985	419.1862645
SIX5	265.972843	236.5958733	200.131375	155.2392251	175.3833406	145.6746593
ACTR3	3789.120576	3969.220221	4123.267441	4579.557141	4778.909456	4566.454626
TRAPPC3	1064.883808	911.4431745	812.6830135	695.8688521	784.066699	666.9322838
BAIAP2L2	652.0304397	553.0553326	540.5417512	445.8614954	473.4203899	411.2583919
TTLL3	141.9183453	169.7101201	148.6957413	100.1834534	90.55741114	103.062344

ZDHHC2	305.6702822	335.427061	358.1790497	432.3231909	426.4222398	467.7444843
MIPEP	418.8079841	386.340097	406.8091034	324.9193084	294.5981603	288.3763664
KRT31	15.8789757	26.95396025	18.70386682	5.415321806	3.438889031	4.954920385
MYO1C	4277.399079	4180.858724	3924.07126	4822.344069	5165.211324	4651.679257
SLAIN1	24.81089953	55.90451016	33.66696028	90.25536344	52.7296318	103.062344
ZNF780A	141.9183453	189.6760166	187.0386682	250.9099104	243.0148248	258.6468441
MAP3K13	611.3405644	694.8131977	663.0520789	802.370181	892.9648516	792.7872615
NPW	99.24359812	81.86017559	76.68585398	44.22512809	52.7296318	43.60329938
SERPINA1	29.77307944	21.96248613	28.99099358	50.54300353	73.36296599	51.531172
HSPA2	20.84115561	28.9505499	43.95408704	13.53830452	8.024074405	6.936888538
GCSH	137.9486014	148.7459288	216.9648552	90.25536344	93.99630017	120.9000574
STAMBP	1268.333184	1243.875351	1318.622611	1030.71625	1063.763007	1069.271819

WRAP73	866.3966116	814.6085766	757.5066064	657.0590458	606.3907657	668.9142519
FGF19	0.992435981	3.993179297	1.870386682	15.34341178	22.92592687	8.918856692
MAD2L1BP	517.0591462	470.1968622	418.0314235	525.2862152	683.1926207	682.788029
DHX58	212.3813	212.6367976	212.2888885	132.6753843	155.8963027	149.6385956
H2AFJ	935.8671303	873.5079712	724.7748395	697.6739594	674.02225	469.7264525
EBP	626.2271041	462.2105036	474.143024	365.5342219	405.7889056	377.5649333
PPP6R2	1305.053315	1308.764515	1241.936757	1107.433309	962.8889286	1070.262803
SPART	452.5508074	482.1764001	516.2267244	642.6181877	638.4870633	582.6986372
XPO4	719.5160864	883.4909195	910.8783144	1119.166507	978.9370774	1116.839055
WHRN	91.30411027	124.786853	87.90817408	157.0443324	158.1888954	164.5033568
ZNF18	113.1377019	90.84482901	86.97298073	40.61491355	61.90000255	58.46806054
CXXC4	44.65961915	60.89598428	39.27812033	18.05107269	14.90185247	26.75657008

C11orf94	2.977307944	3.993179297	0.935193341	20.75873359	11.46296344	15.85574523
ARHGAP33	99.24359812	91.84312383	82.29701403	64.08130804	32.09629762	46.57625161
BMP2K	137.9486014	151.7408133	172.0755748	238.2741595	200.6018601	237.8361785
CLN5	121.0771897	115.8021996	152.4365146	183.2183878	207.4796382	197.2058313
SIDT2	435.6793958	401.3145194	404.9387168	310.4784502	291.1592713	331.9796658
PAIP2	1315.970111	1274.822491	1172.73245	1528.023303	1667.86118	1429.990023
DOP1B	182.6082205	206.6470286	190.7794416	244.5920349	273.9648261	304.2321116
ORC3	481.3314509	559.0451016	521.8378844	379.0725265	424.1296471	394.4116626
TSPAN10	81.37975046	51.91133086	68.26911391	33.39448447	29.80370493	35.67542677
DMAC2L	308.6475902	363.379316	371.2717565	262.6431076	271.6722334	217.0255128
AC124319.1	2926.693709	3362.256968	3494.817516	4086.762857	3543.201998	4604.112021
LTO1	593.4767168	575.0178188	648.0889855	711.2122639	832.2111454	769.0036437

ZNF581	655.0077476	546.0672689	435.800097	797.8574128	952.5722615	596.5724143
ECHS1	1668.284884	1582.297296	1389.697305	1294.261912	1296.461165	1147.559561
SLC25A24	901.1318709	970.3425692	1111.944883	1272.600625	1244.877829	1202.063685
DOCK6	988.4662373	922.4244176	898.7208009	1065.915842	1165.783381	1259.540762
ZNF543	158.789757	137.7646857	185.1682816	223.8333013	254.4777883	227.9263377
MOCS3	581.567485	537.0826155	553.634458	416.9797791	480.2981679	380.5378855
PLAUR	3216.485015	2903.041349	2757.885163	2242.845782	2635.335294	2407.100323
ZNF383	22.82602757	33.94202402	31.7965736	55.0557717	68.77778061	60.45002869
HIST1H1D	13.89410374	16.97101201	16.83348014	38.80980628	48.14444643	33.69345861
CHST10	235.2073275	258.5583595	243.1502687	166.9724224	173.0907479	180.359102
RHOT1	694.7051869	662.8677633	692.9782658	846.5953091	835.6500344	853.2372902
LEMD2	1008.314957	962.3562106	821.0997536	770.7808038	724.4592891	706.5716468

UCN2	241.1619434	243.5839371	275.8820357	183.2183878	181.1148223	173.4222135
NTF4	68.4780827	70.87893252	43.95408704	32.49193084	30.95000127	20.81066562
C9orf85	248.1089953	277.5259611	228.1871753	310.4784502	418.3981654	333.9616339
KDELC2	278.8745107	256.5617698	298.3266759	206.6847823	200.6018601	189.2779587
PRR12	984.4964934	1084.148179	986.628975	886.307669	771.4574392	790.8052934
ST7L	212.3813	206.6470286	204.8073417	277.0839658	272.8185298	316.1239205
IQCH	102.2209061	87.84994453	108.4824276	153.4341178	154.7500064	154.593516
PTPRK	1292.151648	1467.493392	1363.511892	1752.759158	1522.281544	1800.618068
UBA52	7615.95372	6398.071529	5450.306793	4732.991259	5412.811334	5272.035289
SNRPN	39.69743925	35.93861367	28.05580024	66.78896895	75.65555867	62.43199685
PAM	1838.983873	1825.881234	2012.53607	2148.980204	2259.350093	2438.811813
BHLHA15	6.947051869	4.991474121	8.416740071	20.75873359	35.53518665	18.82869746

C8orf58	165.7368089	189.6760166	147.7605479	253.6175713	257.9166773	218.0164969
SYT17	121.0771897	97.83289278	103.8064609	72.20429075	63.04629889	51.531172
EBI3	9.924359812	16.97101201	10.28712675	45.12768172	30.95000127	28.73853823
ASAH1	693.7127509	610.9564324	540.5417512	463.0100145	478.0055752	472.6994047
TMEM52	61.53103084	45.92156192	53.30602045	30.68682357	20.63333418	20.81066562
DYNLL1	2706.372921	2318.040582	2254.751146	1890.849864	2020.920454	2055.300976
LRRC2	2.977307944	10.98124307	4.675966706	21.66128723	25.21851956	24.77460192
IGF1R	2355.050583	2482.759228	2568.040915	2906.222703	2837.08345	2998.717817
STAMBPL1	157.797321	188.6777218	187.0386682	237.3716058	255.6240846	266.5747167
HHEX	84.3570584	106.8175462	115.9639743	48.73789626	64.19259524	62.43199685
GDA	122.0696257	162.7220564	132.7974545	195.8541387	253.3314919	187.2959905
ZNF57	96.26629018	83.85676524	87.90817408	51.44555716	49.29074277	50.54018792

CSNK2B	356.2845173	302.4833317	259.9837489	219.3205332	220.088898	210.0886243
SMC4	5412.745842	6606.715147	7231.850108	7839.580868	7481.876234	8653.27296
GCN1	6722.761337	6505.88737	6613.687309	5898.188001	5064.337246	5782.392089
NR2F6	1519.419487	1363.67073	1285.890844	1214.837192	1080.957452	1060.352962
RPS27L	320.5568219	283.5157301	257.1781688	187.731156	229.2592687	191.2599268
MAP2K4	437.6642677	505.1371811	535.8657845	633.5926514	623.5852109	645.1306341
ZER1	842.5781481	804.6256284	714.4877127	910.6766171	999.5704115	1066.298867
MAK16	448.5810635	495.1542328	506.8747909	595.6853987	600.659284	661.9773634
OIP5	201.4645042	248.5754112	231.9279486	153.4341178	165.0666735	153.6025319
NFX1	1525.374103	1573.312643	1620.69006	1787.95875	1885.657485	1978.995202
TMEM259	2998.149099	2669.44036	2555.883402	2361.982861	2283.422316	2179.173985
TTC30A	54.58397897	51.91133086	67.33392057	76.71705892	124.9463014	111.9812007

MFSD5	393.9970845	394.3264556	281.4931957	245.4945886	283.1351968	217.0255128
ACYP1	247.1165593	190.6743114	179.5571215	122.7472943	155.8963027	127.8369459
KDELR3	146.8805252	175.6998891	157.1124813	80.32727346	119.2148197	105.0443122
DGKH	1146.263558	1587.288771	1596.375033	1969.37203	1671.300069	1996.832915
ZNF614	117.1074458	132.7732116	141.2141945	202.1720141	166.2129698	219.007481
GHDC	132.9864215	111.8090203	105.6768476	179.6081732	225.8203797	146.6656434
HSBP1L1	252.0787392	198.66067	220.7056285	312.2835575	337.011125	287.3853823
CACUL1	1009.307393	1055.197629	1081.083502	1229.27805	1291.875979	1286.297332
ZNF174	159.782193	155.7339926	170.2051881	100.1834534	116.922227	102.0713599
ZC3H8	207.4191201	218.6265665	221.6408219	313.1861111	283.1351968	292.3403027
SELE	0	0.998294824	0	5.415321806	12.60925978	9.909840769
ASAH2B	112.1452659	81.86017559	127.1862944	203.9771214	170.7981552	142.7017071

ITGB3	7.93948785	1.996589649	4.675966706	20.75873359	29.80370493	13.87377708
AMMECR1	1083.740091	1350.692897	1358.835925	1619.18122	1515.403766	1591.520428
PCYT1A	791.963913	825.5898197	740.6731263	945.8762089	977.790781	979.092268
LPCAT3	520.0364542	480.1798105	488.1709241	361.0214538	363.3759409	416.2133123
TMEM87B	434.6869598	436.2548382	467.5966706	550.557717	620.1463218	550.9871468
UMPS	946.7839261	860.5301385	938.9341146	782.514001	712.9963257	714.4995195
BOP1	3105.332185	2646.479579	2480.132741	2296.096446	2310.933429	2081.066562
TWF2	728.4480102	604.9666635	525.5786578	479.2559799	497.4926131	397.3846148
MAMLD1	163.7519369	157.7305822	144.9549679	114.6243116	67.63148427	104.0533281
ZNF235	42.67474719	35.93861367	43.95408704	79.42471983	66.48518792	83.24266246
METTL4	178.6384766	150.7425185	216.9648552	296.0375921	224.6740833	284.4124301
DCUN1D3	217.3434799	197.6623752	178.6219282	274.3763049	308.3537164	254.6829078

FADD	338.4206696	374.3605591	297.3914825	260.8380003	251.0388992	216.0345288
NCKAP5	21.83359159	27.95225508	25.25022021	61.37364714	67.63148427	37.65739492
ALG12	166.7292448	199.6589649	191.714635	252.7150176	265.9407517	265.5837326
VSIG10	658.9774915	621.9376755	604.1348984	506.3325889	516.9796509	456.8436595
TGM1	81.37975046	63.89086875	72.94508062	37.00469901	33.24259396	43.60329938
B4GALT3	950.75367	847.5523058	832.3220737	1016.275392	1139.418565	1105.93823
SLC16A8	22.82602757	32.9437292	26.18541355	53.25066443	49.29074277	71.35085354
TYK2	1496.59346	1514.413248	1509.402053	1304.190002	1177.246345	1274.405523
ATP5MG	368.193749	339.4202402	246.8910421	235.5664986	239.5759358	140.7197389
SRP9	4915.535415	4743.897005	4934.080068	4115.644573	4365.096476	4057.088811
DYNLL2	3044.79359	2827.170942	2739.181296	2482.022495	2454.220471	2329.803565
RALGDS	423.770164	437.253133	367.5309831	336.6525056	300.329642	269.5476689

TLCD2	119.0923177	98.8311876	101.0008809	141.7009206	200.6018601	158.5574523
RPS28	2233.973394	1648.184755	1445.808906	1377.296846	1335.43524	1326.927679
PPP3R1	683.7883911	664.864353	782.7568266	960.317067	974.351892	794.7692297
MOSPD3	469.4222191	428.2684796	360.9846297	300.5503603	345.0351994	274.5025893
RAC3	705.6219826	626.9291496	562.9863914	491.8917308	521.5648363	437.0239779
HNRNPUL2	1208.787025	1047.211271	1088.565049	913.384278	926.2074456	879.0028762
GIT2	504.1574785	519.1133086	570.4679381	651.643724	644.2185451	737.2921532
DNAJC30	183.6006565	204.650439	146.8253546	99.28089978	124.9463014	121.8910415
GAL3ST2	19.84871962	24.95737061	31.7965736	61.37364714	65.33889158	41.62133123
STK38L	679.8186471	705.7944407	875.3409674	941.3634407	947.9870761	991.975061
AP2A1	1967.008115	1828.876118	1737.589228	1493.726265	1523.427841	1588.547475
DARS2	1011.292265	1155.027112	1308.335484	960.317067	890.6722589	902.7864941

ITGAE	268.9501509	238.592463	218.8352418	187.731156	158.1888954	157.5664682
ZNF528	9.924359812	8.984653418	16.83348014	0.902553634	0	1.981968154
LARGE2	551.7944056	508.1320655	488.1709241	401.6363673	334.7185323	432.0690575
SH3GLB2	2313.368272	1938.688549	1806.793535	1574.956092	1721.737108	1581.610587
PRCC	1167.104714	1037.228322	1096.981789	1360.148327	1406.505613	1261.52273
SYTL4	134.9712934	119.7953789	129.0566811	79.42471983	82.53333673	79.27872615
FAM120B	493.2406827	560.0433964	553.634458	389.9031701	449.3481667	400.3575671
CLDND2	7.93948785	16.97101201	11.22232009	0.902553634	2.292592687	0
GALNS	300.7081023	272.534487	239.4094954	355.606132	371.4000153	370.6280448
AC026470.1	1811.195666	1876.79427	1972.322757	1561.417788	1628.887104	1565.754842
METTL8	445.6037556	478.1832208	517.1619177	390.8057237	351.9129775	358.7362358
GFAP	0	0	0	2.707660903	5.731481718	10.90082485

SYNE3	60.53859485	69.8806377	54.24121379	116.4294188	110.044449	89.18856692
CAMK2G	825.7067364	873.5079712	798.6551134	648.0335095	620.1463218	723.4183761
BIK	436.6718317	411.2974676	367.5309831	515.3581252	589.1963206	491.5281021
ELOVL2	2.977307944	0.998294824	3.740773365	12.63575088	13.75555612	19.81968154
NANOS1	28.78064346	20.96419131	35.53734697	13.53830452	8.024074405	1.981968154
INHBA	473.391963	482.1764001	498.4580509	646.2284022	644.2185451	563.8699398
GTDC1	130.0091135	132.7732116	135.6030345	209.3924432	232.6981577	159.5484364
TOB2	743.3345499	794.6426801	740.6731263	952.1940843	935.3778163	908.7323985
NFE2L3	986.4813653	1036.230028	1157.769356	910.6766171	768.0185502	856.2102425
IRAK3	53.59154299	65.8874584	70.13950059	34.29703811	30.95000127	29.72952231
RAPGEF3	146.8805252	129.7783272	88.84336742	67.69152258	80.24074405	65.40494908
NRF1	312.6173341	318.4560489	302.0674492	379.9750801	424.1296471	427.1141371

ANO9	548.8170976	555.0519223	446.0872238	730.1658902	636.1944707	644.13965
LHX1	55.57641495	67.88404805	72.00988727	113.7217579	91.70370748	125.8549778
ZNF558	187.5704004	221.621451	202.936955	284.3043948	272.8185298	288.3763664
DNAAF2	385.0651607	385.3418022	383.4292699	301.4529139	290.0129749	283.421446
SDHC	1879.673748	1801.922158	1660.903374	1528.023303	1455.796356	1463.683482
UBL3	243.1468154	299.4884473	302.0674492	379.9750801	384.0092751	373.600997
C14orf132	36.7201313	41.92838262	46.75966706	21.66128723	16.04814881	13.87377708
KLK10	135.9637294	121.7919686	121.5751344	82.13238073	87.11852211	65.40494908
B3GALT5	0	0	2.805580024	8.12298271	10.31666709	17.83771338
ATP6V1G1	1628.587445	1583.295591	1521.559566	1189.56569	1475.283394	1067.289851
PEX2	617.2951803	582.0058825	656.5057255	518.0657861	489.4685387	439.0059461
PIGV	417.8155481	388.3366866	367.5309831	278.889073	276.2574188	322.069825

MACROD1	840.5932761	752.7142975	680.8207524	540.629627	609.8296548	624.3199685
CUL2	784.0244252	959.3613261	947.3508547	1106.530756	1101.590786	1142.604641
UQCRB	3408.025159	3017.845254	2947.729412	2464.873976	2873.764933	2231.696141
KSR1	239.1770715	242.5856423	208.5481151	322.2116475	275.1111224	362.7001721
ZDHHC7	1459.873328	1528.389376	1500.050119	1871.896238	1819.172297	1678.727026
PIK3IP1	73.44026261	96.83459795	59.85237384	113.7217579	153.60371	120.9000574
OLR1	1145.271122	1299.779861	1408.401172	1021.690714	1044.275969	1055.398042
ZWILCH	587.5221009	580.0092929	673.3392057	524.3836616	457.3722411	438.014962
LONP2	754.2513457	752.7142975	825.7757203	968.4400497	938.8167053	955.3086501
TMEM59L	2.977307944	13.97612754	1.870386682	28.8817163	24.07222321	22.79263377
MAP2K6	48.62936308	43.92497227	58.9171805	25.27150176	14.90185247	26.75657008
UBE4B	1276.272672	1173.994713	1313.011451	1009.054963	990.4000408	1087.109532

TRIM39	175.6611687	175.6998891	158.0476747	245.4945886	269.3796407	213.0615765
TMEM206	298.7232303	288.5072042	262.7893289	394.4159382	374.8389043	360.718204
NDUFA3	979.5343135	867.5182023	734.1267729	646.2284022	700.3870659	660.9863793
SERTAD3	455.5281154	410.2991728	402.1331367	554.1679315	570.8555791	508.3748315
POC5	469.4222191	402.3128142	475.0782173	568.6087897	531.8815034	663.9593315
SNAPIN	533.9305579	475.1883363	461.0503172	636.3003123	621.2926182	609.4552073
NOL10	563.7036373	560.0433964	565.7919714	700.3816203	670.583361	738.2831373
CHM	386.0575967	466.2036829	395.5867833	533.4091979	569.7092827	518.2846722
NOB1	1364.599474	1182.979367	1159.639743	1472.967531	1572.718583	1480.530211
TCEA2	386.0575967	386.340097	311.4193826	263.5456612	265.9407517	269.5476689
NUDT6	75.42513457	74.87211182	70.13950059	44.22512809	28.65740859	42.61231531
FASTKD5	343.3828495	384.3435073	363.7902097	481.9636408	513.5407619	433.0600416

LRRC41	804.8655<br>808	826.5881<br>145	838.8684<br>271	661.5718<br>14	712.9963<br>257	610.4461<br>914
RTL8B	173.6762<br>967	147.7476<br>34	146.8253<br>546	94.76813<br>161	95.14259<br>651	113.9631<br>688
TIA1	674.8564<br>672	708.7893<br>252	819.2293<br>669	935.0455<br>652	886.0870<br>735	935.4889<br>686
ALKBH1	231.2375<br>836	224.6163<br>355	186.1034<br>749	282.4992<br>876	356.4981<br>628	271.5296<br>371
INTS13	1129.392<br>147	1155.027<br>112	1122.232<br>009	1330.364<br>057	1405.359<br>317	1346.747<br>361
BDKRB2	365.2164<br>411	355.3929<br>574	322.6417<br>027	281.5967<br>339	206.3333<br>418	264.5927<br>485
OGFOD3	588.5145<br>369	549.0621<br>533	474.1430<br>24	425.1027<br>618	355.3518<br>665	436.0329<br>938
AEN	1500.563<br>204	1396.614<br>459	1276.538<br>911	1752.759<br>158	1707.981<br>552	1603.412<br>236
IKBIP	369.1861<br>85	329.4372<br>92	355.3734<br>697	466.6202<br>29	440.1777<br>959	460.8075<br>958
NUDCD2	918.9957<br>186	859.5318<br>437	843.5443<br>938	703.0892<br>812	731.3370<br>672	695.6708<br>22
PSMD11	3333.592<br>461	3337.299<br>597	3301.232<br>495	2829.505<br>644	2866.887<br>155	2899.619<br>409
DGKA	3233.356<br>427	2974.918<br>576	2919.673<br>611	3408.945<br>077	3754.120<br>525	3624.028<br>769

1-Dec	1.984871962	3.993179297	1.870386682	17.14851905	14.90185247	12.882793
KCTD15	669.8942873	656.8779944	687.3671058	895.3332053	832.2111454	775.9405322
ARHGEF37	187.5704004	183.6862477	209.4833084	131.7728306	126.0925978	139.7287548
STXBP4	192.5325804	198.66067	238.474302	282.4992876	261.3555663	350.8083632
CP	63.5159028	56.90280498	32.73176694	27.97916267	17.19444515	19.81968154
LYNX1	46.64449112	34.94031885	43.95408704	10.83064361	22.92592687	17.83771338
ARPC5L	2303.443912	2116.385027	2034.980711	1788.861303	1877.633411	1735.213119
RPF1	956.7082859	897.467047	801.4606934	1064.110735	1146.296344	1071.253787
RECQL5	876.3209714	879.4977402	903.3967676	729.2633366	718.7278074	727.3823125
DIRAS3	2.977307944	4.991474121	6.546353389	22.56384086	16.04814881	20.81066562
ABCC3	233.2224556	209.6419131	191.714635	263.5456612	322.1092725	298.2862072
MCEE	113.1377019	96.83459795	81.36182069	56.86087897	44.7055574	63.42298092

TTC9	62.52346682	65.8874584	36.47254031	20.75873359	25.21851956	28.73853823
MKRN1	1363.607038	1325.735527	1266.251784	1575.858646	1659.837105	1508.277765
C5orf63	173.6762967	196.6640804	234.7335286	282.4992876	268.2333444	300.2681753
PSMD13	3350.463873	2987.896409	2717.67185	2461.263761	2630.750108	2372.41588
CRTAP	2075.183637	1979.618637	2077.999604	1522.607981	1780.198221	1782.780354
TRMU	884.2604593	728.7552217	796.7847267	665.1820286	582.3185425	652.0675226
TRAPPC12	740.357242	751.7160027	717.2932927	866.451489	903.2815187	939.4529049
GON7	191.5401444	207.6453234	208.5481151	132.6753843	159.3351917	125.8549778
GOLGA7B	26.79577149	27.95225508	17.76867348	50.54300353	49.29074277	55.49510831
LYRM4	578.590177	525.1030776	533.9953978	369.1444365	489.4685387	383.5108378
CCSAP	354.2996453	425.2735951	413.3554568	301.4529139	238.4296395	328.0157295
OXA1L	1787.377202	1670.147241	1629.1068	1889.94731	2062.187122	2152.417415

MAGOHB	459.4978593	515.1201293	479.754184	400.7338137	348.4740884	369.6370607
PTPRU	937.8520022	973.3374537	877.2113541	794.2471983	670.583361	769.0036437
TMEM140	90.31167429	77.86699629	67.33392057	125.4549552	119.2148197	132.7918663
GTF2H2C	252.0787392	212.6367976	244.0854621	303.2580212	394.3259422	295.3132549
TFCP2L1	81.37975046	57.90109981	90.7137541	47.83534262	33.24259396	38.648379
CYB5A	290.7837425	271.5361922	276.817229	203.9771214	206.3333418	204.1427198
POLG2	544.8473537	607.961548	622.8387653	494.5993917	450.494463	443.9608665
ITGB1BP1	740.357242	730.7518114	663.0520789	593.8802914	589.1963206	488.5551499
FIP1L1	591.4918448	555.0519223	540.5417512	649.8386168	727.8981781	748.1929781
B4GALNT3	16.87141168	36.9369085	19.63906017	47.83534262	55.02222449	60.45002869
ZXDA	42.67474719	49.91474121	49.56524708	83.03493437	90.55741114	78.28774208
ZNF346	345.3677215	309.4713955	363.7902097	434.1282981	440.1777959	455.8526754

CGNL1	31.7579514	29.94884473	31.7965736	12.63575088	11.46296344	9.909840769
ZNF608	96.26629018	98.8311876	107.5472342	166.9724224	137.5555612	156.5754842
ALG11	161.7670649	161.7237615	196.3906017	250.0073567	239.5759358	243.7820829
ACOX3	146.8805252	125.7851479	141.2141945	89.35280981	95.14259651	81.26069431
RSKR	90.31167429	105.8192514	137.4734212	68.59407621	52.7296318	72.34183761
METTL7A	13.89410374	15.97271719	18.70386682	0	5.731481718	2.972952231
ZNF335	861.4344317	829.582999	813.6182069	1046.962216	954.8648541	1055.398042
UBE3D	82.37218644	88.84823936	80.42662735	117.3319725	131.8240795	148.6476115
SDHB	2051.365173	1942.681728	1908.729609	1631.816971	1710.274145	1648.997504
STIL	810.8201967	928.4141866	984.7585883	729.2633366	589.1963206	784.8593889
NT5DC3	86.34193037	101.8260721	101.9360742	45.12768172	45.85185374	72.34183761
LARP7	501.1801705	490.1627587	553.634458	570.4138969	734.7759562	702.6077105

C8orf44	108.175522	117.7987893	125.3159077	60.47109351	80.24074405	75.31478985
VWDE	178.6384766	182.6879528	205.7425351	123.6498479	108.8981526	147.6566275
NUDT14	221.3132238	210.6402079	182.3627015	132.6753843	155.8963027	133.7828504
MYT1	0.992435981	1.996589649	0	8.12298271	13.75555612	11.89180892
DGKQ	379.1105448	345.4100092	358.1790497	275.2788585	283.1351968	257.65586
DDX27	2122.820564	1834.865887	1830.173369	2248.261103	2590.629736	2221.7863
C20orf194	350.3299014	399.3179297	390.9108166	494.5993917	449.3481667	544.0502582
SSR2	1850.893105	1647.18646	1610.402934	1942.295421	2249.033426	2010.706692
SLC25A33	329.4887458	328.4389972	312.354576	250.9099104	257.9166773	201.1697676
MAPRE3	237.1921995	202.6538493	204.8073417	299.6478066	300.329642	281.4394778
URB2	629.2044121	626.9291496	649.0241788	537.9219661	472.2740935	501.4379429
DHRS7B	445.6037556	378.3537384	367.5309831	500.0147135	510.1018729	534.1404175

FAM160A2	577.5977411	585.000767	518.097111	727.4582293	749.6778087	647.1126022
SRFBP1	305.6702822	311.4679852	361.9198231	425.1027618	417.251869	442.9698824
LRRC56	43.66718317	49.91474121	37.40773365	77.61961256	79.0944477	79.27872615
C17orf58	447.5886275	417.2872365	341.3455695	321.3090938	280.8426042	288.3763664
RSU1	682.7959551	629.9240341	622.8387653	846.5953091	759.9944758	794.7692297
DNAJC3	608.3632565	674.8473012	707.9413593	857.4259527	957.1574468	737.2921532
TIPRL	1033.125856	909.4465849	1113.815269	1293.359358	1179.538937	1303.144061
PREX2	1.984871962	0.998294824	4.675966706	14.44085815	21.77963053	9.909840769
TMPRSS4	489.2709387	457.2190295	430.188937	543.3372879	633.901878	585.6715895
CCDC85C	1091.679579	1086.144769	1012.814389	920.6047071	811.5778112	874.0479558
NSDHL	374.1483649	288.5072042	347.8919229	236.4690522	269.3796407	230.8992899
KCTD21	113.1377019	93.83971348	108.4824276	152.5315642	162.7740808	160.5394205

RAP2C	389.0349046	403.311109	452.6335772	503.624928	613.2685438	511.3477837
QRSL1	393.0046486	446.2377864	448.8928038	522.5785543	572.0018754	554.9510831
CASP7	465.4524752	458.2173243	428.3185503	563.1934679	553.6611339	593.5994621
PLAGL2	1107.558555	1150.035638	1210.140184	1331.266611	1380.140798	1467.647418
ZNF181	49.62179906	45.92156192	61.72276052	98.37834615	97.4351892	79.27872615
TCIRG1	1036.103164	1054.199334	962.3139481	1304.190002	1171.514863	1224.856319
SSU72	2456.279054	2173.287832	2273.455013	1883.629435	2016.335268	1910.6173
VGLL4	604.3935126	659.8728788	626.5795386	804.1752883	748.5315123	784.8593889
ZNF33A	420.792856	484.1729898	453.5687705	610.1262569	543.3444668	577.7437168
NPHS1	0	0.998294824	1.870386682	12.63575088	11.46296344	8.918856692
TRNAU1AP	494.2331186	425.2735951	442.3464504	346.5805956	362.2296446	338.9165543
BCORL1	290.7837425	337.4236506	298.3266759	237.3716058	240.7222321	196.2148472

DDX49	879.2982794	758.7040664	789.30318	670.5973504	680.900028	566.842892
CENPBD1	220.3207878	216.6299769	183.2978949	273.4737512	303.768531	277.4755415
TLL2	43.66718317	28.9505499	36.47254031	15.34341178	11.46296344	15.85574523
HIST1H2BC	56.56885093	67.88404805	79.491434	38.80980628	36.68148299	30.72050638
VDR	739.364806	725.7603372	750.0250597	598.3930596	585.7574315	610.4461914
RHBDD1	426.7474719	454.224145	480.6893774	584.8547551	574.2944681	563.8699398
ALDH3A1	300.7081023	245.5805268	269.3356823	379.0725265	394.3259422	328.0157295
FIZ1	500.1877345	440.2480175	405.8739101	593.8802914	619.0000255	530.1764811
NTAN1	316.587078	326.4424075	277.7524223	201.2694605	231.5518614	239.8181466
RAB18	805.8580167	857.535254	947.3508547	1134.509918	1028.22782	1052.42509
NDOR1	625.2346682	594.9837153	585.4310316	458.4972463	478.0055752	498.4649907
PPP1R2	1173.05933	1277.817375	1296.177971	1447.69603	1613.985252	1463.683482

HAGH	213.3737<br>36	187.6794<br>27	159.9180<br>613	126.3575<br>088	128.3851<br>905	123.8730<br>096
ST7	343.3828<br>495	327.4407<br>024	303.0026<br>426	413.3695<br>646	412.6666<br>837	442.9698<br>824
LAPTM4A	1589.882<br>442	1558.338<br>221	1506.596<br>473	1271.698<br>071	1377.848<br>205	1245.666<br>985
STX18	514.0818<br>383	476.1866<br>312	432.9945<br>17	585.7573<br>087	648.8037<br>304	582.6986<br>372
ATG4A	257.0409<br>191	247.5771<br>164	240.3446<br>887	320.4065<br>402	373.6926<br>08	311.1690<br>002
TSPAN12	110.1603<br>939	107.8158<br>41	84.16740<br>071	51.44555<br>716	64.19259<br>524	62.43199<br>685
NUDT19	407.8911<br>883	449.2326<br>709	466.6614<br>773	340.2627<br>202	361.0833<br>482	312.1599<br>842
MGMT	210.3964<br>28	246.5788<br>216	185.1682<br>816	135.3830<br>452	153.6037<br>1	152.6115<br>478
DIP2A	1077.785<br>476	1182.979<br>367	1167.121<br>29	1442.280<br>708	1298.753<br>757	1393.323<br>612
CCNH	825.7067<br>364	851.5454<br>851	831.3868<br>803	964.8298<br>352	1048.861<br>154	1033.596<br>392
RETSAT	1025.186<br>369	916.4346<br>487	889.3688<br>675	750.0220<br>702	794.3833<br>661	754.1388<br>825
DNASE1L<br>1	141.9183<br>453	102.8243<br>669	109.4176<br>209	79.42471<br>983	73.36296<br>599	61.44101<br>277

SERPINB3	18.85628364	42.92667744	23.37983353	9.025536344	9.170370748	8.918856692
FBXO7	2156.563387	2137.349219	2107.925791	2422.453955	2501.218622	2550.793014
ROMO1	2229.011214	1737.032994	1349.483991	1178.735047	1093.566712	848.2823698
EPB41L4A	32.75038738	30.94713955	28.99099358	76.71705892	52.7296318	54.50412423
TBX3	55.57641495	80.86188076	71.07469393	42.42002082	34.38889031	30.72050638
LDLR	3258.167326	3254.441127	2771.913063	3627.363057	3680.757559	3727.091113
RAB17	157.797321	143.7544547	133.7326478	183.2183878	223.527787	223.9624014
SUCLA2	762.1908336	766.690425	798.6551134	912.4817244	989.2537445	936.4799527
R3HDM2	885.2528952	936.4005452	913.6838944	1117.361399	1074.079674	1103.956262
FAT2	3293.895022	4109.979791	4262.611249	2720.296654	1877.633411	2850.070205
USP11	672.8715953	633.9172134	585.4310316	497.3070526	507.8092802	498.4649907
PLPPR2	302.6929743	296.4935628	259.0485555	222.9307477	194.8703784	206.124688

LHX6	30.76551542	39.93179297	35.53734697	12.63575088	14.90185247	14.86476115
TBCC	353.3072093	388.3366866	346.9567296	264.4482149	281.9889005	277.4755415
SAMD11	38.70500327	38.93349815	34.60215363	19.85617996	14.90185247	9.909840769
CCDC24	475.376835	444.2411968	439.5408704	324.9193084	393.1796458	321.0788409
ORC4	401.9365724	440.2480175	456.3743505	543.3372879	573.1481718	535.1314015
FBXW7	310.6324621	408.3025831	351.6326963	520.7734471	463.1037228	438.014962
ZNF658	152.8351411	131.7749168	152.4365146	107.4038825	73.36296599	97.11643954
SIRT5	172.6838607	184.6845425	181.4275082	126.3575088	131.8240795	109.9992325
MAN2B2	389.0349046	390.3332763	400.26275	284.3043948	295.7444566	322.069825
ZNF708	11.90923177	7.986358594	5.611160047	0.902553634	0	0
HMG20A	339.4131056	456.2207347	469.4670573	564.0960215	543.3444668	562.8789557
METRNL	393.0046486	268.5413077	262.7893289	229.2486231	229.2592687	169.4582772

FAM222B	515.0742743	501.1440018	480.6893774	613.7364714	615.5611365	640.1757137
BCAN	86.34193037	97.83289278	100.0656875	158.8494397	139.8481539	135.7648185
SDC1	6248.376938	5609.418617	5002.349182	4745.62701	4985.242798	3875.738725
SNX10	424.7626	471.195157	418.0314235	343.8729347	331.2796433	338.9165543
NUTF2	2071.213893	1872.80109	1825.497402	1552.392251	1595.64451	1680.708994
ZSCAN20	74.43269859	85.85335489	91.64894744	146.2136888	132.9703758	116.9361211
MICAL3	819.7521205	1005.282888	906.2023476	778.9037865	582.3185425	748.1929781
DCLK1	12.90166776	22.96078096	17.76867348	38.80980628	48.14444643	38.648379
RAB24	128.0242416	119.7953789	99.13049417	172.3877442	204.0407491	149.6385956
MED11	166.7292448	124.786853	123.445521	85.74259527	80.24074405	96.12545546
CGAS	908.0789228	960.3596209	1002.527262	797.8574128	769.1648465	790.8052934
CDC42BPB	1499.570768	1739.029584	1594.504647	1968.469477	1822.611186	2006.742756

C4orf19	35.72769532	39.93179297	37.40773365	68.59407621	71.0703733	68.37790131
HCAR2	466.4449112	376.3571487	357.2438563	513.553018	521.5648363	531.1674652
KIN	180.6233486	155.7339926	186.1034749	230.1511768	267.087048	236.8451944
TSNAX	661.9547995	687.8251339	661.1816922	794.2471983	961.7426322	774.9495481
MRPL15	1053.967012	955.3681468	853.8315205	722.0429075	831.064849	729.3642806
CX3CL1	11.90923177	12.97783272	13.09270678	3.610214538	0	0.990984077
DLX4	66.49321074	46.91985674	37.40773365	84.84004163	104.3129673	82.25167838
SHPK	69.47051869	55.90451016	43.0188937	33.39448447	25.21851956	20.81066562
EVL	283.8366906	299.4884473	289.9099358	205.7822286	225.8203797	216.0345288
SMARCA5	2052.357609	2317.042287	2245.399212	2593.939145	2501.218622	2738.089005
PGM2	1516.442179	1720.061982	1734.783648	1991.033318	1996.84823	1936.382886
PTPRG	425.7550359	543.0723844	587.3014183	765.365482	608.6833584	679.8150768

GART	3384.206696	3447.112028	3669.698671	4055.173479	4112.911281	4002.584687
SHROOM1	159.782193	161.7237615	154.3069013	125.4549552	82.53333673	100.0893918
DGAT1	704.6295467	646.8950461	595.7181584	537.9219661	469.9815008	524.2305767
NUP210	299.7156663	320.4526386	331.9936361	448.5691563	373.692608	426.1231531
SWAP70	1753.634379	1836.862477	1938.655796	2113.780612	2260.496389	2158.36332
SNX13	696.6900588	940.3937244	967.9251082	1157.073759	1046.568562	1136.658736
IL3RA	6.947051869	8.984653418	3.740773365	17.14851905	34.38889031	18.82869746
CHID1	1376.508706	1292.791797	1252.223884	1161.586527	1009.887079	1059.361978
EIF1AD	796.9260929	743.7296441	804.2662735	912.4817244	975.4981883	969.1824272
NDUFA11	324.5265659	236.5958733	182.3627015	185.9260487	151.3111173	147.6566275
NSD2	4315.111646	4356.558613	4305.630143	3876.46786	3413.670511	3807.360823
MAP3K7	893.1923831	976.3323381	991.3049417	1149.85333	1117.638935	1190.171876

TBC1D12	129.0166776	154.7356978	156.177288	203.0745677	210.9185272	214.0525606
AC007950.1	484.3087588	450.2309657	463.8558972	558.6806997	663.7055829	561.8879716
DNAAF1	4.962179906	9.982948243	6.546353389	22.56384086	25.21851956	22.79263377
HERPUD2	524.9986341	570.0263447	559.2456181	628.1773295	708.4111403	745.2200258
WARS2	335.4433617	333.4304713	341.3455695	411.5644573	513.5407619	408.2854397
GUCY1A1	43.66718317	34.94031885	45.82447372	16.24596542	22.92592687	14.86476115
GLOD4	1099.619067	1134.06292	966.0547215	833.9595582	933.0852236	827.4717042
MCM9	146.8805252	140.7595702	159.9180613	93.86557798	83.67963308	113.9631688
GABRB2	0.992435981	0.998294824	2.805580024	15.34341178	9.170370748	11.89180892
PSMC5	3472.533498	3035.814561	2801.83925	2533.468052	2701.820482	2493.315938
ZNF3	927.9276424	879.4977402	912.748701	1037.93668	1233.414866	1066.298867
HRG	0.992435981	1.996589649	2.805580024	18.95362632	9.170370748	10.90082485

RPL3	33215.83986	28099.00442	25486.82413	33097.54433	38051.30712	34276.15725
SLC10A7	239.1770715	234.5992837	260.9189422	326.7244157	294.5981603	370.6280448
CGRRF1	71.45539065	89.84653418	71.07469393	114.6243116	136.4092649	118.9180892
RNF141	823.7218644	855.5386644	942.674888	710.3097103	755.4092904	646.1216181
TRPV6	0	1.996589649	0.935193341	12.63575088	10.31666709	8.918856692
TGIF2	1072.823296	1012.270952	944.5452746	1221.155067	1256.340792	1180.262036
SPAG4	268.9501509	260.5549491	199.1961817	320.4065402	416.1055727	295.3132549
KLHL12	1233.597925	1257.851479	1217.62173	1494.628819	1422.553762	1476.566275
SRP72	3843.704555	3978.204875	4117.656281	4424.317916	4799.54279	4621.949735
HIKESHI	989.4586733	896.4687522	834.1924604	758.1450529	740.5074379	701.6167265
ZDHHC20	1548.200131	1904.746525	1970.45237	1496.433926	1408.798206	1508.277765
RTN2	396.9743925	387.3383918	337.6047962	275.2788585	315.2314945	245.7640511

WDR20	315.594642	263.5498336	314.2249627	426.9078691	386.3018678	369.6370607
TXN2	1810.20323	1555.343336	1466.383159	1340.292147	1301.04635	1326.927679
GDI1	1804.248614	1711.077329	1539.32824	1997.351193	2018.627861	2005.751772
PYROXD2	149.8578332	161.7237615	151.5013213	94.76813161	104.3129673	110.9902166
POP4	443.6188836	444.2411968	468.531864	318.6014329	372.5463116	360.718204
HNRNPDL	5900.031908	5484.631764	5423.186186	6391.884839	6964.896583	6239.235748
S100A6	7671.530135	6295.247162	4942.496808	4153.551826	4433.874257	3256.373677
ZNF143	313.6097701	360.3844316	354.4382763	410.6619037	458.5185374	468.7354684
GOLGA6L7	122.0696257	112.8073151	102.8712675	156.1417788	191.4314894	156.5754842
FEZ1	1654.390781	1409.592292	1398.114045	1785.251089	1964.751933	1691.609819
ASPSCR1	539.8851738	501.1440018	546.1529113	405.2465818	432.1537215	418.1952805
PRRC2A	7081.030726	7177.739786	6983.088679	8553.500793	8029.805886	7827.783224

PHRF1	1613.700905	1612.246141	1485.087026	1391.737704	1265.511163	1282.333396
RPIA	423.770164	363.379316	361.9198231	307.7707893	292.3055676	267.5657008
COX8A	2521.779828	1881.785744	1376.604598	1182.345261	1084.396341	759.0938029
SMARCE1	132.9864215	183.6862477	208.5481151	260.8380003	222.3814906	283.421446
MRPL12	585.5372289	467.2019778	321.7065094	265.3507685	210.9185272	124.8639937
FLI1	409.8760602	429.2667744	413.3554568	529.7989834	511.2481692	537.1133697
ABCC1	2185.344031	2406.888821	2426.82672	2037.063553	1905.144523	2004.760788
PDCL	645.0833878	664.864353	634.9962787	486.4764089	562.8315047	507.3838474
WDR78	35.72769532	25.95566543	28.99099358	9.928089978	13.75555612	9.909840769
B4GALT4	334.4509257	310.4696903	357.2438563	255.4226785	262.5018627	234.8632262
STAM2	312.6173341	334.4287661	330.1232495	432.3231909	385.1555714	450.897755
PPA1	1968.992987	2012.562366	1903.118449	2177.86192	2388.88158	2351.605215

RRAGA	1766.536047	1641.196691	1451.420066	1972.982245	2027.798232	1864.041049
GPR180	196.5023243	224.6163355	254.3725888	305.0631284	290.0129749	326.0337613
ZNF460	112.1452659	177.6964787	218.8352418	377.2674192	257.9166773	486.5731818
TMEM11	493.2406827	435.2565434	452.6335772	525.2862152	631.6092853	608.4642232
FCMR	142.9107813	150.7425185	115.9639743	184.1209414	191.4314894	219.9984651
SH3TC2	200.4720682	265.5464233	186.1034749	147.1162424	128.3851905	165.4943408
MINPP1	238.1846355	270.5378974	258.1133622	188.6337096	184.5537113	186.3050065
TTF1	593.4767168	577.0144084	636.8666654	463.0100145	510.1018729	467.7444843
SLC13A5	0.992435981	0	0.935193341	9.025536344	12.60925978	7.927872615
ATF6B	1724.853735	1570.317759	1520.624373	1351.122791	1367.531538	1318.999806
FGFBP1	1935.250163	1706.085855	1486.022219	1225.667836	1344.605611	1519.17859
NOSTRIN	47.6369271	48.91644639	60.78756718	16.24596542	25.21851956	32.70247454

PMF1	334.4509257	328.4389972	288.0395491	238.2741595	239.5759358	232.8812581
RNF39	112.1452659	104.8209565	56.11160047	147.1162424	177.6759332	129.8189141
FUOM	219.3283518	182.6879528	175.8163482	126.3575088	159.3351917	93.15250323
GRAMD4	530.9532499	554.0536275	529.3194311	455.7895854	394.3259422	422.1592168
ZFP90	281.8518187	295.495268	313.2897693	196.7566923	240.7222321	220.9894492
WDR74	1001.367905	881.4943298	828.5813003	750.0220702	766.8722538	643.1486659
USP20	713.5614705	637.9103927	589.171805	537.9219661	461.9574264	523.2395926
RNASET2	163.7519369	118.7970841	116.8991677	74.91195166	75.65555867	98.10742361
RMDN1	954.7234139	959.3613261	961.3787548	743.7041948	836.7963308	790.8052934
TFDP1	2485.059697	2666.445476	2624.152515	2315.050072	2155.037126	2176.201033
MIS12	309.6400261	296.4935628	367.5309831	454.8870317	440.1777959	389.4567422
CAMK2N1	175.6611687	202.6538493	187.9738616	111.9166507	138.7018576	139.7287548

RUBCNL	0.992435<br>981	1.996589<br>649	3.740773<br>365	9.025536<br>344	16.04814<br>881	15.85574<br>523
EML4	1383.455<br>758	1443.534<br>316	1582.347<br>133	1816.840<br>466	1650.666<br>735	1811.518<br>893
PTPRE	461.4827<br>313	459.2156<br>192	421.7721<br>969	335.7499<br>52	343.8889<br>031	365.6731<br>244
GTF2F1	1914.409<br>008	1764.985<br>249	1663.708<br>954	2077.678<br>466	2382.003<br>802	2000.796<br>851
LZTR1	262.9955<br>35	236.5958<br>733	203.8721<br>484	197.6592<br>459	140.9944<br>503	141.7107<br>23
C5orf22	1135.346<br>763	1228.900<br>929	1258.770<br>237	1009.957<br>517	1021.350<br>042	989.0021<br>088
HDLBP	8739.391<br>251	8526.436<br>094	8509.324<br>212	9296.302<br>434	10006.02<br>078	10377.58<br>525
TCTN1	526.9835<br>06	545.0689<br>74	591.9773<br>85	404.3440<br>282	409.2277<br>946	485.5821<br>977
DDX5	13435.59<br>831	13819.39<br>525	14123.28<br>984	15279.33<br>048	16478.00<br>994	15538.63<br>033
TMEM245	1305.053<br>315	1441.537<br>726	1477.605<br>479	1222.057<br>621	1140.564<br>862	1156.478<br>418
ORC5	982.5116<br>214	1014.267<br>541	1013.749<br>582	1150.755<br>884	1211.635<br>235	1237.739<br>112
DDX23	1828.067<br>077	1610.249<br>552	1590.763<br>873	1337.584<br>486	1403.066<br>724	1451.791<br>673

PSIP1	2037.471069	2007.570892	2150.944685	1823.158342	1732.053775	1721.339342
STX1B	8.931923831	12.97783272	14.02790012	1.805107269	1.146296344	1.981968154
G2E3	387.0500327	480.1798105	594.782965	672.4024576	657.9741012	614.4101277
MTF2	803.8731448	773.6784888	838.8684271	973.8553715	943.4018907	1006.839822
NISCH	1268.333184	1189.967431	1186.76035	1363.758542	1442.0408	1575.664682
LSR	1892.575416	1795.932389	1618.819674	1490.11605	1546.353767	1371.521962
SEC22A	169.7065528	165.7169408	193.5850216	234.6639449	267.087048	233.8722422
DPH5	480.3390149	413.2940572	448.8928038	562.2909142	602.9518767	537.1133697
SLC9A8	567.6733813	485.1712846	635.931472	728.360783	696.9481769	717.4724717
BX255925.3	402.9290084	379.3520332	319.8361227	270.7660903	283.1351968	275.4935734
ZC4H2	103.213342	97.83289278	111.2880076	56.86087897	74.50926233	60.45002869
PNPLA4	223.2980958	166.7152357	216.9648552	127.2600625	157.0425991	131.8008822

ZMYM3	942.8141822	935.4022503	949.2212413	782.514001	766.8722538	795.7602138
ZNF629	601.4162046	599.9751894	628.4499253	470.2304435	510.1018729	490.5371181
DLX6	142.9107813	168.7118253	133.7326478	106.5013289	105.4592636	75.31478985
MXD3	225.2829677	197.6623752	164.5940281	155.2392251	131.8240795	101.0803758
SSBP4	1219.703821	1116.093614	1029.647869	1018.0805	817.3092929	858.1922106
HP1BP3	3880.424687	4164.886007	4228.009096	4892.743252	4668.865007	4633.841544
APOLD1	36.7201313	51.91133086	58.9171805	22.56384086	19.48703784	26.75657008
ATM	977.5494415	1220.91457	1380.345372	1727.487656	1284.998201	1634.132743
UBA3	1106.566119	1157.023701	1137.195103	995.5166588	935.3778163	896.8405896
C15orf39	862.4268677	748.7211182	708.8765526	931.4353507	1035.105598	914.678303
NONO	6751.54198	6513.873728	6367.73146	5301.600049	5916.035429	5749.689614
TBC1D19	36.7201313	36.9369085	45.82447372	64.08130804	66.48518792	88.19758285

RRP7A	1023.201497	1017.262426	901.5263809	798.7599665	864.307443	714.4995195
NOP10	1157.180354	973.3374537	904.331961	793.3446446	878.0629991	740.2651055
PTPN3	884.2604593	964.3528002	943.6100813	811.3957173	691.2166951	769.9946278
SNCAIP	162.7595009	147.747634	177.6867348	194.0490314	275.1111224	233.8722422
EXOC8	259.0257911	374.3605591	377.8181099	440.4461736	475.7129826	449.9067709
PFKP	5228.152749	4680.006136	4175.638269	5561.535495	5761.285423	5450.412423
ZNF155	38.70500327	42.92667744	57.98198716	64.08130804	85.97222576	104.0533281
KRIT1	262.003099	332.4321765	288.9747424	374.5597583	362.2296446	442.9698824
HERC4	709.5917266	831.5795886	793.0439534	1025.300929	906.7204077	938.4619208
DPPA2	19.84871962	18.96760166	21.50944685	2.707660903	4.585185374	8.918856692
NPR3	621.2649242	676.8438908	667.7280456	573.1215578	460.8111301	520.2666404
USP37	262.003099	336.4253558	334.7992162	426.9078691	364.5222372	457.8346435

NELFE	1773.483098	1504.4303	1401.854818	1217.544853	1361.800056	1227.829271
TUBGCP6	979.5343135	968.3459795	838.8684271	794.2471983	662.5592866	775.9405322
SMC2	1482.699356	1743.022763	1836.719722	1373.686632	1196.733383	1505.304813
IFNGR2	864.4117396	834.5744731	836.9980404	978.3681397	1086.688934	999.9029336
RP2	286.8139986	307.4748059	293.6507091	216.6128723	191.4314894	245.7640511
ZFP62	341.3979775	418.2855314	440.4760637	491.8917308	502.0777985	568.8248601
EIF3H	4653.532316	4337.591011	4284.120696	4818.733854	5561.829859	5169.963929
RFXAP	191.5401444	201.6555545	234.7335286	312.2835575	298.0370493	252.7009396
MESP2	5.954615887	4.991474121	13.09270678	0	0	0.990984077
MUC20	6.947051869	10.98124307	6.546353389	13.53830452	34.38889031	30.72050638
MTIF2	1030.148549	957.3647365	1023.101515	1116.458846	1267.803756	1252.603873
GRWD1	1154.203046	1059.190809	1053.027702	861.9387209	853.9907759	970.1734113

DNAL4	153.8275771	116.8004944	120.639941	180.5107269	207.4796382	182.3410702
LANCL1	970.6023896	1010.274362	1193.306703	957.6094061	751.9704013	797.7421819
GRIK1	2.977307944	0.998294824	0.935193341	9.928089978	18.3407415	7.927872615
UTP3	785.0168611	719.7705683	751.8954463	600.1981669	644.2185451	593.5994621
WASHC2C	536.9078658	550.0604482	468.531864	623.6645614	684.3389171	642.1576818
SCAF1	1857.840157	1791.93921	1703.922268	1574.956092	1513.111173	1437.917896
SLC8B1	859.4495597	834.5744731	800.5255001	664.2794749	659.1203975	719.4544398
ROM1	44.65961915	44.92326709	33.66696028	12.63575088	18.3407415	22.79263377
XYLB	116.1150098	106.8175462	140.2790012	75.81450529	61.90000255	88.19758285
TMEM223	507.1347864	473.1917467	455.4391572	392.610831	397.7648312	313.1509683
SUPT3H	130.0091135	121.7919686	143.0845812	181.4132805	189.1388967	194.2328791
FAM136A	1094.656887	1085.146474	1094.176209	948.5838698	899.8426297	886.9307488

YIF1B	922.9654625	805.6239232	769.6641198	700.3816203	680.900028	629.2748888
NPTN	1311.007931	1311.759399	1284.955651	1111.043524	1048.861154	1128.730864
PHF20	1721.876427	1743.022763	1673.060887	1389.932597	1509.672284	1465.66545
FAM131A	848.5327639	883.4909195	711.6821327	714.8224785	614.4148401	599.5453665
MRPS11	597.4464607	520.1116034	523.7082711	430.5180836	450.494463	417.2042964
FZD6	1687.141168	1813.901696	1888.155356	2212.158958	2138.988977	2015.661612
MRS2	529.960814	558.0468068	589.171805	433.2257445	421.8370544	475.6723569
ZNF467	56.56885093	48.91644639	29.92618692	84.84004163	84.82592942	74.32380577
CDHR3	74.43269859	75.87040664	66.39872723	37.90725265	38.97407568	44.59428346
PAF1	973.5796976	785.6580267	786.4976	1014.470285	1105.029675	1037.560329
CALCRL	34.73525934	31.94543438	45.82447372	79.42471983	60.75370621	67.38691723
COA5	273.9123308	335.427061	300.1970625	208.4898895	241.8685285	223.9624014

SPATC1L	220.3207878	203.6521441	188.9090549	141.7009206	154.7500064	137.7467867
PMEL	74.43269859	75.87040664	81.36182069	46.03023535	40.12037202	46.57625161
FEZ2	612.3330004	604.9666635	658.3761122	725.6531221	809.2852185	772.96758
ASPHD1	144.8956533	130.776622	126.2511011	86.6451489	102.0203746	71.35085354
NLRP10	0	6.98806377	3.740773365	14.44085815	25.21851956	12.882793
IQCC	119.0923177	112.8073151	114.0935876	74.91195166	72.21666964	74.32380577
AKR7A3	7.93948785	15.97271719	10.28712675	0	2.292592687	1.981968154
NSFL1C	1064.883808	973.3374537	913.6838944	816.8110391	841.3815161	756.1208507
MAN1A2	678.8262112	867.5182023	992.240135	1204.006548	982.3759664	1096.028389
TENM1	5.954615887	7.986358594	9.351933412	33.39448447	12.60925978	28.73853823
LRRC59	4116.62445	3913.315711	3968.025347	4589.485231	4799.54279	4430.689808
TATDN2	393.0046486	379.3520332	389.04043	332.1397375	286.5740859	266.5747167

ATP2C1	2018.614786	2291.086622	2300.575619	2601.159574	2524.144548	2654.846342
SNX15	34.73525934	15.97271719	14.02790012	48.73789626	49.29074277	46.57625161
TTC7A	658.9774915	606.9632531	595.7181584	522.5785543	495.2000204	475.6723569
ALOX5AP	0	0	0	2.707660903	12.60925978	1.981968154
TNFRSF10D	798.9109649	815.6068714	795.8495334	922.4098144	1028.22782	962.2455387
FYCO1	721.5009583	781.6648474	764.0529598	651.643724	533.0277997	631.256857
XRCC4	434.6869598	471.195157	430.188937	314.0886648	377.131497	346.8444269
THAP3	189.5552724	147.747634	165.5292214	226.5409622	231.5518614	241.8001148
BLOC1S2	495.2255546	429.2667744	478.8189907	557.7781461	641.9259524	573.7797805
NUTM1	7.93948785	6.98806377	14.96309346	0	2.292592687	0
ITPRID2	3442.760419	3763.571487	3863.283693	4303.375729	4145.007578	4411.86111
IFNLR1	124.0544977	128.7800323	141.2141945	185.0234951	193.7240821	182.3410702

CHST11	429.7247799	419.2838262	451.6983838	548.7526097	500.9315021	592.608478
RTP4	19.84871962	14.97442236	29.92618692	6.317875441	5.731481718	6.936888538
FAM43A	410.8684962	380.350328	395.5867833	336.6525056	307.2074201	254.6829078
BOLA3	236.1997635	309.4713955	283.3635824	209.3924432	197.1629711	198.1968154
DCAF4	296.7383584	285.5123197	279.622809	182.3158342	245.3074175	206.124688
EXOSC6	807.8428887	852.5437799	808.9422402	678.7203331	726.7518818	591.6174939
XRCC3	601.4162046	543.0723844	453.5687705	456.692139	385.1555714	374.5919811
GADD45G	0.992435981	1.996589649	0	13.53830452	12.60925978	4.954920385
MBTPS1	2116.865948	2135.352629	2088.286731	2470.289297	2322.396392	2620.161899
PRSS36	18.85628364	8.984653418	19.63906017	4.512768172	1.146296344	3.963936308
TRIM62	176.6536047	201.6555545	201.0665684	139.8958133	135.2629685	133.7828504
DPAGT1	773.1076294	714.7790942	731.3211928	524.3836616	592.6352096	658.0134271

PDIK1L	152.8351411	119.7953789	151.5013213	89.35280981	82.53333673	105.0443122
DVL3	2489.029441	2572.605762	2419.345174	2910.735471	2744.233446	3069.077686
TTC30B	52.599107	47.91815156	57.98198716	25.27150176	26.3648159	28.73853823
KLF11	310.6324621	282.5174353	272.1412623	364.6316683	366.8148299	393.4206785
ZNF554	32.75038738	29.94884473	21.50944685	5.415321806	11.46296344	12.882793
ALKBH4	417.8155481	392.3298659	375.9477232	301.4529139	342.7426067	250.7189715
PRSS16	78.40244252	93.83971348	99.13049417	129.0651697	147.8722283	135.7648185
FAM178B	310.6324621	317.4577541	300.1970625	239.1767131	212.0648236	246.7550352
TTC14	601.4162046	696.8097873	813.6182069	898.9434199	845.9667015	916.6602711
TRMT1	868.3814836	724.7620424	702.3301993	628.1773295	632.7555816	566.842892
MAPKAPK2	2282.602757	2098.415721	2035.915904	2426.966723	2552.801957	2531.964317
APOPT1	139.9334734	163.7203512	142.1493879	194.951585	249.8926029	193.241895

ZNF385C	1.984871962	2.994884473	8.416740071	10.83064361	19.48703784	26.75657008
TSC2	1269.32562	1150.035638	1183.019577	1009.957517	941.109298	1047.470169
SLC39A8	630.1968481	641.903572	658.3761122	557.7781461	503.2240948	495.4920385
ADAM12	0.992435981	2.994884473	3.740773365	9.025536344	21.77963053	11.89180892
REXO4	1161.150098	1024.25049	1067.990796	1315.020645	1301.04635	1267.468634
OSBPL1A	458.5054233	463.2087985	451.6983838	323.1142011	332.4259396	406.3034715
OVGP1	22.82602757	14.97442236	21.50944685	38.80980628	40.12037202	50.54018792
ZNF461	2.977307944	2.994884473	0	9.025536344	11.46296344	17.83771338
TCF3	3083.498594	2849.133428	2684.940083	2471.191851	2434.733434	2460.613463
CAMTA1	411.8609322	378.3537384	440.4760637	286.1095021	349.6203848	309.187032
ADCY6	90.31167429	78.86529112	83.23220737	135.3830452	129.5314868	119.9090733
PAQR5	130.0091135	158.7288771	143.0845812	221.1256404	204.0407491	186.3050065

CUL5	849.5251999	1037.228322	1072.666762	1230.180604	1196.733383	1200.081717
EHHADH	220.3207878	225.6146303	238.474302	198.5617996	129.5314868	145.6746593
SGO1	195.5098883	286.5106146	254.3725888	335.749952	326.6944579	339.9075384
C18orf25	331.4736177	446.2377864	371.2717565	459.3997999	526.1500217	515.31172
ACOT2	362.2391331	339.4202402	324.5120894	468.4253363	439.0314996	408.2854397
CDK2AP2	1443.994353	1221.912865	1140.000683	1511.777338	1587.620436	1530.079415
AGL	612.3330004	840.564242	816.4237869	1014.470285	857.429665	1039.542297
POLR3F	248.1089953	256.5617698	293.6507091	306.8682357	425.2759434	351.7993473
DQX1	94.28141822	98.8311876	101.0008809	59.56853987	43.55926105	72.34183761
GORASP2	1628.587445	1599.268308	1689.894368	1389.030043	1391.603761	1416.116246
PHACTR4	1237.567669	1155.027112	1364.447085	1416.106652	1507.379692	1610.349125
DPF1	121.0771897	104.8209565	93.51933412	64.08130804	73.36296599	61.44101277

ARHGAP5	529.960814	778.6699629	883.7577075	1270.795517	1008.740782	1343.774408
DYSF	22.82602757	4.991474121	11.22232009	33.39448447	40.12037202	31.71149046
MITF	209.403992	215.631682	242.2150754	299.6478066	278.5500115	313.1509683
ATAT1	209.403992	203.6521441	266.5301022	158.8494397	175.3833406	146.6656434
CREB3L4	228.2602757	191.6726063	187.0386682	140.798367	149.0185247	139.7287548
ADAT1	578.590177	654.8814047	625.6443453	496.4044989	453.933352	532.1584493
KCNMB4	50.61423504	40.93008779	47.6948604	18.05107269	21.77963053	26.75657008
TMTC2	230.2451476	242.5856423	240.3446887	314.9912184	317.5240872	305.2230957
IRX4	442.6264476	458.2173243	437.6704837	361.0214538	342.7426067	351.7993473
POLI	134.9712934	143.7544547	145.8901612	185.0234951	202.8944528	209.0976402
SPTLC2	847.540328	961.3579158	1016.555162	809.5906101	722.1666964	766.0306915
ATG16L2	183.6006565	211.6385027	199.1961817	158.8494397	98.58148554	143.6926912

FAM13A	235.2073275	242.5856423	283.3635824	357.4112392	311.7926054	339.9075384
CDYL2	43.66718317	29.94884473	50.50044043	76.71705892	69.92407695	76.30577392
FLYWCH2	310.6324621	327.4407024	280.5580024	245.4945886	224.6740833	218.0164969
ALG1L2	49.62179906	26.95396025	48.63005374	14.44085815	19.48703784	20.81066562
CHMP1B	1018.239317	948.380083	890.3040608	1073.136271	1346.898204	1113.866102
KLC3	1168.09715	1009.276067	884.6929008	771.6833574	859.7222576	821.5257998
SMIM3	11.90923177	5.989768946	16.83348014	31.5893772	38.97407568	23.78361785
CMBL	1383.455758	1379.643447	1272.798137	1119.166507	1179.538937	1098.010357
SCNM1	496.2179906	468.2002726	466.6614773	542.4347343	639.6333597	612.4281595
GKAP1	25.80333551	22.96078096	22.44464019	32.49193084	68.77778061	51.531172
HNRNPR	4977.066446	5365.83468	5119.24835	4470.348151	4611.55019	4407.897174
FECH	366.2088771	354.3946626	356.308663	269.8635367	277.4037151	285.4034142

PRPF6	4166.246249	3631.796571	3534.095636	3110.199824	3381.574213	3104.753113
TSPAN6	772.1151934	829.582999	827.646107	648.9360631	640.779656	703.5986946
IFFO2	2074.191201	2075.45494	1654.357021	2605.672343	2375.126024	2145.480527
EFNB1	2678.584713	2583.587005	2319.279486	2102.949968	2172.231571	2178.183001
C1QTNF3	30.76551542	29.94884473	18.70386682	11.73319725	2.292592687	10.90082485
HBA2	0	0	0	8.12298271	3.438889031	3.963936308
NXPE3	282.8442546	324.4458179	303.0026426	231.0537304	243.0148248	210.0886243
ARFRP1	1993.803886	1747.015942	1604.791774	1446.793476	1578.450065	1381.431803
KIAA1211L	19.84871962	29.94884473	9.351933412	6.317875441	2.292592687	5.945904461
CFAP43	60.53859485	33.94202402	41.14850701	17.14851905	25.21851956	19.81968154
NEK3	229.2527117	235.5975785	240.3446887	287.0120557	333.572236	313.1509683
AL365205.1	18.85628364	18.96760166	31.7965736	6.317875441	5.731481718	9.909840769

TMEM17	19.84871962	23.95907578	15.8982868	39.71235991	44.7055574	43.60329938
SNRNP70	3709.725698	3367.248442	3137.57366	3016.334246	2940.250121	2659.801262
CLEC2D	123.0620617	118.7970841	129.9918744	60.47109351	69.92407695	102.0713599
NUP35	225.2829677	272.534487	279.622809	320.4065402	377.131497	337.9255702
FOXD2	30.76551542	28.9505499	36.47254031	15.34341178	12.60925978	10.90082485
C14orf93	214.3661719	185.6828373	175.8163482	141.7009206	128.3851905	134.7738345
C18orf21	89.31923831	81.86017559	74.8154673	120.0396334	127.2388941	127.8369459
CYP3A5	160.774629	162.7220564	157.1124813	213.0026577	240.7222321	210.0886243
PEF1	979.5343135	795.6409749	899.6559943	703.9918348	768.0185502	696.6618061
CNTNAP2	39.69743925	35.93861367	42.08370036	93.86557798	49.29074277	74.32380577
LRP5	1845.930925	1835.864182	1678.672048	2162.518508	1939.533413	2255.479759
NSUN7	65.50077476	77.86699629	77.62104732	139.8958133	110.044449	100.0893918

REEP5	1833.029257	1729.046636	1693.635141	1505.459462	1483.307469	1501.340877
PALMD	14.88653972	19.96589649	19.63906017	0.902553634	4.585185374	7.927872615
NSUN5	1249.4769	1059.190809	984.7585883	878.1846863	939.9630017	854.2282743
DHRS3	1387.425502	1184.975956	1154.028583	944.0711016	1115.346342	979.092268
FAM199X	1502.548076	1820.889759	1901.248063	2139.052114	2031.237121	2131.606749
VPS37C	625.2346682	601.971779	522.7730777	762.6578211	789.7981807	648.1035863
SRGAP2C	194.5174523	195.6657856	216.9648552	277.9865194	269.3796407	265.5837326
ABCD3	770.1303214	865.5216126	848.2203605	669.6947967	686.6315098	687.7429494
TKT	10195.29484	8976.66706	8700.103653	7688.854412	8467.69109	7634.541329
OSGEP	466.4449112	506.1354759	472.2726373	376.3648655	426.4222398	306.2140798
LLGL2	976.5570055	930.4107762	836.9980404	717.5301394	759.9944758	774.9495481
FAM110B	0.992435981	0	5.611160047	9.025536344	8.024074405	26.75657008

PLEKHH2	339.4131056	440.2480175	439.5408704	542.4347343	478.0055752	558.9150194
ADK	1227.643309	1185.974251	1258.770237	1012.665178	1062.61671	1026.659504
CALHM2	335.4433617	286.5106146	327.3176694	412.4670109	412.6666837	395.4026467
GLUL	1710.959632	1646.188165	1753.487515	1334.876825	1491.331543	1500.349892
SLC36A1	433.6945238	447.2360813	487.2357308	593.8802914	525.0037253	594.5904461
FBXO46	1387.425502	1318.747463	1293.372391	1756.369373	1592.205621	1451.791673
ANO6	836.6235322	991.3067605	1146.547036	1277.113393	1107.322268	1369.539994
TADA1	306.6627182	285.5123197	347.8919229	206.6847823	254.4777883	240.8091307
NPAT	603.4010766	609.9581376	689.2374925	498.2096062	436.7389069	562.8789557
EPS8L2	2778.820747	2518.697842	2322.085066	2152.590418	2136.696384	2167.282176
EFTUD2	2999.141535	2867.102735	2884.136264	3315.079499	3382.72051	3333.670435
MMP24	19.84871962	19.96589649	15.8982868	55.95832533	27.51111224	41.62133123

MRPL33	491.2558107	437.253133	453.5687705	578.5368797	674.02225	514.3207359
AKAP8	885.2528952	900.4619315	812.6830135	1024.398375	1106.175971	999.9029336
ZPR1	891.2075111	788.6529112	830.451687	944.0711016	1145.150047	990.9840769
GRIP1	66.49321074	66.88575323	83.23220737	129.0651697	121.5074124	94.14348731
ST3GAL4	444.6113196	416.2889417	352.5678896	288.817163	286.5740859	344.8624588
CABYR	53.59154299	38.93349815	52.37082711	23.46639449	25.21851956	22.79263377
POLA1	633.174156	652.8848151	674.274399	563.1934679	488.3222423	533.1494334
EIF2S3B	66.49321074	76.86870147	84.16740071	105.5987752	127.2388941	121.8910415
CCDC167	122.0696257	104.8209565	103.8064609	71.30173712	61.90000255	76.30577392
C1orf74	235.2073275	208.6436183	242.2150754	180.5107269	136.4092649	172.4312294
MFN1	1452.926276	1497.442236	1517.818793	1713.046798	1713.713034	1787.735275
SF3A1	3188.696808	3074.748059	3021.609686	3482.954475	3482.448292	3727.091113

SLC44A3	169.7065528	203.6521441	202.0017617	233.7613913	271.6722334	281.4394778
PRC1	72.44782663	67.88404805	57.98198716	50.54300353	27.51111224	22.79263377
TTC13	274.9047668	292.5003835	310.4841893	352.8984711	361.0833482	437.0239779
CTSO	82.37218644	81.86017559	102.8712675	55.95832533	52.7296318	52.52215608
DAGLB	523.0137621	489.1644639	449.8279971	617.3466859	610.9759511	586.6625735
HDGFL2	1536.290899	1366.665614	1334.520898	1212.129531	1229.975977	1080.172644
ELFN1	68.4780827	59.89768946	79.491434	37.90725265	42.41296471	36.66641085
ADO	537.9003018	534.087731	566.7271648	676.9152258	669.4370646	657.022443
RAB2A	2188.321339	2182.272486	2120.083305	1882.726881	1902.85193	1820.437749
TTC34	23.81846355	17.96930684	12.15751344	2.707660903	9.170370748	0.990984077
SUMF2	2221.071726	2066.470286	1931.17425	1688.67785	1875.340818	1685.663915
ELAVL2	790.971477	894.4721625	948.286048	762.6578211	566.2703937	740.2651055

CDCA5	2806.608955	2672.435245	2549.337048	2389.05947	2325.835281	2164.309224
C6orf47	415.8306761	338.4219454	360.9846297	303.2580212	278.5500115	269.5476689
ZNF773	92.29654625	84.85506006	94.45452746	161.5571006	169.6518588	98.10742361
RPL28	17200.90043	14749.80603	14078.40056	16739.66226	19990.26193	18091.40531
TMEM79	199.4796322	132.7732116	196.3906017	248.2022495	233.8444541	265.5837326
ZNF275	224.2905318	275.5293715	303.9378359	364.6316683	366.8148299	336.9345861
ARL8B	1583.927826	1599.268308	1673.060887	1795.179179	2168.792682	1843.230383
PCDHAC2	13.89410374	22.96078096	27.1206069	38.80980628	42.41296471	56.48609238
MFSD4A	15.8789757	14.97442236	16.83348014	5.415321806	4.585185374	0.990984077
PLD3	1633.549625	1395.616164	1311.141064	1149.85333	1243.731533	1188.189908
FAM98C	294.7534864	260.5549491	196.3906017	197.6592459	177.6759332	148.6476115
INHA	8.931923831	9.982948243	12.15751344	1.805107269	1.146296344	0.990984077

ATMIN	887.2377672	892.4755729	914.6190877	976.5630324	1130.248195	1150.532513
MCRIP1	256.0484832	219.6248613	213.2240818	160.6545469	192.5777857	136.7558026
ZNF696	218.3359159	278.524256	299.2618692	207.5873359	179.9685259	188.2869746
PGAP2	589.5069728	503.1405914	534.9305912	485.5738553	395.4722385	386.48379
TMEM192	492.2482467	477.184926	492.8468908	400.7338137	398.9111275	366.6641085
BRK1	2696.448561	2288.091737	2093.897891	1871.896238	2082.820456	1888.815651
OAS1	1329.864215	1201.946968	1237.26079	997.321766	1101.590786	1064.316899
AASDHPPT	1279.24998	1395.616164	1457.031226	1250.036784	1138.272269	1013.776711
PELP1	1779.437714	1649.18305	1679.607241	1470.25987	1482.161172	1412.15231
MOCOS	678.8262112	677.8421857	662.1168856	805.0778419	793.2370697	834.4085928
SLC2A4	23.81846355	20.96419131	21.50944685	2.707660903	3.438889031	12.882793
TMPRSS13	8.931923831	4.991474121	9.351933412	18.05107269	27.51111224	24.77460192

HHLA3	48.62936308	35.93861367	44.88928038	66.78896895	84.82592942	74.32380577
AP3B1	1869.749389	1944.678318	2058.360544	2213.964065	2207.766758	2489.352001
PDZD2	809.8277607	992.3050553	1115.685656	766.2680356	687.7778061	842.3364654
ATP8B3	77.41000653	91.84312383	69.20430725	41.51746718	43.55926105	53.51314015
SPDYA	7.93948785	16.97101201	14.96309346	39.71235991	26.3648159	32.70247454
CKS1B	2717.289717	2329.021825	2245.399212	2055.114626	2095.429716	1988.905042
PTER	531.9456859	648.8916358	636.8666654	510.8453571	440.1777959	486.5731818
FBRSL1	3452.684779	3332.308123	3247.926474	3105.687056	2744.233446	2761.872622
SCARF2	28.78064346	22.96078096	11.22232009	2.707660903	9.170370748	5.945904461
ST6GALNAC2	200.4720682	190.6743114	158.0476747	113.7217579	154.7500064	106.0352962
SLC23A3	0	0.998294824	0	8.12298271	8.024074405	4.954920385
WDTC1	1057.936756	1007.279478	953.8972081	881.7949008	798.9685514	827.4717042

SHISA4	1.984871962	4.991474121	6.546353389	15.34341178	16.04814881	20.81066562
S100A1	8.931923831	9.982948243	8.416740071	26.1740554	25.21851956	23.78361785
BAG6	4502.682047	4136.933752	3899.756233	3668.880524	3660.124225	3453.579508
STK17A	1533.313591	1680.130189	1532.781886	1843.014521	1985.385267	1769.897561
NUP205	2543.61342	2758.288599	2918.738418	2354.762432	2187.133423	2451.694606
ANKFY1	1133.361891	1276.81908	1356.965538	1536.146286	1367.531538	1684.672931
TRAPPC13	314.602206	383.3452125	368.4661764	287.0120557	275.1111224	250.7189715
SLC9A2	3.969743925	7.986358594	5.611160047	25.27150176	20.63333418	13.87377708
IFIH1	427.7399079	416.2889417	441.4112571	577.634326	486.0296497	547.0232105
BAD	653.0228756	634.9155082	500.3284376	486.4764089	502.0777985	377.5649333
GSTO2	424.7626	373.3622643	363.7902097	287.9146094	293.4518639	318.1058887
TRIM6	120.0847537	143.7544547	126.2511011	79.42471983	99.72778189	78.28774208

MAP4	5753.151383	5695.271972	5547.5669	6284.480956	6632.470644	6384.910408
EPN1	5287.698908	4831.746949	4754.522947	4239.294421	4527.870557	4059.070779
ILF3	8868.407928	8537.417337	8576.658132	7862.144709	7638.918833	7427.425656
PEMT	270.9350229	251.5702957	231.9279486	190.4388169	183.407415	185.3140224
ADGRE5	856.4722518	818.6017559	744.4138996	674.2075649	679.7537317	630.2658729
SF3B3	5430.609689	5419.742601	5450.306793	4825.05173	4394.900181	4956.902353
NEMF	362.2391331	412.2957624	426.4481636	509.0402498	443.6166849	607.4732391
PALM2	17.86384766	35.93861367	39.27812033	56.86087897	51.58333546	75.31478985
CORO6	113.1377019	85.85335489	96.32491415	56.86087897	65.33889158	61.44101277
ENTPD1	289.7913065	299.4884473	343.2159562	251.812464	207.4796382	239.8181466
LRRC47	1096.641759	956.3664416	965.1195281	833.9595582	856.2833686	813.5979271
OAS3	3893.326354	4164.886007	4287.86147	4860.251321	4428.142775	5061.946665

RPRML	4.962179906	3.993179297	13.09270678	0.902553634	0	0
SNRNP48	478.3541429	498.1491173	588.2366116	413.3695646	355.3518665	445.9428346
TRAIP	355.2920813	305.4782162	339.4751829	260.8380003	230.405565	269.5476689
MEX3B	21.83359159	12.97783272	17.76867348	32.49193084	36.68148299	48.55821977
PYGB	4547.341666	4165.884302	4111.109928	4804.292996	5164.065028	4825.10147
TUBGCP4	389.0349046	354.3946626	365.6605964	490.0866235	439.0314996	467.7444843
RSL1D1	3803.01468	3496.028475	3895.080266	4079.542428	4749.105751	4279.069244
CDK6	5784.909334	7230.649412	7839.725779	8791.774953	7677.892909	8911.919804
FOXN3	463.4676032	522.1081931	565.7919714	672.4024576	585.7574315	686.7519653
NPIPB12	68.4780827	59.89768946	38.34292699	27.07660903	24.07222321	32.70247454
CYFIP1	2723.244332	2716.360217	2609.189422	2364.690522	2228.400092	2383.316705
TMX3	390.0273406	466.2036829	447.9576104	527.9938761	566.2703937	541.077306

USP51	8.931923831	3.993179297	8.416740071	0	0	0.990984077
OCEL1	123.0620617	134.7698013	122.5103277	84.84004163	84.82592942	84.23364654
CARD9	19.84871962	17.96930684	14.02790012	44.22512809	29.80370493	40.63034715
ZNF575	23.81846355	30.94713955	34.60215363	15.34341178	6.877778061	11.89180892
CSTF1	1378.493578	1200.948674	1266.251784	1031.618804	1106.175971	1091.073469
KTN1	4237.70164	4630.091395	4986.450895	5082.279515	5379.56874	5774.464216
MAPK8	521.0288901	600.9734842	596.6533517	691.356084	693.5092878	723.4183761
TMEM126A	627.2195401	652.8848151	596.6533517	480.1585335	568.5629864	449.9067709
SCN1B	43.66718317	33.94202402	25.25022021	18.05107269	16.04814881	6.936888538
CORO2A	1928.303111	1895.761871	1860.099556	1652.575705	1627.740808	1629.177822
TRIM59	582.559921	569.0280498	631.2555053	500.0147135	481.4444643	461.7985798
RASGEF1B	16.87141168	16.97101201	26.18541355	7.220429075	6.877778061	3.963936308

KRTAP3-1	1.984871962	0.998294824	0	14.44085815	12.60925978	2.972952231
PDZD8	579.582613	800.6324491	735.9971595	828.5442364	866.6000357	988.0111247
ASNSD1	279.8669467	304.4799214	281.4931957	351.9959174	361.0833482	402.3395352
RPS6KA2	69.47051869	81.86017559	45.82447372	107.4038825	130.6777832	89.18856692
ZNF354A	31.7579514	36.9369085	28.99099358	54.15321806	75.65555867	52.52215608
SLC47A2	66.49321074	51.91133086	75.75066064	45.12768172	20.63333418	34.68444269
TRUB1	443.6188836	542.0740896	566.7271648	699.4790667	644.2185451	611.4371755
WASHC5	1050.989704	1238.883877	1188.630737	1324.046182	1376.701909	1460.710529
CAT	1009.307393	1028.243669	960.4435614	907.0664026	753.1166977	811.615959
PPP3CB	630.1968481	694.8131977	675.2095924	873.6719181	845.9667015	732.3372328
ACSS2	1360.62973	1274.822491	1227.908857	1067.72095	1108.468564	1094.046421
C7orf26	294.7534864	242.5856423	211.3536951	351.0933638	348.4740884	304.2321116

PABPN1	1696.073092	1508.423479	1433.651392	1377.296846	1328.557462	889.9037011
GRHPR	1694.08822	1393.619575	1329.844931	1181.442707	1217.366717	1240.712064
ACOT8	775.0925013	627.9274445	578.8846782	482.8661944	552.5148376	523.2395926
CAMK1G	5.954615887	4.991474121	3.740773365	19.85617996	13.75555612	18.82869746
UBA2	2429.483282	2476.769459	2475.456774	2689.609831	2872.618637	2948.177629
KIAA1671	2901.882809	3421.156363	3589.272044	2892.684398	2605.531589	2835.205444
GOT2	3816.908784	3769.561256	3920.330486	3338.545894	3418.255696	3348.535196
KCNC3	123.0620617	104.8209565	96.32491415	157.0443324	166.2129698	147.6566275
BRIP1	714.5539065	941.3920193	937.9989212	704.8943885	530.735207	754.1388825
PTPRS	2211.147366	2299.07298	2236.982472	1991.033318	1818.026001	1998.814883
ST6GALNAC3	0.992435981	4.991474121	0.935193341	8.12298271	13.75555612	16.84672931
FRAT2	200.4720682	213.6350924	209.4833084	137.1881524	177.6759332	131.8008822

ETNK1	696.6900588	765.6921302	815.4885935	1022.593268	884.9407772	878.0118921
ADAM19	64.50833878	62.89257393	85.10259405	42.42002082	41.26666837	37.65739492
TM9SF3	2825.465239	3070.754879	3218.935481	3658.04988	3474.424217	3433.759826
EPB41L2	1593.852186	1765.983544	1826.432595	1929.65967	1983.092674	2262.416648
DUOX2	17.86384766	12.97783272	25.25022021	4.512768172	5.731481718	5.945904461
PLD1	712.5690345	769.6853095	788.3679867	886.307669	941.109298	899.8135418
MBD4	706.6144186	698.806377	692.9782658	847.4978627	808.1389222	859.1831947
CMSS1	659.9699275	687.8251339	610.6812518	764.4629283	841.3815161	774.9495481
TBX6	33.74282336	26.95396025	28.99099358	9.928089978	18.3407415	6.936888538
GTF3C4	1934.257727	2139.345808	2165.907778	1828.573663	1671.300069	1817.464797
HGS	4087.843807	3918.307185	3596.75359	3232.044565	3499.642737	3276.193358
KLHL11	128.0242416	210.6402079	231.9279486	299.6478066	239.5759358	288.3763664

NOP9	559.7338934	693.8149029	577.0142915	540.629627	444.7629813	450.897755
FXR2	1321.924727	1273.824196	1272.798137	1617.376113	1506.233395	1429.990023
SURF4	4891.716951	4565.202231	4444.973951	3946.867043	4220.663137	3923.30596
TMSB10	20367.76364	17361.34529	14214.00359	11975.98417	13355.4987	9330.115084
STAU2	405.9063163	390.3332763	456.3743505	520.7734471	489.4685387	564.8609238
NPC1L1	1.984871962	2.994884473	0.935193341	10.83064361	12.60925978	10.90082485
ATG2A	1234.590361	1170.001534	1135.324716	1311.410431	1446.625986	1425.035103
ABCC4	365.2164411	442.2446071	454.5039638	561.3883606	492.9074277	544.0502582
ZNF484	155.8124491	204.650439	229.1223686	286.1095021	231.5518614	304.2321116
PAOX	39.69743925	38.93349815	44.88928038	20.75873359	20.63333418	17.83771338
AC015802.6	36.7201313	39.93179297	32.73176694	12.63575088	10.31666709	23.78361785
EPHB6	23.81846355	32.9437292	34.60215363	5.415321806	17.19444515	12.882793

RWDD2A	66.49321074	63.89086875	66.39872723	39.71235991	34.38889031	37.65739492
NAA35	879.2982794	960.3596209	907.137541	1093.895005	1099.298193	1064.316899
CRACR2B	128.0242416	101.8260721	102.8712675	80.32727346	51.58333546	75.31478985
REEP4	1408.266657	1215.923096	1132.519136	969.3426034	1103.883379	1017.740647
HOXC4	32.75038738	39.93179297	55.17640713	26.1740554	17.19444515	14.86476115
METTL22	505.1499144	492.1593484	437.6704837	319.5039866	359.9370519	430.0870894
LUZP1	1909.446828	2168.296358	2230.436119	2519.027194	2347.614912	2543.856125
NCAPG	1180.998818	1319.745758	1327.039351	1124.581828	1013.325968	1074.226739
PRKD2	2349.095968	2030.531673	1929.303863	1825.866002	1713.713034	1771.87953
TSEN2	418.8079841	446.2377864	459.1799305	377.2674192	302.6222347	354.7722995
PLEKHA1	809.8277607	933.4056607	995.0457151	772.5859111	672.8759536	777.9225004
KCNAB1	43.66718317	48.91644639	74.8154673	27.07660903	27.51111224	31.71149046

MFSD14B	1843.946053	1979.618637	2071.453251	1672.431885	1697.664885	1689.627851
KAT8	657.9850555	614.9496117	601.3293184	545.1423952	476.8592789	496.4830225
NAA10	427.7399079	352.398073	323.5768961	280.6941803	279.6963078	281.4394778
CCDC137	1110.535863	1012.270952	1034.323835	801.4676274	954.8648541	863.147131
FAM161B	39.69743925	39.93179297	29.92618692	69.49662985	56.16852083	69.36888538
DLAT	1137.331634	1234.890698	1207.334604	1010.860071	863.1611467	1079.18166
RDX	1602.78411	1903.74823	2014.406457	2198.620653	2097.722309	2309.983883
STAU1	3128.158213	3119.671326	3107.647473	3422.483382	3752.974229	3545.741027
AP1M2	1412.236401	1169.003239	1125.037589	993.7115515	1061.470414	994.9480132
GRIN3B	10.91679579	18.96760166	17.76867348	5.415321806	2.292592687	3.963936308
NME7	159.782193	176.6981839	210.4185018	251.812464	254.4777883	237.8361785
FKBP5	246.1241233	240.5890526	257.1781688	200.3669068	177.6759332	176.3951657

SC5D	417.8155481	462.2105036	518.097111	538.8245197	629.3166926	592.608478
CDX2	77.41000653	86.85164971	72.00988727	52.3481108	43.55926105	44.59428346
AASDH	486.2936308	508.1320655	526.5138511	422.3951009	367.9611263	424.1411849
CREBZF	1427.122941	1566.324579	1601.986194	1829.476217	1787.076	1756.023784
NEUROD4	0	0.998294824	0	6.317875441	11.46296344	2.972952231
DNAJC11	961.6704658	937.39884	926.7766012	850.2055236	684.3389171	786.8413571
NUP153	2031.516454	2435.839371	2477.327161	1802.399608	1682.763032	2183.137921
GTF2A2	829.6764803	768.6870147	871.600194	667.8896895	732.4833635	629.2748888
TMOD3	1686.148732	1841.853951	2008.795297	2110.170397	2316.66491	2119.714941
SYNGR1	114.1301378	80.86188076	91.64894744	140.798367	128.3851905	158.5574523
PEX11G	52.599107	33.94202402	22.44464019	18.95362632	9.170370748	15.85574523
MAP3K5	218.3359159	279.5225508	291.7803225	215.7103186	174.2370442	184.3230383

WDR77	1008.314957	952.3732623	966.9899148	919.7021535	706.1185476	748.1929781
AL139353.1	4.962179906	6.98806377	8.416740071	0	0	0.990984077
MFSD4B	110.1603939	113.80561	155.2420946	183.2183878	165.0666735	199.1877995
TEX2	879.2982794	894.4721625	909.0079277	736.4837657	652.2426195	812.6069431
TMBIM6	8597.472905	8314.797591	8383.073111	9389.265459	10276.54672	9186.422393
WDR27	437.6642677	440.2480175	487.2357308	370.9495437	299.1833457	390.4477263
HYAL1	80.38731448	94.8380083	69.20430725	55.0557717	48.14444643	41.62133123
TRAPPC2	167.7216808	167.7135305	141.2141945	203.0745677	251.0388992	206.124688
ELOVL1	1521.404359	1278.81567	1245.677531	1121.874168	1169.22227	1065.307883
ABCC6	30.76551542	13.97612754	24.31502687	9.928089978	2.292592687	8.918856692
EMB	946.7839261	1190.965725	1054.898089	917.8970462	840.2352198	863.147131
ZNF680	92.29654625	47.91815156	72.94508062	51.44555716	28.65740859	31.71149046

SAR1A	1593.852186	1545.360388	1651.551441	1392.640258	1336.581537	1383.413771
ANLN	4327.020878	4780.833913	4986.450895	4073.224552	3693.366819	4282.042196
RPE65	260.0182271	260.5549491	289.9099358	353.8010247	296.890753	429.0961053
ERMARD	347.3525934	353.3963678	333.8640228	287.9146094	276.2574188	230.8992899
KIFAP3	175.6611687	197.6623752	173.9459615	211.1975505	293.4518639	248.7370033
MBTPS2	362.2391331	409.3008779	401.1979434	333.0422911	300.329642	279.4575097
KDM2B	900.139435	960.3596209	905.2671543	755.437392	726.7518818	821.5257998
HSD11B1L	10.91679579	21.96248613	17.76867348	2.707660903	6.877778061	3.963936308
ZNF391	26.79577149	41.92838262	27.1206069	47.83534262	61.90000255	70.35986946
HECTD3	717.5312144	708.7893252	634.9962787	555.9730388	586.9037279	546.0322264
EMD	1123.437531	1003.286298	923.9710211	820.4212537	911.3055931	765.0397074
SDCBP2	55.57641495	43.92497227	35.53734697	15.34341178	24.07222321	25.765586

TMEM101	690.7354429	605.9649583	556.440038	729.2633366	774.8963282	771.9765959
GBP6	33.74282336	41.92838262	42.08370036	22.56384086	12.60925978	18.82869746
WDR54	815.7823766	663.8660581	594.782965	542.4347343	573.1481718	525.2215608
CEP83	222.3056598	306.476511	255.3077822	370.0469901	318.6703835	348.8263951
GLB1L	139.9334734	122.7902634	100.0656875	73.10684439	84.82592942	79.27872615
FAM98B	846.547892	897.467047	962.3139481	731.0684439	784.066699	738.2831373
GMCL1	381.0954168	451.2292606	425.5129703	347.4831492	291.1592713	340.8985225
MYO5B	167.7216808	167.7135305	160.8532547	214.807765	223.527787	230.8992899
ACSL1	315.594642	322.4492282	304.8730292	219.3205332	231.5518614	269.5476689
TMEM128	262.995535	230.6061044	216.9648552	135.3830452	186.846304	189.2779587
TTLL11	52.599107	53.90792051	40.21331367	90.25536344	80.24074405	73.33282169
ATXN7L1	179.6309126	180.6913632	160.8532547	235.5664986	218.9426016	246.7550352

ZNF691	139.9334734	112.8073151	94.45452746	76.71705892	73.36296599	73.33282169
NARF	1198.862665	985.3169915	1004.397648	812.298271	888.3796662	919.6332234
GDPD5	110.1603939	149.7442236	116.8991677	88.45025617	81.38704039	78.28774208
KANSL1L	170.6989888	177.6964787	200.131375	217.5154259	255.6240846	271.5296371
HCAR3	104.205778	84.85506006	70.13950059	152.5315642	118.0685234	123.8730096
NOL12	134.9712934	182.6879528	133.7326478	95.67068525	99.72778189	109.9992325
TCTN2	417.8155481	440.2480175	409.6146835	326.7244157	351.9129775	329.9976976
CHRNG	14.88653972	2.994884473	11.22232009	0.902553634	0	1.981968154
INPP4A	345.3677215	361.3827264	379.6884965	471.1329972	409.2277946	491.5281021
DCAF12L2	30.76551542	31.94543438	39.27812033	13.53830452	16.04814881	15.85574523
HECTD2	144.8956533	139.7612754	161.788448	216.6128723	190.285193	206.124688
IL9R	0.992435981	12.97783272	14.02790012	0.902553634	0	0.990984077

FAM89A	155.8124491	124.786853	126.2511011	94.76813161	81.38704039	97.11643954
MTBP	433.6945238	452.2275554	538.6713645	409.75935	363.3759409	337.9255702
MED28	890.2150751	917.4329435	921.1654411	1022.593268	1141.711158	1074.226739
RAB11FIP1	291.7761785	336.4253558	338.5399895	401.6363673	429.8611288	397.3846148
LRRC1	424.7626	453.2258502	417.0962302	559.5832533	515.8333546	523.2395926
MINK1	3327.637845	3244.458179	3059.952612	2918.858454	2550.509364	2823.313635
VPS37D	21.83359159	21.96248613	14.02790012	42.42002082	56.16852083	27.74755415
SMYD2	913.0411027	831.5795886	798.6551134	972.0502643	1079.811156	999.9029336
AANAT	6.947051869	10.98124307	12.15751344	2.707660903	0	0.990984077
CAPN10	395.9819565	381.3486229	339.4751829	313.1861111	275.1111224	278.4665256
AC136616.1	19.84871962	13.97612754	23.37983353	1.805107269	5.731481718	8.918856692
ZNF627	142.9107813	129.7783272	147.7605479	65.88641531	115.7759307	95.13447138

MYO9B	1668.284884	1815.898285	1609.46774	1463.039441	1333.142648	1517.196622
MUC2	23.81846355	31.94543438	24.31502687	46.03023535	35.53518665	81.26069431
ETS2	6037.98051	6167.465424	5748.633469	6509.216811	6855.998431	7069.680405
POMK	109.1679579	152.7391081	177.6867348	249.1048031	169.6518588	224.9533855
SERPINB7	22.82602757	22.96078096	22.44464019	10.83064361	4.585185374	7.927872615
RAB21	1010.299829	997.2965294	1101.657756	1245.524015	1259.779682	1171.343179
PABPC4L	48.62936308	66.88575323	67.33392057	37.90725265	36.68148299	24.77460192
C15orf41	53.59154299	71.87722735	44.88928038	87.54770254	105.4592636	86.21561469
MMP19	4.962179906	4.991474121	3.740773365	10.83064361	24.07222321	15.85574523
DSTN	3880.424687	3718.64822	3887.598719	3140.886648	3630.32052	3120.608858
FBXO6	185.5855285	173.7032994	148.6957413	102.8911143	114.6296344	135.7648185
PTGR1	2168.472619	1995.591354	2009.73049	1718.46212	1805.416741	1805.572988

DNASE2	702.6446747	674.8473012	649.0241788	541.5321806	614.4148401	481.6182614
RARA	296.7383584	290.5037939	241.279882	341.1652738	357.6444592	372.6100129
RNF139	606.3783845	578.0127032	581.6902582	673.3050113	785.2129953	695.670822
DUSP7	1258.408824	1171.998124	1110.074496	1374.589185	1366.385241	1422.06215
ENOPH1	1166.112278	1104.114076	1146.547036	998.2243197	941.109298	961.2545546
PKDREJ	4.962179906	11.97953789	11.22232009	0	2.292592687	0.990984077
EIF3K	2791.722415	2315.045697	2240.723246	1884.531989	2094.28342	2154.399383
UBAP1L	24.81089953	29.94884473	43.95408704	10.83064361	11.46296344	18.82869746
MRPS28	249.1014313	253.5668854	232.863142	197.6592459	192.5777857	153.6025319
ZSCAN31	51.60667102	27.95225508	33.66696028	68.59407621	74.50926233	60.45002869
TBC1D17	371.171057	364.3776109	364.7254031	433.2257445	510.1018729	440.9879142
ZNRF2	250.0938673	300.4867421	282.4283891	188.6337096	220.088898	219.007481

ZSCAN5A	201.4645042	181.689658	159.9180613	115.5268652	135.2629685	134.7738345
ZNF687	934.8746943	843.5591265	914.6190877	1054.182645	1097.005601	1042.515249
PRDM8	100.2360341	111.8090203	97.26010749	62.27620077	65.33889158	72.34183761
ZNF765	226.2754037	270.5378974	270.2708756	342.0678274	330.1333469	322.069825
E2F3	606.3783845	690.8200184	626.5795386	740.0939802	787.505588	800.7151341
OST4	1078.777912	962.3562106	880.0169341	778.0012329	849.4055905	782.8774208
RPS20	16406.95164	14251.65691	12977.678	11892.04669	13423.13018	11521.18088
ATF7IP	733.4101901	876.5028557	860.3778739	979.2706933	927.3537419	1105.93823
RAB5B	2242.905318	2163.304884	2101.379438	1843.917075	1933.801932	1872.959905
RAP1GAP	129.0166776	107.815841	88.84336742	65.88641531	74.50926233	68.37790131
TMEM231	200.4720682	182.6879528	184.2330882	143.5060279	152.4574137	104.0533281
BCL9	371.171057	426.27189	405.8739101	332.1397375	270.5259371	331.9796658

ARHGEF12	1838.983873	2209.226446	2346.400093	2652.605132	2304.05565	2760.881638
TRAPPC6A	389.0349046	307.4748059	290.8451291	265.3507685	252.1851956	223.9624014
CASC4	402.9290084	441.2463123	463.8558972	518.9683398	526.1500217	578.7347009
RWDD1	502.1726065	468.2002726	525.5786578	602.9058278	604.098173	620.3560321
LACTB2	463.4676032	492.1593484	513.4211443	402.5389209	403.4963129	377.5649333
CFAP97	810.8201967	984.3186967	1180.213997	1312.312984	1127.955602	1291.252252
TOX	66.49321074	64.88916358	66.39872723	36.10214538	34.38889031	43.60329938
MAP9	200.4720682	241.5873475	289.9099358	327.6269693	308.3537164	339.9075384
AKR1B1	1820.12759	1688.116548	1540.263433	1454.013905	1482.161172	1323.954727
IWS1	949.761234	895.4704574	883.7577075	1047.86477	1078.664859	1094.046421
MPC1	153.8275771	142.7561599	118.7695543	165.1673151	247.6000102	186.3050065
ZNF552	186.5779645	200.6572597	191.714635	128.1626161	154.7500064	137.7467867

TOMM20	5823.614338	5842.021312	5932.866557	6539.903635	7072.648439	6392.83828
TMTC4	191.5401444	223.6180406	210.4185018	160.6545469	140.9944503	155.5845001
HASPIN	250.0938673	280.5208456	302.0674492	210.2949968	222.3814906	198.1968154
TTC28	64.50833878	48.91644639	67.33392057	113.7217579	83.67963308	92.16151915
PGM1	1639.504241	1577.305822	1514.078019	1348.41513	1437.455615	1229.811239
RALBP1	1521.404359	1552.348452	1625.366027	1371.881524	1328.557462	1345.756376
POLL	343.3828495	368.3707902	329.1880561	421.4925473	429.8611288	452.8797231
DGUOK	821.7369924	666.8609426	694.8486525	564.9985751	628.1703962	579.725685
CITED2	1133.361891	1096.127717	933.3229545	1244.621462	1396.188946	1185.216956
S100A16	5371.06353	4536.251681	4448.714724	3838.560607	4404.070552	3921.323992
NIPSNAP3A	465.4524752	456.2207347	529.3194311	385.3904019	364.5222372	408.2854397
SSR1	1998.766066	2139.345808	2235.112086	1796.081732	1907.437116	1789.717243

XPO5	1783.407458	1797.928978	1769.385802	2065.945269	2061.040826	2013.679644
PLA2G4F	4.962179906	15.97271719	11.22232009	1.805107269	1.146296344	1.981968154
MYL12A	2283.595193	2059.482222	1791.830442	2278.947927	2722.453816	2387.280641
DAB2	342.3904135	353.3963678	392.7812033	277.0839658	293.4518639	283.421446
SPEF2	52.599107	58.89939463	78.55624066	105.5987752	90.55741114	105.0443122
ZNF441	30.76551542	36.9369085	28.99099358	9.928089978	20.63333418	10.90082485
SELPLG	13.89410374	11.97953789	14.02790012	29.78426994	42.41296471	22.79263377
LHPP	353.3072093	309.4713955	258.1133622	219.3205332	260.20927	206.124688
SLC25A5	12076.95346	10553.97288	10183.32029	9105.863618	10100.01708	8995.162466
GDE1	1007.322521	1073.166936	993.1753284	884.5025617	827.62596	880.9848444
INIP	406.8987523	482.1764001	470.4022506	516.2606789	576.5870608	603.5093028
COL11A2	38.70500327	16.97101201	20.57425351	55.95832533	56.16852083	41.62133123

RBP3	9.924359812	6.98806377	15.8982868	3.610214538	1.146296344	0
SRI	2376.884175	2263.134367	2135.981591	1788.861303	2171.085275	1706.47458
SCD5	256.0484832	254.5651802	270.2708756	194.0490314	198.3092674	202.1607517
YEATS4	963.6553378	985.3169915	1015.619969	811.3957173	870.0389247	825.4897361
AC010325.1	17.86384766	20.96419131	13.09270678	24.36894813	44.7055574	46.57625161
NEK2	1044.042652	1060.189103	922.1006344	828.5442364	805.8463295	889.9037011
DDX55	1300.091135	1270.829311	1324.233771	1058.695413	1070.640785	1183.234988
PANK4	613.3254364	605.9649583	505.0044043	462.1074608	471.1277972	455.8526754
PPP2R3C	200.4720682	228.6095148	202.0017617	272.5711976	311.7926054	252.7009396
PRR15L	3.969743925	10.98124307	19.63906017	0	2.292592687	2.972952231
FAAH	255.0560472	203.6521441	217.9000485	182.3158342	161.6277844	150.6295797
ANKRD20A3	12.90166776	7.986358594	3.740773365	18.05107269	25.21851956	27.74755415

RAD9A	1021.216625	992.3050553	869.7298073	780.7088938	839.0889235	779.9044685
TRAF4	3543.988889	3190.550258	2712.06069	3886.39595	3827.483491	3535.831186
CPTP	595.4615887	508.1320655	465.7262839	385.3904019	461.9574264	387.4747741
STXBP3	530.9532499	609.9581376	654.6353389	712.1148175	825.3333673	688.7339335
NCKAP1	3687.892106	4233.76835	4537.558092	4969.460311	4725.033528	4840.957216
ADGRG5	37.71256729	33.94202402	36.47254031	61.37364714	58.46111352	68.37790131
UPF3A	692.7203149	627.9274445	730.3859995	507.2351425	601.8055803	561.8879716
ACSS1	357.2769532	347.4065988	336.6696028	443.1538345	432.1537215	422.1592168
AMACR	69.47051869	70.87893252	83.23220737	45.12768172	45.85185374	43.60329938
ZC3H7B	2077.168509	2001.581123	1956.42447	2344.834342	2320.103799	2256.470743
IDH3A	414.8382401	442.2446071	453.5687705	383.5852946	310.6463091	339.9075384
ATP7A	152.8351411	206.6470286	194.520215	266.2533222	224.6740833	260.6288122

BRMS1	1803.256178	1431.554778	1469.188739	1847.52729	1959.020451	1822.419717
RBM27	566.6809453	548.0638585	573.2735182	694.0637449	778.3352172	610.4461914
GYS1	2136.714668	1965.642509	1963.906017	1656.185919	1790.514889	1782.780354
PLCB2	41.68231121	30.94713955	38.34292699	15.34341178	21.77963053	14.86476115
ARPC1A	1650.421037	1532.382555	1418.688299	1219.34996	1407.65191	1247.648953
TGFB3	38.70500327	20.96419131	34.60215363	17.14851905	12.60925978	8.918856692
STK11	7.93948785	21.96248613	16.83348014	36.10214538	35.53518665	34.68444269
INTU	220.3207878	260.5549491	228.1871753	305.9656821	276.2574188	349.8173791
ZNF684	76.41757055	43.92497227	72.00988727	30.68682357	28.65740859	44.59428346
IER3IP1	172.6838607	149.7442236	196.3906017	118.2345261	140.9944503	104.0533281
CHCHD5	271.9274589	192.6709011	181.4275082	122.7472943	170.7981552	156.5754842
PRIMPOL	126.0393696	133.7715065	169.2699948	89.35280981	99.72778189	104.0533281

KIAA1147	436.6718317	562.0399861	551.7640713	723.8480148	605.2444694	621.3470162
CCNQ	214.3661719	202.6538493	209.4833084	155.2392251	147.8722283	160.5394205
FCGBP	4.962179906	2.994884473	5.611160047	0	0	0
GDPD1	115.1225738	107.815841	148.6957413	159.7519933	170.7981552	202.1607517
GPSM2	1009.307393	1084.148179	1113.815269	850.2055236	792.0907734	1001.884902
DLEU7	0.992435981	1.996589649	1.870386682	9.928089978	18.3407415	3.963936308
BLVRB	1113.513171	1018.260721	809.8774335	768.0731429	868.8926284	714.4995195
PUS7L	500.1877345	720.7688631	833.257267	549.6551634	461.9574264	539.0953378
NDUFB8	230.2451476	185.6828373	134.6678411	98.37834615	155.8963027	109.9992325
SCML2	81.37975046	91.84312383	111.2880076	158.8494397	134.1166722	126.8459618
CASKIN2	509.1196584	455.2224399	504.0692109	656.1564922	562.8315047	587.6535576
ARPP21	0	0	0	3.610214538	6.877778061	2.972952231

SDCCAG8	224.2905318	196.6640804	247.8262354	259.9354467	306.0611237	323.0608091
KLHL36	852.5025079	1001.289709	904.331961	1164.294188	1053.44634	1075.217723
RPL7L1	3543.988889	3734.620938	3883.857946	4186.043756	4390.314996	4153.214266
CLASRP	1606.753854	1377.646857	1321.428191	1673.334438	1784.783407	1649.988488
LPAR1	673.8640312	700.8029666	689.2374925	539.7270734	479.1518716	641.1666978
GIPR	430.7172158	408.3025831	383.4292699	314.0886648	307.2074201	346.8444269
CBWD6	175.6611687	116.8004944	119.7047477	78.52216619	98.58148554	95.13447138
LRFN1	78.40244252	52.90962569	70.13950059	44.22512809	29.80370493	39.63936308
CPSF2	1603.776546	1645.18987	1826.432595	2007.279283	1866.170447	2058.273928
CLDN7	2417.57405	2118.381617	1991.026623	2663.435775	2997.564938	2259.443695
SLC7A6	379.1105448	440.2480175	461.0503172	557.7781461	461.9574264	606.4822551
PRIM2	608.3632565	559.0451016	595.7181584	524.3836616	449.3481667	456.8436595

GLCCI1	80.38731448	105.8192514	98.19530083	65.88641531	44.7055574	65.40494908
HDAC10	49.62179906	40.93008779	27.1206069	24.36894813	12.60925978	15.85574523
TIMM10B	402.9290084	393.3281608	468.531864	491.8917308	529.5889107	556.9330512
SSX2IP	832.6537882	836.5710627	832.3220737	713.9199248	674.02225	714.4995195
IGHMBP2	504.1574785	475.1883363	476.0134107	398.0261528	409.2277946	369.6370607
NT5M	44.65961915	37.93520332	32.73176694	23.46639449	4.585185374	19.81968154
FAM84A	309.6400261	284.5140249	328.2528628	402.5389209	355.3518665	414.2313441
F12	180.6233486	202.6538493	125.3159077	111.014097	121.5074124	114.9541529
ULBP3	94.28141822	92.84141866	116.8991677	57.7634326	71.0703733	67.38691723
TMEM88	8.931923831	0.998294824	1.870386682	23.46639449	12.60925978	12.882793
COLGALT2	0	0	0	3.610214538	4.585185374	4.954920385
RPS15	11877.47382	10298.40941	9552.064787	8352.231333	9759.567069	8841.559934

CELF5	394.9895205	440.2480175	384.3644632	345.678042	283.1351968	327.0247454
MYH3	58.55372289	68.88234287	79.491434	44.22512809	29.80370493	44.59428346
GRID2IP	4.962179906	3.993179297	10.28712675	0	0	0.990984077
SMIM19	237.1921995	224.6163355	251.5670088	176.9005123	185.7000076	175.4041816
UBQLN1	4006.464056	4383.512573	4573.095439	4872.887072	4835.077977	5166.990977
EDF1	3802.022244	3090.720776	2735.440523	2260.896854	2401.49084	1496.385956
IFT74	420.792856	422.2787107	409.6146835	466.620229	555.9537266	532.1584493
MEFV	23.81846355	12.97783272	17.76867348	39.71235991	35.53518665	39.63936308
ZNF628	433.6945238	441.2463123	342.2807629	500.0147135	544.4907632	492.5190862
BET1L	949.761234	820.5983455	793.0439534	762.6578211	708.4111403	610.4461914
RFLNB	84.3570584	62.89257393	72.00988727	18.95362632	44.7055574	54.50412423
MORN1	208.4115561	210.6402079	216.9648552	172.3877442	143.2870429	153.6025319

NUBP1	210.396428	198.66067	197.325795	163.3622078	155.8963027	118.9180892
YAE1	210.396428	199.6589649	196.3906017	240.0792668	278.5500115	280.4484938
GPR135	34.73525934	31.94543438	40.21331367	16.24596542	20.63333418	12.882793
RSPRY1	471.4070911	517.116719	562.9863914	621.8594541	586.9037279	715.4905035
RP1L1	74.43269859	84.85506006	58.9171805	40.61491355	53.87592815	31.71149046
AL121768.1	1.984871962	4.991474121	5.611160047	18.95362632	14.90185247	12.882793
RFX2	240.1695075	272.534487	229.1223686	317.6988793	328.9870506	310.1780161
GTPBP8	153.8275771	140.7595702	138.4086145	180.5107269	205.1870455	206.124688
ARL2BP	89.31923831	108.8141358	88.84336742	70.39918348	60.75370621	49.54920385
TM2D1	359.2618252	350.4014833	333.8640228	398.9287064	495.2000204	426.1231531
YTHDF2	1599.806802	1734.03811	1738.524421	1440.475601	1462.674134	1476.566275
6-Mar	4009.441364	4447.403442	4354.260197	4923.430076	4761.715011	4895.46134

FAM160B2	1057.936756	1074.165231	922.1006344	866.451489	881.5018882	804.6790705
TMPRSS5	20.84115561	14.97442236	20.57425351	8.12298271	3.438889031	5.945904461
ZBED8	240.1695075	252.5685905	251.5670088	211.1975505	167.3592662	178.3771338
TMEM230	1453.918712	1409.592292	1433.651392	1192.273351	1270.096349	1241.703048
GAS7	24.81089953	21.96248613	28.05580024	37.00469901	50.43703911	57.47707646
TBL2	948.768798	838.5676524	834.1924604	753.6322847	732.4833635	700.6257424
LTBP2	196.5023243	189.6760166	192.6498283	147.1162424	123.8000051	150.6295797
PTPMT1	94.28141822	95.83630313	96.32491415	66.78896895	55.02222449	62.43199685
RNF216	1230.620617	1090.137948	1110.074496	1406.178562	1297.607461	1328.909647
ZNF526	276.8896388	360.3844316	355.3734697	259.0328931	249.8926029	251.7099555
SMIM8	168.7141168	189.6760166	197.325795	120.942187	147.8722283	133.7828504
DNAJC19	826.6991724	777.6716681	721.9692594	536.1168588	712.9963257	632.2478411

1-Mar	144.8956533	150.7425185	158.982868	121.8447406	77.94815136	109.9992325
TMEM199	186.5779645	146.7493392	118.7695543	191.3413705	239.5759358	208.1066562
CRMP1	411.8609322	424.2753003	386.2348499	305.9656821	343.8889031	325.0427772
MGRN1	800.8958368	768.6870147	722.9044528	629.0798832	576.5870608	684.7699971
CARNMT1	554.7717135	734.7449907	708.8765526	578.5368797	476.8592789	529.1854971
MARVELD3	371.171057	298.4901525	327.3176694	261.740554	255.6240846	255.6738918
SLC25A6	4788.503609	3875.380508	3346.121775	4902.671342	5576.731711	4337.537305
FZD10	26.79577149	19.96589649	28.99099358	11.73319725	11.46296344	5.945904461
VPS4B	494.2331186	556.0502171	613.4868318	668.7922431	680.900028	684.7699971
GPC6	28.78064346	27.95225508	17.76867348	54.15321806	38.97407568	50.54018792
SGCG	0.992435981	0	0	9.025536344	8.024074405	1.981968154
CASD1	212.3813	258.5583595	329.1880561	375.4623119	337.011125	353.7813155

FZD9	30.76551542	21.96248613	31.7965736	9.025536344	8.024074405	16.84672931
PCSK1	46.64449112	37.93520332	42.08370036	21.66128723	25.21851956	16.84672931
SLIT2	8.931923831	6.98806377	16.83348014	37.00469901	22.92592687	23.78361785
PSMB4	4490.772815	3937.274787	3579.92011	3408.945077	3465.253846	3391.147511
NIPAL1	369.186185	372.3639694	396.5219767	432.3231909	498.6389094	485.5821977
AC007325.2	36.7201313	44.92326709	28.99099358	19.85617996	14.90185247	16.84672931
ZNF805	162.7595009	210.6402079	216.9648552	240.0792668	260.20927	295.3132549
SHTN1	666.9169794	729.7535165	822.9701403	844.7902018	880.3555918	975.1283317
OTUB1	3110.294365	2684.414782	2586.744782	2289.77857	2418.685285	2459.622479
NLGN1	25.80333551	28.9505499	33.66696028	62.27620077	52.7296318	46.57625161
FOXJ2	733.4101901	834.5744731	805.2014668	658.8641531	593.7815059	703.5986946
CHMP4B	3336.569769	3094.713955	2900.034551	2510.001657	2858.863081	2662.774215

PIK3C3	283.8366906	305.4782162	335.7344095	344.7754883	449.3481667	399.366583
AP5B1	615.3103084	610.9564324	623.7739586	726.5556757	729.0444745	752.1569144
CASP8AP2	517.0591462	637.9103927	708.8765526	539.7270734	388.5944605	509.3658155
TMEM56	183.6006565	252.5685905	253.4373955	302.3554675	309.5000127	301.2591594
CLDN16	49.62179906	62.89257393	70.13950059	89.35280981	114.6296344	86.21561469
ANKHD1	85.34949438	74.87211182	96.32491415	128.1626161	104.3129673	151.6205638
SH2D4A	614.3178724	602.9700739	612.5516385	672.4024576	773.7500319	761.0757711
PLCH1	294.7534864	292.5003835	304.8730292	218.4179795	191.4314894	265.5837326
SEMA7A	121.0771897	157.7305822	160.8532547	119.1370797	92.85000382	86.21561469
VRK3	374.1483649	350.4014833	333.8640228	429.61553	420.6907581	468.7354684
CCDC18	258.0333551	293.4986783	363.7902097	238.2741595	191.4314894	248.7370033
IQSEC1	481.3314509	468.2002726	475.0782173	397.1235991	303.768531	417.2042964

M6PR	2103.96428	2054.490748	1998.50817	1786.153642	1785.929703	1806.563972
FBXO34	495.2255546	548.0638585	543.3473313	667.8896895	632.7555816	620.3560321
SYNPO2L	5.954615887	9.982948243	7.48154673	0.902553634	1.146296344	0
SPACA6	150.8502691	135.7680961	126.2511011	103.793668	95.14259651	86.21561469
HBS1L	962.6629018	975.3340433	1052.092509	891.7229908	786.3592917	837.381545
SMTN	1887.613236	1704.089265	1513.142826	1383.614722	1382.43339	1524.13351
TBCE	81.37975046	81.86017559	95.38972081	126.3575088	128.3851905	122.8820255
LBHD1	115.1225738	106.8175462	93.51933412	64.08130804	48.14444643	86.21561469
NAP1L2	68.4780827	74.87211182	52.37082711	42.42002082	38.97407568	30.72050638
SRCIN1	14.88653972	16.97101201	13.09270678	35.19959174	35.53518665	27.74755415
VPS26A	1190.923177	1147.040753	1371.928632	1403.470902	1724.029701	1373.503931
PPIL1	720.5085224	675.845596	709.811746	576.7317724	567.41669	610.4461914

SLC3A2	20057.13118	17755.67174	17236.54847	21693.77916	22882.36761	19324.1895
COX7B2	121.0771897	96.83459795	88.84336742	81.2298271	52.7296318	58.46806054
ZFP69B	11.90923177	20.96419131	9.351933412	1.805107269	3.438889031	4.954920385
WDR44	541.8700457	701.8012615	632.1906987	725.6531221	772.6037355	803.6880864
ZNF251	191.5401444	135.7680961	165.5292214	94.76813161	119.2148197	127.8369459
RAE1	1701.035272	1513.414954	1593.569453	1861.968148	1837.513039	1858.095144
TRIM56	3767.286985	4083.025831	4111.109928	4754.652546	4469.409443	4406.90619
NPY4R2	8.931923831	3.993179297	5.611160047	0	0	0.990984077
CCDC197	8.931923831	3.993179297	5.611160047	0	0	0.990984077
RTN4IP1	224.2905318	237.5941682	187.9738616	138.9932597	165.0666735	172.4312294
CAPN1	12364.75989	11107.02821	10533.0826	10081.5241	10142.43005	9423.267587
NEK5	0	6.98806377	1.870386682	10.83064361	13.75555612	16.84672931

SAMD12	152.8351411	154.7356978	169.2699948	213.0026577	189.1388967	243.7820829
RALYL	0	0	0.935193341	1.805107269	9.170370748	7.927872615
REXO1	842.5781481	851.5454851	770.5993132	1003.639641	990.4000408	925.5791278
CAP1	3795.075192	3716.651631	3516.326963	3339.448447	3169.50939	3192.950696
FAHD1	418.8079841	388.3366866	423.6425836	329.4320766	311.7926054	343.8714747
RFT1	595.4615887	540.0774999	557.3752314	452.1793708	440.1777959	492.5190862
C9orf72	381.0954168	406.3059935	409.6146835	347.4831492	285.4277895	314.1419524
MMADHC	1843.946053	1764.985249	1745.070775	2082.191235	2187.133423	1925.482061
THUMPD3	772.1151934	794.6426801	884.6929008	937.7532261	1011.033375	967.2004591
SMCHD1	949.761234	1152.032227	1421.493879	1470.25987	1321.679684	1619.267982
KANSL2	606.3783845	622.9359703	646.2185988	519.8708934	486.0296497	543.0592741
MTMR14	650.0455677	508.1320655	533.9953978	695.8688521	769.1648465	638.1937455

LEO1	537.9003018	594.9837153	635.931472	672.4024576	724.4592891	750.1749462
IARS2	3465.586446	3657.752236	3585.53127	3186.916883	3048.001977	3210.788409
MORF4L1	3457.646959	3251.446243	3371.371995	2833.115858	3198.166798	2659.801262
GALT	359.2618252	244.5822319	300.1970625	228.3460695	233.8444541	216.0345288
PHAX	738.37237	716.7756838	742.5435129	805.0778419	1046.568562	837.381545
RRM2B	380.1029808	503.1405914	460.1151239	585.7573087	550.2222449	541.077306
LIN54	333.4584897	433.2599537	505.0044043	529.7989834	537.6129851	565.8519079
ARFIP2	2215.11711	1929.703895	1919.95193	2390.864578	2425.563063	2238.63303
SRXN1	131.9939855	128.7800323	133.7326478	101.9885607	80.24074405	89.18856692
TAF1C	1192.908049	1175.991303	1094.176209	966.6349425	958.3037432	1026.659504
FNBP4	2298.481732	2494.738766	2568.976108	2789.793284	2747.672335	2905.565314
ARL4D	775.0925013	619.9410859	544.2825246	542.4347343	497.4926131	493.5100703

FARS2	288.7988705	227.6112199	217.9000485	192.2439241	178.8222296	174.4131975
AKTIP	234.2148916	214.6333872	200.131375	276.1814121	286.5740859	278.4665256
TSN	1425.138069	1459.507033	1510.337246	1230.180604	1328.557462	1229.811239
CYB561	2006.705554	1997.587943	1897.507289	1749.148943	1771.027851	1582.601571
PCYOX1L	235.2073275	195.6657856	209.4833084	140.798367	134.1166722	188.2869746
IL15RA	432.7020878	409.3008779	366.5957898	318.6014329	292.3055676	343.8714747
ZNF280B	206.4266841	268.5413077	238.474302	199.4643532	149.0185247	174.4131975
MSRB2	150.8502691	178.6947735	151.5013213	115.5268652	121.5074124	106.0352962
PMPCB	2294.511989	2330.02012	2210.797059	2476.607173	2763.720484	2602.324186
CCDC90B	565.6885093	541.0757947	501.2636309	592.0751842	729.0444745	653.0585067
EXOC1	1147.255994	1210.931622	1278.409297	1331.266611	1429.43154	1504.313829
SLC25A35	30.76551542	54.90621533	46.75966706	23.46639449	16.04814881	25.765586

TMEM8B	121.0771897	101.8260721	93.51933412	79.42471983	64.19259524	62.43199685
TMEM117	99.24359812	71.87722735	54.24121379	36.10214538	41.26666837	52.52215608
TPPP	67.48564672	78.86529112	51.43563377	40.61491355	40.12037202	33.69345861
RASSF8	323.5341299	368.3707902	387.1700433	470.2304435	416.1055727	462.7895639
ARHGAP10	168.7141168	194.6674907	174.8811548	194.951585	264.7944554	273.5116052
TXLNA	2435.437898	2356.97408	2383.807827	2065.945269	2087.405642	2150.435447
CMTR1	990.4511093	983.3204019	1006.268035	863.7438281	821.8944783	855.2192584
CSGALNACT1	88.32680233	109.8124307	80.42662735	134.4804915	137.5555612	131.8008822
GBGT1	0	0.998294824	0	9.928089978	2.292592687	5.945904461
HPF1	365.2164411	369.369085	375.0125298	240.0792668	330.1333469	294.3222708
SOWAHD	23.81846355	10.98124307	15.8982868	1.805107269	6.877778061	5.945904461
8-Sep	1095.649323	1187.970841	1105.398529	947.6813161	967.4741139	982.0652202

VKORC1	113.1377019	86.85164971	60.78756718	64.08130804	49.29074277	39.63936308
USP36	1944.182087	1840.855656	1791.830442	2126.416363	2208.913054	2068.183769
ARSB	170.6989888	155.7339926	200.131375	136.2855988	77.94815136	140.7197389
ANKRD63	3.969743925	4.991474121	3.740773365	0	0	0
EIF3G	2283.595193	1915.727768	1938.655796	1648.062936	1870.755633	1686.654899
RPGR	134.9712934	151.7408133	144.0197745	203.9771214	184.5537113	193.241895
PRDM2	559.7338934	540.0774999	591.977385	474.7432117	449.3481667	468.7354684
ETHE1	907.0864868	719.7705683	685.4967191	601.1007205	691.2166951	559.9060035
MORC4	1172.066894	1151.033932	1288.696424	1002.737088	1013.325968	1064.316899
TNFSF8	2.977307944	0	0	9.928089978	6.877778061	8.918856692
KTI12	110.1603939	105.8192514	97.26010749	80.32727346	68.77778061	55.49510831
RPP21	14.88653972	23.95907578	21.50944685	9.025536344	5.731481718	5.945904461

YME1L1	2183.359159	2426.854718	2490.419868	2770.839658	2790.0853	2618.179931
PPP1R7	683.7883911	604.9666635	636.8666654	457.5946926	590.3426169	515.31172
FKBP1C	211.388864	200.6572597	190.7794416	120.0396334	145.5796356	172.4312294
MANEA	218.3359159	296.4935628	283.3635824	227.4435159	144.4333393	202.1607517
ALK	1.984871962	0	1.870386682	9.025536344	8.024074405	9.909840769
TMEM242	238.1846355	234.5992837	235.668722	307.7707893	307.2074201	289.3673505
C12orf54	28.78064346	23.95907578	12.15751344	7.220429075	10.31666709	4.954920385
CXorf38	328.4963098	343.4134195	303.0026426	246.3971422	261.3555663	258.6468441
NT5DC1	471.4070911	467.2019778	541.4769446	386.2929555	429.8611288	379.5469015
PIGC	427.7399079	477.184926	453.5687705	515.3581252	615.5611365	542.0682901
APH1A	2760.9569	2455.805268	2380.067053	2191.400224	2310.933429	2010.706692
ABCA13	152.8351411	242.5856423	222.5760152	157.0443324	134.1166722	146.6656434

CCNG1	867.3890476	917.4329435	997.8512951	1079.454147	1191.001901	1037.560329
MGAT4A	17.86384766	26.95396025	13.09270678	52.3481108	36.68148299	31.71149046
GSS	2769.888824	2580.592121	2378.196667	2065.945269	2281.129724	2305.028963
FKTN	437.6642677	521.1098983	509.680371	642.6181877	554.8074303	604.5002869
SLC16A6	16.87141168	18.96760166	24.31502687	8.12298271	8.024074405	4.954920385
ESR1	26.79577149	39.93179297	20.57425351	52.3481108	52.7296318	55.49510831
ZBTB3	108.175522	103.8226617	102.8712675	74.91195166	77.94815136	54.50412423
HIST1H4E	20.84115561	11.97953789	29.92618692	40.61491355	45.85185374	41.62133123
TBC1D2B	421.785292	487.1678742	469.4670573	427.8104227	272.8185298	352.7903314
ENG	454.5356794	381.3486229	361.9198231	583.0496478	431.0074252	511.3477837
RIPK2	396.9743925	386.340097	401.1979434	473.8406581	474.5666862	500.4469588
CSDE1	10654.79269	11007.19873	11511.29484	11929.05139	12776.61904	12596.3986

ANKRD36C	137.9486014	106.8175462	127.1862944	91.15791708	52.7296318	96.12545546
ASTN1	34.73525934	41.92838262	47.6948604	8.12298271	14.90185247	32.70247454
EIF3E	7678.477187	7664.907661	7922.957987	8113.957173	9613.987433	8866.334536
SH2B1	911.0562308	829.582999	831.3868803	759.9501602	716.4352147	683.7790131
ATP8A1	28.78064346	37.93520332	33.66696028	56.86087897	50.43703911	68.37790131
TFIP11	649.0531317	636.9120979	703.2653926	490.0866235	562.8315047	584.6806054
CCDC171	40.68987523	26.95396025	42.08370036	58.66598624	67.63148427	63.42298092
AL031777.3	8.931923831	10.98124307	10.28712675	23.46639449	18.3407415	33.69345861
PIANP	16.87141168	5.989768946	10.28712675	0	0	4.954920385
ZDHHC4	516.0667102	497.1508225	502.1988242	397.1235991	459.6648338	370.6280448
STOML1	122.0696257	133.7715065	129.0566811	103.793668	76.80185502	82.25167838
AADAT	94.28141822	84.85506006	93.51933412	120.0396334	143.2870429	129.8189141

LRRC14	526.983506	481.1781053	432.0593236	412.4670109	387.4481641	352.7903314
NLRC4	2.977307944	3.993179297	12.15751344	14.44085815	19.48703784	26.75657008
VPS51	2184.351595	1897.758461	1804.923149	2336.711359	2360.224171	2184.128906
RABL2B	359.2618252	336.4253558	317.0305427	398.9287064	481.4444643	396.3936308
RBM45	173.6762967	216.6299769	238.474302	149.8239033	151.3111173	160.5394205
PSMD7	1271.310492	1237.885582	1284.020458	1443.183261	1506.233395	1428.008055
OGFRL1	663.9396714	804.6256284	837.9332337	948.5838698	880.3555918	955.3086501
ANKK1	4.962179906	0.998294824	1.870386682	12.63575088	5.731481718	18.82869746
NOXA1	198.4871962	161.7237615	162.7236414	143.5060279	115.7759307	113.9631688
CEP128	208.4115561	209.6419131	192.6498283	243.6894813	255.6240846	300.2681753
CFAP298	223.2980958	188.6777218	216.0296618	134.4804915	166.2129698	164.5033568
WDR92	73.44026261	89.84653418	77.62104732	51.44555716	45.85185374	53.51314015

ANXA11	2703.395613	2326.026941	2353.88164	2125.513809	2214.644536	2016.652597
BPNT1	1004.345213	978.3289278	984.7585883	843.8876482	800.1148478	881.9758285
DARS	1707.982324	1744.021058	1766.580222	1947.710743	2050.724159	1966.112409
MSH5-SAPCD1	33.74282336	20.96419131	32.73176694	12.63575088	16.04814881	8.918856692
CIZ1	2886.996269	2653.467643	2539.985115	2203.133422	2446.196397	2356.560135
PIN4	233.2224556	172.7050046	200.131375	142.6034742	176.5296369	109.9992325
CNKSR2	23.81846355	23.95907578	28.99099358	43.32257445	55.02222449	44.59428346
ATXN7L3B	1139.316506	1162.015175	1187.695543	1446.793476	1341.166722	1276.387491
UVSSA	433.6945238	536.0843206	495.6524708	592.0751842	592.6352096	605.491271
C11orf91	15.8789757	19.96589649	30.86138026	9.025536344	10.31666709	4.954920385
SEC23IP	755.2437817	766.690425	781.8216333	870.9642572	900.988926	940.443889
RPS29	6671.154666	4935.569611	3584.596077	3131.861111	3481.301995	2479.44216

LENG9	265.972843	224.6163355	217.9000485	166.9724224	210.9185272	142.7017071
CSMD3	15.8789757	17.96930684	27.1206069	55.0557717	38.97407568	30.72050638
CARM1	1315.970111	1303.77304	1280.279684	1137.217579	1075.22597	1152.514481
TEAD4	579.582613	576.0161136	524.6434644	419.68744	449.3481667	501.4379429
S100A4	69.47051869	54.90621533	47.6948604	27.97916267	28.65740859	39.63936308
CCDC6	1113.513171	1269.831016	1255.029464	1378.1994	1456.942653	1411.161326
C6	0	0.998294824	2.805580024	3.610214538	16.04814881	8.918856692
CNTNAP3	192.5325804	349.4031885	296.4562892	236.4690522	165.0666735	185.3140224
TUBD1	277.8820747	280.5208456	319.8361227	206.6847823	218.9426016	251.7099555
ZBTB37	159.782193	226.6129251	252.5022021	293.3299312	252.1851956	323.0608091
BICRA	462.4751672	419.2838262	347.8919229	517.1632325	523.857429	493.5100703
STX10	1285.204596	1110.103845	952.9620147	935.0455652	884.9407772	940.443889

ZNF837	26.79577149	45.92156192	25.25022021	16.24596542	18.3407415	7.927872615
ENAH	1591.867314	1748.014237	1832.978949	1982.910335	1970.483415	2017.643581
CSPP1	398.9592644	469.1985674	506.8747909	578.5368797	502.0777985	641.1666978
SYTL5	9.924359812	12.97783272	16.83348014	2.707660903	4.585185374	2.972952231
XKR6	71.45539065	50.91303604	47.6948604	110.1115434	90.55741114	72.34183761
SAGE1	34.73525934	44.92326709	72.94508062	71.30173712	95.14259651	91.17053508
MAPK11	131.9939855	98.8311876	86.97298073	71.30173712	68.77778061	67.38691723
MRPL47	1069.845988	1081.153295	1071.731569	1181.442707	1303.338943	1263.504698
EFEMP2	53.59154299	42.92667744	55.17640713	74.91195166	71.0703733	98.10742361
NELFA	948.768798	773.6784888	825.7757203	1060.50052	1031.666709	955.3086501
DVL1	3188.696808	2777.256201	2540.920308	2536.175713	2439.318619	2262.416648
CDH8	131.0015495	134.7698013	165.5292214	197.6592459	174.2370442	218.0164969

TUSC3	1307.038187	1264.839542	1213.880957	1083.966915	1110.761157	1070.262803
CLCC1	298.7232303	303.4816266	337.6047962	371.8520974	362.2296446	460.8075958
C2CD2L	191.5401444	163.7203512	164.5940281	244.5920349	230.405565	213.0615765
ELF3	1156.187918	1070.172052	1171.797257	1297.872126	1417.968577	1258.549778
HS6ST2	577.5977411	631.9206238	629.3851186	526.1887689	486.0296497	512.3387678
FKBP8	4516.576151	3818.477703	3401.298182	3428.801257	3393.037177	3073.041623
ASB9	61.53103084	47.91815156	38.34292699	24.36894813	32.09629762	22.79263377
NRDC	2985.247431	2977.913461	3018.804105	2670.656204	2588.337144	2695.476689
CCPG1	57.56128691	52.90962569	68.26911391	92.06047071	91.70370748	91.17053508
NKAPD1	452.5508074	400.3162245	457.3095439	518.9683398	576.5870608	511.3477837
SAMHD1	1344.750755	1266.836132	1414.947525	1534.341178	1531.451915	1589.538459
C3orf33	0	0	0.935193341	9.928089978	5.731481718	1.981968154

IL12A	32.75038738	31.94543438	27.1206069	15.34341178	11.46296344	14.86476115
PTTG1IP	2600.182271	2745.310767	2735.440523	3037.09298	3402.207548	2879.799728
PRR29	1.984871962	1.996589649	0.935193341	5.415321806	11.46296344	11.89180892
ABCG2	67.48564672	73.87381699	86.97298073	47.83534262	33.24259396	56.48609238
CDKL1	94.28141822	88.84823936	97.26010749	69.49662985	43.55926105	65.40494908
ATP6AP1L	40.68987523	50.91303604	45.82447372	22.56384086	28.65740859	22.79263377
RPP14	263.987971	231.6043992	281.4931957	354.7035783	307.2074201	329.9976976
UBE2I	1656.375653	1601.264898	1609.46774	1339.389593	1516.550062	1347.738345
CIITA	19.84871962	18.96760166	28.05580024	9.928089978	10.31666709	4.954920385
DCUN1D4	1002.360341	1129.071446	1208.269797	1277.113393	1296.461165	1357.648185
TREM1	1.984871962	0	0	8.12298271	9.170370748	4.954920385
RNF149	885.2528952	889.4806884	915.5542811	754.5348384	764.5796611	775.9405322

FASTKD3	358.2693892	423.2770055	369.4013698	307.7707893	331.2796433	270.538653
EFNA4	189.5552724	159.7271719	163.6588347	141.7009206	121.5074124	104.0533281
USP42	520.0364542	590.990536	645.2834054	683.2331012	671.7296573	793.7782456
PIK3CA	592.4842808	795.6409749	854.7667139	982.8809079	843.6741088	966.209475
CNNM1	229.2527117	237.5941682	248.7614288	183.2183878	160.4814881	199.1877995
EPAS1	1412.236401	1569.319464	1592.63426	1775.322999	1707.981552	1800.618068
PRRC1	1086.717399	1247.86853	1356.030345	1102.920541	1022.496338	965.2184909
PRMT3	918.0032826	971.340864	1002.527262	805.9803955	832.2111454	829.4536724
ISG20	488.2785028	442.2446071	386.2348499	539.7270734	592.6352096	501.4379429
PSMC2	4007.456492	3718.64822	3630.420551	3277.172247	3399.914955	3344.57126
SEMA4D	449.5734995	402.3128142	413.3554568	336.6525056	312.9389018	366.6641085
CCDC102B	15.8789757	25.95566543	20.57425351	39.71235991	34.38889031	48.55821977

NCAM2	82.37218644	87.84994453	85.10259405	112.8192043	112.3370417	146.6656434
RNF215	299.7156663	268.5413077	230.0575619	221.1256404	173.0907479	206.124688
TRIM27	1634.542061	1490.454173	1560.837687	1713.046798	1863.877855	1822.419717
MPPE1	160.774629	162.7220564	152.4365146	111.014097	128.3851905	105.0443122
HEBP2	466.4449112	456.2207347	520.9026911	575.8292188	584.6111352	585.6715895
DFFB	160.774629	141.757865	173.0107681	111.9166507	93.99630017	130.8098982
ZNF880	11.90923177	3.993179297	11.22232009	0.902553634	2.292592687	0.990984077
ATP6V1E2	80.38731448	95.83630313	81.36182069	121.8447406	131.8240795	117.9271052
RAB40C	519.0440182	532.0911413	414.2906502	399.83126	402.3500166	370.6280448
PDK4	35.72769532	38.93349815	43.95408704	52.3481108	68.77778061	79.27872615
HLA-DOB	38.70500327	28.9505499	36.47254031	11.73319725	9.170370748	25.765586
RPS10	84.3570584	69.8806377	72.00988727	47.83534262	34.38889031	55.49510831

GANC	134.9712934	131.7749168	124.3807144	93.86557798	95.14259651	86.21561469
MBTD1	557.7490214	773.6784888	654.6353389	854.7182918	763.4333648	823.5077679
ZNF83	225.2829677	233.6009889	245.0206554	184.1209414	192.5777857	159.5484364
RNF123	671.8791593	631.9206238	627.514732	778.9037865	732.4833635	775.9405322
DTX4	378.1181088	423.2770055	358.1790497	357.4112392	257.9166773	282.4304619
SFSWAP	1831.044385	1681.128484	1715.144588	1943.197975	1999.140823	2039.44523
INTS11	2309.398528	2112.391848	2060.230931	1865.578362	1981.946378	1769.897561
ST3GAL3	72.44782663	61.8942791	66.39872723	42.42002082	40.12037202	39.63936308
VASN	53.59154299	54.90621533	52.37082711	34.29703811	26.3648159	30.72050638
CFD	825.7067364	609.9581376	502.1988242	534.3117516	459.6648338	498.4649907
PRKAB1	623.2497962	583.0041774	619.0979919	702.1867276	738.2148452	727.3823125
PSTPIP1	46.64449112	24.95737061	23.37983353	59.56853987	51.58333546	60.45002869

MYL9	51.60667102	42.92667744	30.86138026	76.71705892	65.33889158	66.39593315
ENY2	1066.86868	923.4227124	903.3967676	812.298271	868.8926284	732.3372328
OGG1	234.2148916	227.6112199	177.6867348	159.7519933	162.7740808	152.6115478
C8orf59	523.0137621	517.116719	529.3194311	466.620229	435.5926105	376.5739492
SCYL1	2109.918896	1932.69878	1837.654916	2209.451297	2260.496389	2281.245345
DBNDD1	99.24359812	94.8380083	81.36182069	137.1881524	138.7018576	117.9271052
VAV3	9.924359812	14.97442236	14.02790012	19.85617996	34.38889031	34.68444269
LRRC42	1003.352777	983.3204019	948.286048	776.1961256	909.0130004	801.7061182
AC011448.1	9.924359812	3.993179297	6.546353389	24.36894813	16.04814881	17.83771338
DRAM2	421.785292	453.2258502	430.188937	527.9938761	523.857429	525.2215608
ZNF639	1060.914064	1088.141358	1082.018696	1216.642299	1212.781531	1313.053902
RBMX	3031.891923	3201.531501	3430.289176	3620.142628	3726.609413	3630.965658

MCTS1	1157.180354	1213.926506	1151.223003	984.6860151	1066.055599	980.0832521
TXNDC9	536.9078658	553.0553326	556.440038	661.571814	659.1203975	639.1847296
MRPS22	723.4858303	751.7160027	718.2284861	825.8365755	898.6963333	857.2012265
PEX11B	348.3450294	275.5293715	303.9378359	253.6175713	243.0148248	224.9533855
RTCB	2886.003833	2632.503452	2526.892408	2330.393484	2459.951953	2198.002683
NMU	172.6838607	142.7561599	146.8253546	111.014097	105.4592636	115.945137
CMTM8	77.41000653	85.85335489	56.11160047	115.5268652	115.7759307	98.10742361
VAMP7	465.4524752	454.224145	437.6704837	546.9475025	560.538912	529.1854971
SMU1	2141.676847	2031.529967	2016.276844	1746.441283	1801.977852	1873.950889
BUB3	2608.121759	2473.774575	2539.049921	2268.117283	2258.203797	2207.912523
FMNL3	209.403992	219.6248613	216.9648552	262.6431076	265.9407517	300.2681753
FBXL22	16.87141168	14.97442236	19.63906017	4.512768172	6.877778061	5.945904461

PRSS53	51.60667102	52.90962569	33.66696028	29.78426994	18.3407415	23.78361785
GOLGA8B	589.5069728	655.8796995	585.4310316	755.437392	718.7278074	706.5716468
NCDN	864.4117396	786.6563215	793.0439534	731.9709975	675.1685463	649.0945704
MMD	224.2905318	210.6402079	253.4373955	175.9979587	153.60371	189.2779587
CHMP2A	2715.304845	2231.188932	2111.666564	1911.608598	2090.844531	1975.031265
PGAP1	436.6718317	559.0451016	564.8567781	465.7176754	361.0833482	406.3034715
PHC3	968.6175177	1167.00665	1187.695543	1361.953434	1195.587086	1405.215421
NMNAT3	105.198214	86.85164971	61.72276052	113.7217579	130.6777832	132.7918663
PTPRD	94.28141822	104.8209565	96.32491415	163.3622078	122.6537088	132.7918663
MRPL46	199.4796322	188.6777218	189.8442483	138.9932597	151.3111173	143.6926912
TAF1B	448.5810635	486.1695794	490.0413108	594.7828451	619.0000255	520.2666404
SEC61A2	139.9334734	141.757865	143.0845812	106.5013289	91.70370748	105.0443122

FAM189A2	10.91679579	18.96760166	13.09270678	26.1740554	43.55926105	25.765586
CNOT7	1293.144084	1362.672435	1399.984432	1523.510535	1548.64636	1587.556491
ISM2	10.91679579	8.984653418	6.546353389	2.707660903	1.146296344	0
OTUD5	998.3905971	916.4346487	874.405774	1005.444749	1180.685234	1114.857087
NPHP3	73.44026261	98.8311876	75.75066064	97.47579252	137.5555612	133.7828504
RIOK2	525.99107	554.0536275	603.1997051	717.5301394	651.0963231	652.0675226
ABHD14A	54.58397897	36.9369085	32.73176694	26.1740554	14.90185247	20.81066562
ZUP1	297.7307944	298.4901525	331.9936361	240.9818204	218.9426016	266.5747167
SEC16A	3382.221824	3680.713017	3658.476351	4205.899936	3844.677936	4106.638015
SGSM2	847.540328	852.5437799	841.6740071	756.3399456	717.581511	686.7519653
RARRES3	39.69743925	45.92156192	36.47254031	18.95362632	25.21851956	19.81968154
STX2	441.6340116	498.1491173	514.3563377	607.418596	558.2463193	592.608478

HOGA1	24.81089953	8.984653418	10.28712675	3.610214538	3.438889031	4.954920385
EPHB2	467.4373472	510.1286552	457.3095439	411.5644573	395.4722385	364.6821403
NEU4	27.78820747	33.94202402	28.05580024	51.44555716	52.7296318	52.52215608
ABCC2	39.69743925	35.93861367	45.82447372	17.14851905	13.75555612	29.72952231
TASOR	1585.912698	1833.867592	1856.358782	2054.212072	2071.357493	1976.022249
SERPING1	10.91679579	4.991474121	4.675966706	12.63575088	22.92592687	23.78361785
MFAP2	393.9970845	384.3435073	341.3455695	311.3810039	309.5000127	272.5206212
ADGRV1	408.8836243	403.311109	376.8829165	509.0402498	475.7129826	462.7895639
IBTK	909.0713588	1184.975956	1157.769356	1342.999808	1177.246345	1407.197389
UBE4A	427.7399079	552.0570378	620.0331852	688.6484231	604.098173	711.5265672
NIM1K	16.87141168	25.95566543	26.18541355	8.12298271	5.731481718	12.882793
TNFSF14	25.80333551	10.98124307	14.02790012	38.80980628	41.26666837	27.74755415

ACP2	472.3995271	477.184926	451.6983838	394.4159382	359.9370519	393.4206785
TOPBP1	1295.128955	1487.459288	1603.85658	1741.025961	1568.133398	1891.788603
RGS12	614.3178724	587.9956515	505.0044043	693.1611912	707.2648439	657.022443
ZNF266	215.3586079	252.5685905	257.1781688	307.7707893	271.6722334	357.7452518
LGALSL	105.198214	71.87722735	115.9639743	144.4085815	147.8722283	131.8008822
MPL	9.924359812	9.982948243	12.15751344	3.610214538	1.146296344	1.981968154
SLC16A12	30.76551542	14.97442236	28.05580024	60.47109351	40.12037202	40.63034715
DLGAP4	1166.112278	1021.255605	1038.064609	1199.49378	1326.264869	1247.648953
DIP2B	1157.180354	1523.397902	1403.725205	1700.411047	1415.675984	1845.212351
SREBF1	8114.156582	7022.005794	7169.192154	6405.423143	6889.241025	6254.100509
SCN4A	1.984871962	2.994884473	3.740773365	10.83064361	14.90185247	9.909840769
SCAMP1	1429.107813	1652.177934	1731.042875	1899.8754	1909.729708	1795.663147

WDR72	664.9321074	742.7313492	866.9242273	862.8412745	967.4741139	917.6512552
KLRC2	8.931923831	15.97271719	5.611160047	22.56384086	44.7055574	13.87377708
CYREN	247.1165593	226.6129251	215.0944685	185.0234951	170.7981552	170.4492612
MYDGF	1672.254628	1452.518969	1283.085264	1259.964874	1345.751907	993.9570291
FAM122A	245.1316874	263.5498336	291.7803225	355.606132	312.9389018	341.8895065
ASB16	34.73525934	31.94543438	38.34292699	21.66128723	13.75555612	15.85574523
SPG11	871.3587915	1038.226617	1009.073615	1272.600625	1044.275969	1162.424322
RUNDC3B	47.6369271	65.8874584	78.55624066	84.84004163	104.3129673	108.0172644
C6orf48	618.2876163	516.1184241	521.8378844	764.4629283	864.307443	517.2936881
MTA1	1770.50579	1741.026174	1578.60636	1943.197975	1938.387117	1947.283711
IL2RG	1.984871962	0	0	6.317875441	11.46296344	3.963936308
EXOSC5	552.7868415	479.1815156	422.7073902	406.1491355	372.5463116	390.4477263

RPS15A	2492.999185	2094.422541	1681.477628	1610.155684	1975.0686	1450.800689
TC2N	513.0894023	591.9888308	607.8756718	677.8177794	737.0685489	651.0765385
CYP27B1	27.78820747	27.95225508	24.31502687	37.90725265	53.87592815	53.51314015
DNAJC14	602.4086406	605.9649583	650.8945655	574.0241115	472.2740935	483.6002295
ATP6V0A4	33.74282336	28.9505499	34.60215363	12.63575088	12.60925978	20.81066562
PLCG2	569.6582532	574.0195239	606.0052851	474.7432117	482.5907606	503.4199111
KIF24	405.9063163	424.2753003	476.0134107	358.3137929	309.5000127	379.5469015
KIAA0319L	813.7975046	857.535254	856.6371006	765.365482	660.2666939	709.5445991
TIMP3	52.599107	32.9437292	45.82447372	16.24596542	32.09629762	19.81968154
SLC12A2	342.3904135	434.2582486	461.9855106	343.8729347	307.2074201	327.0247454
KLHDC2	513.0894023	417.2872365	461.0503172	390.8057237	372.5463116	364.6821403
TMED1	119.0923177	124.786853	124.3807144	92.96302434	90.55741114	73.33282169

BBS10	356.2845173	423.2770055	476.948604	553.2653779	526.1500217	483.6002295
GCA	319.564386	319.4543438	277.7524223	236.4690522	244.1611212	242.7910988
BCL3	1188.938305	1039.224912	988.4993617	1193.175905	1303.338943	1287.288316
SUMO4	0.992435981	2.994884473	0.935193341	6.317875441	11.46296344	9.909840769
FBXO41	511.1045303	493.1576432	481.6245707	661.571814	574.2944681	558.9150194
FXN	218.3359159	202.6538493	195.4554083	165.1673151	145.5796356	154.593516
PRR16	68.4780827	79.86358594	79.491434	101.0860071	113.483338	117.9271052
DUSP4	3712.703006	3811.489639	3477.984036	4351.211071	4063.620538	4029.341257
PHIP	924.9503345	1270.829311	1420.558685	1696.800833	1244.877829	1624.222902
HLA-E	3817.90122	3210.516155	3056.211839	2724.809422	3124.803832	2752.953766
C9orf116	30.76551542	41.92838262	40.21331367	13.53830452	20.63333418	22.79263377
CALML4	7.93948785	16.97101201	8.416740071	3.610214538	0	2.972952231

RMDN3	621.2649242	504.1388862	535.8657845	458.4972463	451.6407593	452.8797231
CCDC130	384.0727247	360.3844316	331.0584428	472.9381044	449.3481667	405.3124875
SLC16A4	15.8789757	40.93008779	40.21331367	9.025536344	8.024074405	22.79263377
TNIP2	910.0637948	735.7432855	762.1825731	968.4400497	1007.594486	901.79551
PEX5L	14.88653972	16.97101201	20.57425351	5.415321806	4.585185374	7.927872615
STIM1	92.29654625	122.7902634	96.32491415	138.0907061	149.0185247	149.6385956
QRFPR	92.29654625	78.86529112	84.16740071	60.47109351	32.09629762	64.413965
MT1X	340.4055416	258.5583595	176.7515415	216.6128723	168.5055625	158.5574523
EFCAB11	97.25872616	108.8141358	95.38972081	66.78896895	61.90000255	74.32380577
VIRMA	1395.36499	1520.403017	1600.115807	1713.949352	1709.127848	1775.843466
AQR	921.9730265	1023.252195	1053.027702	1210.324424	1080.957452	1225.847303
RASGRF2	25.80333551	24.95737061	50.50044043	57.7634326	59.60740986	62.43199685

CXCL11	18.85628364	21.96248613	32.73176694	8.12298271	12.60925978	9.909840769
AREL1	729.4404462	658.874584	731.3211928	791.5395374	824.187071	895.8496055
SLC16A11	1.984871962	3.993179297	2.805580024	12.63575088	12.60925978	9.909840769
HSPA4L	674.8564672	817.6034611	929.5821812	980.173247	899.8426297	1079.18166
TMEM139	1.984871962	0.998294824	1.870386682	10.83064361	10.31666709	5.945904461
APOL6	367.201313	503.1405914	453.5687705	370.0469901	331.2796433	348.8263951
RLIM	1112.520735	1317.749168	1413.077139	1596.617379	1338.874129	1676.745058
MIB1	550.8019696	708.7893252	667.7280456	570.4138969	400.0574239	549.9961627
PREP	958.6931579	1042.219797	1009.073615	1196.786119	1122.22412	1167.379243
NAA20	1067.861116	1125.078267	1010.008809	914.2868317	977.790781	830.4446565
LTC4S	20.84115561	14.97442236	24.31502687	6.317875441	10.31666709	5.945904461
TP53	1384.448194	1452.518969	1405.595592	1259.06232	1240.292644	1199.090733

MFSD9	216.3510439	171.7067098	161.788448	234.6639449	261.3555663	229.9083058
YES1	1686.148732	1834.865887	1952.683696	2209.451297	2004.872305	2092.95837
SNCB	13.89410374	15.97271719	14.02790012	6.317875441	1.146296344	4.954920385
FRG1	343.3828495	279.5225508	338.5399895	353.8010247	439.0314996	422.1592168
PIM2	220.3207878	184.6845425	177.6867348	154.3366715	139.8481539	139.7287548
SIGIRR	921.9730265	919.4295331	758.4417997	1039.741787	1108.468564	955.3086501
RPL14	7326.162413	6453.976039	6512.686428	5641.862769	6376.846559	5759.599455
MRPL49	2026.554274	1866.811321	1874.127456	2157.103186	2200.88898	2197.011699
ZNF672	208.4115561	247.5771164	245.0206554	168.7775296	178.8222296	189.2779587
SRPRB	614.3178724	546.0672689	573.2735182	637.2028659	750.824105	693.6888538
PSMD8	2730.191384	2289.090032	2246.334406	1887.23965	2247.88713	2049.355071
EFCAB14	1093.664451	1212.928211	1284.020458	1006.347302	1071.787081	992.9660451

GLRX3	2075.183637	1918.722652	1897.507289	2076.775913	2522.998252	2243.58795
TRIM15	3.969743925	4.991474121	0	15.34341178	9.170370748	12.882793
NSF	831.6613523	844.5574213	776.2104732	683.2331012	613.2685438	755.1298666
UTRN	605.3859485	805.6239232	807.0718535	877.2821326	812.7241076	1059.361978
FAF1	1004.345213	996.2982346	1100.722563	1147.145669	1223.098199	1233.775176
MTRR	1097.634195	1054.199334	1159.639743	1178.735047	1321.679684	1367.558026
CHST15	614.3178724	629.9240341	620.0331852	740.9965338	738.2148452	715.4905035
GLDC	26.79577149	19.96589649	33.66696028	51.44555716	49.29074277	44.59428346
MOB3C	415.8306761	439.2497227	430.188937	495.5019453	557.1000229	503.4199111
UST	85.34949438	119.7953789	112.2232009	151.6290106	146.725932	145.6746593
AGGF1	395.9819565	384.3435073	442.3464504	351.9959174	330.1333469	304.2321116
SRSF11	3438.790675	3672.726658	3843.644632	4031.707085	4061.327945	4300.870894

TMED2	5319.456859	5166.175716	5408.223092	4744.724456	4854.565015	4635.823512
DUSP28	104.205778	73.87381699	103.8064609	57.7634326	64.19259524	63.42298092
ENOSF1	1089.694707	1156.025406	1186.76035	968.4400497	997.2778189	1000.893918
NUDT16L1	753.2589097	519.1133086	495.6524708	480.1585335	484.8833533	412.249376
RPA4	5.954615887	7.986358594	5.611160047	17.14851905	17.19444515	19.81968154
SH2D3C	21.83359159	10.98124307	10.28712675	6.317875441	1.146296344	3.963936308
RAB5C	882.2755873	766.690425	691.1078792	683.2331012	671.7296573	563.8699398
ZNF329	103.213342	136.7663909	128.1214877	79.42471983	100.8740782	73.33282169
DCAF5	724.4782663	763.6955406	723.8396461	829.44679	866.6000357	894.8586215
MAMSTR	34.73525934	26.95396025	43.95408704	17.14851905	19.48703784	15.85574523
HOXB6	8.931923831	15.97271719	13.09270678	2.707660903	4.585185374	2.972952231
NOTCH4	7.93948785	11.97953789	9.351933412	0.902553634	4.585185374	0

GALNT3	1024.193933	1161.016881	1248.483111	944.9736552	994.9852262	981.0742361
ALPK1	374.1483649	385.3418022	434.8649037	483.768748	489.4685387	480.6272773
TBC1D22A	437.6642677	444.2411968	382.4940766	532.5066443	506.6629838	495.4920385
ABCD1	65.50077476	58.89939463	43.95408704	21.66128723	43.55926105	29.72952231
CEP85L	56.56885093	63.89086875	75.75066064	100.1834534	85.97222576	108.0172644
RNF122	82.37218644	88.84823936	75.75066064	120.0396334	113.483338	118.9180892
ARPC2	6987.741744	6347.158493	6445.352508	5320.553675	6018.055803	6059.86763
POLR1D	1552.169875	1515.411543	1421.493879	1611.058237	1790.514889	1763.951657
MVB12A	644.0909518	599.9751894	548.023298	515.3581252	503.2240948	466.7535002
FKBP14	351.3223373	374.3605591	439.5408704	481.9636408	448.2018703	507.3838474
MSTO1	473.391963	388.3366866	377.8181099	328.5295229	307.2074201	353.7813155
DMWD	1432.085121	1409.592292	1339.196865	1682.359975	1572.718583	1539.989256

AC093512.2	56.56885093	57.90109981	58.9171805	39.71235991	36.68148299	25.765586
CHD9	648.0606957	853.5420747	891.2392542	1011.762624	882.6481845	1028.641472
FBXL12	279.8669467	301.4850369	269.3356823	334.8473984	384.0092751	343.8714747
MST1	10.91679579	18.96760166	14.02790012	35.19959174	28.65740859	28.73853823
TPMT	641.1136439	646.8950461	653.7001455	539.7270734	567.41669	532.1584493
CALR	13401.85549	11782.87381	10878.16895	10136.57987	11182.12083	10017.85803
EXOC3L2	10.91679579	11.97953789	16.83348014	0.902553634	6.877778061	2.972952231
ARMC5	197.4947603	211.6385027	191.714635	274.3763049	281.9889005	222.9714173
DHX35	832.6537882	821.5966404	842.6092004	707.6020494	669.4370646	749.1839621
CES4A	145.8880892	103.8226617	122.5103277	77.61961256	90.55741114	90.179551
ADSSL1	385.0651607	365.3759057	280.5580024	269.8635367	291.1592713	238.8271625
ANKRD44	33.74282336	45.92156192	32.73176694	64.98386168	58.46111352	62.43199685

TDP2	737.379934	727.7569269	740.6731263	852.0106309	891.8185553	833.4176087
ATP9B	88.32680233	107.815841	108.4824276	137.1881524	136.4092649	150.6295797
FN1	156.804885	183.6862477	173.9459615	204.879675	222.3814906	247.7460192
PPP4R1	2524.757136	2789.235739	2480.132741	2874.633326	3004.442716	2971.961247
DYRK2	781.0471172	920.427828	896.8504142	789.7344301	665.9981756	718.4634558
BGLAP	1.984871962	0.998294824	1.870386682	9.928089978	5.731481718	10.90082485
HIST1H2BE	4.962179906	1.996589649	4.675966706	15.34341178	10.31666709	14.86476115
PRRG4	619.2800523	641.903572	741.6083196	538.8245197	605.2444694	514.3207359
RPL37A	11148.03338	9666.488783	7986.551134	7976.769021	8874.626292	7330.309217
DCTN5	1315.970111	1434.549662	1414.947525	1627.304203	1642.64266	1514.22367
LGALS9C	5.954615887	19.96589649	13.09270678	4.512768172	4.585185374	0
PLS3	9124.456411	9276.155507	9278.053138	8178.941035	8486.031831	8323.275262

ASIC1	235.2073275	192.6709011	204.8073417	170.5826369	154.7500064	153.6025319
CLEC17A	6.947051869	21.96248613	3.740773365	3.610214538	0	1.981968154
ONECUT2	26.79577149	32.9437292	40.21331367	55.95832533	56.16852083	57.47707646
CYSRT1	51.60667102	35.93861367	32.73176694	18.05107269	28.65740859	14.86476115
PSMD9	392.0122126	418.2855314	355.3734697	316.7963257	320.9629762	305.2230957
SARNP	3.969743925	5.989768946	5.611160047	17.14851905	14.90185247	14.86476115
IFI44L	325.5190018	368.3707902	432.994517	300.5503603	273.9648261	316.1239205
MFSD13A	169.7065528	167.7135305	148.6957413	124.5524015	113.483338	119.9090733
FAM83H	6618.555559	6596.732199	6056.312078	7415.38066	7697.379947	6725.80893
TMC6	1011.292265	1014.267541	861.3130673	867.3540427	812.7241076	751.1659303
FABP4	2.977307944	2.994884473	10.28712675	0	1.146296344	0
GRAMD1B	883.2680233	976.3323381	960.4435614	1089.382237	1044.275969	1145.577593

SLC25A23	670.8867233	701.8012615	637.8018587	755.437392	786.3592917	825.4897361
SLC44A5	230.2451476	389.3349815	332.9288295	445.8614954	390.8870531	408.2854397
MKS1	477.361707	425.2735951	421.7721969	358.3137929	349.6203848	375.5829651
GPM6B	43.66718317	33.94202402	41.14850701	61.37364714	74.50926233	58.46806054
CNTD2	8.931923831	6.98806377	8.416740071	18.05107269	17.19444515	26.75657008
LPCAT2	638.1363359	639.9069823	798.6551134	621.8594541	552.5148376	528.194513
WASHC2A	474.384399	444.2411968	492.8468908	534.3117516	562.8315047	599.5453665
ERICH2	6.947051869	4.991474121	5.611160047	10.83064361	22.92592687	17.83771338
DZANK1	24.81089953	28.9505499	23.37983353	52.3481108	52.7296318	34.68444269
TMEM232	3.969743925	7.986358594	9.351933412	15.34341178	26.3648159	16.84672931
DCLRE1A	360.2542612	368.3707902	343.2159562	282.4992876	247.6000102	322.069825
BCAS2	963.6553378	879.4977402	899.6559943	990.1013369	1205.903753	1042.515249

NDUFS7	334.4509257	281.5191404	261.8541355	231.0537304	202.8944528	246.7550352
OTUB2	80.38731448	104.8209565	89.77856076	55.0557717	56.16852083	70.35986946
GNAS	19181.80264	17223.5806	16583.78352	14874.98645	15615.99509	16368.084
SARAF	2022.58453	2057.485633	2063.971704	1739.220854	1957.874155	1653.952424
PDIA5	399.9517004	400.3162245	357.2438563	322.2116475	324.4018652	290.3583345
ZNF516	410.8684962	477.184926	412.4202635	551.4602706	484.8833533	542.0682901
VTI1A	365.2164411	365.3759057	418.0314235	454.8870317	486.0296497	458.8256276
MAP3K3	413.8458042	374.3605591	376.8829165	338.4576129	298.0370493	306.2140798
ARMH3	545.8397897	533.0894362	525.5786578	633.5926514	667.1444719	604.5002869
GPKOW	482.3238869	466.2036829	395.5867833	380.8776337	365.6685336	344.8624588
ZHX3	955.7158499	925.4193021	952.0268214	1138.120133	972.0592993	1233.775176
SDHA	494.2331186	432.2616589	365.6605964	346.5805956	333.572236	353.7813155

LIAS	323.5341299	349.4031885	355.3734697	301.4529139	257.9166773	262.6107804
RAVER2	556.7565855	645.8967513	595.7181584	685.9407621	680.900028	781.8864367
GPD1L	327.5038738	289.505499	323.5768961	453.9844781	353.0592738	370.6280448
SHH	10.91679579	17.96930684	7.48154673	21.66128723	32.09629762	28.73853823
GATA3	9.924359812	9.982948243	5.611160047	0	1.146296344	2.972952231
EPS15L1	465.4524752	484.1729898	560.1808114	591.1726305	601.8055803	623.3289844
CPLANE2	83.36462242	74.87211182	51.43563377	50.54300353	25.21851956	46.57625161
GTF3A	1083.740091	1022.2539	1087.629856	857.4259527	904.427815	981.0742361
TBCK	221.3132238	191.6726063	208.5481151	261.740554	231.5518614	312.1599842
DMC1	7.93948785	4.991474121	2.805580024	0	0	0.990984077
C7orf31	84.3570584	95.83630313	89.77856076	111.9166507	160.4814881	115.945137
PRMT1	5791.856386	5145.211524	4730.20792	4352.113625	4717.009454	4597.175133

ATP5ME	318.57195	241.5873475	132.7974545	119.1370797	121.5074124	48.55821977
MIOX	2.977307944	0	3.740773365	8.12298271	11.46296344	11.89180892
GK	151.8427051	179.6930684	185.1682816	241.884374	212.0648236	219.9984651
C19orf44	88.32680233	88.84823936	77.62104732	100.1834534	116.922227	152.6115478
C6orf120	377.1256729	453.2258502	508.7451776	544.2398415	565.1240974	535.1314015
FBXO3	877.3134074	929.4124814	967.9251082	1041.546894	1110.761157	1065.307883
CFAP410	56.56885093	58.89939463	40.21331367	91.15791708	84.82592942	67.38691723
ZNF232	122.0696257	108.8141358	114.0935876	93.86557798	77.94815136	66.39593315
PAX8	161.7670649	194.6674907	193.5850216	263.5456612	202.8944528	253.6919237
PRPSAP1	968.6175177	851.5454851	834.1924604	744.6067484	777.1889209	735.3101851
BCL2L15	10.91679579	7.986358594	3.740773365	1.805107269	0	0.990984077
GLTPD2	16.87141168	14.97442236	23.37983353	6.317875441	9.170370748	4.954920385

AP003108.2	17.86384766	12.97783272	12.15751344	35.19959174	24.07222321	30.72050638
SLC30A4	51.60667102	56.90280498	62.65795386	44.22512809	25.21851956	29.72952231
NIPAL3	601.4162046	663.8660581	651.8297588	574.9266651	468.8352045	547.0232105
TGM2	1064.883808	1056.195924	883.7577075	850.2055236	825.3333673	872.0659877
CARD8	96.26629018	109.8124307	86.03778739	143.5060279	127.2388941	135.7648185
ISOC1	720.5085224	725.7603372	699.5246192	564.0960215	624.7315072	630.2658729
SP5	21.83359159	15.97271719	19.63906017	9.025536344	5.731481718	6.936888538
DCST1	14.88653972	20.96419131	16.83348014	6.317875441	4.585185374	7.927872615
LHFPL5	84.3570584	72.87552217	109.4176209	49.64044989	46.99815008	73.33282169
ACADSB	110.1603939	144.7527495	162.7236414	111.014097	75.65555867	102.0713599
GSG1L	7.93948785	2.994884473	4.675966706	0.902553634	0	0
SRA1	939.8368742	841.5625368	856.6371006	1015.372839	1055.738932	995.9389973

MKRN2	621.2649242	658.874584	631.2555053	694.9662985	784.066699	777.9225004
STK31	5.954615887	7.986358594	8.416740071	0	1.146296344	1.981968154
COX7C	5553.671751	4636.081164	4030.683301	3853.904019	4219.51684	4004.566655
PPP3CA	897.162127	1021.255605	1037.129415	1285.236375	1115.346342	1081.163628
EPN3	302.6929743	253.5668854	270.2708756	237.3716058	199.4555638	208.1066562
TMEM19	245.1316874	284.5140249	326.3824761	256.3252322	206.3333418	191.2599268
ZNF513	335.4433617	319.4543438	333.8640228	426.9078691	420.6907581	366.6641085
SLC38A6	57.56128691	61.8942791	57.04679381	77.61961256	89.41111479	98.10742361
ANAPC4	574.6204331	547.0655637	640.6074387	495.5019453	475.7129826	499.4559748
NOP58	1894.560288	1872.80109	2006.92491	2158.908294	2247.88713	2129.624781
TUBAL3	17.86384766	16.97101201	13.09270678	3.610214538	6.877778061	5.945904461
ESYT2	1785.39233	1850.838604	1811.469502	2045.186536	2031.237121	2067.192784

VAT1	3035.861667	2750.302241	2449.271361	3145.399416	3171.801983	3143.401492
PMM2	362.2391331	413.2940572	427.3833569	365.5342219	324.4018652	260.6288122
CAMLG	612.3330004	510.1286552	526.5138511	623.6645614	785.2129953	611.4371755
ZNF713	41.68231121	37.93520332	30.86138026	56.86087897	56.16852083	68.37790131
EFHD1	40.68987523	30.94713955	27.1206069	47.83534262	57.31481718	62.43199685
ERCC3	1076.79304	1035.231733	1095.111403	905.2612953	922.7685565	953.326682
TP53I3	86.34193037	96.83459795	79.491434	109.2089898	160.4814881	110.9902166
ZNF692	465.4524752	398.3196349	423.6425836	363.7291147	317.5240872	365.6731244
CYP2J2	53.59154299	53.90792051	47.6948604	77.61961256	65.33889158	97.11643954
ZSCAN26	153.8275771	180.6913632	156.177288	225.6384086	197.1629711	217.0255128
SRGAP2	670.8867233	745.7262337	721.9692594	870.0617036	756.5555867	901.79551
CTSD	1014.269573	954.369852	932.3877612	829.44679	920.4759638	635.2207933

IGFBP2	107.183086	107.815841	88.84336742	80.32727346	73.36296599	47.56723569
TLE4	405.9063163	350.4014833	391.84601	324.0167548	269.3796407	328.0157295
BAG5	938.8444382	914.438059	981.0178149	1122.776721	1032.813006	1120.802991
C2orf68	758.2210896	727.7569269	786.4976	620.9569005	656.8278048	660.9863793
DPM1	1807.225922	1688.116548	1819.886242	1431.450064	1659.837105	1538.998271
GLB1	772.1151934	760.7006561	679.8855591	601.1007205	652.2426195	620.3560321
NBPF11	222.3056598	269.5396025	263.7245222	299.6478066	367.9611263	295.3132549
FAM20C	112.1452659	85.85335489	97.26010749	51.44555716	68.77778061	77.296758
UBIAD1	309.6400261	387.3383918	278.6876157	250.0073567	265.9407517	247.7460192
SLC26A1	14.88653972	15.97271719	16.83348014	4.512768172	8.024074405	3.963936308
ABCA7	1144.278686	1165.01006	1023.101515	975.6604788	808.1389222	1021.704583
CLHC1	95.2738542	96.83459795	110.3528143	127.2600625	142.1407466	148.6476115

ARHGAP20	10.91679579	7.986358594	6.546353389	19.85617996	21.77963053	20.81066562
C11orf58	3008.073459	2958.945859	3072.110126	3315.982053	3563.835332	3275.202374
NCF1	21.83359159	15.97271719	12.15751344	28.8817163	40.12037202	31.71149046
RDM1	116.1150098	96.83459795	101.9360742	71.30173712	83.67963308	61.44101277
GVQW2	19.84871962	18.96760166	16.83348014	5.415321806	6.877778061	8.918856692
HCN2	90.31167429	92.84141866	69.20430725	55.95832533	60.75370621	48.55821977
RPS6KL1	40.68987523	34.94031885	45.82447372	22.56384086	21.77963053	21.80164969
SMARCC2	3354.433617	3449.108618	3343.316195	3009.113817	2798.109375	3180.067903
PAK1	1290.166776	1283.807144	1187.695543	1096.602666	1072.933378	1105.93823
RFTN1	42.67474719	55.90451016	52.37082711	76.71705892	77.94815136	77.296758
TAF10	366.2088771	348.4048937	339.4751829	341.1652738	264.7944554	183.3320542
ELF4	913.0411027	941.3920193	942.674888	1089.382237	1007.594486	1139.631688

FAM72B	201.4645042	203.6521441	201.0665684	161.5571006	144.4333393	158.5574523
GPR35	1.984871962	1.996589649	0	7.220429075	6.877778061	9.909840769
STT3B	2284.587629	2462.793331	2670.912183	2804.234142	2768.30567	2862.952998
TARSL2	228.2602757	205.6487338	215.0944685	153.4341178	173.0907479	174.4131975
SNPH	67.48564672	56.90280498	52.37082711	35.19959174	37.82777934	34.68444269
RAMP1	28.78064346	4.991474121	27.1206069	4.512768172	8.024074405	7.927872615
NOP53	3113.271673	2589.576774	2398.77092	3098.466627	3505.374218	2930.339915
PURA	437.6642677	439.2497227	491.9116975	566.8036824	562.8315047	514.3207359
PPP2R3B	309.6400261	281.5191404	259.0485555	246.3971422	199.4555638	219.007481
SRPX	149.8578332	132.7732116	115.9639743	102.8911143	87.11852211	94.14348731
TCEAL1	239.1770715	248.5754112	218.8352418	181.4132805	205.1870455	158.5574523
PIWIL2	1.984871962	2.994884473	6.546353389	15.34341178	11.46296344	12.882793

SLC38A7	581.567485	534.087731	495.6524708	636.3003123	686.6315098	603.5093028
RP1	8.931923831	5.989768946	2.805580024	22.56384086	12.60925978	15.85574523
CSPG4	38.70500327	43.92497227	43.95408704	76.71705892	59.60740986	64.413965
RFXANK	548.8170976	488.1661691	456.3743505	365.5342219	482.5907606	347.835411
SPATA20	772.1151934	642.9018668	688.3022991	607.418596	614.4148401	537.1133697
RBM12B	479.3465789	592.9871256	700.4598126	552.3628243	358.7907555	462.7895639
RBMX2	289.7913065	316.4594593	337.6047962	392.610831	459.6648338	335.9436021
ATPAF1	718.5236504	715.777389	728.5156128	574.9266651	640.779656	626.3019366
MAP2K5	146.8805252	105.8192514	102.8712675	160.6545469	159.3351917	167.476309
ODF2	1413.228837	1263.841248	1399.984432	1131.802258	1188.709308	1221.883367
LRFN3	263.987971	239.5907578	225.3815952	196.7566923	167.3592662	200.1787835
TMEM184B	3153.961548	3034.816266	3027.220846	3492.882565	3551.226072	3289.076151

GBP4	19.84871962	10.98124307	24.31502687	9.025536344	3.438889031	6.936888538
ISY1	290.7837425	228.6095148	251.5670088	317.6988793	338.1574213	313.1509683
SMN2	308.6475902	306.476511	316.0953493	385.3904019	377.131497	373.600997
AMOT	42.67474719	38.93349815	43.95408704	17.14851905	24.07222321	27.74755415
WBP2	2516.817648	2133.356039	2014.406457	1816.840466	2120.648236	1717.375405
RABIF	255.0560472	204.650439	207.6129218	277.0839658	287.7203822	282.4304619
SCPEP1	714.5539065	736.7415803	646.2185988	628.1773295	592.6352096	542.0682901
C17orf107	9.924359812	10.98124307	8.416740071	28.8817163	20.63333418	18.82869746
GMPPA	705.6219826	603.9683687	508.7451776	484.6713017	527.296318	468.7354684
DUS3L	998.3905971	928.4141866	847.2851672	772.5859111	836.7963308	753.1478985
RING1	1339.788575	1136.05951	1008.138422	925.1174753	1016.764857	987.0201406
NAT9	1020.224189	929.4124814	737.8675462	1087.577129	1160.0519	993.9570291

TTC31	431.7096518	392.3298659	414.2906502	323.1142011	349.6203848	345.8534428
MRE11	1005.337649	1084.148179	1185.825157	938.6557798	943.4018907	936.4799527
CPM	446.5961915	466.2036829	478.8189907	511.7479107	565.1240974	590.6265098
C2orf16	22.82602757	41.92838262	32.73176694	63.17875441	67.63148427	39.63936308
TWF1	2042.433249	2358.97067	2519.410861	2522.637408	2740.794557	2720.251291
CAPN12	78.40244252	67.88404805	61.72276052	79.42471983	121.5074124	109.0082485
PHF1	598.4388967	504.1388862	540.5417512	657.9615995	690.0703988	613.4191436
NAGA	434.6869598	399.3179297	375.9477232	350.1908101	302.6222347	330.9886817
TTC33	376.1332369	443.242902	466.6614773	583.0496478	497.4926131	491.5281021
RPL13	14808.13728	12521.61198	11585.17511	14992.31842	16521.5692	13730.08439
HACD2	1320.932291	1370.658794	1631.91238	1704.923815	1584.181547	1752.059848
CAPZB	3010.058331	2733.331229	2568.976108	2231.112584	2638.774183	2338.722422

TRIM69	212.3813	215.631682	206.6777284	250.9099104	257.9166773	295.3132549
MDH1	1897.537596	1749.012532	1832.043755	1634.524632	1654.105624	1530.079415
PTPN18	391.0197766	388.3366866	380.6236899	261.740554	345.0351994	328.0157295
ARF1	7117.750857	6387.090286	6280.75848	5584.099336	5869.037279	6099.506993
MCIDAS	60.53859485	48.91644639	60.78756718	38.80980628	24.07222321	37.65739492
ARHGAP18	235.2073275	258.5583595	266.5301022	332.1397375	281.9889005	337.9255702
NR1I3	5.954615887	2.994884473	0.935193341	10.83064361	9.170370748	16.84672931
NDUFB11	1042.05778	919.4295331	801.4606934	716.6275857	800.1148478	797.7421819
LRRC8A	4105.707654	3909.322532	3599.55917	4261.858262	4542.772409	4294.924989
ANKRD36B	99.24359812	155.7339926	148.6957413	81.2298271	82.53333673	113.9631688
DUSP22	289.7913065	261.553244	298.3266759	244.5920349	222.3814906	204.1427198
LYPLAL1	356.2845173	365.3759057	376.8829165	420.5899936	497.4926131	422.1592168

MIEN1	499.1952986	451.2292606	424.5777769	500.9172671	632.7555816	543.0592741

Table S5 RT-PCR Primer sequences,

related to Fig. 2c-d, S3c, S3e, S3g, S3i and Fig. S5b-c

Gene Symbol	Species	Primer sequence	
		Forward	Reverse
GAPDH	Human	ACATCGCTCAGACACCATG	TGTAGTTGAGGTCAATGAAGGG
	Mouse	CAAGTGTCAGGACGAACAGAG	CAAGTGTCAGGACGAACAGAG
FOS	Human	TTGTGAAGACCATGACAGGAG	CCATCTTATTCCTTTCCCTTCGG
	Mouse	CCTTTGTCTTCACCTACCCTG	CTTGCCTTCTCTGACTGCTC
FOSB	Human	AGCTAAATGCAGGAACCGG	ACCAGCACAAACTCCAGAC
	Mouse	AGTCTCAGTACCTGTCTTCGG	CACGAGCCACTGAAGATCC
JUN	Human	AGCCCAAACTAACCTCACG	TGCTCTGTTTCAGGATCTTGG
	Mouse	TTGTTACAGAAGCAGGGACG	GTCGTAGAAGGTCGTTTCCATC
JUNB	Human	GGACACGCCTTCTGAACG	CGGAGTCCAGTGTGGTTTG
	Mouse	GGACACGCCTTCTGAGAG	GAGTCCAGTGTGTGAGCTG
MYC	Human	TTCGGGTAGTGGAAAACCAG	AGTAGAAATACGGCTGCACC
	Mouse	GCTGTTTGAAGGCTGGATTTC	ATGAAATAGGGCTGTACGGAG
PIM1	Human	CGACATCAAGGACGAAAACATC	ACTCTGGAGGGCTATACACTC
	Mouse	GCTCAAGGACACAGTCTACAC	GCTCAAGGACACAGTCTACAC
BCL2L1	Human	GACATCCCAGCTCCACATC	GTTCCCATAGAGTTCCACAAAAG
	Mouse	GGAAAGCGTAGACAAGGAGATG	GCATTGTTCCCGTAGAGATCC
VEGF	Human	AGTCCAACATCACCATGCAG	TTCCCTTTCCTCGAACTGATTT
	Mouse	GGCAGCTTGAGTTAAACGAAC	TGGTGACATGGTTAATCGGTC
MMP-28	Human	CCGTGCAGAGCCTGTATG	GGAAGAGTGGCAGTATTTAGGG
	Mouse	TGGATGGGCTGTTGGAATG	GTCCTTACTGCTCAAGAGGTTC

TGFB	Human	CCTGAGTGGCTGTCTTTTGA	CGTGGAGTTTGTTATCTTTGCTG
	Mouse	CCTGAGTGGCTGTCTTTTGA	CGTGGAGTTTGTTATCTTTGCTG
TGFA	Human	ACACTCAGTTCTGCTTCCATG	GCACCAACGTACCCAGAATG
	Mouse	CTGGGTATCCTGTTAGCTGTG	GTACTGAGTGTGGGAATCTGG
EGFR	Human	AAGCCATATGACGGAATCCC	GGAACTTTGGGCGACTATCTG
	Mouse	ATCAAAGTTCTGGGTTCGGG	GTTGGCTTTTGGAGATGTGG

Table S6 Primer sequences for ChIP, related to Fig. 4i and S10c

Gene Symbol	Species	Primer sequence	
		Forward	Reverse
MMP-28	Human	TCCCTAGTCCAGGTCAAGCC	TCCTGCCCTCCCTTTGTAGA
	Mouse	TCCTTCTCTTGCCTTACTGCTG	TCCAGACTTGCAGGGATGTTC
